# Chlorpromazine induces cytotoxic autophagy in glioblastoma cells via endoplasmic reticulum stress and unfolded protein response

**DOI:** 10.1186/s13046-021-02144-w

**Published:** 2021-11-05

**Authors:** Silvia Matteoni, Paola Matarrese, Barbara Ascione, Lucia Ricci-Vitiani, Roberto Pallini, Veronica Villani, Andrea Pace, Marco G. Paggi, Claudia Abbruzzese

**Affiliations:** 1 Cellular Networks and Molecular Therapeutic Targets, Proteomics Unit, IRCCS - Regina Elena National Cancer Institute, 00144 Rome, Italy; 2 Department of Therapeutic Research and Medicines Evaluation, Istituto Superiore di Sanità, 00161 Rome, Italy; 3 Department of Oncology and Molecular Medicine, Istituto Superiore di Sanità, 00161 Rome, Italy; 4 Institute of Neurosurgery, Fondazione Policlinico Universitario A. Gemelli IRCCS, Catholic University School of Medicine, 00168 Rome, Italy; 5 Neuro-Oncology, IRCCS - Regina Elena National Cancer Institute, 00144 Rome, Italy

**Keywords:** Glioblastoma, Drug repurposing, Chlorpromazine, Endoplasmic reticulum stress, Unfolded protein response, Autophagy, Mitotic catastrophe, Cell death

## Abstract

**Background:**

Glioblastoma (GBM; grade IV glioma) is characterized by a very short overall survival time and extremely low 5-year survival rates. We intend to promote experimental and clinical research on rationale and scientifically driven drug repurposing. This may represent a safe and often inexpensive way to propose novel pharmacological approaches to GBM. Our precedent work describes the role of chlorpromazine (CPZ) in hindering malignant features of GBM. Here, we investigate in greater detail the molecular mechanisms at the basis of the effect of CPZ on GBM cells.

**Methods:**

We employed proteomics platforms, i.e., activity-based protein profiling plus mass spectrometry, to identify potential cellular targets of the drug. Then, by means of established molecular and cellular biology techniques, we assessed the effects of this drug on GBM cell metabolic and survival pathways.

**Results:**

The experimental output indicated as putative targets of CPZ several of factors implicated in endoplasmic reticulum (ER) stress, with consequent unfolded protein response (UPR). Such a perturbation culminated in a noticeable reactive oxygen species generation and intense autophagic response that resulted in cytotoxic and abortive effects for six GBM cell lines, three of which growing as neurospheres, while it appeared cytoprotective for the RPE-1 human non-cancer neuro-ectodermal cell line.

**Conclusions:**

This discrepancy could be central in explaining the lethal effects of the drug on GBM cells and the relatively scarce cytotoxicity toward normal tissues attributed to this compound. The data presented here offer support to the multicenter phase II clinical trial we have undertaken, which consists of the addition of CPZ to first-line treatment of GBM patients carrying a hypo- or un-methylated *MGMT* gene, i.e. those characterized by intrinsic resistance to temozolomide.

**Supplementary Information:**

The online version contains supplementary material available at 10.1186/s13046-021-02144-w.

## Background

Glioblastoma (GBM; grade IV glioma) is the most frequent and lethal brain tumor in adulthood. The first-line therapeutic approach for newly diagnosed GBM patients consists of surgical ablation followed by radio-chemotherapy with temozolomide (TMZ), plus adjuvant chemotherapy using TMZ alone [1]. This state-of-art therapeutic schedule, substantially unchanged since 2005, is still characterized by a quite adverse prognosis, with an overall survival of 15.6 months and 5-year survival for <5 % of patients [2, 3]. In the effort to identify better therapeutic approaches, besides the experimentation of novel compounds, drug repurposing/repositioning, when scientifically sound, is also widely considered, since this approach is characterized by a safer, faster and less expensive transition from bench to bedside [4]. Among old drugs amenable of repurposing in GBM therapy, we focused our attention on the neuroleptic, antipsychotic medication chlorpromazine (CPZ).

CPZ is a phenothiazine derivative used for over 60 years in psychiatry, mainly in schizophrenia and bipolar disorders. In these diseases, the role of CPZ is to antagonize the CNS dopamine receptor D2 (DRD2), thus decreasing the post-synaptic effect of dopamine [5, 6]. More recently, this drug has also been described as active *in vitro* toward several biological features, hindering the survival capabilities of cancer cells, especially those of GBM [7–16]. Interestingly, CPZ synergizes with TMZ in reducing GBM cell viability, while both drugs cooperate in diminishing cloning efficiency and inducing cell death [17]. Our group is currently involved in exploring the molecular and cellular bases of a possible anticancer effect of CPZ in GBM, exploring the ability of this compound in inhibiting cell viability in an apoptosis-independent manner, inducing hyperdiploidy, reducing cloning efficiency and downregulating the expression of stemness genes in either anchorage-dependent GBM cells or neurospheres *in vitro*.

To delve into the molecular mechanisms of CPZ pharmacodynamics properties in GBM cells, we performed activity-based protein profiling (ABPP) determinations. We operated a kinase enrichment procedure by means of an ATP probe [18, 19]. This technique, coupled with a mass spectrometry (MS) platform, allowed to detect cellular factors whose nucleotide-binding ability appeared modified by CPZ. The results drove our attention toward some factors involved in the endoplasmic reticulum (ER) stress and unfolded protein response (UPR).

ER plays an essential role in protein biosynthesis and homeostasis. Under stress conditions, such as hypoxia, nutrient deprivation, or other pathological conditions, a cellular perturbation (ER stress) occurs, with the consequent accumulation of unfolded or misfolded proteins [20]. In the attempt to restore the physiological conditions, cells activate UPR, a process that sequentially triggers different signal transduction pathways from ER to the nucleus, through the activation, via auto-phosphorylation or proteolytic cleavage, of the main sensors, i.e. IRE1, PERK and ATF6-α, devoted to promoting UPR [21, 22]. UPR plays a double-faceted role: it can restore the correct folding of unfolded/misfolded proteins, thus recovering ER homeostasis and allowing cell survival and, in case of failure in resolving ER stress, UPR triggers damages in cellular functions, thus switching from a salvage program to the induction of piloted cell death, aiming at eliminating irreversibly injured cell [23, 24, 21, 25]. Recently, several investigations demonstrate a fine interplay between UPR and the induction or inhibition of autophagy [26]. Indeed, the three main UPR sensors (PERK, IRE1 and ATF6-α) are involved in inducing an autophagic response [27, 28, 21, 29], which, in turn, can determine cell fate.

Here, we describe how CPZ interferes with ER stress and UPR, thus modifying, via autophagy, the cell fate of GBM cells.

## Methods

### Cell lines

Anchorage-dependent cell lines T98G, U-251 MG and U-87 MG were cultured as previously reported [30]. Anchorage-independent TS#1, TS#83 and TS#163 are patient-derived cell lines from surgical samples classified according to the WHO [31] cultured, as described [32–34] and defined as glioma stem cells growing as neurospheres [35]. The human hTERT-immortalized retinal pigment epithelial cell line hTERT RPE-1 (henceforth RPE-1) [36] was courtesy of Giulia Guarguaglini, CNR, Rome, Italy.

T98G, U-251 MG and U-87 MG were from the laboratory of one of the authors (LRV) and authenticated via short tandem repeat (STR) profiling.

All cell lines were mycoplasma-free and used for a maximum of 20 passages.

### Drugs

CPZ was purchased, as “Largactil”, from Teofarma S.R.L., Valle Salimbene (PV), Italy, as a 25 mg/ml solution (78 mM). 4-phenylbutyrate (4-PBA) was purchased as a powder from Sigma-Aldrich Merck KGaA (Darmstadt, Germany) and dissolved in DMSO as a 1 M stock solution.

### Identification of potential CPZ protein targets

To identify potential cellular targets of CPZ, we employed ABPP, in a competitive mode. Multiple aliquots of the same native GBM cell lysates were incubated with increasing concentrations of CPZ (ranging from 5 to 40 µM) and then mixed with the ATP-mimicking insoluble probe according to the kinase enrichment kit protocol provided by the manufacturer (Thermo Fisher Scientific, Waltham, MA, USA). Pulled-down proteins were separated *via* 10 % SDS-PAGE, stained with Imperial protein stain (Thermo Fisher Scientific); then, proteins whose ATP-binding ability was influenced by the drug were picked and identified by MALDI-MS and MS/MS analysis. All these procedures have been performed as described previously [37].

### RNA extraction and RT-PCR

All GBM cell lines and RPE-1 cells were treated with an amount of CPZ corresponding to their respective IC30 dose (Table S7) for 24 h, while control samples were treated with the same final concentration of solvent (phosphate-buffered saline [PBS]). RNA extraction and ER stress gene expression were performed as previously described [17]. RT-PCR analyses were quantified using the 2^−∆∆^CT method. Values represent the fold changes related to control cells, arbitrarily reported as 1.0.

### Fluorescence microscopy

For fluorescence microscopy data, we plated neurospheres in 35-mm dishes, treated cells with CPZ (IC30) or solvent for controls and cytocentrifuge them on a slide. Anchorage-dependent GBM cells were grown directly on a coverslip, treated with the drug, fixed in 4 % paraformaldehyde and submitted to the specific analyses.

#### ATF6-α subcellular localization

After treatment of GBM cells with CPZ, we performed immunofluorescence analysis using an anti-ATF6-α primary antibody (Novus Biologicals), incubated for 2 h at room temperature and AlexaFluor 594-conjugated anti‐mouse IgG (Invitrogen) as a secondary antibody.

#### Detection of ROS generation

For a qualitative analysis of intracellular ROS production, we assessed the dihydroethidium (DHE) staining, a cell-permeable fluorophore able to detect superoxide anion levels by red fluorescence emission. Briefly, anchorage-dependent GBM cells and RPE-1 cells were plated, treated with IC30 CPZ or solvent for 24 h and then exposed to 1 µM DHE for 5 min; fluorescent products were analyzed using an inverted fluorescence microscope.

#### Detection of mitotic catastrophe

To investigate peculiar nuclear features, distinctive traits of mitotic catastrophe, we treated GBM cells with IC30 CPZ or the same amount of PBS and performed immunofluorescence analysis using, as a primary antibody, an anti-α-tubulin monoclonal antibody (Calbiochem, San Diego, CA, USA); after washing, all samples were counterstained with Hoechst 33,342 (Sigma-Aldrich, St Louis, MO, USA; 1 mg/ml in PBS) and then mounted in glycerol/PBS pH 7.4.

#### Detection of nuclear morphology

All cell lines, untreated or treated with CPZ and 4-PBA, alone or in combination, were stained with Hoechst 33,342. Images were acquired by using an Olympus BX53 Fluorescence Microscope (Olympus Corporation of the Americas, Center Valley, PA, USA) and aberrant nuclei were counted.

### Immunoblot analysis

Autophagic markers LC3 and p62 were evaluated by western blotting using anti-LC3 (MBL) and anti-p62 (Sigma-Aldrich) antibodies.

Nuclear and cytoplasmic extracts were prepared using NE-PER Nuclear and Cytoplasmic Extraction Reagents (Thermo Scientific, Rockford, IL, USA) following the manufacturer’s instructions. Subcellular protein fractions were then separated on 4–12 % gradient gels (Invitrogen) by SDS-polyacrylamide gel electrophoresis, transferred to PVDF membranes and incubated with mouse monoclonal anti-ATF6-α antibody (Novus Biologicals; 1:1000). The purity of protein fractions, such as the relative protein expression of target in each compartment, was evaluated using anti-H3 histone (Invitrogen; 1:4000) and anti-GAPDH (Sigma-Aldrich; 1:20,000) as nuclear and cytoplasmic markers, respectively.

### FACS analyses

#### ROS generation

For a quantitative analysis of intracellular ROS production, we stained cells with 1 µM DHE for 15 min. After washing in PBS cells were immediately analyzed by a cytometer. Quantification of ROS was obtained by using the median fluorescence intensity of the cytometer curves.

*Autophagy detection.* Cells untreated or treated with CPZ and 4-PBA, alone or in combination, were fixed with 4 % paraformaldehyde (Carlo Erba, Milano, Italia) in PBS for 30 min at room temperature and then permeabilized by 0.5 % Triton X-100 (Sigma-Aldrich) in PBS for 5 min at room temperature. After washings, cells were incubated with anti-LC3 (mouse, Invitrogen) and anti-p62/SQSTM1 (rabbit, Sigma-Aldrich) primary antibodies for 1 h, followed by anti-mouse Alexa fluor488 (Invitrogen) and anti-rabbit CY5-conjugated (Abcam) for an additional 45 min at 37 °C. After washing, cells were resuspended in PBS and analyzed on a cytometer. The expression levels of the analyzed proteins were quantified by using the median fluorescence intensity of the cytometer curves.

All samples were acquired and analyzed using a FACSCalibur flow cytometer (BD Biosciences, San Jose, CA, USA) equipped with a 488 nm argon laser and with a 635 nm red diode laser. At least 20,000 events/sample were acquired and analyzed using the Cell Quest Pro software (BD Biosciences).

### Statistical analysis

All tests were done in triplicate and experiments were performed at least three-times. Results are expressed as a mean ± standard error (SE). Differences between each group and relative control were analyzed using the Student’s two-tailed t-test (Prism v5, GraphPad Software Inc., San Diego, CA, USA). Asterisks denote statistical significance (*p<0.05; **p<0.01; ***p<0.001).

## Results

Identification of putative molecular targets of CPZ

To explore the role of CPZ in restraining GBM growth, we employed proteomics techniques and platforms to possibly identify novel molecular targets of this drug. With this aim, we performed ABPP, coupled with MS analysis [18, 19], employing either anchorage-dependent GBM cells or anchorage-independent, patient-derived, GBM cells enriched for their stemness capabilities (neurospheres). Briefly, after a kinase enrichment procedure via an insoluble ATP probe, we isolated and identified some protein factors whose ability to bind ATP appeared modified by the presence of CPZ. A list of identified proteins is reported in Table 1. Raw data are available as Additional File 1 (see Supplementary Material, AF1_MASCOT IDs). Among these ATP- or GTP-binding proteins, we focused our attention on the following: ER chaperone BiP [38–40]; endoplasmin [41, 42]; heat shock protein HSP 90-beta [43]; members of T-complex protein 1 (TRiC) [44, 45], i.e. TCP1, CCT5 and CCT6; heat shock protein 75 kDa [46]; EF1-α1 [47]; EF2 [48], substrate of eEF2K [49, 50]; and TER ATPase [51].

**Table 1:** GBM proteins identified as potential CPZ targets

Protein Name	Gene Name	UNIPROT Protein AC	Main Cellular Localization	Molecular function	Biological role in UPR
BiP	*HSPA5*	P11021	ER- Cytoplasm	Molecular chaperone	Master regulator of UPR [38–40]
Endoplasmin	*HSP90B1*	P14625	ER lumen	Molecular chaperone	Protein folding [41]; ER-associated degradation (ERAD) [42]
Heat shock protein HSP 90-beta	*HSP90AB1*	P08238	Cell membrane – Nucleus –Cytoplasm - Secreted	Molecular chaperone	Protein folding [43]
T-complex protein 1 (TRiC)	*TCP1* (subunit alpha)	P17987	Cytoskeleton; cytosol	Subunits of chaperone complex TRiC	Protein folding [44, 45]
	*CCT5* (subunit epsilon)	P48643	Cytoskeleton; cytoplasm		
	*CCT6* (subunit zeta)	P40227	Cytoplasm		
	*CCT8* (subunit theta)	P50990	Cytoskeleton - Cytoplasm		
Heat shock protein 75 kDa	*TRAP1*	Q12931	Mitocondrion	Molecular chaperone	Translational attenuation [46]
Elongation factor 1-alpha 1	*EEF1A1*	P68104	Cell membrane - nucleus	Elongation factor	Regulation of chaperone-mediated autophagy [47]
EF2; Elongation factor 2	*EEF2*	P13639	Cytoplasm - Nucleus	Elongation factor	Inhibition of protein synthesis [48]
TER ATPase; Transitional endoplasmic reticulum ATPase	*VCP*	P55072	Cytosol; ER; nucleus	Hydrolase	Elimination of misfolded proteins from the ER [51]; ERAD pathway

GBM protein factors whose ability to bind ATP/GTP appeared modified by the presence of CPZ and thus recognized as potential targets of the drug. Their identification has been done via ABPP-MS, using a kinase enrichment procedure with an insoluble ATP probe

In summary, using these techniques, we identified a consistent set of factors whose ability to bind ATP was modulated by CPZ, all involved in protein folding/misfolding, ER stress and the three arms of UPR.

CPZ induces ER stress and activates UPR response

The results described above encouraged us to investigate the effects of CPZ on ER stress and, consequently, on the UPR pathways.

Cells were treated with CPZ for 24 h, using the reference concentrations established in our previous work and corresponding to the IC30 value of the drug, as determined after 48 h of exposure for these cell lines (Table S7) [17] and reported in. For the respective controls (CTL), an equal volume of solvent (PBS) was added. Cells were thus analyzed by qRT-PCR for the relative expression of both the ER stress sensor *HSPA5* and the downstream UPR genes *ATF6*, u-XBP1, s-XBP1 and *ATF4* [29]. Both *XBP1* and its spliced isoform transcripts are target of IRE1 protein activity; mRNA expression of *ATF4*, target of PERK1, is a reliable marker of the ATF4 protein factor [52, 53], while *ATF6* evaluation needed further investigation at the protein level (see below).

**Fig. 1:**
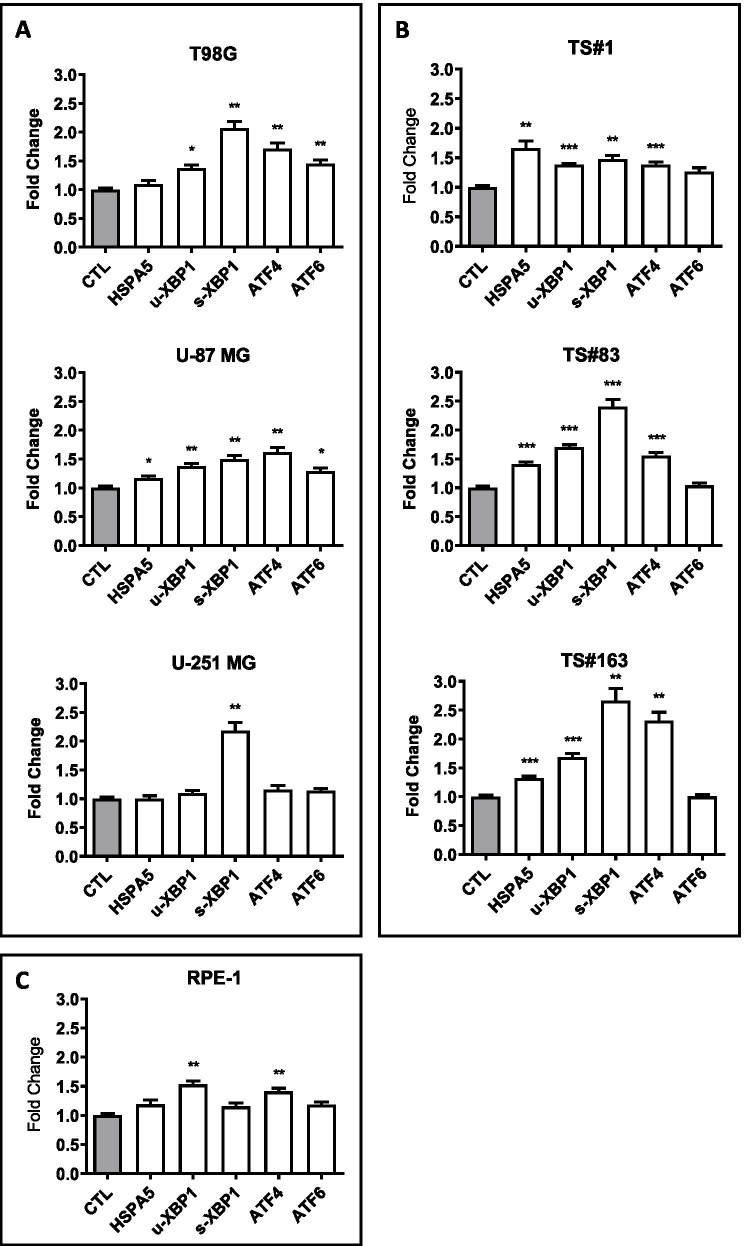
CPZ induces ER stress and activates UPR response. qRT-PCR determinations of the ER stress- and UPR-related genes *HSPA5*, *ATF6*, *XBP1* [either unspliced (u-XBP1) or spliced (s-XBP1)] and *ATF4* in T98G, U-87 MG and U-251 MG anchorage-dependent GBM cells (**A**); TS#1, TS#83 and TS#163 neurospheres (**B**) and RPE-1 non-cancer cells (**C**). Statistical significance is referred toward the respective control (CTL) (*p<0.05; **p<0.01; ***p<0.001)

All these determinants were assayed in three anchorage-dependent GBM cells (Fig. 1 A), in three neurospheres (Fig. 1B) and the RPE-1, anchorage-dependent human immortalized non-cancer cells (Fig. 1 C). Raw data are available as Additional File 2 (see Supplementary Material, AF2_RT-PCR). After treatment with CPZ, we observed a significant up-regulation of most of these genes, especially in neurospheres, thus suggesting the ability of this compound in inducing ER stress and UPR in GBM cells. RPE-1 non-cancer cells appeared less responsive toward CPZ-induced modifications in the expression of these markers. Interestingly, GBM cells showed a significant upregulation of the spliced XBP1 isoform (s-XBP1) compared with non-tumor RPE-1 cells that displayed instead a significant increase in the expression of the unspliced XBP1 transcript (u-XBP1). This last isoform codes for a protein, pXBP1(U), originally considered non-functional [54], to which a role essentially antithetical (e.g. dominant negative) to the most investigated pXBP1(S) isoform has been attributed [55–57].

These results indicate a role of CPZ in promoting ER stress and the consequent UPR in GBM cells while eliciting a peculiar pattern of gene stimulation in the non-cancer RPE-1 cells.

CPZ induces ATF6-α nuclear accumulation in GBM cells

**Fig. 2:**
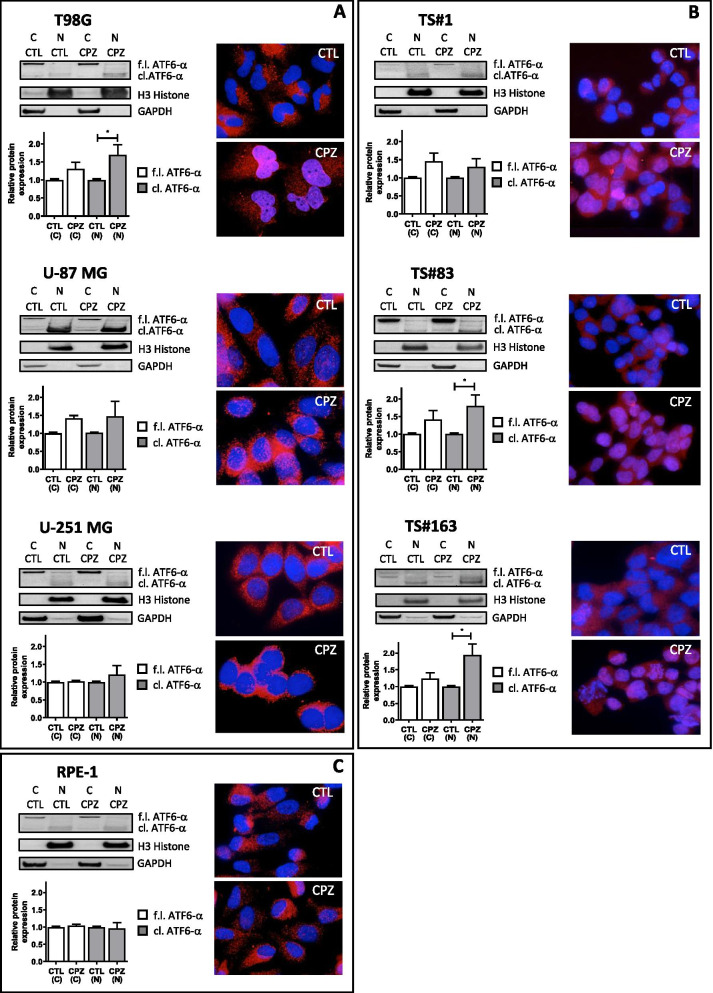
CPZ induces ATF6-α nuclear accumulation in GBM cells. ATF6-α full length (fl.) or cleaved (cl.) determination, in cytoplasmic or nuclear cell fractions, by means of western blot (images, upper left), their relative quantification (histograms, lower left) and immunofluorescence microscopy (pictures, right) in control (CTL) or CPZ-treated T98G, U-87 MG and U-251 MG anchorage-dependent GBM cells (**A**), TS#1, TS#83 and TS#163 (**B**) neurospheres and RPE-1 non-cancer cells (**C**). In the immunofluorescence images, ATF6-α, both fl. and cl., is represented in red, while cell nuclei are represented in blue. In western blots, H3 histone and GAPDH have been determined as nuclear and cytoplasmic markers, respectively, and used for relative quantification. Statistical significance in the histograms is referred solely to nuclear ATF6-α in CPZ-treated cells toward the respective control (CTL) (*p<0.05)

When BiP releases the third sensor ATF6-α, this one translocates to the Golgi apparatus, where it is cleaved by specific proteases. Cleaved ATF6-α undergoes further translocation to the nucleus, where it acts as a transcription factor, upregulating a set of UPR target genes that essentially overlap those activated by *XBP1* and *ATF4* [58]. Actually, in our system, exposure to CPZ prompted nuclear translocation of cleaved ATF6-α in both anchorage-dependent GBM cells and neurospheres, as indicated by western blotting determinations on separate cytoplasmic and nuclear protein fractions (blots and histograms) and representative immunofluorescence images as well (Fig. 2 A and B). Conversely, ATF6-α nuclear translocation was not apparent in RPE-1 non-cancer cells (Fig. 2 C). Raw data for western blotting are available as Additional File 3 (see Supplementary Material, AF3_WB ATF6 C-N).

These results outline the ability of CPZ to trigger ER stress and consequent UPR response in GBM cells, bringing to our attention the different behavior of the non-cancer RPE-1 cells in response to the drug.

### CPZ-mediated ER stress induces an autophagic response

**Fig. 3:**
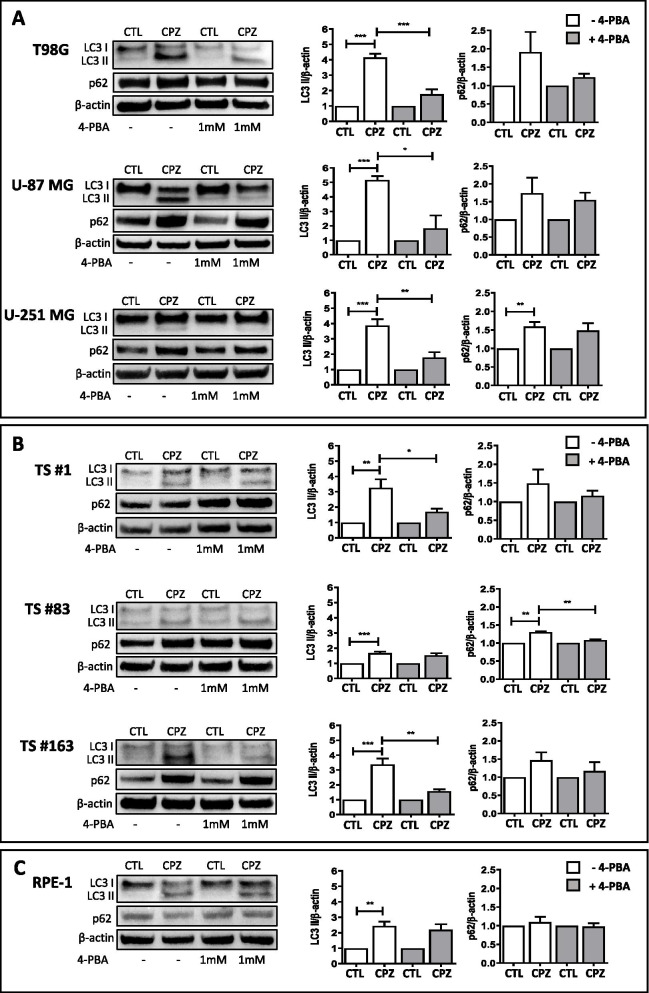
CPZ-mediated ER stress induces an autophagic response – western blot. Determination, by means of western blot (images, left) and their relative quantification (histograms, right) of the autophagy markers LC3 II and p62 in control (CTL) and CPZ-treated cells, either in the presence (grey columns) or absence (white columns) of the ER stress inhibitor 4-PBA, in control (CTL) or CPZ-treated T98G, U-87 MG and U-251 MG anchorage-dependent GBM cells (**A**), TS#1, TS#83 and TS#163 neurospheres (**B**) and RPE-1 non-cancer cells (**C**). β-actin determination has been used for relative quantification. When present, statistical significance is indicated (*p<0.05; **p<0.01; ***p<0.001)

**Fig. 4:**
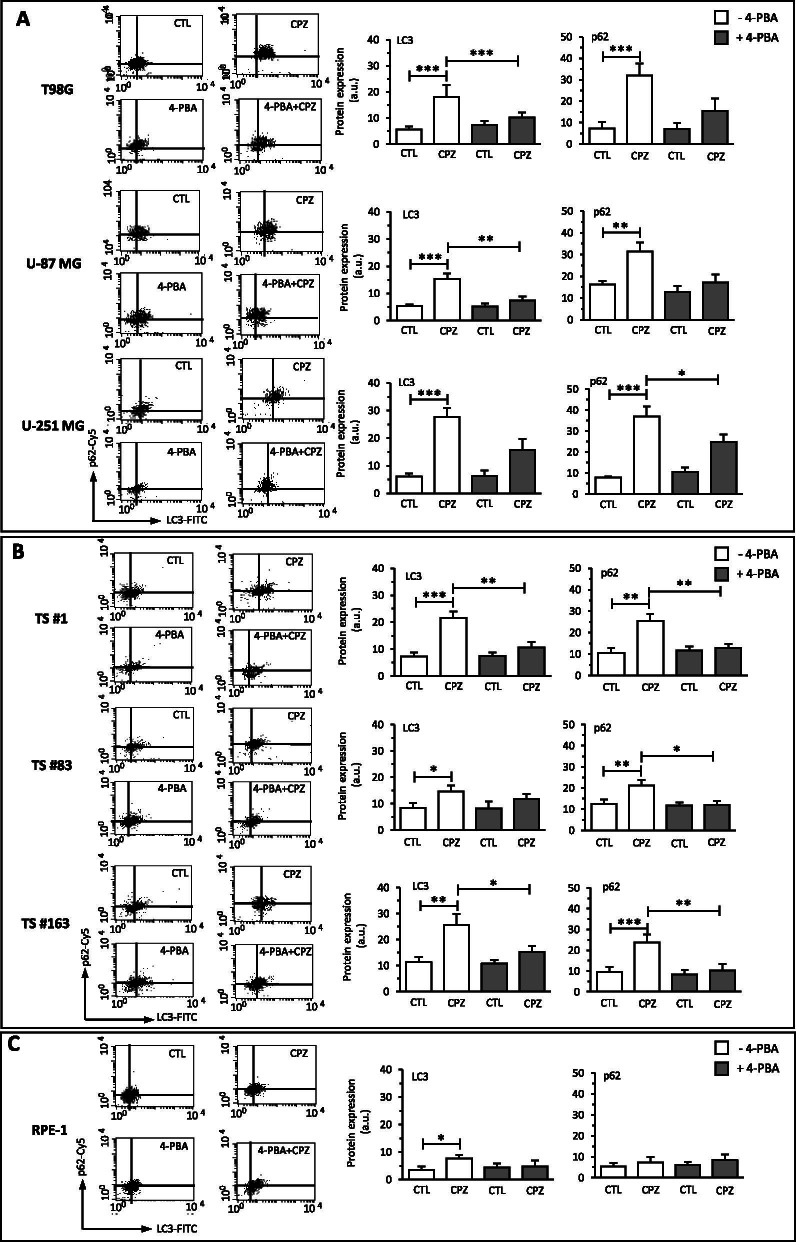
CPZ-mediated ER stress induces an autophagic response – cytofluorimetry. Determination, by means of cytofluorimetry (graphs, left) and relative quantification (histograms, right) of the autophagy markers LC3 II and p62 in control (CTL) and CPZ-treated cells, either in the presence (grey columns) or absence (white columns) of the ER stress inhibitor 4-PBA, in T98G, U-87 MG and U-251 MG anchorage-dependent GBM cells (**A**), TS#1, TS#83 and TS#163 neurospheres (**B**) and RPE-1 non-cancer cells (**C**). When present, statistical significance is indicated (*p<0.05; **p<0.01; ***p<0.001)

CPZ has been shown to trigger autophagy in the U-87 MG GBM cell line, bringing them to non-apoptotic cell death [11]. Autophagy can be induced in normal and cancer cells by multiple conditions *via* stimulation of specific cell sensors, where the AKT/mTOR axis represents one of the major signal transduction pathways involved [59, 60]. Growing evidence indicates an interplay between autophagic processes and UPR, highlighting an essential protective role of autophagy during ER stress [26, 60]. To determine whether CPZ induced autophagy in our GBM cells and if it was elicited through the ER stress-mediated pathway, we evaluated the protein levels of the autophagy markers LC3-II (the phosphatidylethanolamine-conjugated LC3-I protein) and p62/SQSTM1 (henceforth p62) in CTL- versus CPZ-treated GBM cells. Western blot determinations and cytofluorimetric analyses were performed in the absence or presence of the ER stress antagonist 4-phenylbutyrate (4-PBA) [61]. In parallel, RPE-1 non-cancer cells were also assayed. As shown in Fig. 3, western blot analysis of the autophagy markers LC3 II and p62 (images on the left) and their subsequent quantification (histograms on the right) revealed a significant increase of LC3 II after exposure to CPZ in all the cells assayed, namely anchorage-dependent GBM cells (Fig. 3 A), neurospheres (Fig. 3B) and RPE-1 non-cancer cells (Fig. 3 C). Raw data for western blotting are available as Additional File 4 (see Supplementary Material, AF4_WB Autophagy CPZ+PBA). When these cells were pre-treated for 5 h with the ER stress inhibitor 4-PBA (1 mM) and successively exposed to CPZ, a slighter increase in LC3 II was observed. The analysis of p62, after treatment of GBM cells with CPZ, showed a general trend toward a gain of this late autophagic marker, suggestive of an autophagosome accumulation and a consistent arrest in the autophagic flux. Pre-treatment of GBM cells with 4-PBA did not change significantly p62 expression. Interestingly, CPZ induced a milder increase of LC3 II in RPE-1 cells, not coupled with an increase in p62, behavior compatible with a successful cytoprotective effect connected to the initiation of the autophagic process by the drug. Here, pre-treatment of RPE-1 cells with 4-PBA did not produce substantial changes in LC3-II and p62 expression. When both autophagy markers LC3 II and p62 were analyzed by flow cytometry using a dedicated procedure, overlapping results were attained (Fig. 4). Raw data are available as Additional File 5 (see Supplementary Material, AF5_P62-LC3).

Altogether, these data suggest the ability of CPZ to induce a UPR-related autophagic response essentially cytotoxic, abortive, for the GBM cells, but cytoprotective for the RPE-1 cells, which also, in this case, appeared less susceptible to the toxic effects of the drug.

### CPZ induces ROS production

**Fig. 5:**
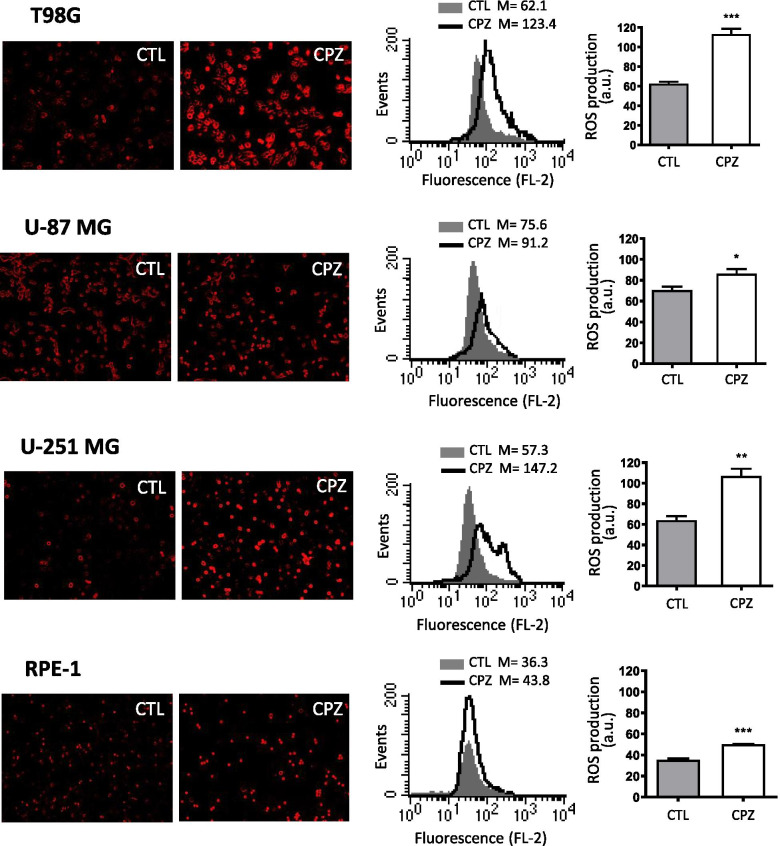
CPZ induces ROS production. Immunofluorescence (red in the pictures, left) and cytofluorimetric (graphs and histograms, right) expression levels of superoxide anion (O_2_^−^) in control (CTL) or CPZ-treated T98G, U-87 MG and U-251 MG anchorage-dependent GBM cells and RPE-1 non-cancer cells. Statistical significance is referred toward the control (CTL) (*p<0.05; **p<0.01; ***p<0.001)

Several studies highlight the role of antipsychotics in inducing reactive oxygen species (ROS) generation in both non-reproductive cells and solid tumors, supporting oxidative stress as a possible mechanism responsible for toxicity and cell death elicited by these drugs [62–64]. On these bases, we investigated the ability of CPZ to induce ROS generation, thus generating oxidative stress, partially caused also by ER stress. To this end, we evaluated the expression levels of the superoxide anion (O_2_^−^). By fluorescence microscopy, we observed an increased number of fluorescent cells (also brighter) in CPZ-treated anchorage-dependent GBM cells when compared with control cells (CTL) (Fig. 5, left), thus indicating enhanced levels of superoxide radicals.

Quantification of ROS production by flow cytometry (Fig. 5, right) showed, besides the constant CPZ-induced ROS increase, some cell line-related differences. Raw data are available as Additional File 6 (see Supplementary Material, AF6_ROS). ROS production appeared more marked in T98G and U-251 MG than in U-87 MG cells. Interestingly, the RPE-1 non-cancer cell line produced significantly less ROS at the baseline, with a limited induction by CPZ.

These concordant results obtained by fluorescence microscopy and cytofluorimetry supported the ability of CPZ in inducing ROS, especially in the cancer phenotype.

### CPZ induces mitotic catastrophe

**Fig. 6:**
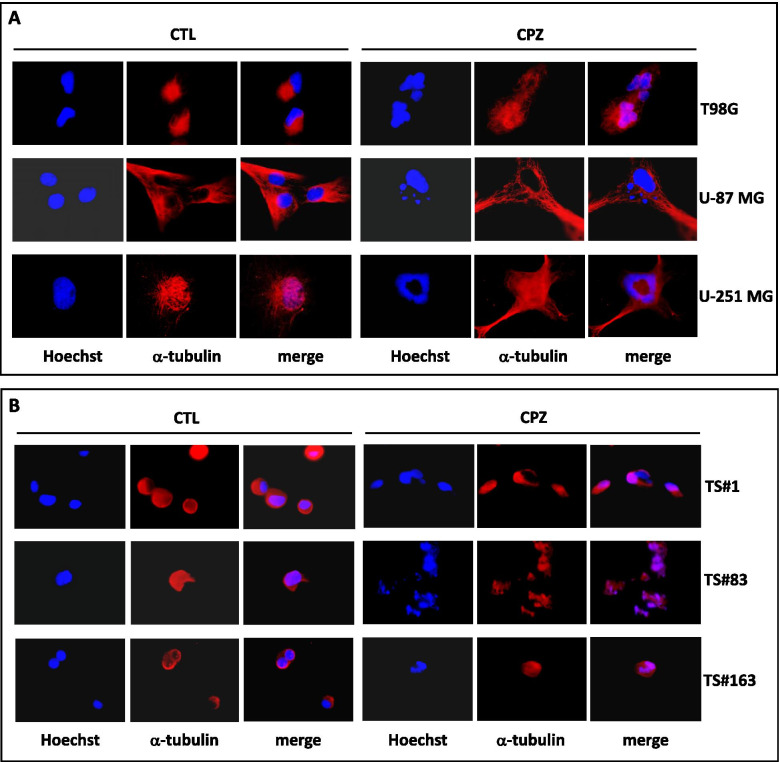
CPZ induces mitotic catastrophe. Nuclear aberrations in control (CTL) or CPZ-treated T98G, U-87 MG and U-251 MG anchorage-dependent GBM cells **(A)** and TS#1, TS#83 and TS#163 neurospheres **(B)**. Nuclei are stained in blue (Hoechst), while α-tubulin is highlighted in red and merge is the overlap of the two stainings. Aberrant nuclei are evident in all CPZ-treated GBM cells

In a previous report, we demonstrated that CPZ-treated GBM cell lines, in front of a clear decrease in cell viability, did not show, *via* cell cycle analysis, the characteristic sub-G1 peak, a hallmark of apoptosis; nevertheless, a noticeable number of hyperdiploid cells was detectable. Fluorescence microscopy confirmed the presence of abnormal nuclei, suggestive of the ability of CPZ to induce aberrant mitotic segregation. Under the same conditions, RPE-1 non-cancer cells showed neither hyperploidy nor aberrant mitosis hallmarks [17]. With the aim to validate those results, we performed an immunofluorescence analysis to detect nuclear aberrations in anchorage-dependent GBM cells, as well as in the neurospheres. Cells were treated for 48 h with IC30 CPZ or solvent (CTL) and then stained for nuclei (Hoechst 33,342, blue) and α-tubulin (fluorescent antibody, red). All CPZ-treated GBM cells exhibited aberrant nuclear features, i.e., micronuclei, aberrant monopolar spindle morphology and multinucleated giant cells (Fig. 6).

We can thus assume that, in this context, the autophagic process elicited by CPZ via UPR was not adequate to guarantee the survival of the hit GBM cells and that these underwent death via apoptosis-independent mechanisms, including mitotic catastrophe.

### CPZ-induced mitotic catastrophe in GBM cells is dependent on ER stress

**Fig. 7:**
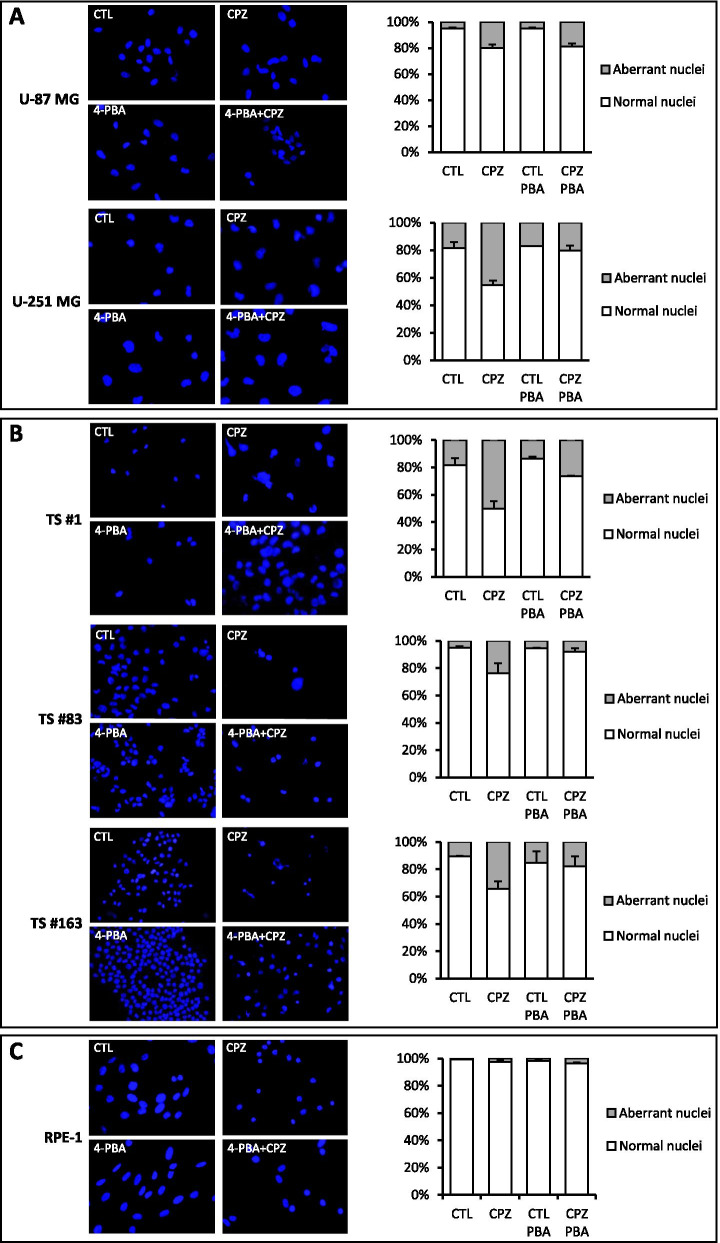
CPZ-induced mitotic catastrophe in GBM cells is dependent on ER stress. Cell nuclei were stained with Hoechst and analyzed in control (CTL) and CPZ-treated cells, either in the presence or absence of the ER stress inhibitor 4-PBA in T98G, U-87 MG and U-251 MG anchorage-dependent GBM cells (**A**), TS#1, TS#83 and TS#163 neurospheres (**B**) and RPE-1 non-cancer cells (**C**). In each panel, left pictures are representative images, while histograms on the right highlight the relative number of normal (white) and aberrant (grey) nuclei counted in all the experimental sets

Mitotic catastrophe acts as an oncosuppressive mechanism able to drive mitosis-incompetent cells toward an irreversible fate, with the aim to eliminate them [65]. To assess whether CPZ induced hyperploidy and mitotic catastrophe related to the ER stress-mediated pathway, we analyzed nuclear morphology in GBM cells after exposure to CPZ with or without pretreatment with the ER stress inhibitor 4-PBA. Confirming our previous results [17], GBM cells exposed for 48 h to CPZ displayed a consistent increase in nuclear aberrations, whose quantification has been reported in the histograms at the right of each set of panels. Noteworthy, in 4-PBA-pretreated GBM cells, a marked reduction in the number of aberrant nuclei, as well as reduced toxicity of CPZ, was apparent, except for the U-87 MG cell line. In this experimental setting, we excluded the evaluation of the anchorage-dependent T98G cell line, in which the outcome appeared biased by the presence of a high portion of hyperdiploid cells at the baseline, as reported [66]. Remarkably, the non-cancer RPE-1 cells displayed lower CPZ-induced aberrant mitoses and, consequently, less evident modifications attributable to the pre-treatment with 4-PBA (Fig. 7).

These results support an active role of the CPZ-induced ER stress in eliciting also mitotic catastrophe in GBM cells and outline the unresponsiveness of the RPE-1 cells to the nuclear modifications induced by the drug.

## Discussion

Besides its well-known clinical efficacy in the treatment of psychiatric disorders, several recent reports depict CPZ as a multifaceted drug that is gaining increasing relevance in oncology, being able to interfere, beyond DRD2 at the synaptic level, with several cancer-related cellular factors [16, 17]. In this work, we identified via ABPP + MS several cellular proteins involved in ER stress and UPR as possible targets of CPZ, due to the ability of this compound to modify their affinity toward ATP and thus, conceivably, their function. Starting from this evidence, we assessed a role for CPZ in increasing ER stress with consequent UPR, inducing autophagy, increasing oxidative stress (ROS production), provoking nuclear aberrations, mitotic catastrophe and ultimately driving GBM cells to death. Presently, we can detect the CPZ-related induction of ER stress and UPR, thus confirming the interference of the drug with specific cellular sensors, as suggested by the ABPP + MS results, but currently, we cannot completely identify the molecular pathways modulated by CPZ that can be considered responsible for these outcomes.

Even if, in some of our experimental results, anchorage-dependent GBM cell lines displayed different sensitivity to CPZ when compared with neurospheres, the striking differences in CPZ sensitivity displayed by the RPE-1 should be outlined. These are non-cancer, anchorage-dependent cells derived from the retinal epithelium and immortalized via hTERT overexpression [36]. These cells, when exposed to CPZ, displayed: (a) a different pattern in ER stress and UPR response, as evaluated via RT-PCR mRNA quantification of reporter genes, where it is worthy of note the significant activation of the *u-XBP1* gene occurring in this cell line; (b) a substantial lack in an increase of cleaved ATF6-α nuclear localization; (c) lower sensitivity towards autophagic cell death, as arguable by a moderate LC3 II increase and stable p62 expression; (d) lower ROS production, either at the baseline or under the effect of CPZ; and (e) absence of aberrant nuclei, either at the baseline or under the effect of CPZ. All these differences appear to converge in providing the RPE-1 cells with higher proficiency in contrasting the toxic effects of the drug. Two faces of the autophagic process should therefore be considered, pro-survival and pro-death, resulting in opposite effects [67]. This can support the hypothesis that the drug-induced autophagy could be overall cytotoxic, abortive, for GBM cells, while partially cytoprotective for the RPE-1 cells and explain why decades of use of CPZ in the clinic did not bring about severe cell toxicity-related side effects in treated patients.

The induction of abortive autophagy has been described also for other anticancer drugs, such as salinomycin that causes non-apoptotic death in GBM cells following ROS-dependent abortive autophagy [68]. Interestingly, the inhibition of ROS generation can restore the autophagic flow, thus suggesting that oxidative stress could play a role in blocking the autophagic process [69].

**Fig. 8:**
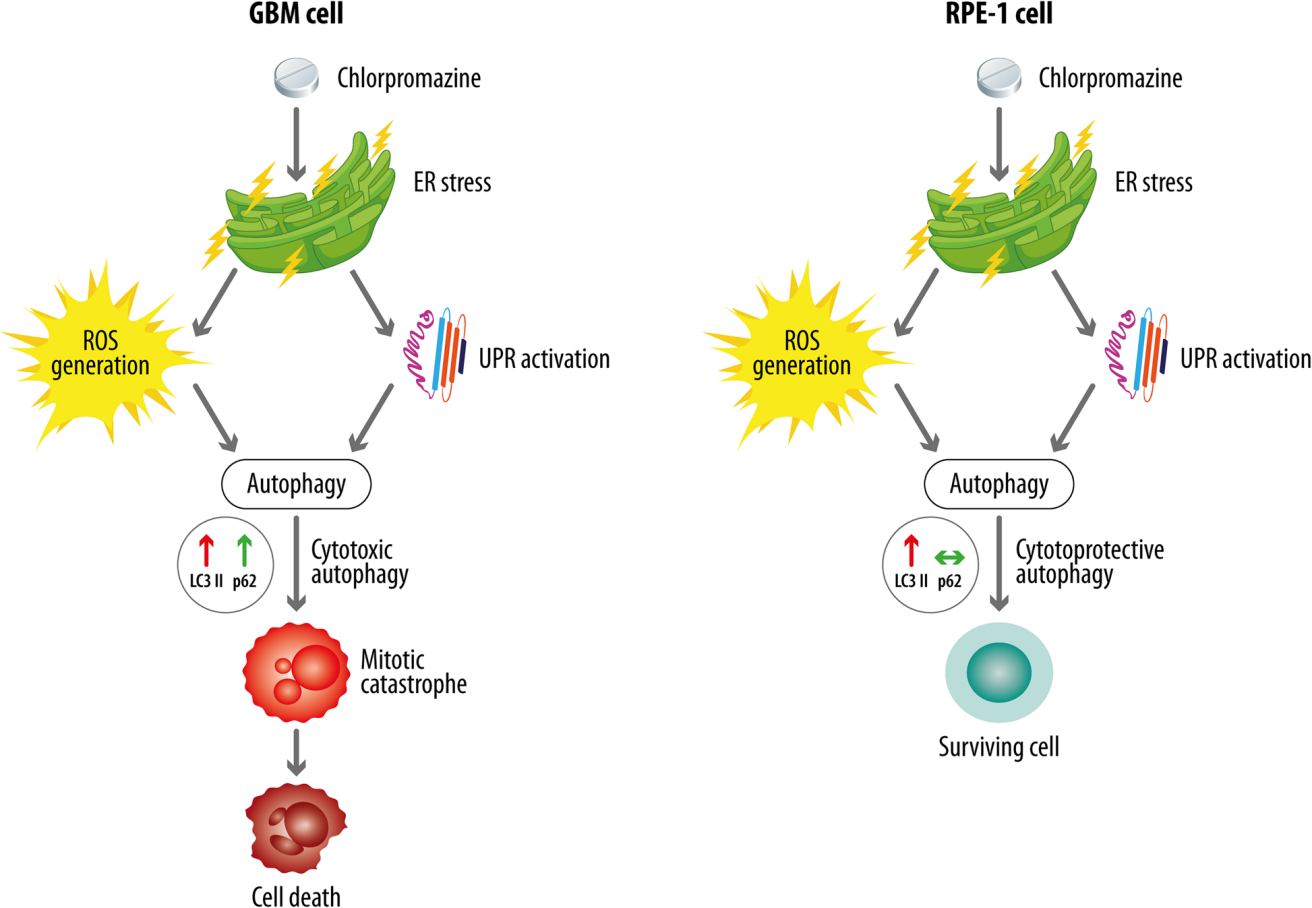
Mechanisms that can explain CPZ toxicity towards GBM cells while partially sparing RPE-1 non-cancer cells. CPZ exerts a role in affecting GBM cell growth and survival. CPZ ignites in GBM, via ER stress, UPR and ROS generation, a non-apoptotic cell death, mainly related to cytotoxic autophagy and induction of nuclear aberrations that culminate in a mitotic catastrophe (left). Our data highlight the higher toxicity of the drug towards GBM cells than RPE-1 non-cancer cells, possibly due to the ability of the latter to conduct a more proficient, survival-oriented, autophagic process (right)

In Fig. 8, we summarized the effects elicited by CPZ in GBM and RPE-1 non-cancer cells and hypothesize that GBM cells were those predominantly damaged by the drug *via* initiation of an abortive autophagy process that induced cytotoxic response and generation of aberrant nuclei with consequent mitotic catastrophe as well.

In our experimental setting, RPE-1 cells appeared less prone to undergo death *via* those CPZ-triggered mechanisms. We chose the RPE-1 non-cancer cells as a reliable counterpart to GBM because RPE-1 cells are from the neuro-ectodermal origin and thus embryologically related to neuro-ectodermal tumors. We are planning to assay CPZ toxicity *in vitro* toward other normal cell models, also derived from different tissues.

CPZ is an old and safe drug, known since the 1950 s as a compound able to interfere at the level of the synaptic dopamine reuptake. It has been, and still is, widely used to treat psychiatric disorders, as acute/chronic psychoses, and provides clinical support in severe vomiting and incoercible hiccups. The most critical side effects of CPZ reside in dose-dependent sedation and, at higher doses, in the occurrence of an extrapyramidal syndrome, both reversible upon drug withdrawal. Presently, second and third-generation neuroleptic drugs with a similar synaptic mechanism of action are available, but CPZ is still listed within the 2019 WHO Model List of Essential Medicines (current version) [70].

Our recent data further support the commitment to undertake a phase II clinical trial, approved by our Institutional Ethical Committee (Comitato Etico Centrale IRCCS - Sezione IFO-Fondazione Bietti, Rome, Italy) on 6 September 2019 (EudraCT # 2019-001988-75; ClinicalTrials.gov Identifier: NCT04224441). The schedule consists of the addition of CPZ to the standard GBM treatment in patients carrying hypo- or un-methylated *MGMT* gene, i.e., those characterized by resistance to TMZ. The dose of CPZ administered to GBM patients is 50 mg/day for 6 months, in concomitance with TMZ, in the adjuvant phase of the first-line treatment. We expect that this clinical trial would provide results concordant with the *in vitro* effects of CPZ in the cancer phenotype.

## Conclusions

Considering the plasticity of GBM and its ability to reformulate its cell population based on the selective pressure generated by treatment [71, 72], this tumor appears difficult to be challenged by targeted therapies and makes it reasonable to consider the opportunity to use “dirty drugs” capable of hitting some generalized vulnerabilities of cancer cells. Indeed, CPZ appears as a drug with pleiotropic effects and is able, according to our results, to show different toxicity patterns between GBM and the non-cancer RPE-1 cells.

In conclusion, we would also outline that the staggering costs of novel cancer drugs and the long time it takes for them to reach the market suggest trying drug repurposing as a profoundly different approach to keeping life-saving therapies affordable for cancer patients. This can be a suitable approach when no approved therapy is available or in the case of patients that have exhausted all available treatment options.

## Supplementary Information

**Additional file 1.**

**Additional file 2.**

**Additional file 3.**

**Additional file 4.**

**Additional file 5.**

**Additional file 6.**

**Additional file 7: Table S1.** CPZ IC30 values for each of the cell lines utilized. They represent the drug concentrations able to inhibit the cell growth by 30% after 48 hours of exposure.

## Data Availability

All the data and materials used and/or analyzed during this study are available from the corresponding authors on reasonable request.

## Abbreviations

CPZ: chlorpromazine
ER: endoplasmic reticulum
UPR: unfolded protein response
GBM: glioblastoma
TMZ: temozolomide
DRD2: dopamine receptor D2
ABPP: Activity-Based Protein Profiling
MS: mass spectrometry
STR: short tandem repeat
4-PBA: 4-phenylbutyrate
PBS: phosphate buffered saline
IC30: 30% inhibitory concentration
DHE: dihydroethidium
SE: Standard Error
CTL: control
ROS: reactive oxygen species
O_2_^-^: superoxide anion
