# Supplementary material for: Chlorpromazine induces cytotoxic autophagy in glioblastoma cells via endoplasmic reticulum stress and unfolded protein response
Source: J Exp Clin Cancer Res. 2021 Nov 5;40:347. doi: 10.1186/s13046-021-02144-w (PMC8569984; doi:10.1186/s13046-021-02144-w)
Supplement: Supplementary file 1 — Additional file 1. [file 13046_2021_2144_MOESM1_ESM.zip › MASCOT IDs/Mascot PMF_ ENPL_HUMAN.html]

Mascot Search Results: ENPL\_HUMAN
  

# MASCOT Search Results

## Protein View: ENPL\_HUMAN

### Endoplasmin OS=Homo sapiens OX=9606 GN=HSP90B1 PE=1 SV=1

|  |  |
| --- | --- |
| Database: | SwissProt |
| Score: | 131 |
| Expect: | 1.6e-09 |
| Monoisotopic mass (Mr): | 92696 |
| Calculated pI: | 4.76 |
| Taxonomy: | Homo sapiens |

Sequence similarity is available as an NCBI BLAST search of ENPL\_HUMAN against nr.

### Search parameters

|  |  |
| --- | --- |
| Enzyme: | Trypsin: cuts C-term side of KR unless next residue is P. |
| Fixed modifications: | Carbamidomethyl (C) |
|  |  |
| --- | --- |
| Mass values searched: | 286 |
| Mass values matched: | 53 |

### Protein sequence coverage: 55%

Matched peptides shown in ***bold red***.

|  |  |  |  |  |  |
| --- | --- | --- | --- | --- | --- |
| `1` | `MRALWVLGLC` | `CVLLTFGSVR` | `ADDEVDVDGT` | `VEEDLGKSRE` | `GSRTDDEVVQ` |
| `51` | `REEEAIQLDG` | `LNASQIRELR` | `EKSEKFAFQA` | `EVNRMMKLII` | `NSLYKNKEIF` |
| `101` | `LRELISNASD` | `ALDKIRLISL` | `TDENALSGNE` | `ELTVKIKCDK` | `EKNLLHVTDT` |
| `151` | `GVGMTREELV` | `KNLGTIAKSG` | `TSEFLNKMTE` | `AQEDGQSTSE` | `LIGQFGVGFY` |
| `201` | `SAFLVADKVI` | `VTSKHNNDTQ` | `HIWESDSNEF` | `SVIADPRGNT` | `LGRGTTITLV` |
| `251` | `LKEEASDYLE` | `LDTIKNLVKK` | `YSQFINFPIY` | `VWSSKTETVE` | `EPMEEEEAAK` |
| `301` | `EEKEESDDEA` | `AVEEEEEEKK` | `PKTKKVEKTV` | `WDWELMNDIK` | `PIWQRPSKEV` |
| `351` | `EEDEYKAFYK` | `SFSKESDDPM` | `AYIHFTAEGE` | `VTFKSILFVP` | `TSAPRGLFDE` |
| `401` | `YGSKKSDYIK` | `LYVRRVFITD` | `DFHDMMPKYL` | `NFVKGVVDSD` | `DLPLNVSRET` |
| `451` | `LQQHKLLKVI` | `RKKLVRKTLD` | `MIKKIADDKY` | `NDTFWKEFGT` | `NIKLGVIEDH` |
| `501` | `SNRTRLAKLL` | `RFQSSHHPTD` | `ITSLDQYVER` | `MKEKQDKIYF` | `MAGSSRKEAE` |
| `551` | `SSPFVERLLK` | `KGYEVIYLTE` | `PVDEYCIQAL` | `PEFDGKRFQN` | `VAKEGVKFDE` |
| `601` | `SEKTKESREA` | `VEKEFEPLLN` | `WMKDKALKDK` | `IEKAVVSQRL` | `TESPCALVAS` |
| `651` | `QYGWSGNMER` | `IMKAQAYQTG` | `KDISTNYYAS` | `QKKTFEINPR` | `HPLIRDMLRR` |
| `701` | `IKEDEDDKTV` | `LDLAVVLFET` | `ATLRSGYLLP` | `DTKAYGDRIE` | `RMLRLSLNID` |
| `751` | `PDAKVEEEPE` | `EEPEETAEDT` | `TEDTEQDEDE` | `EMDVGTDEEE` | `ETAKESTAEK` |
| `801` | `DEL` |  |  |  |  |

Unformatted sequence string: 803 residues (for pasting into other applications).

|  |  |  |  |
| --- | --- | --- | --- |
| Sort by | residue number | increasing mass | decreasing mass |
| Show | matched peptides only | predicted peptides also |  |

| Start | – | End | Observed | Mr(expt) | Mr(calc) | ppm | M | Peptide |
| --- | --- | --- | --- | --- | --- | --- | --- | --- |
| 40 | – | 67 | 3157.7053 | 3156.6980 | 3156.5283 | 53.8 | 2 | R.EGSRTDDEVVQREEEAIQLDGLNASQIR.E |
| 44 | – | 67 | 2728.4578 | 2727.4505 | 2727.3311 | 43.8 | 1 | R.TDDEVVQREEEAIQLDGLNASQIR.E |
| 71 | – | 84 | 1682.9279 | 1681.9206 | 1681.8423 | 46.5 | 2 | R.EKSEKFAFQAEVNR.M |
| 73 | – | 84 | 1425.7625 | 1424.7552 | 1424.7048 | 35.4 | 1 | K.SEKFAFQAEVNR.M |
| 76 | – | 84 | 1081.5781 | 1080.5708 | 1080.5352 | 33.0 | 0 | K.FAFQAEVNR.M |
| 76 | – | 87 | 1471.7866 | 1470.7793 | 1470.7112 | 46.4 | 1 | K.FAFQAEVNRMMK.L |
| 96 | – | 102 | 919.5547 | 918.5474 | 918.5287 | 20.4 | 1 | K.NKEIFLR.E |
| 103 | – | 116 | 1544.8711 | 1543.8638 | 1543.8205 | 28.0 | 1 | R.ELISNASDALDKIR.L |
| 117 | – | 135 | 2046.1292 | 2045.1219 | 2045.0528 | 33.8 | 0 | R.LISLTDENALSGNEELTVK.I |
| 136 | – | 142 | 920.5543 | 919.5470 | 919.4797 | 73.3 | 2 | K.IKCDKEK.N |
| 143 | – | 161 | 2112.1912 | 2111.1839 | 2111.1045 | 37.6 | 1 | K.NLLHVTDTGVGMTREELVK.N |
| 329 | – | 348 | 2542.4207 | 2541.4134 | 2541.2838 | 51.0 | 0 | K.TVWDWELMNDIKPIWQRPSK.E |
| 385 | – | 395 | 1187.7156 | 1186.7083 | 1186.6710 | 31.4 | 0 | K.SILFVPTSAPR.G |
| 396 | – | 404 | 1015.5055 | 1014.4982 | 1014.4658 | 31.9 | 0 | R.GLFDEYGSK.K |
| 396 | – | 405 | 1143.6313 | 1142.6241 | 1142.5608 | 55.4 | 1 | R.GLFDEYGSKK.S |
| 396 | – | 410 | 1749.9392 | 1748.9319 | 1748.8621 | 39.9 | 2 | R.GLFDEYGSKKSDYIK.L |
| 406 | – | 415 | 1312.7141 | 1311.7068 | 1311.7299 | -17.6 | 2 | K.SDYIKLYVRR.V |
| 415 | – | 428 | 1751.9102 | 1750.9029 | 1750.8171 | 49.0 | 1 | R.RVFITDDFHDMMPK.Y |
| 416 | – | 434 | 2360.1782 | 2359.1709 | 2359.1381 | 13.9 | 1 | R.VFITDDFHDMMPKYLNFVK.G |
| 429 | – | 448 | 2250.2910 | 2249.2837 | 2249.1692 | 50.9 | 1 | K.YLNFVKGVVDSDDLPLNVSR.E |
| 435 | – | 455 | 2350.3281 | 2349.3208 | 2349.1925 | 54.7 | 1 | K.GVVDSDDLPLNVSRETLQQHK.L |
| 467 | – | 474 | 976.5652 | 975.5579 | 975.5787 | -21.3 | 2 | R.KTLDMIKK.I |
| 468 | – | 479 | 1390.7562 | 1389.7490 | 1389.7537 | -3.43 | 2 | K.TLDMIKKIADDK.Y |
| 474 | – | 486 | 1643.9076 | 1642.9003 | 1642.7991 | 61.6 | 2 | K.KIADDKYNDTFWK.E |
| 475 | – | 493 | 2305.2139 | 2304.2066 | 2304.1062 | 43.6 | 2 | K.IADDKYNDTFWKEFGTNIK.L |
| 487 | – | 503 | 1929.0168 | 1928.0096 | 1927.9752 | 17.8 | 1 | K.EFGTNIKLGVIEDHSNR.T |
| 494 | – | 503 | 1139.6249 | 1138.6176 | 1138.5731 | 39.1 | 0 | K.LGVIEDHSNR.T |
| 494 | – | 505 | 1396.7749 | 1395.7676 | 1395.7219 | 32.8 | 1 | K.LGVIEDHSNRTR.L |
| 509 | – | 530 | 2642.4478 | 2641.4405 | 2641.3249 | 43.8 | 1 | K.LLRFQSSHHPTDITSLDQYVER.M |
| 512 | – | 530 | 2260.1501 | 2259.1429 | 2259.0556 | 38.6 | 0 | R.FQSSHHPTDITSLDQYVER.M |
| 535 | – | 546 | 1402.7283 | 1401.7210 | 1401.6711 | 35.6 | 1 | K.QDKIYFMAGSSR.K |
| 538 | – | 546 | 1031.5540 | 1030.5467 | 1030.4906 | 54.4 | 0 | K.IYFMAGSSR.K |
| 538 | – | 547 | 1159.6484 | 1158.6412 | 1158.5855 | 48.0 | 1 | K.IYFMAGSSRK.E |
| 547 | – | 557 | 1278.6826 | 1277.6753 | 1277.6252 | 39.3 | 1 | R.KEAESSPFVER.L |
| 561 | – | 587 | 3232.7495 | 3231.7422 | 3231.5798 | 50.3 | 2 | K.KGYEVIYLTEPVDEYCIQALPEFDGKR.F |
| 562 | – | 587 | 3104.6235 | 3103.6163 | 3103.4848 | 42.3 | 1 | K.GYEVIYLTEPVDEYCIQALPEFDGKR.F |
| 609 | – | 625 | 2106.1443 | 2105.1370 | 2105.0503 | 41.2 | 2 | R.EAVEKEFEPLLNWMKDK.A |
| 614 | – | 625 | 1549.8340 | 1548.8267 | 1548.7646 | 40.1 | 1 | K.EFEPLLNWMKDK.A |
| 624 | – | 630 | 817.4987 | 816.4914 | 816.4705 | 25.7 | 2 | K.DKALKDK.I |
| 640 | – | 660 | 2356.1667 | 2355.1595 | 2355.0623 | 41.2 | 0 | R.LTESPCALVASQYGWSGNMER.I |
| 664 | – | 683 | 2265.1965 | 2264.1893 | 2264.1073 | 36.2 | 2 | K.AQAYQTGKDISTNYYASQKK.T |
| 683 | – | 690 | 1004.5806 | 1003.5734 | 1003.5451 | 28.2 | 1 | K.KTFEINPR.H |
| 683 | – | 695 | 1620.9427 | 1619.9355 | 1619.9260 | 5.87 | 2 | K.KTFEINPRHPLIR.D |
| 684 | – | 690 | 876.4843 | 875.4770 | 875.4501 | 30.8 | 0 | K.TFEINPR.H |
| 684 | – | 695 | 1492.8783 | 1491.8710 | 1491.8310 | 26.8 | 1 | K.TFEINPRHPLIR.D |
| 684 | – | 699 | 2008.1199 | 2007.1126 | 2007.0836 | 14.4 | 2 | K.TFEINPRHPLIRDMLR.R |
| 691 | – | 699 | 1150.6296 | 1149.6224 | 1149.6441 | -18.9 | 1 | R.HPLIRDMLR.R |
| 701 | – | 724 | 2733.5830 | 2732.5757 | 2732.4484 | 46.6 | 2 | R.IKEDEDDKTVLDLAVVLFETATLR.S |
| 725 | – | 733 | 993.5744 | 992.5671 | 992.5179 | 49.6 | 0 | R.SGYLLPDTK.A |
| 725 | – | 741 | 1954.0822 | 1953.0749 | 1952.9956 | 40.6 | 2 | R.SGYLLPDTKAYGDRIER.M |
| 734 | – | 741 | 979.5269 | 978.5196 | 978.4883 | 32.1 | 1 | K.AYGDRIER.M |
| 739 | – | 754 | 1884.0557 | 1883.0484 | 1883.0298 | 9.86 | 2 | R.IERMLRLSLNIDPDAK.V |
| 742 | – | 754 | 1485.8022 | 1484.7950 | 1484.8021 | -4.78 | 1 | R.MLRLSLNIDPDAK.V |

`No match to: 801.5214, 803.4986, 819.4869, 832.4329, 833.4824, 834.4663, 842.5275, 845.5510, 846.5398, 847.5027, 849.4869, 850.4879, 854.4830, 861.5255, 863.5021, 869.5416, 878.4976, 879.4996, 880.5009, 881.5181, 886.5644, 889.5754, 890.5602, 900.5728, 905.5395, 921.5380, 933.5997, 935.5681, 937.4854, 938.4927, 941.5581, 949.5640, 953.5447, 957.5504, 968.5578, 969.5801, 970.5251, 972.5645, 974.5688, 977.6169, 981.5270, 997.5518, 1001.5588, 1016.5113, 1017.5331, 1020.5691, 1021.6122, 1030.5728, 1034.6045, 1037.5997, 1043.6273, 1047.5745, 1048.5905, 1052.6135, 1068.5994, 1072.5629, 1078.6407, 1089.5952, 1091.6110, 1092.5862, 1097.6770, 1099.6678, 1100.6466, 1120.5951, 1125.6517, 1128.6246, 1138.6222, 1142.6492, 1145.6757, 1152.7103, 1153.7010, 1160.6464, 1161.6678, 1166.6838, 1168.6571, 1169.7382, 1170.7356, 1174.6549, 1175.6545, 1190.7047, 1198.7399, 1199.6920, 1230.7004, 1232.6721, 1245.7134, 1248.7119, 1251.7090, 1267.7389, 1268.7300, 1296.7317, 1297.7506, 1319.7216, 1320.6963, 1321.6996, 1329.7321, 1330.7289, 1335.7111, 1341.7223, 1376.8040, 1377.8051, 1392.7712, 1398.7814, 1403.7478, 1408.7478, 1427.7693, 1436.7861, 1440.8051, 1442.8201, 1444.8015, 1448.8193, 1450.7780, 1452.8068, 1454.8079, 1486.8040, 1489.8108, 1494.8552, 1497.8295, 1503.8131, 1508.8571, 1536.7603, 1545.8798, 1553.9689, 1567.8763, 1578.8605, 1584.9640, 1603.8130, 1617.8979, 1636.9501, 1638.9347, 1639.9371, 1651.8126, 1654.8286, 1667.8082, 1680.9857, 1697.8842, 1706.9241, 1707.9109, 1709.9982, 1711.0015, 1712.9962, 1714.9858, 1731.9353, 1735.9971, 1744.9321, 1747.0229, 1753.9531, 1758.9720, 1767.9257, 1768.9402, 1770.0189, 1790.9559, 1791.9663, 1808.9796, 1833.0247, 1834.0211, 1837.0332, 1840.0339, 1842.0504, 1844.0492, 1861.0424, 1876.0688, 1878.0151, 1886.0527, 1899.0549, 1919.0525, 1937.1210, 1938.1501, 1946.0559, 1977.9646, 1994.0944, 2011.1132, 2012.1051, 2041.1411, 2065.1570, 2082.1611, 2083.2324, 2088.1465, 2107.1401, 2110.1746, 2113.1897, 2123.1653, 2124.1638, 2127.1726, 2134.1213, 2138.1277, 2145.2363, 2152.1907, 2169.2334, 2172.2375, 2174.2488, 2194.1396, 2223.2720, 2251.2805, 2264.1763, 2266.1943, 2269.2095, 2306.2102, 2309.1863, 2372.1621, 2375.1682, 2386.0750, 2415.3367, 2419.3379, 2434.3137, 2456.3384, 2459.3391, 2502.3613, 2505.3640, 2566.3430, 2574.4573, 2586.3337, 2601.4500, 2602.5305, 2608.4258, 2660.5352, 2724.4778, 2737.4878, 2813.5364, 2814.5127, 2847.5642, 2850.5432, 2979.6558, 3056.7615, 3079.6897, 3121.7170, 3139.7432, 3162.6687, 3176.7595, 3557.9661, 3579.1128, 3589.0520, 3885.1086, 3907.0935`

---

```
ID   ENPL_HUMAN              Reviewed;         803 AA.
AC   P14625; Q96A97;
DT   01-APR-1990, integrated into UniProtKB/Swiss-Prot.
DT   01-APR-1990, sequence version 1.
DT   02-JUN-2021, entry version 241.
DE   RecName: Full=Endoplasmin;
DE   AltName: Full=94 kDa glucose-regulated protein;
DE            Short=GRP-94;
DE   AltName: Full=Heat shock protein 90 kDa beta member 1;
DE   AltName: Full=Tumor rejection antigen 1;
DE   AltName: Full=gp96 homolog;
DE   Flags: Precursor;
GN   Name=HSP90B1 {ECO:0000312|HGNC:HGNC:12028}; Synonyms=GRP94, TRA1;
OS   Homo sapiens (Human).
OC   Eukaryota; Metazoa; Chordata; Craniata; Vertebrata; Euteleostomi; Mammalia;
OC   Eutheria; Euarchontoglires; Primates; Haplorrhini; Catarrhini; Hominidae;
OC   Homo.
OX   NCBI_TaxID=9606;
RN   [1]
RP   NUCLEOTIDE SEQUENCE [MRNA], AND NUCLEOTIDE SEQUENCE [GENOMIC DNA] OF 1-16.
RC   TISSUE=Blood;
RX   PubMed=2377606; DOI=10.1073/pnas.87.15.5658;
RA   Maki R.G., Old L.J., Srivastava P.K.;
RT   "Human homologue of murine tumor rejection antigen gp96: 5'-regulatory and
RT   coding regions and relationship to stress-induced proteins.";
RL   Proc. Natl. Acad. Sci. U.S.A. 87:5658-5662(1990).
RN   [2]
RP   NUCLEOTIDE SEQUENCE [LARGE SCALE MRNA].
RC   TISSUE=Skin;
RX   PubMed=15489334; DOI=10.1101/gr.2596504;
RG   The MGC Project Team;
RT   "The status, quality, and expansion of the NIH full-length cDNA project:
RT   the Mammalian Gene Collection (MGC).";
RL   Genome Res. 14:2121-2127(2004).
RN   [3]
RP   NUCLEOTIDE SEQUENCE [GENOMIC DNA] OF 1-16.
RC   TISSUE=Liver;
RX   PubMed=2546060; DOI=10.1128/mcb.9.5.2153;
RA   Chang S.C., Erwin A.E., Lee A.S.;
RT   "Glucose-regulated protein (GRP94 and GRP78) genes share common regulatory
RT   domains and are coordinately regulated by common trans-acting factors.";
RL   Mol. Cell. Biol. 9:2153-2162(1989).
RN   [4]
RP   NUCLEOTIDE SEQUENCE [MRNA] OF 22-803.
RC   TISSUE=Liver;
RA   Meng S., Tien P.;
RT   "The association of heat shock protein gp96 with HBV-derived peptides in
RT   vitro.";
RL   Submitted (JUN-2001) to the EMBL/GenBank/DDBJ databases.
RN   [5]
RP   PROTEIN SEQUENCE OF 22-39.
RC   TISSUE=Platelet;
RX   PubMed=12665801; DOI=10.1038/nbt810;
RA   Gevaert K., Goethals M., Martens L., Van Damme J., Staes A., Thomas G.R.,
RA   Vandekerckhove J.;
RT   "Exploring proteomes and analyzing protein processing by mass spectrometric
RT   identification of sorted N-terminal peptides.";
RL   Nat. Biotechnol. 21:566-569(2003).
RN   [6]
RP   COMPONENT OF A CHAPERONE COMPLEX, AND SUBCELLULAR LOCATION.
RX   PubMed=12475965; DOI=10.1091/mbc.e02-05-0311;
RA   Meunier L., Usherwood Y.-K., Chung K.T., Hendershot L.M.;
RT   "A subset of chaperones and folding enzymes form multiprotein complexes in
RT   endoplasmic reticulum to bind nascent proteins.";
RL   Mol. Biol. Cell 13:4456-4469(2002).
RN   [7]
RP   SUBCELLULAR LOCATION [LARGE SCALE ANALYSIS].
RC   TISSUE=Melanoma;
RX   PubMed=12643545; DOI=10.1021/pr025562r;
RA   Basrur V., Yang F., Kushimoto T., Higashimoto Y., Yasumoto K., Valencia J.,
RA   Muller J., Vieira W.D., Watabe H., Shabanowitz J., Hearing V.J., Hunt D.F.,
RA   Appella E.;
RT   "Proteomic analysis of early melanosomes: identification of novel
RT   melanosomal proteins.";
RL   J. Proteome Res. 2:69-79(2003).
RN   [8]
RP   GLYCOSYLATION AT ASN-217 AND ASN-445.
RX   PubMed=12754519; DOI=10.1038/nbt827;
RA   Zhang H., Li X.-J., Martin D.B., Aebersold R.;
RT   "Identification and quantification of N-linked glycoproteins using
RT   hydrazide chemistry, stable isotope labeling and mass spectrometry.";
RL   Nat. Biotechnol. 21:660-666(2003).
RN   [9]
RP   IDENTIFICATION BY MASS SPECTROMETRY [LARGE SCALE ANALYSIS].
RC   TISSUE=Cervix carcinoma;
RX   PubMed=18669648; DOI=10.1073/pnas.0805139105;
RA   Dephoure N., Zhou C., Villen J., Beausoleil S.A., Bakalarski C.E.,
RA   Elledge S.J., Gygi S.P.;
RT   "A quantitative atlas of mitotic phosphorylation.";
RL   Proc. Natl. Acad. Sci. U.S.A. 105:10762-10767(2008).
RN   [10]
RP   INTERACTION WITH CNPY3; TLR4 AND TLR9.
RX   PubMed=20865800; DOI=10.1038/ncomms1070;
RA   Liu B., Yang Y., Qiu Z., Staron M., Hong F., Li Y., Wu S., Li Y., Hao B.,
RA   Bona R., Han D., Li Z.;
RT   "Folding of Toll-like receptors by the HSP90 paralogue gp96 requires a
RT   substrate-specific cochaperone.";
RL   Nat. Commun. 1:79-79(2010).
RN   [11]
RP   ERRATUM OF PUBMED:20865800.
RA   Liu B., Yang Y., Qiu Z., Staron M., Hong F., Li Y., Wu S., Li Y., Hao B.,
RA   Bona R., Han D., Li Z.;
RL   Nat. Commun. 3:653-653(2012).
RN   [12]
RP   SUBCELLULAR LOCATION [LARGE SCALE ANALYSIS].
RC   TISSUE=Melanoma;
RX   PubMed=17081065; DOI=10.1021/pr060363j;
RA   Chi A., Valencia J.C., Hu Z.-Z., Watabe H., Yamaguchi H., Mangini N.J.,
RA   Huang H., Canfield V.A., Cheng K.C., Yang F., Abe R., Yamagishi S.,
RA   Shabanowitz J., Hearing V.J., Wu C., Appella E., Hunt D.F.;
RT   "Proteomic and bioinformatic characterization of the biogenesis and
RT   function of melanosomes.";
RL   J. Proteome Res. 5:3135-3144(2006).
RN   [13]
RP   GLYCOSYLATION [LARGE SCALE ANALYSIS] AT ASN-107.
RC   TISSUE=Platelet;
RX   PubMed=16263699; DOI=10.1074/mcp.m500324-mcp200;
RA   Lewandrowski U., Moebius J., Walter U., Sickmann A.;
RT   "Elucidation of N-glycosylation sites on human platelet proteins: a
RT   glycoproteomic approach.";
RL   Mol. Cell. Proteomics 5:226-233(2006).
RN   [14]
RP   FUNCTION, AND INTERACTION WITH OS9.
RX   PubMed=18264092; DOI=10.1038/ncb1689;
RA   Christianson J.C., Shaler T.A., Tyler R.E., Kopito R.R.;
RT   "OS-9 and GRP94 deliver mutant alpha1-antitrypsin to the Hrd1-SEL1L
RT   ubiquitin ligase complex for ERAD.";
RL   Nat. Cell Biol. 10:272-282(2008).
RN   [15]
RP   GLYCOSYLATION [LARGE SCALE ANALYSIS] AT ASN-62; ASN-107; ASN-217 AND
RP   ASN-445.
RC   TISSUE=Liver;
RX   PubMed=19159218; DOI=10.1021/pr8008012;
RA   Chen R., Jiang X., Sun D., Han G., Wang F., Ye M., Wang L., Zou H.;
RT   "Glycoproteomics analysis of human liver tissue by combination of multiple
RT   enzyme digestion and hydrazide chemistry.";
RL   J. Proteome Res. 8:651-661(2009).
RN   [16]
RP   PHOSPHORYLATION [LARGE SCALE ANALYSIS] AT SER-306, AND IDENTIFICATION BY
RP   MASS SPECTROMETRY [LARGE SCALE ANALYSIS].
RC   TISSUE=Cervix carcinoma;
RX   PubMed=20068231; DOI=10.1126/scisignal.2000475;
RA   Olsen J.V., Vermeulen M., Santamaria A., Kumar C., Miller M.L.,
RA   Jensen L.J., Gnad F., Cox J., Jensen T.S., Nigg E.A., Brunak S., Mann M.;
RT   "Quantitative phosphoproteomics reveals widespread full phosphorylation
RT   site occupancy during mitosis.";
RL   Sci. Signal. 3:RA3-RA3(2010).
RN   [17]
RP   IDENTIFICATION BY MASS SPECTROMETRY [LARGE SCALE ANALYSIS].
RX   PubMed=21269460; DOI=10.1186/1752-0509-5-17;
RA   Burkard T.R., Planyavsky M., Kaupe I., Breitwieser F.P., Buerckstuemmer T.,
RA   Bennett K.L., Superti-Furga G., Colinge J.;
RT   "Initial characterization of the human central proteome.";
RL   BMC Syst. Biol. 5:17-17(2011).
RN   [18]
RP   PHOSPHORYLATION [LARGE SCALE ANALYSIS] AT SER-306, AND IDENTIFICATION BY
RP   MASS SPECTROMETRY [LARGE SCALE ANALYSIS].
RX   PubMed=21406692; DOI=10.1126/scisignal.2001570;
RA   Rigbolt K.T., Prokhorova T.A., Akimov V., Henningsen J., Johansen P.T.,
RA   Kratchmarova I., Kassem M., Mann M., Olsen J.V., Blagoev B.;
RT   "System-wide temporal characterization of the proteome and phosphoproteome
RT   of human embryonic stem cell differentiation.";
RL   Sci. Signal. 4:RS3-RS3(2011).
RN   [19]
RP   PHOSPHORYLATION [LARGE SCALE ANALYSIS] AT SER-306, AND IDENTIFICATION BY
RP   MASS SPECTROMETRY [LARGE SCALE ANALYSIS].
RC   TISSUE=Cervix carcinoma, and Erythroleukemia;
RX   PubMed=23186163; DOI=10.1021/pr300630k;
RA   Zhou H., Di Palma S., Preisinger C., Peng M., Polat A.N., Heck A.J.,
RA   Mohammed S.;
RT   "Toward a comprehensive characterization of a human cancer cell
RT   phosphoproteome.";
RL   J. Proteome Res. 12:260-271(2013).
RN   [20]
RP   INTERACTION WITH METTL23.
RX   PubMed=23349634; DOI=10.1371/journal.pgen.1003210;
RA   Cloutier P., Lavallee-Adam M., Faubert D., Blanchette M., Coulombe B.;
RT   "A newly uncovered group of distantly related lysine methyltransferases
RT   preferentially interact with molecular chaperones to regulate their
RT   activity.";
RL   PLoS Genet. 9:E1003210-E1003210(2013).
RN   [21]
RP   PHOSPHORYLATION [LARGE SCALE ANALYSIS] AT SER-64; SER-447 AND THR-786, AND
RP   IDENTIFICATION BY MASS SPECTROMETRY [LARGE SCALE ANALYSIS].
RC   TISSUE=Liver;
RX   PubMed=24275569; DOI=10.1016/j.jprot.2013.11.014;
RA   Bian Y., Song C., Cheng K., Dong M., Wang F., Huang J., Sun D., Wang L.,
RA   Ye M., Zou H.;
RT   "An enzyme assisted RP-RPLC approach for in-depth analysis of human liver
RT   phosphoproteome.";
RL   J. Proteomics 96:253-262(2014).
RN   [22]
RP   PHOSPHORYLATION AT SER-306.
RX   PubMed=26091039; DOI=10.1016/j.cell.2015.05.028;
RA   Tagliabracci V.S., Wiley S.E., Guo X., Kinch L.N., Durrant E., Wen J.,
RA   Xiao J., Cui J., Nguyen K.B., Engel J.L., Coon J.J., Grishin N.,
RA   Pinna L.A., Pagliarini D.J., Dixon J.E.;
RT   "A single kinase generates the majority of the secreted phosphoproteome.";
RL   Cell 161:1619-1632(2015).
RN   [23]
RP   CLEAVAGE OF SIGNAL PEPTIDE [LARGE SCALE ANALYSIS] AFTER ALA-21, AND
RP   IDENTIFICATION BY MASS SPECTROMETRY [LARGE SCALE ANALYSIS].
RX   PubMed=25944712; DOI=10.1002/pmic.201400617;
RA   Vaca Jacome A.S., Rabilloud T., Schaeffer-Reiss C., Rompais M., Ayoub D.,
RA   Lane L., Bairoch A., Van Dorsselaer A., Carapito C.;
RT   "N-terminome analysis of the human mitochondrial proteome.";
RL   Proteomics 15:2519-2524(2015).
RN   [24]
RP   FUNCTION, AND INTERACTION WITH IL1B.
RX   PubMed=32272059; DOI=10.1016/j.cell.2020.03.031;
RA   Zhang M., Liu L., Lin X., Wang Y., Li Y., Guo Q., Li S., Sun Y., Tao X.,
RA   Zhang D., Lv X., Zheng L., Ge L.;
RT   "A Translocation Pathway for Vesicle-Mediated Unconventional Protein
RT   Secretion.";
RL   Cell 181:637-652(2020).
CC   -!- FUNCTION: Molecular chaperone that functions in the processing and
CC       transport of secreted proteins (By similarity). When associated with
CC       CNPY3, required for proper folding of Toll-like receptors (By
CC       similarity). Functions in endoplasmic reticulum associated degradation
CC       (ERAD) (PubMed:18264092). Has ATPase activity (By similarity). May
CC       participate in the unfolding of cytosolic leaderless cargos (lacking
CC       the secretion signal sequence) such as the interleukin 1/IL-1 to
CC       facilitate their translocation into the ERGIC (endoplasmic reticulum-
CC       Golgi intermediate compartment) and secretion; the translocation
CC       process is mediated by the cargo receptor TMED10 (PubMed:32272059).
CC       {ECO:0000250|UniProtKB:P08113, ECO:0000269|PubMed:18264092,
CC       ECO:0000269|PubMed:32272059}.
CC   -!- SUBUNIT: Homodimer; disulfide-linked. Component of an EIF2 complex at
CC       least composed of CELF1/CUGBP1, CALR, CALR3, EIF2S1, EIF2S2, HSP90B1
CC       and HSPA5 (By similarity). Part of a large chaperone multiprotein
CC       complex comprising DNAJB11, HSP90B1, HSPA5, HYOU, PDIA2, PDIA4, PDIA6,
CC       PPIB, SDF2L1, UGGT1 and very small amounts of ERP29, but not, or at
CC       very low levels, CALR nor CANX. Interacts with AIMP1; regulates its
CC       retention in the endoplasmic reticulum. Interacts with OS9. Interacts
CC       with CNPY3. This interaction is disrupted in the presence of ATP (By
CC       similarity). Interacts with TLR4 and TLR9, but not with TLR3. Interacts
CC       with MZB1 in a calcium-dependent manner (By similarity). Interacts with
CC       METTL23. Interacts with IL1B; the interaction facilitates cargo
CC       translocation into the ERGIC (PubMed:32272059). {ECO:0000250,
CC       ECO:0000269|PubMed:18264092, ECO:0000269|PubMed:20865800,
CC       ECO:0000269|PubMed:23349634, ECO:0000269|PubMed:32272059}.
CC   -!- INTERACTION:
CC       P14625; P05067: APP; NbExp=3; IntAct=EBI-359129, EBI-77613;
CC       P14625; P04626: ERBB2; NbExp=2; IntAct=EBI-359129, EBI-641062;
CC       P14625; Q00597: FANCC; NbExp=4; IntAct=EBI-359129, EBI-81625;
CC       P14625; P11021: HSPA5; NbExp=2; IntAct=EBI-359129, EBI-354921;
CC   -!- SUBCELLULAR LOCATION: Endoplasmic reticulum lumen
CC       {ECO:0000305|PubMed:12475965}. Sarcoplasmic reticulum lumen
CC       {ECO:0000250|UniProtKB:P41148}. Melanosome
CC       {ECO:0000269|PubMed:12643545, ECO:0000269|PubMed:17081065}.
CC       Note=Identified by mass spectrometry in melanosome fractions from stage
CC       I to stage IV. {ECO:0000269|PubMed:12643545,
CC       ECO:0000269|PubMed:17081065}.
CC   -!- SIMILARITY: Belongs to the heat shock protein 90 family. {ECO:0000305}.
CC   ---------------------------------------------------------------------------
CC   Copyrighted by the UniProt Consortium, see https://www.uniprot.org/terms
CC   Distributed under the Creative Commons Attribution (CC BY 4.0) License
CC   ---------------------------------------------------------------------------
DR   EMBL; X15187; CAA33261.1; -; mRNA.
DR   EMBL; M33716; AAA68201.1; -; Genomic_DNA.
DR   EMBL; BC066656; AAH66656.1; -; mRNA.
DR   EMBL; M26596; AAA58621.1; -; Genomic_DNA.
DR   EMBL; AY040226; AAK74072.1; -; mRNA.
DR   CCDS; CCDS9094.1; -.
DR   PIR; A35954; A35954.
DR   RefSeq; NP_003290.1; NM_003299.2.
DR   PDB; 4NH9; X-ray; 2.77 A; A=69-337.
DR   PDBsum; 4NH9; -.
DR   SMR; P14625; -.
DR   BioGRID; 113036; 363.
DR   CORUM; P14625; -.
DR   DIP; DIP-36060N; -.
DR   IntAct; P14625; 173.
DR   MINT; P14625; -.
DR   STRING; 9606.ENSP00000299767; -.
DR   BindingDB; P14625; -.
DR   ChEMBL; CHEMBL1075323; -.
DR   DrugBank; DB08465; 2-(3-AMINO-2,5,6-TRIMETHOXYPHENYL)ETHYL 5-CHLORO-2,4-DIHYDROXYBENZOATE.
DR   DrugBank; DB02103; 2-Chlorodideoxyadenosine.
DR   DrugBank; DB09130; Copper.
DR   DrugBank; DB02935; Diglyme.
DR   DrugBank; DB02424; Geldanamycin.
DR   DrugBank; DB08464; METHYL 3-CHLORO-2-{3-[(2,5-DIHYDROXY-4-METHOXYPHENYL)AMINO]-3-OXOPROPYL}-4,6-DIHYDROXYBENZOATE.
DR   DrugBank; DB03719; N-Ethyl-5'-Carboxamido Adenosine.
DR   DrugBank; DB03758; Radicicol.
DR   DrugBank; DB00615; Rifabutin.
DR   GuidetoPHARMACOLOGY; 2904; -.
DR   GlyConnect; 1206; 15 N-Linked glycans (5 sites).
DR   GlyGen; P14625; 8 sites.
DR   iPTMnet; P14625; -.
DR   MetOSite; P14625; -.
DR   PhosphoSitePlus; P14625; -.
DR   SwissPalm; P14625; -.
DR   BioMuta; HSP90B1; -.
DR   DMDM; 119360; -.
DR   DOSAC-COBS-2DPAGE; P14625; -.
DR   OGP; P14625; -.
DR   REPRODUCTION-2DPAGE; IPI00027230; -.
DR   CPTAC; CPTAC-1278; -.
DR   CPTAC; CPTAC-1279; -.
DR   EPD; P14625; -.
DR   jPOST; P14625; -.
DR   MassIVE; P14625; -.
DR   PaxDb; P14625; -.
DR   PeptideAtlas; P14625; -.
DR   PRIDE; P14625; -.
DR   ProteomicsDB; 53067; -.
DR   TopDownProteomics; P14625; -.
DR   ABCD; P14625; 4 sequenced antibodies.
DR   Antibodypedia; 1290; 1359 antibodies.
DR   DNASU; 7184; -.
DR   Ensembl; ENST00000299767; ENSP00000299767; ENSG00000166598.
DR   GeneID; 7184; -.
DR   KEGG; hsa:7184; -.
DR   CTD; 7184; -.
DR   DisGeNET; 7184; -.
DR   GeneCards; HSP90B1; -.
DR   HGNC; HGNC:12028; HSP90B1.
DR   HPA; ENSG00000166598; Tissue enhanced (liver, thyroid gland).
DR   MIM; 191175; gene.
DR   neXtProt; NX_P14625; -.
DR   OpenTargets; ENSG00000166598; -.
DR   PharmGKB; PA36705; -.
DR   VEuPathDB; HostDB:ENSG00000166598.12; -.
DR   eggNOG; KOG0020; Eukaryota.
DR   GeneTree; ENSGT01020000230401; -.
DR   HOGENOM; CLU_006684_1_3_1; -.
DR   InParanoid; P14625; -.
DR   OMA; MRRMKEM; -.
DR   OrthoDB; 924636at2759; -.
DR   PhylomeDB; P14625; -.
DR   TreeFam; TF105969; -.
DR   PathwayCommons; P14625; -.
DR   Reactome; R-HSA-1679131; Trafficking and processing of endosomal TLR.
DR   Reactome; R-HSA-3000480; Scavenging by Class A Receptors.
DR   Reactome; R-HSA-381183; ATF6 (ATF6-alpha) activates chaperone genes.
DR   Reactome; R-HSA-381426; Regulation of Insulin-like Growth Factor (IGF) transport and uptake by Insulin-like Growth Factor Binding Proteins (IGFBPs).
DR   Reactome; R-HSA-6785807; Interleukin-4 and Interleukin-13 signaling.
DR   Reactome; R-HSA-8957275; Post-translational protein phosphorylation.
DR   SIGNOR; P14625; -.
DR   BioGRID-ORCS; 7184; 387 hits in 1001 CRISPR screens.
DR   ChiTaRS; HSP90B1; human.
DR   GeneWiki; HSP90B1; -.
DR   GenomeRNAi; 7184; -.
DR   Pharos; P14625; Tchem.
DR   PRO; PR:P14625; -.
DR   Proteomes; UP000005640; Chromosome 12.
DR   RNAct; P14625; protein.
DR   Bgee; ENSG00000166598; Expressed in testis and 253 other tissues.
DR   ExpressionAtlas; P14625; baseline and differential.
DR   Genevisible; P14625; HS.
DR   GO; GO:0062023; C:collagen-containing extracellular matrix; HDA:UniProtKB.
DR   GO; GO:0005829; C:cytosol; IDA:UniProtKB.
DR   GO; GO:0071682; C:endocytic vesicle lumen; TAS:Reactome.
DR   GO; GO:0005783; C:endoplasmic reticulum; IDA:UniProtKB.
DR   GO; GO:0005788; C:endoplasmic reticulum lumen; IDA:UniProtKB.
DR   GO; GO:0005789; C:endoplasmic reticulum membrane; IDA:UniProtKB.
DR   GO; GO:0070062; C:extracellular exosome; HDA:UniProtKB.
DR   GO; GO:0005576; C:extracellular region; TAS:Reactome.
DR   GO; GO:0005925; C:focal adhesion; HDA:UniProtKB.
DR   GO; GO:0042470; C:melanosome; IEA:UniProtKB-SubCell.
DR   GO; GO:0016020; C:membrane; HDA:UniProtKB.
DR   GO; GO:0030496; C:midbody; IDA:UniProtKB.
DR   GO; GO:0005634; C:nucleus; HDA:UniProtKB.
DR   GO; GO:0048471; C:perinuclear region of cytoplasm; IDA:UniProtKB.
DR   GO; GO:0032991; C:protein-containing complex; IDA:UniProtKB.
DR   GO; GO:0033018; C:sarcoplasmic reticulum lumen; IEA:UniProtKB-SubCell.
DR   GO; GO:0005524; F:ATP binding; IEA:UniProtKB-KW.
DR   GO; GO:0016887; F:ATPase activity; IEA:InterPro.
DR   GO; GO:0005509; F:calcium ion binding; TAS:UniProtKB.
DR   GO; GO:0050750; F:low-density lipoprotein particle receptor binding; IDA:MGI.
DR   GO; GO:0019903; F:protein phosphatase binding; IDA:MGI.
DR   GO; GO:0003723; F:RNA binding; IDA:UniProtKB.
DR   GO; GO:0051082; F:unfolded protein binding; IBA:GO_Central.
DR   GO; GO:0031247; P:actin rod assembly; IDA:MGI.
DR   GO; GO:0036500; P:ATF6-mediated unfolded protein response; TAS:Reactome.
DR   GO; GO:0044267; P:cellular protein metabolic process; TAS:Reactome.
DR   GO; GO:0071318; P:cellular response to ATP; IDA:MGI.
DR   GO; GO:0019221; P:cytokine-mediated signaling pathway; TAS:Reactome.
DR   GO; GO:0043066; P:negative regulation of apoptotic process; IMP:UniProtKB.
DR   GO; GO:0043687; P:post-translational protein modification; TAS:Reactome.
DR   GO; GO:0006457; P:protein folding; IBA:GO_Central.
DR   GO; GO:0034975; P:protein folding in endoplasmic reticulum; TAS:ParkinsonsUK-UCL.
DR   GO; GO:0015031; P:protein transport; NAS:UniProtKB.
DR   GO; GO:0006898; P:receptor-mediated endocytosis; TAS:Reactome.
DR   GO; GO:0043666; P:regulation of phosphoprotein phosphatase activity; IDA:MGI.
DR   GO; GO:0034976; P:response to endoplasmic reticulum stress; TAS:ParkinsonsUK-UCL.
DR   GO; GO:0001666; P:response to hypoxia; IDA:UniProtKB.
DR   GO; GO:0030970; P:retrograde protein transport, ER to cytosol; IMP:ParkinsonsUK-UCL.
DR   GO; GO:0051208; P:sequestering of calcium ion; NAS:UniProtKB.
DR   GO; GO:0002224; P:toll-like receptor signaling pathway; TAS:Reactome.
DR   GO; GO:0030433; P:ubiquitin-dependent ERAD pathway; IMP:UniProtKB.
DR   Gene3D; 1.20.120.790; -; 1.
DR   Gene3D; 3.30.565.10; -; 1.
DR   HAMAP; MF_00505; HSP90; 1.
DR   InterPro; IPR003594; HATPase_C.
DR   InterPro; IPR036890; HATPase_C_sf.
DR   InterPro; IPR019805; Heat_shock_protein_90_CS.
DR   InterPro; IPR037196; HSP90_C.
DR   InterPro; IPR001404; Hsp90_fam.
DR   InterPro; IPR020575; Hsp90_N.
DR   InterPro; IPR020568; Ribosomal_S5_D2-typ_fold.
DR   PANTHER; PTHR11528; PTHR11528; 1.
DR   Pfam; PF02518; HATPase_c; 1.
DR   Pfam; PF00183; HSP90; 1.
DR   PIRSF; PIRSF002583; Hsp90; 1.
DR   PRINTS; PR00775; HEATSHOCK90.
DR   SMART; SM00387; HATPase_c; 1.
DR   SUPFAM; SSF110942; SSF110942; 1.
DR   SUPFAM; SSF54211; SSF54211; 1.
DR   SUPFAM; SSF55874; SSF55874; 1.
DR   PROSITE; PS00014; ER_TARGET; 1.
DR   PROSITE; PS00298; HSP90; 1.
PE   1: Evidence at protein level;
KW   3D-structure; Acetylation; ATP-binding; Calcium; Chaperone;
KW   Direct protein sequencing; Disulfide bond; Endoplasmic reticulum;
KW   Glycoprotein; Nucleotide-binding; Phosphoprotein; Reference proteome;
KW   Sarcoplasmic reticulum; Signal.
FT   SIGNAL          1..21
FT                   /evidence="ECO:0000269|PubMed:12665801,
FT                   ECO:0007744|PubMed:25944712"
FT   CHAIN           22..803
FT                   /note="Endoplasmin"
FT                   /id="PRO_0000013598"
FT   REGION          288..323
FT                   /note="Disordered"
FT                   /evidence="ECO:0000256|SAM:MobiDB-lite"
FT   REGION          750..803
FT                   /note="Disordered"
FT                   /evidence="ECO:0000256|SAM:MobiDB-lite"
FT   MOTIF           800..803
FT                   /note="Prevents secretion from ER"
FT   COMPBIAS        290..314
FT                   /note="Acidic residues"
FT                   /evidence="ECO:0000256|SAM:MobiDB-lite"
FT   COMPBIAS        754..790
FT                   /note="Acidic residues"
FT                   /evidence="ECO:0000256|SAM:MobiDB-lite"
FT   BINDING         107
FT                   /note="ATP"
FT                   /evidence="ECO:0000250|UniProtKB:P41148"
FT   BINDING         149
FT                   /note="ATP"
FT                   /evidence="ECO:0000250|UniProtKB:P41148"
FT   BINDING         162
FT                   /note="ATP"
FT                   /evidence="ECO:0000250|UniProtKB:P41148"
FT   BINDING         199
FT                   /note="ATP; via amide nitrogen"
FT                   /evidence="ECO:0000250|UniProtKB:P41148"
FT   SITE            448
FT                   /note="Important for ATP hydrolysis"
FT                   /evidence="ECO:0000250|UniProtKB:P41148"
FT   MOD_RES         64
FT                   /note="Phosphoserine"
FT                   /evidence="ECO:0007744|PubMed:24275569"
FT   MOD_RES         172
FT                   /note="Phosphoserine"
FT                   /evidence="ECO:0000250|UniProtKB:Q66HD0"
FT   MOD_RES         306
FT                   /note="Phosphoserine; by FAM20C"
FT                   /evidence="ECO:0000269|PubMed:26091039,
FT                   ECO:0007744|PubMed:20068231, ECO:0007744|PubMed:21406692,
FT                   ECO:0007744|PubMed:23186163"
FT   MOD_RES         403
FT                   /note="Phosphoserine"
FT                   /evidence="ECO:0000250|UniProtKB:Q66HD0"
FT   MOD_RES         404
FT                   /note="N6-succinyllysine"
FT                   /evidence="ECO:0000250|UniProtKB:P08113"
FT   MOD_RES         447
FT                   /note="Phosphoserine"
FT                   /evidence="ECO:0007744|PubMed:24275569"
FT   MOD_RES         479
FT                   /note="N6-acetyllysine"
FT                   /evidence="ECO:0000250|UniProtKB:P08113"
FT   MOD_RES         633
FT                   /note="N6-succinyllysine"
FT                   /evidence="ECO:0000250|UniProtKB:P08113"
FT   MOD_RES         786
FT                   /note="Phosphothreonine"
FT                   /evidence="ECO:0007744|PubMed:24275569"
FT   CARBOHYD        62
FT                   /note="N-linked (GlcNAc...) asparagine"
FT                   /evidence="ECO:0000269|PubMed:19159218"
FT   CARBOHYD        107
FT                   /note="N-linked (GlcNAc...) asparagine"
FT                   /evidence="ECO:0000269|PubMed:16263699,
FT                   ECO:0000269|PubMed:19159218"
FT   CARBOHYD        217
FT                   /note="N-linked (GlcNAc...) asparagine"
FT                   /evidence="ECO:0000269|PubMed:12754519,
FT                   ECO:0000269|PubMed:19159218"
FT   CARBOHYD        445
FT                   /note="N-linked (GlcNAc...) asparagine"
FT                   /evidence="ECO:0000269|PubMed:12754519,
FT                   ECO:0000269|PubMed:19159218"
FT   CARBOHYD        481
FT                   /note="N-linked (GlcNAc...) asparagine"
FT                   /evidence="ECO:0000255"
FT   CARBOHYD        502
FT                   /note="N-linked (GlcNAc...) asparagine"
FT                   /evidence="ECO:0000255"
FT   DISULFID        138
FT                   /note="Interchain"
FT                   /evidence="ECO:0000250"
FT   CONFLICT        188
FT                   /note="T -> S (in Ref. 4; AAK74072)"
FT                   /evidence="ECO:0000305"
FT   CONFLICT        419
FT                   /note="T -> P (in Ref. 4; AAK74072)"
FT                   /evidence="ECO:0000305"
FT   CONFLICT        803
FT                   /note="L -> F (in Ref. 4; AAK74072)"
FT                   /evidence="ECO:0000305"
FT   HELIX           80..92
FT                   /evidence="ECO:0007829|PDB:4NH9"
FT   HELIX           99..118
FT                   /evidence="ECO:0007829|PDB:4NH9"
FT   TURN            119..121
FT                   /evidence="ECO:0007829|PDB:4NH9"
FT   TURN            123..128
FT                   /evidence="ECO:0007829|PDB:4NH9"
FT   STRAND          134..139
FT                   /evidence="ECO:0007829|PDB:4NH9"
FT   TURN            140..143
FT                   /evidence="ECO:0007829|PDB:4NH9"
FT   STRAND          144..149
FT                   /evidence="ECO:0007829|PDB:4NH9"
FT   HELIX           156..160
FT                   /evidence="ECO:0007829|PDB:4NH9"
FT   HELIX           175..184
FT                   /evidence="ECO:0007829|PDB:4NH9"
FT   HELIX           189..194
FT                   /evidence="ECO:0007829|PDB:4NH9"
FT   HELIX           198..204
FT                   /evidence="ECO:0007829|PDB:4NH9"
FT   STRAND          206..214
FT                   /evidence="ECO:0007829|PDB:4NH9"
FT   STRAND          221..225
FT                   /evidence="ECO:0007829|PDB:4NH9"
FT   STRAND          230..234
FT                   /evidence="ECO:0007829|PDB:4NH9"
FT   STRAND          241..251
FT                   /evidence="ECO:0007829|PDB:4NH9"
FT   HELIX           256..259
FT                   /evidence="ECO:0007829|PDB:4NH9"
FT   HELIX           261..271
FT                   /evidence="ECO:0007829|PDB:4NH9"
FT   STRAND          279..284
FT                   /evidence="ECO:0007829|PDB:4NH9"
FT   STRAND          331..335
FT                   /evidence="ECO:0007829|PDB:4NH9"
SQ   SEQUENCE   803 AA;  92469 MW;  9BF6705A7A2ED0D0 CRC64;
     MRALWVLGLC CVLLTFGSVR ADDEVDVDGT VEEDLGKSRE GSRTDDEVVQ REEEAIQLDG
     LNASQIRELR EKSEKFAFQA EVNRMMKLII NSLYKNKEIF LRELISNASD ALDKIRLISL
     TDENALSGNE ELTVKIKCDK EKNLLHVTDT GVGMTREELV KNLGTIAKSG TSEFLNKMTE
     AQEDGQSTSE LIGQFGVGFY SAFLVADKVI VTSKHNNDTQ HIWESDSNEF SVIADPRGNT
     LGRGTTITLV LKEEASDYLE LDTIKNLVKK YSQFINFPIY VWSSKTETVE EPMEEEEAAK
     EEKEESDDEA AVEEEEEEKK PKTKKVEKTV WDWELMNDIK PIWQRPSKEV EEDEYKAFYK
     SFSKESDDPM AYIHFTAEGE VTFKSILFVP TSAPRGLFDE YGSKKSDYIK LYVRRVFITD
     DFHDMMPKYL NFVKGVVDSD DLPLNVSRET LQQHKLLKVI RKKLVRKTLD MIKKIADDKY
     NDTFWKEFGT NIKLGVIEDH SNRTRLAKLL RFQSSHHPTD ITSLDQYVER MKEKQDKIYF
     MAGSSRKEAE SSPFVERLLK KGYEVIYLTE PVDEYCIQAL PEFDGKRFQN VAKEGVKFDE
     SEKTKESREA VEKEFEPLLN WMKDKALKDK IEKAVVSQRL TESPCALVAS QYGWSGNMER
     IMKAQAYQTG KDISTNYYAS QKKTFEINPR HPLIRDMLRR IKEDEDDKTV LDLAVVLFET
     ATLRSGYLLP DTKAYGDRIE RMLRLSLNID PDAKVEEEPE EEPEETAEDT TEDTEQDEDE
     EMDVGTDEEE ETAKESTAEK DEL
```

|  |
| --- |
| **Mascot:** http://www.matrixscience.com/ |
