# Supplementary material for: Chlorpromazine induces cytotoxic autophagy in glioblastoma cells via endoplasmic reticulum stress and unfolded protein response
Source: J Exp Clin Cancer Res. 2021 Nov 5;40:347. doi: 10.1186/s13046-021-02144-w (PMC8569984; doi:10.1186/s13046-021-02144-w)
Supplement: Supplementary file 1 — Additional file 1. [file 13046_2021_2144_MOESM1_ESM.zip › MASCOT IDs/Mascot Search Results_ BIP_HUMAN.html]

Mascot Search Results: BIP\_HUMAN
 

# MASCOT Search Results

## Protein View: BIP\_HUMAN

### Endoplasmic reticulum chaperone BiP OS=Homo sapiens OX=9606 GN=HSPA5 PE=1 SV=2

|  |  |
| --- | --- |
| Database: | SwissProt |
| Score: | 58 |
| Monoisotopic mass (Mr): | 72402 |
| Calculated pI: | 5.07 |
| Taxonomy: | Homo sapiens |

Sequence similarity is available as an NCBI BLAST search of BIP\_HUMAN against nr.

### Search parameters

|  |  |
| --- | --- |
| MS data file: | `ppw_D17_157002891600.txt` |
| Enzyme: | Trypsin: cuts C-term side of KR unless next residue is P. |
| Fixed modifications: | Carbamidomethyl (C) |

### Protein sequence coverage: 2%

Matched peptides shown in ***bold red***.

|  |  |  |  |  |  |
| --- | --- | --- | --- | --- | --- |
| `1` | `MKLSLVAAML` | `LLLSAARAEE` | `EDKKEDVGTV` | `VGIDLGTTYS` | `CVGVFKNGRV` |
| `51` | `EIIANDQGNR` | `ITPSYVAFTP` | `EGERLIGDAA` | `KNQLTSNPEN` | `TVFDAKRLIG` |
| `101` | `RTWNDPSVQQ` | `DIKFLPFKVV` | `EKKTKPYIQV` | `DIGGGQTKTF` | `APEEISAMVL` |
| `151` | `TKMKETAEAY` | `LGKKVTHAVV` | `TVPAYFNDAQ` | `RQATKDAGTI` | `AGLNVMRIIN` |
| `201` | `EPTAAAIAYG` | `LDKREGEKNI` | `LVFDLGGGTF` | `DVSLLTIDNG` | `VFEVVATNGD` |
| `251` | `THLGGEDFDQ` | `RVMEHFIKLY` | `KKKTGKDVRK` | `DNRAVQKLRR` | `EVEKAKRALS` |
| `301` | `SQHQARIEIE` | `SFYEGEDFSE` | `TLTRAKFEEL` | `NMDLFRSTMK` | `PVQKVLEDSD` |
| `351` | `LKKSDIDEIV` | `LVGGSTRIPK` | `IQQLVKEFFN` | `GKEPSRGINP` | `DEAVAYGAAV` |
| `401` | `QAGVLSGDQD` | `TGDLVLLDVC` | `PLTLGIETVG` | `GVMTKLIPRN` | `TVVPTKKSQI` |
| `451` | `FSTASDNQPT` | `VTIKVYEGER` | `PLTKDNHLLG` | `TFDLTGIPPA` | `PRGVPQIEVT` |
| `501` | `FEIDVNGILR` | `VTAEDKGTGN` | `KNKITITNDQ` | `NRLTPEEIER` | `MVNDAEKFAE` |
| `551` | `EDKKLKERID` | `TRNELESYAY` | `SLKNQIGDKE` | `KLGGKLSSED` | `KETMEKAVEE` |
| `601` | `KIEWLESHQD` | `ADIEDFKAKK` | `KELEEIVQPI` | `ISKLYGSAGP` | `PPTGEEDTAE` |
| `651` | `KDEL` |  |  |  |  |

Unformatted sequence string: 654 residues (for pasting into other applications).

|  |  |  |  |
| --- | --- | --- | --- |
| Sort by | residue number | increasing mass | decreasing mass |
| Show | matched peptides only | predicted peptides also |  |

| Query | Start | – | End | Observed | Mr(expt) | Mr(calc) | ppm | M | Score | Expect | Rank | U | Peptide |
| --- | --- | --- | --- | --- | --- | --- | --- | --- | --- | --- | --- | --- | --- |
| 61 | 165 | – | 181 | 1888.0291 | 1887.0218 | 1886.9639 | 30.7 | 0 | 36 | 0.0077 | 1Score **> 27** indicates **identity** Score **> 18** indicates **homology** | U | K.VTHAVVTVPAYFNDAQR.Q |
| 62 | 165 | – | 181 | 1888.0291 | 1887.0218 | 1886.9639 | 30.7 | 0 | 41 | 0.0021 | 1Score **> 27** indicates **identity** Score **> 20** indicates **homology** | U | K.VTHAVVTVPAYFNDAQR.Q |

---

```
ID   BIP_HUMAN               Reviewed;         654 AA.
AC   P11021; B0QZ61; Q2EF78; Q9NPF1; Q9UK02;
DT   01-JUL-1989, integrated into UniProtKB/Swiss-Prot.
DT   11-JUL-2001, sequence version 2.
DT   02-JUN-2021, entry version 238.
DE   RecName: Full=Endoplasmic reticulum chaperone BiP {ECO:0000305};
DE            EC=3.6.4.10 {ECO:0000269|PubMed:26655470};
DE   AltName: Full=78 kDa glucose-regulated protein {ECO:0000303|PubMed:2840249};
DE            Short=GRP-78 {ECO:0000303|PubMed:2840249};
DE   AltName: Full=Binding-immunoglobulin protein {ECO:0000303|Ref.4};
DE            Short=BiP {ECO:0000303|Ref.4};
DE   AltName: Full=Heat shock protein 70 family protein 5 {ECO:0000305};
DE            Short=HSP70 family protein 5 {ECO:0000305};
DE   AltName: Full=Heat shock protein family A member 5 {ECO:0000312|HGNC:HGNC:5238};
DE   AltName: Full=Immunoglobulin heavy chain-binding protein {ECO:0000303|Ref.4};
DE   Flags: Precursor;
GN   Name=HSPA5 {ECO:0000312|HGNC:HGNC:5238};
GN   Synonyms=GRP78 {ECO:0000303|PubMed:2840249};
OS   Homo sapiens (Human).
OC   Eukaryota; Metazoa; Chordata; Craniata; Vertebrata; Euteleostomi; Mammalia;
OC   Eutheria; Euarchontoglires; Primates; Haplorrhini; Catarrhini; Hominidae;
OC   Homo.
OX   NCBI_TaxID=9606;
RN   [1]
RP   NUCLEOTIDE SEQUENCE [GENOMIC DNA].
RX   PubMed=2840249; DOI=10.1089/dna.1988.7.275;
RA   Ting J., Lee A.S.;
RT   "Human gene encoding the 78,000-dalton glucose-regulated protein and its
RT   pseudogene: structure, conservation, and regulation.";
RL   DNA 7:275-286(1988).
RN   [2]
RP   NUCLEOTIDE SEQUENCE [MRNA].
RC   TISSUE=Cervix carcinoma;
RA   Chao C.C.K.;
RL   Submitted (DEC-1995) to the EMBL/GenBank/DDBJ databases.
RN   [3]
RP   NUCLEOTIDE SEQUENCE [MRNA].
RC   TISSUE=Fibroblast;
RA   Hansen J.J., Nielsen M.N., Jorgensen M.M., Gregersen N., Bolund L.;
RT   "Grp78 is involved in the quality control of the LDL-receptor.";
RL   Submitted (JAN-2000) to the EMBL/GenBank/DDBJ databases.
RN   [4]
RP   NUCLEOTIDE SEQUENCE [MRNA].
RA   Bermudez-Fajardo A., Llewellyn D.H., Campbell A.K., Errington R.R.;
RT   "Sequence differences between human grp78/BiP isolated from HeLa cells and
RT   previously reported human sequences.";
RL   Submitted (DEC-1999) to the EMBL/GenBank/DDBJ databases.
RN   [5]
RP   NUCLEOTIDE SEQUENCE [GENOMIC DNA], AND VARIANT HIS-543.
RG   NIEHS SNPs program;
RL   Submitted (JAN-2006) to the EMBL/GenBank/DDBJ databases.
RN   [6]
RP   NUCLEOTIDE SEQUENCE [LARGE SCALE GENOMIC DNA].
RX   PubMed=15164053; DOI=10.1038/nature02465;
RA   Humphray S.J., Oliver K., Hunt A.R., Plumb R.W., Loveland J.E., Howe K.L.,
RA   Andrews T.D., Searle S., Hunt S.E., Scott C.E., Jones M.C., Ainscough R.,
RA   Almeida J.P., Ambrose K.D., Ashwell R.I.S., Babbage A.K., Babbage S.,
RA   Bagguley C.L., Bailey J., Banerjee R., Barker D.J., Barlow K.F., Bates K.,
RA   Beasley H., Beasley O., Bird C.P., Bray-Allen S., Brown A.J., Brown J.Y.,
RA   Burford D., Burrill W., Burton J., Carder C., Carter N.P., Chapman J.C.,
RA   Chen Y., Clarke G., Clark S.Y., Clee C.M., Clegg S., Collier R.E.,
RA   Corby N., Crosier M., Cummings A.T., Davies J., Dhami P., Dunn M.,
RA   Dutta I., Dyer L.W., Earthrowl M.E., Faulkner L., Fleming C.J.,
RA   Frankish A., Frankland J.A., French L., Fricker D.G., Garner P.,
RA   Garnett J., Ghori J., Gilbert J.G.R., Glison C., Grafham D.V., Gribble S.,
RA   Griffiths C., Griffiths-Jones S., Grocock R., Guy J., Hall R.E.,
RA   Hammond S., Harley J.L., Harrison E.S.I., Hart E.A., Heath P.D.,
RA   Henderson C.D., Hopkins B.L., Howard P.J., Howden P.J., Huckle E.,
RA   Johnson C., Johnson D., Joy A.A., Kay M., Keenan S., Kershaw J.K.,
RA   Kimberley A.M., King A., Knights A., Laird G.K., Langford C., Lawlor S.,
RA   Leongamornlert D.A., Leversha M., Lloyd C., Lloyd D.M., Lovell J.,
RA   Martin S., Mashreghi-Mohammadi M., Matthews L., McLaren S., McLay K.E.,
RA   McMurray A., Milne S., Nickerson T., Nisbett J., Nordsiek G., Pearce A.V.,
RA   Peck A.I., Porter K.M., Pandian R., Pelan S., Phillimore B., Povey S.,
RA   Ramsey Y., Rand V., Scharfe M., Sehra H.K., Shownkeen R., Sims S.K.,
RA   Skuce C.D., Smith M., Steward C.A., Swarbreck D., Sycamore N., Tester J.,
RA   Thorpe A., Tracey A., Tromans A., Thomas D.W., Wall M., Wallis J.M.,
RA   West A.P., Whitehead S.L., Willey D.L., Williams S.A., Wilming L.,
RA   Wray P.W., Young L., Ashurst J.L., Coulson A., Blocker H., Durbin R.M.,
RA   Sulston J.E., Hubbard T., Jackson M.J., Bentley D.R., Beck S., Rogers J.,
RA   Dunham I.;
RT   "DNA sequence and analysis of human chromosome 9.";
RL   Nature 429:369-374(2004).
RN   [7]
RP   NUCLEOTIDE SEQUENCE [LARGE SCALE GENOMIC DNA].
RA   Mural R.J., Istrail S., Sutton G.G., Florea L., Halpern A.L., Mobarry C.M.,
RA   Lippert R., Walenz B., Shatkay H., Dew I., Miller J.R., Flanigan M.J.,
RA   Edwards N.J., Bolanos R., Fasulo D., Halldorsson B.V., Hannenhalli S.,
RA   Turner R., Yooseph S., Lu F., Nusskern D.R., Shue B.C., Zheng X.H.,
RA   Zhong F., Delcher A.L., Huson D.H., Kravitz S.A., Mouchard L., Reinert K.,
RA   Remington K.A., Clark A.G., Waterman M.S., Eichler E.E., Adams M.D.,
RA   Hunkapiller M.W., Myers E.W., Venter J.C.;
RL   Submitted (JUL-2005) to the EMBL/GenBank/DDBJ databases.
RN   [8]
RP   NUCLEOTIDE SEQUENCE [LARGE SCALE MRNA].
RC   TISSUE=Muscle;
RX   PubMed=15489334; DOI=10.1101/gr.2596504;
RG   The MGC Project Team;
RT   "The status, quality, and expansion of the NIH full-length cDNA project:
RT   the Mammalian Gene Collection (MGC).";
RL   Genome Res. 14:2121-2127(2004).
RN   [9]
RP   NUCLEOTIDE SEQUENCE [GENOMIC DNA] OF 1-25.
RX   PubMed=1480470; DOI=10.1093/nar/20.24.6481;
RA   Chao C.C.K., Lin-Chao S.;
RT   "A direct-repeat sequence of the human BiP gene is required for A23187-
RT   mediated inducibility and an inducible nuclear factor binding.";
RL   Nucleic Acids Res. 20:6481-6485(1992).
RN   [10]
RP   NUCLEOTIDE SEQUENCE [MRNA] OF 20-650, AND DISEASE.
RC   TISSUE=Articular cartilage;
RX   PubMed=11160188; DOI=10.4049/jimmunol.166.3.1492;
RA   Corrigall V.M., Bodman-Smith M.D., Fife M.S., Canas B., Myers L.K.,
RA   Wooley P., Soh C., Staines N.A., Pappin D.J.C., Berlo S.E., van Eden W.,
RA   van Der Zee R., Lanchbury J.S., Panayi G.S.;
RT   "The human endoplasmic reticulum molecular chaperone BiP is an autoantigen
RT   for rheumatoid arthritis and prevents the induction of experimental
RT   arthritis.";
RL   J. Immunol. 166:1492-1498(2001).
RN   [11]
RP   PROTEIN SEQUENCE OF 22-38.
RC   TISSUE=Mammary carcinoma;
RX   PubMed=9150946; DOI=10.1002/elps.1150180342;
RA   Rasmussen R.K., Ji H., Eddes J.S., Moritz R.L., Reid G.E., Simpson R.J.,
RA   Dorow D.S.;
RT   "Two-dimensional electrophoretic analysis of human breast carcinoma
RT   proteins: mapping of proteins that bind to the SH3 domain of mixed lineage
RT   kinase MLK2.";
RL   Electrophoresis 18:588-598(1997).
RN   [12]
RP   PROTEIN SEQUENCE OF 19-39, AND FUNCTION.
RX   PubMed=2294010; DOI=10.1210/endo-126-1-672;
RA   Dana R.C., Welch W.J., Deftos L.J.;
RT   "Heat shock proteins bind calcitonin.";
RL   Endocrinology 126:672-674(1990).
RN   [13]
RP   PROTEIN SEQUENCE OF 19-40.
RC   TISSUE=Colon carcinoma;
RX   PubMed=9150948; DOI=10.1002/elps.1150180344;
RA   Ji H., Reid G.E., Moritz R.L., Eddes J.S., Burgess A.W., Simpson R.J.;
RT   "A two-dimensional gel database of human colon carcinoma proteins.";
RL   Electrophoresis 18:605-613(1997).
RN   [14]
RP   PROTEIN SEQUENCE OF 61-74; 82-96; 102-113; 124-152; 164-181; 186-214;
RP   307-336; 353-367; 448-464; 475-492; 563-573; 602-617 AND 622-654, AND
RP   IDENTIFICATION BY MASS SPECTROMETRY.
RC   TISSUE=Brain, Cajal-Retzius cell, and Fetal brain cortex;
RA   Lubec G., Vishwanath V., Chen W.-Q., Sun Y.;
RL   Submitted (DEC-2008) to UniProtKB.
RN   [15]
RP   FUNCTION.
RX   PubMed=1550958; DOI=10.1091/mbc.3.2.143;
RA   Ng D.T., Watowich S.S., Lamb R.A.;
RT   "Analysis in vivo of GRP78-BiP/substrate interactions and their role in
RT   induction of the GRP78-BiP gene.";
RL   Mol. Biol. Cell 3:143-155(1992).
RN   [16]
RP   PHOSPHORYLATION [LARGE SCALE ANALYSIS] AT THR-518, AND IDENTIFICATION BY
RP   MASS SPECTROMETRY [LARGE SCALE ANALYSIS].
RC   TISSUE=Cervix carcinoma;
RX   PubMed=20068231; DOI=10.1126/scisignal.2000475;
RA   Olsen J.V., Vermeulen M., Santamaria A., Kumar C., Miller M.L.,
RA   Jensen L.J., Gnad F., Cox J., Jensen T.S., Nigg E.A., Brunak S., Mann M.;
RT   "Quantitative phosphoproteomics reveals widespread full phosphorylation
RT   site occupancy during mitosis.";
RL   Sci. Signal. 3:RA3-RA3(2010).
RN   [17]
RP   IDENTIFICATION BY MASS SPECTROMETRY [LARGE SCALE ANALYSIS].
RX   PubMed=21269460; DOI=10.1186/1752-0509-5-17;
RA   Burkard T.R., Planyavsky M., Kaupe I., Breitwieser F.P., Buerckstuemmer T.,
RA   Bennett K.L., Superti-Furga G., Colinge J.;
RT   "Initial characterization of the human central proteome.";
RL   BMC Syst. Biol. 5:17-17(2011).
RN   [18]
RP   INTERACTION WITH CCDC88B, SUBCELLULAR LOCATION, AND INDUCTION BY ER STRESS.
RX   PubMed=21289099; DOI=10.1091/mbc.e10-08-0724;
RA   Matsushita E., Asai N., Enomoto A., Kawamoto Y., Kato T., Mii S., Maeda K.,
RA   Shibata R., Hattori S., Hagikura M., Takahashi K., Sokabe M., Murakumo Y.,
RA   Murohara T., Takahashi M.;
RT   "Protective role of Gipie, a Girdin family protein, in endoplasmic
RT   reticulum stress responses in endothelial cells.";
RL   Mol. Biol. Cell 22:736-747(2011).
RN   [19]
RP   PROTEIN SEQUENCE OF 582-596, INTERACTION WITH METTL23, METHYLATION AT
RP   LYS-585, MUTAGENESIS OF LYS-585, AND IDENTIFICATION BY MASS SPECTROMETRY.
RX   PubMed=23349634; DOI=10.1371/journal.pgen.1003210;
RA   Cloutier P., Lavallee-Adam M., Faubert D., Blanchette M., Coulombe B.;
RT   "A newly uncovered group of distantly related lysine methyltransferases
RT   preferentially interact with molecular chaperones to regulate their
RT   activity.";
RL   PLoS Genet. 9:E1003210-E1003210(2013).
RN   [20]
RP   COMPONENT OF A CHAPERONE COMPLEX.
RX   PubMed=12475965; DOI=10.1091/mbc.e02-05-0311;
RA   Meunier L., Usherwood Y.-K., Chung K.T., Hendershot L.M.;
RT   "A subset of chaperones and folding enzymes form multiprotein complexes in
RT   endoplasmic reticulum to bind nascent proteins.";
RL   Mol. Biol. Cell 13:4456-4469(2002).
RN   [21]
RP   INTERACTION WITH TRIM21.
RX   PubMed=12699405; DOI=10.1046/j.1365-2249.2003.02153.x;
RA   Purcell A.W., Todd A., Kinoshita G., Lynch T.A., Keech C.L., Gething M.J.,
RA   Gordon T.P.;
RT   "Association of stress proteins with autoantigens: a possible mechanism for
RT   triggering autoimmunity?";
RL   Clin. Exp. Immunol. 132:193-200(2003).
RN   [22]
RP   INTERACTION WITH DNAJC10.
RX   PubMed=12411443; DOI=10.1074/jbc.m206995200;
RA   Cunnea P.M., Miranda-Vizuete A., Bertoli G., Simmen T., Damdimopoulos A.E.,
RA   Hermann S., Leinonen S., Huikko M.P., Gustafsson J.-A., Sitia R.,
RA   Spyrou G.;
RT   "ERdj5, an endoplasmic reticulum (ER)-resident protein containing DnaJ and
RT   thioredoxin domains, is expressed in secretory cells or following ER
RT   stress.";
RL   J. Biol. Chem. 278:1059-1066(2003).
RN   [23]
RP   SUBCELLULAR LOCATION [LARGE SCALE ANALYSIS].
RC   TISSUE=Melanoma;
RX   PubMed=12643545; DOI=10.1021/pr025562r;
RA   Basrur V., Yang F., Kushimoto T., Higashimoto Y., Yasumoto K., Valencia J.,
RA   Muller J., Vieira W.D., Watabe H., Shabanowitz J., Hearing V.J., Hunt D.F.,
RA   Appella E.;
RT   "Proteomic analysis of early melanosomes: identification of novel
RT   melanosomal proteins.";
RL   J. Proteome Res. 2:69-79(2003).
RN   [24]
RP   FUNCTION (MICROBIAL INFECTION), AND SUBCELLULAR LOCATION.
RX   PubMed=15098107; DOI=10.1007/s00705-003-0263-x;
RA   Jindadamrongwech S., Thepparit C., Smith D.R.;
RT   "Identification of GRP 78 (BiP) as a liver cell expressed receptor element
RT   for dengue virus serotype 2.";
RL   Arch. Virol. 149:915-927(2004).
RN   [25]
RP   INTERACTION WITH CLN3.
RX   PubMed=18621045; DOI=10.1016/j.yexcr.2008.06.016;
RA   Uusi-Rauva K., Luiro K., Tanhuanpaeae K., Kopra O., Martin-Vasallo P.,
RA   Kyttaelae A., Jalanko A.;
RT   "Novel interactions of CLN3 protein link Batten disease to dysregulation of
RT   fodrin-Na+, K+ ATPase complex.";
RL   Exp. Cell Res. 314:2895-2905(2008).
RN   [26]
RP   SUBCELLULAR LOCATION [LARGE SCALE ANALYSIS].
RC   TISSUE=Melanoma;
RX   PubMed=17081065; DOI=10.1021/pr060363j;
RA   Chi A., Valencia J.C., Hu Z.-Z., Watabe H., Yamaguchi H., Mangini N.J.,
RA   Huang H., Canfield V.A., Cheng K.C., Yang F., Abe R., Yamagishi S.,
RA   Shabanowitz J., Hearing V.J., Wu C., Appella E., Hunt D.F.;
RT   "Proteomic and bioinformatic characterization of the biogenesis and
RT   function of melanosomes.";
RL   J. Proteome Res. 5:3135-3144(2006).
RN   [27]
RP   INTERACTION WITH ERLEC1; OS9; SEL1L AND SYVN1.
RX   PubMed=18502753; DOI=10.1074/jbc.m709336200;
RA   Hosokawa N., Wada I., Nagasawa K., Moriyama T., Okawa K., Nagata K.;
RT   "Human XTP3-B forms an endoplasmic reticulum quality control scaffold with
RT   the HRD1-SEL1L ubiquitin ligase complex and BiP.";
RL   J. Biol. Chem. 283:20914-20924(2008).
RN   [28]
RP   INTERACTION WITH ERLEC1; OS9; SEL1L AND SYVN1.
RX   PubMed=18264092; DOI=10.1038/ncb1689;
RA   Christianson J.C., Shaler T.A., Tyler R.E., Kopito R.R.;
RT   "OS-9 and GRP94 deliver mutant alpha1-antitrypsin to the Hrd1-SEL1L
RT   ubiquitin ligase complex for ERAD.";
RL   Nat. Cell Biol. 10:272-282(2008).
RN   [29]
RP   FUNCTION.
RX   PubMed=19538957; DOI=10.1016/j.yexcr.2009.06.009;
RA   Oikawa D., Kimata Y., Kohno K., Iwawaki T.;
RT   "Activation of mammalian IRE1alpha upon ER stress depends on dissociation
RT   of BiP rather than on direct interaction with unfolded proteins.";
RL   Exp. Cell Res. 315:2496-2504(2009).
RN   [30]
RP   INTERACTION WITH PCSK4, AND SUBCELLULAR LOCATION.
RX   PubMed=21080038; DOI=10.1007/s11010-010-0635-y;
RA   Gyamera-Acheampong C., Sirois F., Denis N.J., Mishra P., Figeys D.,
RA   Basak A., Mbikay M.;
RT   "The precursor to the germ cell-specific PCSK4 proteinase is inefficiently
RT   activated in transfected somatic cells: evidence of interaction with the
RT   BiP chaperone.";
RL   Mol. Cell. Biochem. 348:43-52(2011).
RN   [31]
RP   INTERACTION WITH MANF.
RX   PubMed=22637475; DOI=10.1074/jbc.m112.356345;
RA   Glembotski C.C., Thuerauf D.J., Huang C., Vekich J.A., Gottlieb R.A.,
RA   Doroudgar S.;
RT   "Mesencephalic astrocyte-derived neurotrophic factor protects the heart
RT   from ischemic damage and is selectively secreted upon sarco/endoplasmic
RT   reticulum calcium depletion.";
RL   J. Biol. Chem. 287:25893-25904(2012).
RN   [32]
RP   IDENTIFICATION BY MASS SPECTROMETRY [LARGE SCALE ANALYSIS].
RX   PubMed=22905912; DOI=10.1021/pr300539b;
RA   Rosenow A., Noben J.P., Jocken J., Kallendrusch S., Fischer-Posovszky P.,
RA   Mariman E.C., Renes J.;
RT   "Resveratrol-induced changes of the human adipocyte secretion profile.";
RL   J. Proteome Res. 11:4733-4743(2012).
RN   [33]
RP   METHYLATION AT LYS-585, AND MUTAGENESIS OF LYS-585.
RX   PubMed=23921388; DOI=10.1074/jbc.m113.483248;
RA   Jakobsson M.E., Moen A., Bousset L., Egge-Jacobsen W., Kernstock S.,
RA   Melki R., Falnes P.O.;
RT   "Identification and characterization of a novel human methyltransferase
RT   modulating Hsp70 function through lysine methylation.";
RL   J. Biol. Chem. 288:27752-27763(2013).
RN   [34]
RP   FUNCTION, INTERACTION WITH CEMIP, AND SUBCELLULAR LOCATION.
RX   PubMed=23990668; DOI=10.1093/jnci/djt224;
RA   Evensen N.A., Kuscu C., Nguyen H.L., Zarrabi K., Dufour A., Kadam P.,
RA   Hu Y.J., Pulkoski-Gross A., Bahou W.F., Zucker S., Cao J.;
RT   "Unraveling the role of KIAA1199, a novel endoplasmic reticulum protein, in
RT   cancer cell migration.";
RL   J. Natl. Cancer Inst. 105:1402-1416(2013).
RN   [35]
RP   FUNCTION, AND INTERACTION WITH DNAJC10.
RX   PubMed=23769672; DOI=10.1016/j.molcel.2013.05.014;
RA   Oka O.B., Pringle M.A., Schopp I.M., Braakman I., Bulleid N.J.;
RT   "ERdj5 is the ER reductase that catalyzes the removal of non-native
RT   disulfides and correct folding of the LDL receptor.";
RL   Mol. Cell 50:793-804(2013).
RN   [36]
RP   INTERACTION WITH CLU, SUBCELLULAR LOCATION, AND FUNCTION.
RX   PubMed=22689054; DOI=10.1038/onc.2012.212;
RA   Li N., Zoubeidi A., Beraldi E., Gleave M.E.;
RT   "GRP78 regulates clusterin stability, retrotranslocation and mitochondrial
RT   localization under ER stress in prostate cancer.";
RL   Oncogene 32:1933-1942(2013).
RN   [37]
RP   PHOSPHORYLATION [LARGE SCALE ANALYSIS] AT THR-518, AND IDENTIFICATION BY
RP   MASS SPECTROMETRY [LARGE SCALE ANALYSIS].
RC   TISSUE=Liver;
RX   PubMed=24275569; DOI=10.1016/j.jprot.2013.11.014;
RA   Bian Y., Song C., Cheng K., Dong M., Wang F., Huang J., Sun D., Wang L.,
RA   Ye M., Zou H.;
RT   "An enzyme assisted RP-RPLC approach for in-depth analysis of human liver
RT   phosphoproteome.";
RL   J. Proteomics 96:253-262(2014).
RN   [38]
RP   METHYLATION [LARGE SCALE ANALYSIS] AT LYS-585 AND LYS-591, AND
RP   IDENTIFICATION BY MASS SPECTROMETRY [LARGE SCALE ANALYSIS].
RC   TISSUE=Colon carcinoma;
RX   PubMed=24129315; DOI=10.1074/mcp.o113.027870;
RA   Guo A., Gu H., Zhou J., Mulhern D., Wang Y., Lee K.A., Yang V., Aguiar M.,
RA   Kornhauser J., Jia X., Ren J., Beausoleil S.A., Silva J.C., Vemulapalli V.,
RA   Bedford M.T., Comb M.J.;
RT   "Immunoaffinity enrichment and mass spectrometry analysis of protein
RT   methylation.";
RL   Mol. Cell. Proteomics 13:372-387(2014).
RN   [39]
RP   SUMOYLATION [LARGE SCALE ANALYSIS] AT LYS-352, AND IDENTIFICATION BY MASS
RP   SPECTROMETRY [LARGE SCALE ANALYSIS].
RX   PubMed=25218447; DOI=10.1038/nsmb.2890;
RA   Hendriks I.A., D'Souza R.C., Yang B., Verlaan-de Vries M., Mann M.,
RA   Vertegaal A.C.;
RT   "Uncovering global SUMOylation signaling networks in a site-specific
RT   manner.";
RL   Nat. Struct. Mol. Biol. 21:927-936(2014).
RN   [40]
RP   SUMOYLATION [LARGE SCALE ANALYSIS] AT LYS-352 AND LYS-353, AND
RP   IDENTIFICATION BY MASS SPECTROMETRY [LARGE SCALE ANALYSIS].
RX   PubMed=25114211; DOI=10.1073/pnas.1413825111;
RA   Impens F., Radoshevich L., Cossart P., Ribet D.;
RT   "Mapping of SUMO sites and analysis of SUMOylation changes induced by
RT   external stimuli.";
RL   Proc. Natl. Acad. Sci. U.S.A. 111:12432-12437(2014).
RN   [41]
RP   FUNCTION, INTERACTION WITH CASP7 AND KIAA1324, AND REGION.
RX   PubMed=26045166; DOI=10.1158/0008-5472.can-14-3751;
RA   Kang J.M., Park S., Kim S.J., Kim H., Lee B., Kim J., Park J., Kim S.T.,
RA   Yang H.K., Kim W.H., Kim S.J.;
RT   "KIAA1324 Suppresses Gastric Cancer Progression by Inhibiting the
RT   Oncoprotein GRP78.";
RL   Cancer Res. 75:3087-3097(2015).
RN   [42]
RP   AMPYLATION.
RX   PubMed=25601083; DOI=10.1074/jbc.m114.618348;
RA   Sanyal A., Chen A.J., Nakayasu E.S., Lazar C.S., Zbornik E.A., Worby C.A.,
RA   Koller A., Mattoo S.;
RT   "A novel link between Fic (filamentation induced by cAMP)-mediated
RT   adenylylation/AMPylation and the unfolded protein response.";
RL   J. Biol. Chem. 290:8482-8499(2015).
RN   [43]
RP   SUMOYLATION [LARGE SCALE ANALYSIS] AT LYS-352, AND IDENTIFICATION BY MASS
RP   SPECTROMETRY [LARGE SCALE ANALYSIS].
RX   PubMed=25755297; DOI=10.1074/mcp.o114.044792;
RA   Xiao Z., Chang J.G., Hendriks I.A., Sigurdsson J.O., Olsen J.V.,
RA   Vertegaal A.C.;
RT   "System-wide analysis of SUMOylation dynamics in response to replication
RT   stress reveals novel small ubiquitin-like modified target proteins and
RT   acceptor lysines relevant for genome stability.";
RL   Mol. Cell. Proteomics 14:1419-1434(2015).
RN   [44]
RP   IDENTIFICATION BY MASS SPECTROMETRY [LARGE SCALE ANALYSIS].
RX   PubMed=25944712; DOI=10.1002/pmic.201400617;
RA   Vaca Jacome A.S., Rabilloud T., Schaeffer-Reiss C., Rompais M., Ayoub D.,
RA   Lane L., Bairoch A., Van Dorsselaer A., Carapito C.;
RT   "N-terminome analysis of the human mitochondrial proteome.";
RL   Proteomics 15:2519-2524(2015).
RN   [45]
RP   INTERACTION WITH CIPC.
RX   PubMed=26657846; DOI=10.1016/j.bbrc.2015.11.117;
RA   Matsunaga R., Nishino T., Yokoyama A., Nakashima A., Kikkawa U.,
RA   Konishi H.;
RT   "Versatile function of the circadian protein CIPC as a regulator of Erk
RT   activation.";
RL   Biochem. Biophys. Res. Commun. 469:377-383(2016).
RN   [46]
RP   INTERACTION WITH INPP5K.
RX   PubMed=26940976; DOI=10.1111/gtc.12353;
RA   Ijuin T., Hatano N., Takenawa T.;
RT   "Glucose-regulated protein 78 (GRP78) binds directly to PIP3 phosphatase
RT   SKIP and determines its localization.";
RL   Genes Cells 21:457-465(2016).
RN   [47]
RP   REVIEW.
RX   PubMed=28286085; DOI=10.1016/j.gene.2017.03.005;
RA   Wang J., Lee J., Liem D., Ping P.;
RT   "HSPA5 Gene encoding Hsp70 chaperone BiP in the endoplasmic reticulum.";
RL   Gene 618:14-23(2017).
RN   [48]
RP   SUMOYLATION [LARGE SCALE ANALYSIS] AT LYS-352, AND IDENTIFICATION BY MASS
RP   SPECTROMETRY [LARGE SCALE ANALYSIS].
RX   PubMed=28112733; DOI=10.1038/nsmb.3366;
RA   Hendriks I.A., Lyon D., Young C., Jensen L.J., Vertegaal A.C.,
RA   Nielsen M.L.;
RT   "Site-specific mapping of the human SUMO proteome reveals co-modification
RT   with phosphorylation.";
RL   Nat. Struct. Mol. Biol. 24:325-336(2017).
RN   [49]
RP   FUNCTION, INTERACTION WITH LOXL2, AND IDENTIFICATION BY MASS SPECTROMETRY.
RX   PubMed=28332555; DOI=10.1038/srep44988;
RA   Cuevas E.P., Eraso P., Mazon M.J., Santos V., Moreno-Bueno G., Cano A.,
RA   Portillo F.;
RT   "LOXL2 drives epithelial-mesenchymal transition via activation of IRE1-XBP1
RT   signalling pathway.";
RL   Sci. Rep. 7:44988-44988(2017).
RN   [50]
RP   FUNCTION (MICROBIAL INFECTION), INTERACTION WITH JAPANESE ENCEPHALITIS
RP   VIRUS ENVELOPE PROTEIN E (MICROBIAL INFECTION), AND SUBCELLULAR LOCATION.
RX   PubMed=28053106; DOI=10.1128/jvi.02274-16;
RA   Nain M., Mukherjee S., Karmakar S.P., Paton A.W., Paton J.C., Abdin M.Z.,
RA   Basu A., Kalia M., Vrati S.;
RT   "GRP78 Is an Important Host Factor for Japanese Encephalitis Virus Entry
RT   and Replication in Mammalian Cells.";
RL   J. Virol. 91:0-0(2017).
RN   [51]
RP   FUNCTION.
RX   PubMed=29719251; DOI=10.1016/j.celrep.2018.03.122;
RA   Hassdenteufel S., Johnson N., Paton A.W., Paton J.C., High S.,
RA   Zimmermann R.;
RT   "Chaperone-Mediated Sec61 Channel Gating during ER Import of Small
RT   Precursor Proteins Overcomes Sec61 Inhibitor-Reinforced Energy Barrier.";
RL   Cell Rep. 23:1373-1386(2018).
RN   [52]
RP   SUBCELLULAR LOCATION.
RX   PubMed=29497057; DOI=10.1038/s41467-018-03355-0;
RA   Bai M., Vozdek R., Hnizda A., Jiang C., Wang B., Kuchar L., Li T.,
RA   Zhang Y., Wood C., Feng L., Dang Y., Ma D.K.;
RT   "Conserved roles of C. elegans and human MANFs in sulfatide binding and
RT   cytoprotection.";
RL   Nat. Commun. 9:897-897(2018).
RN   [53]
RP   FUNCTION (MICROBIAL INFECTION), INTERACTION WITH ZIKA VIRUS ENVELOPE
RP   PROTEIN E (MICROBIAL INFECTION), AND SUBCELLULAR LOCATION.
RX   PubMed=33432092; DOI=10.1038/s41598-020-79803-z;
RA   Khongwichit S., Sornjai W., Jitobaom K., Greenwood M., Greenwood M.P.,
RA   Hitakarun A., Wikan N., Murphy D., Smith D.R.;
RT   "A functional interaction between GRP78 and Zika virus E protein.";
RL   Sci. Rep. 11:393-393(2021).
RN   [54]
RP   X-RAY CRYSTALLOGRAPHY (2.40 ANGSTROMS) OF 26-410.
RX   PubMed=20072699; DOI=10.1371/journal.pone.0008625;
RA   Wisniewska M., Karlberg T., Lehtio L., Johansson I., Kotenyova T.,
RA   Moche M., Schuler H.;
RT   "Crystal structures of the ATPase domains of four human Hsp70 isoforms:
RT   HSPA1L/Hsp70-hom, HSPA2/Hsp70-2, HSPA6/Hsp70B', and HSPA5/BiP/GRP78.";
RL   PLoS ONE 5:E8625-E8625(2010).
RN   [55]
RP   X-RAY CRYSTALLOGRAPHY (1.95 ANGSTROMS) OF 26-407 IN COMPLEXES WITH ATP
RP   ANALOG.
RX   PubMed=21526763; DOI=10.1021/jm101625x;
RA   Macias A.T., Williamson D.S., Allen N., Borgognoni J., Clay A., Daniels Z.,
RA   Dokurno P., Drysdale M.J., Francis G.L., Graham C.J., Howes R.,
RA   Matassova N., Murray J.B., Parsons R., Shaw T., Surgenor A.E., Terry L.,
RA   Wang Y., Wood M., Massey A.J.;
RT   "Adenosine-derived inhibitors of 78 kDa glucose regulated protein (Grp78)
RT   ATPase: insights into isoform selectivity.";
RL   J. Med. Chem. 54:4034-4041(2011).
RN   [56] {ECO:0007744|PDB:5E84, ECO:0007744|PDB:5E85, ECO:0007744|PDB:5E86}
RP   X-RAY CRYSTALLOGRAPHY (2.57 ANGSTROMS) OF 418-637 IN COMPLEX WITH ATP,
RP   CATALYTIC ACTIVITY, ACTIVITY REGULATION, AND MUTAGENESIS OF THR-229.
RX   PubMed=26655470; DOI=10.1016/j.str.2015.10.012;
RA   Yang J., Nune M., Zong Y., Zhou L., Liu Q.;
RT   "Close and allosteric opening of the polypeptide-binding site in a human
RT   Hsp70 chaperone BiP.";
RL   Structure 23:2191-2203(2015).
CC   -!- FUNCTION: Endoplasmic reticulum chaperone that plays a key role in
CC       protein folding and quality control in the endoplasmic reticulum lumen
CC       (PubMed:2294010, PubMed:23769672, PubMed:23990668, PubMed:28332555).
CC       Involved in the correct folding of proteins and degradation of
CC       misfolded proteins via its interaction with DNAJC10/ERdj5, probably to
CC       facilitate the release of DNAJC10/ERdj5 from its substrate (By
CC       similarity). Acts as a key repressor of the ERN1/IRE1-mediated unfolded
CC       protein response (UPR) (PubMed:1550958, PubMed:19538957). In the
CC       unstressed endoplasmic reticulum, recruited by DNAJB9/ERdj4 to the
CC       luminal region of ERN1/IRE1, leading to disrupt the dimerization of
CC       ERN1/IRE1, thereby inactivating ERN1/IRE1 (By similarity). Accumulation
CC       of misfolded protein in the endoplasmic reticulum causes release of
CC       HSPA5/BiP from ERN1/IRE1, allowing homodimerization and subsequent
CC       activation of ERN1/IRE1 (By similarity). Plays an auxiliary role in
CC       post-translational transport of small presecretory proteins across
CC       endoplasmic reticulum (ER). May function as an allosteric modulator for
CC       SEC61 channel-forming translocon complex, likely cooperating with SEC62
CC       to enable the productive insertion of these precursors into SEC61
CC       channel. Appears to specifically regulate translocation of precursors
CC       having inhibitory residues in their mature region that weaken channel
CC       gating. May also play a role in apoptosis and cell proliferation
CC       (PubMed:26045166). {ECO:0000250|UniProtKB:G3I8R9,
CC       ECO:0000250|UniProtKB:P20029, ECO:0000269|PubMed:1550958,
CC       ECO:0000269|PubMed:19538957, ECO:0000269|PubMed:2294010,
CC       ECO:0000269|PubMed:23769672, ECO:0000269|PubMed:23990668,
CC       ECO:0000269|PubMed:26045166, ECO:0000269|PubMed:28332555,
CC       ECO:0000269|PubMed:29719251}.
CC   -!- FUNCTION: (Microbial infection) Plays an important role in viral
CC       binding to the host cell membrane and entry for several flaviruses such
CC       as Dengue virus, Zika virus and Japanese encephalitis virus
CC       (PubMed:33432092, PubMed:15098107, PubMed:28053106). Acts as a
CC       component of the cellular receptor for Dengue virus serotype 2/DENV-2
CC       on human liver cells (PubMed:15098107). {ECO:0000269|PubMed:15098107,
CC       ECO:0000269|PubMed:28053106, ECO:0000269|PubMed:33432092}.
CC   -!- CATALYTIC ACTIVITY:
CC       Reaction=ATP + H2O = ADP + H(+) + phosphate; Xref=Rhea:RHEA:13065,
CC         ChEBI:CHEBI:15377, ChEBI:CHEBI:15378, ChEBI:CHEBI:30616,
CC         ChEBI:CHEBI:43474, ChEBI:CHEBI:456216; EC=3.6.4.10;
CC         Evidence={ECO:0000269|PubMed:26655470};
CC   -!- ACTIVITY REGULATION: The chaperone activity is regulated by ATP-induced
CC       allosteric coupling of the nucleotide-binding (NBD) and substrate-
CC       binding (SBD) domains. In the ADP-bound and nucleotide-free (apo)
CC       states, the two domains have little interaction (PubMed:26655470). In
CC       contrast, in the ATP-bound state the two domains are tightly coupled,
CC       which results in drastically accelerated kinetics in both binding and
CC       release of polypeptide substrates (PubMed:26655470). J domain-
CC       containing co-chaperones (DNAJB9/ERdj4 or DNAJC10/ERdj5) stimulate the
CC       ATPase activity and are required for efficient substrate recognition by
CC       HSPA5/BiP (By similarity). Homooligomerization inactivates
CC       participating HSPA5/BiP protomers and probably act as reservoirs to
CC       store HSPA5/BiP molecules when they are not needed by the cell (By
CC       similarity). {ECO:0000250|UniProtKB:G3I8R9,
CC       ECO:0000269|PubMed:26655470, ECO:0000303|PubMed:28286085}.
CC   -!- SUBUNIT: Monomer and homooligomer; homooligomerization via the
CC       interdomain linker inactivates the chaperone activity and acts as a
CC       storage of HSPA5/BiP molecules (By similarity). Interacts with DNAJC1
CC       (via J domain) (By similarity). Component of an EIF2 complex at least
CC       composed of CELF1/CUGBP1, CALR, CALR3, EIF2S1, EIF2S2, HSP90B1 and
CC       HSPA5 (By similarity). Part of a large chaperone multiprotein complex
CC       comprising DNAJB11, HSP90B1, HSPA5, HYOU, PDIA2, PDIA4, PDIA6, PPIB,
CC       SDF2L1, UGGT1 and very small amounts of ERP29, but not, or at very low
CC       levels, CALR nor CANX (By similarity). Interacts with TMEM132A and
CC       TRIM21 (PubMed:12699405). May form a complex with ERLEC1, OS9, SEL1L
CC       and SYVN1 (PubMed:18264092,PubMed:18502753). Interacts with DNAJC10
CC       (PubMed:12411443, PubMed:23769672). Interacts with DNAJB9/ERdj4;
CC       leading to recruit HSPA5/BiP to ERN1/IRE1 (By similarity). Interacts
CC       with ERN1/IRE1; interaction takes place following interaction with
CC       DNAJB9/ERdj4 and leads to inactivate ERN1/IRE1 (By similarity).
CC       Interacts with MX1 (By similarity). Interacts with METTL23
CC       (PubMed:23349634). Interacts with CEMIP; the interaction induces
CC       calcium leakage from the endoplasmic reticulum and cell migration
CC       (PubMed:23990668). Interacts with PCSK4 form; the interaction takes
CC       place in the endoplasmic reticulum (PubMed:21080038). Interacts with
CC       CIPC (PubMed:26657846). Interacts with CCDC88B (via C-terminus); the
CC       interaction opposes ERN1-mediated JNK activation, protecting against
CC       apoptosis (PubMed:21289099). Interacts with INPP5K; necessary for
CC       INPP5K localization at the endoplasmic reticulum (PubMed:26940976).
CC       Interacts with MANF; the interaction is direct (PubMed:22637475).
CC       Interacts with LOXL2; leading to activate the ERN1/IRE1-XBP1 pathway of
CC       the unfolded protein response (PubMed:28332555). Interacts with CLU
CC       under stressed condition; interaction increases CLU protein stability;
CC       facilitates its retrotranslocation and redistribution to the
CC       mitochondria; cooperatively suppress stress-induced apoptosis by
CC       stabilizing mitochondrial membrane integrity (PubMed:22689054).
CC       Interacts with CCDC47 (By similarity). Interacts with CLN3 (Probable).
CC       Interacts with KIAA1324; may regulate the function of HSPA5 in
CC       apoptosis and cell proliferation (PubMed:26045166). Interacts with
CC       CASP7 (PubMed:26045166). {ECO:0000250|UniProtKB:G3I8R9,
CC       ECO:0000250|UniProtKB:P20029, ECO:0000269|PubMed:12411443,
CC       ECO:0000269|PubMed:12699405, ECO:0000269|PubMed:18264092,
CC       ECO:0000269|PubMed:18502753, ECO:0000269|PubMed:21080038,
CC       ECO:0000269|PubMed:21289099, ECO:0000269|PubMed:22637475,
CC       ECO:0000269|PubMed:22689054, ECO:0000269|PubMed:23349634,
CC       ECO:0000269|PubMed:23769672, ECO:0000269|PubMed:23990668,
CC       ECO:0000269|PubMed:26045166, ECO:0000269|PubMed:26657846,
CC       ECO:0000269|PubMed:26940976, ECO:0000269|PubMed:28332555,
CC       ECO:0000305|PubMed:18621045}.
CC   -!- SUBUNIT: (Microbial infection) Interacts with Japanese encephalitis
CC       virus envelope protein E. {ECO:0000269|PubMed:28053106}.
CC   -!- SUBUNIT: (Microbial infection) Interacts with Zika virus envelope
CC       protein E. {ECO:0000269|PubMed:33432092}.
CC   -!- INTERACTION:
CC       P11021; P05067: APP; NbExp=3; IntAct=EBI-354921, EBI-77613;
CC       P11021; P18850: ATF6; NbExp=2; IntAct=EBI-354921, EBI-852157;
CC       P11021; Q9ULD4-2: BRPF3; NbExp=3; IntAct=EBI-354921, EBI-23662416;
CC       P11021; Q6E0U4: DMKN; NbExp=3; IntAct=EBI-354921, EBI-7943171;
CC       P11021; Q9UBS4: DNAJB11; NbExp=4; IntAct=EBI-354921, EBI-713113;
CC       P11021; Q9NZJ5: EIF2AK3; NbExp=4; IntAct=EBI-354921, EBI-766076;
CC       P11021; O75460: ERN1; NbExp=3; IntAct=EBI-354921, EBI-371750;
CC       P11021; O75460-1: ERN1; NbExp=4; IntAct=EBI-354921, EBI-15600828;
CC       P11021; P62993: GRB2; NbExp=4; IntAct=EBI-354921, EBI-401755;
CC       P11021; Q15486: GUSBP1; NbExp=3; IntAct=EBI-354921, EBI-712457;
CC       P11021; P14625: HSP90B1; NbExp=2; IntAct=EBI-354921, EBI-359129;
CC       P11021; Q9Y4L1: HYOU1; NbExp=2; IntAct=EBI-354921, EBI-1054186;
CC       P11021; Q96IZ0: PAWR; NbExp=8; IntAct=EBI-354921, EBI-595869;
CC       P11021; Q15084: PDIA6; NbExp=2; IntAct=EBI-354921, EBI-1043087;
CC       P11021; P04049: RAF1; NbExp=5; IntAct=EBI-354921, EBI-365996;
CC       P11021; P61619: SEC61A1; NbExp=3; IntAct=EBI-354921, EBI-358919;
CC       P11021; Q9H173: SIL1; NbExp=9; IntAct=EBI-354921, EBI-2840325;
CC       P11021; Q9UHI5: SLC7A8; NbExp=3; IntAct=EBI-354921, EBI-13292283;
CC       P11021; Q13573: SNW1; NbExp=5; IntAct=EBI-354921, EBI-632715;
CC       P11021; Q6PF05: TTC23L; NbExp=3; IntAct=EBI-354921, EBI-8656864;
CC       P11021; Q13404: UBE2V1; NbExp=3; IntAct=EBI-354921, EBI-1050671;
CC       P11021; Q6T424; NbExp=3; IntAct=EBI-354921, EBI-7888150;
CC       P11021; Q9QXT0: Cnpy2; Xeno; NbExp=2; IntAct=EBI-354921, EBI-8321111;
CC       P11021; Q91YW3: Dnajc3; Xeno; NbExp=2; IntAct=EBI-354921, EBI-8381770;
CC       P11021; Q9Z2B5: Eif2ak3; Xeno; NbExp=2; IntAct=EBI-354921, EBI-1226344;
CC       P11021; Q62627: Pawr; Xeno; NbExp=4; IntAct=EBI-354921, EBI-1187240;
CC       P11021; K0BRG7: S; Xeno; NbExp=9; IntAct=EBI-354921, EBI-16040613;
CC   -!- SUBCELLULAR LOCATION: Endoplasmic reticulum lumen
CC       {ECO:0000269|PubMed:21080038, ECO:0000269|PubMed:21289099,
CC       ECO:0000269|PubMed:23990668, ECO:0000269|PubMed:29497057}. Melanosome
CC       {ECO:0000269|PubMed:12643545}. Cytoplasm
CC       {ECO:0000250|UniProtKB:P20029}. Cell surface
CC       {ECO:0000269|PubMed:15098107, ECO:0000269|PubMed:28053106,
CC       ECO:0000269|PubMed:33432092}. Note=Identified by mass spectrometry in
CC       melanosome fractions from stage I to stage IV.
CC       {ECO:0000269|PubMed:12643545}.
CC   -!- INDUCTION: By endoplasmic reticulum stress.
CC       {ECO:0000269|PubMed:21289099}.
CC   -!- DOMAIN: The interdomain linker regulates the chaperone activity by
CC       mediating the formation of homooligomers. Homooligomers are formed by
CC       engagement of the interdomain linker of one HSPA5/BiP molecule as a
CC       typical substrate of an adjacent HSPA5/BiP molecule. HSPA5/BiP
CC       oligomerization inactivates participating HSPA5/BiP protomers.
CC       HSPA5/BiP oligomers probably act as reservoirs to store HSPA5/BiP
CC       molecules when they are not needed by the cell. When the levels of
CC       unfolded proteins rise, cells can rapidly break up these oligomers to
CC       make active monomers. {ECO:0000250|UniProtKB:G3I8R9}.
CC   -!- PTM: AMPylated by FICD (PubMed:25601083). In unstressed cells,
CC       AMPylation at Thr-518 by FICD inactivates the chaperome activity:
CC       AMPylated form is locked in a relatively inert state and only weakly
CC       stimulated by J domain-containing proteins (By similarity). In response
CC       to endoplasmic reticulum stress, de-AMPylation by the same protein,
CC       FICD, restores the chaperone activity (By similarity).
CC       {ECO:0000250|UniProtKB:G3I8R9, ECO:0000269|PubMed:25601083}.
CC   -!- DISEASE: Note=Autoantigen in rheumatoid arthritis.
CC       {ECO:0000269|PubMed:11160188}.
CC   -!- SIMILARITY: Belongs to the heat shock protein 70 family. {ECO:0000305}.
CC   -!- CAUTION: AMPylation was initially reported to take place at Ser-365 and
CC       Thr-366 in vitro, and promote activation of HSPA5/BiP
CC       (PubMed:25601083). However, it was later shown that AMPylation takes
CC       place at Thr-518 and leads to inactivation of HSPA5/BiP.
CC       {ECO:0000250|UniProtKB:G3I8R9, ECO:0000269|PubMed:25601083}.
CC   -!- WEB RESOURCE: Name=NIEHS-SNPs;
CC       URL="http://egp.gs.washington.edu/data/hspa5/";
CC   -!- WEB RESOURCE: Name=Atlas of Genetics and Cytogenetics in Oncology and
CC       Haematology;
CC       URL="http://atlasgeneticsoncology.org/Genes/HSPA5ID40876ch9q33.html";
CC   ---------------------------------------------------------------------------
CC   Copyrighted by the UniProt Consortium, see https://www.uniprot.org/terms
CC   Distributed under the Creative Commons Attribution (CC BY 4.0) License
CC   ---------------------------------------------------------------------------
DR   EMBL; M19645; AAA52614.1; -; Genomic_DNA.
DR   EMBL; X87949; CAA61201.1; -; mRNA.
DR   EMBL; AJ271729; CAB71335.1; -; mRNA.
DR   EMBL; AF216292; AAF42836.1; -; mRNA.
DR   EMBL; DQ385847; ABD04090.1; -; Genomic_DNA.
DR   EMBL; AL354710; -; NOT_ANNOTATED_CDS; Genomic_DNA.
DR   EMBL; CH471090; EAW87620.1; -; Genomic_DNA.
DR   EMBL; BC020235; AAH20235.1; -; mRNA.
DR   EMBL; X59969; CAA42595.1; -; Genomic_DNA.
DR   EMBL; AF188611; AAF13605.1; ALT_SEQ; mRNA.
DR   CCDS; CCDS6863.1; -.
DR   PIR; A29821; A29821.
DR   RefSeq; NP_005338.1; NM_005347.4.
DR   PDB; 3IUC; X-ray; 2.40 A; A/C=26-410.
DR   PDB; 3LDL; X-ray; 2.30 A; A/B=26-407.
DR   PDB; 3LDN; X-ray; 2.20 A; A/B=26-407.
DR   PDB; 3LDO; X-ray; 1.95 A; A/B=26-407.
DR   PDB; 3LDP; X-ray; 2.20 A; A/B=26-407.
DR   PDB; 5E84; X-ray; 2.99 A; A/B/C/D/E/F=25-633.
DR   PDB; 5E85; X-ray; 2.57 A; A=418-637.
DR   PDB; 5E86; X-ray; 2.68 A; A=418-637.
DR   PDB; 5EVZ; X-ray; 1.85 A; A/B=26-407.
DR   PDB; 5EX5; X-ray; 1.90 A; A/B=26-407.
DR   PDB; 5EXW; X-ray; 1.90 A; A/B=26-407.
DR   PDB; 5EY4; X-ray; 1.86 A; A/B=26-407.
DR   PDB; 5F0X; X-ray; 1.60 A; A/B=26-407.
DR   PDB; 5F1X; X-ray; 1.90 A; A/B=26-407.
DR   PDB; 5F2R; X-ray; 2.15 A; A/B=26-407.
DR   PDB; 6ASY; X-ray; 1.85 A; A/B=25-633.
DR   PDB; 6CZ1; X-ray; 1.68 A; A/B=26-407.
DR   PDB; 6DFM; X-ray; 2.14 A; A/B=26-407.
DR   PDB; 6DFO; X-ray; 2.54 A; A/B=26-407.
DR   PDB; 6DO2; X-ray; 1.70 A; A/B=26-407.
DR   PDB; 6DWS; X-ray; 1.90 A; A/B=26-407.
DR   PDBsum; 3IUC; -.
DR   PDBsum; 3LDL; -.
DR   PDBsum; 3LDN; -.
DR   PDBsum; 3LDO; -.
DR   PDBsum; 3LDP; -.
DR   PDBsum; 5E84; -.
DR   PDBsum; 5E85; -.
DR   PDBsum; 5E86; -.
DR   PDBsum; 5EVZ; -.
DR   PDBsum; 5EX5; -.
DR   PDBsum; 5EXW; -.
DR   PDBsum; 5EY4; -.
DR   PDBsum; 5F0X; -.
DR   PDBsum; 5F1X; -.
DR   PDBsum; 5F2R; -.
DR   PDBsum; 6ASY; -.
DR   PDBsum; 6CZ1; -.
DR   PDBsum; 6DFM; -.
DR   PDBsum; 6DFO; -.
DR   PDBsum; 6DO2; -.
DR   PDBsum; 6DWS; -.
DR   SMR; P11021; -.
DR   BioGRID; 109541; 809.
DR   CORUM; P11021; -.
DR   DIP; DIP-33189N; -.
DR   ELM; P11021; -.
DR   IntAct; P11021; 406.
DR   MINT; P11021; -.
DR   STRING; 9606.ENSP00000324173; -.
DR   BindingDB; P11021; -.
DR   ChEMBL; CHEMBL1781865; -.
DR   DrugBank; DB00945; Acetylsalicylic acid.
DR   DrugBank; DB00025; Antihemophilic factor, human recombinant.
DR   DrugBank; DB09130; Copper.
DR   DrugBank; DB13998; Lonoctocog alfa.
DR   DrugBank; DB13999; Moroctocog alfa.
DR   TCDB; 1.A.33.1.5; the cation channel-forming heat shock protein-70 (hsp70) family.
DR   GlyGen; P11021; 1 site.
DR   iPTMnet; P11021; -.
DR   MetOSite; P11021; -.
DR   PhosphoSitePlus; P11021; -.
DR   SwissPalm; P11021; -.
DR   BioMuta; HSPA5; -.
DR   DMDM; 14916999; -.
DR   DOSAC-COBS-2DPAGE; P11021; -.
DR   OGP; P11021; -.
DR   REPRODUCTION-2DPAGE; P11021; -.
DR   SWISS-2DPAGE; P11021; -.
DR   UCD-2DPAGE; P11021; -.
DR   CPTAC; CPTAC-524; -.
DR   CPTAC; CPTAC-525; -.
DR   CPTAC; non-CPTAC-2616; -.
DR   EPD; P11021; -.
DR   jPOST; P11021; -.
DR   MassIVE; P11021; -.
DR   PaxDb; P11021; -.
DR   PeptideAtlas; P11021; -.
DR   PRIDE; P11021; -.
DR   ProteomicsDB; 52688; -.
DR   TopDownProteomics; P11021; -.
DR   ABCD; P11021; 16 sequenced antibodies.
DR   Antibodypedia; 3926; 1757 antibodies.
DR   DNASU; 3309; -.
DR   Ensembl; ENST00000324460; ENSP00000324173; ENSG00000044574.
DR   GeneID; 3309; -.
DR   KEGG; hsa:3309; -.
DR   CTD; 3309; -.
DR   DisGeNET; 3309; -.
DR   GeneCards; HSPA5; -.
DR   HGNC; HGNC:5238; HSPA5.
DR   HPA; ENSG00000044574; Low tissue specificity.
DR   MIM; 138120; gene.
DR   neXtProt; NX_P11021; -.
DR   OpenTargets; ENSG00000044574; -.
DR   PharmGKB; PA29504; -.
DR   VEuPathDB; HostDB:ENSG00000044574.7; -.
DR   eggNOG; KOG0100; Eukaryota.
DR   GeneTree; ENSGT00940000154787; -.
DR   HOGENOM; CLU_005965_3_0_1; -.
DR   InParanoid; P11021; -.
DR   OMA; CVGVMQK; -.
DR   OrthoDB; 288077at2759; -.
DR   PhylomeDB; P11021; -.
DR   TreeFam; TF105044; -.
DR   PathwayCommons; P11021; -.
DR   Reactome; R-HSA-114608; Platelet degranulation.
DR   Reactome; R-HSA-3371453; Regulation of HSF1-mediated heat shock response.
DR   Reactome; R-HSA-381033; ATF6 (ATF6-alpha) activates chaperones.
DR   Reactome; R-HSA-381042; PERK regulates gene expression.
DR   Reactome; R-HSA-381070; IRE1alpha activates chaperones.
DR   Reactome; R-HSA-381183; ATF6 (ATF6-alpha) activates chaperone genes.
DR   Reactome; R-HSA-983170; Antigen Presentation: Folding, assembly and peptide loading of class I MHC.
DR   SIGNOR; P11021; -.
DR   BioGRID-ORCS; 3309; 648 hits in 990 CRISPR screens.
DR   ChiTaRS; HSPA5; human.
DR   EvolutionaryTrace; P11021; -.
DR   GeneWiki; Binding_immunoglobulin_protein; -.
DR   GenomeRNAi; 3309; -.
DR   Pharos; P11021; Tchem.
DR   PRO; PR:P11021; -.
DR   Proteomes; UP000005640; Chromosome 9.
DR   RNAct; P11021; protein.
DR   Bgee; ENSG00000044574; Expressed in vena cava and 256 other tissues.
DR   ExpressionAtlas; P11021; baseline and differential.
DR   Genevisible; P11021; HS.
DR   GO; GO:0009986; C:cell surface; IEA:Ensembl.
DR   GO; GO:0005737; C:cytoplasm; IDA:UniProtKB.
DR   GO; GO:0005829; C:cytosol; IDA:UniProtKB.
DR   GO; GO:0005783; C:endoplasmic reticulum; IDA:MGI.
DR   GO; GO:0034663; C:endoplasmic reticulum chaperone complex; IDA:UniProtKB.
DR   GO; GO:0005788; C:endoplasmic reticulum lumen; IDA:UniProtKB.
DR   GO; GO:0005789; C:endoplasmic reticulum membrane; TAS:Reactome.
DR   GO; GO:0005793; C:endoplasmic reticulum-Golgi intermediate compartment; IDA:UniProtKB.
DR   GO; GO:0070062; C:extracellular exosome; HDA:UniProtKB.
DR   GO; GO:0005925; C:focal adhesion; HDA:UniProtKB.
DR   GO; GO:0030176; C:integral component of endoplasmic reticulum membrane; IDA:BHF-UCL.
DR   GO; GO:0043231; C:intracellular membrane-bounded organelle; IDA:UniProtKB.
DR   GO; GO:0042470; C:melanosome; IEA:UniProtKB-SubCell.
DR   GO; GO:0016020; C:membrane; HDA:UniProtKB.
DR   GO; GO:0030496; C:midbody; IDA:UniProtKB.
DR   GO; GO:0005739; C:mitochondrion; IDA:UniProtKB.
DR   GO; GO:0005634; C:nucleus; IMP:UniProtKB.
DR   GO; GO:0005886; C:plasma membrane; IEA:Ensembl.
DR   GO; GO:0032991; C:protein-containing complex; IDA:UniProtKB.
DR   GO; GO:0005790; C:smooth endoplasmic reticulum; IEA:Ensembl.
DR   GO; GO:0005524; F:ATP binding; IDA:UniProtKB.
DR   GO; GO:0016887; F:ATPase activity; IDA:UniProtKB.
DR   GO; GO:0045296; F:cadherin binding; HDA:BHF-UCL.
DR   GO; GO:0005509; F:calcium ion binding; TAS:UniProtKB.
DR   GO; GO:0051087; F:chaperone binding; TAS:BHF-UCL.
DR   GO; GO:0019899; F:enzyme binding; IPI:BHF-UCL.
DR   GO; GO:0031072; F:heat shock protein binding; IBA:GO_Central.
DR   GO; GO:0051787; F:misfolded protein binding; IDA:UniProtKB.
DR   GO; GO:0019904; F:protein domain specific binding; IPI:UniProtKB.
DR   GO; GO:0044183; F:protein folding chaperone; IBA:GO_Central.
DR   GO; GO:0043022; F:ribosome binding; IEA:Ensembl.
DR   GO; GO:0031625; F:ubiquitin protein ligase binding; IPI:UniProtKB.
DR   GO; GO:0051082; F:unfolded protein binding; IBA:GO_Central.
DR   GO; GO:0036500; P:ATF6-mediated unfolded protein response; TAS:Reactome.
DR   GO; GO:0071236; P:cellular response to antibiotic; IEA:Ensembl.
DR   GO; GO:0071277; P:cellular response to calcium ion; IEA:Ensembl.
DR   GO; GO:0071320; P:cellular response to cAMP; IEA:Ensembl.
DR   GO; GO:0035690; P:cellular response to drug; IEA:Ensembl.
DR   GO; GO:0071480; P:cellular response to gamma radiation; IEA:Ensembl.
DR   GO; GO:0042149; P:cellular response to glucose starvation; IDA:UniProtKB.
DR   GO; GO:0071353; P:cellular response to interleukin-4; IEA:Ensembl.
DR   GO; GO:0071287; P:cellular response to manganese ion; IEA:Ensembl.
DR   GO; GO:1990090; P:cellular response to nerve growth factor stimulus; IEA:Ensembl.
DR   GO; GO:0034620; P:cellular response to unfolded protein; IBA:GO_Central.
DR   GO; GO:0021680; P:cerebellar Purkinje cell layer development; IEA:Ensembl.
DR   GO; GO:0021589; P:cerebellum structural organization; IEA:Ensembl.
DR   GO; GO:0051085; P:chaperone cofactor-dependent protein refolding; IBA:GO_Central.
DR   GO; GO:0030968; P:endoplasmic reticulum unfolded protein response; IBA:GO_Central.
DR   GO; GO:0006983; P:ER overload response; IEA:Ensembl.
DR   GO; GO:0036498; P:IRE1-mediated unfolded protein response; TAS:Reactome.
DR   GO; GO:0001554; P:luteolysis; IEA:Ensembl.
DR   GO; GO:0035437; P:maintenance of protein localization in endoplasmic reticulum; IMP:UniProtKB.
DR   GO; GO:0043066; P:negative regulation of apoptotic process; IMP:UniProtKB.
DR   GO; GO:1903895; P:negative regulation of IRE1-mediated unfolded protein response; ISS:UniProtKB.
DR   GO; GO:0031333; P:negative regulation of protein-containing complex assembly; ISS:GO_Central.
DR   GO; GO:0030512; P:negative regulation of transforming growth factor beta receptor signaling pathway; IEA:Ensembl.
DR   GO; GO:0051402; P:neuron apoptotic process; IEA:Ensembl.
DR   GO; GO:0030182; P:neuron differentiation; IEA:Ensembl.
DR   GO; GO:0036499; P:PERK-mediated unfolded protein response; TAS:Reactome.
DR   GO; GO:0030335; P:positive regulation of cell migration; IMP:UniProtKB.
DR   GO; GO:0010976; P:positive regulation of neuron projection development; IEA:Ensembl.
DR   GO; GO:0031398; P:positive regulation of protein ubiquitination; IEA:Ensembl.
DR   GO; GO:1990440; P:positive regulation of transcription from RNA polymerase II promoter in response to endoplasmic reticulum stress; TAS:ParkinsonsUK-UCL.
DR   GO; GO:0031204; P:posttranslational protein targeting to membrane, translocation; IMP:UniProtKB.
DR   GO; GO:0034975; P:protein folding in endoplasmic reticulum; TAS:ParkinsonsUK-UCL.
DR   GO; GO:0042026; P:protein refolding; IBA:GO_Central.
DR   GO; GO:1903891; P:regulation of ATF6-mediated unfolded protein response; TAS:ParkinsonsUK-UCL.
DR   GO; GO:1903894; P:regulation of IRE1-mediated unfolded protein response; TAS:ParkinsonsUK-UCL.
DR   GO; GO:1903897; P:regulation of PERK-mediated unfolded protein response; TAS:ParkinsonsUK-UCL.
DR   GO; GO:0060904; P:regulation of protein folding in endoplasmic reticulum; TAS:BHF-UCL.
DR   GO; GO:0042220; P:response to cocaine; IEA:Ensembl.
DR   GO; GO:1904313; P:response to methamphetamine hydrochloride; IEA:Ensembl.
DR   GO; GO:0097501; P:stress response to metal ion; IEA:Ensembl.
DR   GO; GO:0021762; P:substantia nigra development; HEP:UniProtKB.
DR   GO; GO:1901998; P:toxin transport; IEA:Ensembl.
DR   GO; GO:0030433; P:ubiquitin-dependent ERAD pathway; IBA:GO_Central.
DR   CDD; cd10241; HSPA5-like_NBD; 1.
DR   DisProt; DP01938; -.
DR   Gene3D; 1.20.1270.10; -; 1.
DR   Gene3D; 2.60.34.10; -; 1.
DR   InterPro; IPR043129; ATPase_NBD.
DR   InterPro; IPR042050; BIP_NBD.
DR   InterPro; IPR018181; Heat_shock_70_CS.
DR   InterPro; IPR029048; HSP70_C_sf.
DR   InterPro; IPR029047; HSP70_peptide-bd_sf.
DR   InterPro; IPR013126; Hsp_70_fam.
DR   PANTHER; PTHR19375; PTHR19375; 1.
DR   Pfam; PF00012; HSP70; 1.
DR   SUPFAM; SSF100920; SSF100920; 1.
DR   SUPFAM; SSF100934; SSF100934; 1.
DR   SUPFAM; SSF53067; SSF53067; 2.
DR   PROSITE; PS00014; ER_TARGET; 1.
DR   PROSITE; PS00297; HSP70_1; 1.
DR   PROSITE; PS00329; HSP70_2; 1.
DR   PROSITE; PS01036; HSP70_3; 1.
PE   1: Evidence at protein level;
KW   3D-structure; Acetylation; ATP-binding; Chaperone; Cytoplasm;
KW   Direct protein sequencing; Endoplasmic reticulum; Host-virus interaction;
KW   Hydrolase; Isopeptide bond; Methylation; Nitration; Nucleotide-binding;
KW   Phosphoprotein; Reference proteome; Signal; Ubl conjugation.
FT   SIGNAL          1..18
FT                   /evidence="ECO:0000269|PubMed:2294010,
FT                   ECO:0000269|PubMed:9150948"
FT   CHAIN           19..654
FT                   /note="Endoplasmic reticulum chaperone BiP"
FT                   /id="PRO_0000013566"
FT   NP_BIND         36..39
FT                   /note="ATP"
FT                   /evidence="ECO:0000269|PubMed:21526763"
FT   NP_BIND         227..229
FT                   /note="ATP"
FT                   /evidence="ECO:0000269|PubMed:21526763"
FT   NP_BIND         293..300
FT                   /note="ATP"
FT                   /evidence="ECO:0000269|PubMed:21526763"
FT   NP_BIND         364..367
FT                   /note="ATP"
FT                   /evidence="ECO:0000269|PubMed:21526763"
FT   REGION          1..80
FT                   /note="Required for interaction with KIAA1324"
FT                   /evidence="ECO:0000269|PubMed:26045166"
FT   REGION          125..280
FT                   /note="Nucleotide-binding (NBD)"
FT                   /evidence="ECO:0000303|PubMed:28286085"
FT   REGION          409..419
FT                   /note="Interdomain linker"
FT                   /evidence="ECO:0000250|UniProtKB:G3I8R9"
FT   REGION          420..500
FT                   /note="Substrate-binding (SBD)"
FT                   /evidence="ECO:0000303|PubMed:28286085"
FT   REGION          633..654
FT                   /note="Disordered"
FT                   /evidence="ECO:0000256|SAM:MobiDB-lite"
FT   MOTIF           651..654
FT                   /note="Prevents secretion from ER"
FT                   /evidence="ECO:0000255"
FT   BINDING         96
FT                   /note="ATP"
FT                   /evidence="ECO:0000269|PubMed:21526763"
FT   MOD_RES         86
FT                   /note="Phosphoserine"
FT                   /evidence="ECO:0000250|UniProtKB:P06761"
FT   MOD_RES         125
FT                   /note="N6-acetyllysine"
FT                   /evidence="ECO:0000250|UniProtKB:P20029"
FT   MOD_RES         160
FT                   /note="3'-nitrotyrosine"
FT                   /evidence="ECO:0000250|UniProtKB:P20029"
FT   MOD_RES         213
FT                   /note="N6-acetyllysine"
FT                   /evidence="ECO:0000250|UniProtKB:P20029"
FT   MOD_RES         271
FT                   /note="N6-acetyllysine"
FT                   /evidence="ECO:0000250|UniProtKB:P0DMV8"
FT   MOD_RES         326
FT                   /note="N6-acetyllysine"
FT                   /evidence="ECO:0000250|UniProtKB:P20029"
FT   MOD_RES         353
FT                   /note="N6-acetyllysine; alternate"
FT                   /evidence="ECO:0000250|UniProtKB:P20029"
FT   MOD_RES         447
FT                   /note="N6-succinyllysine"
FT                   /evidence="ECO:0000250|UniProtKB:P20029"
FT   MOD_RES         492
FT                   /note="Omega-N-methylarginine"
FT                   /evidence="ECO:0000250|UniProtKB:P0DMV8"
FT   MOD_RES         518
FT                   /note="O-AMP-threonine; alternate"
FT                   /evidence="ECO:0000250|UniProtKB:G3I8R9"
FT   MOD_RES         518
FT                   /note="Phosphothreonine; alternate"
FT                   /evidence="ECO:0007744|PubMed:20068231,
FT                   ECO:0007744|PubMed:24275569"
FT   MOD_RES         585
FT                   /note="N6,N6,N6-trimethyllysine; by METTL21A; in vitro"
FT                   /evidence="ECO:0000269|PubMed:23349634,
FT                   ECO:0000269|PubMed:23921388, ECO:0007744|PubMed:24129315"
FT   MOD_RES         585
FT                   /note="N6,N6-dimethyllysine; alternate"
FT                   /evidence="ECO:0007744|PubMed:24129315"
FT   MOD_RES         585
FT                   /note="N6-methyllysine; alternate"
FT                   /evidence="ECO:0007744|PubMed:24129315"
FT   MOD_RES         591
FT                   /note="N6-methyllysine"
FT                   /evidence="ECO:0007744|PubMed:24129315"
FT   MOD_RES         643
FT                   /note="Phosphothreonine"
FT                   /evidence="ECO:0000250|UniProtKB:P20029"
FT   MOD_RES         648
FT                   /note="Phosphothreonine"
FT                   /evidence="ECO:0000250|UniProtKB:P20029"
FT   CROSSLNK        352
FT                   /note="Glycyl lysine isopeptide (Lys-Gly) (interchain with
FT                   G-Cter in SUMO2)"
FT                   /evidence="ECO:0007744|PubMed:25114211,
FT                   ECO:0007744|PubMed:25218447, ECO:0007744|PubMed:25755297,
FT                   ECO:0007744|PubMed:28112733"
FT   CROSSLNK        353
FT                   /note="Glycyl lysine isopeptide (Lys-Gly) (interchain with
FT                   G-Cter in SUMO1); alternate"
FT                   /evidence="ECO:0007744|PubMed:25114211"
FT   VARIANT         543
FT                   /note="N -> H (in dbSNP:rs35356639)"
FT                   /evidence="ECO:0000269|Ref.5"
FT                   /id="VAR_025815"
FT   MUTAGEN         229
FT                   /note="T->A: Impaired ATPase activity."
FT                   /evidence="ECO:0000269|PubMed:26655470"
FT   MUTAGEN         585
FT                   /note="K->R: Complete loss of in vitro methylation by
FT                   METTL21A."
FT                   /evidence="ECO:0000269|PubMed:23349634,
FT                   ECO:0000269|PubMed:23921388"
FT   CONFLICT        297
FT                   /note="Missing (in Ref. 1; AAA52614 and 2; CAA61201)"
FT                   /evidence="ECO:0000305"
FT   CONFLICT        418
FT                   /note="D -> H (in Ref. 1; AAA52614 and 2; CAA61201)"
FT                   /evidence="ECO:0000305"
FT   CONFLICT        439
FT                   /note="R -> S (in Ref. 1; AAA52614 and 2; CAA61201)"
FT                   /evidence="ECO:0000305"
FT   CONFLICT        447
FT                   /note="K -> N (in Ref. 1; AAA52614 and 2; CAA61201)"
FT                   /evidence="ECO:0000305"
FT   STRAND          31..34
FT                   /evidence="ECO:0007829|PDB:5F0X"
FT   STRAND          37..45
FT                   /evidence="ECO:0007829|PDB:5F0X"
FT   STRAND          47..52
FT                   /evidence="ECO:0007829|PDB:5F0X"
FT   STRAND          60..63
FT                   /evidence="ECO:0007829|PDB:5F0X"
FT   STRAND          66..68
FT                   /evidence="ECO:0007829|PDB:5F0X"
FT   STRAND          74..76
FT                   /evidence="ECO:0007829|PDB:5F0X"
FT   HELIX           78..82
FT                   /evidence="ECO:0007829|PDB:5F0X"
FT   HELIX           84..86
FT                   /evidence="ECO:0007829|PDB:6ASY"
FT   HELIX           88..90
FT                   /evidence="ECO:0007829|PDB:5F0X"
FT   STRAND          91..93
FT                   /evidence="ECO:0007829|PDB:6ASY"
FT   HELIX           95..97
FT                   /evidence="ECO:0007829|PDB:5F0X"
FT   TURN            98..100
FT                   /evidence="ECO:0007829|PDB:5F0X"
FT   HELIX           106..114
FT                   /evidence="ECO:0007829|PDB:5F0X"
FT   STRAND          116..122
FT                   /evidence="ECO:0007829|PDB:5F0X"
FT   STRAND          125..131
FT                   /evidence="ECO:0007829|PDB:5F0X"
FT   STRAND          137..140
FT                   /evidence="ECO:0007829|PDB:5F0X"
FT   HELIX           142..161
FT                   /evidence="ECO:0007829|PDB:5F0X"
FT   STRAND          167..172
FT                   /evidence="ECO:0007829|PDB:5F0X"
FT   HELIX           178..190
FT                   /evidence="ECO:0007829|PDB:5F0X"
FT   STRAND          194..200
FT                   /evidence="ECO:0007829|PDB:5F0X"
FT   HELIX           201..208
FT                   /evidence="ECO:0007829|PDB:5F0X"
FT   TURN            209..212
FT                   /evidence="ECO:0007829|PDB:5F0X"
FT   STRAND          215..225
FT                   /evidence="ECO:0007829|PDB:5F0X"
FT   STRAND          230..238
FT                   /evidence="ECO:0007829|PDB:5F0X"
FT   STRAND          241..250
FT                   /evidence="ECO:0007829|PDB:5F0X"
FT   HELIX           255..274
FT                   /evidence="ECO:0007829|PDB:5F0X"
FT   HELIX           278..280
FT                   /evidence="ECO:0007829|PDB:5F0X"
FT   HELIX           282..298
FT                   /evidence="ECO:0007829|PDB:5F0X"
FT   TURN            299..301
FT                   /evidence="ECO:0007829|PDB:5F0X"
FT   STRAND          302..313
FT                   /evidence="ECO:0007829|PDB:5F0X"
FT   STRAND          316..323
FT                   /evidence="ECO:0007829|PDB:5F0X"
FT   HELIX           324..337
FT                   /evidence="ECO:0007829|PDB:5F0X"
FT   HELIX           339..348
FT                   /evidence="ECO:0007829|PDB:5F0X"
FT   HELIX           353..355
FT                   /evidence="ECO:0007829|PDB:5F0X"
FT   STRAND          358..363
FT                   /evidence="ECO:0007829|PDB:5F0X"
FT   HELIX           364..367
FT                   /evidence="ECO:0007829|PDB:5F0X"
FT   HELIX           369..378
FT                   /evidence="ECO:0007829|PDB:5F0X"
FT   TURN            379..381
FT                   /evidence="ECO:0007829|PDB:5F0X"
FT   TURN            390..392
FT                   /evidence="ECO:0007829|PDB:5F0X"
FT   HELIX           393..405
FT                   /evidence="ECO:0007829|PDB:5F0X"
FT   STRAND          415..419
FT                   /evidence="ECO:0007829|PDB:6ASY"
FT   STRAND          424..428
FT                   /evidence="ECO:0007829|PDB:6ASY"
FT   TURN            429..431
FT                   /evidence="ECO:0007829|PDB:5E85"
FT   STRAND          433..437
FT                   /evidence="ECO:0007829|PDB:6ASY"
FT   STRAND          442..451
FT                   /evidence="ECO:0007829|PDB:6ASY"
FT   STRAND          461..468
FT                   /evidence="ECO:0007829|PDB:6ASY"
FT   HELIX           473..475
FT                   /evidence="ECO:0007829|PDB:6ASY"
FT   STRAND          476..484
FT                   /evidence="ECO:0007829|PDB:6ASY"
FT   STRAND          491..493
FT                   /evidence="ECO:0007829|PDB:6ASY"
FT   STRAND          497..503
FT                   /evidence="ECO:0007829|PDB:6ASY"
FT   STRAND          509..515
FT                   /evidence="ECO:0007829|PDB:6ASY"
FT   TURN            516..518
FT                   /evidence="ECO:0007829|PDB:6ASY"
FT   STRAND          521..526
FT                   /evidence="ECO:0007829|PDB:5E85"
FT   TURN            528..531
FT                   /evidence="ECO:0007829|PDB:5E85"
FT   HELIX           535..576
FT                   /evidence="ECO:0007829|PDB:6ASY"
FT   TURN            579..581
FT                   /evidence="ECO:0007829|PDB:6ASY"
FT   HELIX           582..585
FT                   /evidence="ECO:0007829|PDB:6ASY"
FT   HELIX           588..607
FT                   /evidence="ECO:0007829|PDB:6ASY"
FT   HELIX           613..631
FT                   /evidence="ECO:0007829|PDB:6ASY"
SQ   SEQUENCE   654 AA;  72333 MW;  59B7D8D85BC32A00 CRC64;
     MKLSLVAAML LLLSAARAEE EDKKEDVGTV VGIDLGTTYS CVGVFKNGRV EIIANDQGNR
     ITPSYVAFTP EGERLIGDAA KNQLTSNPEN TVFDAKRLIG RTWNDPSVQQ DIKFLPFKVV
     EKKTKPYIQV DIGGGQTKTF APEEISAMVL TKMKETAEAY LGKKVTHAVV TVPAYFNDAQ
     RQATKDAGTI AGLNVMRIIN EPTAAAIAYG LDKREGEKNI LVFDLGGGTF DVSLLTIDNG
     VFEVVATNGD THLGGEDFDQ RVMEHFIKLY KKKTGKDVRK DNRAVQKLRR EVEKAKRALS
     SQHQARIEIE SFYEGEDFSE TLTRAKFEEL NMDLFRSTMK PVQKVLEDSD LKKSDIDEIV
     LVGGSTRIPK IQQLVKEFFN GKEPSRGINP DEAVAYGAAV QAGVLSGDQD TGDLVLLDVC
     PLTLGIETVG GVMTKLIPRN TVVPTKKSQI FSTASDNQPT VTIKVYEGER PLTKDNHLLG
     TFDLTGIPPA PRGVPQIEVT FEIDVNGILR VTAEDKGTGN KNKITITNDQ NRLTPEEIER
     MVNDAEKFAE EDKKLKERID TRNELESYAY SLKNQIGDKE KLGGKLSSED KETMEKAVEE
     KIEWLESHQD ADIEDFKAKK KELEEIVQPI ISKLYGSAGP PPTGEEDTAE KDEL
```

|  |
| --- |
| **Mascot:** http://www.matrixscience.com/ |
