# Supplementary material for: Chlorpromazine induces cytotoxic autophagy in glioblastoma cells via endoplasmic reticulum stress and unfolded protein response
Source: J Exp Clin Cancer Res. 2021 Nov 5;40:347. doi: 10.1186/s13046-021-02144-w (PMC8569984; doi:10.1186/s13046-021-02144-w)
Supplement: Supplementary file 1 — Additional file 1. [file 13046_2021_2144_MOESM1_ESM.zip › MASCOT IDs/Mascot Search Results_ EF1A1_HUMAN.html]

Mascot Search Results: EF1A1\_HUMAN
 

# MASCOT Search Results

## Protein View: EF1A1\_HUMAN

### Elongation factor 1-alpha 1 OS=Homo sapiens OX=9606 GN=EEF1A1 PE=1 SV=1

|  |  |
| --- | --- |
| Database: | SwissProt |
| Score: | 88 |
| Monoisotopic mass (Mr): | 50451 |
| Calculated pI: | 9.10 |
| Taxonomy: | Homo sapiens |

Sequence similarity is available as an NCBI BLAST search of EF1A1\_HUMAN against nr.

### Search parameters

|  |  |
| --- | --- |
| MS data file: | `ppw_D15_157002891000.txt` |
| Enzyme: | Trypsin: cuts C-term side of KR unless next residue is P. |
| Fixed modifications: | Carbamidomethyl (C) |

### Protein sequence coverage: 10%

Matched peptides shown in ***bold red***.

|  |  |  |  |  |  |
| --- | --- | --- | --- | --- | --- |
| `1` | `MGKEKTHINI` | `VVIGHVDSGK` | `STTTGHLIYK` | `CGGIDKRTIE` | `KFEKEAAEMG` |
| `51` | `KGSFKYAWVL` | `DKLKAERERG` | `ITIDISLWKF` | `ETSKYYVTII` | `DAPGHRDFIK` |
| `101` | `NMITGTSQAD` | `CAVLIVAAGV` | `GEFEAGISKN` | `GQTREHALLA` | `YTLGVKQLIV` |
| `151` | `GVNKMDSTEP` | `PYSQKRYEEI` | `VKEVSTYIKK` | `IGYNPDTVAF` | `VPISGWNGDN` |
| `201` | `MLEPSANMPW` | `FKGWKVTRKD` | `GNASGTTLLE` | `ALDCILPPTR` | `PTDKPLRLPL` |
| `251` | `QDVYKIGGIG` | `TVPVGRVETG` | `VLKPGMVVTF` | `APVNVTTEVK` | `SVEMHHEALS` |
| `301` | `EALPGDNVGF` | `NVKNVSVKDV` | `RRGNVAGDSK` | `NDPPMEAAGF` | `TAQVIILNHP` |
| `351` | `GQISAGYAPV` | `LDCHTAHIAC` | `KFAELKEKID` | `RRSGKKLEDG` | `PKFLKSGDAA` |
| `401` | `IVDMVPGKPM` | `CVESFSDYPP` | `LGRFAVRDMR` | `QTVAVGVIKA` | `VDKKAAGAGK` |
| `451` | `VTKSAQKAQK` | `AK` |  |  |  |

Unformatted sequence string: 462 residues (for pasting into other applications).

|  |  |  |  |
| --- | --- | --- | --- |
| Sort by | residue number | increasing mass | decreasing mass |
| Show | matched peptides only | predicted peptides also |  |

| Query | Start | – | End | Observed | Mr(expt) | Mr(calc) | ppm | M | Score | Expect | Rank | U | Peptide |
| --- | --- | --- | --- | --- | --- | --- | --- | --- | --- | --- | --- | --- | --- |
| 17 | 256 | – | 266 | 1025.6525 | 1024.6452 | 1024.6030 | 41.3 | 0 | 76 | 2.1e-07 | 1Score **> 22** indicates **identity** | U | K.IGGIGTVPVGR.V |
| 93 | 267 | – | 290 | 2515.4658 | 2514.4585 | 2514.3768 | 32.5 | 0 | 22 | 0.046 | 1Score **> 21** indicates **identity** Score **> 14** indicates **homology** | U | R.VETGVLKPGMVVTFAPVNVTTEVK.S |
| 35 | 428 | – | 439 | 1316.7772 | 1315.7699 | 1315.7282 | 31.7 | 1 | 31 | 0.017 | 1Score **> 26** indicates **identity** | U | R.DMRQTVAVGVIK.A |

---

```
ID   EF1A1_HUMAN             Reviewed;         462 AA.
AC   P68104; P04719; P04720; Q6IQ15;
DT   13-AUG-1987, integrated into UniProtKB/Swiss-Prot.
DT   13-AUG-1987, sequence version 1.
DT   02-JUN-2021, entry version 195.
DE   RecName: Full=Elongation factor 1-alpha 1;
DE            Short=EF-1-alpha-1;
DE   AltName: Full=Elongation factor Tu;
DE            Short=EF-Tu;
DE   AltName: Full=Eukaryotic elongation factor 1 A-1;
DE            Short=eEF1A-1;
DE   AltName: Full=Leukocyte receptor cluster member 7;
GN   Name=EEF1A1; Synonyms=EEF1A, EF1A, LENG7;
OS   Homo sapiens (Human).
OC   Eukaryota; Metazoa; Chordata; Craniata; Vertebrata; Euteleostomi; Mammalia;
OC   Eutheria; Euarchontoglires; Primates; Haplorrhini; Catarrhini; Hominidae;
OC   Homo.
OX   NCBI_TaxID=9606;
RN   [1]
RP   NUCLEOTIDE SEQUENCE [MRNA].
RX   PubMed=3512269; DOI=10.1111/j.1432-1033.1986.tb09472.x;
RA   Brands J.H.G.M., Maassen J.A., van Hemert F.J., Amons R., Moeller W.;
RT   "The primary structure of the alpha subunit of human elongation factor 1.
RT   Structural aspects of guanine-nucleotide-binding sites.";
RL   Eur. J. Biochem. 155:167-171(1986).
RN   [2]
RP   NUCLEOTIDE SEQUENCE [GENOMIC DNA].
RX   PubMed=2564392;
RA   Uetsuki T., Naito A., Nagata S., Kaziro Y.;
RT   "Isolation and characterization of the human chromosomal gene for
RT   polypeptide chain elongation factor-1 alpha.";
RL   J. Biol. Chem. 264:5791-5798(1989).
RN   [3]
RP   NUCLEOTIDE SEQUENCE [MRNA].
RC   TISSUE=Liver;
RX   PubMed=2183196; DOI=10.1093/nar/18.6.1513;
RA   Madsen H.O., Poulsen K., Dahl O., Clark B.F.C., Hjorth J.P.;
RT   "Retropseudogenes constitute the major part of the human elongation factor
RT   1 alpha gene family.";
RL   Nucleic Acids Res. 18:1513-1516(1990).
RN   [4]
RP   NUCLEOTIDE SEQUENCE [MRNA].
RA   Shimazu T., Koike K.;
RT   "Postnatal expression of a novel mRNA isoform from the human elongation
RT   factor-1a gene.";
RL   Submitted (JUL-2001) to the EMBL/GenBank/DDBJ databases.
RN   [5]
RP   NUCLEOTIDE SEQUENCE [LARGE SCALE MRNA].
RC   TISSUE=B-cell, Bone marrow, Cervix, Colon, Hippocampus, Kidney, Lung,
RC   Lymph, Mammary gland, Ovary, Pancreas, Placenta, Testis, and Uterus;
RX   PubMed=15489334; DOI=10.1101/gr.2596504;
RG   The MGC Project Team;
RT   "The status, quality, and expansion of the NIH full-length cDNA project:
RT   the Mammalian Gene Collection (MGC).";
RL   Genome Res. 14:2121-2127(2004).
RN   [6]
RP   NUCLEOTIDE SEQUENCE [MRNA] OF 1-94.
RX   PubMed=3960725; DOI=10.1093/nar/14.5.2409;
RA   Rao T.R., Slobin L.I.;
RT   "Structure of the amino-terminal end of mammalian elongation factor Tu.";
RL   Nucleic Acids Res. 14:2409-2409(1986).
RN   [7]
RP   PROTEIN SEQUENCE OF 6-30; 52-62; 85-96; 101-129; 135-180; 248-313; 396-423
RP   AND 431-439, METHYLATION AT LYS-55 AND LYS-165, AND IDENTIFICATION BY MASS
RP   SPECTROMETRY.
RC   TISSUE=Colon carcinoma, and Ovarian carcinoma;
RA   Bienvenut W.V., Zebisch A., Kolch W.;
RL   Submitted (JAN-2010) to UniProtKB.
RN   [8]
RP   PROTEIN SEQUENCE OF 7-16 AND 85-96, FUNCTION, INTERACTION WITH PARP1 AND
RP   TXK, PHOSPHORYLATION BY TXK, AND SUBCELLULAR LOCATION.
RX   PubMed=17177976; DOI=10.1111/j.1365-2249.2006.03249.x;
RA   Maruyama T., Nara K., Yoshikawa H., Suzuki N.;
RT   "Txk, a member of the non-receptor tyrosine kinase of the Tec family, forms
RT   a complex with poly(ADP-ribose) polymerase 1 and elongation factor 1alpha
RT   and regulates interferon-gamma gene transcription in Th1 cells.";
RL   Clin. Exp. Immunol. 147:164-175(2007).
RN   [9]
RP   PROTEIN SEQUENCE OF 38-44; 70-79; 85-96; 135-172; 248-290; 386-392 AND
RP   428-439.
RC   TISSUE=B-cell lymphoma;
RA   Bienvenut W.V.;
RL   Submitted (JUN-2005) to UniProtKB.
RN   [10]
RP   NUCLEOTIDE SEQUENCE [MRNA] OF 138-462.
RX   PubMed=3346208;
RA   Ann D.K., Wu M.M.J., Huang T., Carlson D.M., Wu R.;
RT   "Retinol-regulated gene expression in human tracheobronchial epithelial
RT   cells. Enhanced expression of elongation factor EF-1 alpha.";
RL   J. Biol. Chem. 263:3546-3549(1988).
RN   [11]
RP   ETHANOLAMINYLATION AT GLU-301 AND GLU-374.
RX   PubMed=2569467;
RA   Whiteheart S.W., Shenbagarmurthi P., Chen L., Cotter R.J., Hart G.W.;
RT   "Murine elongation factor 1 alpha (EF-1 alpha) is posttranslationally
RT   modified by novel amide-linked ethanolamine-phosphoglycerol moieties.
RT   Addition of ethanolamine-phosphoglycerol to specific glutamic acid residues
RT   on EF-1 alpha.";
RL   J. Biol. Chem. 264:14334-14341(1989).
RN   [12]
RP   INTERACTION WITH ZPR1, AND SUBCELLULAR LOCATION.
RX   PubMed=8650580; DOI=10.1126/science.272.5269.1797;
RA   Galcheva-Gargova Z., Konstantinov K.N., Wu I.-H., Klier F.G., Barrett T.,
RA   Davis R.J.;
RT   "Binding of zinc finger protein ZPR1 to the epidermal growth factor
RT   receptor.";
RL   Science 272:1797-1802(1996).
RN   [13]
RP   INDUCTION BY HOMOCYSTEINE.
RX   PubMed=9677419; DOI=10.1074/jbc.273.31.19840;
RA   Chacko G., Ling Q., Hajjar K.A.;
RT   "Induction of acute translational response genes by homocysteine.
RT   Elongation factors-1alpha, -beta, and -delta.";
RL   J. Biol. Chem. 273:19840-19846(1998).
RN   [14]
RP   IDENTIFICATION IN A NUCLEAR EXPORT RECEPTOR COMPLEX WITH XPO5; RAN AND
RP   TRNA, AND INTERACTION WITH XPO5.
RX   PubMed=12426392; DOI=10.1093/emboj/cdf613;
RA   Bohnsack M.T., Regener K., Schwappach B., Saffrich R., Paraskeva E.,
RA   Hartmann E., Goerlich D.;
RT   "Exp5 exports eEF1A via tRNA from nuclei and synergizes with other
RT   transport pathways to confine translation to the cytoplasm.";
RL   EMBO J. 21:6205-6215(2002).
RN   [15]
RP   IDENTIFICATION IN A NUCLEAR EXPORT RECEPTOR COMPLEX WITH XPO5; RAN AND
RP   TRNA, AND INTERACTION WITH XPO5.
RX   PubMed=12426393; DOI=10.1093/emboj/cdf620;
RA   Calado A., Treichel N., Mueller E.-C., Otto A., Kutay U.;
RT   "Exportin-5-mediated nuclear export of eukaryotic elongation factor 1A and
RT   tRNA.";
RL   EMBO J. 21:6216-6224(2002).
RN   [16]
RP   ISGYLATION.
RX   PubMed=16139798; DOI=10.1016/j.bbrc.2005.08.132;
RA   Giannakopoulos N.V., Luo J.K., Papov V., Zou W., Lenschow D.J.,
RA   Jacobs B.S., Borden E.C., Li J., Virgin H.W., Zhang D.E.;
RT   "Proteomic identification of proteins conjugated to ISG15 in mouse and
RT   human cells.";
RL   Biochem. Biophys. Res. Commun. 336:496-506(2005).
RN   [17]
RP   PHOSPHORYLATION AT THR-432, AND MUTAGENESIS OF THR-432.
RX   PubMed=17595531; DOI=10.1159/000104169;
RA   Eckhardt K., Troger J., Reissmann J., Katschinski D.M., Wagner K.F.,
RA   Stengel P., Paasch U., Hunziker P., Borter E., Barth S., Schlafli P.,
RA   Spielmann P., Stiehl D.P., Camenisch G., Wenger R.H.;
RT   "Male germ cell expression of the PAS domain kinase PASKIN and its novel
RT   target eukaryotic translation elongation factor eEF1A1.";
RL   Cell. Physiol. Biochem. 20:227-240(2007).
RN   [18]
RP   INTERACTION WITH KARS1.
RX   PubMed=18029264; DOI=10.1016/j.bbrc.2007.11.028;
RA   Guzzo C.M., Yang D.C.H.;
RT   "Lysyl-tRNA synthetase interacts with EF1alpha, aspartyl-tRNA synthetase
RT   and p38 in vitro.";
RL   Biochem. Biophys. Res. Commun. 365:718-723(2008).
RN   [19]
RP   INTERACTION WITH ERGIC2.
RX   PubMed=17980171; DOI=10.1016/j.bbapap.2007.10.006;
RA   Yang Y.F., Chou M.Y., Fan C.Y., Chen S.F., Lyu P.C., Liu C.C., Tseng T.L.;
RT   "The possible interaction of CDA14 and protein elongation factor 1alpha.";
RL   Biochim. Biophys. Acta 1784:312-318(2008).
RN   [20]
RP   INTERACTION WITH SPHK1 AND SPHK2.
RX   PubMed=18263879; DOI=10.1074/jbc.m708782200;
RA   Leclercq T.M., Moretti P.A., Vadas M.A., Pitson S.M.;
RT   "Eukaryotic elongation factor 1A interacts with sphingosine kinase and
RT   directly enhances its catalytic activity.";
RL   J. Biol. Chem. 283:9606-9614(2008).
RN   [21]
RP   INTERACTION WITH DLC1, AND SUBCELLULAR LOCATION.
RX   PubMed=19158340; DOI=10.1242/jcs.027482;
RA   Zhong D., Zhang J., Yang S., Soh U.J., Buschdorf J.P., Zhou Y.T., Yang D.,
RA   Low B.C.;
RT   "The SAM domain of the RhoGAP DLC1 binds EF1A1 to regulate cell
RT   migration.";
RL   J. Cell Sci. 122:414-424(2009).
RN   [22]
RP   PHOSPHORYLATION AT SER-300.
RX   PubMed=20832312; DOI=10.1016/j.cub.2010.08.017;
RA   Lin K.W., Yakymovych I., Jia M., Yakymovych M., Souchelnytskyi S.;
RT   "Phosphorylation of eEF1A1 at Ser300 by TbetaR-I results in inhibition of
RT   mRNA translation.";
RL   Curr. Biol. 20:1615-1625(2010).
RN   [23]
RP   METHYLATION AT LYS-318, AND MUTAGENESIS OF LYS-36; LYS-55; LYS-79; LYS-165
RP   AND LYS-318.
RX   PubMed=25144183; DOI=10.1371/journal.pone.0105394;
RA   Shimazu T., Barjau J., Sohtome Y., Sodeoka M., Shinkai Y.;
RT   "Selenium-based S-adenosylmethionine analog reveals the mammalian seven-
RT   beta-strand methyltransferase METTL10 to be an EF1A1 lysine
RT   methyltransferase.";
RL   PLoS ONE 9:E105394-E105394(2014).
RN   [24]
RP   SUBCELLULAR LOCATION, INTERACTION WITH PPP1R16B, AND PHOSPHORYLATION BY
RP   ROCK2.
RX   PubMed=26497934; DOI=10.1016/j.biocel.2015.10.021;
RA   Boratko A., Peter M., Thalwieser Z., Kovacs E., Csortos C.;
RT   "Elongation factor-1A1 is a novel substrate of the protein phosphatase 1-
RT   TIMAP complex.";
RL   Int. J. Biochem. Cell Biol. 69:105-113(2015).
RN   [25]
RP   METHYLATION AT GLY-2 AND LYS-79.
RX   PubMed=26545399; DOI=10.1074/mcp.m115.052449;
RA   Hamey J.J., Winter D.L., Yagoub D., Overall C.M., Hart-Smith G.,
RA   Wilkins M.R.;
RT   "Novel N-terminal and lysine methyltransferases that target translation
RT   elongation factor 1A in yeast and human.";
RL   Mol. Cell. Proteomics 15:164-176(2016).
RN   [26]
RP   METHYLATION AT LYS-165, AND MUTAGENESIS OF LYS-165.
RX   PubMed=28108655; DOI=10.1093/nar/gkx002;
RA   Malecki J., Aileni V.K., Ho A.Y., Schwarz J., Moen A., Soerensen V.,
RA   Nilges B.S., Jakobsson M.E., Leidel S.A., Falnes P.O.;
RT   "The novel lysine specific methyltransferase METTL21B affects mRNA
RT   translation through inducible and dynamic methylation of Lys-165 in human
RT   eukaryotic elongation factor 1 alpha (eEF1A).";
RL   Nucleic Acids Res. 45:4370-4389(2017).
RN   [27]
RP   METHYLATION AT LYS-36, IDENTIFICATION BY MASS SPECTROMETRY, AND MUTAGENESIS
RP   OF LYS-36.
RX   PubMed=28520920; DOI=10.1093/nar/gkx432;
RA   Jakobsson M.E., Malecki J., Nilges B.S., Moen A., Leidel S.A., Falnes P.O.;
RT   "Methylation of human eukaryotic elongation factor alpha (eEF1A) by a
RT   member of a novel protein lysine methyltransferase family modulates mRNA
RT   translation.";
RL   Nucleic Acids Res. 45:8239-8254(2017).
RN   [28]
RP   METHYLATION AT LYS-55 BY EEF1AKNMT, AND MUTAGENESIS OF LYS-55.
RX   PubMed=30612740; DOI=10.1016/j.cell.2018.11.038;
RA   Liu S., Hausmann S., Carlson S.M., Fuentes M.E., Francis J.W., Pillai R.,
RA   Lofgren S.M., Hulea L., Tandoc K., Lu J., Li A., Nguyen N.D., Caporicci M.,
RA   Kim M.P., Maitra A., Wang H., Wistuba I.I., Porco J.A. Jr., Bassik M.C.,
RA   Elias J.E., Song J., Topisirovic I., Van Rechem C., Mazur P.K., Gozani O.;
RT   "METTL13 methylation of eEF1A increases translational output to promote
RT   tumorigenesis.";
RL   Cell 0:0-0(2018).
RN   [29]
RP   METHYLATION AT GLY-2 AND LYS-55 BY EEF1AKNMT, AND MUTAGENESIS OF LYS-55.
RX   PubMed=30143613; DOI=10.1038/s41467-018-05646-y;
RA   Jakobsson M.E., Malecki J.M., Halabelian L., Nilges B.S., Pinto R.,
RA   Kudithipudi S., Munk S., Davydova E., Zuhairi F.R., Arrowsmith C.H.,
RA   Jeltsch A., Leidel S.A., Olsen J.V., Falnes P.O.;
RT   "The dual methyltransferase METTL13 targets N terminus and Lys55 of eEF1A
RT   and modulates codon-specific translation rates.";
RL   Nat. Commun. 9:3411-3411(2018).
RN   [30]
RP   FUNCTION (MICROBIAL INFECTION), ACTIVITY REGULATION, AND MUTAGENESIS OF
RP   ALA-399.
RX   PubMed=33495306; DOI=10.1126/science.abf4058;
RA   White K.M., Rosales R., Yildiz S., Kehrer T., Miorin L., Moreno E.,
RA   Jangra S., Uccellini M.B., Rathnasinghe R., Coughlan L.,
RA   Martinez-Romero C., Batra J., Rojc A., Bouhaddou M., Fabius J.M.,
RA   Obernier K., Dejosez M., Guillen M.J., Losada A., Aviles P., Schotsaert M.,
RA   Zwaka T., Vignuzzi M., Shokat K.M., Krogan N.J., Garcia-Sastre A.;
RT   "Plitidepsin has potent preclinical efficacy against SARS-CoV-2 by
RT   targeting the host protein eEF1A.";
RL   Science 0:0-0(2021).
CC   -!- FUNCTION: This protein promotes the GTP-dependent binding of aminoacyl-
CC       tRNA to the A-site of ribosomes during protein biosynthesis. Plays a
CC       role in the positive regulation of IFNG transcription in T-helper 1
CC       cells as part of an IFNG promoter-binding complex with TXK and PARP1
CC       (PubMed:17177976). {ECO:0000269|PubMed:17177976}.
CC   -!- FUNCTION: (Microbial infection) Required for the translation of viral
CC       proteins and viral replication during human coronavirus SARS-CoV-2
CC       infection. {ECO:0000269|PubMed:33495306}.
CC   -!- ACTIVITY REGULATION: Inhibited by plitidepsin, a chemical compound
CC       extracted from the ascidian Aplidium albicans.
CC       {ECO:0000269|PubMed:33495306}.
CC   -!- SUBUNIT: Found in a nuclear export complex with XPO5, EEF1A1, Ran and
CC       aminoacylated tRNA (PubMed:12426392, PubMed:12426393). Interacts with
CC       PARP1 and TXK (PubMed:17177976). Interacts with KARS1
CC       (PubMed:18029264). May interact with ERGIC2. Interacts with IFIT1 (via
CC       TPR repeats 4-7) (By similarity). Interacts with DLC1, facilitating
CC       distribution to the membrane periphery and ruffles upon growth factor
CC       stimulation. Interacts with ZPR1; the interaction occurs in a epidermal
CC       growth factor (EGF)-dependent manner. Interacts with PPP1R16B
CC       (PubMed:26497934). Interacts with SPHK1 and SPHK2; both interactions
CC       increase SPHK1 and SPHK2 kinase activity (PubMed:18263879).
CC       {ECO:0000250, ECO:0000269|PubMed:12426392, ECO:0000269|PubMed:12426393,
CC       ECO:0000269|PubMed:17177976, ECO:0000269|PubMed:17980171,
CC       ECO:0000269|PubMed:18029264, ECO:0000269|PubMed:18263879,
CC       ECO:0000269|PubMed:19158340, ECO:0000269|PubMed:26497934,
CC       ECO:0000269|PubMed:8650580}.
CC   -!- INTERACTION:
CC       P68104; Q8NFJ9: BBS1; NbExp=3; IntAct=EBI-352162, EBI-1805484;
CC       P68104; Q96IK1-2: BOD1; NbExp=3; IntAct=EBI-352162, EBI-18924329;
CC       P68104; Q16539: MAPK14; NbExp=3; IntAct=EBI-352162, EBI-73946;
CC       P68104; Q00987: MDM2; NbExp=9; IntAct=EBI-352162, EBI-389668;
CC       P68104; Q9NZ94-2: NLGN3; NbExp=4; IntAct=EBI-352162, EBI-16423037;
CC       P68104; P12004: PCNA; NbExp=2; IntAct=EBI-352162, EBI-358311;
CC       P68104; P54725: RAD23A; NbExp=2; IntAct=EBI-352162, EBI-746453;
CC       P68104; Q9NYA1: SPHK1; NbExp=2; IntAct=EBI-352162, EBI-985303;
CC       P68104; Q14166: TTLL12; NbExp=4; IntAct=EBI-352162, EBI-923010;
CC       P68104; P63104: YWHAZ; NbExp=2; IntAct=EBI-352162, EBI-347088;
CC       P68104; Q05516: ZBTB16; NbExp=4; IntAct=EBI-352162, EBI-711925;
CC       P68104; Q8IUH5: ZDHHC17; NbExp=2; IntAct=EBI-352162, EBI-524753;
CC   -!- SUBCELLULAR LOCATION: Cytoplasm {ECO:0000269|PubMed:19158340,
CC       ECO:0000269|PubMed:8650580}. Nucleus {ECO:0000269|PubMed:17177976,
CC       ECO:0000269|PubMed:8650580}. Nucleus, nucleolus
CC       {ECO:0000269|PubMed:8650580}. Cell membrane
CC       {ECO:0000269|PubMed:26497934}. Note=Colocalizes with DLC1 at actin-rich
CC       regions in the cell periphery (PubMed:19158340). Translocates together
CC       with ZPR1 from the cytoplasm to the nucleus and nucleolus after
CC       treatment with mitogens (PubMed:8650580). Localization at the cell
CC       membrane depends on EEF1A1 phosphorylation status and the presence of
CC       PPP1R16B (PubMed:26497934). {ECO:0000269|PubMed:19158340,
CC       ECO:0000269|PubMed:26497934, ECO:0000269|PubMed:8650580}.
CC   -!- TISSUE SPECIFICITY: Brain, placenta, lung, liver, kidney, pancreas but
CC       barely detectable in heart and skeletal muscle.
CC   -!- INDUCTION: By homocysteine (HC), may mediate accelerated synthesis of
CC       free thiol-containing proteins in response to HC-induced oxidative
CC       stress. {ECO:0000269|PubMed:9677419}.
CC   -!- PTM: ISGylated. {ECO:0000269|PubMed:16139798}.
CC   -!- PTM: Phosphorylated by TXK. Phosphorylation by PASK increases
CC       translation efficiency. Phosphorylated by ROCK2 (PubMed:26497934).
CC       {ECO:0000269|PubMed:17177976, ECO:0000269|PubMed:17595531,
CC       ECO:0000269|PubMed:20832312, ECO:0000269|PubMed:26497934}.
CC   -!- PTM: Trimethylated at Lys-79 by EEF1AKMT1 (PubMed:26545399). Methylated
CC       at Lys-165 by EEF1AKMT3, methylation by EEF1AKMT3 is dynamic as well as
CC       inducible by stress conditions, such as ER-stress, and plays a
CC       regulatory role on mRNA translation (PubMed:28108655). Trimethylated at
CC       Lys-318 by EEF1AKMT2 (PubMed:25144183). Mono-, di-, and trimethylated
CC       at Lys-36 by EEF1AKMT4; trimethylated form is predominant. Methylation
CC       by EEF1AKMT4 contributes to the fine-tuning of translation rates for a
CC       subset of tRNAs (PubMed:28520920). Trimethylated at Gly-2 by EEF1AKNMT
CC       (PubMed:30143613). Mono- and dimethylated at Lys-55 by EEF1AKNMT;
CC       dimethylated form is predominant (PubMed:30143613, PubMed:30612740).
CC       {ECO:0000269|PubMed:25144183, ECO:0000269|PubMed:26545399,
CC       ECO:0000269|PubMed:28108655, ECO:0000269|PubMed:28520920,
CC       ECO:0000269|PubMed:30143613, ECO:0000269|PubMed:30612740}.
CC   -!- SIMILARITY: Belongs to the TRAFAC class translation factor GTPase
CC       superfamily. Classic translation factor GTPase family. EF-Tu/EF-1A
CC       subfamily. {ECO:0000305}.
CC   -!- SEQUENCE CAUTION:
CC       Sequence=AAA52367.1; Type=Erroneous initiation; Note=Extended N-terminus.; Evidence={ECO:0000305};
CC       Sequence=AAH71619.1; Type=Miscellaneous discrepancy; Note=Probable cloning artifact.; Evidence={ECO:0000305};
CC   -!- WEB RESOURCE: Name=Atlas of Genetics and Cytogenetics in Oncology and
CC       Haematology;
CC       URL="http://atlasgeneticsoncology.org/Genes/EEF1A1ID40407ch6q13.html";
CC   ---------------------------------------------------------------------------
CC   Copyrighted by the UniProt Consortium, see https://www.uniprot.org/terms
CC   Distributed under the Creative Commons Attribution (CC BY 4.0) License
CC   ---------------------------------------------------------------------------
DR   EMBL; X03558; CAA27245.1; -; mRNA.
DR   EMBL; J04617; AAA52343.1; -; Genomic_DNA.
DR   EMBL; X16869; CAA34756.1; -; mRNA.
DR   EMBL; AY043301; AAK95378.1; -; mRNA.
DR   EMBL; BC008587; AAH08587.1; -; mRNA.
DR   EMBL; BC009733; AAH09733.1; -; mRNA.
DR   EMBL; BC009875; AAH09875.1; -; mRNA.
DR   EMBL; BC010735; AAH10735.1; -; mRNA.
DR   EMBL; BC012891; AAH12891.1; -; mRNA.
DR   EMBL; BC014224; AAH14224.1; -; mRNA.
DR   EMBL; BC018150; AAH18150.1; -; mRNA.
DR   EMBL; BC018641; AAH18641.1; -; mRNA.
DR   EMBL; BC021686; AAH21686.1; -; mRNA.
DR   EMBL; BC028674; AAH28674.1; -; mRNA.
DR   EMBL; BC038339; AAH38339.1; -; mRNA.
DR   EMBL; BC057391; AAH57391.1; -; mRNA.
DR   EMBL; BC066893; AAH66893.1; -; mRNA.
DR   EMBL; BC071619; AAH71619.1; ALT_SEQ; mRNA.
DR   EMBL; BC072385; AAH72385.1; -; mRNA.
DR   EMBL; BC082268; AAH82268.1; -; mRNA.
DR   EMBL; X03689; CAA27325.1; -; mRNA.
DR   EMBL; M29548; AAA52367.1; ALT_INIT; mRNA.
DR   CCDS; CCDS4980.1; -.
DR   PIR; B24977; EFHU1.
DR   RefSeq; NP_001393.1; NM_001402.5.
DR   RefSeq; XP_011533816.1; XM_011535514.2.
DR   PDB; 1SYW; Model; -; A=2-443.
DR   PDB; 3C5J; X-ray; 1.80 A; C=343-355.
DR   PDB; 6ZMO; EM; 3.10 A; CD=1-462.
DR   PDBsum; 1SYW; -.
DR   PDBsum; 3C5J; -.
DR   PDBsum; 6ZMO; -.
DR   SMR; P68104; -.
DR   BioGRID; 108237; 517.
DR   CORUM; P68104; -.
DR   DIP; DIP-31277N; -.
DR   IntAct; P68104; 247.
DR   MINT; P68104; -.
DR   STRING; 9606.ENSP00000339063; -.
DR   BindingDB; P68104; -.
DR   ChEMBL; CHEMBL1795120; -.
DR   DrugBank; DB11638; Artenimol.
DR   DrugBank; DB09130; Copper.
DR   DrugBank; DB04315; Guanosine-5'-Diphosphate.
DR   DrugBank; DB01593; Zinc.
DR   DrugBank; DB14487; Zinc acetate.
DR   DrugBank; DB14533; Zinc chloride.
DR   DrugBank; DB14548; Zinc sulfate, unspecified form.
DR   MoonProt; P68104; -.
DR   GlyGen; P68104; 1 site.
DR   iPTMnet; P68104; -.
DR   MetOSite; P68104; -.
DR   PhosphoSitePlus; P68104; -.
DR   SwissPalm; P68104; -.
DR   BioMuta; EEF1A1; -.
DR   DMDM; 55584035; -.
DR   OGP; P68104; -.
DR   SWISS-2DPAGE; P68104; -.
DR   UCD-2DPAGE; P68104; -.
DR   EPD; P68104; -.
DR   jPOST; P68104; -.
DR   MassIVE; P68104; -.
DR   PaxDb; P68104; -.
DR   PeptideAtlas; P68104; -.
DR   PRIDE; P68104; -.
DR   ProteomicsDB; 66470; -.
DR   Antibodypedia; 31353; 326 antibodies.
DR   DNASU; 1915; -.
DR   Ensembl; ENST00000309268; ENSP00000339053; ENSG00000156508.
DR   Ensembl; ENST00000316292; ENSP00000339063; ENSG00000156508.
DR   Ensembl; ENST00000331523; ENSP00000330054; ENSG00000156508.
DR   Ensembl; ENST00000356303; ENSP00000348651; ENSG00000156508.
DR   Ensembl; ENST00000455918; ENSP00000392366; ENSG00000156508.
DR   Ensembl; ENST00000615060; ENSP00000479055; ENSG00000156508.
DR   Ensembl; ENST00000676710; ENSP00000504335; ENSG00000156508.
DR   Ensembl; ENST00000677236; ENSP00000503192; ENSG00000156508.
DR   Ensembl; ENST00000678508; ENSP00000503249; ENSG00000156508.
DR   Ensembl; ENST00000678702; ENSP00000503823; ENSG00000156508.
DR   GeneID; 1915; -.
DR   KEGG; hsa:1915; -.
DR   CTD; 1915; -.
DR   DisGeNET; 1915; -.
DR   GeneCards; EEF1A1; -.
DR   HGNC; HGNC:3189; EEF1A1.
DR   HPA; ENSG00000156508; Low tissue specificity.
DR   MIM; 130590; gene.
DR   neXtProt; NX_P68104; -.
DR   OpenTargets; ENSG00000156508; -.
DR   PharmGKB; PA27625; -.
DR   VEuPathDB; HostDB:ENSG00000156508.17; -.
DR   eggNOG; KOG0052; Eukaryota.
DR   GeneTree; ENSGT00950000183029; -.
DR   InParanoid; P68104; -.
DR   OMA; ICAINKM; -.
DR   PhylomeDB; P68104; -.
DR   TreeFam; TF300304; -.
DR   PathwayCommons; P68104; -.
DR   Reactome; R-HSA-156842; Eukaryotic Translation Elongation.
DR   Reactome; R-HSA-156902; Peptide chain elongation.
DR   Reactome; R-HSA-3371511; HSF1 activation.
DR   Reactome; R-HSA-6798695; Neutrophil degranulation.
DR   Reactome; R-HSA-8876725; Protein methylation.
DR   Reactome; R-HSA-9613829; Chaperone Mediated Autophagy.
DR   SignaLink; P68104; -.
DR   SIGNOR; P68104; -.
DR   BioGRID-ORCS; 1915; 706 hits in 942 CRISPR screens.
DR   ChiTaRS; EEF1A1; human.
DR   GeneWiki; Eukaryotic_translation_elongation_factor_1_alpha_1; -.
DR   GenomeRNAi; 1915; -.
DR   Pharos; P68104; Tchem.
DR   PRO; PR:P68104; -.
DR   Proteomes; UP000005640; Chromosome 6.
DR   RNAct; P68104; protein.
DR   Bgee; ENSG00000156508; Expressed in subventricular zone (outer) (primate) and 228 other tissues.
DR   ExpressionAtlas; P68104; baseline and differential.
DR   Genevisible; P68104; HS.
DR   GO; GO:0030864; C:cortical actin cytoskeleton; IDA:UniProtKB.
DR   GO; GO:0005737; C:cytoplasm; IDA:UniProtKB.
DR   GO; GO:0098574; C:cytoplasmic side of lysosomal membrane; NAS:ParkinsonsUK-UCL.
DR   GO; GO:0005829; C:cytosol; IDA:UniProtKB.
DR   GO; GO:0005853; C:eukaryotic translation elongation factor 1 complex; TAS:UniProtKB.
DR   GO; GO:0070062; C:extracellular exosome; HDA:UniProtKB.
DR   GO; GO:0005576; C:extracellular region; TAS:Reactome.
DR   GO; GO:0005615; C:extracellular space; HDA:UniProtKB.
DR   GO; GO:1904813; C:ficolin-1-rich granule lumen; TAS:Reactome.
DR   GO; GO:0016020; C:membrane; HDA:UniProtKB.
DR   GO; GO:0005730; C:nucleolus; IDA:UniProtKB.
DR   GO; GO:0005634; C:nucleus; IDA:UniProtKB.
DR   GO; GO:0005886; C:plasma membrane; IDA:UniProtKB.
DR   GO; GO:0032587; C:ruffle membrane; IDA:UniProtKB.
DR   GO; GO:0034774; C:secretory granule lumen; TAS:Reactome.
DR   GO; GO:0005516; F:calmodulin binding; IEA:Ensembl.
DR   GO; GO:0005525; F:GTP binding; TAS:UniProtKB.
DR   GO; GO:0003924; F:GTPase activity; IBA:GO_Central.
DR   GO; GO:0019900; F:kinase binding; IPI:UniProtKB.
DR   GO; GO:0019901; F:protein kinase binding; IPI:UniProtKB.
DR   GO; GO:0003723; F:RNA binding; HDA:UniProtKB.
DR   GO; GO:0003746; F:translation elongation factor activity; IBA:GO_Central.
DR   GO; GO:0000049; F:tRNA binding; IDA:UniProtKB.
DR   GO; GO:0071364; P:cellular response to epidermal growth factor stimulus; IDA:UniProtKB.
DR   GO; GO:0043312; P:neutrophil degranulation; TAS:Reactome.
DR   GO; GO:1904714; P:regulation of chaperone-mediated autophagy; NAS:ParkinsonsUK-UCL.
DR   GO; GO:1900022; P:regulation of D-erythro-sphingosine kinase activity; IDA:UniProtKB.
DR   GO; GO:0006412; P:translation; IBA:GO_Central.
DR   GO; GO:0006414; P:translational elongation; IBA:GO_Central.
DR   HAMAP; MF_00118_A; EF_Tu_A; 1.
DR   InterPro; IPR004161; EFTu-like_2.
DR   InterPro; IPR031157; G_TR_CS.
DR   InterPro; IPR027417; P-loop_NTPase.
DR   InterPro; IPR000795; T_Tr_GTP-bd_dom.
DR   InterPro; IPR009000; Transl_B-barrel_sf.
DR   InterPro; IPR009001; Transl_elong_EF1A/Init_IF2_C.
DR   InterPro; IPR004539; Transl_elong_EF1A_euk/arc.
DR   InterPro; IPR004160; Transl_elong_EFTu/EF1A_C.
DR   Pfam; PF00009; GTP_EFTU; 1.
DR   Pfam; PF03144; GTP_EFTU_D2; 1.
DR   Pfam; PF03143; GTP_EFTU_D3; 1.
DR   PRINTS; PR00315; ELONGATNFCT.
DR   SUPFAM; SSF50447; SSF50447; 1.
DR   SUPFAM; SSF50465; SSF50465; 1.
DR   SUPFAM; SSF52540; SSF52540; 1.
DR   TIGRFAMs; TIGR00483; EF-1_alpha; 1.
DR   PROSITE; PS00301; G_TR_1; 1.
DR   PROSITE; PS51722; G_TR_2; 1.
PE   1: Evidence at protein level;
KW   3D-structure; Acetylation; Cell membrane; Cytoplasm;
KW   Direct protein sequencing; Elongation factor; GTP-binding; Membrane;
KW   Methylation; Nucleotide-binding; Nucleus; Phosphoprotein;
KW   Protein biosynthesis; Reference proteome; Transcription;
KW   Transcription regulation; Ubl conjugation.
FT   INIT_MET        1
FT                   /note="Removed"
FT                   /evidence="ECO:0000269|PubMed:26545399"
FT   CHAIN           2..462
FT                   /note="Elongation factor 1-alpha 1"
FT                   /id="PRO_0000090885"
FT   DOMAIN          5..242
FT                   /note="tr-type G"
FT   NP_BIND         14..21
FT                   /note="GTP"
FT                   /evidence="ECO:0000250"
FT   NP_BIND         91..95
FT                   /note="GTP"
FT                   /evidence="ECO:0000250"
FT   NP_BIND         153..156
FT                   /note="GTP"
FT                   /evidence="ECO:0000250"
FT   REGION          14..21
FT                   /note="G1"
FT                   /evidence="ECO:0000250"
FT   REGION          70..74
FT                   /note="G2"
FT                   /evidence="ECO:0000250"
FT   REGION          91..94
FT                   /note="G3"
FT                   /evidence="ECO:0000250"
FT   REGION          153..156
FT                   /note="G4"
FT                   /evidence="ECO:0000250"
FT   REGION          194..196
FT                   /note="G5"
FT                   /evidence="ECO:0000250"
FT   MOD_RES         2
FT                   /note="N,N,N-trimethylglycine"
FT                   /evidence="ECO:0000269|PubMed:26545399,
FT                   ECO:0000269|PubMed:30143613"
FT   MOD_RES         36
FT                   /note="N6,N6,N6-trimethyllysine; alternate; by EEF1AKMT4"
FT                   /evidence="ECO:0000269|PubMed:28520920"
FT   MOD_RES         36
FT                   /note="N6,N6-dimethyllysine; alternate; by EEF1AKMT4"
FT                   /evidence="ECO:0000269|PubMed:28520920"
FT   MOD_RES         36
FT                   /note="N6-methyllysine; alternate; by EEF1AKMT4"
FT                   /evidence="ECO:0000269|PubMed:28520920"
FT   MOD_RES         55
FT                   /note="N6,N6-dimethyllysine"
FT                   /evidence="ECO:0000269|PubMed:30143613,
FT                   ECO:0000269|PubMed:30612740, ECO:0000269|Ref.7"
FT   MOD_RES         79
FT                   /note="N6,N6,N6-trimethyllysine; by EEF1AKMT1"
FT                   /evidence="ECO:0000269|PubMed:26545399"
FT   MOD_RES         165
FT                   /note="N6,N6,N6-trimethyllysine; alternate; by EEF1AKMT3"
FT                   /evidence="ECO:0000269|PubMed:28108655"
FT   MOD_RES         165
FT                   /note="N6,N6-dimethyllysine; alternate; by EEF1AKMT3"
FT                   /evidence="ECO:0000269|PubMed:28108655, ECO:0000269|Ref.7"
FT   MOD_RES         165
FT                   /note="N6-acetyllysine; alternate"
FT                   /evidence="ECO:0000250|UniProtKB:P10126"
FT   MOD_RES         165
FT                   /note="N6-methyllysine; alternate; by EEF1AKMT3"
FT                   /evidence="ECO:0000269|PubMed:28108655"
FT   MOD_RES         172
FT                   /note="N6-acetyllysine"
FT                   /evidence="ECO:0000250|UniProtKB:P10126"
FT   MOD_RES         273
FT                   /note="N6-acetyllysine"
FT                   /evidence="ECO:0000250|UniProtKB:P10126"
FT   MOD_RES         300
FT                   /note="Phosphoserine; by TGFBR1"
FT                   /evidence="ECO:0000269|PubMed:20832312"
FT   MOD_RES         301
FT                   /note="5-glutamyl glycerylphosphorylethanolamine"
FT                   /evidence="ECO:0000269|PubMed:2569467"
FT   MOD_RES         318
FT                   /note="N6,N6,N6-trimethyllysine; by EEF1AKMT2"
FT                   /evidence="ECO:0000269|PubMed:25144183"
FT   MOD_RES         374
FT                   /note="5-glutamyl glycerylphosphorylethanolamine"
FT                   /evidence="ECO:0000269|PubMed:2569467"
FT   MOD_RES         392
FT                   /note="N6-acetyllysine; alternate"
FT                   /evidence="ECO:0000250|UniProtKB:P10126"
FT   MOD_RES         392
FT                   /note="N6-succinyllysine; alternate"
FT                   /evidence="ECO:0000250|UniProtKB:P10126"
FT   MOD_RES         432
FT                   /note="Phosphothreonine; by PASK"
FT                   /evidence="ECO:0000269|PubMed:17595531"
FT   MOD_RES         439
FT                   /note="N6-acetyllysine"
FT                   /evidence="ECO:0000250|UniProtKB:P10126"
FT   MUTAGEN         36
FT                   /note="K->R: No effect on methylation by EEF1AKMT2.
FT                   Abolishes EEF1AKMT4-mediated methylation."
FT                   /evidence="ECO:0000269|PubMed:25144183,
FT                   ECO:0000269|PubMed:28520920"
FT   MUTAGEN         55
FT                   /note="K->R: No effect on methylation by EEF1AKMT2.
FT                   Abolishes methylation by EEF1AKNMT."
FT                   /evidence="ECO:0000269|PubMed:25144183,
FT                   ECO:0000269|PubMed:30143613, ECO:0000269|PubMed:30612740"
FT   MUTAGEN         79
FT                   /note="K->R: No effect on methylation by EEF1AKMT2."
FT                   /evidence="ECO:0000269|PubMed:25144183"
FT   MUTAGEN         165
FT                   /note="K->A: Abolishes methylation by EEF1AKMT3."
FT                   /evidence="ECO:0000269|PubMed:28108655"
FT   MUTAGEN         165
FT                   /note="K->R: No effect on methylation by EEF1AKMT2."
FT                   /evidence="ECO:0000269|PubMed:25144183"
FT   MUTAGEN         318
FT                   /note="K->R: Abolishes methylation by EEF1AKMT2."
FT                   /evidence="ECO:0000269|PubMed:25144183"
FT   MUTAGEN         399
FT                   /note="A->V: Resistant to inhibition by plitidepsin. No
FT                   effect on SARS-CoV-2 proteins translation."
FT                   /evidence="ECO:0000269|PubMed:33495306"
FT   MUTAGEN         432
FT                   /note="T->A: Abolishes phosphorylation by PASK."
FT                   /evidence="ECO:0000269|PubMed:17595531"
FT   CONFLICT        83
FT                   /note="S -> A (in Ref. 6; CAA27325)"
FT                   /evidence="ECO:0000305"
FT   CONFLICT        232
FT                   /note="L -> V (in Ref. 3; CAA34756)"
FT                   /evidence="ECO:0000305"
SQ   SEQUENCE   462 AA;  50141 MW;  D465615545AF686A CRC64;
     MGKEKTHINI VVIGHVDSGK STTTGHLIYK CGGIDKRTIE KFEKEAAEMG KGSFKYAWVL
     DKLKAERERG ITIDISLWKF ETSKYYVTII DAPGHRDFIK NMITGTSQAD CAVLIVAAGV
     GEFEAGISKN GQTREHALLA YTLGVKQLIV GVNKMDSTEP PYSQKRYEEI VKEVSTYIKK
     IGYNPDTVAF VPISGWNGDN MLEPSANMPW FKGWKVTRKD GNASGTTLLE ALDCILPPTR
     PTDKPLRLPL QDVYKIGGIG TVPVGRVETG VLKPGMVVTF APVNVTTEVK SVEMHHEALS
     EALPGDNVGF NVKNVSVKDV RRGNVAGDSK NDPPMEAAGF TAQVIILNHP GQISAGYAPV
     LDCHTAHIAC KFAELKEKID RRSGKKLEDG PKFLKSGDAA IVDMVPGKPM CVESFSDYPP
     LGRFAVRDMR QTVAVGVIKA VDKKAAGAGK VTKSAQKAQK AK
```

|  |
| --- |
| **Mascot:** http://www.matrixscience.com/ |
