# Supplementary material for: Chlorpromazine induces cytotoxic autophagy in glioblastoma cells via endoplasmic reticulum stress and unfolded protein response
Source: J Exp Clin Cancer Res. 2021 Nov 5;40:347. doi: 10.1186/s13046-021-02144-w (PMC8569984; doi:10.1186/s13046-021-02144-w)
Supplement: Supplementary file 1 — Additional file 1. [file 13046_2021_2144_MOESM1_ESM.zip › MASCOT IDs/Mascot Search Results_ EF2_2HUMAN.html]

Mascot Search Results: EF2\_HUMAN
 

# MASCOT Search Results

## Protein View: EF2\_HUMAN

### Elongation factor 2 OS=Homo sapiens OX=9606 GN=EEF2 PE=1 SV=4

|  |  |
| --- | --- |
| Database: | SwissProt |
| Score: | 96 |
| Monoisotopic mass (Mr): | 96246 |
| Calculated pI: | 6.41 |
| Taxonomy: | Homo sapiens |

Sequence similarity is available as an NCBI BLAST search of EF2\_HUMAN against nr.

### Search parameters

|  |  |
| --- | --- |
| MS data file: | `ppw_D16_157002891400.txt` |
| Enzyme: | Trypsin: cuts C-term side of KR unless next residue is P. |
| Fixed modifications: | Carbamidomethyl (C) |

### Protein sequence coverage: 3%

Matched peptides shown in ***bold red***.

|  |  |  |  |  |  |
| --- | --- | --- | --- | --- | --- |
| `1` | `MVNFTVDQIR` | `AIMDKKANIR` | `NMSVIAHVDH` | `GKSTLTDSLV` | `CKAGIIASAR` |
| `51` | `AGETRFTDTR` | `KDEQERCITI` | `KSTAISLFYE` | `LSENDLNFIK` | `QSKDGAGFLI` |
| `101` | `NLIDSPGHVD` | `FSSEVTAALR` | `VTDGALVVVD` | `CVSGVCVQTE` | `TVLRQAIAER` |
| `151` | `IKPVLMMNKM` | `DRALLELQLE` | `PEELYQTFQR` | `IVENVNVIIS` | `TYGEGESGPM` |
| `201` | `GNIMIDPVLG` | `TVGFGSGLHG` | `WAFTLKQFAE` | `MYVAKFAAKG` | `EGQLGPAERA` |
| `251` | `KKVEDMMKKL` | `WGDRYFDPAN` | `GKFSKSATSP` | `EGKKLPRTFC` | `QLILDPIFKV` |
| `301` | `FDAIMNFKKE` | `ETAKLIEKLD` | `IKLDSEDKDK` | `EGKPLLKAVM` | `RRWLPAGDAL` |
| `351` | `LQMITIHLPS` | `PVTAQKYRCE` | `LLYEGPPDDE` | `AAMGIKSCDP` | `KGPLMMYISK` |
| `401` | `MVPTSDKGRF` | `YAFGRVFSGL` | `VSTGLKVRIM` | `GPNYTPGKKE` | `DLYLKPIQRT` |
| `451` | `ILMMGRYVEP` | `IEDVPCGNIV` | `GLVGVDQFLV` | `KTGTITTFEH` | `AHNMRVMKFS` |
| `501` | `VSPVVRVAVE` | `AKNPADLPKL` | `VEGLKRLAKS` | `DPMVQCIIEE` | `SGEHIIAGAG` |
| `551` | `ELHLEICLKD` | `LEEDHACIPI` | `KKSDPVVSYR` | `ETVSEESNVL` | `CLSKSPNKHN` |
| `601` | `RLYMKARPFP` | `DGLAEDIDKG` | `EVSARQELKQ` | `RARYLAEKYE` | `WDVAEARKIW` |
| `651` | `CFGPDGTGPN` | `ILTDITKGVQ` | `YLNEIKDSVV` | `AGFQWATKEG` | `ALCEENMRGV` |
| `701` | `RFDVHDVTLH` | `ADAIHRGGGQ` | `IIPTARRCLY` | `ASVLTAQPRL` | `MEPIYLVEIQ` |
| `751` | `CPEQVVGGIY` | `GVLNRKRGHV` | `FEESQVAGTP` | `MFVVKAYLPV` | `NESFGFTADL` |
| `801` | `RSNTGGQAFP` | `QCVFDHWQIL` | `PGDPFDNSSR` | `PSQVVAETRK` | `RKGLKEGIPA` |
| `851` | `LDNFLDKL` |  |  |  |  |

Unformatted sequence string: 858 residues (for pasting into other applications).

|  |  |  |  |
| --- | --- | --- | --- |
| Sort by | residue number | increasing mass | decreasing mass |
| Show | matched peptides only | predicted peptides also |  |

| Query | Start | – | End | Observed | Mr(expt) | Mr(calc) | ppm | M | Score | Expect | Rank | U | Peptide |
| --- | --- | --- | --- | --- | --- | --- | --- | --- | --- | --- | --- | --- | --- |
| 18 | 2 | – | 10 | 1091.6259 | 1090.6186 | 1090.5771 | 38.1 | 0 | 60 | 3.1e-05 | 1Score **> 27** indicates **identity** | U | M.VNFTVDQIR.A |
| 78 | 606 | – | 625 | 2143.1543 | 2142.1470 | 2142.0705 | 35.7 | 1 | 62 | 1.5e-05 | 1Score **> 27** indicates **identity** Score **> 26** indicates **homology** | U | K.ARPFPDGLAEDIDKGEVSAR.Q |

---

```
ID   EF2_HUMAN               Reviewed;         858 AA.
AC   P13639; B2RMP5; D6W618; Q58J86;
DT   01-JAN-1990, integrated into UniProtKB/Swiss-Prot.
DT   23-JAN-2007, sequence version 4.
DT   02-JUN-2021, entry version 229.
DE   RecName: Full=Elongation factor 2;
DE            Short=EF-2;
GN   Name=EEF2; Synonyms=EF2;
OS   Homo sapiens (Human).
OC   Eukaryota; Metazoa; Chordata; Craniata; Vertebrata; Euteleostomi; Mammalia;
OC   Eutheria; Euarchontoglires; Primates; Haplorrhini; Catarrhini; Hominidae;
OC   Homo.
OX   NCBI_TaxID=9606;
RN   [1]
RP   NUCLEOTIDE SEQUENCE [MRNA].
RC   TISSUE=Ovary;
RX   PubMed=2610926; DOI=10.1515/bchm3.1989.370.2.1071;
RA   Rapp G., Klaudiny J., Hagendorff G., Luck M.R., Heinz K.;
RT   "Complete sequence of the coding region of human elongation factor 2 (EF-2)
RT   by enzymatic amplification of cDNA from human ovarian granulosa cells.";
RL   Biol. Chem. Hoppe-Seyler 370:1071-1075(1989).
RN   [2]
RP   NUCLEOTIDE SEQUENCE [MRNA].
RX   PubMed=1596361; DOI=10.1515/bchm3.1992.373.1.201;
RA   Hanes J., Freudenstein J., Rapp G., Scheit K.H.;
RT   "Construction of a plasmid containing the complete coding region of human
RT   elongation factor 2.";
RL   Biol. Chem. Hoppe-Seyler 373:201-204(1992).
RN   [3]
RP   NUCLEOTIDE SEQUENCE [MRNA].
RC   TISSUE=Peripheral blood;
RA   Ustek D., Bektas M., Cakiris A., Oku B., Bermek E.;
RL   Submitted (FEB-2005) to the EMBL/GenBank/DDBJ databases.
RN   [4]
RP   NUCLEOTIDE SEQUENCE [LARGE SCALE GENOMIC DNA].
RA   Mural R.J., Istrail S., Sutton G.G., Florea L., Halpern A.L., Mobarry C.M.,
RA   Lippert R., Walenz B., Shatkay H., Dew I., Miller J.R., Flanigan M.J.,
RA   Edwards N.J., Bolanos R., Fasulo D., Halldorsson B.V., Hannenhalli S.,
RA   Turner R., Yooseph S., Lu F., Nusskern D.R., Shue B.C., Zheng X.H.,
RA   Zhong F., Delcher A.L., Huson D.H., Kravitz S.A., Mouchard L., Reinert K.,
RA   Remington K.A., Clark A.G., Waterman M.S., Eichler E.E., Adams M.D.,
RA   Hunkapiller M.W., Myers E.W., Venter J.C.;
RL   Submitted (SEP-2005) to the EMBL/GenBank/DDBJ databases.
RN   [5]
RP   NUCLEOTIDE SEQUENCE [LARGE SCALE MRNA].
RC   TISSUE=Brain;
RX   PubMed=15489334; DOI=10.1101/gr.2596504;
RG   The MGC Project Team;
RT   "The status, quality, and expansion of the NIH full-length cDNA project:
RT   the Mammalian Gene Collection (MGC).";
RL   Genome Res. 14:2121-2127(2004).
RN   [6]
RP   NUCLEOTIDE SEQUENCE [MRNA] OF 501-858.
RX   PubMed=2840927; DOI=10.1515/bchm3.1988.369.1.247;
RA   Rapp G., Mucha J., Einspanier R., Luck M., Scheit K.H.;
RT   "Cloning and sequence analysis of a cDNA from human ovarian granulosa cells
RT   encoding the C-terminal part of human elongation factor 2.";
RL   Biol. Chem. Hoppe-Seyler 369:247-250(1988).
RN   [7]
RP   PROTEIN SEQUENCE OF 796-801, AND IDENTIFICATION BY MASS SPECTROMETRY.
RC   TISSUE=Brain, and Cajal-Retzius cell;
RA   Lubec G., Vishwanath V.;
RL   Submitted (MAR-2007) to UniProtKB.
RN   [8]
RP   CLEAVAGE OF INITIATOR METHIONINE.
RA   Bienvenut W.V.;
RL   Submitted (AUG-2001) to UniProtKB.
RN   [9]
RP   IDENTIFICATION BY MASS SPECTROMETRY.
RC   TISSUE=Lymphoblast;
RX   PubMed=14654843; DOI=10.1038/nature02166;
RA   Andersen J.S., Wilkinson C.J., Mayor T., Mortensen P., Nigg E.A., Mann M.;
RT   "Proteomic characterization of the human centrosome by protein correlation
RT   profiling.";
RL   Nature 426:570-574(2003).
RN   [10]
RP   ISGYLATION.
RX   PubMed=16139798; DOI=10.1016/j.bbrc.2005.08.132;
RA   Giannakopoulos N.V., Luo J.K., Papov V., Zou W., Lenschow D.J.,
RA   Jacobs B.S., Borden E.C., Li J., Virgin H.W., Zhang D.E.;
RT   "Proteomic identification of proteins conjugated to ISG15 in mouse and
RT   human cells.";
RL   Biochem. Biophys. Res. Commun. 336:496-506(2005).
RN   [11]
RP   IDENTIFICATION BY MASS SPECTROMETRY [LARGE SCALE ANALYSIS].
RC   TISSUE=Cervix carcinoma;
RX   PubMed=17081983; DOI=10.1016/j.cell.2006.09.026;
RA   Olsen J.V., Blagoev B., Gnad F., Macek B., Kumar C., Mortensen P., Mann M.;
RT   "Global, in vivo, and site-specific phosphorylation dynamics in signaling
RT   networks.";
RL   Cell 127:635-648(2006).
RN   [12]
RP   MUTAGENESIS OF HIS-715, AND DIPHTHAMIDE AT HIS-715.
RX   PubMed=16901746; DOI=10.1016/j.mrgentox.2006.06.027;
RA   Ivankovic M., Rubelj I., Matulic M., Reich E., Brdar B.;
RT   "Site-specific mutagenesis of the histidine precursor of diphthamide in the
RT   human elongation factor-2 gene confers resistance to diphtheria toxin.";
RL   Mutat. Res. 609:34-42(2006).
RN   [13]
RP   PHOSPHORYLATION [LARGE SCALE ANALYSIS] AT THR-435 AND SER-502, AND
RP   IDENTIFICATION BY MASS SPECTROMETRY [LARGE SCALE ANALYSIS].
RC   TISSUE=Cervix carcinoma;
RX   PubMed=18669648; DOI=10.1073/pnas.0805139105;
RA   Dephoure N., Zhou C., Villen J., Beausoleil S.A., Bakalarski C.E.,
RA   Elledge S.J., Gygi S.P.;
RT   "A quantitative atlas of mitotic phosphorylation.";
RL   Proc. Natl. Acad. Sci. U.S.A. 105:10762-10767(2008).
RN   [14]
RP   IDENTIFICATION BY MASS SPECTROMETRY [LARGE SCALE ANALYSIS].
RX   PubMed=19413330; DOI=10.1021/ac9004309;
RA   Gauci S., Helbig A.O., Slijper M., Krijgsveld J., Heck A.J., Mohammed S.;
RT   "Lys-N and trypsin cover complementary parts of the phosphoproteome in a
RT   refined SCX-based approach.";
RL   Anal. Chem. 81:4493-4501(2009).
RN   [15]
RP   IDENTIFICATION IN THE SURF COMPLEX.
RX   PubMed=19417104; DOI=10.1101/gad.1767209;
RA   Yamashita A., Izumi N., Kashima I., Ohnishi T., Saari B., Katsuhata Y.,
RA   Muramatsu R., Morita T., Iwamatsu A., Hachiya T., Kurata R., Hirano H.,
RA   Anderson P., Ohno S.;
RT   "SMG-8 and SMG-9, two novel subunits of the SMG-1 complex, regulate
RT   remodeling of the mRNA surveillance complex during nonsense-mediated mRNA
RT   decay.";
RL   Genes Dev. 23:1091-1105(2009).
RN   [16]
RP   ACETYLATION [LARGE SCALE ANALYSIS] AT LYS-235; LYS-239; LYS-272; LYS-275
RP   AND LYS-445, AND IDENTIFICATION BY MASS SPECTROMETRY [LARGE SCALE
RP   ANALYSIS].
RX   PubMed=19608861; DOI=10.1126/science.1175371;
RA   Choudhary C., Kumar C., Gnad F., Nielsen M.L., Rehman M., Walther T.C.,
RA   Olsen J.V., Mann M.;
RT   "Lysine acetylation targets protein complexes and co-regulates major
RT   cellular functions.";
RL   Science 325:834-840(2009).
RN   [17]
RP   PHOSPHORYLATION [LARGE SCALE ANALYSIS] AT THR-57; THR-59 AND THR-435, AND
RP   IDENTIFICATION BY MASS SPECTROMETRY [LARGE SCALE ANALYSIS].
RC   TISSUE=Cervix carcinoma;
RX   PubMed=20068231; DOI=10.1126/scisignal.2000475;
RA   Olsen J.V., Vermeulen M., Santamaria A., Kumar C., Miller M.L.,
RA   Jensen L.J., Gnad F., Cox J., Jensen T.S., Nigg E.A., Brunak S., Mann M.;
RT   "Quantitative phosphoproteomics reveals widespread full phosphorylation
RT   site occupancy during mitosis.";
RL   Sci. Signal. 3:RA3-RA3(2010).
RN   [18]
RP   IDENTIFICATION BY MASS SPECTROMETRY [LARGE SCALE ANALYSIS].
RX   PubMed=21269460; DOI=10.1186/1752-0509-5-17;
RA   Burkard T.R., Planyavsky M., Kaupe I., Breitwieser F.P., Buerckstuemmer T.,
RA   Bennett K.L., Superti-Furga G., Colinge J.;
RT   "Initial characterization of the human central proteome.";
RL   BMC Syst. Biol. 5:17-17(2011).
RN   [19]
RP   PHOSPHORYLATION [LARGE SCALE ANALYSIS] AT THR-54; THR-57 AND THR-59, AND
RP   IDENTIFICATION BY MASS SPECTROMETRY [LARGE SCALE ANALYSIS].
RX   PubMed=21406692; DOI=10.1126/scisignal.2001570;
RA   Rigbolt K.T., Prokhorova T.A., Akimov V., Henningsen J., Johansen P.T.,
RA   Kratchmarova I., Kassem M., Mann M., Olsen J.V., Blagoev B.;
RT   "System-wide temporal characterization of the proteome and phosphoproteome
RT   of human embryonic stem cell differentiation.";
RL   Sci. Signal. 4:RS3-RS3(2011).
RN   [20]
RP   CLEAVAGE OF INITIATOR METHIONINE [LARGE SCALE ANALYSIS], AND IDENTIFICATION
RP   BY MASS SPECTROMETRY [LARGE SCALE ANALYSIS].
RX   PubMed=22223895; DOI=10.1074/mcp.m111.015131;
RA   Bienvenut W.V., Sumpton D., Martinez A., Lilla S., Espagne C., Meinnel T.,
RA   Giglione C.;
RT   "Comparative large-scale characterisation of plant vs. mammal proteins
RT   reveals similar and idiosyncratic N-alpha acetylation features.";
RL   Mol. Cell. Proteomics 11:M111.015131-M111.015131(2012).
RN   [21]
RP   IDENTIFICATION BY MASS SPECTROMETRY [LARGE SCALE ANALYSIS].
RX   PubMed=22814378; DOI=10.1073/pnas.1210303109;
RA   Van Damme P., Lasa M., Polevoda B., Gazquez C., Elosegui-Artola A.,
RA   Kim D.S., De Juan-Pardo E., Demeyer K., Hole K., Larrea E., Timmerman E.,
RA   Prieto J., Arnesen T., Sherman F., Gevaert K., Aldabe R.;
RT   "N-terminal acetylome analyses and functional insights of the N-terminal
RT   acetyltransferase NatB.";
RL   Proc. Natl. Acad. Sci. U.S.A. 109:12449-12454(2012).
RN   [22]
RP   PHOSPHORYLATION [LARGE SCALE ANALYSIS] AT THR-57; THR-59; THR-435; SER-502
RP   AND SER-595, AND IDENTIFICATION BY MASS SPECTROMETRY [LARGE SCALE
RP   ANALYSIS].
RC   TISSUE=Cervix carcinoma, and Erythroleukemia;
RX   PubMed=23186163; DOI=10.1021/pr300630k;
RA   Zhou H., Di Palma S., Preisinger C., Peng M., Polat A.N., Heck A.J.,
RA   Mohammed S.;
RT   "Toward a comprehensive characterization of a human cancer cell
RT   phosphoproteome.";
RL   J. Proteome Res. 12:260-271(2013).
RN   [23]
RP   PHOSPHORYLATION AT THR-57 AND SER-595, AND MUTAGENESIS OF SER-595 AND
RP   HIS-599.
RX   PubMed=23184662; DOI=10.1128/mcb.01270-12;
RA   Hizli A.A., Chi Y., Swanger J., Carter J.H., Liao Y., Welcker M.,
RA   Ryazanov A.G., Clurman B.E.;
RT   "Phosphorylation of eukaryotic elongation factor 2 (eEF2) by cyclin A-
RT   cyclin-dependent kinase 2 regulates its inhibition by eEF2 kinase.";
RL   Mol. Cell. Biol. 33:596-604(2013).
RN   [24]
RP   PHOSPHORYLATION AT TYR-265 AND TYR-373 BY CSK, SUMOYLATION AT LYS-322 AND
RP   LYS-529, PROTEOLYTIC PROCESSING, AND SUBCELLULAR LOCATION.
RX   PubMed=24648518; DOI=10.1074/jbc.m113.546481;
RA   Yao Q., Liu B.Q., Li H., McGarrigle D., Xing B.W., Zhou M.T., Wang Z.,
RA   Zhang J.J., Huang X.Y., Guo L.;
RT   "C-terminal Src kinase (Csk)-mediated phosphorylation of eukaryotic
RT   elongation factor 2 (eEF2) promotes proteolytic cleavage and nuclear
RT   translocation of eEF2.";
RL   J. Biol. Chem. 289:12666-12678(2014).
RN   [25]
RP   METHYLATION AT LYS-525.
RX   PubMed=25231979; DOI=10.1074/jbc.m114.601658;
RA   Davydova E., Ho A.Y., Malecki J., Moen A., Enserink J.M., Jakobsson M.E.,
RA   Loenarz C., Falnes P.O.;
RT   "Identification and characterization of a novel evolutionarily conserved
RT   lysine-specific methyltransferase targeting eukaryotic translation
RT   elongation factor 2 (eEF2).";
RL   J. Biol. Chem. 289:30499-30510(2014).
RN   [26]
RP   IDENTIFICATION BY MASS SPECTROMETRY [LARGE SCALE ANALYSIS].
RC   TISSUE=Liver;
RX   PubMed=24275569; DOI=10.1016/j.jprot.2013.11.014;
RA   Bian Y., Song C., Cheng K., Dong M., Wang F., Huang J., Sun D., Wang L.,
RA   Ye M., Zou H.;
RT   "An enzyme assisted RP-RPLC approach for in-depth analysis of human liver
RT   phosphoproteome.";
RL   J. Proteomics 96:253-262(2014).
RN   [27]
RP   INTERACTION WITH RBPMS2, AND SUBCELLULAR LOCATION.
RX   PubMed=25064856; DOI=10.1093/nar/gku692;
RA   Sagnol S., Yang Y., Bessin Y., Allemand F., Hapkova I., Notarnicola C.,
RA   Guichou J.F., Faure S., Labesse G., de Santa Barbara P.;
RT   "Homodimerization of RBPMS2 through a new RRM-interaction motif is
RT   necessary to control smooth muscle plasticity.";
RL   Nucleic Acids Res. 42:10173-10184(2014).
RN   [28]
RP   SUMOYLATION [LARGE SCALE ANALYSIS] AT LYS-239, AND IDENTIFICATION BY MASS
RP   SPECTROMETRY [LARGE SCALE ANALYSIS].
RX   PubMed=25114211; DOI=10.1073/pnas.1413825111;
RA   Impens F., Radoshevich L., Cossart P., Ribet D.;
RT   "Mapping of SUMO sites and analysis of SUMOylation changes induced by
RT   external stimuli.";
RL   Proc. Natl. Acad. Sci. U.S.A. 111:12432-12437(2014).
RN   [29]
RP   CLEAVAGE OF INITIATOR METHIONINE [LARGE SCALE ANALYSIS], AND IDENTIFICATION
RP   BY MASS SPECTROMETRY [LARGE SCALE ANALYSIS].
RX   PubMed=25944712; DOI=10.1002/pmic.201400617;
RA   Vaca Jacome A.S., Rabilloud T., Schaeffer-Reiss C., Rompais M., Ayoub D.,
RA   Lane L., Bairoch A., Van Dorsselaer A., Carapito C.;
RT   "N-terminome analysis of the human mitochondrial proteome.";
RL   Proteomics 15:2519-2524(2015).
RN   [30]
RP   STRUCTURE BY ELECTRON MICROSCOPY (5.0 ANGSTROMS) IN COMPLEX WITH 80S
RP   RIBOSOME.
RX   PubMed=23636399; DOI=10.1038/nature12104;
RA   Anger A.M., Armache J.P., Berninghausen O., Habeck M., Subklewe M.,
RA   Wilson D.N., Beckmann R.;
RT   "Structures of the human and Drosophila 80S ribosome.";
RL   Nature 497:80-85(2013).
RN   [31]
RP   VARIANT SCA26 HIS-596, AND CHARACTERIZATION OF VARIANT SCA26 HIS-596.
RX   PubMed=23001565; DOI=10.1093/hmg/dds392;
RA   Hekman K.E., Yu G.Y., Brown C.D., Zhu H., Du X., Gervin K., Undlien D.E.,
RA   Peterson A., Stevanin G., Clark H.B., Pulst S.M., Bird T.D., White K.P.,
RA   Gomez C.M.;
RT   "A conserved eEF2 coding variant in SCA26 leads to loss of translational
RT   fidelity and increased susceptibility to proteostatic insult.";
RL   Hum. Mol. Genet. 21:5472-5483(2012).
CC   -!- FUNCTION: Catalyzes the GTP-dependent ribosomal translocation step
CC       during translation elongation. During this step, the ribosome changes
CC       from the pre-translocational (PRE) to the post-translocational (POST)
CC       state as the newly formed A-site-bound peptidyl-tRNA and P-site-bound
CC       deacylated tRNA move to the P and E sites, respectively. Catalyzes the
CC       coordinated movement of the two tRNA molecules, the mRNA and
CC       conformational changes in the ribosome.
CC   -!- SUBUNIT: Component of the mRNA surveillance SURF complex, at least
CC       composed of ERF1, ERF3 (ERF3A or ERF3B), EEF2, UPF1/RENT1, SMG1, SMG8
CC       and SMG9 (PubMed:19417104). Interacts with RBPMS2 (PubMed:25064856).
CC       {ECO:0000269|PubMed:19417104, ECO:0000269|PubMed:25064856}.
CC   -!- INTERACTION:
CC       P13639; Q99714: HSD17B10; NbExp=3; IntAct=EBI-352560, EBI-79964;
CC       P13639; P42858: HTT; NbExp=3; IntAct=EBI-352560, EBI-466029;
CC   -!- SUBCELLULAR LOCATION: Cytoplasm {ECO:0000269|PubMed:25064856}. Nucleus
CC       {ECO:0000269|PubMed:24648518}. Note=Phosphorylation by CSK promotes
CC       cleavage and SUMOylation-dependent nuclear translocation of the C-
CC       terminal cleavage product. {ECO:0000269|PubMed:24648518}.
CC   -!- PTM: Phosphorylation by EF-2 kinase completely inactivates EF-2; it
CC       requires prior phosphorylation by CDK2 at Ser-595 during mitotic
CC       prometaphase. Phosphorylation by CSK promotes SUMOylation, proteolytic
CC       cleavage, and nuclear translocation if the C-terminal fragment.
CC       {ECO:0000269|PubMed:23184662, ECO:0000269|PubMed:24648518}.
CC   -!- PTM: Diphthamide is 2-[3-carboxyamido-3-(trimethyl-
CC       ammonio)propyl]histidine (By similarity).
CC       {ECO:0000250|UniProtKB:P05197}.
CC   -!- PTM: (Microbial infection) Diphthamide can be ADP-ribosylated by
CC       diphtheria toxin and by Pseudomonas exotoxin A, thus arresting protein
CC       synthesis. {ECO:0000269|PubMed:16901746}.
CC   -!- PTM: ISGylated. {ECO:0000269|PubMed:16139798}.
CC   -!- PTM: Proteolytically processed at two sites following phosphorylation
CC       by CSK. {ECO:0000269|PubMed:24648518}.
CC   -!- PTM: SUMOylated following phosphorylation by CSK, promotes proteolytic
CC       cleavage. {ECO:0000269|PubMed:24648518}.
CC   -!- DISEASE: Spinocerebellar ataxia 26 (SCA26) [MIM:609306]: A form of
CC       spinocerebellar ataxia, a clinically and genetically heterogeneous
CC       group of cerebellar disorders. Patients show progressive incoordination
CC       of gait and often poor coordination of hands, speech and eye movements,
CC       due to degeneration of the cerebellum with variable involvement of the
CC       brainstem and spinal cord. {ECO:0000269|PubMed:23001565}. Note=The
CC       disease is caused by variants affecting the gene represented in this
CC       entry.
CC   -!- SIMILARITY: Belongs to the TRAFAC class translation factor GTPase
CC       superfamily. Classic translation factor GTPase family. EF-G/EF-2
CC       subfamily. {ECO:0000255|PROSITE-ProRule:PRU01059}.
CC   ---------------------------------------------------------------------------
CC   Copyrighted by the UniProt Consortium, see https://www.uniprot.org/terms
CC   Distributed under the Creative Commons Attribution (CC BY 4.0) License
CC   ---------------------------------------------------------------------------
DR   EMBL; X51466; CAA35829.1; -; mRNA.
DR   EMBL; Z11692; CAA77750.1; -; mRNA.
DR   EMBL; AY942181; AAX34409.1; -; mRNA.
DR   EMBL; CH471139; EAW69274.1; -; Genomic_DNA.
DR   EMBL; CH471139; EAW69275.1; -; Genomic_DNA.
DR   EMBL; BC126259; AAI26260.1; -; mRNA.
DR   EMBL; BC136313; AAI36314.1; -; mRNA.
DR   EMBL; M19997; AAA50388.1; -; mRNA.
DR   CCDS; CCDS12117.1; -.
DR   PIR; S18294; EFHU2.
DR   RefSeq; NP_001952.1; NM_001961.3.
DR   PDB; 4V6X; EM; 5.00 A; Az=1-858.
DR   PDB; 6D9J; EM; 3.20 A; 9=3-858.
DR   PDB; 6Z6M; EM; 3.10 A; CB=1-858.
DR   PDB; 6Z6N; EM; 2.90 A; CB=1-858.
DR   PDBsum; 4V6X; -.
DR   PDBsum; 6D9J; -.
DR   PDBsum; 6Z6M; -.
DR   PDBsum; 6Z6N; -.
DR   SMR; P13639; -.
DR   BioGRID; 108258; 332.
DR   CORUM; P13639; -.
DR   IntAct; P13639; 115.
DR   MINT; P13639; -.
DR   STRING; 9606.ENSP00000307940; -.
DR   ChEMBL; CHEMBL1795108; -.
DR   DrugBank; DB02059; Adenosine-5-Diphosphoribose.
DR   DrugBank; DB03223; Diphthamide.
DR   DrugBank; DB11823; Esketamine.
DR   DrugBank; DB04315; Guanosine-5'-Diphosphate.
DR   DrugBank; DB12688; Moxetumomab Pasudotox.
DR   DrugBank; DB08348; N~2~,N~2~-DIMETHYL-N~1~-(6-OXO-5,6-DIHYDROPHENANTHRIDIN-2-YL)GLYCINAMIDE.
DR   MoonProt; P13639; -.
DR   iPTMnet; P13639; -.
DR   MetOSite; P13639; -.
DR   PhosphoSitePlus; P13639; -.
DR   SwissPalm; P13639; -.
DR   BioMuta; EEF2; -.
DR   DMDM; 119172; -.
DR   REPRODUCTION-2DPAGE; IPI00186290; -.
DR   EPD; P13639; -.
DR   jPOST; P13639; -.
DR   MassIVE; P13639; -.
DR   MaxQB; P13639; -.
DR   PaxDb; P13639; -.
DR   PeptideAtlas; P13639; -.
DR   PRIDE; P13639; -.
DR   ProteomicsDB; 52947; -.
DR   Antibodypedia; 23430; 545 antibodies.
DR   DNASU; 1938; -.
DR   Ensembl; ENST00000309311; ENSP00000307940; ENSG00000167658.
DR   GeneID; 1938; -.
DR   KEGG; hsa:1938; -.
DR   UCSC; uc002lze.4; human.
DR   CTD; 1938; -.
DR   DisGeNET; 1938; -.
DR   GeneCards; EEF2; -.
DR   HGNC; HGNC:3214; EEF2.
DR   HPA; ENSG00000167658; Low tissue specificity.
DR   MalaCards; EEF2; -.
DR   MIM; 130610; gene.
DR   MIM; 609306; phenotype.
DR   neXtProt; NX_P13639; -.
DR   OpenTargets; ENSG00000167658; -.
DR   Orphanet; 101112; Spinocerebellar ataxia type 26.
DR   PharmGKB; PA27650; -.
DR   VEuPathDB; HostDB:ENSG00000167658.15; -.
DR   eggNOG; KOG0469; Eukaryota.
DR   GeneTree; ENSGT00940000154662; -.
DR   HOGENOM; CLU_002794_11_1_1; -.
DR   InParanoid; P13639; -.
DR   OMA; GVMTQTE; -.
DR   OrthoDB; 140796at2759; -.
DR   PhylomeDB; P13639; -.
DR   TreeFam; TF300575; -.
DR   BRENDA; 3.6.5.3; 2681.
DR   PathwayCommons; P13639; -.
DR   Reactome; R-HSA-156902; Peptide chain elongation.
DR   Reactome; R-HSA-5336415; Uptake and function of diphtheria toxin.
DR   Reactome; R-HSA-5358493; Synthesis of diphthamide-EEF2.
DR   Reactome; R-HSA-6798695; Neutrophil degranulation.
DR   Reactome; R-HSA-8876725; Protein methylation.
DR   SIGNOR; P13639; -.
DR   BioGRID-ORCS; 1938; 813 hits in 975 CRISPR screens.
DR   ChiTaRS; EEF2; human.
DR   GeneWiki; EEF2; -.
DR   GenomeRNAi; 1938; -.
DR   Pharos; P13639; Tchem.
DR   PRO; PR:P13639; -.
DR   Proteomes; UP000005640; Chromosome 19.
DR   RNAct; P13639; protein.
DR   Bgee; ENSG00000167658; Expressed in testis and 259 other tissues.
DR   ExpressionAtlas; P13639; baseline and differential.
DR   Genevisible; P13639; HS.
DR   GO; GO:0016235; C:aggresome; IDA:HPA.
DR   GO; GO:0005737; C:cytoplasm; IDA:UniProtKB.
DR   GO; GO:0005829; C:cytosol; IDA:HPA.
DR   GO; GO:0070062; C:extracellular exosome; HDA:UniProtKB.
DR   GO; GO:0005576; C:extracellular region; TAS:Reactome.
DR   GO; GO:1904813; C:ficolin-1-rich granule lumen; TAS:Reactome.
DR   GO; GO:0016020; C:membrane; HDA:UniProtKB.
DR   GO; GO:0045121; C:membrane raft; IEA:Ensembl.
DR   GO; GO:0005634; C:nucleus; HDA:UniProtKB.
DR   GO; GO:0005886; C:plasma membrane; IDA:HPA.
DR   GO; GO:0042788; C:polysomal ribosome; IEA:Ensembl.
DR   GO; GO:1990904; C:ribonucleoprotein complex; IDA:MGI.
DR   GO; GO:0034774; C:secretory granule lumen; TAS:Reactome.
DR   GO; GO:0045202; C:synapse; IEA:Ensembl.
DR   GO; GO:0008097; F:5S rRNA binding; IEA:Ensembl.
DR   GO; GO:0051015; F:actin filament binding; IEA:Ensembl.
DR   GO; GO:0045296; F:cadherin binding; HDA:BHF-UCL.
DR   GO; GO:0005525; F:GTP binding; IEA:UniProtKB-KW.
DR   GO; GO:0003924; F:GTPase activity; IBA:GO_Central.
DR   GO; GO:0002039; F:p53 binding; IEA:Ensembl.
DR   GO; GO:0019901; F:protein kinase binding; IPI:UniProtKB.
DR   GO; GO:0043022; F:ribosome binding; IDA:FlyBase.
DR   GO; GO:0003723; F:RNA binding; HDA:UniProtKB.
DR   GO; GO:0003746; F:translation elongation factor activity; IBA:GO_Central.
DR   GO; GO:0007568; P:aging; IEA:Ensembl.
DR   GO; GO:1990416; P:cellular response to brain-derived neurotrophic factor stimulus; IEA:Ensembl.
DR   GO; GO:0014009; P:glial cell proliferation; IEA:Ensembl.
DR   GO; GO:0002244; P:hematopoietic progenitor cell differentiation; IEA:Ensembl.
DR   GO; GO:0043312; P:neutrophil degranulation; TAS:Reactome.
DR   GO; GO:2000767; P:positive regulation of cytoplasmic translation; IEA:Ensembl.
DR   GO; GO:0045727; P:positive regulation of translation; IMP:UniProtKB.
DR   GO; GO:0042493; P:response to drug; IEA:Ensembl.
DR   GO; GO:0034976; P:response to endoplasmic reticulum stress; IEA:Ensembl.
DR   GO; GO:0032355; P:response to estradiol; IEA:Ensembl.
DR   GO; GO:0045471; P:response to ethanol; IEA:Ensembl.
DR   GO; GO:0051593; P:response to folic acid; IEA:Ensembl.
DR   GO; GO:0042542; P:response to hydrogen peroxide; IEA:Ensembl.
DR   GO; GO:0002931; P:response to ischemia; IEA:Ensembl.
DR   GO; GO:0035914; P:skeletal muscle cell differentiation; IEA:Ensembl.
DR   GO; GO:0003009; P:skeletal muscle contraction; IEA:Ensembl.
DR   GO; GO:0006414; P:translational elongation; IBA:GO_Central.
DR   Gene3D; 3.30.230.10; -; 1.
DR   InterPro; IPR041095; EFG_II.
DR   InterPro; IPR035647; EFG_III/V.
DR   InterPro; IPR000640; EFG_V-like.
DR   InterPro; IPR004161; EFTu-like_2.
DR   InterPro; IPR031157; G_TR_CS.
DR   InterPro; IPR027417; P-loop_NTPase.
DR   InterPro; IPR020568; Ribosomal_S5_D2-typ_fold.
DR   InterPro; IPR014721; Ribosomal_S5_D2-typ_fold_subgr.
DR   InterPro; IPR005225; Small_GTP-bd_dom.
DR   InterPro; IPR000795; T_Tr_GTP-bd_dom.
DR   InterPro; IPR009000; Transl_B-barrel_sf.
DR   InterPro; IPR005517; Transl_elong_EFG/EF2_IV.
DR   Pfam; PF00679; EFG_C; 1.
DR   Pfam; PF14492; EFG_III; 1.
DR   Pfam; PF03764; EFG_IV; 1.
DR   Pfam; PF00009; GTP_EFTU; 1.
DR   Pfam; PF03144; GTP_EFTU_D2; 1.
DR   PRINTS; PR00315; ELONGATNFCT.
DR   SMART; SM00838; EFG_C; 1.
DR   SMART; SM00889; EFG_IV; 1.
DR   SUPFAM; SSF50447; SSF50447; 1.
DR   SUPFAM; SSF52540; SSF52540; 1.
DR   SUPFAM; SSF54211; SSF54211; 1.
DR   SUPFAM; SSF54980; SSF54980; 2.
DR   TIGRFAMs; TIGR00231; small_GTP; 1.
DR   PROSITE; PS00301; G_TR_1; 1.
DR   PROSITE; PS51722; G_TR_2; 1.
PE   1: Evidence at protein level;
KW   3D-structure; Acetylation; ADP-ribosylation; Cytoplasm;
KW   Direct protein sequencing; Disease variant; Elongation factor; GTP-binding;
KW   Isopeptide bond; Methylation; Neurodegeneration; Nucleotide-binding;
KW   Nucleus; Phosphoprotein; Protein biosynthesis; Reference proteome;
KW   Spinocerebellar ataxia; Ubl conjugation.
FT   INIT_MET        1
FT                   /note="Removed"
FT                   /evidence="ECO:0000269|Ref.8, ECO:0007744|PubMed:22223895,
FT                   ECO:0007744|PubMed:25944712"
FT   CHAIN           2..858
FT                   /note="Elongation factor 2"
FT                   /id="PRO_0000091000"
FT   DOMAIN          17..362
FT                   /note="tr-type G"
FT                   /evidence="ECO:0000255|PROSITE-ProRule:PRU01059"
FT   NP_BIND         26..33
FT                   /note="GTP"
FT                   /evidence="ECO:0000250"
FT   NP_BIND         104..108
FT                   /note="GTP"
FT                   /evidence="ECO:0000250"
FT   NP_BIND         158..161
FT                   /note="GTP"
FT                   /evidence="ECO:0000250"
FT   SITE            586..587
FT                   /note="Cleavage"
FT                   /evidence="ECO:0000269|PubMed:24648518"
FT   SITE            605..606
FT                   /note="Cleavage"
FT                   /evidence="ECO:0000269|PubMed:24648518"
FT   MOD_RES         54
FT                   /note="Phosphothreonine"
FT                   /evidence="ECO:0007744|PubMed:21406692"
FT   MOD_RES         57
FT                   /note="Phosphothreonine; by EEF2K"
FT                   /evidence="ECO:0000269|PubMed:23184662,
FT                   ECO:0007744|PubMed:20068231, ECO:0007744|PubMed:21406692,
FT                   ECO:0007744|PubMed:23186163"
FT   MOD_RES         59
FT                   /note="Phosphothreonine"
FT                   /evidence="ECO:0007744|PubMed:20068231,
FT                   ECO:0007744|PubMed:21406692, ECO:0007744|PubMed:23186163"
FT   MOD_RES         152
FT                   /note="N6-succinyllysine"
FT                   /evidence="ECO:0000250|UniProtKB:P58252"
FT   MOD_RES         235
FT                   /note="N6-acetyllysine"
FT                   /evidence="ECO:0007744|PubMed:19608861"
FT   MOD_RES         239
FT                   /note="N6-acetyllysine; alternate"
FT                   /evidence="ECO:0007744|PubMed:19608861"
FT   MOD_RES         265
FT                   /note="Phosphotyrosine; by CSK"
FT                   /evidence="ECO:0000269|PubMed:24648518"
FT   MOD_RES         272
FT                   /note="N6-acetyllysine; alternate"
FT                   /evidence="ECO:0007744|PubMed:19608861"
FT   MOD_RES         272
FT                   /note="N6-succinyllysine; alternate"
FT                   /evidence="ECO:0000250|UniProtKB:P58252"
FT   MOD_RES         275
FT                   /note="N6-acetyllysine"
FT                   /evidence="ECO:0007744|PubMed:19608861"
FT   MOD_RES         325
FT                   /note="Phosphoserine"
FT                   /evidence="ECO:0000250|UniProtKB:P05197"
FT   MOD_RES         373
FT                   /note="Phosphotyrosine; by CSK"
FT                   /evidence="ECO:0000269|PubMed:24648518"
FT   MOD_RES         435
FT                   /note="Phosphothreonine"
FT                   /evidence="ECO:0007744|PubMed:18669648,
FT                   ECO:0007744|PubMed:20068231, ECO:0007744|PubMed:23186163"
FT   MOD_RES         439
FT                   /note="N6-acetyllysine"
FT                   /evidence="ECO:0000250|UniProtKB:P58252"
FT   MOD_RES         445
FT                   /note="N6-acetyllysine"
FT                   /evidence="ECO:0007744|PubMed:19608861"
FT   MOD_RES         502
FT                   /note="Phosphoserine"
FT                   /evidence="ECO:0007744|PubMed:18669648,
FT                   ECO:0007744|PubMed:23186163"
FT   MOD_RES         525
FT                   /note="N6,N6,N6-trimethyllysine; by EEF2KMT"
FT                   /evidence="ECO:0000269|PubMed:25231979"
FT   MOD_RES         572
FT                   /note="N6-succinyllysine"
FT                   /evidence="ECO:0000250|UniProtKB:P58252"
FT   MOD_RES         595
FT                   /note="Phosphoserine; by CDK2"
FT                   /evidence="ECO:0000269|PubMed:23184662,
FT                   ECO:0007744|PubMed:23186163"
FT   MOD_RES         619
FT                   /note="N6-acetyllysine"
FT                   /evidence="ECO:0000250|UniProtKB:P58252"
FT   MOD_RES         715
FT                   /note="(Microbial infection) ADP-ribosyldiphthamide"
FT                   /evidence="ECO:0000269|PubMed:16901746"
FT   MOD_RES         715
FT                   /note="Diphthamide"
FT                   /evidence="ECO:0000269|PubMed:16901746"
FT   CROSSLNK        239
FT                   /note="Glycyl lysine isopeptide (Lys-Gly) (interchain with
FT                   G-Cter in SUMO1); alternate"
FT                   /evidence="ECO:0007744|PubMed:25114211"
FT   CROSSLNK        322
FT                   /note="Glycyl lysine isopeptide (Lys-Gly) (interchain with
FT                   G-Cter in SUMO)"
FT                   /evidence="ECO:0000269|PubMed:24648518"
FT   CROSSLNK        529
FT                   /note="Glycyl lysine isopeptide (Lys-Gly) (interchain with
FT                   G-Cter in SUMO)"
FT                   /evidence="ECO:0000269|PubMed:24648518"
FT   VARIANT         596
FT                   /note="P -> H (in SCA26; compromises the mechanics of
FT                   translocation; dbSNP:rs587777052)"
FT                   /evidence="ECO:0000269|PubMed:23001565"
FT                   /id="VAR_070792"
FT   MUTAGEN         595
FT                   /note="S->A: Strongly reduced phosphorylation at Thr-57."
FT                   /evidence="ECO:0000269|PubMed:23184662"
FT   MUTAGEN         599
FT                   /note="H->P: Strongly reduced phosphorylation at Thr-57."
FT                   /evidence="ECO:0000269|PubMed:23184662"
FT   MUTAGEN         715
FT                   /note="H->L,M,N,Q: Confers resistance to diphtheria toxin."
FT                   /evidence="ECO:0000269|PubMed:16901746"
SQ   SEQUENCE   858 AA;  95338 MW;  78BD1710236C0D9C CRC64;
     MVNFTVDQIR AIMDKKANIR NMSVIAHVDH GKSTLTDSLV CKAGIIASAR AGETRFTDTR
     KDEQERCITI KSTAISLFYE LSENDLNFIK QSKDGAGFLI NLIDSPGHVD FSSEVTAALR
     VTDGALVVVD CVSGVCVQTE TVLRQAIAER IKPVLMMNKM DRALLELQLE PEELYQTFQR
     IVENVNVIIS TYGEGESGPM GNIMIDPVLG TVGFGSGLHG WAFTLKQFAE MYVAKFAAKG
     EGQLGPAERA KKVEDMMKKL WGDRYFDPAN GKFSKSATSP EGKKLPRTFC QLILDPIFKV
     FDAIMNFKKE ETAKLIEKLD IKLDSEDKDK EGKPLLKAVM RRWLPAGDAL LQMITIHLPS
     PVTAQKYRCE LLYEGPPDDE AAMGIKSCDP KGPLMMYISK MVPTSDKGRF YAFGRVFSGL
     VSTGLKVRIM GPNYTPGKKE DLYLKPIQRT ILMMGRYVEP IEDVPCGNIV GLVGVDQFLV
     KTGTITTFEH AHNMRVMKFS VSPVVRVAVE AKNPADLPKL VEGLKRLAKS DPMVQCIIEE
     SGEHIIAGAG ELHLEICLKD LEEDHACIPI KKSDPVVSYR ETVSEESNVL CLSKSPNKHN
     RLYMKARPFP DGLAEDIDKG EVSARQELKQ RARYLAEKYE WDVAEARKIW CFGPDGTGPN
     ILTDITKGVQ YLNEIKDSVV AGFQWATKEG ALCEENMRGV RFDVHDVTLH ADAIHRGGGQ
     IIPTARRCLY ASVLTAQPRL MEPIYLVEIQ CPEQVVGGIY GVLNRKRGHV FEESQVAGTP
     MFVVKAYLPV NESFGFTADL RSNTGGQAFP QCVFDHWQIL PGDPFDNSSR PSQVVAETRK
     RKGLKEGIPA LDNFLDKL
```

|  |
| --- |
| **Mascot:** http://www.matrixscience.com/ |
