# Supplementary material for: Chlorpromazine induces cytotoxic autophagy in glioblastoma cells via endoplasmic reticulum stress and unfolded protein response
Source: J Exp Clin Cancer Res. 2021 Nov 5;40:347. doi: 10.1186/s13046-021-02144-w (PMC8569984; doi:10.1186/s13046-021-02144-w)
Supplement: Supplementary file 1 — Additional file 1. [file 13046_2021_2144_MOESM1_ESM.zip › MASCOT IDs/Mascot Search Results_ HS90B_HUMAN.html]

Mascot Search Results: HS90B\_HUMAN
 

# MASCOT Search Results

## Protein View: HS90B\_HUMAN

### Heat shock protein HSP 90-beta OS=Homo sapiens OX=9606 GN=HSP90AB1 PE=1 SV=4

|  |  |
| --- | --- |
| Database: | SwissProt |
| Score: | 538 |
| Monoisotopic mass (Mr): | 83554 |
| Calculated pI: | 4.97 |
| Taxonomy: | Homo sapiens |

Sequence similarity is available as an NCBI BLAST search of HS90B\_HUMAN against nr.

### Search parameters

|  |  |
| --- | --- |
| MS data file: | `ppw_D10_157002889600.txt` |
| Enzyme: | Trypsin: cuts C-term side of KR unless next residue is P. |
| Fixed modifications: | Carbamidomethyl (C) |

### Protein sequence coverage: 12%

Matched peptides shown in ***bold red***.

|  |  |  |  |  |  |
| --- | --- | --- | --- | --- | --- |
| `1` | `MPEEVHHGEE` | `EVETFAFQAE` | `IAQLMSLIIN` | `TFYSNKEIFL` | `RELISNASDA` |
| `51` | `LDKIRYESLT` | `DPSKLDSGKE` | `LKIDIIPNPQ` | `ERTLTLVDTG` | `IGMTKADLIN` |
| `101` | `NLGTIAKSGT` | `KAFMEALQAG` | `ADISMIGQFG` | `VGFYSAYLVA` | `EKVVVITKHN` |
| `151` | `DDEQYAWESS` | `AGGSFTVRAD` | `HGEPIGRGTK` | `VILHLKEDQT` | `EYLEERRVKE` |
| `201` | `VVKKHSQFIG` | `YPITLYLEKE` | `REKEISDDEA` | `EEEKGEKEEE` | `DKDDEEKPKI` |
| `251` | `EDVGSDEEDD` | `SGKDKKKKTK` | `KIKEKYIDQE` | `ELNKTKPIWT` | `RNPDDITQEE` |
| `301` | `YGEFYKSLTN` | `DWEDHLAVKH` | `FSVEGQLEFR` | `ALLFIPRRAP` | `FDLFENKKKK` |
| `351` | `NNIKLYVRRV` | `FIMDSCDELI` | `PEYLNFIRGV` | `VDSEDLPLNI` | `SREMLQQSKI` |
| `401` | `LKVIRKNIVK` | `KCLELFSELA` | `EDKENYKKFY` | `EAFSKNLKLG` | `IHEDSTNRRR` |
| `451` | `LSELLRYHTS` | `QSGDEMTSLS` | `EYVSRMKETQ` | `KSIYYITGES` | `KEQVANSAFV` |
| `501` | `ERVRKRGFEV` | `VYMTEPIDEY` | `CVQQLKEFDG` | `KSLVSVTKEG` | `LELPEDEEEK` |
| `551` | `KKMEESKAKF` | `ENLCKLMKEI` | `LDKKVEKVTI` | `SNRLVSSPCC` | `IVTSTYGWTA` |
| `601` | `NMERIMKAQA` | `LRDNSTMGYM` | `MAKKHLEINP` | `DHPIVETLRQ` | `KAEADKNDKA` |
| `651` | `VKDLVVLLFE` | `TALLSSGFSL` | `EDPQTHSNRI` | `YRMIKLGLGI` | `DEDEVAAEEP` |
| `701` | `NAAVPDEIPP` | `LEGDEDASRM` | `EEVD` |  |  |

Unformatted sequence string: 724 residues (for pasting into other applications).

|  |  |  |  |
| --- | --- | --- | --- |
| Sort by | residue number | increasing mass | decreasing mass |
| Show | matched peptides only | predicted peptides also |  |

| Query | Start | – | End | Observed | Mr(expt) | Mr(calc) | ppm | M | Score | Expect | Rank | U | Peptide |
| --- | --- | --- | --- | --- | --- | --- | --- | --- | --- | --- | --- | --- | --- |
| 24 | 73 | – | 82 | 1194.6753 | 1193.6680 | 1193.6404 | 23.1 | 0 | 52 | 0.00015 | 1Score **> 27** indicates **identity** | U | K.IDIIPNPQER.T |
| 3 | 331 | – | 337 | 829.5649 | 828.5577 | 828.5221 | 42.9 | 0 | 46 | 0.00026 | 1Score **> 23** indicates **identity** | U | R.ALLFIPR.R |
| 44 | 379 | – | 392 | 1513.8182 | 1512.8109 | 1512.7784 | 21.5 | 0 | 87 | 7.9e-08 | 1Score **> 28** indicates **identity** Score **> 26** indicates **homology** | U | R.GVVDSEDLPLNISR.E |
| 79 | 457 | – | 475 | 2176.9980 | 2175.9907 | 2175.9379 | 24.3 | 0 | 159 | 2.2e-15 | 1Score **> 25** indicates **identity** Score **> 23** indicates **homology** | U | R.YHTSQSGDEMTSLSEYVSR.M |
| 90 | 482 | – | 502 | 2391.2354 | 2390.2281 | 2390.1754 | 22.1 | 1 | 157 | 5.4e-15 | 1Score **> 27** indicates **identity** | U | K.SIYYITGESKEQVANSAFVER.V |
| 67 | 624 | – | 639 | 1911.0822 | 1910.0749 | 1910.0374 | 19.7 | 1 | 112 | 1.1e-10 | 1Score **> 25** indicates **identity** Score **> 24** indicates **homology** | U | K.KHLEINPDHPIVETLR.Q |
| 54 | 625 | – | 639 | 1782.9872 | 1781.9799 | 1781.9424 | 21.1 | 0 | 76 | 6e-07 | 1Score **> 27** indicates **identity** | U | K.HLEINPDHPIVETLR.Q |

---

```
ID   HS90B_HUMAN             Reviewed;         724 AA.
AC   P08238; B2R5P0; Q5T9W7; Q9NQW0; Q9NTK6;
DT   01-AUG-1988, integrated into UniProtKB/Swiss-Prot.
DT   23-JAN-2007, sequence version 4.
DT   02-JUN-2021, entry version 257.
DE   RecName: Full=Heat shock protein HSP 90-beta;
DE            Short=HSP 90;
DE   AltName: Full=Heat shock 84 kDa;
DE            Short=HSP 84;
DE            Short=HSP84;
GN   Name=HSP90AB1 {ECO:0000312|HGNC:HGNC:5258}; Synonyms=HSP90B, HSPC2, HSPCB;
OS   Homo sapiens (Human).
OC   Eukaryota; Metazoa; Chordata; Craniata; Vertebrata; Euteleostomi; Mammalia;
OC   Eutheria; Euarchontoglires; Primates; Haplorrhini; Catarrhini; Hominidae;
OC   Homo.
OX   NCBI_TaxID=9606;
RN   [1]
RP   NUCLEOTIDE SEQUENCE [MRNA].
RX   PubMed=3301534; DOI=10.1016/0378-1119(87)90012-6;
RA   Rebbe N.F., Ware J., Bertina R.M., Modrich P., Stafford D.W.;
RT   "Nucleotide sequence of a cDNA for a member of the human 90-kDa heat-shock
RT   protein family.";
RL   Gene 53:235-245(1987).
RN   [2]
RP   NUCLEOTIDE SEQUENCE [GENOMIC DNA].
RX   PubMed=2768249;
RA   Rebbe N.F., Hickman W.S., Ley T.J., Stafford D.W., Hickman S.;
RT   "Nucleotide sequence and regulation of a human 90-kDa heat shock protein
RT   gene.";
RL   J. Biol. Chem. 264:15006-15011(1989).
RN   [3]
RP   NUCLEOTIDE SEQUENCE [MRNA].
RX   PubMed=2469626; DOI=10.1016/0378-1119(88)90182-5;
RA   Hoffmann T., Hovemann B.;
RT   "Heat-shock proteins, Hsp84 and Hsp86, of mice and men: two related genes
RT   encode formerly identified tumour-specific transplantation antigens.";
RL   Gene 74:491-501(1988).
RN   [4]
RP   NUCLEOTIDE SEQUENCE [MRNA].
RC   TISSUE=Testis;
RA   Lu L., Huang X.Y., Yin L.L., Xu M., Li J.M., Zhou Z.M., Sha J.H.;
RT   "Cloning a new isoform of heat shock 90kDa in testis.";
RL   Submitted (AUG-2003) to the EMBL/GenBank/DDBJ databases.
RN   [5]
RP   NUCLEOTIDE SEQUENCE [LARGE SCALE MRNA].
RC   TISSUE=Amygdala;
RX   PubMed=11230166; DOI=10.1101/gr.gr1547r;
RA   Wiemann S., Weil B., Wellenreuther R., Gassenhuber J., Glassl S.,
RA   Ansorge W., Boecher M., Bloecker H., Bauersachs S., Blum H., Lauber J.,
RA   Duesterhoeft A., Beyer A., Koehrer K., Strack N., Mewes H.-W.,
RA   Ottenwaelder B., Obermaier B., Tampe J., Heubner D., Wambutt R., Korn B.,
RA   Klein M., Poustka A.;
RT   "Towards a catalog of human genes and proteins: sequencing and analysis of
RT   500 novel complete protein coding human cDNAs.";
RL   Genome Res. 11:422-435(2001).
RN   [6]
RP   NUCLEOTIDE SEQUENCE [LARGE SCALE MRNA].
RX   PubMed=14702039; DOI=10.1038/ng1285;
RA   Ota T., Suzuki Y., Nishikawa T., Otsuki T., Sugiyama T., Irie R.,
RA   Wakamatsu A., Hayashi K., Sato H., Nagai K., Kimura K., Makita H.,
RA   Sekine M., Obayashi M., Nishi T., Shibahara T., Tanaka T., Ishii S.,
RA   Yamamoto J., Saito K., Kawai Y., Isono Y., Nakamura Y., Nagahari K.,
RA   Murakami K., Yasuda T., Iwayanagi T., Wagatsuma M., Shiratori A., Sudo H.,
RA   Hosoiri T., Kaku Y., Kodaira H., Kondo H., Sugawara M., Takahashi M.,
RA   Kanda K., Yokoi T., Furuya T., Kikkawa E., Omura Y., Abe K., Kamihara K.,
RA   Katsuta N., Sato K., Tanikawa M., Yamazaki M., Ninomiya K., Ishibashi T.,
RA   Yamashita H., Murakawa K., Fujimori K., Tanai H., Kimata M., Watanabe M.,
RA   Hiraoka S., Chiba Y., Ishida S., Ono Y., Takiguchi S., Watanabe S.,
RA   Yosida M., Hotuta T., Kusano J., Kanehori K., Takahashi-Fujii A., Hara H.,
RA   Tanase T.-O., Nomura Y., Togiya S., Komai F., Hara R., Takeuchi K.,
RA   Arita M., Imose N., Musashino K., Yuuki H., Oshima A., Sasaki N.,
RA   Aotsuka S., Yoshikawa Y., Matsunawa H., Ichihara T., Shiohata N., Sano S.,
RA   Moriya S., Momiyama H., Satoh N., Takami S., Terashima Y., Suzuki O.,
RA   Nakagawa S., Senoh A., Mizoguchi H., Goto Y., Shimizu F., Wakebe H.,
RA   Hishigaki H., Watanabe T., Sugiyama A., Takemoto M., Kawakami B.,
RA   Yamazaki M., Watanabe K., Kumagai A., Itakura S., Fukuzumi Y., Fujimori Y.,
RA   Komiyama M., Tashiro H., Tanigami A., Fujiwara T., Ono T., Yamada K.,
RA   Fujii Y., Ozaki K., Hirao M., Ohmori Y., Kawabata A., Hikiji T.,
RA   Kobatake N., Inagaki H., Ikema Y., Okamoto S., Okitani R., Kawakami T.,
RA   Noguchi S., Itoh T., Shigeta K., Senba T., Matsumura K., Nakajima Y.,
RA   Mizuno T., Morinaga M., Sasaki M., Togashi T., Oyama M., Hata H.,
RA   Watanabe M., Komatsu T., Mizushima-Sugano J., Satoh T., Shirai Y.,
RA   Takahashi Y., Nakagawa K., Okumura K., Nagase T., Nomura N., Kikuchi H.,
RA   Masuho Y., Yamashita R., Nakai K., Yada T., Nakamura Y., Ohara O.,
RA   Isogai T., Sugano S.;
RT   "Complete sequencing and characterization of 21,243 full-length human
RT   cDNAs.";
RL   Nat. Genet. 36:40-45(2004).
RN   [7]
RP   NUCLEOTIDE SEQUENCE [GENOMIC DNA].
RG   NHLBI resequencing and genotyping service (RS&G);
RL   Submitted (DEC-2005) to the EMBL/GenBank/DDBJ databases.
RN   [8]
RP   NUCLEOTIDE SEQUENCE [LARGE SCALE GENOMIC DNA].
RX   PubMed=14574404; DOI=10.1038/nature02055;
RA   Mungall A.J., Palmer S.A., Sims S.K., Edwards C.A., Ashurst J.L.,
RA   Wilming L., Jones M.C., Horton R., Hunt S.E., Scott C.E., Gilbert J.G.R.,
RA   Clamp M.E., Bethel G., Milne S., Ainscough R., Almeida J.P., Ambrose K.D.,
RA   Andrews T.D., Ashwell R.I.S., Babbage A.K., Bagguley C.L., Bailey J.,
RA   Banerjee R., Barker D.J., Barlow K.F., Bates K., Beare D.M., Beasley H.,
RA   Beasley O., Bird C.P., Blakey S.E., Bray-Allen S., Brook J., Brown A.J.,
RA   Brown J.Y., Burford D.C., Burrill W., Burton J., Carder C., Carter N.P.,
RA   Chapman J.C., Clark S.Y., Clark G., Clee C.M., Clegg S., Cobley V.,
RA   Collier R.E., Collins J.E., Colman L.K., Corby N.R., Coville G.J.,
RA   Culley K.M., Dhami P., Davies J., Dunn M., Earthrowl M.E., Ellington A.E.,
RA   Evans K.A., Faulkner L., Francis M.D., Frankish A., Frankland J.,
RA   French L., Garner P., Garnett J., Ghori M.J., Gilby L.M., Gillson C.J.,
RA   Glithero R.J., Grafham D.V., Grant M., Gribble S., Griffiths C.,
RA   Griffiths M.N.D., Hall R., Halls K.S., Hammond S., Harley J.L., Hart E.A.,
RA   Heath P.D., Heathcott R., Holmes S.J., Howden P.J., Howe K.L., Howell G.R.,
RA   Huckle E., Humphray S.J., Humphries M.D., Hunt A.R., Johnson C.M.,
RA   Joy A.A., Kay M., Keenan S.J., Kimberley A.M., King A., Laird G.K.,
RA   Langford C., Lawlor S., Leongamornlert D.A., Leversha M., Lloyd C.R.,
RA   Lloyd D.M., Loveland J.E., Lovell J., Martin S., Mashreghi-Mohammadi M.,
RA   Maslen G.L., Matthews L., McCann O.T., McLaren S.J., McLay K., McMurray A.,
RA   Moore M.J.F., Mullikin J.C., Niblett D., Nickerson T., Novik K.L.,
RA   Oliver K., Overton-Larty E.K., Parker A., Patel R., Pearce A.V., Peck A.I.,
RA   Phillimore B.J.C.T., Phillips S., Plumb R.W., Porter K.M., Ramsey Y.,
RA   Ranby S.A., Rice C.M., Ross M.T., Searle S.M., Sehra H.K., Sheridan E.,
RA   Skuce C.D., Smith S., Smith M., Spraggon L., Squares S.L., Steward C.A.,
RA   Sycamore N., Tamlyn-Hall G., Tester J., Theaker A.J., Thomas D.W.,
RA   Thorpe A., Tracey A., Tromans A., Tubby B., Wall M., Wallis J.M.,
RA   West A.P., White S.S., Whitehead S.L., Whittaker H., Wild A., Willey D.J.,
RA   Wilmer T.E., Wood J.M., Wray P.W., Wyatt J.C., Young L., Younger R.M.,
RA   Bentley D.R., Coulson A., Durbin R.M., Hubbard T., Sulston J.E., Dunham I.,
RA   Rogers J., Beck S.;
RT   "The DNA sequence and analysis of human chromosome 6.";
RL   Nature 425:805-811(2003).
RN   [9]
RP   NUCLEOTIDE SEQUENCE [LARGE SCALE GENOMIC DNA].
RA   Mural R.J., Istrail S., Sutton G.G., Florea L., Halpern A.L., Mobarry C.M.,
RA   Lippert R., Walenz B., Shatkay H., Dew I., Miller J.R., Flanigan M.J.,
RA   Edwards N.J., Bolanos R., Fasulo D., Halldorsson B.V., Hannenhalli S.,
RA   Turner R., Yooseph S., Lu F., Nusskern D.R., Shue B.C., Zheng X.H.,
RA   Zhong F., Delcher A.L., Huson D.H., Kravitz S.A., Mouchard L., Reinert K.,
RA   Remington K.A., Clark A.G., Waterman M.S., Eichler E.E., Adams M.D.,
RA   Hunkapiller M.W., Myers E.W., Venter J.C.;
RL   Submitted (JUL-2005) to the EMBL/GenBank/DDBJ databases.
RN   [10]
RP   NUCLEOTIDE SEQUENCE [LARGE SCALE MRNA].
RC   TISSUE=Colon, Lymph, Muscle, Skin, and Testis;
RX   PubMed=15489334; DOI=10.1101/gr.2596504;
RG   The MGC Project Team;
RT   "The status, quality, and expansion of the NIH full-length cDNA project:
RT   the Mammalian Gene Collection (MGC).";
RL   Genome Res. 14:2121-2127(2004).
RN   [11]
RP   PROTEIN SEQUENCE OF 2-21, AND PHOSPHORYLATION.
RX   PubMed=2492519;
RA   Lees-Miller S.P., Anderson C.W.;
RT   "Two human 90-kDa heat shock proteins are phosphorylated in vivo at
RT   conserved serines that are phosphorylated in vitro by casein kinase II.";
RL   J. Biol. Chem. 264:2431-2437(1989).
RN   [12]
RP   PROTEIN SEQUENCE OF 42-107; 149-168; 181-197; 204-221; 250-265; 274-284;
RP   292-348; 360-392; 412-427; 439-448; 450-475; 482-502; 506-526; 539-551;
RP   584-604; 613-639 AND 653-679, PHOSPHORYLATION AT SER-255, AND
RP   IDENTIFICATION BY MASS SPECTROMETRY.
RC   TISSUE=Embryonic kidney;
RA   Bienvenut W.V., Waridel P., Quadroni M.;
RL   Submitted (MAR-2009) to UniProtKB.
RN   [13]
RP   NUCLEOTIDE SEQUENCE [GENOMIC DNA] OF 50-118.
RX   PubMed=8180474; DOI=10.1007/bf00292342;
RA   Takahashi I., Tanuma R., Hirata M., Hashimoto K.;
RT   "A cosmid clone at the D6S182 locus on human chromosome 6p12 contains the
RT   90-kDa heat shock protein beta gene (HSP90 beta).";
RL   Mamm. Genome 5:121-122(1994).
RN   [14]
RP   SEQUENCE REVISION.
RA   Takahashi I., Tanuma R., Hirata M., Hashimoto K.;
RL   Submitted (JUL-2011) to the EMBL/GenBank/DDBJ databases.
RN   [15]
RP   PROTEIN SEQUENCE OF 54-64 AND 187-199.
RC   TISSUE=Colon carcinoma;
RX   PubMed=9150948; DOI=10.1002/elps.1150180344;
RA   Ji H., Reid G.E., Moritz R.L., Eddes J.S., Burgess A.W., Simpson R.J.;
RT   "A two-dimensional gel database of human colon carcinoma proteins.";
RL   Electrophoresis 18:605-613(1997).
RN   [16]
RP   NUCLEOTIDE SEQUENCE [MRNA] OF 93-724.
RC   TISSUE=Pancreas;
RA   Mason A., O'Connor D., Greenhalf W.;
RT   "Novel sequence for human Hsp90 beta giving a substitution of R55T (R147 in
RT   original sequence) and M85R (M177 in original sequence).";
RL   Submitted (JUN-2000) to the EMBL/GenBank/DDBJ databases.
RN   [17]
RP   HOMODIMERIZATION.
RX   PubMed=7588731; DOI=10.1111/j.1432-1033.1995.001_1.x;
RA   Nemoto T., Ohara-Nemoto Y., Ota M., Takagi T., Yokoyama K.;
RT   "Mechanism of dimer formation of the 90-kDa heat-shock protein.";
RL   Eur. J. Biochem. 233:1-8(1995).
RN   [18]
RP   SUBCELLULAR LOCATION, AND INTERACTION WITH CDK6 AND CDC37.
RX   PubMed=9482106; DOI=10.1038/sj.onc.1201570;
RA   Mahony D., Parry D.A., Lees E.;
RT   "Active cdk6 complexes are predominantly nuclear and represent only a
RT   minority of the cdk6 in T cells.";
RL   Oncogene 16:603-611(1998).
RN   [19]
RP   IDENTIFICATION BY MASS SPECTROMETRY.
RC   TISSUE=Lymphoblast;
RX   PubMed=14654843; DOI=10.1038/nature02166;
RA   Andersen J.S., Wilkinson C.J., Mayor T., Mortensen P., Nigg E.A., Mann M.;
RT   "Proteomic characterization of the human centrosome by protein correlation
RT   profiling.";
RL   Nature 426:570-574(2003).
RN   [20]
RP   PHOSPHORYLATION AT SER-226 AND SER-255, MUTAGENESIS OF SER-226 AND SER-255,
RP   AND INTERACTION WITH AHR.
RX   PubMed=15581363; DOI=10.1021/bi048736m;
RA   Ogiso H., Kagi N., Matsumoto E., Nishimoto M., Arai R., Shirouzu M.,
RA   Mimura J., Fujii-Kuriyama Y., Yokoyama S.;
RT   "Phosphorylation analysis of 90 kDa heat shock protein within the cytosolic
RT   arylhydrocarbon receptor complex.";
RL   Biochemistry 43:15510-15519(2004).
RN   [21]
RP   INTERACTION WITH TP53, AND REGION.
RX   PubMed=15358771; DOI=10.1074/jbc.m407687200;
RA   Mueller L., Schaupp A., Walerych D., Wegele H., Buchner J.;
RT   "Hsp90 regulates the activity of wild type p53 under physiological and
RT   elevated temperatures.";
RL   J. Biol. Chem. 279:48846-48854(2004).
RN   [22]
RP   ISGYLATION.
RX   PubMed=16139798; DOI=10.1016/j.bbrc.2005.08.132;
RA   Giannakopoulos N.V., Luo J.K., Papov V., Zou W., Lenschow D.J.,
RA   Jacobs B.S., Borden E.C., Li J., Virgin H.W., Zhang D.E.;
RT   "Proteomic identification of proteins conjugated to ISG15 in mouse and
RT   human cells.";
RL   Biochem. Biophys. Res. Commun. 336:496-506(2005).
RN   [23]
RP   IDENTIFICATION BY MASS SPECTROMETRY [LARGE SCALE ANALYSIS].
RX   PubMed=15592455; DOI=10.1038/nbt1046;
RA   Rush J., Moritz A., Lee K.A., Guo A., Goss V.L., Spek E.J., Zhang H.,
RA   Zha X.-M., Polakiewicz R.D., Comb M.J.;
RT   "Immunoaffinity profiling of tyrosine phosphorylation in cancer cells.";
RL   Nat. Biotechnol. 23:94-101(2005).
RN   [24]
RP   INTERACTION WITH SGTA, AND SUBCELLULAR LOCATION.
RX   PubMed=16580629; DOI=10.1016/j.bbrc.2006.03.090;
RA   Yin H., Wang H., Zong H., Chen X., Wang Y., Yun X., Wu Y., Wang J., Gu J.;
RT   "SGT, a Hsp90beta binding partner, is accumulated in the nucleus during
RT   cell apoptosis.";
RL   Biochem. Biophys. Res. Commun. 343:1153-1158(2006).
RN   [25]
RP   PHOSPHORYLATION [LARGE SCALE ANALYSIS] AT SER-255, AND IDENTIFICATION BY
RP   MASS SPECTROMETRY [LARGE SCALE ANALYSIS].
RC   TISSUE=Cervix carcinoma;
RX   PubMed=17081983; DOI=10.1016/j.cell.2006.09.026;
RA   Olsen J.V., Blagoev B., Gnad F., Macek B., Kumar C., Mortensen P., Mann M.;
RT   "Global, in vivo, and site-specific phosphorylation dynamics in signaling
RT   networks.";
RL   Cell 127:635-648(2006).
RN   [26]
RP   SUBCELLULAR LOCATION [LARGE SCALE ANALYSIS].
RC   TISSUE=Melanoma;
RX   PubMed=17081065; DOI=10.1021/pr060363j;
RA   Chi A., Valencia J.C., Hu Z.-Z., Watabe H., Yamaguchi H., Mangini N.J.,
RA   Huang H., Canfield V.A., Cheng K.C., Yang F., Abe R., Yamagishi S.,
RA   Shabanowitz J., Hearing V.J., Wu C., Appella E., Hunt D.F.;
RT   "Proteomic and bioinformatic characterization of the biogenesis and
RT   function of melanosomes.";
RL   J. Proteome Res. 5:3135-3144(2006).
RN   [27]
RP   FUNCTION, AND INTERACTION WITH UNC45A.
RX   PubMed=16478993; DOI=10.1128/mcb.26.5.1722-1730.2006;
RA   Chadli A., Graham J.D., Abel M.G., Jackson T.A., Gordon D.F., Wood W.M.,
RA   Felts S.J., Horwitz K.B., Toft D.;
RT   "GCUNC-45 is a novel regulator for the progesterone receptor/hsp90
RT   chaperoning pathway.";
RL   Mol. Cell. Biol. 26:1722-1730(2006).
RN   [28]
RP   PHOSPHORYLATION [LARGE SCALE ANALYSIS] AT THR-297, AND IDENTIFICATION BY
RP   MASS SPECTROMETRY [LARGE SCALE ANALYSIS].
RC   TISSUE=Embryonic kidney;
RX   PubMed=17525332; DOI=10.1126/science.1140321;
RA   Matsuoka S., Ballif B.A., Smogorzewska A., McDonald E.R. III, Hurov K.E.,
RA   Luo J., Bakalarski C.E., Zhao Z., Solimini N., Lerenthal Y., Shiloh Y.,
RA   Gygi S.P., Elledge S.J.;
RT   "ATM and ATR substrate analysis reveals extensive protein networks
RT   responsive to DNA damage.";
RL   Science 316:1160-1166(2007).
RN   [29]
RP   UBIQUITINATION.
RX   PubMed=18042044; DOI=10.1042/bj20071338;
RA   Windheim M., Peggie M., Cohen P.;
RT   "Two different classes of E2 ubiquitin-conjugating enzymes are required for
RT   the mono-ubiquitination of proteins and elongation by polyubiquitin chains
RT   with a specific topology.";
RL   Biochem. J. 409:723-729(2008).
RN   [30]
RP   INTERACTION WITH DNAJC7.
RX   PubMed=18620420; DOI=10.1021/bi800770g;
RA   Moffatt N.S., Bruinsma E., Uhl C., Obermann W.M., Toft D.;
RT   "Role of the cochaperone Tpr2 in Hsp90 chaperoning.";
RL   Biochemistry 47:8203-8213(2008).
RN   [31]
RP   IDENTIFICATION BY MASS SPECTROMETRY, INTERACTION WITH BIRC2, SUBCELLULAR
RP   LOCATION, AND FUNCTION.
RX   PubMed=18239673; DOI=10.1038/cdd.2008.5;
RA   Didelot C., Lanneau D., Brunet M., Bouchot A., Cartier J., Jacquel A.,
RA   Ducoroy P., Cathelin S., Decologne N., Chiosis G., Dubrez-Daloz L.,
RA   Solary E., Garrido C.;
RT   "Interaction of heat-shock protein 90 beta isoform (HSP90 beta) with
RT   cellular inhibitor of apoptosis 1 (c-IAP1) is required for cell
RT   differentiation.";
RL   Cell Death Differ. 15:859-866(2008).
RN   [32]
RP   SUBUNIT, ACTIVITY REGULATION, AND BIOPHYSICOCHEMICAL PROPERTIES.
RX   PubMed=18400751; DOI=10.1074/jbc.m800540200;
RA   Richter K., Soroka J., Skalniak L., Leskovar A., Hessling M., Reinstein J.,
RA   Buchner J.;
RT   "Conserved conformational changes in the ATPase cycle of human Hsp90.";
RL   J. Biol. Chem. 283:17757-17765(2008).
RN   [33]
RP   PHOSPHORYLATION [LARGE SCALE ANALYSIS] AT SER-226, AND IDENTIFICATION BY
RP   MASS SPECTROMETRY [LARGE SCALE ANALYSIS].
RC   TISSUE=Platelet;
RX   PubMed=18088087; DOI=10.1021/pr0704130;
RA   Zahedi R.P., Lewandrowski U., Wiesner J., Wortelkamp S., Moebius J.,
RA   Schuetz C., Walter U., Gambaryan S., Sickmann A.;
RT   "Phosphoproteome of resting human platelets.";
RL   J. Proteome Res. 7:526-534(2008).
RN   [34]
RP   INTERACTION WITH TTC4.
RX   PubMed=18320024; DOI=10.1371/journal.pone.0001737;
RA   Crevel G., Bennett D., Cotterill S.;
RT   "The human TPR protein TTC4 is a putative Hsp90 co-chaperone which
RT   interacts with CDC6 and shows alterations in transformed cells.";
RL   PLoS ONE 3:E0001737-E0001737(2008).
RN   [35]
RP   PHOSPHORYLATION [LARGE SCALE ANALYSIS] AT SER-307, AND IDENTIFICATION BY
RP   MASS SPECTROMETRY [LARGE SCALE ANALYSIS].
RC   TISSUE=Cervix carcinoma;
RX   PubMed=18669648; DOI=10.1073/pnas.0805139105;
RA   Dephoure N., Zhou C., Villen J., Beausoleil S.A., Bakalarski C.E.,
RA   Elledge S.J., Gygi S.P.;
RT   "A quantitative atlas of mitotic phosphorylation.";
RL   Proc. Natl. Acad. Sci. U.S.A. 105:10762-10767(2008).
RN   [36]
RP   PHOSPHORYLATION [LARGE SCALE ANALYSIS] AT SER-226, AND IDENTIFICATION BY
RP   MASS SPECTROMETRY [LARGE SCALE ANALYSIS].
RC   TISSUE=Liver;
RX   PubMed=18318008; DOI=10.1002/pmic.200700884;
RA   Han G., Ye M., Zhou H., Jiang X., Feng S., Jiang X., Tian R., Wan D.,
RA   Zou H., Gu J.;
RT   "Large-scale phosphoproteome analysis of human liver tissue by enrichment
RT   and fractionation of phosphopeptides with strong anion exchange
RT   chromatography.";
RL   Proteomics 8:1346-1361(2008).
RN   [37]
RP   FUNCTION, S-NITROSYLATION AT CYS-590, AND MUTAGENESIS OF CYS-590.
RX   PubMed=19696785; DOI=10.1038/embor.2009.153;
RA   Retzlaff M., Stahl M., Eberl H.C., Lagleder S., Beck J., Kessler H.,
RA   Buchner J.;
RT   "Hsp90 is regulated by a switch point in the C-terminal domain.";
RL   EMBO Rep. 10:1147-1153(2009).
RN   [38]
RP   ACETYLATION [LARGE SCALE ANALYSIS] AT LYS-435 AND LYS-481, AND
RP   IDENTIFICATION BY MASS SPECTROMETRY [LARGE SCALE ANALYSIS].
RX   PubMed=19608861; DOI=10.1126/science.1175371;
RA   Choudhary C., Kumar C., Gnad F., Nielsen M.L., Rehman M., Walther T.C.,
RA   Olsen J.V., Mann M.;
RT   "Lysine acetylation targets protein complexes and co-regulates major
RT   cellular functions.";
RL   Science 325:834-840(2009).
RN   [39]
RP   INTERACTION WITH TGFB1 PROCESSED FORM (LAP), AND SUBCELLULAR LOCATION.
RX   PubMed=20599762; DOI=10.1016/j.bbrc.2010.06.112;
RA   Suzuki S., Kulkarni A.B.;
RT   "Extracellular heat shock protein HSP90beta secreted by MG63 osteosarcoma
RT   cells inhibits activation of latent TGF-beta1.";
RL   Biochem. Biophys. Res. Commun. 398:525-531(2010).
RN   [40]
RP   INTERACTION WITH HSP90AA1; JAK2 AND PRKCE, INDUCTION, AND FUNCTION.
RX   PubMed=20353823; DOI=10.1016/j.cellsig.2010.03.012;
RA   Cheng M.B., Zhang Y., Zhong X., Sutter B., Cao C.Y., Chen X.S., Cheng X.K.,
RA   Zhang Y., Xiao L., Shen Y.F.;
RT   "Stat1 mediates an auto-regulation of hsp90beta gene in heat shock
RT   response.";
RL   Cell. Signal. 22:1206-1213(2010).
RN   [41]
RP   PHOSPHORYLATION [LARGE SCALE ANALYSIS] AT SER-226, AND IDENTIFICATION BY
RP   MASS SPECTROMETRY [LARGE SCALE ANALYSIS].
RC   TISSUE=Cervix carcinoma;
RX   PubMed=20068231; DOI=10.1126/scisignal.2000475;
RA   Olsen J.V., Vermeulen M., Santamaria A., Kumar C., Miller M.L.,
RA   Jensen L.J., Gnad F., Cox J., Jensen T.S., Nigg E.A., Brunak S., Mann M.;
RT   "Quantitative phosphoproteomics reveals widespread full phosphorylation
RT   site occupancy during mitosis.";
RL   Sci. Signal. 3:RA3-RA3(2010).
RN   [42]
RP   IDENTIFICATION BY MASS SPECTROMETRY [LARGE SCALE ANALYSIS].
RX   PubMed=21269460; DOI=10.1186/1752-0509-5-17;
RA   Burkard T.R., Planyavsky M., Kaupe I., Breitwieser F.P., Buerckstuemmer T.,
RA   Bennett K.L., Superti-Furga G., Colinge J.;
RT   "Initial characterization of the human central proteome.";
RL   BMC Syst. Biol. 5:17-17(2011).
RN   [43]
RP   MALONYLATION AT LYS-399.
RX   PubMed=21908771; DOI=10.1074/mcp.m111.012658;
RA   Peng C., Lu Z., Xie Z., Cheng Z., Chen Y., Tan M., Luo H., Zhang Y., He W.,
RA   Yang K., Zwaans B.M., Tishkoff D., Ho L., Lombard D., He T.C., Dai J.,
RA   Verdin E., Ye Y., Zhao Y.;
RT   "The first identification of lysine malonylation substrates and its
RT   regulatory enzyme.";
RL   Mol. Cell. Proteomics 10:M111.012658.01-M111.012658.12(2011).
RN   [44]
RP   INTERACTION WITH AHSA1 AND XPO1.
RX   PubMed=22022502; DOI=10.1371/journal.pone.0026044;
RA   Echeverria P.C., Bernthaler A., Dupuis P., Mayer B., Picard D.;
RT   "An interaction network predicted from public data as a discovery tool:
RT   application to the Hsp90 molecular chaperone machine.";
RL   PLoS ONE 6:E26044-E26044(2011).
RN   [45]
RP   PHOSPHORYLATION [LARGE SCALE ANALYSIS] AT SER-226, AND IDENTIFICATION BY
RP   MASS SPECTROMETRY [LARGE SCALE ANALYSIS].
RX   PubMed=21406692; DOI=10.1126/scisignal.2001570;
RA   Rigbolt K.T., Prokhorova T.A., Akimov V., Henningsen J., Johansen P.T.,
RA   Kratchmarova I., Kassem M., Mann M., Olsen J.V., Blagoev B.;
RT   "System-wide temporal characterization of the proteome and phosphoproteome
RT   of human embryonic stem cell differentiation.";
RL   Sci. Signal. 4:RS3-RS3(2011).
RN   [46]
RP   PHOSPHORYLATION AT SER-718 BY PLK2 AND PLK3.
RX   PubMed=22828320; DOI=10.1016/j.bbapap.2012.07.003;
RA   Salvi M., Trashi E., Cozza G., Franchin C., Arrigoni G., Pinna L.A.;
RT   "Investigation on PLK2 and PLK3 substrate recognition.";
RL   Biochim. Biophys. Acta 1824:1366-1373(2012).
RN   [47]
RP   INTERACTION WITH CDC25A.
RX   PubMed=22843495; DOI=10.1093/hmg/dds303;
RA   Giessrigl B., Krieger S., Rosner M., Huttary N., Saiko P., Alami M.,
RA   Messaoudi S., Peyrat J.F., Maciuk A., Gollinger M., Kopf S., Kazlauskas E.,
RA   Mazal P., Szekeres T., Hengstschlaeger M., Matulis D., Jaeger W.,
RA   Krupitza G.;
RT   "Hsp90 stabilizes Cdc25A and counteracts heat shock-mediated Cdc25A
RT   degradation and cell-cycle attenuation in pancreatic carcinoma cells.";
RL   Hum. Mol. Genet. 21:4615-4627(2012).
RN   [48]
RP   PROTEIN CLEAVAGE, AND IDENTIFICATION BY MASS SPECTROMETRY.
RX   PubMed=22848402; DOI=10.1371/journal.pone.0040795;
RA   Beck R., Dejeans N., Glorieux C., Creton M., Delaive E., Dieu M., Raes M.,
RA   Leveque P., Gallez B., Depuydt M., Collet J.F., Calderon P.B., Verrax J.;
RT   "Hsp90 is cleaved by reactive oxygen species at a highly conserved N-
RT   terminal amino acid motif.";
RL   PLoS ONE 7:E40795-E40795(2012).
RN   [49]
RP   INTERACTION WITH NOS3, MUTAGENESIS OF TYR-301, AND PHOSPHORYLATION AT
RP   TYR-301 BY SRC.
RX   PubMed=23585225; DOI=10.1152/ajplung.00419.2012;
RA   Barabutis N., Handa V., Dimitropoulou C., Rafikov R., Snead C., Kumar S.,
RA   Joshi A., Thangjam G., Fulton D., Black S.M., Patel V., Catravas J.D.;
RT   "LPS induces pp60c-src-mediated tyrosine phosphorylation of Hsp90 in lung
RT   vascular endothelial cells and mouse lung.";
RL   Am. J. Physiol. 304:L883-L893(2013).
RN   [50]
RP   PHOSPHORYLATION [LARGE SCALE ANALYSIS] AT SER-226; SER-255; THR-297;
RP   SER-445 AND THR-479, AND IDENTIFICATION BY MASS SPECTROMETRY [LARGE SCALE
RP   ANALYSIS].
RC   TISSUE=Cervix carcinoma, and Erythroleukemia;
RX   PubMed=23186163; DOI=10.1021/pr300630k;
RA   Zhou H., Di Palma S., Preisinger C., Peng M., Polat A.N., Heck A.J.,
RA   Mohammed S.;
RT   "Toward a comprehensive characterization of a human cancer cell
RT   phosphoproteome.";
RL   J. Proteome Res. 12:260-271(2013).
RN   [51]
RP   INTERACTION WITH MAPK7.
RX   PubMed=23428871; DOI=10.1128/mcb.01246-12;
RA   Erazo T., Moreno A., Ruiz-Babot G., Rodriguez-Asiain A., Morrice N.A.,
RA   Espadamala J., Bayascas J.R., Gomez N., Lizcano J.M.;
RT   "Canonical and kinase activity-independent mechanisms for extracellular
RT   signal-regulated kinase 5 (ERK5) nuclear translocation require dissociation
RT   of Hsp90 from the ERK5-Cdc37 complex.";
RL   Mol. Cell. Biol. 33:1671-1686(2013).
RN   [52]
RP   INTERACTION WITH KCNQ4.
RX   PubMed=23431407; DOI=10.1371/journal.pone.0057282;
RA   Gao Y., Yechikov S., Vazquez A.E., Chen D., Nie L.;
RT   "Distinct roles of molecular chaperones HSP90alpha and HSP90beta in the
RT   biogenesis of KCNQ4 channels.";
RL   PLoS ONE 8:E57282-E57282(2013).
RN   [53]
RP   FUNCTION, AND INTERACTION WITH STUB1 AND SMAD3.
RX   PubMed=24613385; DOI=10.1016/j.bbrc.2014.02.124;
RA   Shang Y., Xu X., Duan X., Guo J., Wang Y., Ren F., He D., Chang Z.;
RT   "Hsp70 and Hsp90 oppositely regulate TGF-beta signaling through
RT   CHIP/Stub1.";
RL   Biochem. Biophys. Res. Commun. 446:387-392(2014).
RN   [54]
RP   METHYLATION AT LYS-531 AND LYS-574 BY SMYD2, IDENTIFICATION BY MASS
RP   SPECTROMETRY, MUTAGENESIS OF LYS-531 AND LYS-574, INTERACTION WITH STIP1
RP   AND CDC37, AND SUBCELLULAR LOCATION.
RX   PubMed=24880080; DOI=10.1016/j.canlet.2014.05.014;
RA   Hamamoto R., Toyokawa G., Nakakido M., Ueda K., Nakamura Y.;
RT   "SMYD2-dependent HSP90 methylation promotes cancer cell proliferation by
RT   regulating the chaperone complex formation.";
RL   Cancer Lett. 351:126-133(2014).
RN   [55]
RP   PHOSPHORYLATION [LARGE SCALE ANALYSIS] AT SER-669, AND IDENTIFICATION BY
RP   MASS SPECTROMETRY [LARGE SCALE ANALYSIS].
RC   TISSUE=Liver;
RX   PubMed=24275569; DOI=10.1016/j.jprot.2013.11.014;
RA   Bian Y., Song C., Cheng K., Dong M., Wang F., Huang J., Sun D., Wang L.,
RA   Ye M., Zou H.;
RT   "An enzyme assisted RP-RPLC approach for in-depth analysis of human liver
RT   phosphoproteome.";
RL   J. Proteomics 96:253-262(2014).
RN   [56]
RP   INTERACTION WITH NWD1.
RX   PubMed=24681825; DOI=10.18632/oncotarget.1850;
RA   Correa R.G., Krajewska M., Ware C.F., Gerlic M., Reed J.C.;
RT   "The NLR-related protein NWD1 is associated with prostate cancer and
RT   modulates androgen receptor signaling.";
RL   Oncotarget 5:1666-1682(2014).
RN   [57]
RP   INTERACTION WITH AHSA1; BIRC2 AND CDC37, AND REGION.
RX   PubMed=25486457; DOI=10.1016/j.bbamcr.2014.11.026;
RA   Synoradzki K., Bieganowski P.;
RT   "Middle domain of human Hsp90 isoforms differentially binds Aha1 in human
RT   cells and alters Hsp90 activity in yeast.";
RL   Biochim. Biophys. Acta 1853:445-452(2015).
RN   [58]
RP   REVIEW.
RX   PubMed=25973397; DOI=10.3389/fonc.2015.00100;
RA   Khurana N., Bhattacharyya S.;
RT   "Hsp90, the concertmaster: tuning transcription.";
RL   Front. Oncol. 5:100-100(2015).
RN   [59]
RP   INTERACTION WITH HSF1; HIF1A; ERBB2; MET; KEAP1 AND RHOBTB2, AND
RP   MUTAGENESIS OF GLU-42 AND ASP-88.
RX   PubMed=26517842; DOI=10.1371/journal.pone.0141786;
RA   Prince T.L., Kijima T., Tatokoro M., Lee S., Tsutsumi S., Yim K., Rivas C.,
RA   Alarcon S., Schwartz H., Khamit-Kush K., Scroggins B.T., Beebe K.,
RA   Trepel J.B., Neckers L.;
RT   "Client proteins and small molecule inhibitors display distinct binding
RT   preferences for constitutive and stress-induced HSP90 isoforms and their
RT   conformationally restricted mutants.";
RL   PLoS ONE 10:E0141786-E0141786(2015).
RN   [60]
RP   IDENTIFICATION BY MASS SPECTROMETRY [LARGE SCALE ANALYSIS].
RX   PubMed=25944712; DOI=10.1002/pmic.201400617;
RA   Vaca Jacome A.S., Rabilloud T., Schaeffer-Reiss C., Rompais M., Ayoub D.,
RA   Lane L., Bairoch A., Van Dorsselaer A., Carapito C.;
RT   "N-terminome analysis of the human mitochondrial proteome.";
RL   Proteomics 15:2519-2524(2015).
RN   [61]
RP   REVIEW.
RX   PubMed=27295069; DOI=10.1016/j.biochi.2016.05.018;
RA   Verma S., Goyal S., Jamal S., Singh A., Grover A.;
RT   "Hsp90: Friends, clients and natural foes.";
RL   Biochimie 127:227-240(2016).
RN   [62]
RP   REVIEW.
RX   PubMed=26991466; DOI=10.1002/bip.22835;
RA   Pearl L.H.;
RT   "Review: The HSP90 molecular chaperone-an enigmatic ATPase.";
RL   Biopolymers 105:594-607(2016).
RN   [63]
RP   FUNCTION, AND INTERACTION WITH IL1B.
RX   PubMed=32272059; DOI=10.1016/j.cell.2020.03.031;
RA   Zhang M., Liu L., Lin X., Wang Y., Li Y., Guo Q., Li S., Sun Y., Tao X.,
RA   Zhang D., Lv X., Zheng L., Ge L.;
RT   "A Translocation Pathway for Vesicle-Mediated Unconventional Protein
RT   Secretion.";
RL   Cell 181:637-652(2020).
RN   [64]
RP   X-RAY CRYSTALLOGRAPHY (2.45 ANGSTROMS) OF 1-221 IN COMPLEX WITH PURINE
RP   ANALOG.
RX   PubMed=15217611; DOI=10.1016/j.chembiol.2004.03.033;
RA   Wright L., Barril X., Dymock B., Sheridan L., Surgenor A., Beswick M.,
RA   Drysdale M., Collier A., Massey A., Davies N., Fink A., Fromont C.,
RA   Aherne W., Boxall K., Sharp S., Workman P., Hubbard R.E.;
RT   "Structure-activity relationships in purine-based inhibitor binding to
RT   HSP90 isoforms.";
RL   Chem. Biol. 11:775-785(2004).
RN   [65]
RP   X-RAY CRYSTALLOGRAPHY (3.00 ANGSTROMS) OF 720-724 IN COMPLEX WITH FKBP4.
RX   PubMed=15159550; DOI=10.1073/pnas.0305969101;
RA   Wu B., Li P., Liu Y., Lou Z., Ding Y., Shu C., Ye S., Bartlam M., Shen B.,
RA   Rao Z.;
RT   "3D structure of human FK506-binding protein 52: implications for the
RT   assembly of the glucocorticoid receptor/Hsp90/immunophilin heterocomplex.";
RL   Proc. Natl. Acad. Sci. U.S.A. 101:8348-8353(2004).
RN   [66]
RP   X-RAY CRYSTALLOGRAPHY (2.28 ANGSTROMS) OF 284-543.
RG   Structural genomics consortium (SGC);
RT   "Crystal structure of the middle domain of human hsp90-beta.";
RL   Submitted (DEC-2010) to the PDB data bank.
CC   -!- FUNCTION: Molecular chaperone that promotes the maturation, structural
CC       maintenance and proper regulation of specific target proteins involved
CC       for instance in cell cycle control and signal transduction. Undergoes a
CC       functional cycle linked to its ATPase activity. This cycle probably
CC       induces conformational changes in the client proteins, thereby causing
CC       their activation. Interacts dynamically with various co-chaperones that
CC       modulate its substrate recognition, ATPase cycle and chaperone function
CC       (PubMed:16478993, PubMed:19696785). Engages with a range of client
CC       protein classes via its interaction with various co-chaperone proteins
CC       or complexes, that act as adapters, simultaneously able to interact
CC       with the specific client and the central chaperone itself. Recruitment
CC       of ATP and co-chaperone followed by client protein forms a functional
CC       chaperone. After the completion of the chaperoning process, properly
CC       folded client protein and co-chaperone leave HSP90 in an ADP-bound
CC       partially open conformation and finally, ADP is released from HSP90
CC       which acquires an open conformation for the next cycle
CC       (PubMed:27295069, PubMed:26991466). Apart from its chaperone activity,
CC       it also plays a role in the regulation of the transcription machinery.
CC       HSP90 and its co-chaperones modulate transcription at least at three
CC       different levels. They first alter the steady-state levels of certain
CC       transcription factors in response to various physiological cues.
CC       Second, they modulate the activity of certain epigenetic modifiers,
CC       such as histone deacetylases or DNA methyl transferases, and thereby
CC       respond to the change in the environment. Third, they participate in
CC       the eviction of histones from the promoter region of certain genes and
CC       thereby turn on gene expression (PubMed:25973397). Antagonizes STUB1-
CC       mediated inhibition of TGF-beta signaling via inhibition of STUB1-
CC       mediated SMAD3 ubiquitination and degradation (PubMed:24613385).
CC       Promotes cell differentiation by chaperoning BIRC2 and thereby
CC       protecting from auto-ubiquitination and degradation by the proteasomal
CC       machinery (PubMed:18239673). Main chaperone involved in the
CC       phosphorylation/activation of the STAT1 by chaperoning both JAK2 and
CC       PRKCE under heat shock and in turn, activates its own transcription
CC       (PubMed:20353823). Involved in the translocation into ERGIC
CC       (endoplasmic reticulum-Golgi intermediate compartment) of leaderless
CC       cargos (lacking the secretion signal sequence) such as the interleukin
CC       1/IL-1; the translocation process is mediated by the cargo receptor
CC       TMED10 (PubMed:32272059). {ECO:0000269|PubMed:16478993,
CC       ECO:0000269|PubMed:18239673, ECO:0000269|PubMed:19696785,
CC       ECO:0000269|PubMed:20353823, ECO:0000269|PubMed:24613385,
CC       ECO:0000269|PubMed:32272059, ECO:0000303|PubMed:25973397,
CC       ECO:0000303|PubMed:26991466, ECO:0000303|PubMed:27295069}.
CC   -!- ACTIVITY REGULATION: In the resting state, through the dimerization of
CC       its C-terminal domain, HSP90 forms a homodimer which is defined as the
CC       open conformation. Upon ATP-binding, the N-terminal domain undergoes
CC       significant conformational changes and comes in contact to form an
CC       active closed conformation. After HSP90 finishes its chaperoning tasks
CC       of assisting the proper folding, stabilization and activation of client
CC       proteins under the active state, ATP molecule is hydrolyzed to ADP
CC       which then dissociates from HSP90 and directs the protein back to the
CC       resting state. {ECO:0000269|PubMed:18400751}.
CC   -!- BIOPHYSICOCHEMICAL PROPERTIES:
CC       Kinetic parameters:
CC         KM=300 uM for ATP {ECO:0000269|PubMed:18400751};
CC   -!- SUBUNIT: Monomer (PubMed:24880080). Homodimer (PubMed:7588731,
CC       PubMed:18400751). Forms a complex with CDK6 and CDC37 (PubMed:9482106,
CC       PubMed:25486457). Interacts with UNC45A; binding to UNC45A involves 2
CC       UNC45A monomers per HSP90AB1 dimer (PubMed:16478993). Interacts with
CC       CHORDC1 (By similarity). Interacts with DNAJC7 (PubMed:18620420).
CC       Interacts with FKBP4 (PubMed:15159550). May interact with NWD1
CC       (PubMed:24681825). Interacts with SGTA (PubMed:16580629). Interacts
CC       with HSF1 in an ATP-dependent manner. Interacts with MET; the
CC       interaction suppresses MET kinase activity. Interacts with ERBB2 in an
CC       ATP-dependent manner; the interaction suppresses ERBB2 kinase activity.
CC       Interacts with HIF1A, KEAP1 and RHOBTB2 (PubMed:26517842). Interacts
CC       with STUB1 and SMAD3 (PubMed:24613385). Interacts with XPO1 and AHSA1
CC       (PubMed:22022502, PubMed:25486457). Interacts with BIRC2
CC       (PubMed:25486457). Interacts with KCNQ4; promotes cell surface
CC       expression of KCNQ4 (PubMed:23431407). Interacts with BIRC2; prevents
CC       auto-ubiquitination and degradation of its client protein BIRC2
CC       (PubMed:18239673). Interacts with NOS3 (PubMed:23585225). Interacts
CC       with AHR; interaction is inhibited by HSP90AB1 phosphorylation on Ser-
CC       226 and Ser-255 (PubMed:15581363). Interacts with STIP1 and CDC37; upon
CC       SMYD2-dependent methylation (PubMed:24880080). Interacts with JAK2 and
CC       PRKCE; promotes functional activation in a heat shock-dependent manner
CC       (PubMed:20353823). Interacts with HSP90AA1; interaction is constitutive
CC       (PubMed:20353823). HSP90AB1-CDC37 chaperone complex interacts with
CC       inactive MAPK7 (via N-terminal half) in resting cells; the interaction
CC       is MAP2K5-independent and prevents from ubiquitination and proteasomal
CC       degradation (PubMed:23428871). Interacts with CDC25A; prevents heat
CC       shock-mediated CDC25A degradation and contributes to cell cycle
CC       progression (PubMed:22843495). Interacts with TP53 (via DNA binding
CC       domain); suppresses TP53 aggregation and prevents from irreversible
CC       thermal inactivation (PubMed:15358771). Interacts with TGFB1 processed
CC       form (LAP); inhibits latent TGFB1 activation (PubMed:20599762).
CC       Interacts with TRIM8; prevents nucleus translocation of phosphorylated
CC       STAT3 and HSP90AB1 (By similarity). Interacts with NR3C1 (via domain NR
CC       LBD) and NR1D1 (via domain NR LBD) (By similarity). Interacts with
CC       PDCL3 (By similarity). Interacts with TTC4 (via TPR repeats)
CC       (PubMed:18320024). Interacts with IL1B; the interaction facilitates
CC       cargo translocation into the ERGIC (PubMed:32272059).
CC       {ECO:0000250|UniProtKB:P11499, ECO:0000250|UniProtKB:P34058,
CC       ECO:0000269|PubMed:15159550, ECO:0000269|PubMed:15358771,
CC       ECO:0000269|PubMed:15581363, ECO:0000269|PubMed:16478993,
CC       ECO:0000269|PubMed:16580629, ECO:0000269|PubMed:18239673,
CC       ECO:0000269|PubMed:18320024, ECO:0000269|PubMed:18400751,
CC       ECO:0000269|PubMed:18620420, ECO:0000269|PubMed:20353823,
CC       ECO:0000269|PubMed:20599762, ECO:0000269|PubMed:22022502,
CC       ECO:0000269|PubMed:22843495, ECO:0000269|PubMed:23428871,
CC       ECO:0000269|PubMed:23431407, ECO:0000269|PubMed:23585225,
CC       ECO:0000269|PubMed:24613385, ECO:0000269|PubMed:24681825,
CC       ECO:0000269|PubMed:24880080, ECO:0000269|PubMed:25486457,
CC       ECO:0000269|PubMed:26517842, ECO:0000269|PubMed:32272059,
CC       ECO:0000269|PubMed:7588731, ECO:0000269|PubMed:9482106}.
CC   -!- INTERACTION:
CC       P08238; P36896: ACVR1B; NbExp=2; IntAct=EBI-352572, EBI-1384128;
CC       P08238; Q9UL18: AGO1; NbExp=3; IntAct=EBI-352572, EBI-527363;
CC       P08238; O95433: AHSA1; NbExp=4; IntAct=EBI-352572, EBI-448610;
CC       P08238; O00170: AIP; NbExp=8; IntAct=EBI-352572, EBI-704197;
CC       P08238; P31749: AKT1; NbExp=3; IntAct=EBI-352572, EBI-296087;
CC       P08238; P31751: AKT2; NbExp=2; IntAct=EBI-352572, EBI-296058;
CC       P08238; Q9UM73: ALK; NbExp=2; IntAct=EBI-352572, EBI-357361;
CC       P08238; Q16671: AMHR2; NbExp=2; IntAct=EBI-352572, EBI-6423788;
CC       P08238; Q01432: AMPD3; NbExp=2; IntAct=EBI-352572, EBI-1223554;
CC       P08238; P10398: ARAF; NbExp=8; IntAct=EBI-352572, EBI-365961;
CC       P08238; Q96GD4: AURKB; NbExp=4; IntAct=EBI-352572, EBI-624291;
CC       P08238; P30530: AXL; NbExp=3; IntAct=EBI-352572, EBI-2850927;
CC       P08238; P51451: BLK; NbExp=2; IntAct=EBI-352572, EBI-2105445;
CC       P08238; P51813: BMX; NbExp=3; IntAct=EBI-352572, EBI-696657;
CC       P08238; P15056: BRAF; NbExp=4; IntAct=EBI-352572, EBI-365980;
CC       P08238; Q06187: BTK; NbExp=2; IntAct=EBI-352572, EBI-624835;
CC       P08238; Q9UQM7: CAMK2A; NbExp=3; IntAct=EBI-352572, EBI-1383687;
CC       P08238; Q13555: CAMK2G; NbExp=2; IntAct=EBI-352572, EBI-1383465;
CC       P08238; Q16543: CDC37; NbExp=11; IntAct=EBI-352572, EBI-295634;
CC       P08238; Q7L3B6: CDC37L1; NbExp=5; IntAct=EBI-352572, EBI-2841876;
CC       P08238; Q15131: CDK10; NbExp=2; IntAct=EBI-352572, EBI-1646959;
CC       P08238; O94921: CDK14; NbExp=2; IntAct=EBI-352572, EBI-1043945;
CC       P08238; Q96Q40: CDK15; NbExp=2; IntAct=EBI-352572, EBI-1051975;
CC       P08238; Q00526: CDK3; NbExp=2; IntAct=EBI-352572, EBI-1245761;
CC       P08238; P11802: CDK4; NbExp=4; IntAct=EBI-352572, EBI-295644;
CC       P08238; Q00534: CDK6; NbExp=2; IntAct=EBI-352572, EBI-295663;
CC       P08238; P50613: CDK7; NbExp=2; IntAct=EBI-352572, EBI-1245958;
CC       P08238; P50750: CDK9; NbExp=2; IntAct=EBI-352572, EBI-1383449;
CC       P08238; O14757: CHEK1; NbExp=3; IntAct=EBI-352572, EBI-974488;
CC       P08238; Q9UHD1: CHORDC1; NbExp=6; IntAct=EBI-352572, EBI-2550959;
CC       P08238; Q9UPZ9: CILK1; NbExp=2; IntAct=EBI-352572, EBI-6381479;
CC       P08238; P49674: CSNK1E; NbExp=2; IntAct=EBI-352572, EBI-749343;
CC       P08238; Q13616: CUL1; NbExp=2; IntAct=EBI-352572, EBI-359390;
CC       P08238; Q13617: CUL2; NbExp=2; IntAct=EBI-352572, EBI-456179;
CC       P08238; Q13618: CUL3; NbExp=3; IntAct=EBI-352572, EBI-456129;
CC       P08238; Q13619: CUL4A; NbExp=2; IntAct=EBI-352572, EBI-456106;
CC       P08238; Q13620: CUL4B; NbExp=2; IntAct=EBI-352572, EBI-456067;
CC       P08238; Q16832: DDR2; NbExp=2; IntAct=EBI-352572, EBI-1381484;
CC       P08238; P00533: EGFR; NbExp=10; IntAct=EBI-352572, EBI-297353;
CC       P08238; P29317: EPHA2; NbExp=2; IntAct=EBI-352572, EBI-702104;
CC       P08238; P04626: ERBB2; NbExp=4; IntAct=EBI-352572, EBI-641062;
CC       P08238; P21860: ERBB3; NbExp=3; IntAct=EBI-352572, EBI-720706;
CC       P08238; Q15303: ERBB4; NbExp=2; IntAct=EBI-352572, EBI-80371;
CC       P08238; Q96A26: FAM162A; NbExp=3; IntAct=EBI-352572, EBI-6123466;
CC       P08238; Q9UKC9: FBXL2; NbExp=2; IntAct=EBI-352572, EBI-724253;
CC       P08238; O75426: FBXO24; NbExp=2; IntAct=EBI-352572, EBI-6425658;
CC       P08238; Q9UKT8: FBXW2; NbExp=2; IntAct=EBI-352572, EBI-914727;
CC       P08238; P11362: FGFR1; NbExp=2; IntAct=EBI-352572, EBI-1028277;
CC       P08238; P22607: FGFR3; NbExp=2; IntAct=EBI-352572, EBI-348399;
CC       P08238; P09769: FGR; NbExp=4; IntAct=EBI-352572, EBI-1383732;
CC       P08238; Q02790: FKBP4; NbExp=6; IntAct=EBI-352572, EBI-1047444;
CC       P08238; Q13451: FKBP5; NbExp=16; IntAct=EBI-352572, EBI-306914;
CC       P08238; P35916: FLT4; NbExp=2; IntAct=EBI-352572, EBI-1005467;
CC       P08238; P06241: FYN; NbExp=4; IntAct=EBI-352572, EBI-515315;
CC       P08238; P49840: GSK3A; NbExp=3; IntAct=EBI-352572, EBI-1044067;
CC       P08238; Q8TF76: HASPIN; NbExp=2; IntAct=EBI-352572, EBI-1237328;
CC       P08238; P08631: HCK; NbExp=3; IntAct=EBI-352572, EBI-346340;
CC       P08238; P08238: HSP90AB1; NbExp=4; IntAct=EBI-352572, EBI-352572;
CC       P08238; Q14164: IKBKE; NbExp=2; IntAct=EBI-352572, EBI-307369;
CC       P08238; Q9Y6K9: IKBKG; NbExp=3; IntAct=EBI-352572, EBI-81279;
CC       P08238; Q08881: ITK; NbExp=2; IntAct=EBI-352572, EBI-968552;
CC       P08238; Q2WGJ6: KLHL38; NbExp=3; IntAct=EBI-352572, EBI-6426443;
CC       P08238; P06239: LCK; NbExp=4; IntAct=EBI-352572, EBI-1348;
CC       P08238; P53671: LIMK2; NbExp=2; IntAct=EBI-352572, EBI-1384350;
CC       P08238; P07948: LYN; NbExp=2; IntAct=EBI-352572, EBI-79452;
CC       P08238; Q8N7X4: MAGEB6; NbExp=3; IntAct=EBI-352572, EBI-6447163;
CC       P08238; Q13163: MAP2K5; NbExp=3; IntAct=EBI-352572, EBI-307294;
CC       P08238; Q99558: MAP3K14; NbExp=4; IntAct=EBI-352572, EBI-358011;
CC       P08238; P41279: MAP3K8; NbExp=2; IntAct=EBI-352572, EBI-354900;
CC       P08238; P80192: MAP3K9; NbExp=2; IntAct=EBI-352572, EBI-3951604;
CC       P08238; Q92918: MAP4K1; NbExp=3; IntAct=EBI-352572, EBI-881;
CC       P08238; P31152: MAPK4; NbExp=2; IntAct=EBI-352572, EBI-3906061;
CC       P08238; P10636-8: MAPT; NbExp=11; IntAct=EBI-352572, EBI-366233;
CC       P08238; P42679: MATK; NbExp=2; IntAct=EBI-352572, EBI-751664;
CC       P08238; O15146: MUSK; NbExp=2; IntAct=EBI-352572, EBI-6423196;
CC       P08238; Q9H1R3: MYLK2; NbExp=3; IntAct=EBI-352572, EBI-356910;
CC       P08238; Q86YV6: MYLK4; NbExp=2; IntAct=EBI-352572, EBI-6424604;
CC       P08238; Q8TD19: NEK9; NbExp=2; IntAct=EBI-352572, EBI-1044009;
CC       P08238; O75469: NR1I2; NbExp=2; IntAct=EBI-352572, EBI-3905991;
CC       P08238; Q8N165: PDIK1L; NbExp=2; IntAct=EBI-352572, EBI-6423298;
CC       P08238; Q9P215: POGK; NbExp=2; IntAct=EBI-352572, EBI-2555775;
CC       P08238; P53041: PPP5C; NbExp=7; IntAct=EBI-352572, EBI-716663;
CC       P08238; Q13131: PRKAA1; NbExp=2; IntAct=EBI-352572, EBI-1181405;
CC       P08238; P22694: PRKACB; NbExp=2; IntAct=EBI-352572, EBI-2679622;
CC       P08238; Q02156: PRKCE; NbExp=4; IntAct=EBI-352572, EBI-706254;
CC       P08238; P05129: PRKCG; NbExp=2; IntAct=EBI-352572, EBI-949799;
CC       P08238; Q05513: PRKCZ; NbExp=2; IntAct=EBI-352572, EBI-295351;
CC       P08238; Q15139: PRKD1; NbExp=2; IntAct=EBI-352572, EBI-1181072;
CC       P08238; O60260: PRKN; NbExp=2; IntAct=EBI-352572, EBI-716346;
CC       P08238; P51817: PRKX; NbExp=2; IntAct=EBI-352572, EBI-4302903;
CC       P08238; P11801: PSKH1; NbExp=2; IntAct=EBI-352572, EBI-3922781;
CC       P08238; Q96QS6: PSKH2; NbExp=2; IntAct=EBI-352572, EBI-6424813;
CC       P08238; Q15185: PTGES3; NbExp=4; IntAct=EBI-352572, EBI-1049387;
CC       P08238; Q13882: PTK6; NbExp=3; IntAct=EBI-352572, EBI-1383632;
CC       P08238; P04049: RAF1; NbExp=5; IntAct=EBI-352572, EBI-365996;
CC       P08238; P49758: RGS6; NbExp=2; IntAct=EBI-352572, EBI-6426927;
CC       P08238; Q01974: ROR2; NbExp=2; IntAct=EBI-352572, EBI-6422642;
CC       P08238; P62913: RPL11; NbExp=2; IntAct=EBI-352572, EBI-354380;
CC       P08238; Q15418: RPS6KA1; NbExp=4; IntAct=EBI-352572, EBI-963034;
CC       P08238; P51812: RPS6KA3; NbExp=3; IntAct=EBI-352572, EBI-1046616;
CC       P08238; P31948: STIP1; NbExp=4; IntAct=EBI-352572, EBI-1054052;
CC       P08238; Q15831: STK11; NbExp=4; IntAct=EBI-352572, EBI-306838;
CC       P08238; Q15208: STK38; NbExp=2; IntAct=EBI-352572, EBI-458376;
CC       P08238; Q9UNE7: STUB1; NbExp=5; IntAct=EBI-352572, EBI-357085;
CC       P08238; Q9Y2Z0-2: SUGT1; NbExp=2; IntAct=EBI-352572, EBI-10768076;
CC       P08238; Q9UHD2: TBK1; NbExp=2; IntAct=EBI-352572, EBI-356402;
CC       P08238; Q96S53: TESK2; NbExp=2; IntAct=EBI-352572, EBI-1384110;
CC       P08238; Q07912: TNK2; NbExp=3; IntAct=EBI-352572, EBI-603457;
CC       P08238; Q9BXA6: TSSK6; NbExp=3; IntAct=EBI-352572, EBI-851883;
CC       P08238; O95801: TTC4; NbExp=5; IntAct=EBI-352572, EBI-1050890;
CC       P08238; P29597: TYK2; NbExp=2; IntAct=EBI-352572, EBI-1383454;
CC       P08238; Q8IWX7: UNC45B; NbExp=2; IntAct=EBI-352572, EBI-9363363;
CC       P08238; P07947: YES1; NbExp=3; IntAct=EBI-352572, EBI-515331;
CC   -!- SUBCELLULAR LOCATION: Cytoplasm {ECO:0000269|PubMed:16580629,
CC       ECO:0000269|PubMed:18239673, ECO:0000269|PubMed:24880080,
CC       ECO:0000269|PubMed:9482106}. Melanosome {ECO:0000269|PubMed:17081065}.
CC       Nucleus {ECO:0000269|PubMed:18239673}. Secreted
CC       {ECO:0000269|PubMed:20599762}. Cell membrane
CC       {ECO:0000269|PubMed:20599762}. Dynein axonemal particle
CC       {ECO:0000250|UniProtKB:Q6AZV1}. Note=Identified by mass spectrometry in
CC       melanosome fractions from stage I to stage IV (PubMed:17081065).
CC       Translocates with BIRC2 from the nucleus to the cytoplasm during
CC       differentiation (PubMed:18239673). Secreted when associated with TGFB1
CC       processed form (LAP) (PubMed:20599762). {ECO:0000269|PubMed:17081065,
CC       ECO:0000269|PubMed:18239673, ECO:0000269|PubMed:20599762}.
CC   -!- INDUCTION: By heat shock. {ECO:0000269|PubMed:20353823}.
CC   -!- DOMAIN: The TPR repeat-binding motif mediates interaction with TPR
CC       repeat-containing proteins. {ECO:0000250|UniProtKB:P07900}.
CC   -!- PTM: Ubiquitinated in the presence of STUB1-UBE2D1 complex (in vitro).
CC       {ECO:0000269|PubMed:18042044}.
CC   -!- PTM: ISGylated. {ECO:0000269|PubMed:16139798}.
CC   -!- PTM: S-nitrosylated; negatively regulates the ATPase activity.
CC       {ECO:0000305|PubMed:19696785}.
CC   -!- PTM: Phosphorylation at Tyr-301 by SRC is induced by lipopolysaccharide
CC       (PubMed:23585225). Phosphorylation at Ser-226 and Ser-255 inhibits AHR
CC       interaction (PubMed:15581363). {ECO:0000269|PubMed:15581363,
CC       ECO:0000269|PubMed:23585225}.
CC   -!- PTM: Methylated by SMYD2; facilitates dimerization and chaperone
CC       complex formation; promotes cancer cell proliferation.
CC       {ECO:0000269|PubMed:24880080}.
CC   -!- PTM: Cleaved following oxidative stress resulting in HSP90AB1 protein
CC       radicals formation; disrupts the chaperoning function and the
CC       degradation of its client proteins. {ECO:0000269|PubMed:22848402}.
CC   -!- SIMILARITY: Belongs to the heat shock protein 90 family. {ECO:0000305}.
CC   -!- SEQUENCE CAUTION:
CC       Sequence=AAD14062.3; Type=Erroneous initiation; Note=Truncated N-terminus.; Evidence={ECO:0000305};
CC       Sequence=CAB66478.1; Type=Frameshift; Evidence={ECO:0000305};
CC   ---------------------------------------------------------------------------
CC   Copyrighted by the UniProt Consortium, see https://www.uniprot.org/terms
CC   Distributed under the Creative Commons Attribution (CC BY 4.0) License
CC   ---------------------------------------------------------------------------
DR   EMBL; M16660; AAA36025.1; -; mRNA.
DR   EMBL; J04988; AAA36026.1; -; Genomic_DNA.
DR   EMBL; AY359878; AAQ63401.1; -; mRNA.
DR   EMBL; AL136543; CAB66478.1; ALT_FRAME; mRNA.
DR   EMBL; AK312255; BAG35187.1; -; mRNA.
DR   EMBL; DQ314872; ABC40731.1; -; Genomic_DNA.
DR   EMBL; AL139392; -; NOT_ANNOTATED_CDS; Genomic_DNA.
DR   EMBL; CH471081; EAX04257.1; -; Genomic_DNA.
DR   EMBL; BC004928; AAH04928.1; -; mRNA.
DR   EMBL; BC009206; AAH09206.2; -; mRNA.
DR   EMBL; BC012807; AAH12807.1; -; mRNA.
DR   EMBL; BC014485; AAH14485.1; -; mRNA.
DR   EMBL; BC016753; AAH16753.1; -; mRNA.
DR   EMBL; BC068474; AAH68474.1; -; mRNA.
DR   EMBL; AH007358; AAD14062.3; ALT_INIT; Genomic_DNA.
DR   EMBL; AF275719; AAF82792.1; -; mRNA.
DR   CCDS; CCDS4909.1; -.
DR   PIR; A29461; HHHU84.
DR   PIR; T46243; T46243.
DR   RefSeq; NP_001258898.1; NM_001271969.1.
DR   RefSeq; NP_001258899.1; NM_001271970.1.
DR   RefSeq; NP_001258900.1; NM_001271971.1.
DR   RefSeq; NP_031381.2; NM_007355.3.
DR   PDB; 1QZ2; X-ray; 3.00 A; G/H=720-724.
DR   PDB; 1UYM; X-ray; 2.45 A; A=2-221.
DR   PDB; 2L6J; NMR; -; B=720-724.
DR   PDB; 3FWV; X-ray; 2.20 A; C/D=719-723.
DR   PDB; 3NMQ; X-ray; 2.20 A; A=1-223.
DR   PDB; 3PRY; X-ray; 2.28 A; A/B/C=284-543.
DR   PDB; 3UQ3; X-ray; 2.60 A; B/C=720-724.
DR   PDB; 5FWK; EM; 3.90 A; A/B=1-724.
DR   PDB; 5FWL; EM; 9.00 A; A/B=1-724.
DR   PDB; 5FWM; EM; 8.00 A; A/B=1-724.
DR   PDB; 5FWP; EM; 7.20 A; A/B=1-724.
DR   PDB; 5UC4; X-ray; 2.05 A; A/B/C/D=1-218.
DR   PDB; 5UCH; X-ray; 2.65 A; A/B/C/D=1-218.
DR   PDB; 5UCI; X-ray; 2.70 A; A/B/C/D=1-218.
DR   PDB; 5UCJ; X-ray; 1.69 A; A/B/C/D=1-218.
DR   PDB; 6N8W; X-ray; 3.09 A; A/B/C/D=1-231.
DR   PDB; 6N8Y; X-ray; 1.55 A; A=1-221.
DR   PDBsum; 1QZ2; -.
DR   PDBsum; 1UYM; -.
DR   PDBsum; 2L6J; -.
DR   PDBsum; 3FWV; -.
DR   PDBsum; 3NMQ; -.
DR   PDBsum; 3PRY; -.
DR   PDBsum; 3UQ3; -.
DR   PDBsum; 5FWK; -.
DR   PDBsum; 5FWL; -.
DR   PDBsum; 5FWM; -.
DR   PDBsum; 5FWP; -.
DR   PDBsum; 5UC4; -.
DR   PDBsum; 5UCH; -.
DR   PDBsum; 5UCI; -.
DR   PDBsum; 5UCJ; -.
DR   PDBsum; 6N8W; -.
DR   PDBsum; 6N8Y; -.
DR   SMR; P08238; -.
DR   BioGRID; 109558; 525.
DR   ComplexPortal; CPX-3285; HSP90B-CDC37 chaperone complex.
DR   CORUM; P08238; -.
DR   DIP; DIP-413N; -.
DR   IntAct; P08238; 566.
DR   MINT; P08238; -.
DR   STRING; 9606.ENSP00000360609; -.
DR   BindingDB; P08238; -.
DR   ChEMBL; CHEMBL4303; -.
DR   DrugBank; DB08293; (5E)-12-CHLORO-13,15-DIHYDROXY-4,7,8,9-TETRAHYDRO-2-BENZOXACYCLOTRIDECINE-1,10(3H,11H)-DIONE.
DR   DrugBank; DB08153; (5E)-14-CHLORO-15,17-DIHYDROXY-4,7,8,9,10,11-HEXAHYDRO-2-BENZOXACYCLOPENTADECINE-1,12(3H,13H)-DIONE.
DR   DrugBank; DB08292; (5Z)-12-CHLORO-13,15-DIHYDROXY-4,7,8,9-TETRAHYDRO-2-BENZOXACYCLOTRIDECINE-1,10(3H,11H)-DIONE.
DR   DrugBank; DB08346; (5Z)-13-CHLORO-14,16-DIHYDROXY-3,4,7,8,9,10-HEXAHYDRO-1H-2-BENZOXACYCLOTETRADECINE-1,11(12H)-DIONE.
DR   DrugBank; DB08465; 2-(3-AMINO-2,5,6-TRIMETHOXYPHENYL)ETHYL 5-CHLORO-2,4-DIHYDROXYBENZOATE.
DR   DrugBank; DB08045; 4-{4-[4-(3-AMINOPROPOXY)PHENYL]-1H-PYRAZOL-5-YL}-6-CHLOROBENZENE-1,3-DIOL.
DR   DrugBank; DB07877; 8-(6-BROMO-BENZO[1,3]DIOXOL-5-YLSULFANYL)-9-(3-ISOPROPYLAMINO-PROPYL)-ADENINE.
DR   DrugBank; DB02754; 9-Butyl-8-(3,4,5-Trimethoxybenzyl)-9h-Purin-6-Amine.
DR   DrugBank; DB07594; CCT-018159.
DR   DrugBank; DB02424; Geldanamycin.
DR   DrugBank; DB08464; METHYL 3-CHLORO-2-{3-[(2,5-DIHYDROXY-4-METHOXYPHENYL)AMINO]-3-OXOPROPYL}-4,6-DIHYDROXYBENZOATE.
DR   DrugBank; DB09221; Polaprezinc.
DR   DrugBank; DB03758; Radicicol.
DR   DrugBank; DB06070; SNX-5422.
DR   DrugBank; DB05134; Tanespimycin.
DR   GuidetoPHARMACOLOGY; 2907; -.
DR   MoonDB; P08238; Predicted.
DR   CarbonylDB; P08238; -.
DR   GlyConnect; 1302; 2 N-Linked glycans (2 sites).
DR   GlyGen; P08238; 5 sites, 1 O-linked glycan (1 site).
DR   iPTMnet; P08238; -.
DR   MetOSite; P08238; -.
DR   PhosphoSitePlus; P08238; -.
DR   SwissPalm; P08238; -.
DR   BioMuta; HSP90AB1; -.
DR   DMDM; 17865718; -.
DR   OGP; P08238; -.
DR   EPD; P08238; -.
DR   jPOST; P08238; -.
DR   MassIVE; P08238; -.
DR   MaxQB; P08238; -.
DR   PaxDb; P08238; -.
DR   PeptideAtlas; P08238; -.
DR   PRIDE; P08238; -.
DR   ProteomicsDB; 52096; -.
DR   TopDownProteomics; P08238; -.
DR   ABCD; P08238; 1 sequenced antibody.
DR   Antibodypedia; 3929; 1709 antibodies.
DR   DNASU; 3326; -.
DR   Ensembl; ENST00000353801; ENSP00000325875; ENSG00000096384.
DR   Ensembl; ENST00000371554; ENSP00000360609; ENSG00000096384.
DR   Ensembl; ENST00000371646; ENSP00000360709; ENSG00000096384.
DR   Ensembl; ENST00000620073; ENSP00000481908; ENSG00000096384.
DR   GeneID; 3326; -.
DR   KEGG; hsa:3326; -.
DR   UCSC; uc003oxa.3; human.
DR   CTD; 3326; -.
DR   DisGeNET; 3326; -.
DR   GeneCards; HSP90AB1; -.
DR   HGNC; HGNC:5258; HSP90AB1.
DR   HPA; ENSG00000096384; Low tissue specificity.
DR   MIM; 140572; gene.
DR   neXtProt; NX_P08238; -.
DR   OpenTargets; ENSG00000096384; -.
DR   PharmGKB; PA29524; -.
DR   VEuPathDB; HostDB:ENSG00000096384.19; -.
DR   eggNOG; KOG0019; Eukaryota.
DR   GeneTree; ENSGT01020000230401; -.
DR   HOGENOM; CLU_006684_1_3_1; -.
DR   InParanoid; P08238; -.
DR   OMA; IMDNCEQ; -.
DR   OrthoDB; 924636at2759; -.
DR   PhylomeDB; P08238; -.
DR   TreeFam; TF300686; -.
DR   BRENDA; 3.6.4.10; 2681.
DR   PathwayCommons; P08238; -.
DR   Reactome; R-HSA-2029482; Regulation of actin dynamics for phagocytic cup formation.
DR   Reactome; R-HSA-3371497; HSP90 chaperone cycle for steroid hormone receptors (SHR).
DR   Reactome; R-HSA-3371511; HSF1 activation.
DR   Reactome; R-HSA-3371568; Attenuation phase.
DR   Reactome; R-HSA-3371571; HSF1-dependent transactivation.
DR   Reactome; R-HSA-399954; Sema3A PAK dependent Axon repulsion.
DR   Reactome; R-HSA-5336415; Uptake and function of diphtheria toxin.
DR   Reactome; R-HSA-6798695; Neutrophil degranulation.
DR   Reactome; R-HSA-844456; The NLRP3 inflammasome.
DR   Reactome; R-HSA-8852276; The role of GTSE1 in G2/M progression after G2 checkpoint.
DR   Reactome; R-HSA-8937144; Aryl hydrocarbon receptor signalling.
DR   Reactome; R-HSA-8939211; ESR-mediated signaling.
DR   Reactome; R-HSA-9013418; RHOBTB2 GTPase cycle.
DR   Reactome; R-HSA-9018519; Estrogen-dependent gene expression.
DR   Reactome; R-HSA-9613829; Chaperone Mediated Autophagy.
DR   Reactome; R-HSA-9660826; Purinergic signaling in leishmaniasis infection.
DR   Reactome; R-HSA-9679191; Potential therapeutics for SARS.
DR   SIGNOR; P08238; -.
DR   BioGRID-ORCS; 3326; 109 hits in 1017 CRISPR screens.
DR   ChiTaRS; HSP90AB1; human.
DR   EvolutionaryTrace; P08238; -.
DR   GeneWiki; HSP90AB1; -.
DR   GenomeRNAi; 3326; -.
DR   Pharos; P08238; Tchem.
DR   PRO; PR:P08238; -.
DR   Proteomes; UP000005640; Chromosome 6.
DR   RNAct; P08238; protein.
DR   Bgee; ENSG00000096384; Expressed in hypothalamus and 252 other tissues.
DR   ExpressionAtlas; P08238; baseline and differential.
DR   Genevisible; P08238; HS.
DR   GO; GO:0016324; C:apical plasma membrane; IEA:Ensembl.
DR   GO; GO:0034751; C:aryl hydrocarbon receptor complex; IDA:UniProtKB.
DR   GO; GO:0044295; C:axonal growth cone; ISS:ARUK-UCL.
DR   GO; GO:0016323; C:basolateral plasma membrane; IEA:Ensembl.
DR   GO; GO:0031526; C:brush border membrane; IEA:Ensembl.
DR   GO; GO:0009986; C:cell surface; IEA:Ensembl.
DR   GO; GO:0005737; C:cytoplasm; IDA:UniProtKB.
DR   GO; GO:0005829; C:cytosol; IDA:HPA.
DR   GO; GO:0044294; C:dendritic growth cone; ISS:ARUK-UCL.
DR   GO; GO:0120293; C:dynein axonemal particle; ISS:UniProtKB.
DR   GO; GO:0070062; C:extracellular exosome; HDA:UniProtKB.
DR   GO; GO:0005576; C:extracellular region; IDA:UniProtKB.
DR   GO; GO:1904813; C:ficolin-1-rich granule lumen; TAS:Reactome.
DR   GO; GO:1990565; C:HSP90-CDC37 chaperone complex; IDA:ParkinsonsUK-UCL.
DR   GO; GO:0016234; C:inclusion body; IEA:Ensembl.
DR   GO; GO:0005765; C:lysosomal membrane; IEA:Ensembl.
DR   GO; GO:0042470; C:melanosome; IEA:UniProtKB-SubCell.
DR   GO; GO:0016020; C:membrane; HDA:UniProtKB.
DR   GO; GO:0005739; C:mitochondrion; HDA:UniProtKB.
DR   GO; GO:0043025; C:neuronal cell body; ISS:ARUK-UCL.
DR   GO; GO:0005654; C:nucleoplasm; TAS:Reactome.
DR   GO; GO:0005634; C:nucleus; IDA:UniProtKB.
DR   GO; GO:1990917; C:ooplasm; IEA:Ensembl.
DR   GO; GO:0048471; C:perinuclear region of cytoplasm; ISS:ARUK-UCL.
DR   GO; GO:0005886; C:plasma membrane; IBA:GO_Central.
DR   GO; GO:0032991; C:protein-containing complex; IDA:UniProtKB.
DR   GO; GO:0034774; C:secretory granule lumen; TAS:Reactome.
DR   GO; GO:1990913; C:sperm head plasma membrane; IEA:Ensembl.
DR   GO; GO:0005524; F:ATP binding; IDA:CAFA.
DR   GO; GO:0043008; F:ATP-dependent protein binding; IPI:CAFA.
DR   GO; GO:0016887; F:ATPase activity; IEA:InterPro.
DR   GO; GO:0045296; F:cadherin binding; HDA:BHF-UCL.
DR   GO; GO:0002135; F:CTP binding; IEA:Ensembl.
DR   GO; GO:0032564; F:dATP binding; IEA:Ensembl.
DR   GO; GO:0097718; F:disordered domain specific binding; IPI:CAFA.
DR   GO; GO:0070182; F:DNA polymerase binding; IPI:BHF-UCL.
DR   GO; GO:0003725; F:double-stranded RNA binding; IDA:MGI.
DR   GO; GO:0005525; F:GTP binding; IEA:Ensembl.
DR   GO; GO:0031072; F:heat shock protein binding; IPI:UniProtKB.
DR   GO; GO:0042826; F:histone deacetylase binding; IPI:BHF-UCL.
DR   GO; GO:1990226; F:histone methyltransferase binding; IPI:UniProtKB.
DR   GO; GO:0042802; F:identical protein binding; IPI:IntAct.
DR   GO; GO:0044325; F:ion channel binding; IEA:Ensembl.
DR   GO; GO:0019900; F:kinase binding; IPI:UniProtKB.
DR   GO; GO:0023026; F:MHC class II protein complex binding; HDA:UniProtKB.
DR   GO; GO:0030235; F:nitric-oxide synthase regulator activity; ISS:UniProtKB.
DR   GO; GO:0042277; F:peptide binding; IPI:UniProtKB.
DR   GO; GO:0046983; F:protein dimerization activity; IDA:UniProtKB.
DR   GO; GO:0044183; F:protein folding chaperone; IEA:Ensembl.
DR   GO; GO:0042803; F:protein homodimerization activity; IDA:CAFA.
DR   GO; GO:0019901; F:protein kinase binding; IEA:Ensembl.
DR   GO; GO:0019887; F:protein kinase regulator activity; IEA:Ensembl.
DR   GO; GO:0003723; F:RNA binding; HDA:UniProtKB.
DR   GO; GO:0017098; F:sulfonylurea receptor binding; IEA:Ensembl.
DR   GO; GO:0048156; F:tau protein binding; NAS:ARUK-UCL.
DR   GO; GO:0030911; F:TPR domain binding; ISS:UniProtKB.
DR   GO; GO:0031625; F:ubiquitin protein ligase binding; IPI:ARUK-UCL.
DR   GO; GO:0051082; F:unfolded protein binding; IBA:GO_Central.
DR   GO; GO:0002134; F:UTP binding; IEA:Ensembl.
DR   GO; GO:0048675; P:axon extension; ISS:ARUK-UCL.
DR   GO; GO:0034605; P:cellular response to heat; IBA:GO_Central.
DR   GO; GO:0071353; P:cellular response to interleukin-4; IEA:Ensembl.
DR   GO; GO:0071407; P:cellular response to organic cyclic compound; IEA:Ensembl.
DR   GO; GO:0021955; P:central nervous system neuron axonogenesis; ISS:ARUK-UCL.
DR   GO; GO:0051131; P:chaperone-mediated protein complex assembly; IDA:CAFA.
DR   GO; GO:0030010; P:establishment of cell polarity; ISS:ARUK-UCL.
DR   GO; GO:0038096; P:Fc-gamma receptor signaling pathway involved in phagocytosis; TAS:Reactome.
DR   GO; GO:0071157; P:negative regulation of cell cycle arrest; IMP:UniProtKB.
DR   GO; GO:1903660; P:negative regulation of complement-dependent cytotoxicity; IEA:Ensembl.
DR   GO; GO:0043524; P:negative regulation of neuron apoptotic process; IEA:Ensembl.
DR   GO; GO:1901799; P:negative regulation of proteasomal protein catabolic process; ISS:ARUK-UCL.
DR   GO; GO:0032435; P:negative regulation of proteasomal ubiquitin-dependent protein catabolic process; IDA:UniProtKB.
DR   GO; GO:0051248; P:negative regulation of protein metabolic process; ISS:ARUK-UCL.
DR   GO; GO:1901389; P:negative regulation of transforming growth factor beta activation; IDA:UniProtKB.
DR   GO; GO:0043312; P:neutrophil degranulation; TAS:Reactome.
DR   GO; GO:0001890; P:placenta development; IEA:Ensembl.
DR   GO; GO:0045597; P:positive regulation of cell differentiation; IMP:UniProtKB.
DR   GO; GO:0045793; P:positive regulation of cell size; IEA:Ensembl.
DR   GO; GO:1904031; P:positive regulation of cyclin-dependent protein kinase activity; ISS:ARUK-UCL.
DR   GO; GO:0045429; P:positive regulation of nitric oxide biosynthetic process; ISS:UniProtKB.
DR   GO; GO:0033138; P:positive regulation of peptidyl-serine phosphorylation; ISS:ARUK-UCL.
DR   GO; GO:0032516; P:positive regulation of phosphoprotein phosphatase activity; IDA:UniProtKB.
DR   GO; GO:0032092; P:positive regulation of protein binding; IEA:Ensembl.
DR   GO; GO:0042307; P:positive regulation of protein import into nucleus; IEA:Ensembl.
DR   GO; GO:0051897; P:positive regulation of protein kinase B signaling; ISS:ARUK-UCL.
DR   GO; GO:2000010; P:positive regulation of protein localization to cell surface; IDA:UniProtKB.
DR   GO; GO:0071902; P:positive regulation of protein serine/threonine kinase activity; IEA:Ensembl.
DR   GO; GO:1902949; P:positive regulation of tau-protein kinase activity; IC:ARUK-UCL.
DR   GO; GO:0051973; P:positive regulation of telomerase activity; IDA:BHF-UCL.
DR   GO; GO:0030511; P:positive regulation of transforming growth factor beta receptor signaling pathway; IDA:UniProtKB.
DR   GO; GO:0006457; P:protein folding; IBA:GO_Central.
DR   GO; GO:0050821; P:protein stabilization; IBA:GO_Central.
DR   GO; GO:0035590; P:purinergic nucleotide receptor signaling pathway; TAS:Reactome.
DR   GO; GO:1903827; P:regulation of cellular protein localization; ISS:ARUK-UCL.
DR   GO; GO:1900034; P:regulation of cellular response to heat; TAS:Reactome.
DR   GO; GO:0060334; P:regulation of interferon-gamma-mediated signaling pathway; IMP:MGI.
DR   GO; GO:0031396; P:regulation of protein ubiquitination; IDA:BHF-UCL.
DR   GO; GO:0060338; P:regulation of type I interferon-mediated signaling pathway; IMP:MGI.
DR   GO; GO:0042220; P:response to cocaine; IEA:Ensembl.
DR   GO; GO:0042493; P:response to drug; IEA:Ensembl.
DR   GO; GO:0009651; P:response to salt stress; IEA:Ensembl.
DR   GO; GO:0006986; P:response to unfolded protein; NAS:UniProtKB.
DR   GO; GO:0097435; P:supramolecular fiber organization; IMP:CACAO.
DR   GO; GO:1905323; P:telomerase holoenzyme complex assembly; IDA:BHF-UCL.
DR   GO; GO:0007004; P:telomere maintenance via telomerase; IDA:BHF-UCL.
DR   GO; GO:0019062; P:virion attachment to host cell; IMP:CACAO.
DR   GO; GO:0006805; P:xenobiotic metabolic process; TAS:Reactome.
DR   Gene3D; 1.20.120.790; -; 1.
DR   Gene3D; 3.30.565.10; -; 1.
DR   HAMAP; MF_00505; HSP90; 1.
DR   IDEAL; IID00536; -.
DR   InterPro; IPR003594; HATPase_C.
DR   InterPro; IPR036890; HATPase_C_sf.
DR   InterPro; IPR019805; Heat_shock_protein_90_CS.
DR   InterPro; IPR037196; HSP90_C.
DR   InterPro; IPR001404; Hsp90_fam.
DR   InterPro; IPR020575; Hsp90_N.
DR   InterPro; IPR020568; Ribosomal_S5_D2-typ_fold.
DR   PANTHER; PTHR11528; PTHR11528; 1.
DR   Pfam; PF02518; HATPase_c; 1.
DR   Pfam; PF00183; HSP90; 1.
DR   PIRSF; PIRSF002583; Hsp90; 1.
DR   PRINTS; PR00775; HEATSHOCK90.
DR   SMART; SM00387; HATPase_c; 1.
DR   SUPFAM; SSF110942; SSF110942; 1.
DR   SUPFAM; SSF54211; SSF54211; 1.
DR   SUPFAM; SSF55874; SSF55874; 1.
DR   PROSITE; PS00298; HSP90; 1.
PE   1: Evidence at protein level;
KW   3D-structure; Acetylation; ATP-binding; Cell membrane; Chaperone;
KW   Cytoplasm; Direct protein sequencing; Glycoprotein; Membrane; Methylation;
KW   Nucleotide-binding; Nucleus; Phosphoprotein; Reference proteome;
KW   S-nitrosylation; Secreted; Stress response; Ubl conjugation.
FT   INIT_MET        1
FT                   /note="Removed"
FT                   /evidence="ECO:0000269|PubMed:2492519"
FT   CHAIN           2..724
FT                   /note="Heat shock protein HSP 90-beta"
FT                   /id="PRO_0000062917"
FT   REGION          2..527
FT                   /note="Interaction with TP53"
FT                   /evidence="ECO:0000269|PubMed:15358771"
FT   REGION          2..214
FT                   /note="Interaction with BIRC2"
FT                   /evidence="ECO:0000269|PubMed:25486457"
FT   REGION          9..231
FT                   /note="Interaction with NR3C1"
FT                   /evidence="ECO:0000250|UniProtKB:P11499"
FT   REGION          215..552
FT                   /note="Interaction with AHSA1"
FT                   /evidence="ECO:0000269|PubMed:25486457"
FT   REGION          222..270
FT                   /note="Disordered"
FT                   /evidence="ECO:0000256|SAM:MobiDB-lite"
FT   REGION          264..608
FT                   /note="Interaction with NR3C1"
FT                   /evidence="ECO:0000250|UniProtKB:P11499"
FT   REGION          620..723
FT                   /note="Interaction with NR1D1"
FT                   /evidence="ECO:0000250|UniProtKB:P11499"
FT   REGION          696..724
FT                   /note="Disordered"
FT                   /evidence="ECO:0000256|SAM:MobiDB-lite"
FT   MOTIF           720..724
FT                   /note="TPR repeat-binding"
FT   COMPBIAS        241..270
FT                   /note="Basic and acidic residues"
FT                   /evidence="ECO:0000256|SAM:MobiDB-lite"
FT   BINDING         46
FT                   /note="ATP"
FT                   /evidence="ECO:0000250"
FT   BINDING         88
FT                   /note="ATP"
FT   BINDING         107
FT                   /note="ATP"
FT                   /evidence="ECO:0000250"
FT   BINDING         133
FT                   /note="ATP; via amide nitrogen"
FT                   /evidence="ECO:0000250"
FT   BINDING         392
FT                   /note="ATP"
FT                   /evidence="ECO:0000250"
FT   SITE            126..127
FT                   /note="Cleaved under oxidative stress"
FT                   /evidence="ECO:0000269|PubMed:22848402"
FT   MOD_RES         219
FT                   /note="N6-succinyllysine"
FT                   /evidence="ECO:0000250|UniProtKB:P11499"
FT   MOD_RES         226
FT                   /note="Phosphoserine"
FT                   /evidence="ECO:0007744|PubMed:18088087,
FT                   ECO:0007744|PubMed:18318008, ECO:0007744|PubMed:20068231,
FT                   ECO:0007744|PubMed:21406692, ECO:0007744|PubMed:23186163"
FT   MOD_RES         255
FT                   /note="Phosphoserine"
FT                   /evidence="ECO:0000269|Ref.12, ECO:0007744|PubMed:17081983,
FT                   ECO:0007744|PubMed:23186163"
FT   MOD_RES         261
FT                   /note="Phosphoserine"
FT                   /evidence="ECO:0000250|UniProtKB:P11499"
FT   MOD_RES         297
FT                   /note="Phosphothreonine"
FT                   /evidence="ECO:0007744|PubMed:17525332,
FT                   ECO:0007744|PubMed:23186163"
FT   MOD_RES         301
FT                   /note="Phosphotyrosine; by SRC"
FT                   /evidence="ECO:0000269|PubMed:23585225"
FT   MOD_RES         305
FT                   /note="Phosphotyrosine"
FT                   /evidence="ECO:0000250|UniProtKB:P11499"
FT   MOD_RES         307
FT                   /note="Phosphoserine"
FT                   /evidence="ECO:0007744|PubMed:18669648"
FT   MOD_RES         399
FT                   /note="N6-malonyllysine"
FT                   /evidence="ECO:0000269|PubMed:21908771"
FT   MOD_RES         435
FT                   /note="N6-acetyllysine"
FT                   /evidence="ECO:0007744|PubMed:19608861"
FT   MOD_RES         445
FT                   /note="Phosphoserine"
FT                   /evidence="ECO:0007744|PubMed:23186163"
FT   MOD_RES         479
FT                   /note="Phosphothreonine"
FT                   /evidence="ECO:0007744|PubMed:23186163"
FT   MOD_RES         481
FT                   /note="N6-acetyllysine"
FT                   /evidence="ECO:0007744|PubMed:19608861"
FT   MOD_RES         484
FT                   /note="Phosphotyrosine"
FT                   /evidence="ECO:0000250|UniProtKB:P11499"
FT   MOD_RES         531
FT                   /note="N6-methylated lysine; alternate"
FT                   /evidence="ECO:0000269|PubMed:24880080"
FT   MOD_RES         531
FT                   /note="N6-succinyllysine; alternate"
FT                   /evidence="ECO:0000250|UniProtKB:P11499"
FT   MOD_RES         574
FT                   /note="N6-methylated lysine"
FT                   /evidence="ECO:0000269|PubMed:24880080"
FT   MOD_RES         577
FT                   /note="N6-succinyllysine"
FT                   /evidence="ECO:0000250|UniProtKB:P11499"
FT   MOD_RES         590
FT                   /note="S-nitrosocysteine"
FT                   /evidence="ECO:0000305|PubMed:19696785"
FT   MOD_RES         624
FT                   /note="N6-acetyllysine"
FT                   /evidence="ECO:0000250|UniProtKB:P11499"
FT   MOD_RES         669
FT                   /note="Phosphoserine"
FT                   /evidence="ECO:0007744|PubMed:24275569"
FT   MOD_RES         718
FT                   /note="Phosphoserine; by PLK2 and PLK3"
FT                   /evidence="ECO:0000269|PubMed:22828320"
FT   CARBOHYD        434
FT                   /note="O-linked (GlcNAc) serine"
FT                   /evidence="ECO:0000250"
FT   CARBOHYD        452
FT                   /note="O-linked (GlcNAc) serine"
FT                   /evidence="ECO:0000250"
FT   VARIANT         349
FT                   /note="K -> E (in dbSNP:rs11538975)"
FT                   /id="VAR_049624"
FT   MUTAGEN         42
FT                   /note="E->A: Strong ATP-binding. Strong interaction with
FT                   HSF1, HIF1A, ERBB2, MET, KEAP1 and RHOBTB2."
FT                   /evidence="ECO:0000269|PubMed:26517842"
FT   MUTAGEN         88
FT                   /note="D->A: Impaired ATP-binding. Strong interaction with
FT                   HIF1A, MET, KEAP1 and RHOBTB2. Loss of interaction with
FT                   HSF1 and ERBB2."
FT                   /evidence="ECO:0000269|PubMed:26517842"
FT   MUTAGEN         226
FT                   /note="S->A: Increases the binding affinity for AHR; when
FT                   associated with A-255. Increases AHR transcription
FT                   activity; when associated with A-255."
FT                   /evidence="ECO:0000269|PubMed:15581363"
FT   MUTAGEN         226
FT                   /note="S->E: No effect on the interaction with AHR; when
FT                   associated with E-255."
FT                   /evidence="ECO:0000269|PubMed:15581363"
FT   MUTAGEN         255
FT                   /note="S->A: Increases the binding affinity for AHR; when
FT                   associated with A-226. Increases AHR transcription
FT                   activity; when associated with A-226."
FT                   /evidence="ECO:0000269|PubMed:15581363"
FT   MUTAGEN         255
FT                   /note="S->E: No effect on the interaction with AHR; when
FT                   associated with E-226."
FT                   /evidence="ECO:0000269|PubMed:15581363"
FT   MUTAGEN         301
FT                   /note="Y->F: Decreases interaction with NOS3 and SRC.
FT                   impairs resists LPS-induced tyrosine phosphorylation. Does
FT                   not block LPS-induced pp60src phosphorylation."
FT                   /evidence="ECO:0000269|PubMed:23585225"
FT   MUTAGEN         531
FT                   /note="K->A: Highly decreases the signal of SMYD2-dependent
FT                   HSP90AB1 methylation; when associated with A-574.
FT                   Diminishes dimerized form; when associated with A-574.
FT                   Reduces interaction with STIP1 or CDC37; when associated
FT                   with A-574."
FT                   /evidence="ECO:0000269|PubMed:24880080"
FT   MUTAGEN         574
FT                   /note="K->A: Decreases the signal of SMYD2-dependent
FT                   HSP90AB1 methylation. Highly decreases the signal of SMYD2-
FT                   dependent HSP90AB1 methylation; when associated with A-531.
FT                   Diminishes dimerized form; when associated with A-531.
FT                   Reduces interaction with STIP1 or CDC37; when associated
FT                   with A-531."
FT                   /evidence="ECO:0000269|PubMed:24880080"
FT   MUTAGEN         590
FT                   /note="C->A,N,D: Reduced ATPase activity and client protein
FT                   activation."
FT                   /evidence="ECO:0000269|PubMed:19696785"
FT   CONFLICT        147
FT                   /note="T -> R (in Ref. 1; AAA36025)"
FT                   /evidence="ECO:0000305"
FT   CONFLICT        177
FT                   /note="R -> M (in Ref. 1; AAA36025)"
FT                   /evidence="ECO:0000305"
FT   CONFLICT        403
FT                   /note="V -> A (in Ref. 5; CAB66478)"
FT                   /evidence="ECO:0000305"
FT   STRAND          2..6
FT                   /evidence="ECO:0007829|PDB:6N8W"
FT   STRAND          13..16
FT                   /evidence="ECO:0007829|PDB:6N8Y"
FT   HELIX           19..30
FT                   /evidence="ECO:0007829|PDB:6N8Y"
FT   TURN            34..37
FT                   /evidence="ECO:0007829|PDB:5UCH"
FT   HELIX           38..60
FT                   /evidence="ECO:0007829|PDB:6N8Y"
FT   HELIX           62..65
FT                   /evidence="ECO:0007829|PDB:6N8Y"
FT   STRAND          73..78
FT                   /evidence="ECO:0007829|PDB:6N8Y"
FT   TURN            79..82
FT                   /evidence="ECO:0007829|PDB:6N8Y"
FT   STRAND          83..88
FT                   /evidence="ECO:0007829|PDB:6N8Y"
FT   HELIX           95..99
FT                   /evidence="ECO:0007829|PDB:6N8Y"
FT   HELIX           101..118
FT                   /evidence="ECO:0007829|PDB:6N8Y"
FT   HELIX           123..129
FT                   /evidence="ECO:0007829|PDB:6N8Y"
FT   HELIX           132..138
FT                   /evidence="ECO:0007829|PDB:6N8Y"
FT   STRAND          140..148
FT                   /evidence="ECO:0007829|PDB:6N8Y"
FT   STRAND          155..159
FT                   /evidence="ECO:0007829|PDB:6N8Y"
FT   STRAND          164..169
FT                   /evidence="ECO:0007829|PDB:6N8Y"
FT   STRAND          176..185
FT                   /evidence="ECO:0007829|PDB:6N8Y"
FT   HELIX           187..193
FT                   /evidence="ECO:0007829|PDB:6N8Y"
FT   HELIX           195..205
FT                   /evidence="ECO:0007829|PDB:6N8Y"
FT   STRAND          213..215
FT                   /evidence="ECO:0007829|PDB:6N8Y"
FT   HELIX           288..290
FT                   /evidence="ECO:0007829|PDB:3PRY"
FT   HELIX           293..295
FT                   /evidence="ECO:0007829|PDB:3PRY"
FT   HELIX           298..309
FT                   /evidence="ECO:0007829|PDB:3PRY"
FT   STRAND          316..323
FT                   /evidence="ECO:0007829|PDB:3PRY"
FT   STRAND          325..327
FT                   /evidence="ECO:0007829|PDB:3PRY"
FT   STRAND          329..335
FT                   /evidence="ECO:0007829|PDB:3PRY"
FT   STRAND          353..357
FT                   /evidence="ECO:0007829|PDB:3PRY"
FT   STRAND          360..364
FT                   /evidence="ECO:0007829|PDB:3PRY"
FT   HELIX           367..369
FT                   /evidence="ECO:0007829|PDB:3PRY"
FT   HELIX           372..374
FT                   /evidence="ECO:0007829|PDB:3PRY"
FT   STRAND          378..386
FT                   /evidence="ECO:0007829|PDB:3PRY"
FT   HELIX           395..420
FT                   /evidence="ECO:0007829|PDB:3PRY"
FT   HELIX           423..443
FT                   /evidence="ECO:0007829|PDB:3PRY"
FT   HELIX           445..447
FT                   /evidence="ECO:0007829|PDB:3PRY"
FT   HELIX           448..453
FT                   /evidence="ECO:0007829|PDB:3PRY"
FT   STRAND          456..459
FT                   /evidence="ECO:0007829|PDB:3PRY"
FT   TURN            460..464
FT                   /evidence="ECO:0007829|PDB:3PRY"
FT   HELIX           469..474
FT                   /evidence="ECO:0007829|PDB:3PRY"
FT   STRAND          482..486
FT                   /evidence="ECO:0007829|PDB:3PRY"
FT   HELIX           491..495
FT                   /evidence="ECO:0007829|PDB:3PRY"
FT   HELIX           498..504
FT                   /evidence="ECO:0007829|PDB:3PRY"
FT   TURN            505..507
FT                   /evidence="ECO:0007829|PDB:3PRY"
FT   STRAND          510..512
FT                   /evidence="ECO:0007829|PDB:3PRY"
FT   HELIX           518..525
FT                   /evidence="ECO:0007829|PDB:3PRY"
FT   STRAND          531..535
FT                   /evidence="ECO:0007829|PDB:3PRY"
SQ   SEQUENCE   724 AA;  83264 MW;  A93118C214D03810 CRC64;
     MPEEVHHGEE EVETFAFQAE IAQLMSLIIN TFYSNKEIFL RELISNASDA LDKIRYESLT
     DPSKLDSGKE LKIDIIPNPQ ERTLTLVDTG IGMTKADLIN NLGTIAKSGT KAFMEALQAG
     ADISMIGQFG VGFYSAYLVA EKVVVITKHN DDEQYAWESS AGGSFTVRAD HGEPIGRGTK
     VILHLKEDQT EYLEERRVKE VVKKHSQFIG YPITLYLEKE REKEISDDEA EEEKGEKEEE
     DKDDEEKPKI EDVGSDEEDD SGKDKKKKTK KIKEKYIDQE ELNKTKPIWT RNPDDITQEE
     YGEFYKSLTN DWEDHLAVKH FSVEGQLEFR ALLFIPRRAP FDLFENKKKK NNIKLYVRRV
     FIMDSCDELI PEYLNFIRGV VDSEDLPLNI SREMLQQSKI LKVIRKNIVK KCLELFSELA
     EDKENYKKFY EAFSKNLKLG IHEDSTNRRR LSELLRYHTS QSGDEMTSLS EYVSRMKETQ
     KSIYYITGES KEQVANSAFV ERVRKRGFEV VYMTEPIDEY CVQQLKEFDG KSLVSVTKEG
     LELPEDEEEK KKMEESKAKF ENLCKLMKEI LDKKVEKVTI SNRLVSSPCC IVTSTYGWTA
     NMERIMKAQA LRDNSTMGYM MAKKHLEINP DHPIVETLRQ KAEADKNDKA VKDLVVLLFE
     TALLSSGFSL EDPQTHSNRI YRMIKLGLGI DEDEVAAEEP NAAVPDEIPP LEGDEDASRM
     EEVD
```

|  |
| --- |
| **Mascot:** http://www.matrixscience.com/ |
