# Supplementary material for: Chlorpromazine induces cytotoxic autophagy in glioblastoma cells via endoplasmic reticulum stress and unfolded protein response
Source: J Exp Clin Cancer Res. 2021 Nov 5;40:347. doi: 10.1186/s13046-021-02144-w (PMC8569984; doi:10.1186/s13046-021-02144-w)
Supplement: Supplementary file 1 — Additional file 1. [file 13046_2021_2144_MOESM1_ESM.zip › MASCOT IDs/Mascot Search Results_ TCPA_HUMAN.html]

Mascot Search Results: TCPA\_HUMAN
 

# MASCOT Search Results

## Protein View: TCPA\_HUMAN

### T-complex protein 1 subunit alpha OS=Homo sapiens OX=9606 GN=TCP1 PE=1 SV=1

|  |  |
| --- | --- |
| Database: | SwissProt |
| Score: | 80 |
| Monoisotopic mass (Mr): | 60819 |
| Calculated pI: | 5.80 |
| Taxonomy: | Homo sapiens |

Sequence similarity is available as an NCBI BLAST search of TCPA\_HUMAN against nr.

### Search parameters

|  |  |
| --- | --- |
| MS data file: | `ppw_D8_152941904600.txt` |
| Enzyme: | Trypsin: cuts C-term side of KR unless next residue is P. |
| Fixed modifications: | Carbamidomethyl (C) |

### Protein sequence coverage: 2%

Matched peptides shown in ***bold red***.

|  |  |  |  |  |  |
| --- | --- | --- | --- | --- | --- |
| `1` | `MEGPLSVFGD` | `RSTGETIRSQ` | `NVMAAASIAN` | `IVKSSLGPVG` | `LDKMLVDDIG` |
| `51` | `DVTITNDGAT` | `ILKLLEVEHP` | `AAKVLCELAD` | `LQDKEVGDGT` | `TSVVIIAAEL` |
| `101` | `LKNADELVKQ` | `KIHPTSVISG` | `YRLACKEAVR` | `YINENLIVNT` | `DELGRDCLIN` |
| `151` | `AAKTSMSSKI` | `IGINGDFFAN` | `MVVDAVLAIK` | `YTDIRGQPRY` | `PVNSVNILKA` |
| `201` | `HGRSQMESML` | `ISGYALNCVV` | `GSQGMPKRIV` | `NAKIACLDFS` | `LQKTKMKLGV` |
| `251` | `QVVITDPEKL` | `DQIRQRESDI` | `TKERIQKILA` | `TGANVILTTG` | `GIDDMCLKYF` |
| `301` | `VEAGAMAVRR` | `VLKRDLKRIA` | `KASGATILST` | `LANLEGEETF` | `EAAMLGQAEE` |
| `351` | `VVQERICDDE` | `LILIKNTKAR` | `TSASIILRGA` | `NDFMCDEMER` | `SLHDALCVVK` |
| `401` | `RVLESKSVVP` | `GGGAVEAALS` | `IYLENYATSM` | `GSREQLAIAE` | `FARSLLVIPN` |
| `451` | `TLAVNAAQDS` | `TDLVAKLRAF` | `HNEAQVNPER` | `KNLKWIGLDL` | `SNGKPRDNKQ` |
| `501` | `AGVFEPTIVK` | `VKSLKFATEA` | `AITILRIDDL` | `IKLHPESKDD` | `KHGSYEDAVH` |
| `551` | `SGALND` |  |  |  |  |

Unformatted sequence string: 556 residues (for pasting into other applications).

|  |  |  |  |
| --- | --- | --- | --- |
| Sort by | residue number | increasing mass | decreasing mass |
| Show | matched peptides only | predicted peptides also |  |

| Query | Start | – | End | Observed | Mr(expt) | Mr(calc) | ppm | M | Score | Expect | Rank | U | Peptide |
| --- | --- | --- | --- | --- | --- | --- | --- | --- | --- | --- | --- | --- | --- |
| 49 | 469 | – | 481 | 1539.7798 | 1538.7725 | 1538.7590 | 8.82 | 1 | 80 | 3.5e-07 | 1Score **> 28** indicates **identity** | U | R.AFHNEAQVNPERK.N |

---

```
ID   TCPA_HUMAN              Reviewed;         556 AA.
AC   P17987; E1P5B2; Q15556; Q5TCM3;
DT   01-NOV-1990, integrated into UniProtKB/Swiss-Prot.
DT   01-NOV-1990, sequence version 1.
DT   02-JUN-2021, entry version 217.
DE   RecName: Full=T-complex protein 1 subunit alpha;
DE            Short=TCP-1-alpha;
DE   AltName: Full=CCT-alpha;
GN   Name=TCP1; Synonyms=CCT1, CCTA;
OS   Homo sapiens (Human).
OC   Eukaryota; Metazoa; Chordata; Craniata; Vertebrata; Euteleostomi; Mammalia;
OC   Eutheria; Euarchontoglires; Primates; Haplorrhini; Catarrhini; Hominidae;
OC   Homo.
OX   NCBI_TaxID=9606;
RN   [1]
RP   NUCLEOTIDE SEQUENCE [MRNA].
RC   TISSUE=Testis;
RX   PubMed=2377466; DOI=10.1093/nar/18.14.4247;
RA   Kirchhoff C., Willison K.R.;
RT   "Nucleotide and amino-acid sequence of human testis-derived TCP1.";
RL   Nucleic Acids Res. 18:4247-4247(1990).
RN   [2]
RP   NUCLEOTIDE SEQUENCE [LARGE SCALE MRNA].
RA   Kalnine N., Chen X., Rolfs A., Halleck A., Hines L., Eisenstein S.,
RA   Koundinya M., Raphael J., Moreira D., Kelley T., LaBaer J., Lin Y.,
RA   Phelan M., Farmer A.;
RT   "Cloning of human full-length CDSs in BD Creator(TM) system donor vector.";
RL   Submitted (OCT-2004) to the EMBL/GenBank/DDBJ databases.
RN   [3]
RP   NUCLEOTIDE SEQUENCE [LARGE SCALE GENOMIC DNA].
RX   PubMed=14574404; DOI=10.1038/nature02055;
RA   Mungall A.J., Palmer S.A., Sims S.K., Edwards C.A., Ashurst J.L.,
RA   Wilming L., Jones M.C., Horton R., Hunt S.E., Scott C.E., Gilbert J.G.R.,
RA   Clamp M.E., Bethel G., Milne S., Ainscough R., Almeida J.P., Ambrose K.D.,
RA   Andrews T.D., Ashwell R.I.S., Babbage A.K., Bagguley C.L., Bailey J.,
RA   Banerjee R., Barker D.J., Barlow K.F., Bates K., Beare D.M., Beasley H.,
RA   Beasley O., Bird C.P., Blakey S.E., Bray-Allen S., Brook J., Brown A.J.,
RA   Brown J.Y., Burford D.C., Burrill W., Burton J., Carder C., Carter N.P.,
RA   Chapman J.C., Clark S.Y., Clark G., Clee C.M., Clegg S., Cobley V.,
RA   Collier R.E., Collins J.E., Colman L.K., Corby N.R., Coville G.J.,
RA   Culley K.M., Dhami P., Davies J., Dunn M., Earthrowl M.E., Ellington A.E.,
RA   Evans K.A., Faulkner L., Francis M.D., Frankish A., Frankland J.,
RA   French L., Garner P., Garnett J., Ghori M.J., Gilby L.M., Gillson C.J.,
RA   Glithero R.J., Grafham D.V., Grant M., Gribble S., Griffiths C.,
RA   Griffiths M.N.D., Hall R., Halls K.S., Hammond S., Harley J.L., Hart E.A.,
RA   Heath P.D., Heathcott R., Holmes S.J., Howden P.J., Howe K.L., Howell G.R.,
RA   Huckle E., Humphray S.J., Humphries M.D., Hunt A.R., Johnson C.M.,
RA   Joy A.A., Kay M., Keenan S.J., Kimberley A.M., King A., Laird G.K.,
RA   Langford C., Lawlor S., Leongamornlert D.A., Leversha M., Lloyd C.R.,
RA   Lloyd D.M., Loveland J.E., Lovell J., Martin S., Mashreghi-Mohammadi M.,
RA   Maslen G.L., Matthews L., McCann O.T., McLaren S.J., McLay K., McMurray A.,
RA   Moore M.J.F., Mullikin J.C., Niblett D., Nickerson T., Novik K.L.,
RA   Oliver K., Overton-Larty E.K., Parker A., Patel R., Pearce A.V., Peck A.I.,
RA   Phillimore B.J.C.T., Phillips S., Plumb R.W., Porter K.M., Ramsey Y.,
RA   Ranby S.A., Rice C.M., Ross M.T., Searle S.M., Sehra H.K., Sheridan E.,
RA   Skuce C.D., Smith S., Smith M., Spraggon L., Squares S.L., Steward C.A.,
RA   Sycamore N., Tamlyn-Hall G., Tester J., Theaker A.J., Thomas D.W.,
RA   Thorpe A., Tracey A., Tromans A., Tubby B., Wall M., Wallis J.M.,
RA   West A.P., White S.S., Whitehead S.L., Whittaker H., Wild A., Willey D.J.,
RA   Wilmer T.E., Wood J.M., Wray P.W., Wyatt J.C., Young L., Younger R.M.,
RA   Bentley D.R., Coulson A., Durbin R.M., Hubbard T., Sulston J.E., Dunham I.,
RA   Rogers J., Beck S.;
RT   "The DNA sequence and analysis of human chromosome 6.";
RL   Nature 425:805-811(2003).
RN   [4]
RP   NUCLEOTIDE SEQUENCE [LARGE SCALE GENOMIC DNA].
RA   Mural R.J., Istrail S., Sutton G.G., Florea L., Halpern A.L., Mobarry C.M.,
RA   Lippert R., Walenz B., Shatkay H., Dew I., Miller J.R., Flanigan M.J.,
RA   Edwards N.J., Bolanos R., Fasulo D., Halldorsson B.V., Hannenhalli S.,
RA   Turner R., Yooseph S., Lu F., Nusskern D.R., Shue B.C., Zheng X.H.,
RA   Zhong F., Delcher A.L., Huson D.H., Kravitz S.A., Mouchard L., Reinert K.,
RA   Remington K.A., Clark A.G., Waterman M.S., Eichler E.E., Adams M.D.,
RA   Hunkapiller M.W., Myers E.W., Venter J.C.;
RL   Submitted (SEP-2005) to the EMBL/GenBank/DDBJ databases.
RN   [5]
RP   NUCLEOTIDE SEQUENCE [LARGE SCALE MRNA].
RC   TISSUE=Colon;
RX   PubMed=15489334; DOI=10.1101/gr.2596504;
RG   The MGC Project Team;
RT   "The status, quality, and expansion of the NIH full-length cDNA project:
RT   the Mammalian Gene Collection (MGC).";
RL   Genome Res. 14:2121-2127(2004).
RN   [6]
RP   PROTEIN SEQUENCE OF 1-11, AND ACETYLATION AT MET-1.
RC   TISSUE=Platelet;
RX   PubMed=12665801; DOI=10.1038/nbt810;
RA   Gevaert K., Goethals M., Martens L., Van Damme J., Staes A., Thomas G.R.,
RA   Vandekerckhove J.;
RT   "Exploring proteomes and analyzing protein processing by mass spectrometric
RT   identification of sorted N-terminal peptides.";
RL   Nat. Biotechnol. 21:566-569(2003).
RN   [7]
RP   PROTEIN SEQUENCE OF 112-122; 131-145; 248-264; 469-480 AND 516-526, AND
RP   IDENTIFICATION BY MASS SPECTROMETRY.
RC   TISSUE=Brain, and Cajal-Retzius cell;
RA   Lubec G., Vishwanath V.;
RL   Submitted (MAR-2007) to UniProtKB.
RN   [8]
RP   NUCLEOTIDE SEQUENCE [GENOMIC DNA] OF 308-365 AND 462-516.
RX   PubMed=3653076; DOI=10.1002/j.1460-2075.1987.tb02459.x;
RA   Willison K., Kelly A., Dudley K., Goodfellow P., Spurr N., Groves V.,
RA   Gorman P., Sheer D., Trowsdale J.;
RT   "The human homologue of the mouse T-complex gene, TCP1, is located on
RT   chromosome 6 but is not near the HLA region.";
RL   EMBO J. 6:1967-1974(1987).
RN   [9]
RP   SUBCELLULAR LOCATION, AND SUBUNIT.
RX   PubMed=1630492; DOI=10.1038/358249a0;
RA   Lewis V.A., Hynes G.M., Zheng D., Saibil H., Willison K.R.;
RT   "T-complex polypeptide-1 is a subunit of a heteromeric particle in the
RT   eukaryotic cytosol.";
RL   Nature 358:249-252(1992).
RN   [10]
RP   INTERACTION WITH PACRG.
RX   PubMed=14532270; DOI=10.1074/jbc.m309655200;
RA   Imai Y., Soda M., Murakami T., Shoji M., Abe K., Takahashi R.;
RT   "A product of the human gene adjacent to parkin is a component of Lewy
RT   bodies and suppresses Pael receptor-induced cell death.";
RL   J. Biol. Chem. 278:51901-51910(2003).
RN   [11]
RP   IDENTIFICATION BY MASS SPECTROMETRY, AND SUBCELLULAR LOCATION [LARGE SCALE
RP   ANALYSIS].
RC   TISSUE=Lymphoblast;
RX   PubMed=14654843; DOI=10.1038/nature02166;
RA   Andersen J.S., Wilkinson C.J., Mayor T., Mortensen P., Nigg E.A., Mann M.;
RT   "Proteomic characterization of the human centrosome by protein correlation
RT   profiling.";
RL   Nature 426:570-574(2003).
RN   [12]
RP   IDENTIFICATION BY MASS SPECTROMETRY [LARGE SCALE ANALYSIS].
RC   TISSUE=Cervix carcinoma;
RX   PubMed=17081983; DOI=10.1016/j.cell.2006.09.026;
RA   Olsen J.V., Blagoev B., Gnad F., Macek B., Kumar C., Mortensen P., Mann M.;
RT   "Global, in vivo, and site-specific phosphorylation dynamics in signaling
RT   networks.";
RL   Cell 127:635-648(2006).
RN   [13]
RP   IDENTIFICATION BY MASS SPECTROMETRY [LARGE SCALE ANALYSIS].
RC   TISSUE=Platelet;
RX   PubMed=18088087; DOI=10.1021/pr0704130;
RA   Zahedi R.P., Lewandrowski U., Wiesner J., Wortelkamp S., Moebius J.,
RA   Schuetz C., Walter U., Gambaryan S., Sickmann A.;
RT   "Phosphoproteome of resting human platelets.";
RL   J. Proteome Res. 7:526-534(2008).
RN   [14]
RP   PHOSPHORYLATION [LARGE SCALE ANALYSIS] AT SER-544, AND IDENTIFICATION BY
RP   MASS SPECTROMETRY [LARGE SCALE ANALYSIS].
RC   TISSUE=Cervix carcinoma;
RX   PubMed=18669648; DOI=10.1073/pnas.0805139105;
RA   Dephoure N., Zhou C., Villen J., Beausoleil S.A., Bakalarski C.E.,
RA   Elledge S.J., Gygi S.P.;
RT   "A quantitative atlas of mitotic phosphorylation.";
RL   Proc. Natl. Acad. Sci. U.S.A. 105:10762-10767(2008).
RN   [15]
RP   ACETYLATION [LARGE SCALE ANALYSIS] AT MET-1, AND IDENTIFICATION BY MASS
RP   SPECTROMETRY [LARGE SCALE ANALYSIS].
RX   PubMed=19413330; DOI=10.1021/ac9004309;
RA   Gauci S., Helbig A.O., Slijper M., Krijgsveld J., Heck A.J., Mohammed S.;
RT   "Lys-N and trypsin cover complementary parts of the phosphoproteome in a
RT   refined SCX-based approach.";
RL   Anal. Chem. 81:4493-4501(2009).
RN   [16]
RP   PHOSPHORYLATION [LARGE SCALE ANALYSIS] AT TYR-181 AND SER-544, AND
RP   IDENTIFICATION BY MASS SPECTROMETRY [LARGE SCALE ANALYSIS].
RC   TISSUE=Leukemic T-cell;
RX   PubMed=19690332; DOI=10.1126/scisignal.2000007;
RA   Mayya V., Lundgren D.H., Hwang S.-I., Rezaul K., Wu L., Eng J.K.,
RA   Rodionov V., Han D.K.;
RT   "Quantitative phosphoproteomic analysis of T cell receptor signaling
RT   reveals system-wide modulation of protein-protein interactions.";
RL   Sci. Signal. 2:RA46-RA46(2009).
RN   [17]
RP   ACETYLATION [LARGE SCALE ANALYSIS] AT LYS-199 AND LYS-400, AND
RP   IDENTIFICATION BY MASS SPECTROMETRY [LARGE SCALE ANALYSIS].
RX   PubMed=19608861; DOI=10.1126/science.1175371;
RA   Choudhary C., Kumar C., Gnad F., Nielsen M.L., Rehman M., Walther T.C.,
RA   Olsen J.V., Mann M.;
RT   "Lysine acetylation targets protein complexes and co-regulates major
RT   cellular functions.";
RL   Science 325:834-840(2009).
RN   [18]
RP   FUNCTION, SUBCELLULAR LOCATION, AND IDENTIFICATION IN THE
RP   CHAPERONIN-CONTAINING T-COMPLEX.
RX   PubMed=20080638; DOI=10.1073/pnas.0910268107;
RA   Seo S., Baye L.M., Schulz N.P., Beck J.S., Zhang Q., Slusarski D.C.,
RA   Sheffield V.C.;
RT   "BBS6, BBS10, and BBS12 form a complex with CCT/TRiC family chaperonins and
RT   mediate BBSome assembly.";
RL   Proc. Natl. Acad. Sci. U.S.A. 107:1488-1493(2010).
RN   [19]
RP   INTERACTION WITH GBA.
RX   PubMed=21098288; DOI=10.1073/pnas.1014376107;
RA   Lu J., Chiang J., Iyer R.R., Thompson E., Kaneski C.R., Xu D.S., Yang C.,
RA   Chen M., Hodes R.J., Lonser R.R., Brady R.O., Zhuang Z.;
RT   "Decreased glucocerebrosidase activity in Gaucher disease parallels
RT   quantitative enzyme loss due to abnormal interaction with TCP1 and c-Cbl.";
RL   Proc. Natl. Acad. Sci. U.S.A. 107:21665-21670(2010).
RN   [20]
RP   PHOSPHORYLATION [LARGE SCALE ANALYSIS] AT SER-544 AND SER-551, AND
RP   IDENTIFICATION BY MASS SPECTROMETRY [LARGE SCALE ANALYSIS].
RC   TISSUE=Cervix carcinoma;
RX   PubMed=20068231; DOI=10.1126/scisignal.2000475;
RA   Olsen J.V., Vermeulen M., Santamaria A., Kumar C., Miller M.L.,
RA   Jensen L.J., Gnad F., Cox J., Jensen T.S., Nigg E.A., Brunak S., Mann M.;
RT   "Quantitative phosphoproteomics reveals widespread full phosphorylation
RT   site occupancy during mitosis.";
RL   Sci. Signal. 3:RA3-RA3(2010).
RN   [21]
RP   IDENTIFICATION BY MASS SPECTROMETRY [LARGE SCALE ANALYSIS].
RX   PubMed=21269460; DOI=10.1186/1752-0509-5-17;
RA   Burkard T.R., Planyavsky M., Kaupe I., Breitwieser F.P., Buerckstuemmer T.,
RA   Bennett K.L., Superti-Furga G., Colinge J.;
RT   "Initial characterization of the human central proteome.";
RL   BMC Syst. Biol. 5:17-17(2011).
RN   [22]
RP   PHOSPHORYLATION [LARGE SCALE ANALYSIS] AT SER-544 AND SER-551, AND
RP   IDENTIFICATION BY MASS SPECTROMETRY [LARGE SCALE ANALYSIS].
RX   PubMed=21406692; DOI=10.1126/scisignal.2001570;
RA   Rigbolt K.T., Prokhorova T.A., Akimov V., Henningsen J., Johansen P.T.,
RA   Kratchmarova I., Kassem M., Mann M., Olsen J.V., Blagoev B.;
RT   "System-wide temporal characterization of the proteome and phosphoproteome
RT   of human embryonic stem cell differentiation.";
RL   Sci. Signal. 4:RS3-RS3(2011).
RN   [23]
RP   ACETYLATION [LARGE SCALE ANALYSIS] AT MET-1, AND IDENTIFICATION BY MASS
RP   SPECTROMETRY [LARGE SCALE ANALYSIS].
RX   PubMed=22223895; DOI=10.1074/mcp.m111.015131;
RA   Bienvenut W.V., Sumpton D., Martinez A., Lilla S., Espagne C., Meinnel T.,
RA   Giglione C.;
RT   "Comparative large-scale characterisation of plant vs. mammal proteins
RT   reveals similar and idiosyncratic N-alpha acetylation features.";
RL   Mol. Cell. Proteomics 11:M111.015131-M111.015131(2012).
RN   [24]
RP   ACETYLATION [LARGE SCALE ANALYSIS] AT MET-1, AND IDENTIFICATION BY MASS
RP   SPECTROMETRY [LARGE SCALE ANALYSIS].
RX   PubMed=22814378; DOI=10.1073/pnas.1210303109;
RA   Van Damme P., Lasa M., Polevoda B., Gazquez C., Elosegui-Artola A.,
RA   Kim D.S., De Juan-Pardo E., Demeyer K., Hole K., Larrea E., Timmerman E.,
RA   Prieto J., Arnesen T., Sherman F., Gevaert K., Aldabe R.;
RT   "N-terminal acetylome analyses and functional insights of the N-terminal
RT   acetyltransferase NatB.";
RL   Proc. Natl. Acad. Sci. U.S.A. 109:12449-12454(2012).
RN   [25]
RP   FUNCTION, AND IDENTIFICATION IN THE CHAPERONIN-CONTAINING T-COMPLEX.
RX   PubMed=25467444; DOI=10.1016/j.cell.2014.10.059;
RA   Freund A., Zhong F.L., Venteicher A.S., Meng Z., Veenstra T.D., Frydman J.,
RA   Artandi S.E.;
RT   "Proteostatic control of telomerase function through TRiC-mediated folding
RT   of TCAB1.";
RL   Cell 159:1389-1403(2014).
RN   [26]
RP   PHOSPHORYLATION [LARGE SCALE ANALYSIS] AT SER-6; SER-491; SER-544 AND
RP   SER-551, AND IDENTIFICATION BY MASS SPECTROMETRY [LARGE SCALE ANALYSIS].
RC   TISSUE=Cervix carcinoma, and Erythroleukemia;
RX   PubMed=23186163; DOI=10.1021/pr300630k;
RA   Zhou H., Di Palma S., Preisinger C., Peng M., Polat A.N., Heck A.J.,
RA   Mohammed S.;
RT   "Toward a comprehensive characterization of a human cancer cell
RT   phosphoproteome.";
RL   J. Proteome Res. 12:260-271(2013).
RN   [27]
RP   PHOSPHORYLATION [LARGE SCALE ANALYSIS] AT SER-544, AND IDENTIFICATION BY
RP   MASS SPECTROMETRY [LARGE SCALE ANALYSIS].
RC   TISSUE=Liver;
RX   PubMed=24275569; DOI=10.1016/j.jprot.2013.11.014;
RA   Bian Y., Song C., Cheng K., Dong M., Wang F., Huang J., Sun D., Wang L.,
RA   Ye M., Zou H.;
RT   "An enzyme assisted RP-RPLC approach for in-depth analysis of human liver
RT   phosphoproteome.";
RL   J. Proteomics 96:253-262(2014).
RN   [28]
RP   IDENTIFICATION BY MASS SPECTROMETRY [LARGE SCALE ANALYSIS].
RX   PubMed=25944712; DOI=10.1002/pmic.201400617;
RA   Vaca Jacome A.S., Rabilloud T., Schaeffer-Reiss C., Rompais M., Ayoub D.,
RA   Lane L., Bairoch A., Van Dorsselaer A., Carapito C.;
RT   "N-terminome analysis of the human mitochondrial proteome.";
RL   Proteomics 15:2519-2524(2015).
RN   [29]
RP   INTERACTION WITH DLEC1.
RX   PubMed=33144677; DOI=10.1038/s41598-020-75957-y;
RA   Okitsu Y., Nagano M., Yamagata T., Ito C., Toshimori K., Dohra H.,
RA   Fujii W., Yogo K.;
RT   "Dlec1 is required for spermatogenesis and male fertility in mice.";
RL   Sci. Rep. 10:18883-18883(2020).
RN   [30]
RP   VARIANT [LARGE SCALE ANALYSIS] LEU-7.
RX   PubMed=16959974; DOI=10.1126/science.1133427;
RA   Sjoeblom T., Jones S., Wood L.D., Parsons D.W., Lin J., Barber T.D.,
RA   Mandelker D., Leary R.J., Ptak J., Silliman N., Szabo S., Buckhaults P.,
RA   Farrell C., Meeh P., Markowitz S.D., Willis J., Dawson D., Willson J.K.V.,
RA   Gazdar A.F., Hartigan J., Wu L., Liu C., Parmigiani G., Park B.H.,
RA   Bachman K.E., Papadopoulos N., Vogelstein B., Kinzler K.W.,
RA   Velculescu V.E.;
RT   "The consensus coding sequences of human breast and colorectal cancers.";
RL   Science 314:268-274(2006).
CC   -!- FUNCTION: Component of the chaperonin-containing T-complex (TRiC), a
CC       molecular chaperone complex that assists the folding of proteins upon
CC       ATP hydrolysis (PubMed:25467444). The TRiC complex mediates the folding
CC       of WRAP53/TCAB1, thereby regulating telomere maintenance
CC       (PubMed:25467444). As part of the TRiC complex may play a role in the
CC       assembly of BBSome, a complex involved in ciliogenesis regulating
CC       transports vesicles to the cilia (PubMed:20080638). The TRiC complex
CC       plays a role in the folding of actin and tubulin (Probable).
CC       {ECO:0000269|PubMed:20080638, ECO:0000269|PubMed:25467444,
CC       ECO:0000305}.
CC   -!- SUBUNIT: Component of the chaperonin-containing T-complex (TRiC), a
CC       heterooligomeric complex of about 850 to 900 kDa that forms two stacked
CC       rings, 12 to 16 nm in diameter (PubMed:1630492, PubMed:20080638,
CC       PubMed:25467444). Interacts with PACRG (PubMed:14532270). Interacts
CC       with GBA (PubMed:21098288). Interacts with DLEC1 (PubMed:33144677).
CC       {ECO:0000269|PubMed:14532270, ECO:0000269|PubMed:1630492,
CC       ECO:0000269|PubMed:20080638, ECO:0000269|PubMed:21098288,
CC       ECO:0000269|PubMed:25467444, ECO:0000269|PubMed:33144677}.
CC   -!- INTERACTION:
CC       P17987; Q9Y2Y0: ARL2BP; NbExp=3; IntAct=EBI-356553, EBI-3449344;
CC       P17987; Q9ULD4-2: BRPF3; NbExp=3; IntAct=EBI-356553, EBI-23662416;
CC       P17987; O43439: CBFA2T2; NbExp=3; IntAct=EBI-356553, EBI-748628;
CC       P17987; P78371: CCT2; NbExp=3; IntAct=EBI-356553, EBI-357407;
CC       P17987; P50991: CCT4; NbExp=2; IntAct=EBI-356553, EBI-356876;
CC       P17987; P50990: CCT8; NbExp=2; IntAct=EBI-356553, EBI-356507;
CC       P17987; P04062: GBA; NbExp=2; IntAct=EBI-356553, EBI-1564609;
CC       P17987; P83110: HTRA3; NbExp=5; IntAct=EBI-356553, EBI-2867394;
CC       P17987; P83110-1: HTRA3; NbExp=6; IntAct=EBI-356553, EBI-25469082;
CC       P17987; P83110-2: HTRA3; NbExp=7; IntAct=EBI-356553, EBI-22017714;
CC       P17987; P83105: HTRA4; NbExp=7; IntAct=EBI-356553, EBI-21776319;
CC       P17987; P42858: HTT; NbExp=3; IntAct=EBI-356553, EBI-466029;
CC       P17987; P78380: OLR1; NbExp=5; IntAct=EBI-356553, EBI-7151999;
CC   -!- SUBCELLULAR LOCATION: Cytoplasm, cytosol {ECO:0000269|PubMed:1630492}.
CC       Cytoplasm, cytoskeleton, microtubule organizing center, centrosome
CC       {ECO:0000269|PubMed:14654843, ECO:0000269|PubMed:20080638}.
CC   -!- SIMILARITY: Belongs to the TCP-1 chaperonin family. {ECO:0000305}.
CC   ---------------------------------------------------------------------------
CC   Copyrighted by the UniProt Consortium, see https://www.uniprot.org/terms
CC   Distributed under the Creative Commons Attribution (CC BY 4.0) License
CC   ---------------------------------------------------------------------------
DR   EMBL; X52882; CAA37064.1; -; mRNA.
DR   EMBL; BT006969; AAP35615.1; -; mRNA.
DR   EMBL; AL135914; -; NOT_ANNOTATED_CDS; Genomic_DNA.
DR   EMBL; CH471051; EAW47616.1; -; Genomic_DNA.
DR   EMBL; CH471051; EAW47618.1; -; Genomic_DNA.
DR   EMBL; BC000665; AAH00665.1; -; mRNA.
DR   EMBL; M26885; AAA61059.1; -; Genomic_DNA.
DR   EMBL; M27272; AAA61060.1; -; Genomic_DNA.
DR   EMBL; M26889; AAA61060.1; JOINED; Genomic_DNA.
DR   CCDS; CCDS5269.1; -.
DR   PIR; S10486; S10486.
DR   RefSeq; NP_110379.2; NM_030752.2.
DR   PDB; 6NR8; EM; 7.80 A; A/I=1-534.
DR   PDB; 6NR9; EM; 8.50 A; A/I=1-534.
DR   PDB; 6NRA; EM; 7.70 A; A/I=1-534.
DR   PDB; 6NRB; EM; 8.70 A; A/I=1-534.
DR   PDB; 6NRC; EM; 8.30 A; A/I=1-534.
DR   PDB; 6NRD; EM; 8.20 A; A/I=1-534.
DR   PDB; 6QB8; EM; 3.97 A; A/a=1-556.
DR   PDB; 7LUM; EM; 4.50 A; G/O=1-556.
DR   PDB; 7LUP; EM; 6.20 A; G/O=1-556.
DR   PDBsum; 6NR8; -.
DR   PDBsum; 6NR9; -.
DR   PDBsum; 6NRA; -.
DR   PDBsum; 6NRB; -.
DR   PDBsum; 6NRC; -.
DR   PDBsum; 6NRD; -.
DR   PDBsum; 6QB8; -.
DR   PDBsum; 7LUM; -.
DR   PDBsum; 7LUP; -.
DR   SMR; P17987; -.
DR   BioGRID; 112810; 370.
DR   ComplexPortal; CPX-6030; Chaperonin-containing T-complex.
DR   CORUM; P17987; -.
DR   DIP; DIP-33676N; -.
DR   IntAct; P17987; 220.
DR   MINT; P17987; -.
DR   STRING; 9606.ENSP00000317334; -.
DR   iPTMnet; P17987; -.
DR   MetOSite; P17987; -.
DR   PhosphoSitePlus; P17987; -.
DR   SwissPalm; P17987; -.
DR   BioMuta; TCP1; -.
DR   DMDM; 135538; -.
DR   OGP; P17987; -.
DR   REPRODUCTION-2DPAGE; IPI00290566; -.
DR   REPRODUCTION-2DPAGE; P17987; -.
DR   UCD-2DPAGE; P17987; -.
DR   EPD; P17987; -.
DR   jPOST; P17987; -.
DR   MassIVE; P17987; -.
DR   MaxQB; P17987; -.
DR   PaxDb; P17987; -.
DR   PeptideAtlas; P17987; -.
DR   PRIDE; P17987; -.
DR   ProteomicsDB; 53538; -.
DR   Antibodypedia; 20023; 684 antibodies.
DR   DNASU; 6950; -.
DR   Ensembl; ENST00000321394; ENSP00000317334; ENSG00000120438.
DR   GeneID; 6950; -.
DR   KEGG; hsa:6950; -.
DR   UCSC; uc003qsr.4; human.
DR   CTD; 6950; -.
DR   DisGeNET; 6950; -.
DR   GeneCards; TCP1; -.
DR   HGNC; HGNC:11655; TCP1.
DR   HPA; ENSG00000120438; Low tissue specificity.
DR   MIM; 186980; gene.
DR   neXtProt; NX_P17987; -.
DR   OpenTargets; ENSG00000120438; -.
DR   PharmGKB; PA36406; -.
DR   VEuPathDB; HostDB:ENSG00000120438.11; -.
DR   eggNOG; KOG0360; Eukaryota.
DR   GeneTree; ENSGT00550000074878; -.
DR   InParanoid; P17987; -.
DR   OMA; KNYKNYG; -.
DR   OrthoDB; 335406at2759; -.
DR   PhylomeDB; P17987; -.
DR   TreeFam; TF106331; -.
DR   BRENDA; 3.6.4.B10; 2681.
DR   PathwayCommons; P17987; -.
DR   Reactome; R-HSA-389957; Prefoldin mediated transfer of substrate to CCT/TriC.
DR   Reactome; R-HSA-389960; Formation of tubulin folding intermediates by CCT/TriC.
DR   Reactome; R-HSA-390450; Folding of actin by CCT/TriC.
DR   Reactome; R-HSA-390471; Association of TriC/CCT with target proteins during biosynthesis.
DR   Reactome; R-HSA-5620922; BBSome-mediated cargo-targeting to cilium.
DR   Reactome; R-HSA-6814122; Cooperation of PDCL (PhLP1) and TRiC/CCT in G-protein beta folding.
DR   Reactome; R-HSA-8950505; Gene and protein expression by JAK-STAT signaling after Interleukin-12 stimulation.
DR   BioGRID-ORCS; 6950; 771 hits in 997 CRISPR screens.
DR   ChiTaRS; TCP1; human.
DR   GeneWiki; T-complex_1; -.
DR   GenomeRNAi; 6950; -.
DR   Pharos; P17987; Tbio.
DR   PRO; PR:P17987; -.
DR   Proteomes; UP000005640; Chromosome 6.
DR   RNAct; P17987; protein.
DR   Bgee; ENSG00000120438; Expressed in testis and 241 other tissues.
DR   ExpressionAtlas; P17987; baseline and differential.
DR   Genevisible; P17987; HS.
DR   GO; GO:0001669; C:acrosomal vesicle; IEA:Ensembl.
DR   GO; GO:0044297; C:cell body; IEA:Ensembl.
DR   GO; GO:0005813; C:centrosome; IDA:MGI.
DR   GO; GO:0005832; C:chaperonin-containing T-complex; IDA:UniProtKB.
DR   GO; GO:0005829; C:cytosol; TAS:Reactome.
DR   GO; GO:0070062; C:extracellular exosome; HDA:UniProtKB.
DR   GO; GO:0005794; C:Golgi apparatus; IEA:Ensembl.
DR   GO; GO:0000792; C:heterochromatin; IEA:Ensembl.
DR   GO; GO:0005874; C:microtubule; IDA:UniProtKB.
DR   GO; GO:0000242; C:pericentriolar material; IEA:Ensembl.
DR   GO; GO:0002199; C:zona pellucida receptor complex; IEA:Ensembl.
DR   GO; GO:0005524; F:ATP binding; IEA:UniProtKB-KW.
DR   GO; GO:0016887; F:ATPase activity; IEA:InterPro.
DR   GO; GO:0044183; F:protein folding chaperone; IDA:FlyBase.
DR   GO; GO:0003723; F:RNA binding; HDA:UniProtKB.
DR   GO; GO:0031625; F:ubiquitin protein ligase binding; IPI:ParkinsonsUK-UCL.
DR   GO; GO:0051082; F:unfolded protein binding; IBA:GO_Central.
DR   GO; GO:0007339; P:binding of sperm to zona pellucida; IEA:Ensembl.
DR   GO; GO:0035722; P:interleukin-12-mediated signaling pathway; TAS:Reactome.
DR   GO; GO:1904851; P:positive regulation of establishment of protein localization to telomere; IMP:BHF-UCL.
DR   GO; GO:1904871; P:positive regulation of protein localization to Cajal body; HMP:BHF-UCL.
DR   GO; GO:0051973; P:positive regulation of telomerase activity; IMP:BHF-UCL.
DR   GO; GO:1904874; P:positive regulation of telomerase RNA localization to Cajal body; IMP:BHF-UCL.
DR   GO; GO:0032212; P:positive regulation of telomere maintenance via telomerase; IMP:BHF-UCL.
DR   GO; GO:0006457; P:protein folding; IDA:FlyBase.
DR   GO; GO:0050821; P:protein stabilization; IMP:BHF-UCL.
DR   GO; GO:2000109; P:regulation of macrophage apoptotic process; IEA:Ensembl.
DR   GO; GO:0090666; P:scaRNA localization to Cajal body; IMP:BHF-UCL.
DR   GO; GO:1901998; P:toxin transport; IEA:Ensembl.
DR   GO; GO:0044053; P:translocation of peptides or proteins into host cell cytoplasm; IEA:Ensembl.
DR   GO; GO:0007021; P:tubulin complex assembly; NAS:UniProtKB.
DR   CDD; cd03335; TCP1_alpha; 1.
DR   Gene3D; 1.10.560.10; -; 1.
DR   Gene3D; 3.30.260.10; -; 1.
DR   Gene3D; 3.50.7.10; -; 1.
DR   InterPro; IPR012715; Chap_CCT_alpha.
DR   InterPro; IPR017998; Chaperone_TCP-1.
DR   InterPro; IPR002194; Chaperonin_TCP-1_CS.
DR   InterPro; IPR002423; Cpn60/TCP-1.
DR   InterPro; IPR027409; GroEL-like_apical_dom_sf.
DR   InterPro; IPR027413; GROEL-like_equatorial_sf.
DR   InterPro; IPR027410; TCP-1-like_intermed_sf.
DR   Pfam; PF00118; Cpn60_TCP1; 1.
DR   PRINTS; PR00304; TCOMPLEXTCP1.
DR   SUPFAM; SSF48592; SSF48592; 1.
DR   SUPFAM; SSF52029; SSF52029; 1.
DR   SUPFAM; SSF54849; SSF54849; 1.
DR   TIGRFAMs; TIGR02340; chap_CCT_alpha; 1.
DR   PROSITE; PS00750; TCP1_1; 1.
DR   PROSITE; PS00751; TCP1_2; 1.
DR   PROSITE; PS00995; TCP1_3; 1.
PE   1: Evidence at protein level;
KW   3D-structure; Acetylation; ATP-binding; Chaperone; Cytoplasm; Cytoskeleton;
KW   Direct protein sequencing; Nucleotide-binding; Phosphoprotein;
KW   Reference proteome.
FT   CHAIN           1..556
FT                   /note="T-complex protein 1 subunit alpha"
FT                   /id="PRO_0000128302"
FT   MOD_RES         1
FT                   /note="N-acetylmethionine"
FT                   /evidence="ECO:0000269|PubMed:12665801,
FT                   ECO:0007744|PubMed:19413330, ECO:0007744|PubMed:22223895,
FT                   ECO:0007744|PubMed:22814378"
FT   MOD_RES         6
FT                   /note="Phosphoserine"
FT                   /evidence="ECO:0007744|PubMed:23186163"
FT   MOD_RES         181
FT                   /note="Phosphotyrosine"
FT                   /evidence="ECO:0007744|PubMed:19690332"
FT   MOD_RES         199
FT                   /note="N6-acetyllysine"
FT                   /evidence="ECO:0007744|PubMed:19608861"
FT   MOD_RES         400
FT                   /note="N6-acetyllysine"
FT                   /evidence="ECO:0007744|PubMed:19608861"
FT   MOD_RES         491
FT                   /note="Phosphoserine"
FT                   /evidence="ECO:0007744|PubMed:23186163"
FT   MOD_RES         494
FT                   /note="N6-acetyllysine"
FT                   /evidence="ECO:0000250|UniProtKB:P11983"
FT   MOD_RES         544
FT                   /note="Phosphoserine"
FT                   /evidence="ECO:0007744|PubMed:18669648,
FT                   ECO:0007744|PubMed:19690332, ECO:0007744|PubMed:20068231,
FT                   ECO:0007744|PubMed:21406692, ECO:0007744|PubMed:23186163,
FT                   ECO:0007744|PubMed:24275569"
FT   MOD_RES         551
FT                   /note="Phosphoserine"
FT                   /evidence="ECO:0007744|PubMed:20068231,
FT                   ECO:0007744|PubMed:21406692, ECO:0007744|PubMed:23186163"
FT   VARIANT         7
FT                   /note="V -> L (in a breast cancer sample; somatic mutation;
FT                   dbSNP:rs537218073)"
FT                   /evidence="ECO:0000269|PubMed:16959974"
FT                   /id="VAR_036258"
FT   CONFLICT        480
FT                   /note="R -> S (in Ref. 8; AAA61060)"
FT                   /evidence="ECO:0000305"
FT   CONFLICT        537..540
FT                   /note="SKDD -> ILRI (in Ref. 1; CAA37064)"
FT                   /evidence="ECO:0000305"
SQ   SEQUENCE   556 AA;  60344 MW;  486ECA836EA258A1 CRC64;
     MEGPLSVFGD RSTGETIRSQ NVMAAASIAN IVKSSLGPVG LDKMLVDDIG DVTITNDGAT
     ILKLLEVEHP AAKVLCELAD LQDKEVGDGT TSVVIIAAEL LKNADELVKQ KIHPTSVISG
     YRLACKEAVR YINENLIVNT DELGRDCLIN AAKTSMSSKI IGINGDFFAN MVVDAVLAIK
     YTDIRGQPRY PVNSVNILKA HGRSQMESML ISGYALNCVV GSQGMPKRIV NAKIACLDFS
     LQKTKMKLGV QVVITDPEKL DQIRQRESDI TKERIQKILA TGANVILTTG GIDDMCLKYF
     VEAGAMAVRR VLKRDLKRIA KASGATILST LANLEGEETF EAAMLGQAEE VVQERICDDE
     LILIKNTKAR TSASIILRGA NDFMCDEMER SLHDALCVVK RVLESKSVVP GGGAVEAALS
     IYLENYATSM GSREQLAIAE FARSLLVIPN TLAVNAAQDS TDLVAKLRAF HNEAQVNPER
     KNLKWIGLDL SNGKPRDNKQ AGVFEPTIVK VKSLKFATEA AITILRIDDL IKLHPESKDD
     KHGSYEDAVH SGALND
```

|  |
| --- |
| **Mascot:** http://www.matrixscience.com/ |
