# Supplementary material for: Chlorpromazine induces cytotoxic autophagy in glioblastoma cells via endoplasmic reticulum stress and unfolded protein response
Source: J Exp Clin Cancer Res. 2021 Nov 5;40:347. doi: 10.1186/s13046-021-02144-w (PMC8569984; doi:10.1186/s13046-021-02144-w)
Supplement: Supplementary file 1 — Additional file 1. [file 13046_2021_2144_MOESM1_ESM.zip › MASCOT IDs/Mascot Search Results_ TCPE_HUMAN.html]

Mascot Search Results: TCPE\_HUMAN
 

# MASCOT Search Results

## Protein View: TCPE\_HUMAN

### T-complex protein 1 subunit epsilon OS=Homo sapiens OX=9606 GN=CCT5 PE=1 SV=1

|  |  |
| --- | --- |
| Database: | SwissProt |
| Score: | 84 |
| Monoisotopic mass (Mr): | 60089 |
| Calculated pI: | 5.45 |
| Taxonomy: | Homo sapiens |

Sequence similarity is available as an NCBI BLAST search of TCPE\_HUMAN against nr.

### Search parameters

|  |  |
| --- | --- |
| MS data file: | `ppw_B4_156283227800.txt` |
| Enzyme: | Trypsin: cuts C-term side of KR unless next residue is P. |
| Fixed modifications: | Carbamidomethyl (C) |

### Protein sequence coverage: 3%

Matched peptides shown in ***bold red***.

|  |  |  |  |  |  |
| --- | --- | --- | --- | --- | --- |
| `1` | `MASMGTLAFD` | `EYGRPFLIIK` | `DQDRKSRLMG` | `LEALKSHIMA` | `AKAVANTMRT` |
| `51` | `SLGPNGLDKM` | `MVDKDGDVTV` | `TNDGATILSM` | `MDVDHQIAKL` | `MVELSKSQDD` |
| `101` | `EIGDGTTGVV` | `VLAGALLEEA` | `EQLLDRGIHP` | `IRIADGYEQA` | `ARVAIEHLDK` |
| `151` | `ISDSVLVDIK` | `DTEPLIQTAK` | `TTLGSKVVNS` | `CHRQMAEIAV` | `NAVLTVADME` |
| `201` | `RRDVDFELIK` | `VEGKVGGRLE` | `DTKLIKGVIV` | `DKDFSHPQMP` | `KKVEDAKIAI` |
| `251` | `LTCPFEPPKP` | `KTKHKLDVTS` | `VEDYKALQKY` | `EKEKFEEMIQ` | `QIKETGANLA` |
| `301` | `ICQWGFDDEA` | `NHLLLQNNLP` | `AVRWVGGPEI` | `ELIAIATGGR` | `IVPRFSELTA` |
| `351` | `EKLGFAGLVQ` | `EISFGTTKDK` | `MLVIEQCKNS` | `RAVTIFIRGG` | `NKMIIEEAKR` |
| `401` | `SLHDALCVIR` | `NLIRDNRVVY` | `GGGAAEISCA` | `LAVSQEADKC` | `PTLEQYAMRA` |
| `451` | `FADALEVIPM` | `ALSENSGMNP` | `IQTMTEVRAR` | `QVKEMNPALG` | `IDCLHKGTND` |
| `501` | `MKQQHVIETL` | `IGKKQQISLA` | `TQMVRMILKI` | `DDIRKPGESE` | `E` |

Unformatted sequence string: 541 residues (for pasting into other applications).

|  |  |  |  |
| --- | --- | --- | --- |
| Sort by | residue number | increasing mass | decreasing mass |
| Show | matched peptides only | predicted peptides also |  |

| Query | Start | – | End | Observed | Mr(expt) | Mr(calc) | ppm | M | Score | Expect | Rank | U | Peptide |
| --- | --- | --- | --- | --- | --- | --- | --- | --- | --- | --- | --- | --- | --- |
| 53 | 324 | – | 340 | 1738.9879 | 1737.9806 | 1737.9414 | 22.6 | 0 | 84 | 1.3e-07 | 1Score **> 28** indicates **identity** | U | R.WVGGPEIELIAIATGGR.I |

---

```
ID   TCPE_HUMAN              Reviewed;         541 AA.
AC   P48643; A8JZY8; A8K2X8; B4DYD8;
DT   01-FEB-1996, integrated into UniProtKB/Swiss-Prot.
DT   01-FEB-1996, sequence version 1.
DT   02-JUN-2021, entry version 207.
DE   RecName: Full=T-complex protein 1 subunit epsilon;
DE            Short=TCP-1-epsilon;
DE   AltName: Full=CCT-epsilon;
GN   Name=CCT5; Synonyms=CCTE, KIAA0098 {ECO:0000303|PubMed:7788527};
OS   Homo sapiens (Human).
OC   Eukaryota; Metazoa; Chordata; Craniata; Vertebrata; Euteleostomi; Mammalia;
OC   Eutheria; Euarchontoglires; Primates; Haplorrhini; Catarrhini; Hominidae;
OC   Homo.
OX   NCBI_TaxID=9606;
RN   [1]
RP   NUCLEOTIDE SEQUENCE [LARGE SCALE MRNA] (ISOFORM 1).
RC   TISSUE=Bone marrow;
RX   PubMed=7788527; DOI=10.1093/dnares/2.1.37;
RA   Nagase T., Miyajima N., Tanaka A., Sazuka T., Seki N., Sato S., Tabata S.,
RA   Ishikawa K., Kawarabayasi Y., Kotani H., Nomura N.;
RT   "Prediction of the coding sequences of unidentified human genes. III. The
RT   coding sequences of 40 new genes (KIAA0081-KIAA0120) deduced by analysis of
RT   cDNA clones from human cell line KG-1.";
RL   DNA Res. 2:37-43(1995).
RN   [2]
RP   NUCLEOTIDE SEQUENCE [LARGE SCALE MRNA] (ISOFORMS 1 AND 2).
RC   TISSUE=Testis, and Umbilical cord blood;
RX   PubMed=14702039; DOI=10.1038/ng1285;
RA   Ota T., Suzuki Y., Nishikawa T., Otsuki T., Sugiyama T., Irie R.,
RA   Wakamatsu A., Hayashi K., Sato H., Nagai K., Kimura K., Makita H.,
RA   Sekine M., Obayashi M., Nishi T., Shibahara T., Tanaka T., Ishii S.,
RA   Yamamoto J., Saito K., Kawai Y., Isono Y., Nakamura Y., Nagahari K.,
RA   Murakami K., Yasuda T., Iwayanagi T., Wagatsuma M., Shiratori A., Sudo H.,
RA   Hosoiri T., Kaku Y., Kodaira H., Kondo H., Sugawara M., Takahashi M.,
RA   Kanda K., Yokoi T., Furuya T., Kikkawa E., Omura Y., Abe K., Kamihara K.,
RA   Katsuta N., Sato K., Tanikawa M., Yamazaki M., Ninomiya K., Ishibashi T.,
RA   Yamashita H., Murakawa K., Fujimori K., Tanai H., Kimata M., Watanabe M.,
RA   Hiraoka S., Chiba Y., Ishida S., Ono Y., Takiguchi S., Watanabe S.,
RA   Yosida M., Hotuta T., Kusano J., Kanehori K., Takahashi-Fujii A., Hara H.,
RA   Tanase T.-O., Nomura Y., Togiya S., Komai F., Hara R., Takeuchi K.,
RA   Arita M., Imose N., Musashino K., Yuuki H., Oshima A., Sasaki N.,
RA   Aotsuka S., Yoshikawa Y., Matsunawa H., Ichihara T., Shiohata N., Sano S.,
RA   Moriya S., Momiyama H., Satoh N., Takami S., Terashima Y., Suzuki O.,
RA   Nakagawa S., Senoh A., Mizoguchi H., Goto Y., Shimizu F., Wakebe H.,
RA   Hishigaki H., Watanabe T., Sugiyama A., Takemoto M., Kawakami B.,
RA   Yamazaki M., Watanabe K., Kumagai A., Itakura S., Fukuzumi Y., Fujimori Y.,
RA   Komiyama M., Tashiro H., Tanigami A., Fujiwara T., Ono T., Yamada K.,
RA   Fujii Y., Ozaki K., Hirao M., Ohmori Y., Kawabata A., Hikiji T.,
RA   Kobatake N., Inagaki H., Ikema Y., Okamoto S., Okitani R., Kawakami T.,
RA   Noguchi S., Itoh T., Shigeta K., Senba T., Matsumura K., Nakajima Y.,
RA   Mizuno T., Morinaga M., Sasaki M., Togashi T., Oyama M., Hata H.,
RA   Watanabe M., Komatsu T., Mizushima-Sugano J., Satoh T., Shirai Y.,
RA   Takahashi Y., Nakagawa K., Okumura K., Nagase T., Nomura N., Kikuchi H.,
RA   Masuho Y., Yamashita R., Nakai K., Yada T., Nakamura Y., Ohara O.,
RA   Isogai T., Sugano S.;
RT   "Complete sequencing and characterization of 21,243 full-length human
RT   cDNAs.";
RL   Nat. Genet. 36:40-45(2004).
RN   [3]
RP   NUCLEOTIDE SEQUENCE [LARGE SCALE GENOMIC DNA].
RX   PubMed=15372022; DOI=10.1038/nature02919;
RA   Schmutz J., Martin J., Terry A., Couronne O., Grimwood J., Lowry S.,
RA   Gordon L.A., Scott D., Xie G., Huang W., Hellsten U., Tran-Gyamfi M.,
RA   She X., Prabhakar S., Aerts A., Altherr M., Bajorek E., Black S.,
RA   Branscomb E., Caoile C., Challacombe J.F., Chan Y.M., Denys M.,
RA   Detter J.C., Escobar J., Flowers D., Fotopulos D., Glavina T., Gomez M.,
RA   Gonzales E., Goodstein D., Grigoriev I., Groza M., Hammon N., Hawkins T.,
RA   Haydu L., Israni S., Jett J., Kadner K., Kimball H., Kobayashi A.,
RA   Lopez F., Lou Y., Martinez D., Medina C., Morgan J., Nandkeshwar R.,
RA   Noonan J.P., Pitluck S., Pollard M., Predki P., Priest J., Ramirez L.,
RA   Retterer J., Rodriguez A., Rogers S., Salamov A., Salazar A., Thayer N.,
RA   Tice H., Tsai M., Ustaszewska A., Vo N., Wheeler J., Wu K., Yang J.,
RA   Dickson M., Cheng J.-F., Eichler E.E., Olsen A., Pennacchio L.A.,
RA   Rokhsar D.S., Richardson P., Lucas S.M., Myers R.M., Rubin E.M.;
RT   "The DNA sequence and comparative analysis of human chromosome 5.";
RL   Nature 431:268-274(2004).
RN   [4]
RP   NUCLEOTIDE SEQUENCE [LARGE SCALE GENOMIC DNA].
RA   Mural R.J., Istrail S., Sutton G.G., Florea L., Halpern A.L., Mobarry C.M.,
RA   Lippert R., Walenz B., Shatkay H., Dew I., Miller J.R., Flanigan M.J.,
RA   Edwards N.J., Bolanos R., Fasulo D., Halldorsson B.V., Hannenhalli S.,
RA   Turner R., Yooseph S., Lu F., Nusskern D.R., Shue B.C., Zheng X.H.,
RA   Zhong F., Delcher A.L., Huson D.H., Kravitz S.A., Mouchard L., Reinert K.,
RA   Remington K.A., Clark A.G., Waterman M.S., Eichler E.E., Adams M.D.,
RA   Hunkapiller M.W., Myers E.W., Venter J.C.;
RL   Submitted (SEP-2005) to the EMBL/GenBank/DDBJ databases.
RN   [5]
RP   NUCLEOTIDE SEQUENCE [LARGE SCALE MRNA] (ISOFORM 1).
RC   TISSUE=Lung, and Uterus;
RX   PubMed=15489334; DOI=10.1101/gr.2596504;
RG   The MGC Project Team;
RT   "The status, quality, and expansion of the NIH full-length cDNA project:
RT   the Mammalian Gene Collection (MGC).";
RL   Genome Res. 14:2121-2127(2004).
RN   [6]
RP   PROTEIN SEQUENCE OF 2-24; 28-35; 50-59; 90-96; 133-170; 184-201; 203-214;
RP   248-261; 264-275; 283-293; 324-340; 345-368; 382-388; 393-399; 401-410;
RP   484-496; 514-525 AND 530-541, CLEAVAGE OF INITIATOR METHIONINE, ACETYLATION
RP   AT ALA-2, AND IDENTIFICATION BY MASS SPECTROMETRY.
RC   TISSUE=Lung carcinoma;
RA   Bienvenut W.V., Vousden K.H., Lukashchuk N.;
RL   Submitted (MAR-2008) to UniProtKB.
RN   [7]
RP   PROTEIN SEQUENCE OF 2-20; 97-126; 133-170; 184-201; 203-214; 266-275;
RP   294-340; 345-368; 382-388 AND 401-410, CLEAVAGE OF INITIATOR METHIONINE,
RP   ACETYLATION AT ALA-2, AND IDENTIFICATION BY MASS SPECTROMETRY.
RC   TISSUE=Embryonic kidney;
RA   Bienvenut W.V., Waridel P., Quadroni M.;
RL   Submitted (MAR-2009) to UniProtKB.
RN   [8]
RP   PROTEIN SEQUENCE OF 203-210; 248-261; 324-340; 353-368 AND 515-525, AND
RP   IDENTIFICATION BY MASS SPECTROMETRY.
RC   TISSUE=Brain, Cajal-Retzius cell, and Fetal brain cortex;
RA   Lubec G., Afjehi-Sadat L., Chen W.-Q., Sun Y.;
RL   Submitted (DEC-2008) to UniProtKB.
RN   [9]
RP   INTERACTION WITH PACRG.
RX   PubMed=14532270; DOI=10.1074/jbc.m309655200;
RA   Imai Y., Soda M., Murakami T., Shoji M., Abe K., Takahashi R.;
RT   "A product of the human gene adjacent to parkin is a component of Lewy
RT   bodies and suppresses Pael receptor-induced cell death.";
RL   J. Biol. Chem. 278:51901-51910(2003).
RN   [10]
RP   IDENTIFICATION BY MASS SPECTROMETRY, AND SUBCELLULAR LOCATION [LARGE SCALE
RP   ANALYSIS].
RC   TISSUE=Lymphoblast;
RX   PubMed=14654843; DOI=10.1038/nature02166;
RA   Andersen J.S., Wilkinson C.J., Mayor T., Mortensen P., Nigg E.A., Mann M.;
RT   "Proteomic characterization of the human centrosome by protein correlation
RT   profiling.";
RL   Nature 426:570-574(2003).
RN   [11]
RP   INDUCTION, AND IDENTIFICATION BY MASS SPECTROMETRY.
RX   PubMed=16548883; DOI=10.1111/j.1462-5822.2005.00644.x;
RA   Leong W.F., Chow V.T.;
RT   "Transcriptomic and proteomic analyses of rhabdomyosarcoma cells reveal
RT   differential cellular gene expression in response to enterovirus 71
RT   infection.";
RL   Cell. Microbiol. 8:565-580(2006).
RN   [12]
RP   ACETYLATION [LARGE SCALE ANALYSIS] AT ALA-2, CLEAVAGE OF INITIATOR
RP   METHIONINE [LARGE SCALE ANALYSIS], AND IDENTIFICATION BY MASS SPECTROMETRY
RP   [LARGE SCALE ANALYSIS].
RX   PubMed=19413330; DOI=10.1021/ac9004309;
RA   Gauci S., Helbig A.O., Slijper M., Krijgsveld J., Heck A.J., Mohammed S.;
RT   "Lys-N and trypsin cover complementary parts of the phosphoproteome in a
RT   refined SCX-based approach.";
RL   Anal. Chem. 81:4493-4501(2009).
RN   [13]
RP   FUNCTION, SUBCELLULAR LOCATION, AND IDENTIFICATION IN CHAPERONIN-CONTAINING
RP   T-COMPLEX.
RX   PubMed=20080638; DOI=10.1073/pnas.0910268107;
RA   Seo S., Baye L.M., Schulz N.P., Beck J.S., Zhang Q., Slusarski D.C.,
RA   Sheffield V.C.;
RT   "BBS6, BBS10, and BBS12 form a complex with CCT/TRiC family chaperonins and
RT   mediate BBSome assembly.";
RL   Proc. Natl. Acad. Sci. U.S.A. 107:1488-1493(2010).
RN   [14]
RP   IDENTIFICATION BY MASS SPECTROMETRY [LARGE SCALE ANALYSIS].
RX   PubMed=21269460; DOI=10.1186/1752-0509-5-17;
RA   Burkard T.R., Planyavsky M., Kaupe I., Breitwieser F.P., Buerckstuemmer T.,
RA   Bennett K.L., Superti-Furga G., Colinge J.;
RT   "Initial characterization of the human central proteome.";
RL   BMC Syst. Biol. 5:17-17(2011).
RN   [15]
RP   PHOSPHORYLATION [LARGE SCALE ANALYSIS] AT SER-26, AND IDENTIFICATION BY
RP   MASS SPECTROMETRY [LARGE SCALE ANALYSIS].
RX   PubMed=21406692; DOI=10.1126/scisignal.2001570;
RA   Rigbolt K.T., Prokhorova T.A., Akimov V., Henningsen J., Johansen P.T.,
RA   Kratchmarova I., Kassem M., Mann M., Olsen J.V., Blagoev B.;
RT   "System-wide temporal characterization of the proteome and phosphoproteome
RT   of human embryonic stem cell differentiation.";
RL   Sci. Signal. 4:RS3-RS3(2011).
RN   [16]
RP   ACETYLATION [LARGE SCALE ANALYSIS] AT ALA-2, CLEAVAGE OF INITIATOR
RP   METHIONINE [LARGE SCALE ANALYSIS], AND IDENTIFICATION BY MASS SPECTROMETRY
RP   [LARGE SCALE ANALYSIS].
RX   PubMed=22223895; DOI=10.1074/mcp.m111.015131;
RA   Bienvenut W.V., Sumpton D., Martinez A., Lilla S., Espagne C., Meinnel T.,
RA   Giglione C.;
RT   "Comparative large-scale characterisation of plant vs. mammal proteins
RT   reveals similar and idiosyncratic N-alpha acetylation features.";
RL   Mol. Cell. Proteomics 11:M111.015131-M111.015131(2012).
RN   [17]
RP   ACETYLATION [LARGE SCALE ANALYSIS] AT ALA-2, CLEAVAGE OF INITIATOR
RP   METHIONINE [LARGE SCALE ANALYSIS], AND IDENTIFICATION BY MASS SPECTROMETRY
RP   [LARGE SCALE ANALYSIS].
RX   PubMed=22814378; DOI=10.1073/pnas.1210303109;
RA   Van Damme P., Lasa M., Polevoda B., Gazquez C., Elosegui-Artola A.,
RA   Kim D.S., De Juan-Pardo E., Demeyer K., Hole K., Larrea E., Timmerman E.,
RA   Prieto J., Arnesen T., Sherman F., Gevaert K., Aldabe R.;
RT   "N-terminal acetylome analyses and functional insights of the N-terminal
RT   acetyltransferase NatB.";
RL   Proc. Natl. Acad. Sci. U.S.A. 109:12449-12454(2012).
RN   [18]
RP   PHOSPHORYLATION [LARGE SCALE ANALYSIS] AT SER-346 AND SER-539, AND
RP   IDENTIFICATION BY MASS SPECTROMETRY [LARGE SCALE ANALYSIS].
RC   TISSUE=Erythroleukemia;
RX   PubMed=23186163; DOI=10.1021/pr300630k;
RA   Zhou H., Di Palma S., Preisinger C., Peng M., Polat A.N., Heck A.J.,
RA   Mohammed S.;
RT   "Toward a comprehensive characterization of a human cancer cell
RT   phosphoproteome.";
RL   J. Proteome Res. 12:260-271(2013).
RN   [19]
RP   FUNCTION, AND IDENTIFICATION IN THE CHAPERONIN-CONTAINING T-COMPLEX.
RX   PubMed=25467444; DOI=10.1016/j.cell.2014.10.059;
RA   Freund A., Zhong F.L., Venteicher A.S., Meng Z., Veenstra T.D., Frydman J.,
RA   Artandi S.E.;
RT   "Proteostatic control of telomerase function through TRiC-mediated folding
RT   of TCAB1.";
RL   Cell 159:1389-1403(2014).
RN   [20]
RP   IDENTIFICATION BY MASS SPECTROMETRY [LARGE SCALE ANALYSIS].
RC   TISSUE=Liver;
RX   PubMed=24275569; DOI=10.1016/j.jprot.2013.11.014;
RA   Bian Y., Song C., Cheng K., Dong M., Wang F., Huang J., Sun D., Wang L.,
RA   Ye M., Zou H.;
RT   "An enzyme assisted RP-RPLC approach for in-depth analysis of human liver
RT   phosphoproteome.";
RL   J. Proteomics 96:253-262(2014).
RN   [21]
RP   ACETYLATION [LARGE SCALE ANALYSIS] AT ALA-2, CLEAVAGE OF INITIATOR
RP   METHIONINE [LARGE SCALE ANALYSIS], AND IDENTIFICATION BY MASS SPECTROMETRY
RP   [LARGE SCALE ANALYSIS].
RX   PubMed=25944712; DOI=10.1002/pmic.201400617;
RA   Vaca Jacome A.S., Rabilloud T., Schaeffer-Reiss C., Rompais M., Ayoub D.,
RA   Lane L., Bairoch A., Van Dorsselaer A., Carapito C.;
RT   "N-terminome analysis of the human mitochondrial proteome.";
RL   Proteomics 15:2519-2524(2015).
RN   [22]
RP   SUMOYLATION [LARGE SCALE ANALYSIS] AT LYS-20; LYS-210; LYS-214; LYS-265;
RP   LYS-275; LYS-279 AND LYS-392, AND IDENTIFICATION BY MASS SPECTROMETRY
RP   [LARGE SCALE ANALYSIS].
RX   PubMed=28112733; DOI=10.1038/nsmb.3366;
RA   Hendriks I.A., Lyon D., Young C., Jensen L.J., Vertegaal A.C.,
RA   Nielsen M.L.;
RT   "Site-specific mapping of the human SUMO proteome reveals co-modification
RT   with phosphorylation.";
RL   Nat. Struct. Mol. Biol. 24:325-336(2017).
RN   [23]
RP   INTERACTION WITH DLEC1.
RX   PubMed=33144677; DOI=10.1038/s41598-020-75957-y;
RA   Okitsu Y., Nagano M., Yamagata T., Ito C., Toshimori K., Dohra H.,
RA   Fujii W., Yogo K.;
RT   "Dlec1 is required for spermatogenesis and male fertility in mice.";
RL   Sci. Rep. 10:18883-18883(2020).
RN   [24]
RP   VARIANT HSNSP ARG-147.
RX   PubMed=16399879; DOI=10.1136/jmg.2005.039230;
RA   Bouhouche A., Benomar A., Bouslam N., Chkili T., Yahyaoui M.;
RT   "Mutation in the epsilon subunit of the cytosolic chaperonin-containing T-
RT   complex peptide-1 (Cct5) gene causes autosomal recessive mutilating sensory
RT   neuropathy with spastic paraplegia.";
RL   J. Med. Genet. 43:441-443(2006).
CC   -!- FUNCTION: Component of the chaperonin-containing T-complex (TRiC), a
CC       molecular chaperone complex that assists the folding of proteins upon
CC       ATP hydrolysis (PubMed:25467444). The TRiC complex mediates the folding
CC       of WRAP53/TCAB1, thereby regulating telomere maintenance
CC       (PubMed:25467444). As part of the TRiC complex may play a role in the
CC       assembly of BBSome, a complex involved in ciliogenesis regulating
CC       transports vesicles to the cilia (PubMed:20080638). The TRiC complex
CC       plays a role in the folding of actin and tubulin (Probable).
CC       {ECO:0000269|PubMed:20080638, ECO:0000269|PubMed:25467444,
CC       ECO:0000305}.
CC   -!- SUBUNIT: Component of the chaperonin-containing T-complex (TRiC), a
CC       heterooligomeric complex of about 850 to 900 kDa that forms two stacked
CC       rings, 12 to 16 nm in diameter (PubMed:20080638, PubMed:25467444).
CC       Interacts with PACRG (PubMed:14532270). Interacts with DNAAF4 (By
CC       similarity). Interacts with DLEC1 (PubMed:33144677).
CC       {ECO:0000250|UniProtKB:P80316, ECO:0000269|PubMed:14532270,
CC       ECO:0000269|PubMed:20080638, ECO:0000269|PubMed:25467444,
CC       ECO:0000269|PubMed:33144677}.
CC   -!- INTERACTION:
CC       P48643; Q9NP61: ARFGAP3; NbExp=3; IntAct=EBI-355710, EBI-2875816;
CC       P48643; P54253: ATXN1; NbExp=3; IntAct=EBI-355710, EBI-930964;
CC       P48643; O95817: BAG3; NbExp=3; IntAct=EBI-355710, EBI-747185;
CC       P48643; Q14457: BECN1; NbExp=3; IntAct=EBI-355710, EBI-949378;
CC       P48643; Q32Q52: C12orf74; NbExp=3; IntAct=EBI-355710, EBI-12891828;
CC       P48643; Q9UNS2: COPS3; NbExp=3; IntAct=EBI-355710, EBI-350590;
CC       P48643; Q9H9Q2: COPS7B; NbExp=3; IntAct=EBI-355710, EBI-2510162;
CC       P48643; Q6NXG1: ESRP1; NbExp=3; IntAct=EBI-355710, EBI-10213520;
CC       P48643; Q9UKC9: FBXL2; NbExp=3; IntAct=EBI-355710, EBI-724253;
CC       P48643; Q9NXC2: GFOD1; NbExp=3; IntAct=EBI-355710, EBI-8799578;
CC       P48643; O75409: H2AP; NbExp=3; IntAct=EBI-355710, EBI-6447217;
CC       P48643; P52790: HK3; NbExp=3; IntAct=EBI-355710, EBI-2965780;
CC       P48643; P49639: HOXA1; NbExp=3; IntAct=EBI-355710, EBI-740785;
CC       P48643; P42858: HTT; NbExp=3; IntAct=EBI-355710, EBI-466029;
CC       P48643; Q14525: KRT33B; NbExp=3; IntAct=EBI-355710, EBI-1049638;
CC       P48643; Q68G74: LHX8; NbExp=3; IntAct=EBI-355710, EBI-8474075;
CC       P48643; A2RU56: LOC401296; NbExp=3; IntAct=EBI-355710, EBI-9088215;
CC       P48643; Q1L5Z9: LONRF2; NbExp=3; IntAct=EBI-355710, EBI-2510853;
CC       P48643; O15130-2: NPFF; NbExp=3; IntAct=EBI-355710, EBI-25840002;
CC       P48643; Q96LB9: PGLYRP3; NbExp=3; IntAct=EBI-355710, EBI-12339509;
CC       P48643; O75925: PIAS1; NbExp=3; IntAct=EBI-355710, EBI-629434;
CC       P48643; Q9H0F5-2: RNF38; NbExp=3; IntAct=EBI-355710, EBI-25866807;
CC       P48643; Q9NTN9-3: SEMA4G; NbExp=3; IntAct=EBI-355710, EBI-9089805;
CC       P48643; O60902-3: SHOX2; NbExp=3; IntAct=EBI-355710, EBI-9092164;
CC       P48643; Q99932-2: SPAG8; NbExp=3; IntAct=EBI-355710, EBI-11959123;
CC       P48643; O75886: STAM2; NbExp=3; IntAct=EBI-355710, EBI-373258;
CC       P48643; O95630: STAMBP; NbExp=3; IntAct=EBI-355710, EBI-396676;
CC       P48643; A1L378: STRC; NbExp=3; IntAct=EBI-355710, EBI-22013242;
CC       P48643; O60220: TIMM8A; NbExp=3; IntAct=EBI-355710, EBI-1049822;
CC       P48643; Q9UGJ1-2: TUBGCP4; NbExp=3; IntAct=EBI-355710, EBI-10964469;
CC       P48643; Q96B02: UBE2W; NbExp=3; IntAct=EBI-355710, EBI-716589;
CC       P48643; P45880: VDAC2; NbExp=3; IntAct=EBI-355710, EBI-354022;
CC       P48643; O00308: WWP2; NbExp=6; IntAct=EBI-355710, EBI-743923;
CC       P48643; Q96N77-2: ZNF641; NbExp=3; IntAct=EBI-355710, EBI-12939666;
CC       P48643; O60232: ZNRD2; NbExp=4; IntAct=EBI-355710, EBI-741415;
CC   -!- SUBCELLULAR LOCATION: Cytoplasm {ECO:0000269|PubMed:20080638}.
CC       Cytoplasm, cytoskeleton, microtubule organizing center, centrosome
CC       {ECO:0000269|PubMed:14654843, ECO:0000269|PubMed:20080638}.
CC   -!- ALTERNATIVE PRODUCTS:
CC       Event=Alternative splicing; Named isoforms=2;
CC       Name=1;
CC         IsoId=P48643-1; Sequence=Displayed;
CC       Name=2;
CC         IsoId=P48643-2; Sequence=VSP_054005, VSP_054006;
CC   -!- INDUCTION: Down-regulated in response to enterovirus 71 (EV71)
CC       infection (at protein level). {ECO:0000269|PubMed:16548883}.
CC   -!- DISEASE: Neuropathy, hereditary sensory, with spastic paraplegia,
CC       autosomal recessive (HSNSP) [MIM:256840]: A disease characterized by
CC       spastic paraplegia and progressive distal sensory neuropathy leading to
CC       mutilating ulcerations of the upper and lower limbs.
CC       {ECO:0000269|PubMed:16399879}. Note=The disease is caused by variants
CC       affecting the gene represented in this entry.
CC   -!- SIMILARITY: Belongs to the TCP-1 chaperonin family. {ECO:0000305}.
CC   -!- SEQUENCE CAUTION:
CC       Sequence=BAA07894.2; Type=Erroneous initiation; Evidence={ECO:0000305};
CC   ---------------------------------------------------------------------------
CC   Copyrighted by the UniProt Consortium, see https://www.uniprot.org/terms
CC   Distributed under the Creative Commons Attribution (CC BY 4.0) License
CC   ---------------------------------------------------------------------------
DR   EMBL; D43950; BAA07894.2; ALT_INIT; mRNA.
DR   EMBL; AK289353; BAF82042.1; -; mRNA.
DR   EMBL; AK290393; BAF83082.1; -; mRNA.
DR   EMBL; AK302383; BAG63700.1; -; mRNA.
DR   EMBL; AC012640; -; NOT_ANNOTATED_CDS; Genomic_DNA.
DR   EMBL; CH471102; EAX08072.1; -; Genomic_DNA.
DR   EMBL; BC006543; AAH06543.1; -; mRNA.
DR   EMBL; BC035499; AAH35499.1; -; mRNA.
DR   CCDS; CCDS3877.1; -. [P48643-1]
DR   CCDS; CCDS82990.1; -. [P48643-2]
DR   RefSeq; NP_001293084.1; NM_001306155.1. [P48643-2]
DR   RefSeq; NP_036205.1; NM_012073.4. [P48643-1]
DR   PDB; 5UYX; X-ray; 3.50 A; A/B/C/D=1-541.
DR   PDB; 5UYZ; X-ray; 3.60 A; A/B/C/D=1-541.
DR   PDB; 6NR8; EM; 7.80 A; E/M=25-541.
DR   PDB; 6NR9; EM; 8.50 A; E/M=25-541.
DR   PDB; 6NRA; EM; 7.70 A; E/M=25-541.
DR   PDB; 6NRB; EM; 8.70 A; E/M=25-541.
DR   PDB; 6NRC; EM; 8.30 A; E/M=25-541.
DR   PDB; 6NRD; EM; 8.20 A; E/M=25-541.
DR   PDB; 6QB8; EM; 3.97 A; E/e=1-541.
DR   PDB; 7LUM; EM; 4.50 A; D/L=1-541.
DR   PDB; 7LUP; EM; 6.20 A; D/L=1-541.
DR   PDBsum; 5UYX; -.
DR   PDBsum; 5UYZ; -.
DR   PDBsum; 6NR8; -.
DR   PDBsum; 6NR9; -.
DR   PDBsum; 6NRA; -.
DR   PDBsum; 6NRB; -.
DR   PDBsum; 6NRC; -.
DR   PDBsum; 6NRD; -.
DR   PDBsum; 6QB8; -.
DR   PDBsum; 7LUM; -.
DR   PDBsum; 7LUP; -.
DR   SMR; P48643; -.
DR   BioGRID; 116603; 347.
DR   ComplexPortal; CPX-6030; Chaperonin-containing T-complex.
DR   CORUM; P48643; -.
DR   DIP; DIP-31181N; -.
DR   IntAct; P48643; 221.
DR   MINT; P48643; -.
DR   STRING; 9606.ENSP00000280326; -.
DR   ChEMBL; CHEMBL4295766; -.
DR   iPTMnet; P48643; -.
DR   MetOSite; P48643; -.
DR   PhosphoSitePlus; P48643; -.
DR   SwissPalm; P48643; -.
DR   BioMuta; CCT5; -.
DR   DMDM; 1351211; -.
DR   OGP; P48643; -.
DR   REPRODUCTION-2DPAGE; IPI00010720; -.
DR   SWISS-2DPAGE; P48643; -.
DR   EPD; P48643; -.
DR   jPOST; P48643; -.
DR   MassIVE; P48643; -.
DR   PaxDb; P48643; -.
DR   PeptideAtlas; P48643; -.
DR   PRIDE; P48643; -.
DR   ProteomicsDB; 5518; -.
DR   ProteomicsDB; 55918; -. [P48643-1]
DR   Antibodypedia; 1210; 325 antibodies.
DR   DNASU; 22948; -.
DR   Ensembl; ENST00000280326; ENSP00000280326; ENSG00000150753. [P48643-1]
DR   Ensembl; ENST00000506600; ENSP00000423052; ENSG00000150753. [P48643-2]
DR   GeneID; 22948; -.
DR   KEGG; hsa:22948; -.
DR   UCSC; uc011cmt.3; human. [P48643-1]
DR   CTD; 22948; -.
DR   DisGeNET; 22948; -.
DR   GeneCards; CCT5; -.
DR   HGNC; HGNC:1618; CCT5.
DR   HPA; ENSG00000150753; Low tissue specificity.
DR   MalaCards; CCT5; -.
DR   MIM; 256840; phenotype.
DR   MIM; 610150; gene.
DR   neXtProt; NX_P48643; -.
DR   OpenTargets; ENSG00000150753; -.
DR   Orphanet; 139578; Mutilating hereditary sensory neuropathy with spastic paraplegia.
DR   PharmGKB; PA26182; -.
DR   VEuPathDB; HostDB:ENSG00000150753.11; -.
DR   eggNOG; KOG0357; Eukaryota.
DR   GeneTree; ENSGT00550000074988; -.
DR   InParanoid; P48643; -.
DR   OMA; AHDQFAN; -.
DR   PhylomeDB; P48643; -.
DR   TreeFam; TF105638; -.
DR   BRENDA; 3.6.4.B10; 2681.
DR   PathwayCommons; P48643; -.
DR   Reactome; R-HSA-389957; Prefoldin mediated transfer of substrate to CCT/TriC.
DR   Reactome; R-HSA-389960; Formation of tubulin folding intermediates by CCT/TriC.
DR   Reactome; R-HSA-390450; Folding of actin by CCT/TriC.
DR   Reactome; R-HSA-390471; Association of TriC/CCT with target proteins during biosynthesis.
DR   Reactome; R-HSA-5620922; BBSome-mediated cargo-targeting to cilium.
DR   Reactome; R-HSA-6814122; Cooperation of PDCL (PhLP1) and TRiC/CCT in G-protein beta folding.
DR   BioGRID-ORCS; 22948; 751 hits in 992 CRISPR screens.
DR   ChiTaRS; CCT5; human.
DR   GeneWiki; CCT5_(gene); -.
DR   GenomeRNAi; 22948; -.
DR   Pharos; P48643; Tbio.
DR   PRO; PR:P48643; -.
DR   Proteomes; UP000005640; Chromosome 5.
DR   RNAct; P48643; protein.
DR   Bgee; ENSG00000150753; Expressed in female gonad and 249 other tissues.
DR   ExpressionAtlas; P48643; baseline and differential.
DR   Genevisible; P48643; HS.
DR   GO; GO:0044297; C:cell body; IEA:Ensembl.
DR   GO; GO:0005813; C:centrosome; IDA:MGI.
DR   GO; GO:0005832; C:chaperonin-containing T-complex; IDA:UniProtKB.
DR   GO; GO:0005829; C:cytosol; TAS:Reactome.
DR   GO; GO:0070062; C:extracellular exosome; HDA:UniProtKB.
DR   GO; GO:0005874; C:microtubule; IDA:UniProtKB.
DR   GO; GO:0005524; F:ATP binding; IEA:UniProtKB-KW.
DR   GO; GO:0016887; F:ATPase activity; IEA:InterPro.
DR   GO; GO:0048487; F:beta-tubulin binding; IPI:UniProtKB.
DR   GO; GO:0031681; F:G-protein beta-subunit binding; IPI:UniProtKB.
DR   GO; GO:0003730; F:mRNA 3'-UTR binding; IDA:CAFA.
DR   GO; GO:0048027; F:mRNA 5'-UTR binding; IDA:CAFA.
DR   GO; GO:0044183; F:protein folding chaperone; IDA:FlyBase.
DR   GO; GO:0051082; F:unfolded protein binding; IBA:GO_Central.
DR   GO; GO:0007339; P:binding of sperm to zona pellucida; IEA:Ensembl.
DR   GO; GO:1904851; P:positive regulation of establishment of protein localization to telomere; IMP:BHF-UCL.
DR   GO; GO:1904871; P:positive regulation of protein localization to Cajal body; HMP:BHF-UCL.
DR   GO; GO:1904874; P:positive regulation of telomerase RNA localization to Cajal body; HMP:BHF-UCL.
DR   GO; GO:0032212; P:positive regulation of telomere maintenance via telomerase; IMP:BHF-UCL.
DR   GO; GO:0006457; P:protein folding; IDA:FlyBase.
DR   GO; GO:0050821; P:protein stabilization; IMP:BHF-UCL.
DR   GO; GO:0009615; P:response to virus; IEP:UniProtKB.
DR   GO; GO:1901998; P:toxin transport; IEA:Ensembl.
DR   CDD; cd03339; TCP1_epsilon; 1.
DR   Gene3D; 1.10.560.10; -; 1.
DR   Gene3D; 3.30.260.10; -; 1.
DR   Gene3D; 3.50.7.10; -; 1.
DR   InterPro; IPR012718; Chap_CCT_epsi.
DR   InterPro; IPR017998; Chaperone_TCP-1.
DR   InterPro; IPR002194; Chaperonin_TCP-1_CS.
DR   InterPro; IPR002423; Cpn60/TCP-1.
DR   InterPro; IPR027409; GroEL-like_apical_dom_sf.
DR   InterPro; IPR027413; GROEL-like_equatorial_sf.
DR   InterPro; IPR027410; TCP-1-like_intermed_sf.
DR   Pfam; PF00118; Cpn60_TCP1; 1.
DR   PRINTS; PR00304; TCOMPLEXTCP1.
DR   SUPFAM; SSF48592; SSF48592; 1.
DR   SUPFAM; SSF52029; SSF52029; 1.
DR   SUPFAM; SSF54849; SSF54849; 1.
DR   TIGRFAMs; TIGR02343; chap_CCT_epsi; 1.
DR   PROSITE; PS00750; TCP1_1; 1.
DR   PROSITE; PS00751; TCP1_2; 1.
DR   PROSITE; PS00995; TCP1_3; 1.
PE   1: Evidence at protein level;
KW   3D-structure; Acetylation; Alternative splicing; ATP-binding; Chaperone;
KW   Cytoplasm; Cytoskeleton; Direct protein sequencing; Disease variant;
KW   Isopeptide bond; Neuropathy; Nucleotide-binding; Phosphoprotein;
KW   Reference proteome; Ubl conjugation.
FT   INIT_MET        1
FT                   /note="Removed"
FT                   /evidence="ECO:0000269|Ref.6, ECO:0000269|Ref.7,
FT                   ECO:0007744|PubMed:19413330, ECO:0007744|PubMed:22223895,
FT                   ECO:0007744|PubMed:22814378, ECO:0007744|PubMed:25944712"
FT   CHAIN           2..541
FT                   /note="T-complex protein 1 subunit epsilon"
FT                   /id="PRO_0000128346"
FT   MOD_RES         2
FT                   /note="N-acetylalanine"
FT                   /evidence="ECO:0000269|Ref.6, ECO:0000269|Ref.7,
FT                   ECO:0007744|PubMed:19413330, ECO:0007744|PubMed:22223895,
FT                   ECO:0007744|PubMed:22814378, ECO:0007744|PubMed:25944712"
FT   MOD_RES         26
FT                   /note="Phosphoserine"
FT                   /evidence="ECO:0007744|PubMed:21406692"
FT   MOD_RES         346
FT                   /note="Phosphoserine"
FT                   /evidence="ECO:0007744|PubMed:23186163"
FT   MOD_RES         539
FT                   /note="Phosphoserine"
FT                   /evidence="ECO:0007744|PubMed:23186163"
FT   CROSSLNK        20
FT                   /note="Glycyl lysine isopeptide (Lys-Gly) (interchain with
FT                   G-Cter in SUMO2)"
FT                   /evidence="ECO:0007744|PubMed:28112733"
FT   CROSSLNK        210
FT                   /note="Glycyl lysine isopeptide (Lys-Gly) (interchain with
FT                   G-Cter in SUMO2)"
FT                   /evidence="ECO:0007744|PubMed:28112733"
FT   CROSSLNK        214
FT                   /note="Glycyl lysine isopeptide (Lys-Gly) (interchain with
FT                   G-Cter in SUMO2)"
FT                   /evidence="ECO:0007744|PubMed:28112733"
FT   CROSSLNK        265
FT                   /note="Glycyl lysine isopeptide (Lys-Gly) (interchain with
FT                   G-Cter in SUMO2)"
FT                   /evidence="ECO:0007744|PubMed:28112733"
FT   CROSSLNK        275
FT                   /note="Glycyl lysine isopeptide (Lys-Gly) (interchain with
FT                   G-Cter in SUMO2)"
FT                   /evidence="ECO:0007744|PubMed:28112733"
FT   CROSSLNK        279
FT                   /note="Glycyl lysine isopeptide (Lys-Gly) (interchain with
FT                   G-Cter in SUMO2)"
FT                   /evidence="ECO:0007744|PubMed:28112733"
FT   CROSSLNK        392
FT                   /note="Glycyl lysine isopeptide (Lys-Gly) (interchain with
FT                   G-Cter in SUMO2)"
FT                   /evidence="ECO:0007744|PubMed:28112733"
FT   VAR_SEQ         1..38
FT                   /note="Missing (in isoform 2)"
FT                   /evidence="ECO:0000303|PubMed:14702039"
FT                   /id="VSP_054005"
FT   VAR_SEQ         56..110
FT                   /note="Missing (in isoform 2)"
FT                   /evidence="ECO:0000303|PubMed:14702039"
FT                   /id="VSP_054006"
FT   VARIANT         146
FT                   /note="E -> V (in dbSNP:rs11557652)"
FT                   /id="VAR_052267"
FT   VARIANT         147
FT                   /note="H -> R (in HSNSP; dbSNP:rs118203986)"
FT                   /evidence="ECO:0000269|PubMed:16399879"
FT                   /id="VAR_030658"
FT   CONFLICT        55
FT                   /note="N -> D (in Ref. 2; BAF83082)"
FT                   /evidence="ECO:0000305"
FT   CONFLICT        512
FT                   /note="G -> D (in Ref. 2; BAF83082)"
FT                   /evidence="ECO:0000305"
FT   HELIX           32..49
FT                   /evidence="ECO:0007829|PDB:5UYX"
FT   HELIX           74..80
FT                   /evidence="ECO:0007829|PDB:5UYX"
FT   HELIX           86..102
FT                   /evidence="ECO:0007829|PDB:5UYX"
FT   HELIX           107..125
FT                   /evidence="ECO:0007829|PDB:5UYX"
FT   HELIX           130..151
FT                   /evidence="ECO:0007829|PDB:5UYX"
FT   STRAND          158..161
FT                   /evidence="ECO:0007829|PDB:5UYX"
FT   HELIX           164..169
FT                   /evidence="ECO:0007829|PDB:5UYX"
FT   HELIX           183..195
FT                   /evidence="ECO:0007829|PDB:5UYX"
FT   STRAND          200..202
FT                   /evidence="ECO:0007829|PDB:5UYX"
FT   STRAND          208..213
FT                   /evidence="ECO:0007829|PDB:5UYX"
FT   HELIX           219..221
FT                   /evidence="ECO:0007829|PDB:5UYX"
FT   STRAND          226..230
FT                   /evidence="ECO:0007829|PDB:5UYX"
FT   STRAND          241..246
FT                   /evidence="ECO:0007829|PDB:5UYX"
FT   STRAND          248..251
FT                   /evidence="ECO:0007829|PDB:5UYX"
FT   HELIX           271..293
FT                   /evidence="ECO:0007829|PDB:5UYX"
FT   STRAND          298..301
FT                   /evidence="ECO:0007829|PDB:5UYX"
FT   HELIX           308..315
FT                   /evidence="ECO:0007829|PDB:5UYX"
FT   TURN            316..318
FT                   /evidence="ECO:0007829|PDB:5UYX"
FT   TURN            328..330
FT                   /evidence="ECO:0007829|PDB:5UYX"
FT   HELIX           331..336
FT                   /evidence="ECO:0007829|PDB:5UYX"
FT   HELIX           350..352
FT                   /evidence="ECO:0007829|PDB:5UYX"
FT   STRAND          356..364
FT                   /evidence="ECO:0007829|PDB:5UYX"
FT   STRAND          367..374
FT                   /evidence="ECO:0007829|PDB:5UYX"
FT   STRAND          383..387
FT                   /evidence="ECO:0007829|PDB:5UYX"
FT   TURN            391..393
FT                   /evidence="ECO:0007829|PDB:5UYX"
FT   HELIX           394..414
FT                   /evidence="ECO:0007829|PDB:5UYX"
FT   TURN            421..423
FT                   /evidence="ECO:0007829|PDB:5UYX"
FT   HELIX           424..438
FT                   /evidence="ECO:0007829|PDB:5UYX"
FT   HELIX           444..455
FT                   /evidence="ECO:0007829|PDB:5UYX"
FT   HELIX           457..464
FT                   /evidence="ECO:0007829|PDB:5UYX"
FT   TURN            465..467
FT                   /evidence="ECO:0007829|PDB:5UYX"
FT   HELIX           470..484
FT                   /evidence="ECO:0007829|PDB:5UYX"
FT   HELIX           503..505
FT                   /evidence="ECO:0007829|PDB:5UYX"
FT   HELIX           510..528
FT                   /evidence="ECO:0007829|PDB:5UYX"
SQ   SEQUENCE   541 AA;  59671 MW;  164168BB80EF022A CRC64;
     MASMGTLAFD EYGRPFLIIK DQDRKSRLMG LEALKSHIMA AKAVANTMRT SLGPNGLDKM
     MVDKDGDVTV TNDGATILSM MDVDHQIAKL MVELSKSQDD EIGDGTTGVV VLAGALLEEA
     EQLLDRGIHP IRIADGYEQA ARVAIEHLDK ISDSVLVDIK DTEPLIQTAK TTLGSKVVNS
     CHRQMAEIAV NAVLTVADME RRDVDFELIK VEGKVGGRLE DTKLIKGVIV DKDFSHPQMP
     KKVEDAKIAI LTCPFEPPKP KTKHKLDVTS VEDYKALQKY EKEKFEEMIQ QIKETGANLA
     ICQWGFDDEA NHLLLQNNLP AVRWVGGPEI ELIAIATGGR IVPRFSELTA EKLGFAGLVQ
     EISFGTTKDK MLVIEQCKNS RAVTIFIRGG NKMIIEEAKR SLHDALCVIR NLIRDNRVVY
     GGGAAEISCA LAVSQEADKC PTLEQYAMRA FADALEVIPM ALSENSGMNP IQTMTEVRAR
     QVKEMNPALG IDCLHKGTND MKQQHVIETL IGKKQQISLA TQMVRMILKI DDIRKPGESE
     E
```

|  |
| --- |
| **Mascot:** http://www.matrixscience.com/ |
