# Supplementary material for: Chlorpromazine induces cytotoxic autophagy in glioblastoma cells via endoplasmic reticulum stress and unfolded protein response
Source: J Exp Clin Cancer Res. 2021 Nov 5;40:347. doi: 10.1186/s13046-021-02144-w (PMC8569984; doi:10.1186/s13046-021-02144-w)
Supplement: Supplementary file 1 — Additional file 1. [file 13046_2021_2144_MOESM1_ESM.zip › MASCOT IDs/Mascot Search Results_ TCPQ_HUMAN.html]

Mascot Search Results: TCPQ\_HUMAN
 

# MASCOT Search Results

## Protein View: TCPQ\_HUMAN

### T-complex protein 1 subunit theta OS=Homo sapiens OX=9606 GN=CCT8 PE=1 SV=4

|  |  |
| --- | --- |
| Database: | SwissProt |
| Score: | 64 |
| Monoisotopic mass (Mr): | 60153 |
| Calculated pI: | 5.42 |
| Taxonomy: | Homo sapiens |

Sequence similarity is available as an NCBI BLAST search of TCPQ\_HUMAN against nr.

### Search parameters

|  |  |
| --- | --- |
| MS data file: | `ppw_D21_152941902000.txt` |
| Enzyme: | Trypsin: cuts C-term side of KR unless next residue is P. |
| Fixed modifications: | Carbamidomethyl (C) |

### Protein sequence coverage: 1%

Matched peptides shown in ***bold red***.

|  |  |  |  |  |  |
| --- | --- | --- | --- | --- | --- |
| `1` | `MALHVPKAPG` | `FAQMLKEGAK` | `HFSGLEEAVY` | `RNIQACKELA` | `QTTRTAYGPN` |
| `51` | `GMNKMVINHL` | `EKLFVTNDAA` | `TILRELEVQH` | `PAAKMIVMAS` | `HMQEQEVGDG` |
| `101` | `TNFVLVFAGA` | `LLELAEELLR` | `IGLSVSEVIE` | `GYEIACRKAH` | `EILPNLVCCS` |
| `151` | `AKNLRDIDEV` | `SSLLRTSIMS` | `KQYGNEVFLA` | `KLIAQACVSI` | `FPDSGHFNVD` |
| `201` | `NIRVCKILGS` | `GISSSSVLHG` | `MVFKKETEGD` | `VTSVKDAKIA` | `VYSCPFDGMI` |
| `251` | `TETKGTVLIK` | `TAEELMNFSK` | `GEENLMDAQV` | `KAIADTGANV` | `VVTGGKVADM` |
| `301` | `ALHYANKYNI` | `MLVRLNSKWD` | `LRRLCKTVGA` | `TALPRLTPPV` | `LEEMGHCDSV` |
| `351` | `YLSEVGDTQV` | `VVFKHEKEDG` | `AISTIVLRGS` | `TDNLMDDIER` | `AVDDGVNTFK` |
| `401` | `VLTRDKRLVP` | `GGGATEIELA` | `KQITSYGETC` | `PGLEQYAIKK` | `FAEAFEAIPR` |
| `451` | `ALAENSGVKA` | `NEVISKLYAV` | `HQEGNKNVGL` | `DIEAEVPAVK` | `DMLEAGILDT` |
| `501` | `YLGKYWAIKL` | `ATNAAVTVLR` | `VDQIIMAKPA` | `GGPKPPSGKK` | `DWDDDQND` |

Unformatted sequence string: 548 residues (for pasting into other applications).

|  |  |  |  |
| --- | --- | --- | --- |
| Sort by | residue number | increasing mass | decreasing mass |
| Show | matched peptides only | predicted peptides also |  |

| Query | Start | – | End | Observed | Mr(expt) | Mr(calc) | ppm | M | Score | Expect | Rank | U | Peptide |
| --- | --- | --- | --- | --- | --- | --- | --- | --- | --- | --- | --- | --- | --- |
| 20 | 441 | – | 450 | 1150.6035 | 1149.5962 | 1149.5818 | 12.5 | 0 | 64 | 1.5e-05 | 1Score **> 28** indicates **identity** | U | K.FAEAFEAIPR.A |

---

```
ID   TCPQ_HUMAN              Reviewed;         548 AA.
AC   P50990; A6NN54; B4DEM7; B4DQH4; Q4VBP8;
DT   01-OCT-1996, integrated into UniProtKB/Swiss-Prot.
DT   23-JAN-2007, sequence version 4.
DT   02-JUN-2021, entry version 208.
DE   RecName: Full=T-complex protein 1 subunit theta;
DE            Short=TCP-1-theta;
DE   AltName: Full=CCT-theta;
DE   AltName: Full=Chaperonin containing T-complex polypeptide 1 subunit 8;
DE   AltName: Full=Renal carcinoma antigen NY-REN-15;
GN   Name=CCT8; Synonyms=C21orf112, CCTQ, KIAA0002;
OS   Homo sapiens (Human).
OC   Eukaryota; Metazoa; Chordata; Craniata; Vertebrata; Euteleostomi; Mammalia;
OC   Eutheria; Euarchontoglires; Primates; Haplorrhini; Catarrhini; Hominidae;
OC   Homo.
OX   NCBI_TaxID=9606;
RN   [1]
RP   NUCLEOTIDE SEQUENCE [LARGE SCALE MRNA] (ISOFORM 1).
RC   TISSUE=Bone marrow;
RX   PubMed=7584026; DOI=10.1093/dnares/1.1.27;
RA   Nomura N., Miyajima N., Sazuka T., Tanaka A., Kawarabayasi Y., Sato S.,
RA   Nagase T., Seki N., Ishikawa K., Tabata S.;
RT   "Prediction of the coding sequences of unidentified human genes. I. The
RT   coding sequences of 40 new genes (KIAA0001-KIAA0040) deduced by analysis of
RT   randomly sampled cDNA clones from human immature myeloid cell line KG-1.";
RL   DNA Res. 1:27-35(1994).
RN   [2]
RP   NUCLEOTIDE SEQUENCE [GENOMIC DNA].
RX   PubMed=8590283; DOI=10.1093/dnares/2.4.187;
RA   Yamazaki M., Ono A., Watanabe K., Sasaki K., Tashiro H., Nomura T.;
RT   "Nucleotide sequence surrounding the locus marker D21S246 on human
RT   chromosome 21.";
RL   DNA Res. 2:187-189(1995).
RN   [3]
RP   NUCLEOTIDE SEQUENCE [GENOMIC DNA].
RA   Taudien S., Dagand E., Delabar J., Nordsiek G., Drescher B., Weber J.,
RA   Schattevoy R., Menzel U., Yaspo M.-L., Rosenthal A.;
RL   Submitted (FEB-1999) to the EMBL/GenBank/DDBJ databases.
RN   [4]
RP   NUCLEOTIDE SEQUENCE [LARGE SCALE MRNA] (ISOFORMS 2 AND 3).
RC   TISSUE=Cerebellum;
RX   PubMed=14702039; DOI=10.1038/ng1285;
RA   Ota T., Suzuki Y., Nishikawa T., Otsuki T., Sugiyama T., Irie R.,
RA   Wakamatsu A., Hayashi K., Sato H., Nagai K., Kimura K., Makita H.,
RA   Sekine M., Obayashi M., Nishi T., Shibahara T., Tanaka T., Ishii S.,
RA   Yamamoto J., Saito K., Kawai Y., Isono Y., Nakamura Y., Nagahari K.,
RA   Murakami K., Yasuda T., Iwayanagi T., Wagatsuma M., Shiratori A., Sudo H.,
RA   Hosoiri T., Kaku Y., Kodaira H., Kondo H., Sugawara M., Takahashi M.,
RA   Kanda K., Yokoi T., Furuya T., Kikkawa E., Omura Y., Abe K., Kamihara K.,
RA   Katsuta N., Sato K., Tanikawa M., Yamazaki M., Ninomiya K., Ishibashi T.,
RA   Yamashita H., Murakawa K., Fujimori K., Tanai H., Kimata M., Watanabe M.,
RA   Hiraoka S., Chiba Y., Ishida S., Ono Y., Takiguchi S., Watanabe S.,
RA   Yosida M., Hotuta T., Kusano J., Kanehori K., Takahashi-Fujii A., Hara H.,
RA   Tanase T.-O., Nomura Y., Togiya S., Komai F., Hara R., Takeuchi K.,
RA   Arita M., Imose N., Musashino K., Yuuki H., Oshima A., Sasaki N.,
RA   Aotsuka S., Yoshikawa Y., Matsunawa H., Ichihara T., Shiohata N., Sano S.,
RA   Moriya S., Momiyama H., Satoh N., Takami S., Terashima Y., Suzuki O.,
RA   Nakagawa S., Senoh A., Mizoguchi H., Goto Y., Shimizu F., Wakebe H.,
RA   Hishigaki H., Watanabe T., Sugiyama A., Takemoto M., Kawakami B.,
RA   Yamazaki M., Watanabe K., Kumagai A., Itakura S., Fukuzumi Y., Fujimori Y.,
RA   Komiyama M., Tashiro H., Tanigami A., Fujiwara T., Ono T., Yamada K.,
RA   Fujii Y., Ozaki K., Hirao M., Ohmori Y., Kawabata A., Hikiji T.,
RA   Kobatake N., Inagaki H., Ikema Y., Okamoto S., Okitani R., Kawakami T.,
RA   Noguchi S., Itoh T., Shigeta K., Senba T., Matsumura K., Nakajima Y.,
RA   Mizuno T., Morinaga M., Sasaki M., Togashi T., Oyama M., Hata H.,
RA   Watanabe M., Komatsu T., Mizushima-Sugano J., Satoh T., Shirai Y.,
RA   Takahashi Y., Nakagawa K., Okumura K., Nagase T., Nomura N., Kikuchi H.,
RA   Masuho Y., Yamashita R., Nakai K., Yada T., Nakamura Y., Ohara O.,
RA   Isogai T., Sugano S.;
RT   "Complete sequencing and characterization of 21,243 full-length human
RT   cDNAs.";
RL   Nat. Genet. 36:40-45(2004).
RN   [5]
RP   NUCLEOTIDE SEQUENCE [LARGE SCALE GENOMIC DNA].
RX   PubMed=10830953; DOI=10.1038/35012518;
RA   Hattori M., Fujiyama A., Taylor T.D., Watanabe H., Yada T., Park H.-S.,
RA   Toyoda A., Ishii K., Totoki Y., Choi D.-K., Groner Y., Soeda E., Ohki M.,
RA   Takagi T., Sakaki Y., Taudien S., Blechschmidt K., Polley A., Menzel U.,
RA   Delabar J., Kumpf K., Lehmann R., Patterson D., Reichwald K., Rump A.,
RA   Schillhabel M., Schudy A., Zimmermann W., Rosenthal A., Kudoh J.,
RA   Shibuya K., Kawasaki K., Asakawa S., Shintani A., Sasaki T., Nagamine K.,
RA   Mitsuyama S., Antonarakis S.E., Minoshima S., Shimizu N., Nordsiek G.,
RA   Hornischer K., Brandt P., Scharfe M., Schoen O., Desario A., Reichelt J.,
RA   Kauer G., Bloecker H., Ramser J., Beck A., Klages S., Hennig S.,
RA   Riesselmann L., Dagand E., Wehrmeyer S., Borzym K., Gardiner K.,
RA   Nizetic D., Francis F., Lehrach H., Reinhardt R., Yaspo M.-L.;
RT   "The DNA sequence of human chromosome 21.";
RL   Nature 405:311-319(2000).
RN   [6]
RP   NUCLEOTIDE SEQUENCE [LARGE SCALE MRNA] (ISOFORM 1).
RC   TISSUE=Chondrosarcoma, and Liver;
RX   PubMed=15489334; DOI=10.1101/gr.2596504;
RG   The MGC Project Team;
RT   "The status, quality, and expansion of the NIH full-length cDNA project:
RT   the Mammalian Gene Collection (MGC).";
RL   Genome Res. 14:2121-2127(2004).
RN   [7]
RP   PROTEIN SEQUENCE OF 2-16; 55-74; 156-165; 172-181; 283-296; 408-421;
RP   441-450 AND 510-520, CLEAVAGE OF INITIATOR METHIONINE, ACETYLATION AT
RP   ALA-2, AND IDENTIFICATION BY MASS SPECTROMETRY.
RC   TISSUE=B-cell lymphoma;
RA   Bienvenut W.V.;
RL   Submitted (OCT-2004) to UniProtKB.
RN   [8]
RP   NUCLEOTIDE SEQUENCE [MRNA] OF 33-65 (ISOFORM 1/2).
RX   PubMed=7890169; DOI=10.1016/0378-1119(94)00880-2;
RA   Kubota H., Hynes G., Willison K.;
RT   "The eighth Cct gene, Cctq, encoding the theta subunit of the cytosolic
RT   chaperonin containing TCP-1.";
RL   Gene 154:231-236(1995).
RN   [9]
RP   IDENTIFICATION AS A RENAL CANCER ANTIGEN.
RC   TISSUE=Renal cell carcinoma;
RX   PubMed=10508479;
RX   DOI=10.1002/(sici)1097-0215(19991112)83:4<456::aid-ijc4>3.0.co;2-5;
RA   Scanlan M.J., Gordan J.D., Williamson B., Stockert E., Bander N.H.,
RA   Jongeneel C.V., Gure A.O., Jaeger D., Jaeger E., Knuth A., Chen Y.-T.,
RA   Old L.J.;
RT   "Antigens recognized by autologous antibody in patients with renal-cell
RT   carcinoma.";
RL   Int. J. Cancer 83:456-464(1999).
RN   [10]
RP   INTERACTION WITH PACRG.
RX   PubMed=14532270; DOI=10.1074/jbc.m309655200;
RA   Imai Y., Soda M., Murakami T., Shoji M., Abe K., Takahashi R.;
RT   "A product of the human gene adjacent to parkin is a component of Lewy
RT   bodies and suppresses Pael receptor-induced cell death.";
RL   J. Biol. Chem. 278:51901-51910(2003).
RN   [11]
RP   IDENTIFICATION BY MASS SPECTROMETRY, AND SUBCELLULAR LOCATION [LARGE SCALE
RP   ANALYSIS].
RC   TISSUE=Lymphoblast;
RX   PubMed=14654843; DOI=10.1038/nature02166;
RA   Andersen J.S., Wilkinson C.J., Mayor T., Mortensen P., Nigg E.A., Mann M.;
RT   "Proteomic characterization of the human centrosome by protein correlation
RT   profiling.";
RL   Nature 426:570-574(2003).
RN   [12]
RP   PHOSPHORYLATION [LARGE SCALE ANALYSIS] AT TYR-505, AND IDENTIFICATION BY
RP   MASS SPECTROMETRY [LARGE SCALE ANALYSIS].
RX   PubMed=15592455; DOI=10.1038/nbt1046;
RA   Rush J., Moritz A., Lee K.A., Guo A., Goss V.L., Spek E.J., Zhang H.,
RA   Zha X.-M., Polakiewicz R.D., Comb M.J.;
RT   "Immunoaffinity profiling of tyrosine phosphorylation in cancer cells.";
RL   Nat. Biotechnol. 23:94-101(2005).
RN   [13]
RP   PHOSPHORYLATION [LARGE SCALE ANALYSIS] AT SER-23, AND IDENTIFICATION BY
RP   MASS SPECTROMETRY [LARGE SCALE ANALYSIS].
RC   TISSUE=Cervix carcinoma;
RX   PubMed=18669648; DOI=10.1073/pnas.0805139105;
RA   Dephoure N., Zhou C., Villen J., Beausoleil S.A., Bakalarski C.E.,
RA   Elledge S.J., Gygi S.P.;
RT   "A quantitative atlas of mitotic phosphorylation.";
RL   Proc. Natl. Acad. Sci. U.S.A. 105:10762-10767(2008).
RN   [14]
RP   PHOSPHORYLATION [LARGE SCALE ANALYSIS] AT SER-23 AND TYR-30, AND
RP   IDENTIFICATION BY MASS SPECTROMETRY [LARGE SCALE ANALYSIS].
RC   TISSUE=Leukemic T-cell;
RX   PubMed=19690332; DOI=10.1126/scisignal.2000007;
RA   Mayya V., Lundgren D.H., Hwang S.-I., Rezaul K., Wu L., Eng J.K.,
RA   Rodionov V., Han D.K.;
RT   "Quantitative phosphoproteomic analysis of T cell receptor signaling
RT   reveals system-wide modulation of protein-protein interactions.";
RL   Sci. Signal. 2:RA46-RA46(2009).
RN   [15]
RP   ACETYLATION [LARGE SCALE ANALYSIS] AT LYS-318; LYS-400 AND LYS-466, AND
RP   IDENTIFICATION BY MASS SPECTROMETRY [LARGE SCALE ANALYSIS].
RX   PubMed=19608861; DOI=10.1126/science.1175371;
RA   Choudhary C., Kumar C., Gnad F., Nielsen M.L., Rehman M., Walther T.C.,
RA   Olsen J.V., Mann M.;
RT   "Lysine acetylation targets protein complexes and co-regulates major
RT   cellular functions.";
RL   Science 325:834-840(2009).
RN   [16]
RP   FUNCTION, SUBCELLULAR LOCATION, AND IDENTIFICATION IN THE
RP   CHAPERONIN-CONTAINING T-COMPLEX.
RX   PubMed=20080638; DOI=10.1073/pnas.0910268107;
RA   Seo S., Baye L.M., Schulz N.P., Beck J.S., Zhang Q., Slusarski D.C.,
RA   Sheffield V.C.;
RT   "BBS6, BBS10, and BBS12 form a complex with CCT/TRiC family chaperonins and
RT   mediate BBSome assembly.";
RL   Proc. Natl. Acad. Sci. U.S.A. 107:1488-1493(2010).
RN   [17]
RP   IDENTIFICATION BY MASS SPECTROMETRY [LARGE SCALE ANALYSIS].
RX   PubMed=21269460; DOI=10.1186/1752-0509-5-17;
RA   Burkard T.R., Planyavsky M., Kaupe I., Breitwieser F.P., Buerckstuemmer T.,
RA   Bennett K.L., Superti-Furga G., Colinge J.;
RT   "Initial characterization of the human central proteome.";
RL   BMC Syst. Biol. 5:17-17(2011).
RN   [18]
RP   PHOSPHORYLATION [LARGE SCALE ANALYSIS] AT SER-23; TYR-30; SER-162; SER-213;
RP   SER-269; SER-317 AND SER-537, AND IDENTIFICATION BY MASS SPECTROMETRY
RP   [LARGE SCALE ANALYSIS].
RC   TISSUE=Cervix carcinoma, and Erythroleukemia;
RX   PubMed=23186163; DOI=10.1021/pr300630k;
RA   Zhou H., Di Palma S., Preisinger C., Peng M., Polat A.N., Heck A.J.,
RA   Mohammed S.;
RT   "Toward a comprehensive characterization of a human cancer cell
RT   phosphoproteome.";
RL   J. Proteome Res. 12:260-271(2013).
RN   [19]
RP   FUNCTION, AND IDENTIFICATION IN THE CHAPERONIN-CONTAINING T-COMPLEX.
RX   PubMed=25467444; DOI=10.1016/j.cell.2014.10.059;
RA   Freund A., Zhong F.L., Venteicher A.S., Meng Z., Veenstra T.D., Frydman J.,
RA   Artandi S.E.;
RT   "Proteostatic control of telomerase function through TRiC-mediated folding
RT   of TCAB1.";
RL   Cell 159:1389-1403(2014).
RN   [20]
RP   IDENTIFICATION BY MASS SPECTROMETRY [LARGE SCALE ANALYSIS].
RC   TISSUE=Liver;
RX   PubMed=24275569; DOI=10.1016/j.jprot.2013.11.014;
RA   Bian Y., Song C., Cheng K., Dong M., Wang F., Huang J., Sun D., Wang L.,
RA   Ye M., Zou H.;
RT   "An enzyme assisted RP-RPLC approach for in-depth analysis of human liver
RT   phosphoproteome.";
RL   J. Proteomics 96:253-262(2014).
RN   [21]
RP   SUMOYLATION [LARGE SCALE ANALYSIS] AT LYS-534, AND IDENTIFICATION BY MASS
RP   SPECTROMETRY [LARGE SCALE ANALYSIS].
RX   PubMed=25218447; DOI=10.1038/nsmb.2890;
RA   Hendriks I.A., D'Souza R.C., Yang B., Verlaan-de Vries M., Mann M.,
RA   Vertegaal A.C.;
RT   "Uncovering global SUMOylation signaling networks in a site-specific
RT   manner.";
RL   Nat. Struct. Mol. Biol. 21:927-936(2014).
RN   [22]
RP   SUMOYLATION [LARGE SCALE ANALYSIS] AT LYS-459, AND IDENTIFICATION BY MASS
RP   SPECTROMETRY [LARGE SCALE ANALYSIS].
RX   PubMed=25114211; DOI=10.1073/pnas.1413825111;
RA   Impens F., Radoshevich L., Cossart P., Ribet D.;
RT   "Mapping of SUMO sites and analysis of SUMOylation changes induced by
RT   external stimuli.";
RL   Proc. Natl. Acad. Sci. U.S.A. 111:12432-12437(2014).
RN   [23]
RP   SUMOYLATION [LARGE SCALE ANALYSIS] AT LYS-534 AND LYS-539, AND
RP   IDENTIFICATION BY MASS SPECTROMETRY [LARGE SCALE ANALYSIS].
RX   PubMed=25755297; DOI=10.1074/mcp.o114.044792;
RA   Xiao Z., Chang J.G., Hendriks I.A., Sigurdsson J.O., Olsen J.V.,
RA   Vertegaal A.C.;
RT   "System-wide analysis of SUMOylation dynamics in response to replication
RT   stress reveals novel small ubiquitin-like modified target proteins and
RT   acceptor lysines relevant for genome stability.";
RL   Mol. Cell. Proteomics 14:1419-1434(2015).
RN   [24]
RP   IDENTIFICATION BY MASS SPECTROMETRY [LARGE SCALE ANALYSIS].
RX   PubMed=25944712; DOI=10.1002/pmic.201400617;
RA   Vaca Jacome A.S., Rabilloud T., Schaeffer-Reiss C., Rompais M., Ayoub D.,
RA   Lane L., Bairoch A., Van Dorsselaer A., Carapito C.;
RT   "N-terminome analysis of the human mitochondrial proteome.";
RL   Proteomics 15:2519-2524(2015).
RN   [25]
RP   SUMOYLATION [LARGE SCALE ANALYSIS] AT LYS-224; LYS-254; LYS-260; LYS-534
RP   AND LYS-539, AND IDENTIFICATION BY MASS SPECTROMETRY [LARGE SCALE
RP   ANALYSIS].
RX   PubMed=28112733; DOI=10.1038/nsmb.3366;
RA   Hendriks I.A., Lyon D., Young C., Jensen L.J., Vertegaal A.C.,
RA   Nielsen M.L.;
RT   "Site-specific mapping of the human SUMO proteome reveals co-modification
RT   with phosphorylation.";
RL   Nat. Struct. Mol. Biol. 24:325-336(2017).
CC   -!- FUNCTION: Component of the chaperonin-containing T-complex (TRiC), a
CC       molecular chaperone complex that assists the folding of proteins upon
CC       ATP hydrolysis (PubMed:25467444). The TRiC complex mediates the folding
CC       of WRAP53/TCAB1, thereby regulating telomere maintenance
CC       (PubMed:25467444). As part of the TRiC complex may play a role in the
CC       assembly of BBSome, a complex involved in ciliogenesis regulating
CC       transports vesicles to the cilia (PubMed:20080638). The TRiC complex
CC       plays a role in the folding of actin and tubulin (Probable).
CC       {ECO:0000269|PubMed:20080638, ECO:0000269|PubMed:25467444,
CC       ECO:0000305}.
CC   -!- SUBUNIT: Component of the chaperonin-containing T-complex (TRiC), a
CC       heterooligomeric complex of about 850 to 900 kDa that forms two stacked
CC       rings, 12 to 16 nm in diameter (PubMed:20080638, PubMed:25467444).
CC       Interacts with PACRG (PubMed:14532270). Interacts with DNAAF4 (By
CC       similarity). {ECO:0000250|UniProtKB:P42932,
CC       ECO:0000269|PubMed:14532270, ECO:0000269|PubMed:20080638,
CC       ECO:0000269|PubMed:25467444}.
CC   -!- INTERACTION:
CC       P50990; P46379-2: BAG6; NbExp=3; IntAct=EBI-356507, EBI-10988864;
CC       P50990; Q0VDD7: BRME1; NbExp=3; IntAct=EBI-356507, EBI-741210;
CC       P50990; P40227: CCT6A; NbExp=4; IntAct=EBI-356507, EBI-356687;
CC       P50990; Q6ZTQ4: CDHR3; NbExp=3; IntAct=EBI-356507, EBI-12143631;
CC       P50990; P42858: HTT; NbExp=4; IntAct=EBI-356507, EBI-466029;
CC       P50990; Q8N7X4: MAGEB6; NbExp=3; IntAct=EBI-356507, EBI-6447163;
CC       P50990; Q9UMY4: SNX12; NbExp=3; IntAct=EBI-356507, EBI-1752602;
CC       P50990; P17987: TCP1; NbExp=2; IntAct=EBI-356507, EBI-356553;
CC       P50990; P68363-2: TUBA1B; NbExp=3; IntAct=EBI-356507, EBI-25895616;
CC   -!- SUBCELLULAR LOCATION: Cytoplasm {ECO:0000269|PubMed:20080638}.
CC       Cytoplasm, cytoskeleton, microtubule organizing center, centrosome
CC       {ECO:0000269|PubMed:14654843, ECO:0000269|PubMed:20080638}. Cytoplasm,
CC       cytoskeleton, cilium basal body {ECO:0000250|UniProtKB:P42932}.
CC   -!- ALTERNATIVE PRODUCTS:
CC       Event=Alternative splicing; Named isoforms=3;
CC       Name=1;
CC         IsoId=P50990-1; Sequence=Displayed;
CC       Name=2;
CC         IsoId=P50990-2; Sequence=VSP_054692;
CC       Name=3;
CC         IsoId=P50990-3; Sequence=VSP_054691;
CC   -!- SIMILARITY: Belongs to the TCP-1 chaperonin family. {ECO:0000305}.
CC   -!- SEQUENCE CAUTION:
CC       Sequence=BAA02792.2; Type=Erroneous initiation; Evidence={ECO:0000305};
CC   ---------------------------------------------------------------------------
CC   Copyrighted by the UniProt Consortium, see https://www.uniprot.org/terms
CC   Distributed under the Creative Commons Attribution (CC BY 4.0) License
CC   ---------------------------------------------------------------------------
DR   EMBL; D13627; BAA02792.2; ALT_INIT; mRNA.
DR   EMBL; D42052; BAA07652.1; ALT_SEQ; Genomic_DNA.
DR   EMBL; AF129075; -; NOT_ANNOTATED_CDS; Genomic_DNA.
DR   EMBL; AK293705; BAG57138.1; -; mRNA.
DR   EMBL; AK298801; BAG60936.1; -; mRNA.
DR   EMBL; AL163249; CAB90433.1; -; Genomic_DNA.
DR   EMBL; BC072001; AAH72001.1; -; mRNA.
DR   EMBL; BC095470; AAH95470.1; -; mRNA.
DR   EMBL; Z37163; CAA85520.1; -; mRNA.
DR   CCDS; CCDS33528.1; -. [P50990-1]
DR   CCDS; CCDS68180.1; -. [P50990-3]
DR   CCDS; CCDS68181.1; -. [P50990-2]
DR   PIR; PC4021; PC4021.
DR   RefSeq; NP_001269836.1; NM_001282907.1. [P50990-2]
DR   RefSeq; NP_001269838.1; NM_001282909.1. [P50990-3]
DR   RefSeq; NP_006576.2; NM_006585.3. [P50990-1]
DR   PDB; 6NR8; EM; 7.80 A; H/P=14-527.
DR   PDB; 6NR9; EM; 8.50 A; H/P=14-527.
DR   PDB; 6NRA; EM; 7.70 A; H/P=14-527.
DR   PDB; 6NRB; EM; 8.70 A; H/P=14-527.
DR   PDB; 6NRC; EM; 8.30 A; H/P=14-527.
DR   PDB; 6NRD; EM; 8.20 A; H/P=14-527.
DR   PDB; 6QB8; EM; 3.97 A; Q/q=1-548.
DR   PDB; 7LUM; EM; 4.50 A; B/J=1-548.
DR   PDB; 7LUP; EM; 6.20 A; B/J=1-548.
DR   PDBsum; 6NR8; -.
DR   PDBsum; 6NR9; -.
DR   PDBsum; 6NRA; -.
DR   PDBsum; 6NRB; -.
DR   PDBsum; 6NRC; -.
DR   PDBsum; 6NRD; -.
DR   PDBsum; 6QB8; -.
DR   PDBsum; 7LUM; -.
DR   PDBsum; 7LUP; -.
DR   SMR; P50990; -.
DR   BioGRID; 115933; 326.
DR   ComplexPortal; CPX-6030; Chaperonin-containing T-complex.
DR   CORUM; P50990; -.
DR   DIP; DIP-38124N; -.
DR   IntAct; P50990; 185.
DR   MINT; P50990; -.
DR   STRING; 9606.ENSP00000286788; -.
DR   ChEMBL; CHEMBL4295775; -.
DR   iPTMnet; P50990; -.
DR   MetOSite; P50990; -.
DR   PhosphoSitePlus; P50990; -.
DR   SwissPalm; P50990; -.
DR   BioMuta; CCT8; -.
DR   DMDM; 9988062; -.
DR   OGP; P50990; -.
DR   REPRODUCTION-2DPAGE; IPI00784090; -.
DR   REPRODUCTION-2DPAGE; P50990; -.
DR   CPTAC; CPTAC-331; -.
DR   CPTAC; CPTAC-332; -.
DR   EPD; P50990; -.
DR   jPOST; P50990; -.
DR   MassIVE; P50990; -.
DR   MaxQB; P50990; -.
DR   PaxDb; P50990; -.
DR   PeptideAtlas; P50990; -.
DR   PRIDE; P50990; -.
DR   ProteomicsDB; 3972; -.
DR   ProteomicsDB; 4872; -.
DR   ProteomicsDB; 56272; -. [P50990-1]
DR   TopDownProteomics; P50990-1; -. [P50990-1]
DR   Antibodypedia; 6409; 281 antibodies.
DR   DNASU; 10694; -.
DR   Ensembl; ENST00000286788; ENSP00000286788; ENSG00000156261. [P50990-1]
DR   Ensembl; ENST00000540844; ENSP00000442730; ENSG00000156261. [P50990-3]
DR   Ensembl; ENST00000626972; ENSP00000486921; ENSG00000156261. [P50990-2]
DR   GeneID; 10694; -.
DR   KEGG; hsa:10694; -.
DR   UCSC; uc002ynb.5; human. [P50990-1]
DR   CTD; 10694; -.
DR   DisGeNET; 10694; -.
DR   GeneCards; CCT8; -.
DR   HGNC; HGNC:1623; CCT8.
DR   HPA; ENSG00000156261; Low tissue specificity.
DR   MIM; 617786; gene.
DR   neXtProt; NX_P50990; -.
DR   OpenTargets; ENSG00000156261; -.
DR   PharmGKB; PA26186; -.
DR   VEuPathDB; HostDB:ENSG00000156261.12; -.
DR   eggNOG; KOG0362; Eukaryota.
DR   GeneTree; ENSGT00550000074783; -.
DR   InParanoid; P50990; -.
DR   OMA; WGLKYAV; -.
DR   OrthoDB; 617040at2759; -.
DR   PhylomeDB; P50990; -.
DR   TreeFam; TF105699; -.
DR   BRENDA; 3.6.4.B10; 2681.
DR   PathwayCommons; P50990; -.
DR   Reactome; R-HSA-389957; Prefoldin mediated transfer of substrate to CCT/TriC.
DR   Reactome; R-HSA-389960; Formation of tubulin folding intermediates by CCT/TriC.
DR   Reactome; R-HSA-390450; Folding of actin by CCT/TriC.
DR   Reactome; R-HSA-390471; Association of TriC/CCT with target proteins during biosynthesis.
DR   Reactome; R-HSA-5620922; BBSome-mediated cargo-targeting to cilium.
DR   Reactome; R-HSA-6798695; Neutrophil degranulation.
DR   Reactome; R-HSA-6814122; Cooperation of PDCL (PhLP1) and TRiC/CCT in G-protein beta folding.
DR   BioGRID-ORCS; 10694; 767 hits in 963 CRISPR screens.
DR   ChiTaRS; CCT8; human.
DR   GeneWiki; CCT8; -.
DR   GenomeRNAi; 10694; -.
DR   Pharos; P50990; Tbio.
DR   PRO; PR:P50990; -.
DR   Proteomes; UP000005640; Chromosome 21.
DR   RNAct; P50990; protein.
DR   Bgee; ENSG00000156261; Expressed in material anatomical entity and 251 other tissues.
DR   ExpressionAtlas; P50990; baseline and differential.
DR   Genevisible; P50990; HS.
DR   GO; GO:0035578; C:azurophil granule lumen; TAS:Reactome.
DR   GO; GO:0044297; C:cell body; IEA:Ensembl.
DR   GO; GO:0005813; C:centrosome; IDA:MGI.
DR   GO; GO:0005832; C:chaperonin-containing T-complex; IDA:UniProtKB.
DR   GO; GO:0005929; C:cilium; IEA:UniProtKB-KW.
DR   GO; GO:0005737; C:cytoplasm; IDA:BHF-UCL.
DR   GO; GO:0005829; C:cytosol; IDA:HPA.
DR   GO; GO:0070062; C:extracellular exosome; HDA:UniProtKB.
DR   GO; GO:0005576; C:extracellular region; TAS:Reactome.
DR   GO; GO:1904813; C:ficolin-1-rich granule lumen; TAS:Reactome.
DR   GO; GO:0045111; C:intermediate filament cytoskeleton; IDA:HPA.
DR   GO; GO:0005874; C:microtubule; IDA:UniProtKB.
DR   GO; GO:0005654; C:nucleoplasm; IDA:HPA.
DR   GO; GO:0034774; C:secretory granule lumen; TAS:Reactome.
DR   GO; GO:0002199; C:zona pellucida receptor complex; IEA:Ensembl.
DR   GO; GO:0005524; F:ATP binding; IEA:UniProtKB-KW.
DR   GO; GO:0016887; F:ATPase activity; TAS:ProtInc.
DR   GO; GO:0045296; F:cadherin binding; HDA:BHF-UCL.
DR   GO; GO:0044183; F:protein folding chaperone; IDA:FlyBase.
DR   GO; GO:0051082; F:unfolded protein binding; IBA:GO_Central.
DR   GO; GO:0007339; P:binding of sperm to zona pellucida; IEA:Ensembl.
DR   GO; GO:0043312; P:neutrophil degranulation; TAS:Reactome.
DR   GO; GO:0046931; P:pore complex assembly; IEA:Ensembl.
DR   GO; GO:1904851; P:positive regulation of establishment of protein localization to telomere; IMP:BHF-UCL.
DR   GO; GO:1904871; P:positive regulation of protein localization to Cajal body; HMP:BHF-UCL.
DR   GO; GO:1904874; P:positive regulation of telomerase RNA localization to Cajal body; HMP:BHF-UCL.
DR   GO; GO:0032212; P:positive regulation of telomere maintenance via telomerase; IMP:BHF-UCL.
DR   GO; GO:0006457; P:protein folding; IDA:FlyBase.
DR   GO; GO:0050821; P:protein stabilization; IMP:BHF-UCL.
DR   GO; GO:1901998; P:toxin transport; IEA:Ensembl.
DR   CDD; cd03341; TCP1_theta; 1.
DR   Gene3D; 1.10.560.10; -; 1.
DR   Gene3D; 3.30.260.10; -; 1.
DR   Gene3D; 3.50.7.10; -; 1.
DR   InterPro; IPR012721; Chap_CCT_theta.
DR   InterPro; IPR017998; Chaperone_TCP-1.
DR   InterPro; IPR002194; Chaperonin_TCP-1_CS.
DR   InterPro; IPR002423; Cpn60/TCP-1.
DR   InterPro; IPR027409; GroEL-like_apical_dom_sf.
DR   InterPro; IPR027413; GROEL-like_equatorial_sf.
DR   InterPro; IPR027410; TCP-1-like_intermed_sf.
DR   Pfam; PF00118; Cpn60_TCP1; 1.
DR   PRINTS; PR00304; TCOMPLEXTCP1.
DR   SUPFAM; SSF48592; SSF48592; 1.
DR   SUPFAM; SSF52029; SSF52029; 1.
DR   SUPFAM; SSF54849; SSF54849; 1.
DR   TIGRFAMs; TIGR02346; chap_CCT_theta; 1.
DR   PROSITE; PS00750; TCP1_1; 1.
DR   PROSITE; PS00751; TCP1_2; 1.
DR   PROSITE; PS00995; TCP1_3; 1.
PE   1: Evidence at protein level;
KW   3D-structure; Acetylation; Alternative splicing; ATP-binding;
KW   Cell projection; Chaperone; Cilium; Cytoplasm; Cytoskeleton;
KW   Direct protein sequencing; Isopeptide bond; Nucleotide-binding;
KW   Phosphoprotein; Reference proteome; Ubl conjugation.
FT   INIT_MET        1
FT                   /note="Removed"
FT                   /evidence="ECO:0000269|Ref.7"
FT   CHAIN           2..548
FT                   /note="T-complex protein 1 subunit theta"
FT                   /id="PRO_0000128373"
FT   REGION          529..548
FT                   /note="Disordered"
FT                   /evidence="ECO:0000256|SAM:MobiDB-lite"
FT   MOD_RES         2
FT                   /note="N-acetylalanine"
FT                   /evidence="ECO:0000269|Ref.7"
FT   MOD_RES         23
FT                   /note="Phosphoserine"
FT                   /evidence="ECO:0007744|PubMed:18669648,
FT                   ECO:0007744|PubMed:19690332, ECO:0007744|PubMed:23186163"
FT   MOD_RES         30
FT                   /note="Phosphotyrosine"
FT                   /evidence="ECO:0007744|PubMed:19690332,
FT                   ECO:0007744|PubMed:23186163"
FT   MOD_RES         162
FT                   /note="Phosphoserine"
FT                   /evidence="ECO:0007744|PubMed:23186163"
FT   MOD_RES         213
FT                   /note="Phosphoserine"
FT                   /evidence="ECO:0007744|PubMed:23186163"
FT   MOD_RES         269
FT                   /note="Phosphoserine"
FT                   /evidence="ECO:0007744|PubMed:23186163"
FT   MOD_RES         317
FT                   /note="Phosphoserine"
FT                   /evidence="ECO:0007744|PubMed:23186163"
FT   MOD_RES         318
FT                   /note="N6-acetyllysine"
FT                   /evidence="ECO:0007744|PubMed:19608861"
FT   MOD_RES         400
FT                   /note="N6-acetyllysine"
FT                   /evidence="ECO:0007744|PubMed:19608861"
FT   MOD_RES         466
FT                   /note="N6-acetyllysine"
FT                   /evidence="ECO:0007744|PubMed:19608861"
FT   MOD_RES         505
FT                   /note="Phosphotyrosine"
FT                   /evidence="ECO:0007744|PubMed:15592455"
FT   MOD_RES         537
FT                   /note="Phosphoserine"
FT                   /evidence="ECO:0007744|PubMed:23186163"
FT   CROSSLNK        224
FT                   /note="Glycyl lysine isopeptide (Lys-Gly) (interchain with
FT                   G-Cter in SUMO2)"
FT                   /evidence="ECO:0007744|PubMed:28112733"
FT   CROSSLNK        254
FT                   /note="Glycyl lysine isopeptide (Lys-Gly) (interchain with
FT                   G-Cter in SUMO2)"
FT                   /evidence="ECO:0007744|PubMed:28112733"
FT   CROSSLNK        260
FT                   /note="Glycyl lysine isopeptide (Lys-Gly) (interchain with
FT                   G-Cter in SUMO2)"
FT                   /evidence="ECO:0007744|PubMed:28112733"
FT   CROSSLNK        459
FT                   /note="Glycyl lysine isopeptide (Lys-Gly) (interchain with
FT                   G-Cter in SUMO1)"
FT                   /evidence="ECO:0007744|PubMed:25114211"
FT   CROSSLNK        534
FT                   /note="Glycyl lysine isopeptide (Lys-Gly) (interchain with
FT                   G-Cter in SUMO2)"
FT                   /evidence="ECO:0007744|PubMed:25218447,
FT                   ECO:0007744|PubMed:25755297, ECO:0007744|PubMed:28112733"
FT   CROSSLNK        539
FT                   /note="Glycyl lysine isopeptide (Lys-Gly) (interchain with
FT                   G-Cter in SUMO2)"
FT                   /evidence="ECO:0007744|PubMed:25755297,
FT                   ECO:0007744|PubMed:28112733"
FT   VAR_SEQ         1..78
FT                   /note="MALHVPKAPGFAQMLKEGAKHFSGLEEAVYRNIQACKELAQTTRTAYGPNGM
FT                   NKMVINHLEKLFVTNDAATILRELEV -> MDQMV (in isoform 3)"
FT                   /evidence="ECO:0000303|PubMed:14702039"
FT                   /id="VSP_054691"
FT   VAR_SEQ         1..20
FT                   /note="MALHVPKAPGFAQMLKEGAK -> M (in isoform 2)"
FT                   /evidence="ECO:0000303|PubMed:14702039"
FT                   /id="VSP_054692"
FT   VARIANT         4
FT                   /note="H -> Q (in dbSNP:rs16983693)"
FT                   /id="VAR_052270"
FT   VARIANT         409
FT                   /note="V -> I (in dbSNP:rs8129954)"
FT                   /id="VAR_052271"
FT   CONFLICT        50
FT                   /note="N -> K (in Ref. 1 and 2)"
FT                   /evidence="ECO:0000305"
FT   CONFLICT        391
FT                   /note="A -> V (in Ref. 1; BAA02792)"
FT                   /evidence="ECO:0000305"
SQ   SEQUENCE   548 AA;  59621 MW;  566A6622BC2D15E9 CRC64;
     MALHVPKAPG FAQMLKEGAK HFSGLEEAVY RNIQACKELA QTTRTAYGPN GMNKMVINHL
     EKLFVTNDAA TILRELEVQH PAAKMIVMAS HMQEQEVGDG TNFVLVFAGA LLELAEELLR
     IGLSVSEVIE GYEIACRKAH EILPNLVCCS AKNLRDIDEV SSLLRTSIMS KQYGNEVFLA
     KLIAQACVSI FPDSGHFNVD NIRVCKILGS GISSSSVLHG MVFKKETEGD VTSVKDAKIA
     VYSCPFDGMI TETKGTVLIK TAEELMNFSK GEENLMDAQV KAIADTGANV VVTGGKVADM
     ALHYANKYNI MLVRLNSKWD LRRLCKTVGA TALPRLTPPV LEEMGHCDSV YLSEVGDTQV
     VVFKHEKEDG AISTIVLRGS TDNLMDDIER AVDDGVNTFK VLTRDKRLVP GGGATEIELA
     KQITSYGETC PGLEQYAIKK FAEAFEAIPR ALAENSGVKA NEVISKLYAV HQEGNKNVGL
     DIEAEVPAVK DMLEAGILDT YLGKYWAIKL ATNAAVTVLR VDQIIMAKPA GGPKPPSGKK
     DWDDDQND
```

|  |
| --- |
| **Mascot:** http://www.matrixscience.com/ |
