# Supplementary material for: Chlorpromazine induces cytotoxic autophagy in glioblastoma cells via endoplasmic reticulum stress and unfolded protein response
Source: J Exp Clin Cancer Res. 2021 Nov 5;40:347. doi: 10.1186/s13046-021-02144-w (PMC8569984; doi:10.1186/s13046-021-02144-w)
Supplement: Supplementary file 1 — Additional file 1. [file 13046_2021_2144_MOESM1_ESM.zip › MASCOT IDs/Mascot Search Results_ TCPZ_HUMAN.html]

Mascot Search Results: TCPZ\_HUMAN
 

# MASCOT Search Results

## Protein View: TCPZ\_HUMAN

### T-complex protein 1 subunit zeta OS=Homo sapiens OX=9606 GN=CCT6A PE=1 SV=3

|  |  |
| --- | --- |
| Database: | SwissProt |
| Score: | 41 |
| Monoisotopic mass (Mr): | 58444 |
| Calculated pI: | 6.23 |
| Taxonomy: | Homo sapiens |

Sequence similarity is available as an NCBI BLAST search of TCPZ\_HUMAN against nr.

### Search parameters

|  |  |
| --- | --- |
| MS data file: | `ppw_B4_156283227800.txt` |
| Enzyme: | Trypsin: cuts C-term side of KR unless next residue is P. |
| Fixed modifications: | Carbamidomethyl (C) |

### Protein sequence coverage: 6%

Matched peptides shown in ***bold red***.

|  |  |  |  |  |  |
| --- | --- | --- | --- | --- | --- |
| `1` | `MAAVKTLNPK` | `AEVARAQAAL` | `AVNISAARGL` | `QDVLRTNLGP` | `KGTMKMLVSG` |
| `51` | `AGDIKLTKDG` | `NVLLHEMQIQ` | `HPTASLIAKV` | `ATAQDDITGD` | `GTTSNVLIIG` |
| `101` | `ELLKQADLYI` | `SEGLHPRIIT` | `EGFEAAKEKA` | `LQFLEEVKVS` | `REMDRETLID` |
| `151` | `VARTSLRTKV` | `HAELADVLTE` | `AVVDSILAIK` | `KQDEPIDLFM` | `IEIMEMKHKS` |
| `201` | `ETDTSLIRGL` | `VLDHGARHPD` | `MKKRVEDAYI` | `LTCNVSLEYE` | `KTEVNSGFFY` |
| `251` | `KSAEEREKLV` | `KAERKFIEDR` | `VKKIIELKRK` | `VCGDSDKGFV` | `VINQKGIDPF` |
| `301` | `SLDALSKEGI` | `VALRRAKRRN` | `MERLTLACGG` | `VALNSFDDLS` | `PDCLGHAGLV` |
| `351` | `YEYTLGEEKF` | `TFIEKCNNPR` | `SVTLLIKGPN` | `KHTLTQIKDA` | `VRDGLRAVKN` |
| `401` | `AIDDGCVVPG` | `AGAVEVAMAE` | `ALIKHKPSVK` | `GRAQLGVQAF` | `ADALLIIPKV` |
| `451` | `LAQNSGFDLQ` | `ETLVKIQAEH` | `SESGQLVGVD` | `LNTGEPMVAA` | `EVGVWDNYCV` |
| `501` | `KKQLLHSCTV` | `IATNILLVDE` | `IMRAGMSSLK` | `G` |  |

Unformatted sequence string: 531 residues (for pasting into other applications).

|  |  |  |  |
| --- | --- | --- | --- |
| Sort by | residue number | increasing mass | decreasing mass |
| Show | matched peptides only | predicted peptides also |  |

| Query | Start | – | End | Observed | Mr(expt) | Mr(calc) | ppm | M | Score | Expect | Rank | U | Peptide |
| --- | --- | --- | --- | --- | --- | --- | --- | --- | --- | --- | --- | --- | --- |
| 36 | 130 | – | 141 | 1418.8346 | 1417.8273 | 1417.7929 | 24.3 | 1 | 36 | 0.0073 | 1Score **> 28** indicates **identity** Score **> 25** indicates **homology** | U | K.ALQFLEEVKVSR.E |
| 77 | 296 | – | 315 | 2157.2463 | 2156.2390 | 2156.1953 | 20.3 | 2 | 29 | 0.027 | 1Score **> 26** indicates **identity** Score **> 25** indicates **homology** | U | K.GIDPFSLDALSKEGIVALRR.A |

---

```
ID   TCPZ_HUMAN              Reviewed;         531 AA.
AC   P40227; A6NCD2; Q3KP28; Q75LP4; Q96S46;
DT   01-FEB-1995, integrated into UniProtKB/Swiss-Prot.
DT   23-JAN-2007, sequence version 3.
DT   02-JUN-2021, entry version 204.
DE   RecName: Full=T-complex protein 1 subunit zeta;
DE            Short=TCP-1-zeta;
DE   AltName: Full=Acute morphine dependence-related protein 2;
DE   AltName: Full=CCT-zeta-1;
DE   AltName: Full=HTR3;
DE   AltName: Full=Tcp20 {ECO:0000303|PubMed:8034610};
GN   Name=CCT6A; Synonyms=CCT6, CCTZ;
OS   Homo sapiens (Human).
OC   Eukaryota; Metazoa; Chordata; Craniata; Vertebrata; Euteleostomi; Mammalia;
OC   Eutheria; Euarchontoglires; Primates; Haplorrhini; Catarrhini; Hominidae;
OC   Homo.
OX   NCBI_TaxID=9606;
RN   [1]
RP   NUCLEOTIDE SEQUENCE [MRNA] (ISOFORM 1), PROTEIN SEQUENCE OF 324-339, AND
RP   SUBCELLULAR LOCATION.
RX   PubMed=8034610;
RA   Li W.-Z., Lin P., Frydman J., Boal T.R., Cardillo T.S., Richard L.M.,
RA   Toth D., Lichtman M.A., Hartl F.-U., Sherman F., Segel G.B.;
RT   "Tcp20, a subunit of the eukaryotic TRiC chaperonin from humans and
RT   yeast.";
RL   J. Biol. Chem. 269:18616-18622(1994).
RN   [2]
RP   NUCLEOTIDE SEQUENCE [MRNA] (ISOFORM 1).
RA   Lee Y.-K., Yoo Y.-D.;
RT   "Homo sapiens chaperonin mRNA sequence.";
RL   Submitted (MAY-2001) to the EMBL/GenBank/DDBJ databases.
RN   [3]
RP   NUCLEOTIDE SEQUENCE [MRNA] (ISOFORM 1).
RA   Wang H., Gao X., Li L., Wang B., Huang Y., Han J.;
RT   "Homo sapiens chaperonin (MoDP) mRNA expressed in SH-SY5Y neuroblastoma
RT   cells.";
RL   Submitted (JUN-2001) to the EMBL/GenBank/DDBJ databases.
RN   [4]
RP   NUCLEOTIDE SEQUENCE [LARGE SCALE GENOMIC DNA].
RX   PubMed=12853948; DOI=10.1038/nature01782;
RA   Hillier L.W., Fulton R.S., Fulton L.A., Graves T.A., Pepin K.H.,
RA   Wagner-McPherson C., Layman D., Maas J., Jaeger S., Walker R., Wylie K.,
RA   Sekhon M., Becker M.C., O'Laughlin M.D., Schaller M.E., Fewell G.A.,
RA   Delehaunty K.D., Miner T.L., Nash W.E., Cordes M., Du H., Sun H.,
RA   Edwards J., Bradshaw-Cordum H., Ali J., Andrews S., Isak A., Vanbrunt A.,
RA   Nguyen C., Du F., Lamar B., Courtney L., Kalicki J., Ozersky P.,
RA   Bielicki L., Scott K., Holmes A., Harkins R., Harris A., Strong C.M.,
RA   Hou S., Tomlinson C., Dauphin-Kohlberg S., Kozlowicz-Reilly A., Leonard S.,
RA   Rohlfing T., Rock S.M., Tin-Wollam A.-M., Abbott A., Minx P., Maupin R.,
RA   Strowmatt C., Latreille P., Miller N., Johnson D., Murray J.,
RA   Woessner J.P., Wendl M.C., Yang S.-P., Schultz B.R., Wallis J.W.,
RA   Spieth J., Bieri T.A., Nelson J.O., Berkowicz N., Wohldmann P.E.,
RA   Cook L.L., Hickenbotham M.T., Eldred J., Williams D., Bedell J.A.,
RA   Mardis E.R., Clifton S.W., Chissoe S.L., Marra M.A., Raymond C., Haugen E.,
RA   Gillett W., Zhou Y., James R., Phelps K., Iadanoto S., Bubb K., Simms E.,
RA   Levy R., Clendenning J., Kaul R., Kent W.J., Furey T.S., Baertsch R.A.,
RA   Brent M.R., Keibler E., Flicek P., Bork P., Suyama M., Bailey J.A.,
RA   Portnoy M.E., Torrents D., Chinwalla A.T., Gish W.R., Eddy S.R.,
RA   McPherson J.D., Olson M.V., Eichler E.E., Green E.D., Waterston R.H.,
RA   Wilson R.K.;
RT   "The DNA sequence of human chromosome 7.";
RL   Nature 424:157-164(2003).
RN   [5]
RP   NUCLEOTIDE SEQUENCE [LARGE SCALE MRNA] (ISOFORM 1), AND NUCLEOTIDE SEQUENCE
RP   [LARGE SCALE MRNA] OF 1-233 (ISOFORM 2).
RC   TISSUE=Lung carcinoma;
RX   PubMed=15489334; DOI=10.1101/gr.2596504;
RG   The MGC Project Team;
RT   "The status, quality, and expansion of the NIH full-length cDNA project:
RT   the Mammalian Gene Collection (MGC).";
RL   Genome Res. 14:2121-2127(2004).
RN   [6]
RP   NUCLEOTIDE SEQUENCE [MRNA] OF 98-531 (ISOFORM 1).
RC   TISSUE=B-cell;
RX   PubMed=1352881; DOI=10.1073/pnas.89.13.6060;
RA   Segel G.B., Boal T.R., Cardillo T.S., Murant F.G., Lichtman M.A.,
RA   Sherman F.;
RT   "Isolation of a gene encoding a chaperonin-like protein by complementation
RT   of yeast amino acid transport mutants with human cDNA.";
RL   Proc. Natl. Acad. Sci. U.S.A. 89:6060-6064(1992).
RN   [7]
RP   PROTEIN SEQUENCE OF 2-15.
RC   TISSUE=Platelet;
RX   PubMed=12665801; DOI=10.1038/nbt810;
RA   Gevaert K., Goethals M., Martens L., Van Damme J., Staes A., Thomas G.R.,
RA   Vandekerckhove J.;
RT   "Exploring proteomes and analyzing protein processing by mass spectrometric
RT   identification of sorted N-terminal peptides.";
RL   Nat. Biotechnol. 21:566-569(2003).
RN   [8]
RP   PROTEIN SEQUENCE OF 105-117 AND 160-180, AND IDENTIFICATION BY MASS
RP   SPECTROMETRY.
RC   TISSUE=Brain, and Cajal-Retzius cell;
RA   Lubec G., Vishwanath V.;
RL   Submitted (MAR-2007) to UniProtKB.
RN   [9]
RP   INTERACTION WITH PACRG.
RX   PubMed=14532270; DOI=10.1074/jbc.m309655200;
RA   Imai Y., Soda M., Murakami T., Shoji M., Abe K., Takahashi R.;
RT   "A product of the human gene adjacent to parkin is a component of Lewy
RT   bodies and suppresses Pael receptor-induced cell death.";
RL   J. Biol. Chem. 278:51901-51910(2003).
RN   [10]
RP   ACETYLATION [LARGE SCALE ANALYSIS] AT LYS-199; LYS-365; LYS-377 AND
RP   LYS-388, AND IDENTIFICATION BY MASS SPECTROMETRY [LARGE SCALE ANALYSIS].
RX   PubMed=19608861; DOI=10.1126/science.1175371;
RA   Choudhary C., Kumar C., Gnad F., Nielsen M.L., Rehman M., Walther T.C.,
RA   Olsen J.V., Mann M.;
RT   "Lysine acetylation targets protein complexes and co-regulates major
RT   cellular functions.";
RL   Science 325:834-840(2009).
RN   [11]
RP   IDENTIFICATION BY MASS SPECTROMETRY [LARGE SCALE ANALYSIS].
RX   PubMed=21269460; DOI=10.1186/1752-0509-5-17;
RA   Burkard T.R., Planyavsky M., Kaupe I., Breitwieser F.P., Buerckstuemmer T.,
RA   Bennett K.L., Superti-Furga G., Colinge J.;
RT   "Initial characterization of the human central proteome.";
RL   BMC Syst. Biol. 5:17-17(2011).
RN   [12]
RP   ACETYLATION [LARGE SCALE ANALYSIS] AT ALA-2, CLEAVAGE OF INITIATOR
RP   METHIONINE [LARGE SCALE ANALYSIS], AND IDENTIFICATION BY MASS SPECTROMETRY
RP   [LARGE SCALE ANALYSIS].
RX   PubMed=22814378; DOI=10.1073/pnas.1210303109;
RA   Van Damme P., Lasa M., Polevoda B., Gazquez C., Elosegui-Artola A.,
RA   Kim D.S., De Juan-Pardo E., Demeyer K., Hole K., Larrea E., Timmerman E.,
RA   Prieto J., Arnesen T., Sherman F., Gevaert K., Aldabe R.;
RT   "N-terminal acetylome analyses and functional insights of the N-terminal
RT   acetyltransferase NatB.";
RL   Proc. Natl. Acad. Sci. U.S.A. 109:12449-12454(2012).
RN   [13]
RP   PHOSPHORYLATION [LARGE SCALE ANALYSIS] AT SER-205, AND IDENTIFICATION BY
RP   MASS SPECTROMETRY [LARGE SCALE ANALYSIS].
RC   TISSUE=Erythroleukemia;
RX   PubMed=23186163; DOI=10.1021/pr300630k;
RA   Zhou H., Di Palma S., Preisinger C., Peng M., Polat A.N., Heck A.J.,
RA   Mohammed S.;
RT   "Toward a comprehensive characterization of a human cancer cell
RT   phosphoproteome.";
RL   J. Proteome Res. 12:260-271(2013).
RN   [14]
RP   FUNCTION, AND IDENTIFICATION IN THE CHAPERONIN-CONTAINING T-COMPLEX.
RX   PubMed=25467444; DOI=10.1016/j.cell.2014.10.059;
RA   Freund A., Zhong F.L., Venteicher A.S., Meng Z., Veenstra T.D., Frydman J.,
RA   Artandi S.E.;
RT   "Proteostatic control of telomerase function through TRiC-mediated folding
RT   of TCAB1.";
RL   Cell 159:1389-1403(2014).
RN   [15]
RP   IDENTIFICATION BY MASS SPECTROMETRY [LARGE SCALE ANALYSIS].
RC   TISSUE=Liver;
RX   PubMed=24275569; DOI=10.1016/j.jprot.2013.11.014;
RA   Bian Y., Song C., Cheng K., Dong M., Wang F., Huang J., Sun D., Wang L.,
RA   Ye M., Zou H.;
RT   "An enzyme assisted RP-RPLC approach for in-depth analysis of human liver
RT   phosphoproteome.";
RL   J. Proteomics 96:253-262(2014).
RN   [16]
RP   IDENTIFICATION BY MASS SPECTROMETRY [LARGE SCALE ANALYSIS].
RX   PubMed=25944712; DOI=10.1002/pmic.201400617;
RA   Vaca Jacome A.S., Rabilloud T., Schaeffer-Reiss C., Rompais M., Ayoub D.,
RA   Lane L., Bairoch A., Van Dorsselaer A., Carapito C.;
RT   "N-terminome analysis of the human mitochondrial proteome.";
RL   Proteomics 15:2519-2524(2015).
RN   [17]
RP   SUMOYLATION [LARGE SCALE ANALYSIS] AT LYS-251, AND IDENTIFICATION BY MASS
RP   SPECTROMETRY [LARGE SCALE ANALYSIS].
RX   PubMed=28112733; DOI=10.1038/nsmb.3366;
RA   Hendriks I.A., Lyon D., Young C., Jensen L.J., Vertegaal A.C.,
RA   Nielsen M.L.;
RT   "Site-specific mapping of the human SUMO proteome reveals co-modification
RT   with phosphorylation.";
RL   Nat. Struct. Mol. Biol. 24:325-336(2017).
CC   -!- FUNCTION: Component of the chaperonin-containing T-complex (TRiC), a
CC       molecular chaperone complex that assists the folding of proteins upon
CC       ATP hydrolysis (PubMed:25467444). The TRiC complex mediates the folding
CC       of WRAP53/TCAB1, thereby regulating telomere maintenance
CC       (PubMed:25467444). The TRiC complex plays a role in the folding of
CC       actin and tubulin (Probable). {ECO:0000269|PubMed:25467444,
CC       ECO:0000305}.
CC   -!- SUBUNIT: Component of the chaperonin-containing T-complex (TRiC), a
CC       heterooligomeric complex of about 850 to 900 kDa that forms two stacked
CC       rings, 12 to 16 nm in diameter (PubMed:25467444). Interacts with PACRG
CC       (PubMed:14532270). {ECO:0000269|PubMed:14532270,
CC       ECO:0000269|PubMed:25467444}.
CC   -!- INTERACTION:
CC       P40227; P05067: APP; NbExp=3; IntAct=EBI-356687, EBI-77613;
CC       P40227; P42331-2: ARHGAP25; NbExp=3; IntAct=EBI-356687, EBI-21499901;
CC       P40227; P54253: ATXN1; NbExp=3; IntAct=EBI-356687, EBI-930964;
CC       P40227; P54252: ATXN3; NbExp=3; IntAct=EBI-356687, EBI-946046;
CC       P40227; Q8WUW1: BRK1; NbExp=3; IntAct=EBI-356687, EBI-2837444;
CC       P40227; P50990: CCT8; NbExp=4; IntAct=EBI-356687, EBI-356507;
CC       P40227; P42858: HTT; NbExp=10; IntAct=EBI-356687, EBI-466029;
CC       P40227; P17612: PRKACA; NbExp=3; IntAct=EBI-356687, EBI-476586;
CC       P40227; Q16560-2: SNRNP35; NbExp=3; IntAct=EBI-356687, EBI-12938570;
CC       P40227; P37173: TGFBR2; NbExp=3; IntAct=EBI-356687, EBI-296151;
CC       P40227; Q9BZY9: TRIM31; NbExp=3; IntAct=EBI-356687, EBI-747544;
CC   -!- SUBCELLULAR LOCATION: Cytoplasm {ECO:0000269|PubMed:8034610}.
CC   -!- ALTERNATIVE PRODUCTS:
CC       Event=Alternative splicing; Named isoforms=2;
CC       Name=1;
CC         IsoId=P40227-1; Sequence=Displayed;
CC       Name=2;
CC         IsoId=P40227-2; Sequence=VSP_044918;
CC   -!- SIMILARITY: Belongs to the TCP-1 chaperonin family. {ECO:0000305}.
CC   -!- SEQUENCE CAUTION:
CC       Sequence=AAA58676.1; Type=Erroneous initiation; Evidence={ECO:0000305};
CC       Sequence=BU540578; Type=Miscellaneous discrepancy; Note=Several sequencing errors.; Evidence={ECO:0000305};
CC   ---------------------------------------------------------------------------
CC   Copyrighted by the UniProt Consortium, see https://www.uniprot.org/terms
CC   Distributed under the Creative Commons Attribution (CC BY 4.0) License
CC   ---------------------------------------------------------------------------
DR   EMBL; L27706; AAA61061.1; -; mRNA.
DR   EMBL; AF385084; AAK61354.1; -; mRNA.
DR   EMBL; AB063318; BAB61032.1; -; mRNA.
DR   EMBL; AC092101; AAS07451.1; -; Genomic_DNA.
DR   EMBL; AC092579; -; NOT_ANNOTATED_CDS; Genomic_DNA.
DR   EMBL; BC106942; AAI06943.1; -; mRNA.
DR   EMBL; BU540578; -; NOT_ANNOTATED_CDS; mRNA.
DR   EMBL; M94083; AAA58676.1; ALT_INIT; mRNA.
DR   CCDS; CCDS34640.1; -. [P40227-2]
DR   CCDS; CCDS5523.1; -. [P40227-1]
DR   PIR; S48087; S48087.
DR   RefSeq; NP_001009186.1; NM_001009186.1. [P40227-2]
DR   RefSeq; NP_001753.1; NM_001762.3. [P40227-1]
DR   PDB; 6NR8; EM; 7.80 A; F/N=11-525.
DR   PDB; 6NR9; EM; 8.50 A; F/N=11-525.
DR   PDB; 6NRA; EM; 7.70 A; F/N=11-525.
DR   PDB; 6NRB; EM; 8.70 A; F/N=11-525.
DR   PDB; 6NRC; EM; 8.30 A; F/N=11-525.
DR   PDB; 6NRD; EM; 8.20 A; F/N=11-525.
DR   PDB; 6QB8; EM; 3.97 A; Z/z=1-531.
DR   PDB; 7LUM; EM; 4.50 A; A/I=1-531.
DR   PDB; 7LUP; EM; 6.20 A; A/I=1-531.
DR   PDBsum; 6NR8; -.
DR   PDBsum; 6NR9; -.
DR   PDBsum; 6NRA; -.
DR   PDBsum; 6NRB; -.
DR   PDBsum; 6NRC; -.
DR   PDBsum; 6NRD; -.
DR   PDBsum; 6QB8; -.
DR   PDBsum; 7LUM; -.
DR   PDBsum; 7LUP; -.
DR   SMR; P40227; -.
DR   BioGRID; 107346; 385.
DR   ComplexPortal; CPX-6030; Chaperonin-containing T-complex.
DR   CORUM; P40227; -.
DR   DIP; DIP-33558N; -.
DR   IntAct; P40227; 249.
DR   MINT; P40227; -.
DR   STRING; 9606.ENSP00000275603; -.
DR   iPTMnet; P40227; -.
DR   MetOSite; P40227; -.
DR   PhosphoSitePlus; P40227; -.
DR   SwissPalm; P40227; -.
DR   BioMuta; CCT6A; -.
DR   DMDM; 730922; -.
DR   DOSAC-COBS-2DPAGE; P40227; -.
DR   REPRODUCTION-2DPAGE; IPI00027626; -.
DR   REPRODUCTION-2DPAGE; P40227; -.
DR   SWISS-2DPAGE; P40227; -.
DR   UCD-2DPAGE; P40227; -.
DR   CPTAC; CPTAC-1643; -.
DR   CPTAC; CPTAC-41; -.
DR   CPTAC; CPTAC-42; -.
DR   EPD; P40227; -.
DR   jPOST; P40227; -.
DR   MassIVE; P40227; -.
DR   MaxQB; P40227; -.
DR   PaxDb; P40227; -.
DR   PeptideAtlas; P40227; -.
DR   PRIDE; P40227; -.
DR   ProteomicsDB; 55353; -. [P40227-1]
DR   ProteomicsDB; 824; -.
DR   Antibodypedia; 44791; 147 antibodies.
DR   DNASU; 908; -.
DR   Ensembl; ENST00000275603; ENSP00000275603; ENSG00000146731. [P40227-1]
DR   Ensembl; ENST00000335503; ENSP00000352019; ENSG00000146731. [P40227-2]
DR   GeneID; 908; -.
DR   KEGG; hsa:908; -.
DR   UCSC; uc003trl.2; human. [P40227-1]
DR   CTD; 908; -.
DR   DisGeNET; 908; -.
DR   GeneCards; CCT6A; -.
DR   HGNC; HGNC:1620; CCT6A.
DR   HPA; ENSG00000146731; Low tissue specificity.
DR   MIM; 104613; gene.
DR   neXtProt; NX_P40227; -.
DR   OpenTargets; ENSG00000146731; -.
DR   PharmGKB; PA26183; -.
DR   VEuPathDB; HostDB:ENSG00000146731.10; -.
DR   eggNOG; KOG0359; Eukaryota.
DR   GeneTree; ENSGT00940000154631; -.
DR   HOGENOM; CLU_008891_3_1_1; -.
DR   InParanoid; P40227; -.
DR   OMA; TMQHRTA; -.
DR   PhylomeDB; P40227; -.
DR   TreeFam; TF106333; -.
DR   BRENDA; 3.6.4.B10; 2681.
DR   PathwayCommons; P40227; -.
DR   Reactome; R-HSA-389957; Prefoldin mediated transfer of substrate to CCT/TriC.
DR   Reactome; R-HSA-389960; Formation of tubulin folding intermediates by CCT/TriC.
DR   Reactome; R-HSA-390450; Folding of actin by CCT/TriC.
DR   Reactome; R-HSA-390471; Association of TriC/CCT with target proteins during biosynthesis.
DR   Reactome; R-HSA-6814122; Cooperation of PDCL (PhLP1) and TRiC/CCT in G-protein beta folding.
DR   BioGRID-ORCS; 908; 742 hits in 980 CRISPR screens.
DR   ChiTaRS; CCT6A; human.
DR   GeneWiki; CCT6A; -.
DR   GenomeRNAi; 908; -.
DR   Pharos; P40227; Tbio.
DR   PRO; PR:P40227; -.
DR   Proteomes; UP000005640; Chromosome 7.
DR   RNAct; P40227; protein.
DR   Bgee; ENSG00000146731; Expressed in cortical plate and 246 other tissues.
DR   ExpressionAtlas; P40227; baseline and differential.
DR   Genevisible; P40227; HS.
DR   GO; GO:0005832; C:chaperonin-containing T-complex; IDA:UniProtKB.
DR   GO; GO:0005737; C:cytoplasm; TAS:ProtInc.
DR   GO; GO:0005829; C:cytosol; IDA:HPA.
DR   GO; GO:0070062; C:extracellular exosome; HDA:UniProtKB.
DR   GO; GO:0005874; C:microtubule; IDA:UniProtKB.
DR   GO; GO:0005524; F:ATP binding; IEA:UniProtKB-KW.
DR   GO; GO:0016887; F:ATPase activity; IEA:InterPro.
DR   GO; GO:0044183; F:protein folding chaperone; IDA:FlyBase.
DR   GO; GO:0003723; F:RNA binding; HDA:UniProtKB.
DR   GO; GO:0051082; F:unfolded protein binding; IBA:GO_Central.
DR   GO; GO:0071987; F:WD40-repeat domain binding; IPI:BHF-UCL.
DR   GO; GO:1904851; P:positive regulation of establishment of protein localization to telomere; IMP:BHF-UCL.
DR   GO; GO:1904871; P:positive regulation of protein localization to Cajal body; HMP:BHF-UCL.
DR   GO; GO:1904874; P:positive regulation of telomerase RNA localization to Cajal body; HMP:BHF-UCL.
DR   GO; GO:0032212; P:positive regulation of telomere maintenance via telomerase; IMP:BHF-UCL.
DR   GO; GO:0006457; P:protein folding; IDA:FlyBase.
DR   GO; GO:0050821; P:protein stabilization; IMP:BHF-UCL.
DR   CDD; cd03342; TCP1_zeta; 1.
DR   Gene3D; 1.10.560.10; -; 1.
DR   Gene3D; 3.30.260.10; -; 1.
DR   Gene3D; 3.50.7.10; -; 1.
DR   InterPro; IPR012722; Chap_CCT_zeta.
DR   InterPro; IPR017998; Chaperone_TCP-1.
DR   InterPro; IPR002194; Chaperonin_TCP-1_CS.
DR   InterPro; IPR002423; Cpn60/TCP-1.
DR   InterPro; IPR027409; GroEL-like_apical_dom_sf.
DR   InterPro; IPR027413; GROEL-like_equatorial_sf.
DR   InterPro; IPR027410; TCP-1-like_intermed_sf.
DR   Pfam; PF00118; Cpn60_TCP1; 1.
DR   PRINTS; PR00304; TCOMPLEXTCP1.
DR   SUPFAM; SSF48592; SSF48592; 1.
DR   SUPFAM; SSF52029; SSF52029; 1.
DR   SUPFAM; SSF54849; SSF54849; 1.
DR   TIGRFAMs; TIGR02347; chap_CCT_zeta; 1.
DR   PROSITE; PS00750; TCP1_1; 1.
DR   PROSITE; PS00751; TCP1_2; 1.
DR   PROSITE; PS00995; TCP1_3; 1.
PE   1: Evidence at protein level;
KW   3D-structure; Acetylation; Alternative splicing; ATP-binding; Chaperone;
KW   Cytoplasm; Direct protein sequencing; Isopeptide bond; Nucleotide-binding;
KW   Phosphoprotein; Reference proteome; Ubl conjugation.
FT   INIT_MET        1
FT                   /note="Removed"
FT                   /evidence="ECO:0000269|PubMed:12665801,
FT                   ECO:0007744|PubMed:22814378"
FT   CHAIN           2..531
FT                   /note="T-complex protein 1 subunit zeta"
FT                   /id="PRO_0000128355"
FT   MOD_RES         2
FT                   /note="N-acetylalanine"
FT                   /evidence="ECO:0007744|PubMed:22814378"
FT   MOD_RES         5
FT                   /note="N6-acetyllysine"
FT                   /evidence="ECO:0000250|UniProtKB:P80317"
FT   MOD_RES         199
FT                   /note="N6-acetyllysine"
FT                   /evidence="ECO:0007744|PubMed:19608861"
FT   MOD_RES         205
FT                   /note="Phosphoserine"
FT                   /evidence="ECO:0007744|PubMed:23186163"
FT   MOD_RES         287
FT                   /note="N6-acetyllysine"
FT                   /evidence="ECO:0000250|UniProtKB:P80317"
FT   MOD_RES         365
FT                   /note="N6-acetyllysine"
FT                   /evidence="ECO:0007744|PubMed:19608861"
FT   MOD_RES         377
FT                   /note="N6-acetyllysine"
FT                   /evidence="ECO:0007744|PubMed:19608861"
FT   MOD_RES         388
FT                   /note="N6-acetyllysine"
FT                   /evidence="ECO:0007744|PubMed:19608861"
FT   CROSSLNK        251
FT                   /note="Glycyl lysine isopeptide (Lys-Gly) (interchain with
FT                   G-Cter in SUMO2)"
FT                   /evidence="ECO:0007744|PubMed:28112733"
FT   VAR_SEQ         68..112
FT                   /note="Missing (in isoform 2)"
FT                   /evidence="ECO:0000303|PubMed:15489334"
FT                   /id="VSP_044918"
FT   VARIANT         229
FT                   /note="Y -> C (in dbSNP:rs33922584)"
FT                   /id="VAR_052268"
FT   CONFLICT        301..303
FT                   /note="SLD -> PLS (in Ref. 3; BAB61032)"
FT                   /evidence="ECO:0000305"
SQ   SEQUENCE   531 AA;  58024 MW;  43ABCF548CC82B81 CRC64;
     MAAVKTLNPK AEVARAQAAL AVNISAARGL QDVLRTNLGP KGTMKMLVSG AGDIKLTKDG
     NVLLHEMQIQ HPTASLIAKV ATAQDDITGD GTTSNVLIIG ELLKQADLYI SEGLHPRIIT
     EGFEAAKEKA LQFLEEVKVS REMDRETLID VARTSLRTKV HAELADVLTE AVVDSILAIK
     KQDEPIDLFM IEIMEMKHKS ETDTSLIRGL VLDHGARHPD MKKRVEDAYI LTCNVSLEYE
     KTEVNSGFFY KSAEEREKLV KAERKFIEDR VKKIIELKRK VCGDSDKGFV VINQKGIDPF
     SLDALSKEGI VALRRAKRRN MERLTLACGG VALNSFDDLS PDCLGHAGLV YEYTLGEEKF
     TFIEKCNNPR SVTLLIKGPN KHTLTQIKDA VRDGLRAVKN AIDDGCVVPG AGAVEVAMAE
     ALIKHKPSVK GRAQLGVQAF ADALLIIPKV LAQNSGFDLQ ETLVKIQAEH SESGQLVGVD
     LNTGEPMVAA EVGVWDNYCV KKQLLHSCTV IATNILLVDE IMRAGMSSLK G
```

|  |
| --- |
| **Mascot:** http://www.matrixscience.com/ |
