# Supplementary material for: Chlorpromazine induces cytotoxic autophagy in glioblastoma cells via endoplasmic reticulum stress and unfolded protein response
Source: J Exp Clin Cancer Res. 2021 Nov 5;40:347. doi: 10.1186/s13046-021-02144-w (PMC8569984; doi:10.1186/s13046-021-02144-w)
Supplement: Supplementary file 1 — Additional file 1. [file 13046_2021_2144_MOESM1_ESM.zip › MASCOT IDs/Mascot Search Results_ TERA_HUMAN.html]

Mascot Search Results: TERA\_HUMAN
 

# MASCOT Search Results

## Protein View: TERA\_HUMAN

### Transitional endoplasmic reticulum ATPase OS=Homo sapiens OX=9606 GN=VCP PE=1 SV=4

|  |  |
| --- | --- |
| Database: | SwissProt |
| Score: | 225 |
| Monoisotopic mass (Mr): | 89950 |
| Calculated pI: | 5.14 |
| Taxonomy: | Homo sapiens |

Sequence similarity is available as an NCBI BLAST search of TERA\_HUMAN against nr.

### Search parameters

|  |  |
| --- | --- |
| MS data file: | `ppw_D2_152941901400.txt` |
| Enzyme: | Trypsin: cuts C-term side of KR unless next residue is P. |
| Fixed modifications: | Carbamidomethyl (C) |

### Protein sequence coverage: 6%

Matched peptides shown in ***bold red***.

|  |  |  |  |  |  |
| --- | --- | --- | --- | --- | --- |
| `1` | `MASGADSKGD` | `DLSTAILKQK` | `NRPNRLIVDE` | `AINEDNSVVS` | `LSQPKMDELQ` |
| `51` | `LFRGDTVLLK` | `GKKRREAVCI` | `VLSDDTCSDE` | `KIRMNRVVRN` | `NLRVRLGDVI` |
| `101` | `SIQPCPDVKY` | `GKRIHVLPID` | `DTVEGITGNL` | `FEVYLKPYFL` | `EAYRPIRKGD` |
| `151` | `IFLVRGGMRA` | `VEFKVVETDP` | `SPYCIVAPDT` | `VIHCEGEPIK` | `REDEEESLNE` |
| `201` | `VGYDDIGGCR` | `KQLAQIKEMV` | `ELPLRHPALF` | `KAIGVKPPRG` | `ILLYGPPGTG` |
| `251` | `KTLIARAVAN` | `ETGAFFFLIN` | `GPEIMSKLAG` | `ESESNLRKAF` | `EEAEKNAPAI` |
| `301` | `IFIDELDAIA` | `PKREKTHGEV` | `ERRIVSQLLT` | `LMDGLKQRAH` | `VIVMAATNRP` |
| `351` | `NSIDPALRRF` | `GRFDREVDIG` | `IPDATGRLEI` | `LQIHTKNMKL` | `ADDVDLEQVA` |
| `401` | `NETHGHVGAD` | `LAALCSEAAL` | `QAIRKKMDLI` | `DLEDETIDAE` | `VMNSLAVTMD` |
| `451` | `DFRWALSQSN` | `PSALRETVVE` | `VPQVTWEDIG` | `GLEDVKRELQ` | `ELVQYPVEHP` |
| `501` | `DKFLKFGMTP` | `SKGVLFYGPP` | `GCGKTLLAKA` | `IANECQANFI` | `SIKGPELLTM` |
| `551` | `WFGESEANVR` | `EIFDKARQAA` | `PCVLFFDELD` | `SIAKARGGNI` | `GDGGGAADRV` |
| `601` | `INQILTEMDG` | `MSTKKNVFII` | `GATNRPDIID` | `PAILRPGRLD` | `QLIYIPLPDE` |
| `651` | `KSRVAILKAN` | `LRKSPVAKDV` | `DLEFLAKMTN` | `GFSGADLTEI` | `CQRACKLAIR` |
| `701` | `ESIESEIRRE` | `RERQTNPSAM` | `EVEEDDPVPE` | `IRRDHFEEAM` | `RFARRSVSDN` |
| `751` | `DIRKYEMFAQ` | `TLQQSRGFGS` | `FRFPSGNQGG` | `AGPSQGSGGG` | `TGGSVYTEDN` |
| `801` | `DDDLYG` |  |  |  |  |

Unformatted sequence string: 806 residues (for pasting into other applications).

|  |  |  |  |
| --- | --- | --- | --- |
| Sort by | residue number | increasing mass | decreasing mass |
| Show | matched peptides only | predicted peptides also |  |

| Query | Start | – | End | Observed | Mr(expt) | Mr(calc) | ppm | M | Score | Expect | Rank | U | Peptide |
| --- | --- | --- | --- | --- | --- | --- | --- | --- | --- | --- | --- | --- | --- |
| 57 | 678 | – | 693 | 1799.8757 | 1798.8684 | 1798.7978 | 39.3 | 0 | 83 | 1.5e-07 | 1Score **> 27** indicates **identity** | U | K.MTNGFSGADLTEICQR.A |
| 105 | 767 | – | 806 | 3871.6934 | 3870.6861 | 3870.6267 | 15.4 | 1 | 166 | 1.5e-16 | 1Score **> 20** indicates **identity** | U | R.GFGSFRFPSGNQGGAGPSQGSGGGTGGSVYTEDNDDDLYG.- |

---

```
ID   TERA_HUMAN              Reviewed;         806 AA.
AC   P55072; B2R5T8; Q0V924; Q2TAI5; Q969G7; Q9UCD5;
DT   01-OCT-1996, integrated into UniProtKB/Swiss-Prot.
DT   23-JAN-2007, sequence version 4.
DT   02-JUN-2021, entry version 227.
DE   RecName: Full=Transitional endoplasmic reticulum ATPase;
DE            Short=TER ATPase;
DE            EC=3.6.4.6 {ECO:0000269|PubMed:26471729};
DE   AltName: Full=15S Mg(2+)-ATPase p97 subunit;
DE   AltName: Full=Valosin-containing protein;
DE            Short=VCP;
GN   Name=VCP;
OS   Homo sapiens (Human).
OC   Eukaryota; Metazoa; Chordata; Craniata; Vertebrata; Euteleostomi; Mammalia;
OC   Eutheria; Euarchontoglires; Primates; Haplorrhini; Catarrhini; Hominidae;
OC   Homo.
OX   NCBI_TaxID=9606;
RN   [1]
RP   NUCLEOTIDE SEQUENCE [GENOMIC DNA].
RA   Lamerdin J.E., McCready P.M., Skowronski E., Adamson A.W.,
RA   Burkhart-Schultz K., Gordon L., Kyle A., Ramirez M., Stilwagen S., Phan H.,
RA   Velasco N., Garnes J., Danganan L., Poundstone P., Christensen M.,
RA   Georgescu A., Avila J., Liu S., Attix C., Andreise T., Trankheim M.,
RA   Amico-Keller G., Coefield J., Duarte S., Lucas S., Bruce R., Thomas P.,
RA   Quan G., Kronmiller B., Arellano A., Montgomery M., Ow D., Nolan M.,
RA   Trong S., Kobayashi A., Olsen A.O., Carrano A.V.;
RT   "Sequence analysis of a human P1 clone containing the XRCC9 DNA repair
RT   gene.";
RL   Submitted (MAR-1998) to the EMBL/GenBank/DDBJ databases.
RN   [2]
RP   NUCLEOTIDE SEQUENCE [LARGE SCALE MRNA].
RC   TISSUE=Pituitary;
RX   PubMed=10931946; DOI=10.1073/pnas.160270997;
RA   Hu R.-M., Han Z.-G., Song H.-D., Peng Y.-D., Huang Q.-H., Ren S.-X.,
RA   Gu Y.-J., Huang C.-H., Li Y.-B., Jiang C.-L., Fu G., Zhang Q.-H., Gu B.-W.,
RA   Dai M., Mao Y.-F., Gao G.-F., Rong R., Ye M., Zhou J., Xu S.-H., Gu J.,
RA   Shi J.-X., Jin W.-R., Zhang C.-K., Wu T.-M., Huang G.-Y., Chen Z.,
RA   Chen M.-D., Chen J.-L.;
RT   "Gene expression profiling in the human hypothalamus-pituitary-adrenal axis
RT   and full-length cDNA cloning.";
RL   Proc. Natl. Acad. Sci. U.S.A. 97:9543-9548(2000).
RN   [3]
RP   NUCLEOTIDE SEQUENCE [LARGE SCALE MRNA].
RC   TISSUE=Cerebellum;
RX   PubMed=14702039; DOI=10.1038/ng1285;
RA   Ota T., Suzuki Y., Nishikawa T., Otsuki T., Sugiyama T., Irie R.,
RA   Wakamatsu A., Hayashi K., Sato H., Nagai K., Kimura K., Makita H.,
RA   Sekine M., Obayashi M., Nishi T., Shibahara T., Tanaka T., Ishii S.,
RA   Yamamoto J., Saito K., Kawai Y., Isono Y., Nakamura Y., Nagahari K.,
RA   Murakami K., Yasuda T., Iwayanagi T., Wagatsuma M., Shiratori A., Sudo H.,
RA   Hosoiri T., Kaku Y., Kodaira H., Kondo H., Sugawara M., Takahashi M.,
RA   Kanda K., Yokoi T., Furuya T., Kikkawa E., Omura Y., Abe K., Kamihara K.,
RA   Katsuta N., Sato K., Tanikawa M., Yamazaki M., Ninomiya K., Ishibashi T.,
RA   Yamashita H., Murakawa K., Fujimori K., Tanai H., Kimata M., Watanabe M.,
RA   Hiraoka S., Chiba Y., Ishida S., Ono Y., Takiguchi S., Watanabe S.,
RA   Yosida M., Hotuta T., Kusano J., Kanehori K., Takahashi-Fujii A., Hara H.,
RA   Tanase T.-O., Nomura Y., Togiya S., Komai F., Hara R., Takeuchi K.,
RA   Arita M., Imose N., Musashino K., Yuuki H., Oshima A., Sasaki N.,
RA   Aotsuka S., Yoshikawa Y., Matsunawa H., Ichihara T., Shiohata N., Sano S.,
RA   Moriya S., Momiyama H., Satoh N., Takami S., Terashima Y., Suzuki O.,
RA   Nakagawa S., Senoh A., Mizoguchi H., Goto Y., Shimizu F., Wakebe H.,
RA   Hishigaki H., Watanabe T., Sugiyama A., Takemoto M., Kawakami B.,
RA   Yamazaki M., Watanabe K., Kumagai A., Itakura S., Fukuzumi Y., Fujimori Y.,
RA   Komiyama M., Tashiro H., Tanigami A., Fujiwara T., Ono T., Yamada K.,
RA   Fujii Y., Ozaki K., Hirao M., Ohmori Y., Kawabata A., Hikiji T.,
RA   Kobatake N., Inagaki H., Ikema Y., Okamoto S., Okitani R., Kawakami T.,
RA   Noguchi S., Itoh T., Shigeta K., Senba T., Matsumura K., Nakajima Y.,
RA   Mizuno T., Morinaga M., Sasaki M., Togashi T., Oyama M., Hata H.,
RA   Watanabe M., Komatsu T., Mizushima-Sugano J., Satoh T., Shirai Y.,
RA   Takahashi Y., Nakagawa K., Okumura K., Nagase T., Nomura N., Kikuchi H.,
RA   Masuho Y., Yamashita R., Nakai K., Yada T., Nakamura Y., Ohara O.,
RA   Isogai T., Sugano S.;
RT   "Complete sequencing and characterization of 21,243 full-length human
RT   cDNAs.";
RL   Nat. Genet. 36:40-45(2004).
RN   [4]
RP   NUCLEOTIDE SEQUENCE [LARGE SCALE GENOMIC DNA].
RX   PubMed=15164053; DOI=10.1038/nature02465;
RA   Humphray S.J., Oliver K., Hunt A.R., Plumb R.W., Loveland J.E., Howe K.L.,
RA   Andrews T.D., Searle S., Hunt S.E., Scott C.E., Jones M.C., Ainscough R.,
RA   Almeida J.P., Ambrose K.D., Ashwell R.I.S., Babbage A.K., Babbage S.,
RA   Bagguley C.L., Bailey J., Banerjee R., Barker D.J., Barlow K.F., Bates K.,
RA   Beasley H., Beasley O., Bird C.P., Bray-Allen S., Brown A.J., Brown J.Y.,
RA   Burford D., Burrill W., Burton J., Carder C., Carter N.P., Chapman J.C.,
RA   Chen Y., Clarke G., Clark S.Y., Clee C.M., Clegg S., Collier R.E.,
RA   Corby N., Crosier M., Cummings A.T., Davies J., Dhami P., Dunn M.,
RA   Dutta I., Dyer L.W., Earthrowl M.E., Faulkner L., Fleming C.J.,
RA   Frankish A., Frankland J.A., French L., Fricker D.G., Garner P.,
RA   Garnett J., Ghori J., Gilbert J.G.R., Glison C., Grafham D.V., Gribble S.,
RA   Griffiths C., Griffiths-Jones S., Grocock R., Guy J., Hall R.E.,
RA   Hammond S., Harley J.L., Harrison E.S.I., Hart E.A., Heath P.D.,
RA   Henderson C.D., Hopkins B.L., Howard P.J., Howden P.J., Huckle E.,
RA   Johnson C., Johnson D., Joy A.A., Kay M., Keenan S., Kershaw J.K.,
RA   Kimberley A.M., King A., Knights A., Laird G.K., Langford C., Lawlor S.,
RA   Leongamornlert D.A., Leversha M., Lloyd C., Lloyd D.M., Lovell J.,
RA   Martin S., Mashreghi-Mohammadi M., Matthews L., McLaren S., McLay K.E.,
RA   McMurray A., Milne S., Nickerson T., Nisbett J., Nordsiek G., Pearce A.V.,
RA   Peck A.I., Porter K.M., Pandian R., Pelan S., Phillimore B., Povey S.,
RA   Ramsey Y., Rand V., Scharfe M., Sehra H.K., Shownkeen R., Sims S.K.,
RA   Skuce C.D., Smith M., Steward C.A., Swarbreck D., Sycamore N., Tester J.,
RA   Thorpe A., Tracey A., Tromans A., Thomas D.W., Wall M., Wallis J.M.,
RA   West A.P., Whitehead S.L., Willey D.L., Williams S.A., Wilming L.,
RA   Wray P.W., Young L., Ashurst J.L., Coulson A., Blocker H., Durbin R.M.,
RA   Sulston J.E., Hubbard T., Jackson M.J., Bentley D.R., Beck S., Rogers J.,
RA   Dunham I.;
RT   "DNA sequence and analysis of human chromosome 9.";
RL   Nature 429:369-374(2004).
RN   [5]
RP   NUCLEOTIDE SEQUENCE [LARGE SCALE GENOMIC DNA].
RA   Mural R.J., Istrail S., Sutton G.G., Florea L., Halpern A.L., Mobarry C.M.,
RA   Lippert R., Walenz B., Shatkay H., Dew I., Miller J.R., Flanigan M.J.,
RA   Edwards N.J., Bolanos R., Fasulo D., Halldorsson B.V., Hannenhalli S.,
RA   Turner R., Yooseph S., Lu F., Nusskern D.R., Shue B.C., Zheng X.H.,
RA   Zhong F., Delcher A.L., Huson D.H., Kravitz S.A., Mouchard L., Reinert K.,
RA   Remington K.A., Clark A.G., Waterman M.S., Eichler E.E., Adams M.D.,
RA   Hunkapiller M.W., Myers E.W., Venter J.C.;
RL   Submitted (SEP-2005) to the EMBL/GenBank/DDBJ databases.
RN   [6]
RP   NUCLEOTIDE SEQUENCE [LARGE SCALE MRNA].
RC   TISSUE=Uterus;
RX   PubMed=15489334; DOI=10.1101/gr.2596504;
RG   The MGC Project Team;
RT   "The status, quality, and expansion of the NIH full-length cDNA project:
RT   the Mammalian Gene Collection (MGC).";
RL   Genome Res. 14:2121-2127(2004).
RN   [7]
RP   PROTEIN SEQUENCE OF 2-25.
RC   TISSUE=Platelet;
RX   PubMed=12665801; DOI=10.1038/nbt810;
RA   Gevaert K., Goethals M., Martens L., Van Damme J., Staes A., Thomas G.R.,
RA   Vandekerckhove J.;
RT   "Exploring proteomes and analyzing protein processing by mass spectrometric
RT   identification of sorted N-terminal peptides.";
RL   Nat. Biotechnol. 21:566-569(2003).
RN   [8]
RP   PROTEIN SEQUENCE OF 2-18; 148-155; 278-287; 296-312; 366-377; 466-487;
RP   587-599; 639-651 AND 669-677, CLEAVAGE OF INITIATOR METHIONINE, ACETYLATION
RP   AT ALA-2, AND IDENTIFICATION BY MASS SPECTROMETRY.
RC   TISSUE=Platelet;
RA   Bienvenut W.V., Claeys D.;
RL   Submitted (NOV-2005) to UniProtKB.
RN   [9]
RP   PROTEIN SEQUENCE OF 27-41 AND 233-238, AND INTERACTION WITH CLATHRIN.
RC   TISSUE=Glial tumor;
RX   PubMed=8413590; DOI=10.1038/365459a0;
RA   Pleasure I.T., Black M.M., Keen J.H.;
RT   "Valosin-containing protein, VCP, is a ubiquitous clathrin-binding
RT   protein.";
RL   Nature 365:459-462(1993).
RN   [10]
RP   PROTEIN SEQUENCE OF 46-53; 66-81; 96-109; 148-155; 240-251; 323-336;
RP   454-502; 530-560; 600-614; 639-651; 678-693; 714-732 AND 754-766, AND
RP   IDENTIFICATION BY MASS SPECTROMETRY.
RC   TISSUE=Fetal brain cortex;
RA   Lubec G., Chen W.-Q., Sun Y.;
RL   Submitted (DEC-2008) to UniProtKB.
RN   [11]
RP   PROTEIN SEQUENCE OF 314-322, IDENTIFICATION BY MASS SPECTROMETRY,
RP   METHYLATION AT LYS-315, MUTAGENESIS OF LYS-315, CHARACTERIZATION OF
RP   VARIANTS IBMPFD1 HIS-155 AND GLN-191, AND CHARACTERIZATION OF VARIANT
RP   FTDALS6 GLY-159.
RX   PubMed=23349634; DOI=10.1371/journal.pgen.1003210;
RA   Cloutier P., Lavallee-Adam M., Faubert D., Blanchette M., Coulombe B.;
RT   "A newly uncovered group of distantly related lysine methyltransferases
RT   preferentially interact with molecular chaperones to regulate their
RT   activity.";
RL   PLoS Genet. 9:E1003210-E1003210(2013).
RN   [12]
RP   NUCLEOTIDE SEQUENCE [MRNA] OF 388-483.
RC   TISSUE=Fetal brain;
RA   Dmitrenko V.V., Garifulin O.M., Kavsan V.M.;
RT   "Characterization of different mRNA types expressed in human brain.";
RL   Submitted (APR-1996) to the EMBL/GenBank/DDBJ databases.
RN   [13]
RP   INTERACTION WITH NGLY1.
RX   PubMed=15362974; DOI=10.1042/bj20041498;
RA   McNeill H., Knebel A., Arthur J.S., Cuenda A., Cohen P.;
RT   "A novel UBA and UBX domain protein that binds polyubiquitin and VCP and is
RT   a substrate for SAPKs.";
RL   Biochem. J. 384:391-400(2004).
RN   [14]
RP   FUNCTION, INTERACTION WITH RNF19A, IDENTIFICATION BY MASS SPECTROMETRY,
RP   SUBCELLULAR LOCATION, AND MUTAGENESIS OF LYS-524.
RX   PubMed=15456787; DOI=10.1074/jbc.m406683200;
RA   Ishigaki S., Hishikawa N., Niwa J., Iemura S., Natsume T., Hori S.,
RA   Kakizuka A., Tanaka K., Sobue G.;
RT   "Physical and functional interaction between dorfin and valosin-containing
RT   protein that are colocalized in ubiquitylated inclusions in
RT   neurodegenerative disorders.";
RL   J. Biol. Chem. 279:51376-51385(2004).
RN   [15]
RP   INTERACTION WITH SELENOS, AND SUBCELLULAR LOCATION.
RX   PubMed=15215856; DOI=10.1038/nature02656;
RA   Ye Y., Shibata Y., Yun C., Ron D., Rapoport T.A.;
RT   "A membrane protein complex mediates retro-translocation from the ER lumen
RT   into the cytosol.";
RL   Nature 429:841-847(2004).
RN   [16]
RP   ISGYLATION.
RX   PubMed=16139798; DOI=10.1016/j.bbrc.2005.08.132;
RA   Giannakopoulos N.V., Luo J.K., Papov V., Zou W., Lenschow D.J.,
RA   Jacobs B.S., Borden E.C., Li J., Virgin H.W., Zhang D.E.;
RT   "Proteomic identification of proteins conjugated to ISG15 in mouse and
RT   human cells.";
RL   Biochem. Biophys. Res. Commun. 336:496-506(2005).
RN   [17]
RP   INTERACTION WITH SYVN1 AND DERL1.
RX   PubMed=16289116; DOI=10.1016/j.jmb.2005.10.020;
RA   Schulze A., Standera S., Buerger E., Kikkert M., van Voorden S., Wiertz E.,
RA   Koning F., Kloetzel P.-M., Seeger M.;
RT   "The ubiquitin-domain protein HERP forms a complex with components of the
RT   endoplasmic reticulum associated degradation pathway.";
RL   J. Mol. Biol. 354:1021-1027(2005).
RN   [18]
RP   INTERACTION WITH AMFR, FUNCTION, SUBCELLULAR LOCATION, AND MUTAGENESIS OF
RP   LYS-251 AND LYS-524.
RX   PubMed=16168377; DOI=10.1016/j.molcel.2005.08.009;
RA   Song B.L., Sever N., DeBose-Boyd R.A.;
RT   "Gp78, a membrane-anchored ubiquitin ligase, associates with Insig-1 and
RT   couples sterol-regulated ubiquitination to degradation of HMG CoA
RT   reductase.";
RL   Mol. Cell 19:829-840(2005).
RN   [19]
RP   FUNCTION, AND INTERACTION WITH DERL1; AMFR; SYVN1 AND SELENOS.
RX   PubMed=16186510; DOI=10.1073/pnas.0505006102;
RA   Ye Y., Shibata Y., Kikkert M., van Voorden S., Wiertz E., Rapoport T.A.;
RT   "Recruitment of the p97 ATPase and ubiquitin ligases to the site of
RT   retrotranslocation at the endoplasmic reticulum membrane.";
RL   Proc. Natl. Acad. Sci. U.S.A. 102:14132-14138(2005).
RN   [20]
RP   INTERACTION WITH DERL1 AND DERL2.
RX   PubMed=16186509; DOI=10.1073/pnas.0505014102;
RA   Lilley B.N., Ploegh H.L.;
RT   "Multiprotein complexes that link dislocation, ubiquitination, and
RT   extraction of misfolded proteins from the endoplasmic reticulum membrane.";
RL   Proc. Natl. Acad. Sci. U.S.A. 102:14296-14301(2005).
RN   [21]
RP   INTERACTION WITH CASR AND RNF19A.
RX   PubMed=16513638; DOI=10.1074/jbc.m513552200;
RA   Huang Y., Niwa J., Sobue G., Breitwieser G.E.;
RT   "Calcium-sensing receptor ubiquitination and degradation mediated by the E3
RT   ubiquitin ligase dorfin.";
RL   J. Biol. Chem. 281:11610-11617(2006).
RN   [22]
RP   INTERACTION WITH DERL1; DERL2 AND DERL3.
RX   PubMed=16449189; DOI=10.1083/jcb.200507057;
RA   Oda Y., Okada T., Yoshida H., Kaufman R.J., Nagata K., Mori K.;
RT   "Derlin-2 and Derlin-3 are regulated by the mammalian unfolded protein
RT   response and are required for ER-associated degradation.";
RL   J. Cell Biol. 172:383-393(2006).
RN   [23]
RP   INTERACTION WITH UBXN4.
RX   PubMed=16968747; DOI=10.1242/jcs.03163;
RA   Liang J., Yin C., Doong H., Fang S., Peterhoff C., Nixon R.A.,
RA   Monteiro M.J.;
RT   "Characterization of erasin (UBXD2): a new ER protein that promotes ER-
RT   associated protein degradation.";
RL   J. Cell Sci. 119:4011-4024(2006).
RN   [24]
RP   INTERACTION WITH TRIM13.
RX   PubMed=17314412; DOI=10.1091/mbc.e06-03-0248;
RA   Lerner M., Corcoran M., Cepeda D., Nielsen M.L., Zubarev R., Ponten F.,
RA   Uhlen M., Hober S., Grander D., Sangfelt O.;
RT   "The RBCC gene RFP2 (Leu5) encodes a novel transmembrane E3 ubiquitin
RT   ligase involved in ERAD.";
RL   Mol. Biol. Cell 18:1670-1682(2007).
RN   [25]
RP   IDENTIFICATION BY MASS SPECTROMETRY [LARGE SCALE ANALYSIS].
RC   TISSUE=Embryonic kidney;
RX   PubMed=17525332; DOI=10.1126/science.1140321;
RA   Matsuoka S., Ballif B.A., Smogorzewska A., McDonald E.R. III, Hurov K.E.,
RA   Luo J., Bakalarski C.E., Zhao Z., Solimini N., Lerenthal Y., Shiloh Y.,
RA   Gygi S.P., Elledge S.J.;
RT   "ATM and ATR substrate analysis reveals extensive protein networks
RT   responsive to DNA damage.";
RL   Science 316:1160-1166(2007).
RN   [26]
RP   INTERACTION WITH RNF103.
RX   PubMed=18675248; DOI=10.1016/j.bbrc.2008.07.126;
RA   Maruyama Y., Yamada M., Takahashi K., Yamada M.;
RT   "Ubiquitin ligase Kf-1 is involved in the endoplasmic reticulum-associated
RT   degradation pathway.";
RL   Biochem. Biophys. Res. Commun. 374:737-741(2008).
RN   [27]
RP   INTERACTION WITH UBXN6.
RX   PubMed=18656546; DOI=10.1016/j.biocel.2008.06.008;
RA   Madsen L., Andersen K.M., Prag S., Moos T., Semple C.A., Seeger M.,
RA   Hartmann-Petersen R.;
RT   "Ubxd1 is a novel co-factor of the human p97 ATPase.";
RL   Int. J. Biochem. Cell Biol. 40:2927-2942(2008).
RN   [28]
RP   PHOSPHORYLATION [LARGE SCALE ANALYSIS] AT SER-3; THR-436 AND SER-787, AND
RP   IDENTIFICATION BY MASS SPECTROMETRY [LARGE SCALE ANALYSIS].
RC   TISSUE=Cervix carcinoma;
RX   PubMed=18691976; DOI=10.1016/j.molcel.2008.07.007;
RA   Daub H., Olsen J.V., Bairlein M., Gnad F., Oppermann F.S., Korner R.,
RA   Greff Z., Keri G., Stemmann O., Mann M.;
RT   "Kinase-selective enrichment enables quantitative phosphoproteomics of the
RT   kinome across the cell cycle.";
RL   Mol. Cell 31:438-448(2008).
RN   [29]
RP   INTERACTION WITH TRIM21.
RX   PubMed=18022694; DOI=10.1016/j.molimm.2007.10.023;
RA   Takahata M., Bohgaki M., Tsukiyama T., Kondo T., Asaka M., Hatakeyama S.;
RT   "Ro52 functionally interacts with IgG1 and regulates its quality control
RT   via the ERAD system.";
RL   Mol. Immunol. 45:2045-2054(2008).
RN   [30]
RP   ACETYLATION [LARGE SCALE ANALYSIS] AT ALA-2, CLEAVAGE OF INITIATOR
RP   METHIONINE [LARGE SCALE ANALYSIS], AND IDENTIFICATION BY MASS SPECTROMETRY
RP   [LARGE SCALE ANALYSIS].
RX   PubMed=19413330; DOI=10.1021/ac9004309;
RA   Gauci S., Helbig A.O., Slijper M., Krijgsveld J., Heck A.J., Mohammed S.;
RT   "Lys-N and trypsin cover complementary parts of the phosphoproteome in a
RT   refined SCX-based approach.";
RL   Anal. Chem. 81:4493-4501(2009).
RN   [31]
RP   INTERACTION WITH UBXN6.
RX   PubMed=19174149; DOI=10.1016/j.bbrc.2009.01.076;
RA   Kern M., Fernandez-Saiz V., Schaefer Z., Buchberger A.;
RT   "UBXD1 binds p97 through two independent binding sites.";
RL   Biochem. Biophys. Res. Commun. 380:303-307(2009).
RN   [32]
RP   INTERACTION WITH UBXN6.
RX   PubMed=19275885; DOI=10.1016/j.bbrc.2009.03.012;
RA   Nagahama M., Ohnishi M., Kawate Y., Matsui T., Miyake H., Yuasa K.,
RA   Tani K., Tagaya M., Tsuji A.;
RT   "UBXD1 is a VCP-interacting protein that is involved in ER-associated
RT   degradation.";
RL   Biochem. Biophys. Res. Commun. 382:303-308(2009).
RN   [33]
RP   INTERACTION WITH UBXN4, AND IDENTIFICATION IN A COMPLEX WITH UBQLN1 AND
RP   UBXN4.
RX   PubMed=19822669; DOI=10.1083/jcb.200903024;
RA   Lim P.J., Danner R., Liang J., Doong H., Harman C., Srinivasan D.,
RA   Rothenberg C., Wang H., Ye Y., Fang S., Monteiro M.J.;
RT   "Ubiquilin and p97/VCP bind erasin, forming a complex involved in ERAD.";
RL   J. Cell Biol. 187:201-217(2009).
RN   [34]
RP   INTERACTION WITH YOD1.
RX   PubMed=19818707; DOI=10.1016/j.molcel.2009.09.016;
RA   Ernst R., Mueller B., Ploegh H.L., Schlieker C.;
RT   "The otubain YOD1 is a deubiquitinating enzyme that associates with p97 to
RT   facilitate protein dislocation from the ER.";
RL   Mol. Cell 36:28-38(2009).
RN   [35]
RP   ACETYLATION [LARGE SCALE ANALYSIS] AT ALA-2, PHOSPHORYLATION [LARGE SCALE
RP   ANALYSIS] AT SER-3 AND SER-37, CLEAVAGE OF INITIATOR METHIONINE [LARGE
RP   SCALE ANALYSIS], AND IDENTIFICATION BY MASS SPECTROMETRY [LARGE SCALE
RP   ANALYSIS].
RX   PubMed=19369195; DOI=10.1074/mcp.m800588-mcp200;
RA   Oppermann F.S., Gnad F., Olsen J.V., Hornberger R., Greff Z., Keri G.,
RA   Mann M., Daub H.;
RT   "Large-scale proteomics analysis of the human kinome.";
RL   Mol. Cell. Proteomics 8:1751-1764(2009).
RN   [36]
RP   PHOSPHORYLATION [LARGE SCALE ANALYSIS] AT SER-770 AND SER-775, AND
RP   IDENTIFICATION BY MASS SPECTROMETRY [LARGE SCALE ANALYSIS].
RC   TISSUE=Leukemic T-cell;
RX   PubMed=19690332; DOI=10.1126/scisignal.2000007;
RA   Mayya V., Lundgren D.H., Hwang S.-I., Rezaul K., Wu L., Eng J.K.,
RA   Rodionov V., Han D.K.;
RT   "Quantitative phosphoproteomic analysis of T cell receptor signaling
RT   reveals system-wide modulation of protein-protein interactions.";
RL   Sci. Signal. 2:RA46-RA46(2009).
RN   [37]
RP   INTERACTION WITH WASHC5.
RX   PubMed=20833645; DOI=10.1093/brain/awq222;
RA   Clemen C.S., Tangavelou K., Strucksberg K.H., Just S., Gaertner L.,
RA   Regus-Leidig H., Stumpf M., Reimann J., Coras R., Morgan R.O.,
RA   Fernandez M.P., Hofmann A., Muller S., Schoser B., Hanisch F.G.,
RA   Rottbauer W., Blumcke I., von Horsten S., Eichinger L., Schroder R.;
RT   "Strumpellin is a novel valosin-containing protein binding partner linking
RT   hereditary spastic paraplegia to protein aggregation diseases.";
RL   Brain 133:2920-2941(2010).
RN   [38]
RP   IDENTIFICATION BY MASS SPECTROMETRY [LARGE SCALE ANALYSIS].
RC   TISSUE=Cervix carcinoma;
RX   PubMed=20068231; DOI=10.1126/scisignal.2000475;
RA   Olsen J.V., Vermeulen M., Santamaria A., Kumar C., Miller M.L.,
RA   Jensen L.J., Gnad F., Cox J., Jensen T.S., Nigg E.A., Brunak S., Mann M.;
RT   "Quantitative phosphoproteomics reveals widespread full phosphorylation
RT   site occupancy during mitosis.";
RL   Sci. Signal. 3:RA3-RA3(2010).
RN   [39]
RP   IDENTIFICATION BY MASS SPECTROMETRY [LARGE SCALE ANALYSIS].
RX   PubMed=21269460; DOI=10.1186/1752-0509-5-17;
RA   Burkard T.R., Planyavsky M., Kaupe I., Breitwieser F.P., Buerckstuemmer T.,
RA   Bennett K.L., Superti-Furga G., Colinge J.;
RT   "Initial characterization of the human central proteome.";
RL   BMC Syst. Biol. 5:17-17(2011).
RN   [40]
RP   FUNCTION IN OMM PROTEIN TURNOVER.
RX   PubMed=21118995; DOI=10.1091/mbc.e10-09-0748;
RA   Xu S., Peng G., Wang Y., Fang S., Karbowski M.;
RT   "The AAA-ATPase p97 is essential for outer mitochondrial membrane protein
RT   turnover.";
RL   Mol. Biol. Cell 22:291-300(2011).
RN   [41]
RP   INTERACTION WITH BAG6.
RX   PubMed=21636303; DOI=10.1016/j.molcel.2011.05.010;
RA   Wang Q., Liu Y., Soetandyo N., Baek K., Hegde R., Ye Y.;
RT   "A ubiquitin ligase-associated chaperone holdase maintains polypeptides in
RT   soluble states for proteasome degradation.";
RL   Mol. Cell 42:758-770(2011).
RN   [42]
RP   FUNCTION, INTERACTION WITH CAV1 AND UBXN6, CHARACTERIZATION OF VARIANTS
RP   IBMPFD1 GLY-95; HIS-155 AND GLU-232, AND MUTAGENESIS OF GLU-578.
RX   PubMed=21822278; DOI=10.1038/ncb2301;
RA   Ritz D., Vuk M., Kirchner P., Bug M., Schuetz S., Hayer A., Bremer S.,
RA   Lusk C., Baloh R.H., Lee H., Glatter T., Gstaiger M., Aebersold R.,
RA   Weihl C.C., Meyer H.;
RT   "Endolysosomal sorting of ubiquitylated caveolin-1 is regulated by VCP and
RT   UBXD1 and impaired by VCP disease mutations.";
RL   Nat. Cell Biol. 13:1116-1123(2011).
RN   [43]
RP   FUNCTION.
RX   PubMed=22020440; DOI=10.1038/ncb2367;
RA   Meerang M., Ritz D., Paliwal S., Garajova Z., Bosshard M., Mailand N.,
RA   Janscak P., Hubscher U., Meyer H., Ramadan K.;
RT   "The ubiquitin-selective segregase VCP/p97 orchestrates the response to DNA
RT   double-strand breaks.";
RL   Nat. Cell Biol. 13:1376-1382(2011).
RN   [44]
RP   FUNCTION, INTERACTION WITH L3MBTL1, AND SUBCELLULAR LOCATION.
RX   PubMed=22120668; DOI=10.1038/nsmb.2188;
RA   Acs K., Luijsterburg M.S., Ackermann L., Salomons F.A., Hoppe T.,
RA   Dantuma N.P.;
RT   "The AAA-ATPase VCP/p97 promotes 53BP1 recruitment by removing L3MBTL1 from
RT   DNA double-strand breaks.";
RL   Nat. Struct. Mol. Biol. 18:1345-1350(2011).
RN   [45]
RP   INTERACTION WITH UBXN8.
RX   PubMed=21949850; DOI=10.1371/journal.pone.0025061;
RA   Madsen L., Kriegenburg F., Vala A., Best D., Prag S., Hofmann K.,
RA   Seeger M., Adams I.R., Hartmann-Petersen R.;
RT   "The tissue-specific Rep8/UBXD6 tethers p97 to the endoplasmic reticulum
RT   membrane for degradation of misfolded proteins.";
RL   PLoS ONE 6:E25061-E25061(2011).
RN   [46]
RP   ACETYLATION [LARGE SCALE ANALYSIS] AT ALA-2, PHOSPHORYLATION [LARGE SCALE
RP   ANALYSIS] AT SER-3, CLEAVAGE OF INITIATOR METHIONINE [LARGE SCALE
RP   ANALYSIS], AND IDENTIFICATION BY MASS SPECTROMETRY [LARGE SCALE ANALYSIS].
RX   PubMed=21406692; DOI=10.1126/scisignal.2001570;
RA   Rigbolt K.T., Prokhorova T.A., Akimov V., Henningsen J., Johansen P.T.,
RA   Kratchmarova I., Kassem M., Mann M., Olsen J.V., Blagoev B.;
RT   "System-wide temporal characterization of the proteome and phosphoproteome
RT   of human embryonic stem cell differentiation.";
RL   Sci. Signal. 4:RS3-RS3(2011).
RN   [47]
RP   INTERACTION WITH UBXN7.
RX   PubMed=22537386; DOI=10.1186/1741-7007-10-36;
RA   Bandau S., Knebel A., Gage Z.O., Wood N.T., Alexandru G.;
RT   "UBXN7 docks on neddylated cullin complexes using its UIM motif and causes
RT   HIF1alpha accumulation.";
RL   BMC Biol. 10:36-36(2012).
RN   [48]
RP   INTERACTION WITH RHBDD1, MUTAGENESIS OF LYS-251; LYS-524 AND GLU-578, AND
RP   IDENTIFICATION BY MASS SPECTROMETRY.
RX   PubMed=22795130; DOI=10.1016/j.molcel.2012.06.008;
RA   Fleig L., Bergbold N., Sahasrabudhe P., Geiger B., Kaltak L., Lemberg M.K.;
RT   "Ubiquitin-dependent intramembrane rhomboid protease promotes ERAD of
RT   membrane proteins.";
RL   Mol. Cell 47:558-569(2012).
RN   [49]
RP   INTERACTION WITH SPRTN.
RX   PubMed=22902628; DOI=10.1074/jbc.m112.400135;
RA   Ghosal G., Leung J.W., Nair B.C., Fong K.W., Chen J.;
RT   "Proliferating cell nuclear antigen (PCNA)-binding protein C1orf124 is a
RT   regulator of translesion synthesis.";
RL   J. Biol. Chem. 287:34225-34233(2012).
RN   [50]
RP   FUNCTION IN ERAD PATHWAY.
RX   PubMed=22607976; DOI=10.1016/j.molcel.2012.04.015;
RA   Sato T., Sako Y., Sho M., Momohara M., Suico M.A., Shuto T., Nishitoh H.,
RA   Okiyoneda T., Kokame K., Kaneko M., Taura M., Miyata M., Chosa K., Koga T.,
RA   Morino-Koga S., Wada I., Kai H.;
RT   "STT3B-dependent posttranslational N-glycosylation as a surveillance system
RT   for secretory protein.";
RL   Mol. Cell 47:99-110(2012).
RN   [51]
RP   ACETYLATION [LARGE SCALE ANALYSIS] AT ALA-2, CLEAVAGE OF INITIATOR
RP   METHIONINE [LARGE SCALE ANALYSIS], AND IDENTIFICATION BY MASS SPECTROMETRY
RP   [LARGE SCALE ANALYSIS].
RX   PubMed=22223895; DOI=10.1074/mcp.m111.015131;
RA   Bienvenut W.V., Sumpton D., Martinez A., Lilla S., Espagne C., Meinnel T.,
RA   Giglione C.;
RT   "Comparative large-scale characterisation of plant vs. mammal proteins
RT   reveals similar and idiosyncratic N-alpha acetylation features.";
RL   Mol. Cell. Proteomics 11:M111.015131-M111.015131(2012).
RN   [52]
RP   METHYLATION AT LYS-315, AND MUTAGENESIS OF LYS-312; ARG-313; GLU-314;
RP   LYS-315; THR-316; HIS-317 AND GLY-318.
RX   PubMed=22948820; DOI=10.1038/ncomms2041;
RA   Kernstock S., Davydova E., Jakobsson M., Moen A., Pettersen S.,
RA   Maelandsmo G.M., Egge-Jacobsen W., Falnes P.O.;
RT   "Lysine methylation of VCP by a member of a novel human protein
RT   methyltransferase family.";
RL   Nat. Commun. 3:1038-1038(2012).
RN   [53]
RP   FUNCTION, AND INTERACTION WITH SPRTN.
RX   PubMed=23042607; DOI=10.1038/nsmb.2394;
RA   Davis E.J., Lachaud C., Appleton P., Macartney T.J., Nathke I., Rouse J.;
RT   "DVC1 (C1orf124) recruits the p97 protein segregase to sites of DNA
RT   damage.";
RL   Nat. Struct. Mol. Biol. 19:1093-1100(2012).
RN   [54]
RP   FUNCTION, SUBCELLULAR LOCATION, AND INTERACTION WITH SPRTN.
RX   PubMed=23042605; DOI=10.1038/nsmb.2395;
RA   Mosbech A., Gibbs-Seymour I., Kagias K., Thorslund T., Beli P., Povlsen L.,
RA   Nielsen S.V., Smedegaard S., Sedgwick G., Lukas C., Hartmann-Petersen R.,
RA   Lukas J., Choudhary C., Pocock R., Bekker-Jensen S., Mailand N.;
RT   "DVC1 (C1orf124) is a DNA damage-targeting p97 adaptor that promotes
RT   ubiquitin-dependent responses to replication blocks.";
RL   Nat. Struct. Mol. Biol. 19:1084-1092(2012).
RN   [55]
RP   FUNCTION.
RX   PubMed=23335559; DOI=10.1074/jbc.m112.429076;
RA   Kirchner P., Bug M., Meyer H.;
RT   "Ubiquitination of the N-terminal region of caveolin-1 regulates endosomal
RT   sorting by the VCP/p97 AAA-ATPase.";
RL   J. Biol. Chem. 288:7363-7372(2013).
RN   [56]
RP   PHOSPHORYLATION [LARGE SCALE ANALYSIS] AT SER-3; SER-7; SER-13; SER-462 AND
RP   SER-702, AND IDENTIFICATION BY MASS SPECTROMETRY [LARGE SCALE ANALYSIS].
RC   TISSUE=Cervix carcinoma, and Erythroleukemia;
RX   PubMed=23186163; DOI=10.1021/pr300630k;
RA   Zhou H., Di Palma S., Preisinger C., Peng M., Polat A.N., Heck A.J.,
RA   Mohammed S.;
RT   "Toward a comprehensive characterization of a human cancer cell
RT   phosphoproteome.";
RL   J. Proteome Res. 12:260-271(2013).
RN   [57]
RP   INTERACTION WITH ZFAND2B.
RX   PubMed=24160817; DOI=10.1042/bj20130710;
RA   Glinka T., Alter J., Braunstein I., Tzach L., Wei Sheng C., Geifman S.,
RA   Edelmann M.J., Kessler B.M., Stanhill A.;
RT   "Signal-peptide-mediated translocation is regulated by a p97-AIRAPL
RT   complex.";
RL   Biochem. J. 457:253-261(2014).
RN   [58]
RP   IDENTIFICATION BY MASS SPECTROMETRY [LARGE SCALE ANALYSIS].
RC   TISSUE=Liver;
RX   PubMed=24275569; DOI=10.1016/j.jprot.2013.11.014;
RA   Bian Y., Song C., Cheng K., Dong M., Wang F., Huang J., Sun D., Wang L.,
RA   Ye M., Zou H.;
RT   "An enzyme assisted RP-RPLC approach for in-depth analysis of human liver
RT   phosphoproteome.";
RL   J. Proteomics 96:253-262(2014).
RN   [59]
RP   INTERACTION WITH RNF31.
RX   PubMed=24726327; DOI=10.1016/j.molcel.2014.03.016;
RA   Schaeffer V., Akutsu M., Olma M.H., Gomes L.C., Kawasaki M., Dikic I.;
RT   "Binding of OTULIN to the PUB domain of HOIP controls NF-kappaB
RT   signaling.";
RL   Mol. Cell 54:349-361(2014).
RN   [60]
RP   FUNCTION.
RX   PubMed=26565908; DOI=10.1016/j.celrep.2015.09.047;
RA   Kadowaki H., Nagai A., Maruyama T., Takami Y., Satrimafitrah P., Kato H.,
RA   Honda A., Hatta T., Natsume T., Sato T., Kai H., Ichijo H., Nishitoh H.;
RT   "Pre-emptive quality control protects the ER from protein overload via the
RT   proximity of ERAD components and SRP.";
RL   Cell Rep. 13:944-956(2015).
RN   [61]
RP   FUNCTION, CATALYTIC ACTIVITY, INTERACTION WITH DDX58 AND RNF125, AND
RP   MUTAGENESIS OF 52-PHE--ASP-55; TYR-110; GLU-305 AND GLU-578.
RX   PubMed=26471729; DOI=10.15252/embj.201591888;
RA   Hao Q., Jiao S., Shi Z., Li C., Meng X., Zhang Z., Wang Y., Song X.,
RA   Wang W., Zhang R., Zhao Y., Wong C.C., Zhou Z.;
RT   "A non-canonical role of the p97 complex in RIG-I antiviral signaling.";
RL   EMBO J. 34:2903-2920(2015).
RN   [62]
RP   INTERACTION WITH ZFAND2B.
RX   PubMed=26337389; DOI=10.1091/mbc.e15-02-0085;
RA   Braunstein I., Zach L., Allan S., Kalies K.U., Stanhill A.;
RT   "Proteasomal degradation of preemptive quality control (pQC) substrates is
RT   mediated by an AIRAPL-p97 complex.";
RL   Mol. Biol. Cell 26:3719-3727(2015).
RN   [63]
RP   INTERACTION WITH UBXN10.
RX   PubMed=26389662; DOI=10.1038/ncb3238;
RA   Raman M., Sergeev M., Garnaas M., Lydeard J.R., Huttlin E.L., Goessling W.,
RA   Shah J.V., Harper J.W.;
RT   "Systematic proteomics of the VCP-UBXD adaptor network identifies a role
RT   for UBXN10 in regulating ciliogenesis.";
RL   Nat. Cell Biol. 17:1356-1369(2015).
RN   [64]
RP   ACETYLATION [LARGE SCALE ANALYSIS] AT ALA-2, CLEAVAGE OF INITIATOR
RP   METHIONINE [LARGE SCALE ANALYSIS], AND IDENTIFICATION BY MASS SPECTROMETRY
RP   [LARGE SCALE ANALYSIS].
RX   PubMed=25944712; DOI=10.1002/pmic.201400617;
RA   Vaca Jacome A.S., Rabilloud T., Schaeffer-Reiss C., Rompais M., Ayoub D.,
RA   Lane L., Bairoch A., Van Dorsselaer A., Carapito C.;
RT   "N-terminome analysis of the human mitochondrial proteome.";
RL   Proteomics 15:2519-2524(2015).
RN   [65]
RP   INTERACTION WITH FAF1, AND SUBCELLULAR LOCATION.
RX   PubMed=26842564; DOI=10.1038/ncomms10612;
RA   Franz A., Pirson P.A., Pilger D., Halder S., Achuthankutty D., Kashkar H.,
RA   Ramadan K., Hoppe T.;
RT   "Chromatin-associated degradation is defined by UBXN-3/FAF1 to safeguard
RT   DNA replication fork progression.";
RL   Nat. Commun. 7:10612-10612(2016).
RN   [66]
RP   FUNCTION.
RX   PubMed=26692333; DOI=10.1038/nm.4013;
RA   Osorio F.G., Soria-Valles C., Santiago-Fernandez O., Bernal T.,
RA   Mittelbrunn M., Colado E., Rodriguez F., Bonzon-Kulichenko E., Vazquez J.,
RA   Porta-de-la-Riva M., Ceron J., Fueyo A., Li J., Green A.R., Freije J.M.,
RA   Lopez-Otin C.;
RT   "Loss of the proteostasis factor AIRAPL causes myeloid transformation by
RT   deregulating IGF-1 signaling.";
RL   Nat. Med. 22:91-96(2016).
RN   [67]
RP   INTERACTION WITH ANKZF1.
RX   PubMed=28302725; DOI=10.1074/jbc.m116.772038;
RA   van Haaften-Visser D.Y., Harakalova M., Mocholi E., van Montfrans J.M.,
RA   Elkadri A., Rieter E., Fiedler K., van Hasselt P.M., Triffaux E.M.M.,
RA   van Haelst M.M., Nijman I.J., Kloosterman W.P., Nieuwenhuis E.E.S.,
RA   Muise A.M., Cuppen E., Houwen R.H.J., Coffer P.J.;
RT   "Ankyrin repeat and zinc-finger domain-containing 1 mutations are
RT   associated with infantile-onset inflammatory bowel disease.";
RL   J. Biol. Chem. 292:7904-7920(2017).
RN   [68]
RP   SUMOYLATION [LARGE SCALE ANALYSIS] AT LYS-8 AND LYS-18, AND IDENTIFICATION
RP   BY MASS SPECTROMETRY [LARGE SCALE ANALYSIS].
RX   PubMed=28112733; DOI=10.1038/nsmb.3366;
RA   Hendriks I.A., Lyon D., Young C., Jensen L.J., Vertegaal A.C.,
RA   Nielsen M.L.;
RT   "Site-specific mapping of the human SUMO proteome reveals co-modification
RT   with phosphorylation.";
RL   Nat. Struct. Mol. Biol. 24:325-336(2017).
RN   [69]
RP   INTERACTION WITH ATXN3.
RX   PubMed=30455355; DOI=10.1074/jbc.ra118.005801;
RA   Weishaeupl D., Schneider J., Peixoto Pinheiro B., Ruess C., Dold S.M.,
RA   von Zweydorf F., Gloeckner C.J., Schmidt J., Riess O., Schmidt T.;
RT   "Physiological and pathophysiological characteristics of ataxin-3
RT   isoforms.";
RL   J. Biol. Chem. 294:644-661(2019).
RN   [70]
RP   INTERACTION WITH LMBR1L.
RX   PubMed=31073040; DOI=10.1126/science.aau0812;
RA   Choi J.H., Zhong X., McAlpine W., Liao T.C., Zhang D., Fang B., Russell J.,
RA   Ludwig S., Nair-Gill E., Zhang Z., Wang K.W., Misawa T., Zhan X., Choi M.,
RA   Wang T., Li X., Tang M., Sun Q., Yu L., Murray A.R., Moresco E.M.Y.,
RA   Beutler B.;
RT   "LMBR1L regulates lymphopoiesis through Wnt/beta-catenin signaling.";
RL   Science 364:0-0(2019).
RN   [71]
RP   FUNCTION, AND INTERACTION WITH TEX264.
RX   PubMed=32152270; DOI=10.1038/s41467-020-15000-w;
RA   Fielden J., Wiseman K., Torrecilla I., Li S., Hume S., Chiang S.C.,
RA   Ruggiano A., Narayan Singh A., Freire R., Hassanieh S., Domingo E.,
RA   Vendrell I., Fischer R., Kessler B.M., Maughan T.S., El-Khamisy S.F.,
RA   Ramadan K.;
RT   "TEX264 coordinates p97- and SPRTN-mediated resolution of topoisomerase 1-
RT   DNA adducts.";
RL   Nat. Commun. 11:1274-1274(2020).
RN   [72]
RP   X-RAY CRYSTALLOGRAPHY (2.2 ANGSTROMS) OF 1-481 IN COMPLEX WITH ATP ANALOG,
RP   CHARACTERIZATION OF VARIANTS IBMPFD1 GLY-95 AND HIS-155, MUTAGENESIS OF
RP   ARG-53 AND ARG-86, AND SUBUNIT.
RX   PubMed=20512113; DOI=10.1038/emboj.2010.104;
RA   Tang W.K., Li D., Li C.C., Esser L., Dai R., Guo L., Xia D.;
RT   "A novel ATP-dependent conformation in p97 N-D1 fragment revealed by
RT   crystal structures of disease-related mutants.";
RL   EMBO J. 29:2217-2229(2010).
RN   [73]
RP   X-RAY CRYSTALLOGRAPHY (1.9 ANGSTROMS) OF 797-806 IN COMPLEX WITH PLAA.
RX   PubMed=19887378; DOI=10.1074/jbc.m109.044685;
RA   Qiu L., Pashkova N., Walker J.R., Winistorfer S., Allali-Hassani A.,
RA   Akutsu M., Piper R., Dhe-Paganon S.;
RT   "Structure and function of the PLAA/Ufd3-p97/Cdc48 complex.";
RL   J. Biol. Chem. 285:365-372(2010).
RN   [74]
RP   X-RAY CRYSTALLOGRAPHY (1.8 ANGSTROMS) OF 1-187 IN COMPLEX WITH AMFR.
RX   PubMed=21914798; DOI=10.1074/jbc.m111.274506;
RA   Hanzelmann P., Schindelin H.;
RT   "The structural and functional basis of the p97/valosin-containing protein
RT   (VCP)-interacting motif (VIM): mutually exclusive binding of cofactors to
RT   the N-terminal domain of p97.";
RL   J. Biol. Chem. 286:38679-38690(2011).
RN   [75]
RP   VARIANTS IBMPFD1 GLY-95; CYS-155; HIS-155; PRO-155; GLN-191 AND GLU-232.
RX   PubMed=15034582; DOI=10.1038/ng1332;
RA   Watts G.D.J., Wymer J., Kovach M.J., Mehta S.G., Mumm S., Darvish D.,
RA   Pestronk A., Whyte M.P., Kimonis V.E.;
RT   "Inclusion body myopathy associated with Paget disease of bone and
RT   frontotemporal dementia is caused by mutant valosin-containing protein.";
RL   Nat. Genet. 36:377-381(2004).
RN   [76]
RP   VARIANT IBMPFD1 CYS-155.
RX   PubMed=15732117; DOI=10.1002/ana.20407;
RA   Schroeder R., Watts G.D.J., Mehta S.G., Evert B.O., Broich P.,
RA   Fliessbach K., Pauls K., Hans V.H., Kimonis V., Thal D.R.;
RT   "Mutant valosin-containing protein causes a novel type of frontotemporal
RT   dementia.";
RL   Ann. Neurol. 57:457-461(2005).
RN   [77]
RP   VARIANT IBMPFD1 HIS-159.
RX   PubMed=16247064; DOI=10.1212/01.wnl.0000180407.15369.92;
RA   Haubenberger D., Bittner R.E., Rauch-Shorny S., Zimprich F., Mannhalter C.,
RA   Wagner L., Mineva I., Vass K., Auff E., Zimprich A.;
RT   "Inclusion body myopathy and Paget disease is linked to a novel mutation in
RT   the VCP gene.";
RL   Neurology 65:1304-1305(2005).
RN   [78]
RP   CHARACTERIZATION OF VARIANTS IBMPFD1 GLY-95 AND HIS-155.
RX   PubMed=16321991; DOI=10.1093/hmg/ddi426;
RA   Weihl C.C., Dalal S., Pestronk A., Hanson P.I.;
RT   "Inclusion body myopathy-associated mutations in p97/VCP impair endoplasmic
RT   reticulum-associated degradation.";
RL   Hum. Mol. Genet. 15:189-199(2006).
RN   [79]
RP   VARIANTS IBMPFD1 TRP-198 AND HIS-387.
RX   PubMed=17935506; DOI=10.1111/j.1399-0004.2007.00887.x;
RA   Watts G.D., Thomasova D., Ramdeen S.K., Fulchiero E.C., Mehta S.G.,
RA   Drachman D.A., Weihl C.C., Jamrozik Z., Kwiecinski H., Kaminska A.,
RA   Kimonis V.E.;
RT   "Novel VCP mutations in inclusion body myopathy associated with Paget
RT   disease of bone and frontotemporal dementia.";
RL   Clin. Genet. 72:420-426(2007).
RN   [80]
RP   CHARACTERIZATION OF VARIANTS IBMPFD1 HIS-155; SER-155 AND GLU-232,
RP   MUTAGENESIS OF GLU-305 AND GLU-578, AND FUNCTION.
RX   PubMed=20104022; DOI=10.4161/auto.6.2.11014;
RA   Tresse E., Salomons F.A., Vesa J., Bott L.C., Kimonis V., Yao T.P.,
RA   Dantuma N.P., Taylor J.P.;
RT   "VCP/p97 is essential for maturation of ubiquitin-containing autophagosomes
RT   and this function is impaired by mutations that cause IBMPFD.";
RL   Autophagy 6:217-227(2010).
RN   [81]
RP   FUNCTION, INTERACTION WITH ZFAND1, MUTAGENESIS OF GLU-578, AND
RP   CHARACTERIZATION OF VARIANT IBMPFD1 HIS-155.
RX   PubMed=29804830; DOI=10.1016/j.molcel.2018.04.021;
RA   Turakhiya A., Meyer S.R., Marincola G., Boehm S., Vanselow J.T.,
RA   Schlosser A., Hofmann K., Buchberger A.;
RT   "ZFAND1 recruits p97 and the 26S proteasome to promote the clearance of
RT   arsenite-induced stress granules.";
RL   Mol. Cell 70:906-919(2018).
RN   [82]
RP   VARIANTS IBMPFD1 LEU-155 AND TRP-198.
RX   PubMed=20335036; DOI=10.1016/j.nmd.2010.03.002;
RA   Kumar K.R., Needham M., Mina K., Davis M., Brewer J., Staples C., Ng K.,
RA   Sue C.M., Mastaglia F.L.;
RT   "Two Australian families with inclusion-body myopathy, Paget's disease of
RT   bone and frontotemporal dementia: novel clinical and genetic findings.";
RL   Neuromuscul. Disord. 20:330-334(2010).
RN   [83]
RP   VARIANTS FTDALS6 HIS-155; GLY-159; GLN-191 AND ASN-592.
RX   PubMed=21145000; DOI=10.1016/j.neuron.2010.11.036;
RA   Johnson J.O., Mandrioli J., Benatar M., Abramzon Y., Van Deerlin V.M.,
RA   Trojanowski J.Q., Gibbs J.R., Brunetti M., Gronka S., Wuu J., Ding J.,
RA   McCluskey L., Martinez-Lage M., Falcone D., Hernandez D.G., Arepalli S.,
RA   Chong S., Schymick J.C., Rothstein J., Landi F., Wang Y.D., Calvo A.,
RA   Mora G., Sabatelli M., Monsurro M.R., Battistini S., Salvi F., Spataro R.,
RA   Sola P., Borghero G., Galassi G., Scholz S.W., Taylor J.P., Restagno G.,
RA   Chio A., Traynor B.J.;
RT   "Exome sequencing reveals VCP mutations as a cause of familial ALS.";
RL   Neuron 68:857-864(2010).
RN   [84]
RP   VARIANT CMT2Y LYS-185, CHARACTERIZATION OF VARIANT CMT2Y LYS-185, AND
RP   CHARACTERIZATION OF VARIANTS IBMPFD1 HIS-155 AND GLU-232.
RX   PubMed=25125609; DOI=10.1093/brain/awu224;
RA   Gonzalez M.A., Feely S.M., Speziani F., Strickland A.V., Danzi M.,
RA   Bacon C., Lee Y., Chou T.F., Blanton S.H., Weihl C.C., Zuchner S.,
RA   Shy M.E.;
RT   "A novel mutation in VCP causes Charcot-Marie-Tooth Type 2 disease.";
RL   Brain 137:2897-2902(2014).
RN   [85]
RP   VARIANT CMT2Y GLU-97, CHARACTERIZATION OF VARIANT CMT2Y GLU-97, AND
RP   CHARACTERIZATION OF VARIANTS IBMPFD1 HIS-155; TRP-198 AND GLU-232.
RX   PubMed=25878907; DOI=10.1155/2015/239167;
RA   Jerath N.U., Crockett C.D., Moore S.A., Shy M.E., Weihl C.C., Chou T.F.,
RA   Grider T., Gonzalez M.A., Zuchner S., Swenson A.;
RT   "Rare Manifestation of a c.290 C>T, p.Gly97Glu VCP Mutation.";
RL   Case Rep. Genet. 2015:239167-239167(2015).
RN   [86]
RP   VARIANT IBMPFD1 PHE-126.
RX   PubMed=27209344; DOI=10.1016/j.nmd.2016.05.001;
RA   Matsubara S., Shimizu T., Komori T., Mori-Yoshimura M., Minami N.,
RA   Hayashi Y.K.;
RT   "Nuclear inclusions mimicking poly(A)-binding protein nuclear 1 inclusions
RT   in a case of inclusion body myopathy associated with Paget disease of bone
RT   and frontotemporal dementia with a novel mutation in the valosin-containing
RT   protein gene.";
RL   Neuromuscul. Disord. 26:436-440(2016).
RN   [87]
RP   FUNCTION, INTERACTION WITH PLAA; UBXN6 AND YOD1, SUBCELLULAR LOCATION,
RP   CHARACTERIZATION OF VARIANTS IBMPFD1 HIS-155; TRP-198 AND GLU-232, AND
RP   MUTAGENESIS OF GLU-578.
RX   PubMed=27753622; DOI=10.15252/embj.201695148;
RA   Papadopoulos C., Kirchner P., Bug M., Grum D., Koerver L., Schulze N.,
RA   Poehler R., Dressler A., Fengler S., Arhzaouy K., Lux V., Ehrmann M.,
RA   Weihl C.C., Meyer H.;
RT   "VCP/p97 cooperates with YOD1, UBXD1 and PLAA to drive clearance of
RT   ruptured lysosomes by autophagy.";
RL   EMBO J. 36:135-150(2017).
CC   -!- FUNCTION: Necessary for the fragmentation of Golgi stacks during
CC       mitosis and for their reassembly after mitosis. Involved in the
CC       formation of the transitional endoplasmic reticulum (tER). The transfer
CC       of membranes from the endoplasmic reticulum to the Golgi apparatus
CC       occurs via 50-70 nm transition vesicles which derive from part-rough,
CC       part-smooth transitional elements of the endoplasmic reticulum (tER).
CC       Vesicle budding from the tER is an ATP-dependent process. The ternary
CC       complex containing UFD1, VCP and NPLOC4 binds ubiquitinated proteins
CC       and is necessary for the export of misfolded proteins from the ER to
CC       the cytoplasm, where they are degraded by the proteasome. The NPLOC4-
CC       UFD1-VCP complex regulates spindle disassembly at the end of mitosis
CC       and is necessary for the formation of a closed nuclear envelope.
CC       Regulates E3 ubiquitin-protein ligase activity of RNF19A. Component of
CC       the VCP/p97-AMFR/gp78 complex that participates in the final step of
CC       the sterol-mediated ubiquitination and endoplasmic reticulum-associated
CC       degradation (ERAD) of HMGCR. Involved in endoplasmic reticulum stress-
CC       induced pre-emptive quality control, a mechanism that selectively
CC       attenuates the translocation of newly synthesized proteins into the
CC       endoplasmic reticulum and reroutes them to the cytosol for proteasomal
CC       degradation (PubMed:26565908). Plays a role in the regulation of stress
CC       granules (SGs) clearance process upon arsenite-induced response
CC       (PubMed:29804830). Also involved in DNA damage response: recruited to
CC       double-strand breaks (DSBs) sites in a RNF8- and RNF168-dependent
CC       manner and promotes the recruitment of TP53BP1 at DNA damage sites
CC       (PubMed:22020440, PubMed:22120668). Recruited to stalled replication
CC       forks by SPRTN: may act by mediating extraction of DNA polymerase eta
CC       (POLH) to prevent excessive translesion DNA synthesis and limit the
CC       incidence of mutations induced by DNA damage (PubMed:23042607,
CC       PubMed:23042605). Together with SPRTN metalloprotease, involved in the
CC       repair of covalent DNA-protein cross-links (DPCs) during DNA synthesis
CC       (PubMed:32152270). Involved in interstrand cross-link repair in
CC       response to replication stress by mediating unloading of the
CC       ubiquitinated CMG helicase complex (By similarity). Required for
CC       cytoplasmic retrotranslocation of stressed/damaged mitochondrial outer-
CC       membrane proteins and their subsequent proteasomal degradation
CC       (PubMed:16186510, PubMed:21118995). Essential for the maturation of
CC       ubiquitin-containing autophagosomes and the clearance of ubiquitinated
CC       protein by autophagy (PubMed:20104022, PubMed:27753622). Acts as a
CC       negative regulator of type I interferon production by interacting with
CC       DDX58/RIG-I: interaction takes place when DDX58/RIG-I is ubiquitinated
CC       via 'Lys-63'-linked ubiquitin on its CARD domains, leading to recruit
CC       RNF125 and promote ubiquitination and degradation of DDX58/RIG-I
CC       (PubMed:26471729). May play a role in the ubiquitin-dependent sorting
CC       of membrane proteins to lysosomes where they undergo degradation
CC       (PubMed:21822278). May more particularly play a role in caveolins
CC       sorting in cells (PubMed:21822278, PubMed:23335559). By controlling the
CC       steady-state expression of the IGF1R receptor, indirectly regulates the
CC       insulin-like growth factor receptor signaling pathway
CC       (PubMed:26692333). {ECO:0000250|UniProtKB:P23787,
CC       ECO:0000269|PubMed:15456787, ECO:0000269|PubMed:16168377,
CC       ECO:0000269|PubMed:16186510, ECO:0000269|PubMed:20104022,
CC       ECO:0000269|PubMed:21118995, ECO:0000269|PubMed:21822278,
CC       ECO:0000269|PubMed:22020440, ECO:0000269|PubMed:22120668,
CC       ECO:0000269|PubMed:22607976, ECO:0000269|PubMed:23042605,
CC       ECO:0000269|PubMed:23042607, ECO:0000269|PubMed:23335559,
CC       ECO:0000269|PubMed:26471729, ECO:0000269|PubMed:26565908,
CC       ECO:0000269|PubMed:26692333, ECO:0000269|PubMed:27753622,
CC       ECO:0000269|PubMed:29804830, ECO:0000269|PubMed:32152270}.
CC   -!- CATALYTIC ACTIVITY:
CC       Reaction=ATP + H2O = ADP + H(+) + phosphate; Xref=Rhea:RHEA:13065,
CC         ChEBI:CHEBI:15377, ChEBI:CHEBI:15378, ChEBI:CHEBI:30616,
CC         ChEBI:CHEBI:43474, ChEBI:CHEBI:456216; EC=3.6.4.6;
CC         Evidence={ECO:0000269|PubMed:26471729};
CC   -!- SUBUNIT: Homohexamer. Forms a ring-shaped particle of 12.5 nm diameter,
CC       that displays 6-fold radial symmetry. Part of a ternary complex
CC       containing STX5A, NSFL1C and VCP. NSFL1C forms a homotrimer that binds
CC       to one end of a VCP homohexamer. The complex binds to membranes
CC       enriched in phosphatidylethanolamine-containing lipids and promotes
CC       Golgi membrane fusion. Binds to a heterodimer of NPLOC4 and UFD1,
CC       binding to this heterodimer inhibits Golgi-membrane fusion
CC       (PubMed:26471729). Interaction with VCIP135 leads to dissociation of
CC       the complex via ATP hydrolysis by VCP. Part of a ternary complex
CC       containing NPLOC4, UFD1 and VCP. Interacts with NSFL1C-like protein
CC       p37; the complex has membrane fusion activity and is required for Golgi
CC       and endoplasmic reticulum biogenesis. Interacts with SELENOS and SYVN1,
CC       as well as with DERL1, DERL2 and DERL3; which probably transfer
CC       misfolded proteins from the ER to VCP. Interacts with SVIP. Component
CC       of a complex required to couple retrotranslocation, ubiquitination and
CC       deglycosylation composed of NGLY1, SAKS1, AMFR, VCP and RAD23B.
CC       Directly interacts with UBXN4 and RNF19A. Interacts with CASR.
CC       Interacts with UBE4B and YOD1. Interacts with clathrin. Interacts with
CC       RNF103. Interacts with TRIM13 and TRIM21. Component of a VCP/p97-
CC       AMFR/gp78 complex that participates in the final step of the
CC       endoplasmic reticulum-associated degradation (ERAD) of HMGCR. Interacts
CC       directly with AMFR/gp78 (via its VIM). Interacts with RHBDD1 (via C-
CC       terminal domain). Interacts with SPRTN; leading to recruitment to
CC       stalled replication forks (PubMed:23042607, PubMed:23042605). Interacts
CC       with WASHC5. Interacts with UBOX5. Interacts (via N-terminus) with
CC       UBXN7, UBXN8, and probably several other UBX domain-containing proteins
CC       (via UBX domains); the interactions are mutually exclusive with VIM-
CC       dependent interactions such as those with AMFR and SELENOS. Forms a
CC       complex with UBQLN1 and UBXN4. Interacts (via the PIM motif) with RNF31
CC       (via the PUB domain) (PubMed:24726327). Interacts with DDX58/RIG-I and
CC       RNF125; interaction takes place when DDX58/RIG-I is ubiquitinated via
CC       'Lys-63'-linked ubiquitin on its CARD domains, leading to recruit
CC       RNF125 and promote ubiquitination and degradation of DDX58/RIG-I
CC       (PubMed:26471729). Interacts with BAG6 (PubMed:21636303). Interacts
CC       with UBXN10 (PubMed:26389662). Interacts with UBXN6; the interaction
CC       with UBXN6 is direct and competitive with UFD1 (PubMed:19174149,
CC       PubMed:19275885). Forms a ternary complex with CAV1 and UBXN6
CC       (PubMed:21822278, PubMed:18656546, PubMed:19174149). Interacts with
CC       PLAA, UBXN6 and YOD1; may form a complex involved in macroautophagy
CC       (PubMed:27753622). Interacts with ANKZF1 (PubMed:28302725). Interacts
CC       with ubiquitin-binding protein FAF1 (PubMed:26842564). Interacts with
CC       ZFAND2B (via VIM motif); the interaction is direct (PubMed:24160817,
CC       PubMed:26337389). Interacts with ZFAND1 (via its ubiquitin-like
CC       region); this interaction occurs in an arsenite-dependent manner
CC       (PubMed:29804830). Interacts with CCDC47 (By similarity). Interacts
CC       with UBAC2 (By similarity). Interacts with LMBR1L (PubMed:31073040).
CC       Interacts with ATXN3 (PubMed:30455355). Interacts with TEX264; bridging
CC       VCP to covalent DNA-protein cross-links (DPCs) (PubMed:32152270).
CC       {ECO:0000250|UniProtKB:P46462, ECO:0000250|UniProtKB:Q01853,
CC       ECO:0000269|PubMed:15215856, ECO:0000269|PubMed:15362974,
CC       ECO:0000269|PubMed:15456787, ECO:0000269|PubMed:16168377,
CC       ECO:0000269|PubMed:16186509, ECO:0000269|PubMed:16186510,
CC       ECO:0000269|PubMed:16289116, ECO:0000269|PubMed:16449189,
CC       ECO:0000269|PubMed:16513638, ECO:0000269|PubMed:16968747,
CC       ECO:0000269|PubMed:17314412, ECO:0000269|PubMed:18022694,
CC       ECO:0000269|PubMed:18656546, ECO:0000269|PubMed:18675248,
CC       ECO:0000269|PubMed:19174149, ECO:0000269|PubMed:19275885,
CC       ECO:0000269|PubMed:19818707, ECO:0000269|PubMed:19822669,
CC       ECO:0000269|PubMed:19887378, ECO:0000269|PubMed:20512113,
CC       ECO:0000269|PubMed:20833645, ECO:0000269|PubMed:21636303,
CC       ECO:0000269|PubMed:21822278, ECO:0000269|PubMed:21914798,
CC       ECO:0000269|PubMed:21949850, ECO:0000269|PubMed:22120668,
CC       ECO:0000269|PubMed:22537386, ECO:0000269|PubMed:22795130,
CC       ECO:0000269|PubMed:22902628, ECO:0000269|PubMed:23042605,
CC       ECO:0000269|PubMed:23042607, ECO:0000269|PubMed:24160817,
CC       ECO:0000269|PubMed:24726327, ECO:0000269|PubMed:26337389,
CC       ECO:0000269|PubMed:26389662, ECO:0000269|PubMed:26471729,
CC       ECO:0000269|PubMed:26842564, ECO:0000269|PubMed:27753622,
CC       ECO:0000269|PubMed:28302725, ECO:0000269|PubMed:29804830,
CC       ECO:0000269|PubMed:30455355, ECO:0000269|PubMed:32152270,
CC       ECO:0000269|PubMed:8413590, ECO:0000305|PubMed:31073040}.
CC   -!- INTERACTION:
CC       P55072; Q9UKV5: AMFR; NbExp=12; IntAct=EBI-355164, EBI-1046367;
CC       P55072; Q9BZE9: ASPSCR1; NbExp=28; IntAct=EBI-355164, EBI-1993677;
CC       P55072; P54253: ATXN1; NbExp=3; IntAct=EBI-355164, EBI-930964;
CC       P55072; P54252: ATXN3; NbExp=4; IntAct=EBI-355164, EBI-946046;
CC       P55072; P54252-1: ATXN3; NbExp=16; IntAct=EBI-355164, EBI-946068;
CC       P55072; Q96LK0: CEP19; NbExp=3; IntAct=EBI-355164, EBI-741885;
CC       P55072; O96017: CHEK2; NbExp=2; IntAct=EBI-355164, EBI-1180783;
CC       P55072; O75175: CNOT3; NbExp=3; IntAct=EBI-355164, EBI-743073;
CC       P55072; Q13619: CUL4A; NbExp=2; IntAct=EBI-355164, EBI-456106;
CC       P55072; O60941-5: DTNB; NbExp=3; IntAct=EBI-355164, EBI-11984733;
CC       P55072; P26378-2: ELAVL4; NbExp=3; IntAct=EBI-355164, EBI-21603100;
CC       P55072; Q96J88-3: EPSTI1; NbExp=3; IntAct=EBI-355164, EBI-25885343;
CC       P55072; Q9UNN5-1: FAF1; NbExp=4; IntAct=EBI-355164, EBI-15930546;
CC       P55072; Q96CS3: FAF2; NbExp=13; IntAct=EBI-355164, EBI-1055805;
CC       P55072; Q969W3: FAM104A; NbExp=6; IntAct=EBI-355164, EBI-10281506;
CC       P55072; O94868: FCHSD2; NbExp=2; IntAct=EBI-355164, EBI-1215612;
CC       P55072; P09471: GNAO1; NbExp=3; IntAct=EBI-355164, EBI-715087;
CC       P55072; P62993: GRB2; NbExp=3; IntAct=EBI-355164, EBI-401755;
CC       P55072; P42858: HTT; NbExp=10; IntAct=EBI-355164, EBI-466029;
CC       P55072; Q8TBB1: LNX1; NbExp=4; IntAct=EBI-355164, EBI-739832;
CC       P55072; Q8WZA0: LZIC; NbExp=3; IntAct=EBI-355164, EBI-5774346;
CC       P55072; Q9H7H0-2: METTL17; NbExp=3; IntAct=EBI-355164, EBI-11098807;
CC       P55072; Q8TAT6: NPLOC4; NbExp=9; IntAct=EBI-355164, EBI-1994109;
CC       P55072; Q9UNZ2: NSFL1C; NbExp=20; IntAct=EBI-355164, EBI-721577;
CC       P55072; Q96HA8: NTAQ1; NbExp=3; IntAct=EBI-355164, EBI-741158;
CC       P55072; Q9Y263: PLAA; NbExp=3; IntAct=EBI-355164, EBI-1994037;
CC       P55072; Q07869: PPARA; NbExp=3; IntAct=EBI-355164, EBI-78615;
CC       P55072; P62136: PPP1CA; NbExp=2; IntAct=EBI-355164, EBI-357253;
CC       P55072; P07602-1: PSAP; NbExp=3; IntAct=EBI-355164, EBI-10635648;
CC       P55072; P25786: PSMA1; NbExp=3; IntAct=EBI-355164, EBI-359352;
CC       P55072; P62191: PSMC1; NbExp=3; IntAct=EBI-355164, EBI-357598;
CC       P55072; P26045: PTPN3; NbExp=2; IntAct=EBI-355164, EBI-1047946;
CC       P55072; Q9Y4L5: RNF115; NbExp=3; IntAct=EBI-355164, EBI-2129242;
CC       P55072; P32969: RPL9P9; NbExp=3; IntAct=EBI-355164, EBI-358122;
CC       P55072; Q9H0K1: SIK2; NbExp=4; IntAct=EBI-355164, EBI-1181664;
CC       P55072; Q8NBI5: SLC43A3; NbExp=3; IntAct=EBI-355164, EBI-2855542;
CC       P55072; Q16560-2: SNRNP35; NbExp=3; IntAct=EBI-355164, EBI-12938570;
CC       P55072; P46977: STT3A; NbExp=3; IntAct=EBI-355164, EBI-719212;
CC       P55072; P51668: UBE2D1; NbExp=3; IntAct=EBI-355164, EBI-743540;
CC       P55072; B1AQ61: UBE4B; NbExp=4; IntAct=EBI-355164, EBI-7931266;
CC       P55072; O94941: UBOX5; NbExp=3; IntAct=EBI-355164, EBI-751901;
CC       P55072; Q96LJ8: UBXN10; NbExp=7; IntAct=EBI-355164, EBI-1993941;
CC       P55072; Q5T124-6: UBXN11; NbExp=7; IntAct=EBI-355164, EBI-11524408;
CC       P55072; P68543: UBXN2A; NbExp=17; IntAct=EBI-355164, EBI-1993668;
CC       P55072; Q14CS0: UBXN2B; NbExp=11; IntAct=EBI-355164, EBI-1993619;
CC       P55072; Q92575: UBXN4; NbExp=11; IntAct=EBI-355164, EBI-723441;
CC       P55072; Q9BZV1: UBXN6; NbExp=20; IntAct=EBI-355164, EBI-1993899;
CC       P55072; O94888: UBXN7; NbExp=18; IntAct=EBI-355164, EBI-1993627;
CC       P55072; O00124: UBXN8; NbExp=8; IntAct=EBI-355164, EBI-1993850;
CC       P55072; Q92890: UFD1; NbExp=5; IntAct=EBI-355164, EBI-1994090;
CC       P55072; P63027: VAMP2; NbExp=3; IntAct=EBI-355164, EBI-520113;
CC       P55072; P55072: VCP; NbExp=7; IntAct=EBI-355164, EBI-355164;
CC       P55072; Q6GPH4: XAF1; NbExp=3; IntAct=EBI-355164, EBI-2815120;
CC       P55072; Q5VVQ6: YOD1; NbExp=3; IntAct=EBI-355164, EBI-2510804;
CC       P55072; P63104: YWHAZ; NbExp=2; IntAct=EBI-355164, EBI-347088;
CC       P55072; P24278: ZBTB25; NbExp=3; IntAct=EBI-355164, EBI-739899;
CC       P55072; Q9WTX6: Cul1; Xeno; NbExp=2; IntAct=EBI-355164, EBI-1551052;
CC   -!- SUBCELLULAR LOCATION: Cytoplasm, cytosol {ECO:0000269|PubMed:15456787}.
CC       Endoplasmic reticulum {ECO:0000269|PubMed:15215856}. Nucleus
CC       {ECO:0000269|PubMed:23042605, ECO:0000269|PubMed:26842564}. Cytoplasm,
CC       Stress granule {ECO:0000269|PubMed:29804830}. Note=Present in the
CC       neuronal hyaline inclusion bodies specifically found in motor neurons
CC       from amyotrophic lateral sclerosis patients (PubMed:15456787). Present
CC       in the Lewy bodies specifically found in neurons from Parkinson disease
CC       patients (PubMed:15456787). Recruited to the cytoplasmic surface of the
CC       endoplasmic reticulum via interaction with AMFR/gp78 (PubMed:16168377).
CC       Following DNA double-strand breaks, recruited to the sites of damage
CC       (PubMed:22120668). Recruited to stalled replication forks via
CC       interaction with SPRTN (PubMed:23042605). Recruited to damaged
CC       lysosomes decorated with K48-linked ubiquitin chains (PubMed:27753622).
CC       Colocalizes with TIA1, ZFAND1 and G3BP1 in cytoplasmic stress granules
CC       (SGs) in response to arsenite-induced stress treatment
CC       (PubMed:29804830). {ECO:0000269|PubMed:15456787,
CC       ECO:0000269|PubMed:16168377, ECO:0000269|PubMed:22120668,
CC       ECO:0000269|PubMed:23042605, ECO:0000269|PubMed:27753622,
CC       ECO:0000269|PubMed:29804830}.
CC   -!- DOMAIN: The PIM (PUB-interaction motif) motif mediates interaction with
CC       the PUB domain of RNF31. {ECO:0000269|PubMed:24726327}.
CC   -!- PTM: Phosphorylated by tyrosine kinases in response to T-cell antigen
CC       receptor activation. Phosphorylated in mitotic cells.
CC       {ECO:0000250|UniProtKB:P46462}.
CC   -!- PTM: ISGylated. {ECO:0000269|PubMed:16139798}.
CC   -!- PTM: Methylation at Lys-315 catalyzed by VCPKMT is increased in the
CC       presence of ASPSCR1. Lys-315 methylation may decrease ATPase activity.
CC       {ECO:0000269|PubMed:22948820, ECO:0000269|PubMed:23349634}.
CC   -!- DISEASE: Inclusion body myopathy with early-onset Paget disease with or
CC       without frontotemporal dementia 1 (IBMPFD1) [MIM:167320]: An autosomal
CC       dominant disease characterized by disabling muscle weakness clinically
CC       resembling to limb girdle muscular dystrophy, osteolytic bone lesions
CC       consistent with Paget disease, and premature frontotemporal dementia.
CC       Clinical features show incomplete penetrance.
CC       {ECO:0000269|PubMed:15034582, ECO:0000269|PubMed:15732117,
CC       ECO:0000269|PubMed:16247064, ECO:0000269|PubMed:16321991,
CC       ECO:0000269|PubMed:17935506, ECO:0000269|PubMed:20104022,
CC       ECO:0000269|PubMed:20335036, ECO:0000269|PubMed:20512113,
CC       ECO:0000269|PubMed:21822278, ECO:0000269|PubMed:23349634,
CC       ECO:0000269|PubMed:25125609, ECO:0000269|PubMed:25878907,
CC       ECO:0000269|PubMed:27209344, ECO:0000269|PubMed:27753622,
CC       ECO:0000269|PubMed:29804830}. Note=The disease is caused by variants
CC       affecting the gene represented in this entry.
CC   -!- DISEASE: Frontotemporal dementia and/or amyotrophic lateral sclerosis 6
CC       (FTDALS6) [MIM:613954]: A neurodegenerative disorder characterized by
CC       frontotemporal dementia and/or amyotrophic lateral sclerosis in
CC       affected individuals. There is high intrafamilial variation.
CC       Frontotemporal dementia (FTD) is characterized by frontal and temporal
CC       lobe atrophy associated with neuronal loss, gliosis, and dementia.
CC       Patients exhibit progressive changes in social, behavioral, and/or
CC       language function. Amyotrophic lateral sclerosis (ALS) is characterized
CC       by the death of motor neurons in the brain, brainstem, and spinal cord,
CC       resulting in fatal paralysis. FTDALS6 is an autosomal dominant form
CC       characterized by onset of ALS or FTD in adulthood. Some patients with
CC       the disorder may have features of both diseases.
CC       {ECO:0000269|PubMed:21145000, ECO:0000269|PubMed:23349634}. Note=The
CC       disease is caused by variants affecting the gene represented in this
CC       entry.
CC   -!- DISEASE: Charcot-Marie-Tooth disease 2Y (CMT2Y) [MIM:616687]: An
CC       autosomal dominant, axonal form of Charcot-Marie-Tooth disease, a
CC       disorder of the peripheral nervous system, characterized by progressive
CC       weakness and atrophy, initially of the peroneal muscles and later of
CC       the distal muscles of the arms. Charcot-Marie-Tooth disease is
CC       classified in two main groups on the basis of electrophysiologic
CC       properties and histopathology: primary peripheral demyelinating
CC       neuropathies (designated CMT1 when they are dominantly inherited) and
CC       primary peripheral axonal neuropathies (CMT2). Neuropathies of the CMT2
CC       group are characterized by signs of axonal degeneration in the absence
CC       of obvious myelin alterations, normal or slightly reduced nerve
CC       conduction velocities, and progressive distal muscle weakness and
CC       atrophy. {ECO:0000269|PubMed:25125609, ECO:0000269|PubMed:25878907}.
CC       Note=The disease is caused by variants affecting the gene represented
CC       in this entry.
CC   -!- SIMILARITY: Belongs to the AAA ATPase family. {ECO:0000305}.
CC   -!- CAUTION: It is unclear how it participates in the recruitment of
CC       TP53BP1 at DNA damage sites. According to a first report, participates
CC       in the recruitment of TP53BP1 by promoting ubiquitination and removal
CC       of L3MBTL1 from DNA damage sites (PubMed:22120668). According to a
CC       second report, it acts by removing 'Lys-48'-linked ubiquitination from
CC       sites of DNA damage (PubMed:22020440). {ECO:0000305|PubMed:22020440,
CC       ECO:0000305|PubMed:22120668}.
CC   ---------------------------------------------------------------------------
CC   Copyrighted by the UniProt Consortium, see https://www.uniprot.org/terms
CC   Distributed under the Creative Commons Attribution (CC BY 4.0) License
CC   ---------------------------------------------------------------------------
DR   EMBL; AC004472; AAC07984.1; -; Genomic_DNA.
DR   EMBL; AF100752; AAD43016.1; -; mRNA.
DR   EMBL; AK312310; BAG35235.1; -; mRNA.
DR   EMBL; AL353795; -; NOT_ANNOTATED_CDS; Genomic_DNA.
DR   EMBL; CH471071; EAW58404.1; -; Genomic_DNA.
DR   EMBL; BC110913; AAI10914.1; -; mRNA.
DR   EMBL; BC121794; AAI21795.1; -; mRNA.
DR   EMBL; Z70768; CAA94809.1; -; mRNA.
DR   CCDS; CCDS6573.1; -.
DR   PIR; T02243; T02243.
DR   RefSeq; NP_009057.1; NM_007126.3.
DR   PDB; 3EBB; X-ray; 1.90 A; E/F/G/H=797-806.
DR   PDB; 3HU1; X-ray; 2.81 A; A/B/C/D/E/F=1-481.
DR   PDB; 3HU2; X-ray; 2.85 A; A/B/C/D/E/F=1-481.
DR   PDB; 3HU3; X-ray; 2.20 A; A/B=1-481.
DR   PDB; 3QC8; X-ray; 2.20 A; A=21-196.
DR   PDB; 3QQ7; X-ray; 2.65 A; A=2-187.
DR   PDB; 3QQ8; X-ray; 2.00 A; A=2-187.
DR   PDB; 3QWZ; X-ray; 2.00 A; A=1-208.
DR   PDB; 3TIW; X-ray; 1.80 A; A/B=1-187.
DR   PDB; 4KDI; X-ray; 1.86 A; A/B=21-196.
DR   PDB; 4KDL; X-ray; 1.81 A; A=21-196.
DR   PDB; 4KLN; X-ray; 2.62 A; A/B/C/D/E/F=1-481.
DR   PDB; 4KO8; X-ray; 1.98 A; A/B=1-481.
DR   PDB; 4KOD; X-ray; 2.96 A; A/B/C/D/E/F/G/H/I/J/K/L=1-481.
DR   PDB; 4P0A; X-ray; 2.30 A; B/D=797-806.
DR   PDB; 5B6C; X-ray; 1.55 A; A=21-191.
DR   PDB; 5C18; X-ray; 3.30 A; A/B/C/D/E/F=2-806.
DR   PDB; 5C19; X-ray; 4.20 A; A/B/C/D/E/F=2-806.
DR   PDB; 5C1A; X-ray; 3.80 A; A/B/C/D/E/F/G/H/I/J/K/L=2-806.
DR   PDB; 5C1B; X-ray; 3.08 A; A/B/C/D/E/F=2-806.
DR   PDB; 5DYG; X-ray; 2.20 A; A=1-460.
DR   PDB; 5DYI; X-ray; 3.71 A; A/B/C/D/E/F/G/H/I/J/K/L=1-481.
DR   PDB; 5EPP; X-ray; 1.88 A; A=21-199.
DR   PDB; 5FTJ; EM; 2.30 A; A/B/C/D/E/F=1-806.
DR   PDB; 5FTK; EM; 2.40 A; A/B/C/D/E/F=1-806.
DR   PDB; 5FTL; EM; 3.30 A; A/B/C/D/E/F=1-806.
DR   PDB; 5FTM; EM; 3.20 A; A/B/C/D/E/F=1-806.
DR   PDB; 5FTN; EM; 3.30 A; A/B/C/D/E/F=1-806.
DR   PDB; 5GLF; X-ray; 2.25 A; A/C/E/G=21-199.
DR   PDB; 5IFS; X-ray; 2.46 A; B/D=1-481.
DR   PDB; 5IFW; X-ray; 3.40 A; B=2-806.
DR   PDB; 5KIW; X-ray; 3.41 A; A/B=1-460.
DR   PDB; 5KIY; X-ray; 2.79 A; A=1-460.
DR   PDB; 5X4L; X-ray; 2.40 A; A/B=23-196.
DR   PDB; 6G2V; X-ray; 1.90 A; A=462-764.
DR   PDB; 6G2W; X-ray; 2.68 A; A=462-764.
DR   PDB; 6G2X; X-ray; 2.08 A; A=462-764.
DR   PDB; 6G2Y; X-ray; 2.15 A; A=462-764.
DR   PDB; 6G2Z; X-ray; 1.92 A; A=462-764.
DR   PDB; 6G30; X-ray; 2.42 A; A=462-764.
DR   PDB; 6HD0; X-ray; 3.73 A; A/B/C/T/U/V=1-481.
DR   PDB; 6MCK; X-ray; 3.77 A; A/B/C/D/E/F/G/H/I/J/K/L=210-806.
DR   PDB; 7JY5; EM; 2.89 A; A/B/C/D/E/F=1-806.
DR   PDBsum; 3EBB; -.
DR   PDBsum; 3HU1; -.
DR   PDBsum; 3HU2; -.
DR   PDBsum; 3HU3; -.
DR   PDBsum; 3QC8; -.
DR   PDBsum; 3QQ7; -.
DR   PDBsum; 3QQ8; -.
DR   PDBsum; 3QWZ; -.
DR   PDBsum; 3TIW; -.
DR   PDBsum; 4KDI; -.
DR   PDBsum; 4KDL; -.
DR   PDBsum; 4KLN; -.
DR   PDBsum; 4KO8; -.
DR   PDBsum; 4KOD; -.
DR   PDBsum; 4P0A; -.
DR   PDBsum; 5B6C; -.
DR   PDBsum; 5C18; -.
DR   PDBsum; 5C19; -.
DR   PDBsum; 5C1A; -.
DR   PDBsum; 5C1B; -.
DR   PDBsum; 5DYG; -.
DR   PDBsum; 5DYI; -.
DR   PDBsum; 5EPP; -.
DR   PDBsum; 5FTJ; -.
DR   PDBsum; 5FTK; -.
DR   PDBsum; 5FTL; -.
DR   PDBsum; 5FTM; -.
DR   PDBsum; 5FTN; -.
DR   PDBsum; 5GLF; -.
DR   PDBsum; 5IFS; -.
DR   PDBsum; 5IFW; -.
DR   PDBsum; 5KIW; -.
DR   PDBsum; 5KIY; -.
DR   PDBsum; 5X4L; -.
DR   PDBsum; 6G2V; -.
DR   PDBsum; 6G2W; -.
DR   PDBsum; 6G2X; -.
DR   PDBsum; 6G2Y; -.
DR   PDBsum; 6G2Z; -.
DR   PDBsum; 6G30; -.
DR   PDBsum; 6HD0; -.
DR   PDBsum; 6MCK; -.
DR   PDBsum; 7JY5; -.
DR   SMR; P55072; -.
DR   BioGRID; 113258; 976.
DR   ComplexPortal; CPX-137; VCP-NPL4-UFD1 AAA ATPase complex.
DR   ComplexPortal; CPX-262; NSFL1C-VCP complex.
DR   CORUM; P55072; -.
DR   DIP; DIP-33543N; -.
DR   IntAct; P55072; 161.
DR   MINT; P55072; -.
DR   STRING; 9606.ENSP00000351777; -.
DR   BindingDB; P55072; -.
DR   ChEMBL; CHEMBL1075145; -.
DR   DrugBank; DB12695; Phenethyl Isothiocyanate.
DR   DrugBank; DB04395; Phosphoaminophosphonic Acid-Adenylate Ester.
DR   DrugCentral; P55072; -.
DR   MoonDB; P55072; Predicted.
DR   TCDB; 3.A.16.1.1; the endoplasmic reticular retrotranslocon (er-rt) family.
DR   CarbonylDB; P55072; -.
DR   iPTMnet; P55072; -.
DR   MetOSite; P55072; -.
DR   PhosphoSitePlus; P55072; -.
DR   SwissPalm; P55072; -.
DR   BioMuta; VCP; -.
DR   DMDM; 6094447; -.
DR   DOSAC-COBS-2DPAGE; P55072; -.
DR   OGP; P55072; -.
DR   REPRODUCTION-2DPAGE; IPI00022774; -.
DR   REPRODUCTION-2DPAGE; P55072; -.
DR   CPTAC; CPTAC-295; -.
DR   CPTAC; CPTAC-296; -.
DR   EPD; P55072; -.
DR   jPOST; P55072; -.
DR   MassIVE; P55072; -.
DR   PaxDb; P55072; -.
DR   PeptideAtlas; P55072; -.
DR   PRIDE; P55072; -.
DR   ProteomicsDB; 56776; -.
DR   TopDownProteomics; P55072; -.
DR   Antibodypedia; 2215; 649 antibodies.
DR   DNASU; 7415; -.
DR   Ensembl; ENST00000358901; ENSP00000351777; ENSG00000165280.
DR   GeneID; 7415; -.
DR   KEGG; hsa:7415; -.
DR   CTD; 7415; -.
DR   DisGeNET; 7415; -.
DR   GeneCards; VCP; -.
DR   GeneReviews; VCP; -.
DR   HGNC; HGNC:12666; VCP.
DR   HPA; ENSG00000165280; Low tissue specificity.
DR   MalaCards; VCP; -.
DR   MIM; 167320; phenotype.
DR   MIM; 601023; gene.
DR   MIM; 613954; phenotype.
DR   MIM; 616687; phenotype.
DR   neXtProt; NX_P55072; -.
DR   OpenTargets; ENSG00000165280; -.
DR   Orphanet; 329478; Adult-onset distal myopathy due to VCP mutation.
DR   Orphanet; 803; Amyotrophic lateral sclerosis.
DR   Orphanet; 435387; Autosomal dominant Charcot-Marie-Tooth disease type 2Y.
DR   Orphanet; 275864; Behavioral variant of frontotemporal dementia.
DR   Orphanet; 275872; Frontotemporal dementia with motor neuron disease.
DR   Orphanet; 52430; Inclusion body myopathy with Paget disease of bone and frontotemporal dementia.
DR   Orphanet; 100070; Progressive non-fluent aphasia.
DR   Orphanet; 329475; Spastic paraplegia-Paget disease of bone syndrome.
DR   PharmGKB; PA37289; -.
DR   VEuPathDB; HostDB:ENSG00000165280.15; -.
DR   eggNOG; KOG0730; Eukaryota.
DR   GeneTree; ENSGT00900000141071; -.
DR   HOGENOM; CLU_000688_12_3_1; -.
DR   InParanoid; P55072; -.
DR   OMA; PIDDTTE; -.
DR   OrthoDB; 194195at2759; -.
DR   PhylomeDB; P55072; -.
DR   TreeFam; TF300542; -.
DR   BRENDA; 3.6.4.6; 2681.
DR   PathwayCommons; P55072; -.
DR   Reactome; R-HSA-110320; Translesion Synthesis by POLH.
DR   Reactome; R-HSA-3371511; HSF1 activation.
DR   Reactome; R-HSA-382556; ABC-family proteins mediated transport.
DR   Reactome; R-HSA-532668; N-glycan trimming in the ER and Calnexin/Calreticulin cycle.
DR   Reactome; R-HSA-5358346; Hedgehog ligand biogenesis.
DR   Reactome; R-HSA-5362768; Hh mutants are degraded by ERAD.
DR   Reactome; R-HSA-5678895; Defective CFTR causes cystic fibrosis.
DR   Reactome; R-HSA-5689877; Josephin domain DUBs.
DR   Reactome; R-HSA-5689896; Ovarian tumor domain proteases.
DR   Reactome; R-HSA-6798695; Neutrophil degranulation.
DR   Reactome; R-HSA-8866654; E3 ubiquitin ligases ubiquitinate target proteins.
DR   Reactome; R-HSA-8876725; Protein methylation.
DR   Reactome; R-HSA-9646399; Aggrephagy.
DR   Reactome; R-HSA-9678110; Attachment and Entry.
DR   Reactome; R-HSA-9694614; Attachment and Entry.
DR   SignaLink; P55072; -.
DR   SIGNOR; P55072; -.
DR   BioGRID-ORCS; 7415; 799 hits in 966 CRISPR screens.
DR   ChiTaRS; VCP; human.
DR   EvolutionaryTrace; P55072; -.
DR   GeneWiki; Valosin-containing_protein; -.
DR   GenomeRNAi; 7415; -.
DR   Pharos; P55072; Tchem.
DR   PRO; PR:P55072; -.
DR   Proteomes; UP000005640; Chromosome 9.
DR   RNAct; P55072; protein.
DR   Bgee; ENSG00000165280; Expressed in stromal cell of endometrium and 243 other tissues.
DR   ExpressionAtlas; P55072; baseline and differential.
DR   Genevisible; P55072; HS.
DR   GO; GO:1904949; C:ATPase complex; IEA:Ensembl.
DR   GO; GO:0035578; C:azurophil granule lumen; TAS:Reactome.
DR   GO; GO:0005737; C:cytoplasm; IDA:UniProtKB.
DR   GO; GO:0010494; C:cytoplasmic stress granule; IDA:UniProtKB.
DR   GO; GO:0005829; C:cytosol; IDA:UniProtKB.
DR   GO; GO:0036513; C:Derlin-1 retrotranslocation complex; IDA:UniProtKB.
DR   GO; GO:0005783; C:endoplasmic reticulum; IDA:UniProtKB.
DR   GO; GO:0005789; C:endoplasmic reticulum membrane; IDA:UniProtKB.
DR   GO; GO:0070062; C:extracellular exosome; HDA:UniProtKB.
DR   GO; GO:0005576; C:extracellular region; TAS:Reactome.
DR   GO; GO:1904813; C:ficolin-1-rich granule lumen; TAS:Reactome.
DR   GO; GO:0098978; C:glutamatergic synapse; IEA:Ensembl.
DR   GO; GO:0043231; C:intracellular membrane-bounded organelle; ISS:UniProtKB.
DR   GO; GO:0005811; C:lipid droplet; IDA:MGI.
DR   GO; GO:0005654; C:nucleoplasm; IDA:HPA.
DR   GO; GO:0005634; C:nucleus; IDA:UniProtKB.
DR   GO; GO:0048471; C:perinuclear region of cytoplasm; IDA:ParkinsonsUK-UCL.
DR   GO; GO:0000502; C:proteasome complex; IDA:BHF-UCL.
DR   GO; GO:0032991; C:protein-containing complex; IDA:UniProtKB.
DR   GO; GO:0034774; C:secretory granule lumen; TAS:Reactome.
DR   GO; GO:0035861; C:site of double-strand break; IDA:UniProtKB.
DR   GO; GO:0034098; C:VCP-NPL4-UFD1 AAA ATPase complex; ISS:ParkinsonsUK-UCL.
DR   GO; GO:1990730; C:VCP-NSFL1C complex; ISS:ParkinsonsUK-UCL.
DR   GO; GO:0043531; F:ADP binding; IEA:Ensembl.
DR   GO; GO:0005524; F:ATP binding; IEA:UniProtKB-KW.
DR   GO; GO:0016887; F:ATPase activity; IMP:UniProtKB.
DR   GO; GO:1904288; F:BAT3 complex binding; IPI:ParkinsonsUK-UCL.
DR   GO; GO:0035800; F:deubiquitinase activator activity; IDA:ParkinsonsUK-UCL.
DR   GO; GO:0042802; F:identical protein binding; IPI:IntAct.
DR   GO; GO:0036435; F:K48-linked polyubiquitin modification-dependent protein binding; IEA:Ensembl.
DR   GO; GO:0008289; F:lipid binding; IEA:UniProtKB-KW.
DR   GO; GO:0042288; F:MHC class I protein binding; IEA:Ensembl.
DR   GO; GO:0031593; F:polyubiquitin modification-dependent protein binding; IDA:BHF-UCL.
DR   GO; GO:0019904; F:protein domain specific binding; IPI:UniProtKB.
DR   GO; GO:0019903; F:protein phosphatase binding; IPI:BHF-UCL.
DR   GO; GO:0003723; F:RNA binding; HDA:UniProtKB.
DR   GO; GO:0031625; F:ubiquitin protein ligase binding; IPI:ParkinsonsUK-UCL.
DR   GO; GO:0044389; F:ubiquitin-like protein ligase binding; IPI:ParkinsonsUK-UCL.
DR   GO; GO:1990381; F:ubiquitin-specific protease binding; IPI:ParkinsonsUK-UCL.
DR   GO; GO:0006919; P:activation of cysteine-type endopeptidase activity involved in apoptotic process; ISS:UniProtKB.
DR   GO; GO:0070842; P:aggresome assembly; IEA:Ensembl.
DR   GO; GO:0046034; P:ATP metabolic process; IEA:Ensembl.
DR   GO; GO:0097352; P:autophagosome maturation; IMP:UniProtKB.
DR   GO; GO:0006914; P:autophagy; IMP:UniProtKB.
DR   GO; GO:1903843; P:cellular response to arsenite ion; IMP:UniProtKB.
DR   GO; GO:0006974; P:cellular response to DNA damage stimulus; IDA:UniProtKB.
DR   GO; GO:0034605; P:cellular response to heat; IMP:UniProtKB.
DR   GO; GO:0006281; P:DNA repair; NAS:UniProtKB.
DR   GO; GO:0006302; P:double-strand break repair; IDA:UniProtKB.
DR   GO; GO:0061857; P:endoplasmic reticulum stress-induced pre-emptive quality control; IMP:UniProtKB.
DR   GO; GO:0006888; P:endoplasmic reticulum to Golgi vesicle-mediated transport; IEA:Ensembl.
DR   GO; GO:0030968; P:endoplasmic reticulum unfolded protein response; TAS:UniProtKB.
DR   GO; GO:0032510; P:endosome to lysosome transport via multivesicular body sorting pathway; IMP:UniProtKB.
DR   GO; GO:0071712; P:ER-associated misfolded protein catabolic process; IMP:ParkinsonsUK-UCL.
DR   GO; GO:0036503; P:ERAD pathway; IDA:ParkinsonsUK-UCL.
DR   GO; GO:0070987; P:error-free translesion synthesis; TAS:Reactome.
DR   GO; GO:0045184; P:establishment of protein localization; TAS:UniProtKB.
DR   GO; GO:0072389; P:flavin adenine dinucleotide catabolic process; IMP:ParkinsonsUK-UCL.
DR   GO; GO:0036297; P:interstrand cross-link repair; ISS:UniProtKB.
DR   GO; GO:0016236; P:macroautophagy; IMP:UniProtKB.
DR   GO; GO:0051228; P:mitotic spindle disassembly; IBA:GO_Central.
DR   GO; GO:0006734; P:NADH metabolic process; IMP:ParkinsonsUK-UCL.
DR   GO; GO:0045879; P:negative regulation of smoothened signaling pathway; IMP:FlyBase.
DR   GO; GO:0043312; P:neutrophil degranulation; TAS:Reactome.
DR   GO; GO:2001171; P:positive regulation of ATP biosynthetic process; IMP:ParkinsonsUK-UCL.
DR   GO; GO:0090263; P:positive regulation of canonical Wnt signaling pathway; IDA:FlyBase.
DR   GO; GO:1903007; P:positive regulation of Lys63-specific deubiquitinase activity; IDA:ParkinsonsUK-UCL.
DR   GO; GO:0010918; P:positive regulation of mitochondrial membrane potential; IMP:ParkinsonsUK-UCL.
DR   GO; GO:1903862; P:positive regulation of oxidative phosphorylation; IMP:ParkinsonsUK-UCL.
DR   GO; GO:0032436; P:positive regulation of proteasomal ubiquitin-dependent protein catabolic process; IDA:BHF-UCL.
DR   GO; GO:0045732; P:positive regulation of protein catabolic process; IDA:BHF-UCL.
DR   GO; GO:1903006; P:positive regulation of protein K63-linked deubiquitination; IDA:ParkinsonsUK-UCL.
DR   GO; GO:0031334; P:positive regulation of protein-containing complex assembly; IDA:BHF-UCL.
DR   GO; GO:0010498; P:proteasomal protein catabolic process; IMP:UniProtKB.
DR   GO; GO:0043161; P:proteasome-mediated ubiquitin-dependent protein catabolic process; IMP:UniProtKB.
DR   GO; GO:0016579; P:protein deubiquitination; TAS:Reactome.
DR   GO; GO:0006457; P:protein folding; TAS:Reactome.
DR   GO; GO:0016567; P:protein ubiquitination; IDA:UniProtKB.
DR   GO; GO:0106300; P:protein-DNA covalent cross-linking repair; IDA:UniProtKB.
DR   GO; GO:1903715; P:regulation of aerobic respiration; IMP:ParkinsonsUK-UCL.
DR   GO; GO:0042981; P:regulation of apoptotic process; TAS:UniProtKB.
DR   GO; GO:1905634; P:regulation of protein localization to chromatin; IDA:UniProtKB.
DR   GO; GO:0050807; P:regulation of synapse organization; IEA:Ensembl.
DR   GO; GO:0030970; P:retrograde protein transport, ER to cytosol; IDA:UniProtKB.
DR   GO; GO:0035617; P:stress granule disassembly; IMP:UniProtKB.
DR   GO; GO:0019985; P:translesion synthesis; IMP:UniProtKB.
DR   GO; GO:0055085; P:transmembrane transport; TAS:Reactome.
DR   GO; GO:0030433; P:ubiquitin-dependent ERAD pathway; IDA:UniProtKB.
DR   GO; GO:0019079; P:viral genome replication; IMP:CACAO.
DR   IDEAL; IID00201; -.
DR   InterPro; IPR003593; AAA+_ATPase.
DR   InterPro; IPR005938; AAA_ATPase_CDC48.
DR   InterPro; IPR041569; AAA_lid_3.
DR   InterPro; IPR009010; Asp_de-COase-like_dom_sf.
DR   InterPro; IPR003959; ATPase_AAA_core.
DR   InterPro; IPR003960; ATPase_AAA_CS.
DR   InterPro; IPR004201; Cdc48_dom2.
DR   InterPro; IPR029067; CDC48_domain_2-like_sf.
DR   InterPro; IPR003338; CDC4_N-term_subdom.
DR   InterPro; IPR027417; P-loop_NTPase.
DR   InterPro; IPR015415; Vps4_C.
DR   Pfam; PF00004; AAA; 2.
DR   Pfam; PF17862; AAA_lid_3; 2.
DR   Pfam; PF02933; CDC48_2; 1.
DR   Pfam; PF02359; CDC48_N; 1.
DR   Pfam; PF09336; Vps4_C; 1.
DR   SMART; SM00382; AAA; 2.
DR   SMART; SM01072; CDC48_2; 1.
DR   SMART; SM01073; CDC48_N; 1.
DR   SUPFAM; SSF50692; SSF50692; 1.
DR   SUPFAM; SSF52540; SSF52540; 2.
DR   SUPFAM; SSF54585; SSF54585; 1.
DR   TIGRFAMs; TIGR01243; CDC48; 1.
DR   PROSITE; PS00674; AAA; 2.
PE   1: Evidence at protein level;
KW   3D-structure; Acetylation; Amyotrophic lateral sclerosis; ATP-binding;
KW   Autophagy; Charcot-Marie-Tooth disease; Cytoplasm;
KW   Direct protein sequencing; Disease variant; DNA damage; DNA repair;
KW   Endoplasmic reticulum; Hydrolase; Isopeptide bond; Lipid-binding;
KW   Methylation; Neurodegeneration; Neuropathy; Nucleotide-binding; Nucleus;
KW   Phosphoprotein; Reference proteome; Transport; Ubl conjugation;
KW   Ubl conjugation pathway.
FT   INIT_MET        1
FT                   /note="Removed"
FT                   /evidence="ECO:0000269|PubMed:12665801, ECO:0000269|Ref.8,
FT                   ECO:0007744|PubMed:19369195, ECO:0007744|PubMed:19413330,
FT                   ECO:0007744|PubMed:21406692, ECO:0007744|PubMed:22223895,
FT                   ECO:0007744|PubMed:25944712"
FT   CHAIN           2..806
FT                   /note="Transitional endoplasmic reticulum ATPase"
FT                   /id="PRO_0000084572"
FT   NP_BIND         247..253
FT                   /note="ATP 1"
FT                   /evidence="ECO:0000269|PubMed:20512113"
FT   NP_BIND         521..526
FT                   /note="ATP 2"
FT                   /evidence="ECO:0000250|UniProtKB:Q01853"
FT   REGION          708..727
FT                   /note="Disordered"
FT                   /evidence="ECO:0000256|SAM:MobiDB-lite"
FT   REGION          768..806
FT                   /note="Disordered"
FT                   /evidence="ECO:0000256|SAM:MobiDB-lite"
FT   REGION          797..806
FT                   /note="Interaction with UBXN6"
FT                   /evidence="ECO:0000269|PubMed:18656546"
FT   MOTIF           802..806
FT                   /note="PIM motif"
FT                   /evidence="ECO:0000269|PubMed:24726327"
FT   COMPBIAS        768..794
FT                   /note="Polar residues"
FT                   /evidence="ECO:0000256|SAM:MobiDB-lite"
FT   BINDING         348
FT                   /note="ATP 1"
FT                   /evidence="ECO:0000269|PubMed:20512113"
FT   BINDING         384
FT                   /note="ATP 1"
FT                   /evidence="ECO:0000269|PubMed:20512113"
FT   MOD_RES         2
FT                   /note="N-acetylalanine"
FT                   /evidence="ECO:0000269|Ref.8, ECO:0007744|PubMed:19369195,
FT                   ECO:0007744|PubMed:19413330, ECO:0007744|PubMed:21406692,
FT                   ECO:0007744|PubMed:22223895, ECO:0007744|PubMed:25944712"
FT   MOD_RES         3
FT                   /note="Phosphoserine"
FT                   /evidence="ECO:0007744|PubMed:18691976,
FT                   ECO:0007744|PubMed:19369195, ECO:0007744|PubMed:21406692,
FT                   ECO:0007744|PubMed:23186163"
FT   MOD_RES         7
FT                   /note="Phosphoserine"
FT                   /evidence="ECO:0007744|PubMed:23186163"
FT   MOD_RES         13
FT                   /note="Phosphoserine"
FT                   /evidence="ECO:0007744|PubMed:23186163"
FT   MOD_RES         37
FT                   /note="Phosphoserine"
FT                   /evidence="ECO:0007744|PubMed:19369195"
FT   MOD_RES         315
FT                   /note="N6,N6,N6-trimethyllysine; by VCPKMT"
FT                   /evidence="ECO:0000269|PubMed:22948820,
FT                   ECO:0000269|PubMed:23349634"
FT   MOD_RES         436
FT                   /note="Phosphothreonine"
FT                   /evidence="ECO:0007744|PubMed:18691976"
FT   MOD_RES         462
FT                   /note="Phosphoserine"
FT                   /evidence="ECO:0007744|PubMed:23186163"
FT   MOD_RES         502
FT                   /note="N6-acetyllysine"
FT                   /evidence="ECO:0000250|UniProtKB:Q01853"
FT   MOD_RES         505
FT                   /note="N6-acetyllysine"
FT                   /evidence="ECO:0000250|UniProtKB:Q01853"
FT   MOD_RES         668
FT                   /note="N6-acetyllysine; alternate"
FT                   /evidence="ECO:0000250|UniProtKB:Q01853"
FT   MOD_RES         668
FT                   /note="N6-succinyllysine; alternate"
FT                   /evidence="ECO:0000250|UniProtKB:Q01853"
FT   MOD_RES         702
FT                   /note="Phosphoserine"
FT                   /evidence="ECO:0007744|PubMed:23186163"
FT   MOD_RES         754
FT                   /note="N6-acetyllysine"
FT                   /evidence="ECO:0000250|UniProtKB:Q01853"
FT   MOD_RES         770
FT                   /note="Phosphoserine"
FT                   /evidence="ECO:0007744|PubMed:19690332"
FT   MOD_RES         775
FT                   /note="Phosphoserine"
FT                   /evidence="ECO:0007744|PubMed:19690332"
FT   MOD_RES         787
FT                   /note="Phosphoserine"
FT                   /evidence="ECO:0007744|PubMed:18691976"
FT   MOD_RES         805
FT                   /note="Phosphotyrosine"
FT                   /evidence="ECO:0000250|UniProtKB:Q01853"
FT   CROSSLNK        8
FT                   /note="Glycyl lysine isopeptide (Lys-Gly) (interchain with
FT                   G-Cter in SUMO2)"
FT                   /evidence="ECO:0007744|PubMed:28112733"
FT   CROSSLNK        18
FT                   /note="Glycyl lysine isopeptide (Lys-Gly) (interchain with
FT                   G-Cter in SUMO2)"
FT                   /evidence="ECO:0007744|PubMed:28112733"
FT   VARIANT         95
FT                   /note="R -> G (in IBMPFD1; cultured cells expressing the
FT                   mutant protein show a marked general increase in the level
FT                   of ubiquitin-conjugated proteins and impaired protein
FT                   degradation through the endoplasmic reticulum-associated
FT                   degradation (ERAD) pathway; shows strongly reduced affinity
FT                   for ADP and increased affinity for ATP; abolishes
FT                   enhancement of K-315 methylation by ASPSCR1; decreased
FT                   interaction with CAV1 and UBXN6; dbSNP:rs121909332)"
FT                   /evidence="ECO:0000269|PubMed:15034582,
FT                   ECO:0000269|PubMed:16321991, ECO:0000269|PubMed:20512113,
FT                   ECO:0000269|PubMed:21822278"
FT                   /id="VAR_033016"
FT   VARIANT         97
FT                   /note="G -> E (in CMT2Y; increased ATPase activity;
FT                   dbSNP:rs864309502)"
FT                   /evidence="ECO:0000269|PubMed:25878907"
FT                   /id="VAR_076464"
FT   VARIANT         126
FT                   /note="I -> F (in IBMPFD1; unknown pathological
FT                   significance)"
FT                   /evidence="ECO:0000269|PubMed:27209344"
FT                   /id="VAR_076465"
FT   VARIANT         155
FT                   /note="R -> C (in IBMPFD1; also in one patient without
FT                   evidence of Paget disease of the bone; dbSNP:rs121909330)"
FT                   /evidence="ECO:0000269|PubMed:15034582,
FT                   ECO:0000269|PubMed:15732117"
FT                   /id="VAR_033017"
FT   VARIANT         155
FT                   /note="R -> H (in FTDALS6 and IBMPFD1; properly assembles
FT                   into a hexameric structure; cultured cells expressing the
FT                   mutant protein show a marked general increase in the level
FT                   of ubiquitin-conjugated proteins and impaired protein
FT                   degradation through the endoplasmic reticulum-associated
FT                   degradation (ERAD) pathway; shows strongly reduced affinity
FT                   for ADP and increased affinity for ATP; shows normal ATPase
FT                   activity according to PubMed:16321991 while according to
FT                   PubMed:25878907 and PubMed:25125609 shows increased ATPase
FT                   activity; no defect in ubiquitin-dependent protein
FT                   degradation by the proteasome; impaired autophagic
FT                   function; defective maturation of ubiquitin-containing
FT                   autophagosomes; decreased interaction with CAV1 and UBXN6;
FT                   decreased endosome to lysosome transport via multivesicular
FT                   body sorting pathway of CAV1; decreases the arsenite-
FT                   induced stress granules (SGs) clearance process;
FT                   dbSNP:rs121909329)"
FT                   /evidence="ECO:0000269|PubMed:15034582,
FT                   ECO:0000269|PubMed:16321991, ECO:0000269|PubMed:20104022,
FT                   ECO:0000269|PubMed:20512113, ECO:0000269|PubMed:21145000,
FT                   ECO:0000269|PubMed:21822278, ECO:0000269|PubMed:23349634,
FT                   ECO:0000269|PubMed:25125609, ECO:0000269|PubMed:25878907,
FT                   ECO:0000269|PubMed:27753622, ECO:0000269|PubMed:29804830"
FT                   /id="VAR_033018"
FT   VARIANT         155
FT                   /note="R -> L (in IBMPFD1; dbSNP:rs121909329)"
FT                   /evidence="ECO:0000269|PubMed:20335036"
FT                   /id="VAR_078910"
FT   VARIANT         155
FT                   /note="R -> P (in IBMPFD1; dbSNP:rs121909329)"
FT                   /evidence="ECO:0000269|PubMed:15034582"
FT                   /id="VAR_033019"
FT   VARIANT         155
FT                   /note="R -> S (in IBMPFD1; impaired autophagic function;
FT                   dbSNP:rs121909330)"
FT                   /evidence="ECO:0000269|PubMed:20104022"
FT                   /id="VAR_076466"
FT   VARIANT         159
FT                   /note="R -> G (in FTDALS6; dbSNP:rs387906789)"
FT                   /evidence="ECO:0000269|PubMed:21145000,
FT                   ECO:0000269|PubMed:23349634"
FT                   /id="VAR_065910"
FT   VARIANT         159
FT                   /note="R -> H (in IBMPFD1; without frontotemporal dementia;
FT                   abolishes enhancement of K-315 methylation by ASPSCR1;
FT                   dbSNP:rs121909335)"
FT                   /evidence="ECO:0000269|PubMed:16247064"
FT                   /id="VAR_033020"
FT   VARIANT         185
FT                   /note="E -> K (in CMT2Y; normal ATPase activity; impaired
FT                   autophagic function; dbSNP:rs864309501)"
FT                   /evidence="ECO:0000269|PubMed:25125609"
FT                   /id="VAR_076467"
FT   VARIANT         191
FT                   /note="R -> Q (in FTDALS6 and IBMPFD1; abolishes
FT                   enhancement of K-315 methylation by ASPSCR1;
FT                   dbSNP:rs121909334)"
FT                   /evidence="ECO:0000269|PubMed:15034582,
FT                   ECO:0000269|PubMed:21145000, ECO:0000269|PubMed:23349634"
FT                   /id="VAR_033021"
FT   VARIANT         198
FT                   /note="L -> W (in IBMPFD1; increased ATPase activity;
FT                   impaired autophagic function; dbSNP:rs748447593)"
FT                   /evidence="ECO:0000269|PubMed:17935506,
FT                   ECO:0000269|PubMed:20335036, ECO:0000269|PubMed:25878907,
FT                   ECO:0000269|PubMed:27753622"
FT                   /id="VAR_076468"
FT   VARIANT         232
FT                   /note="A -> E (in IBMPFD1; increased ATPase activity; no
FT                   defect in ubiquitin-dependent protein degradation by the
FT                   proteasome; impaired autophagic function; defect in
FT                   maturation of ubiquitin-containing autophagosomes;
FT                   decreased interaction with CAV1 and UBXN6;
FT                   dbSNP:rs121909331)"
FT                   /evidence="ECO:0000269|PubMed:15034582,
FT                   ECO:0000269|PubMed:20104022, ECO:0000269|PubMed:21822278,
FT                   ECO:0000269|PubMed:25125609, ECO:0000269|PubMed:25878907,
FT                   ECO:0000269|PubMed:27753622"
FT                   /id="VAR_033022"
FT   VARIANT         387
FT                   /note="N -> H (in IBMPFD1; unknown pathological
FT                   significance; dbSNP:rs1554668420)"
FT                   /evidence="ECO:0000269|PubMed:17935506"
FT                   /id="VAR_078911"
FT   VARIANT         592
FT                   /note="D -> N (in FTDALS6; dbSNP:rs387906790)"
FT                   /evidence="ECO:0000269|PubMed:21145000"
FT                   /id="VAR_065911"
FT   MUTAGEN         52..55
FT                   /note="FRGD->ARGA: Abolishes interaction with NPLOC4; when
FT                   associated with A-110."
FT                   /evidence="ECO:0000269|PubMed:26471729"
FT   MUTAGEN         53
FT                   /note="R->A: Minor effect on affinity for ATP and ADP."
FT                   /evidence="ECO:0000269|PubMed:20512113"
FT   MUTAGEN         86
FT                   /note="R->A: Strongly increased affinity for ATP. Strongly
FT                   reduced affinity for ADP."
FT                   /evidence="ECO:0000269|PubMed:20512113"
FT   MUTAGEN         110
FT                   /note="Y->A: Abolishes interaction with NPLOC4; when
FT                   associated with 52-A--A-55."
FT                   /evidence="ECO:0000269|PubMed:26471729"
FT   MUTAGEN         251
FT                   /note="K->Q: Impairs ERAD degradation of HMGCR and does not
FT                   inhibit interaction with RHBDD1; when associated with Q-
FT                   524."
FT                   /evidence="ECO:0000269|PubMed:16168377,
FT                   ECO:0000269|PubMed:22795130"
FT   MUTAGEN         305
FT                   /note="E->Q: Defect in ubiquitin-dependent protein
FT                   degradation by the proteasome; when associated with Q-578."
FT                   /evidence="ECO:0000269|PubMed:20104022,
FT                   ECO:0000269|PubMed:26471729"
FT   MUTAGEN         312
FT                   /note="K->A: Does not affect methylation by VCPKMT."
FT                   /evidence="ECO:0000269|PubMed:22948820"
FT   MUTAGEN         313
FT                   /note="R->A: Does not affect methylation by VCPKMT."
FT                   /evidence="ECO:0000269|PubMed:22948820"
FT   MUTAGEN         314
FT                   /note="E->A: Does not affect methylation by VCPKMT."
FT                   /evidence="ECO:0000269|PubMed:22948820"
FT   MUTAGEN         314
FT                   /note="Missing: Strongly impairs methylation by VCPKMT."
FT                   /evidence="ECO:0000269|PubMed:22948820"
FT   MUTAGEN         315
FT                   /note="K->L,Q,R: Abolishes methylation by VCPKMT."
FT                   /evidence="ECO:0000269|PubMed:22948820,
FT                   ECO:0000269|PubMed:23349634"
FT   MUTAGEN         316
FT                   /note="T->A: Does not affect methylation by VCPKMT."
FT                   /evidence="ECO:0000269|PubMed:22948820"
FT   MUTAGEN         317
FT                   /note="H->A: Does not affect methylation by VCPKMT."
FT                   /evidence="ECO:0000269|PubMed:22948820"
FT   MUTAGEN         318
FT                   /note="G->A: Does not affect methylation by VCPKMT."
FT                   /evidence="ECO:0000269|PubMed:22948820"
FT   MUTAGEN         524
FT                   /note="K->A: Impairs catalytic activity of RNF19A toward
FT                   SOD1 mutant. Does not inhibit interaction with RHBDD1; when
FT                   associated with A-251."
FT                   /evidence="ECO:0000269|PubMed:15456787,
FT                   ECO:0000269|PubMed:16168377, ECO:0000269|PubMed:22795130"
FT   MUTAGEN         524
FT                   /note="K->Q: Impairs ERAD degradation of HMGCR; when
FT                   associated with Q-251."
FT                   /evidence="ECO:0000269|PubMed:15456787,
FT                   ECO:0000269|PubMed:16168377, ECO:0000269|PubMed:22795130"
FT   MUTAGEN         578
FT                   /note="E->Q: Does not inhibit interaction with RHBDD1.
FT                   Increased interaction with CAV1 and UBXN6. Impaired
FT                   autophagic function. Defect in ubiquitin-dependent protein
FT                   degradation by the proteasome; when associated with Q-305.
FT                   Increases interaction with ZFAND1 in an arsenite-dependent
FT                   manner."
FT                   /evidence="ECO:0000269|PubMed:20104022,
FT                   ECO:0000269|PubMed:21822278, ECO:0000269|PubMed:22795130,
FT                   ECO:0000269|PubMed:26471729, ECO:0000269|PubMed:29804830"
FT   CONFLICT        169
FT                   /note="D -> H (in Ref. 6; AAI21795)"
FT                   /evidence="ECO:0000305"
FT   CONFLICT        312
FT                   /note="K -> I (in Ref. 3; BAG35235)"
FT                   /evidence="ECO:0000305"
FT   TURN            15..17
FT                   /evidence="ECO:0007829|PDB:4KLN"
FT   HELIX           22..24
FT                   /evidence="ECO:0007829|PDB:5IFW"
FT   STRAND          25..29
FT                   /evidence="ECO:0007829|PDB:5B6C"
FT   STRAND          38..41
FT                   /evidence="ECO:0007829|PDB:5B6C"
FT   HELIX           43..48
FT                   /evidence="ECO:0007829|PDB:5B6C"
FT   STRAND          53..55
FT                   /evidence="ECO:0007829|PDB:5FTN"
FT   STRAND          56..60
FT                   /evidence="ECO:0007829|PDB:5B6C"
FT   HELIX           62..64
FT                   /evidence="ECO:0007829|PDB:3QQ8"
FT   STRAND          66..73
FT                   /evidence="ECO:0007829|PDB:5B6C"
FT   STRAND          75..77
FT                   /evidence="ECO:0007829|PDB:3HU1"
FT   STRAND          81..83
FT                   /evidence="ECO:0007829|PDB:5B6C"
FT   HELIX           86..92
FT                   /evidence="ECO:0007829|PDB:5B6C"
FT   STRAND          93..95
FT                   /evidence="ECO:0007829|PDB:5FTN"
FT   STRAND          99..104
FT                   /evidence="ECO:0007829|PDB:5B6C"
FT   STRAND          112..119
FT                   /evidence="ECO:0007829|PDB:5B6C"
FT   HELIX           120..123
FT                   /evidence="ECO:0007829|PDB:5B6C"
FT   STRAND          126..128
FT                   /evidence="ECO:0007829|PDB:5IFS"
FT   HELIX           130..133
FT                   /evidence="ECO:0007829|PDB:5B6C"
FT   HELIX           135..139
FT                   /evidence="ECO:0007829|PDB:5B6C"
FT   TURN            140..142
FT                   /evidence="ECO:0007829|PDB:5B6C"
FT   STRAND          144..147
FT                   /evidence="ECO:0007829|PDB:5B6C"
FT   STRAND          151..154
FT                   /evidence="ECO:0007829|PDB:5B6C"
FT   STRAND          157..159
FT                   /evidence="ECO:0007829|PDB:4KDI"
FT   STRAND          161..176
FT                   /evidence="ECO:0007829|PDB:5B6C"
FT   STRAND          181..183
FT                   /evidence="ECO:0007829|PDB:5B6C"
FT   HELIX           191..193
FT                   /evidence="ECO:0007829|PDB:4KDL"
FT   STRAND          198..200
FT                   /evidence="ECO:0007829|PDB:5FTJ"
FT   HELIX           203..205
FT                   /evidence="ECO:0007829|PDB:4KO8"
FT   HELIX           210..219
FT                   /evidence="ECO:0007829|PDB:4KO8"
FT   HELIX           221..225
FT                   /evidence="ECO:0007829|PDB:4KO8"
FT   HELIX           227..233
FT                   /evidence="ECO:0007829|PDB:4KO8"
FT   STRAND          240..244
FT                   /evidence="ECO:0007829|PDB:4KO8"
FT   STRAND          246..250
FT                   /evidence="ECO:0007829|PDB:4KLN"
FT   HELIX           251..261
FT                   /evidence="ECO:0007829|PDB:4KO8"
FT   STRAND          263..270
FT                   /evidence="ECO:0007829|PDB:4KO8"
FT   HELIX           271..275
FT                   /evidence="ECO:0007829|PDB:4KO8"
FT   HELIX           281..295
FT                   /evidence="ECO:0007829|PDB:4KO8"
FT   STRAND          298..305
FT                   /evidence="ECO:0007829|PDB:4KO8"
FT   HELIX           306..308
FT                   /evidence="ECO:0007829|PDB:4KO8"
FT   STRAND          313..315
FT                   /evidence="ECO:0007829|PDB:5FTK"
FT   HELIX           319..333
FT                   /evidence="ECO:0007829|PDB:4KO8"
FT   HELIX           335..337
FT                   /evidence="ECO:0007829|PDB:5DYG"
FT   STRAND          341..348
FT                   /evidence="ECO:0007829|PDB:4KO8"
FT   HELIX           350..352
FT                   /evidence="ECO:0007829|PDB:4KO8"
FT   HELIX           355..358
FT                   /evidence="ECO:0007829|PDB:4KO8"
FT   TURN            360..362
FT                   /evidence="ECO:0007829|PDB:5FTL"
FT   STRAND          365..368
FT                   /evidence="ECO:0007829|PDB:4KO8"
FT   HELIX           374..385
FT                   /evidence="ECO:0007829|PDB:4KO8"
FT   STRAND          388..390
FT                   /evidence="ECO:0007829|PDB:5DYG"
FT   HELIX           396..401
FT                   /evidence="ECO:0007829|PDB:4KO8"
FT   TURN            403..405
FT                   /evidence="ECO:0007829|PDB:5FTJ"
FT   HELIX           408..424
FT                   /evidence="ECO:0007829|PDB:4KO8"
FT   TURN            425..429
FT                   /evidence="ECO:0007829|PDB:4KO8"
FT   STRAND          432..436
FT                   /evidence="ECO:0007829|PDB:3HU1"
FT   HELIX           439..444
FT                   /evidence="ECO:0007829|PDB:4KO8"
FT   HELIX           449..457
FT                   /evidence="ECO:0007829|PDB:4KO8"
FT   STRAND          458..461
FT                   /evidence="ECO:0007829|PDB:4KO8"
FT   TURN            462..468
FT                   /evidence="ECO:0007829|PDB:4KO8"
FT   HELIX           476..478
FT                   /evidence="ECO:0007829|PDB:6G2V"
FT   HELIX           483..498
FT                   /evidence="ECO:0007829|PDB:6G2V"
FT   HELIX           500..505
FT                   /evidence="ECO:0007829|PDB:6G2V"
FT   STRAND          513..519
FT                   /evidence="ECO:0007829|PDB:6G2V"
FT   HELIX           524..534
FT                   /evidence="ECO:0007829|PDB:6G2V"
FT   STRAND          538..543
FT                   /evidence="ECO:0007829|PDB:6G2V"
FT   HELIX           544..547
FT                   /evidence="ECO:0007829|PDB:6G2V"
FT   TURN            552..554
FT                   /evidence="ECO:0007829|PDB:5C1B"
FT   HELIX           557..568
FT                   /evidence="ECO:0007829|PDB:6G2V"
FT   STRAND          571..577
FT                   /evidence="ECO:0007829|PDB:6G2V"
FT   HELIX           579..581
FT                   /evidence="ECO:0007829|PDB:6G2V"
FT   TURN            584..586
FT                   /evidence="ECO:0007829|PDB:5FTN"
FT   STRAND          588..590
FT                   /evidence="ECO:0007829|PDB:5FTJ"
FT   HELIX           597..609
FT                   /evidence="ECO:0007829|PDB:6G2V"
FT   HELIX           613..615
FT                   /evidence="ECO:0007829|PDB:6G2V"
FT   STRAND          617..624
FT                   /evidence="ECO:0007829|PDB:6G2V"
FT   HELIX           626..628
FT                   /evidence="ECO:0007829|PDB:6G2V"
FT   HELIX           631..634
FT                   /evidence="ECO:0007829|PDB:6G2V"
FT   STRAND          635..639
FT                   /evidence="ECO:0007829|PDB:5FTK"
FT   STRAND          641..644
FT                   /evidence="ECO:0007829|PDB:6G2V"
FT   HELIX           650..661
FT                   /evidence="ECO:0007829|PDB:6G2V"
FT   STRAND          662..664
FT                   /evidence="ECO:0007829|PDB:5C1B"
FT   HELIX           672..677
FT                   /evidence="ECO:0007829|PDB:6G2V"
FT   TURN            678..681
FT                   /evidence="ECO:0007829|PDB:6G2V"
FT   HELIX           684..711
FT                   /evidence="ECO:0007829|PDB:6G2V"
FT   STRAND          722..724
FT                   /evidence="ECO:0007829|PDB:5IFW"
FT   HELIX           733..739
FT                   /evidence="ECO:0007829|PDB:6G2V"
FT   HELIX           740..742
FT                   /evidence="ECO:0007829|PDB:6G2V"
FT   HELIX           749..761
FT                   /evidence="ECO:0007829|PDB:6G2V"
FT   HELIX           762..766
FT                   /evidence="ECO:0007829|PDB:5C1B"
SQ   SEQUENCE   806 AA;  89322 MW;  501B721D3A77BA8A CRC64;
     MASGADSKGD DLSTAILKQK NRPNRLIVDE AINEDNSVVS LSQPKMDELQ LFRGDTVLLK
     GKKRREAVCI VLSDDTCSDE KIRMNRVVRN NLRVRLGDVI SIQPCPDVKY GKRIHVLPID
     DTVEGITGNL FEVYLKPYFL EAYRPIRKGD IFLVRGGMRA VEFKVVETDP SPYCIVAPDT
     VIHCEGEPIK REDEEESLNE VGYDDIGGCR KQLAQIKEMV ELPLRHPALF KAIGVKPPRG
     ILLYGPPGTG KTLIARAVAN ETGAFFFLIN GPEIMSKLAG ESESNLRKAF EEAEKNAPAI
     IFIDELDAIA PKREKTHGEV ERRIVSQLLT LMDGLKQRAH VIVMAATNRP NSIDPALRRF
     GRFDREVDIG IPDATGRLEI LQIHTKNMKL ADDVDLEQVA NETHGHVGAD LAALCSEAAL
     QAIRKKMDLI DLEDETIDAE VMNSLAVTMD DFRWALSQSN PSALRETVVE VPQVTWEDIG
     GLEDVKRELQ ELVQYPVEHP DKFLKFGMTP SKGVLFYGPP GCGKTLLAKA IANECQANFI
     SIKGPELLTM WFGESEANVR EIFDKARQAA PCVLFFDELD SIAKARGGNI GDGGGAADRV
     INQILTEMDG MSTKKNVFII GATNRPDIID PAILRPGRLD QLIYIPLPDE KSRVAILKAN
     LRKSPVAKDV DLEFLAKMTN GFSGADLTEI CQRACKLAIR ESIESEIRRE RERQTNPSAM
     EVEEDDPVPE IRRDHFEEAM RFARRSVSDN DIRKYEMFAQ TLQQSRGFGS FRFPSGNQGG
     AGPSQGSGGG TGGSVYTEDN DDDLYG
```

|  |
| --- |
| **Mascot:** http://www.matrixscience.com/ |
