# Supplementary material for: Chlorpromazine induces cytotoxic autophagy in glioblastoma cells via endoplasmic reticulum stress and unfolded protein response
Source: J Exp Clin Cancer Res. 2021 Nov 5;40:347. doi: 10.1186/s13046-021-02144-w (PMC8569984; doi:10.1186/s13046-021-02144-w)
Supplement: Supplementary file 1 — Additional file 1. [file 13046_2021_2144_MOESM1_ESM.zip › MASCOT IDs/Mascot Search Results_ TRAP1_HUMAN2.html]

Mascot Search Results: TRAP1\_HUMAN
 

# MASCOT Search Results

## Protein View: TRAP1\_HUMAN

### Heat shock protein 75 kDa, mitochondrial OS=Homo sapiens OX=9606 GN=TRAP1 PE=1 SV=3

|  |  |
| --- | --- |
| Database: | SwissProt |
| Score: | 87 |
| Monoisotopic mass (Mr): | 80345 |
| Calculated pI: | 8.30 |
| Taxonomy: | Homo sapiens |

Sequence similarity is available as an NCBI BLAST search of TRAP1\_HUMAN against nr.

### Search parameters

|  |  |
| --- | --- |
| MS data file: | `ppw_D10_157002889600.txt` |
| Enzyme: | Trypsin: cuts C-term side of KR unless next residue is P. |
| Fixed modifications: | Carbamidomethyl (C) |

### Protein sequence coverage: 1%

Matched peptides shown in ***bold red***.

|  |  |  |  |  |  |
| --- | --- | --- | --- | --- | --- |
| `1` | `MARELRALLL` | `WGRRLRPLLR` | `APALAAVPGG` | `KPILCPRRTT` | `AQLGPRRNPA` |
| `51` | `WSLQAGRLFS` | `TQTAEDKEEP` | `LHSIISSTES` | `VQGSTSKHEF` | `QAETKKLLDI` |
| `101` | `VARSLYSEKE` | `VFIRELISNA` | `SDALEKLRHK` | `LVSDGQALPE` | `MEIHLQTNAE` |
| `151` | `KGTITIQDTG` | `IGMTQEELVS` | `NLGTIARSGS` | `KAFLDALQNQ` | `AEASSKIIGQ` |
| `201` | `FGVGFYSAFM` | `VADRVEVYSR` | `SAAPGSLGYQ` | `WLSDGSGVFE` | `IAEASGVRTG` |
| `251` | `TKIIIHLKSD` | `CKEFSSEARV` | `RDVVTKYSNF` | `VSFPLYLNGR` | `RMNTLQAIWM` |
| `301` | `MDPKDVREWQ` | `HEEFYRYVAQ` | `AHDKPRYTLH` | `YKTDAPLNIR` | `SIFYVPDMKP` |
| `351` | `SMFDVSRELG` | `SSVALYSRKV` | `LIQTKATDIL` | `PKWLRFIRGV` | `VDSEDIPLNL` |
| `401` | `SRELLQESAL` | `IRKLRDVLQQ` | `RLIKFFIDQS` | `KKDAEKYAKF` | `FEDYGLFMRE` |
| `451` | `GIVTATEQEV` | `KEDIAKLLRY` | `ESSALPSGQL` | `TSLSEYASRM` | `RAGTRNIYYL` |
| `501` | `CAPNRHLAEH` | `SPYYEAMKKK` | `DTEVLFCFEQ` | `FDELTLLHLR` | `EFDKKKLISV` |
| `551` | `ETDIVVDHYK` | `EEKFEDRSPA` | `AECLSEKETE` | `ELMAWMRNVL` | `GSRVTNVKVT` |
| `601` | `LRLDTHPAMV` | `TVLEMGAARH` | `FLRMQQLAKT` | `QEERAQLLQP` | `TLEINPRHAL` |
| `651` | `IKKLNQLRAS` | `EPGLAQLLVD` | `QIYENAMIAA` | `GLVDDPRAMV` | `GRLNELLVKA` |
| `701` | `LERH` |  |  |  |  |

Unformatted sequence string: 704 residues (for pasting into other applications).

|  |  |  |  |
| --- | --- | --- | --- |
| Sort by | residue number | increasing mass | decreasing mass |
| Show | matched peptides only | predicted peptides also |  |

| Query | Start | – | End | Observed | Mr(expt) | Mr(calc) | ppm | M | Score | Expect | Rank | U | Peptide |
| --- | --- | --- | --- | --- | --- | --- | --- | --- | --- | --- | --- | --- | --- |
| 44 | 389 | – | 402 | 1513.8182 | 1512.8109 | 1512.7784 | 21.5 | 0 | 87 | 7.9e-08 | 1Score **> 28** indicates **identity** Score **> 26** indicates **homology** | U | R.GVVDSEDIPLNLSR.E |

---

```
ID   TRAP1_HUMAN             Reviewed;         704 AA.
AC   Q12931; B4DR68; D3DUC8; F5H897; O43642; O75235; Q9UHL5;
DT   15-JUL-1999, integrated into UniProtKB/Swiss-Prot.
DT   07-JUN-2005, sequence version 3.
DT   02-JUN-2021, entry version 209.
DE   RecName: Full=Heat shock protein 75 kDa, mitochondrial;
DE            Short=HSP 75;
DE   AltName: Full=TNFR-associated protein 1;
DE   AltName: Full=Tumor necrosis factor type 1 receptor-associated protein;
DE            Short=TRAP-1;
DE   Flags: Precursor;
GN   Name=TRAP1; Synonyms=HSP75;
OS   Homo sapiens (Human).
OC   Eukaryota; Metazoa; Chordata; Craniata; Vertebrata; Euteleostomi; Mammalia;
OC   Eutheria; Euarchontoglires; Primates; Haplorrhini; Catarrhini; Hominidae;
OC   Homo.
OX   NCBI_TaxID=9606;
RN   [1]
RP   NUCLEOTIDE SEQUENCE [MRNA], AND VARIANT GLY-307.
RX   PubMed=10545594; DOI=10.1093/hmg/8.12.2155;
RA   Simmons A.D., Musy M.M., Lopes C.S., Hwang L.-Y., Yang Y.-P., Lovett M.;
RT   "A direct interaction between EXT proteins and glycosyltransferases is
RT   defective in hereditary multiple exostoses.";
RL   Hum. Mol. Genet. 8:2155-2164(1999).
RN   [2]
RP   NUCLEOTIDE SEQUENCE [LARGE SCALE MRNA] (ISOFORM 2).
RX   PubMed=14702039; DOI=10.1038/ng1285;
RA   Ota T., Suzuki Y., Nishikawa T., Otsuki T., Sugiyama T., Irie R.,
RA   Wakamatsu A., Hayashi K., Sato H., Nagai K., Kimura K., Makita H.,
RA   Sekine M., Obayashi M., Nishi T., Shibahara T., Tanaka T., Ishii S.,
RA   Yamamoto J., Saito K., Kawai Y., Isono Y., Nakamura Y., Nagahari K.,
RA   Murakami K., Yasuda T., Iwayanagi T., Wagatsuma M., Shiratori A., Sudo H.,
RA   Hosoiri T., Kaku Y., Kodaira H., Kondo H., Sugawara M., Takahashi M.,
RA   Kanda K., Yokoi T., Furuya T., Kikkawa E., Omura Y., Abe K., Kamihara K.,
RA   Katsuta N., Sato K., Tanikawa M., Yamazaki M., Ninomiya K., Ishibashi T.,
RA   Yamashita H., Murakawa K., Fujimori K., Tanai H., Kimata M., Watanabe M.,
RA   Hiraoka S., Chiba Y., Ishida S., Ono Y., Takiguchi S., Watanabe S.,
RA   Yosida M., Hotuta T., Kusano J., Kanehori K., Takahashi-Fujii A., Hara H.,
RA   Tanase T.-O., Nomura Y., Togiya S., Komai F., Hara R., Takeuchi K.,
RA   Arita M., Imose N., Musashino K., Yuuki H., Oshima A., Sasaki N.,
RA   Aotsuka S., Yoshikawa Y., Matsunawa H., Ichihara T., Shiohata N., Sano S.,
RA   Moriya S., Momiyama H., Satoh N., Takami S., Terashima Y., Suzuki O.,
RA   Nakagawa S., Senoh A., Mizoguchi H., Goto Y., Shimizu F., Wakebe H.,
RA   Hishigaki H., Watanabe T., Sugiyama A., Takemoto M., Kawakami B.,
RA   Yamazaki M., Watanabe K., Kumagai A., Itakura S., Fukuzumi Y., Fujimori Y.,
RA   Komiyama M., Tashiro H., Tanigami A., Fujiwara T., Ono T., Yamada K.,
RA   Fujii Y., Ozaki K., Hirao M., Ohmori Y., Kawabata A., Hikiji T.,
RA   Kobatake N., Inagaki H., Ikema Y., Okamoto S., Okitani R., Kawakami T.,
RA   Noguchi S., Itoh T., Shigeta K., Senba T., Matsumura K., Nakajima Y.,
RA   Mizuno T., Morinaga M., Sasaki M., Togashi T., Oyama M., Hata H.,
RA   Watanabe M., Komatsu T., Mizushima-Sugano J., Satoh T., Shirai Y.,
RA   Takahashi Y., Nakagawa K., Okumura K., Nagase T., Nomura N., Kikuchi H.,
RA   Masuho Y., Yamashita R., Nakai K., Yada T., Nakamura Y., Ohara O.,
RA   Isogai T., Sugano S.;
RT   "Complete sequencing and characterization of 21,243 full-length human
RT   cDNAs.";
RL   Nat. Genet. 36:40-45(2004).
RN   [3]
RP   NUCLEOTIDE SEQUENCE [LARGE SCALE GENOMIC DNA].
RX   PubMed=15616553; DOI=10.1038/nature03187;
RA   Martin J., Han C., Gordon L.A., Terry A., Prabhakar S., She X., Xie G.,
RA   Hellsten U., Chan Y.M., Altherr M., Couronne O., Aerts A., Bajorek E.,
RA   Black S., Blumer H., Branscomb E., Brown N.C., Bruno W.J., Buckingham J.M.,
RA   Callen D.F., Campbell C.S., Campbell M.L., Campbell E.W., Caoile C.,
RA   Challacombe J.F., Chasteen L.A., Chertkov O., Chi H.C., Christensen M.,
RA   Clark L.M., Cohn J.D., Denys M., Detter J.C., Dickson M.,
RA   Dimitrijevic-Bussod M., Escobar J., Fawcett J.J., Flowers D., Fotopulos D.,
RA   Glavina T., Gomez M., Gonzales E., Goodstein D., Goodwin L.A., Grady D.L.,
RA   Grigoriev I., Groza M., Hammon N., Hawkins T., Haydu L., Hildebrand C.E.,
RA   Huang W., Israni S., Jett J., Jewett P.B., Kadner K., Kimball H.,
RA   Kobayashi A., Krawczyk M.-C., Leyba T., Longmire J.L., Lopez F., Lou Y.,
RA   Lowry S., Ludeman T., Manohar C.F., Mark G.A., McMurray K.L., Meincke L.J.,
RA   Morgan J., Moyzis R.K., Mundt M.O., Munk A.C., Nandkeshwar R.D.,
RA   Pitluck S., Pollard M., Predki P., Parson-Quintana B., Ramirez L., Rash S.,
RA   Retterer J., Ricke D.O., Robinson D.L., Rodriguez A., Salamov A.,
RA   Saunders E.H., Scott D., Shough T., Stallings R.L., Stalvey M.,
RA   Sutherland R.D., Tapia R., Tesmer J.G., Thayer N., Thompson L.S., Tice H.,
RA   Torney D.C., Tran-Gyamfi M., Tsai M., Ulanovsky L.E., Ustaszewska A.,
RA   Vo N., White P.S., Williams A.L., Wills P.L., Wu J.-R., Wu K., Yang J.,
RA   DeJong P., Bruce D., Doggett N.A., Deaven L., Schmutz J., Grimwood J.,
RA   Richardson P., Rokhsar D.S., Eichler E.E., Gilna P., Lucas S.M.,
RA   Myers R.M., Rubin E.M., Pennacchio L.A.;
RT   "The sequence and analysis of duplication-rich human chromosome 16.";
RL   Nature 432:988-994(2004).
RN   [4]
RP   NUCLEOTIDE SEQUENCE [LARGE SCALE GENOMIC DNA].
RA   Mural R.J., Istrail S., Sutton G.G., Florea L., Halpern A.L., Mobarry C.M.,
RA   Lippert R., Walenz B., Shatkay H., Dew I., Miller J.R., Flanigan M.J.,
RA   Edwards N.J., Bolanos R., Fasulo D., Halldorsson B.V., Hannenhalli S.,
RA   Turner R., Yooseph S., Lu F., Nusskern D.R., Shue B.C., Zheng X.H.,
RA   Zhong F., Delcher A.L., Huson D.H., Kravitz S.A., Mouchard L., Reinert K.,
RA   Remington K.A., Clark A.G., Waterman M.S., Eichler E.E., Adams M.D.,
RA   Hunkapiller M.W., Myers E.W., Venter J.C.;
RL   Submitted (SEP-2005) to the EMBL/GenBank/DDBJ databases.
RN   [5]
RP   NUCLEOTIDE SEQUENCE [LARGE SCALE MRNA].
RC   TISSUE=Brain, and Eye;
RX   PubMed=15489334; DOI=10.1101/gr.2596504;
RG   The MGC Project Team;
RT   "The status, quality, and expansion of the NIH full-length cDNA project:
RT   the Mammalian Gene Collection (MGC).";
RL   Genome Res. 14:2121-2127(2004).
RN   [6]
RP   NUCLEOTIDE SEQUENCE [MRNA] OF 16-704, AND VARIANT GLY-307.
RX   PubMed=7876093; DOI=10.1074/jbc.270.28.16630;
RA   Song H.Y., Dunbar J.D., Zhang Y.X., Guo D., Donner D.B.;
RT   "Identification of a protein with homology to hsp90 that binds the type 1
RT   tumor necrosis factor receptor.";
RL   J. Biol. Chem. 270:3574-3581(1995).
RN   [7]
RP   NUCLEOTIDE SEQUENCE [MRNA] OF 53-704, AND VARIANT GLU-395.
RX   PubMed=8756626; DOI=10.1128/mcb.16.9.4691;
RA   Chen C.-F., Chen Y., Dai K., Chen P.-L., Riley D.J., Lee W.-H.;
RT   "A new member of the hsp90 family of molecular chaperones interacts with
RT   the retinoblastoma protein during mitosis and after heat shock.";
RL   Mol. Cell. Biol. 16:4691-4699(1996).
RN   [8]
RP   CHARACTERIZATION.
RX   PubMed=10652318; DOI=10.1074/jbc.275.5.3305;
RA   Felts S.J., Owen B.A.L., Nguyen P., Trepel J., Donner D.B., Toft D.O.;
RT   "The hsp90-related protein TRAP1 is a mitochondrial protein with distinct
RT   functional properties.";
RL   J. Biol. Chem. 275:3305-3312(2000).
RN   [9]
RP   PHOSPHORYLATION [LARGE SCALE ANALYSIS] AT THR-494, AND IDENTIFICATION BY
RP   MASS SPECTROMETRY [LARGE SCALE ANALYSIS].
RC   TISSUE=Cervix carcinoma;
RX   PubMed=18669648; DOI=10.1073/pnas.0805139105;
RA   Dephoure N., Zhou C., Villen J., Beausoleil S.A., Bakalarski C.E.,
RA   Elledge S.J., Gygi S.P.;
RT   "A quantitative atlas of mitotic phosphorylation.";
RL   Proc. Natl. Acad. Sci. U.S.A. 105:10762-10767(2008).
RN   [10]
RP   ACETYLATION [LARGE SCALE ANALYSIS] AT LYS-332; LYS-424 AND LYS-466, AND
RP   IDENTIFICATION BY MASS SPECTROMETRY [LARGE SCALE ANALYSIS].
RX   PubMed=19608861; DOI=10.1126/science.1175371;
RA   Choudhary C., Kumar C., Gnad F., Nielsen M.L., Rehman M., Walther T.C.,
RA   Olsen J.V., Mann M.;
RT   "Lysine acetylation targets protein complexes and co-regulates major
RT   cellular functions.";
RL   Science 325:834-840(2009).
RN   [11]
RP   IDENTIFICATION BY MASS SPECTROMETRY [LARGE SCALE ANALYSIS].
RX   PubMed=21269460; DOI=10.1186/1752-0509-5-17;
RA   Burkard T.R., Planyavsky M., Kaupe I., Breitwieser F.P., Buerckstuemmer T.,
RA   Bennett K.L., Superti-Furga G., Colinge J.;
RT   "Initial characterization of the human central proteome.";
RL   BMC Syst. Biol. 5:17-17(2011).
RN   [12]
RP   FUNCTION AS NEGATIVE REGULATOR OF MITOCHONDRIAL RESPIRATION, AND
RP   INTERACTION WITH SDHA.
RX   PubMed=23747254; DOI=10.1016/j.cmet.2013.04.019;
RA   Sciacovelli M., Guzzo G., Morello V., Frezza C., Zheng L., Nannini N.,
RA   Calabrese F., Laudiero G., Esposito F., Landriscina M., Defilippi P.,
RA   Bernardi P., Rasola A.;
RT   "The mitochondrial chaperone TRAP1 promotes neoplastic growth by inhibiting
RT   succinate dehydrogenase.";
RL   Cell Metab. 17:988-999(2013).
RN   [13]
RP   FUNCTION.
RX   PubMed=23525905; DOI=10.1093/hmg/ddt132;
RA   Zhang L., Karsten P., Hamm S., Pogson J.H., Muller-Rischart A.K., Exner N.,
RA   Haass C., Whitworth A.J., Winklhofer K.F., Schulz J.B., Voigt A.;
RT   "TRAP1 rescues PINK1 loss-of-function phenotypes.";
RL   Hum. Mol. Genet. 22:2829-2841(2013).
RN   [14]
RP   PHOSPHORYLATION [LARGE SCALE ANALYSIS] AT SER-194, AND IDENTIFICATION BY
RP   MASS SPECTROMETRY [LARGE SCALE ANALYSIS].
RC   TISSUE=Cervix carcinoma, and Erythroleukemia;
RX   PubMed=23186163; DOI=10.1021/pr300630k;
RA   Zhou H., Di Palma S., Preisinger C., Peng M., Polat A.N., Heck A.J.,
RA   Mohammed S.;
RT   "Toward a comprehensive characterization of a human cancer cell
RT   phosphoproteome.";
RL   J. Proteome Res. 12:260-271(2013).
RN   [15]
RP   FUNCTION AS NEGATIVE REGULATOR OF MITOCHONDRIAL RESPIRATION, INTERACTION
RP   WITH SRC, TISSUE SPECIFICITY, AND SUBCELLULAR LOCATION.
RX   PubMed=23564345; DOI=10.1073/pnas.1220659110;
RA   Yoshida S., Tsutsumi S., Muhlebach G., Sourbier C., Lee M.J., Lee S.,
RA   Vartholomaiou E., Tatokoro M., Beebe K., Miyajima N., Mohney R.P., Chen Y.,
RA   Hasumi H., Xu W., Fukushima H., Nakamura K., Koga F., Kihara K., Trepel J.,
RA   Picard D., Neckers L.;
RT   "Molecular chaperone TRAP1 regulates a metabolic switch between
RT   mitochondrial respiration and aerobic glycolysis.";
RL   Proc. Natl. Acad. Sci. U.S.A. 110:E1604-E1612(2013).
RN   [16]
RP   PHOSPHORYLATION [LARGE SCALE ANALYSIS] AT SER-568, AND IDENTIFICATION BY
RP   MASS SPECTROMETRY [LARGE SCALE ANALYSIS].
RC   TISSUE=Liver;
RX   PubMed=24275569; DOI=10.1016/j.jprot.2013.11.014;
RA   Bian Y., Song C., Cheng K., Dong M., Wang F., Huang J., Sun D., Wang L.,
RA   Ye M., Zou H.;
RT   "An enzyme assisted RP-RPLC approach for in-depth analysis of human liver
RT   phosphoproteome.";
RL   J. Proteomics 96:253-262(2014).
RN   [17]
RP   IDENTIFICATION BY MASS SPECTROMETRY [LARGE SCALE ANALYSIS].
RX   PubMed=25944712; DOI=10.1002/pmic.201400617;
RA   Vaca Jacome A.S., Rabilloud T., Schaeffer-Reiss C., Rompais M., Ayoub D.,
RA   Lane L., Bairoch A., Van Dorsselaer A., Carapito C.;
RT   "N-terminome analysis of the human mitochondrial proteome.";
RL   Proteomics 15:2519-2524(2015).
CC   -!- FUNCTION: Chaperone that expresses an ATPase activity. Involved in
CC       maintaining mitochondrial function and polarization, downstream of
CC       PINK1 and mitochondrial complex I. Is a negative regulator of
CC       mitochondrial respiration able to modulate the balance between
CC       oxidative phosphorylation and aerobic glycolysis. The impact of TRAP1
CC       on mitochondrial respiration is probably mediated by modulation of
CC       mitochondrial SRC and inhibition of SDHA. {ECO:0000269|PubMed:23525905,
CC       ECO:0000269|PubMed:23564345, ECO:0000269|PubMed:23747254}.
CC   -!- SUBUNIT: Binds to the intracellular domain of tumor necrosis factor
CC       type 1 receptor. Binds to RB1. Interacts with SRC. Interacts with SDHA.
CC       {ECO:0000269|PubMed:23564345, ECO:0000269|PubMed:23747254}.
CC   -!- INTERACTION:
CC       Q12931; Q99714: HSD17B10; NbExp=3; IntAct=EBI-1055869, EBI-79964;
CC       Q12931; Q9BXM7-1: PINK1; NbExp=4; IntAct=EBI-1055869, EBI-15643376;
CC   -!- SUBCELLULAR LOCATION: Mitochondrion {ECO:0000269|PubMed:23564345}.
CC       Mitochondrion inner membrane {ECO:0000269|PubMed:23564345}.
CC       Mitochondrion matrix {ECO:0000269|PubMed:23564345}.
CC   -!- ALTERNATIVE PRODUCTS:
CC       Event=Alternative splicing; Named isoforms=2;
CC       Name=1;
CC         IsoId=Q12931-1; Sequence=Displayed;
CC       Name=2;
CC         IsoId=Q12931-2; Sequence=VSP_055061;
CC   -!- TISSUE SPECIFICITY: Found in skeletal muscle, liver, heart, brain,
CC       kidney, pancreas, lung, placenta and bladder. Expression is highly
CC       reduced in bladder cancer and renal cell carcinoma specimens compared
CC       to healthy tissues, but it is increased in other type of tumors.
CC       {ECO:0000269|PubMed:23564345}.
CC   -!- SIMILARITY: Belongs to the heat shock protein 90 family. {ECO:0000305}.
CC   -!- SEQUENCE CAUTION:
CC       Sequence=AAA87704.1; Type=Frameshift; Evidence={ECO:0000305};
CC   -!- WEB RESOURCE: Name=Atlas of Genetics and Cytogenetics in Oncology and
CC       Haematology;
CC       URL="http://atlasgeneticsoncology.org/Genes/TRAP1ID42692ch16p13.html";
CC   ---------------------------------------------------------------------------
CC   Copyrighted by the UniProt Consortium, see https://www.uniprot.org/terms
CC   Distributed under the Creative Commons Attribution (CC BY 4.0) License
CC   ---------------------------------------------------------------------------
DR   EMBL; AF154108; AAF15314.1; -; mRNA.
DR   EMBL; AK299127; BAG61180.1; -; mRNA.
DR   EMBL; AC005203; AAC24722.1; -; Genomic_DNA.
DR   EMBL; AC006111; -; NOT_ANNOTATED_CDS; Genomic_DNA.
DR   EMBL; CH471112; EAW85338.1; -; Genomic_DNA.
DR   EMBL; CH471112; EAW85340.1; -; Genomic_DNA.
DR   EMBL; BC018950; AAH18950.1; -; mRNA.
DR   EMBL; BC023585; AAH23585.1; -; mRNA.
DR   EMBL; U12595; AAA87704.1; ALT_FRAME; mRNA.
DR   EMBL; AF043254; AAC02679.1; -; mRNA.
DR   CCDS; CCDS10508.1; -. [Q12931-1]
DR   CCDS; CCDS61824.1; -. [Q12931-2]
DR   RefSeq; NP_001258978.1; NM_001272049.1. [Q12931-2]
DR   RefSeq; NP_057376.2; NM_016292.2. [Q12931-1]
DR   PDB; 4Z1F; X-ray; 2.70 A; A=60-561.
DR   PDB; 4Z1G; X-ray; 3.10 A; A=60-561.
DR   PDB; 4Z1H; X-ray; 2.90 A; A=60-561.
DR   PDB; 4Z1I; X-ray; 3.30 A; A/B/C/D=60-561.
DR   PDB; 5F3K; X-ray; 1.82 A; A/B=60-294.
DR   PDB; 5F5R; X-ray; 1.85 A; A/B=60-294.
DR   PDB; 5HPH; X-ray; 2.43 A; A/B=60-554.
DR   PDB; 5Y3N; X-ray; 2.40 A; A=60-561.
DR   PDB; 5Y3O; X-ray; 2.70 A; A=60-561.
DR   PDB; 6XG6; EM; 3.20 A; A/B=60-704.
DR   PDB; 7C04; X-ray; 1.70 A; A=60-294.
DR   PDB; 7C05; X-ray; 2.59 A; A=60-561.
DR   PDBsum; 4Z1F; -.
DR   PDBsum; 4Z1G; -.
DR   PDBsum; 4Z1H; -.
DR   PDBsum; 4Z1I; -.
DR   PDBsum; 5F3K; -.
DR   PDBsum; 5F5R; -.
DR   PDBsum; 5HPH; -.
DR   PDBsum; 5Y3N; -.
DR   PDBsum; 5Y3O; -.
DR   PDBsum; 6XG6; -.
DR   PDBsum; 7C04; -.
DR   PDBsum; 7C05; -.
DR   BMRB; Q12931; -.
DR   SMR; Q12931; -.
DR   BioGRID; 115435; 193.
DR   CORUM; Q12931; -.
DR   DIP; DIP-6250N; -.
DR   IntAct; Q12931; 75.
DR   MINT; Q12931; -.
DR   STRING; 9606.ENSP00000246957; -.
DR   BindingDB; Q12931; -.
DR   ChEMBL; CHEMBL1075132; -.
DR   DrugCentral; Q12931; -.
DR   GuidetoPHARMACOLOGY; 2909; -.
DR   iPTMnet; Q12931; -.
DR   MetOSite; Q12931; -.
DR   PhosphoSitePlus; Q12931; -.
DR   SwissPalm; Q12931; -.
DR   BioMuta; TRAP1; -.
DR   DMDM; 67477458; -.
DR   REPRODUCTION-2DPAGE; IPI00030275; -.
DR   CPTAC; CPTAC-288; -.
DR   CPTAC; CPTAC-289; -.
DR   EPD; Q12931; -.
DR   jPOST; Q12931; -.
DR   MassIVE; Q12931; -.
DR   MaxQB; Q12931; -.
DR   PaxDb; Q12931; -.
DR   PeptideAtlas; Q12931; -.
DR   PRIDE; Q12931; -.
DR   ProteomicsDB; 27722; -.
DR   ProteomicsDB; 59034; -. [Q12931-1]
DR   Antibodypedia; 3798; 702 antibodies.
DR   DNASU; 10131; -.
DR   Ensembl; ENST00000246957; ENSP00000246957; ENSG00000126602. [Q12931-1]
DR   Ensembl; ENST00000538171; ENSP00000442070; ENSG00000126602. [Q12931-2]
DR   GeneID; 10131; -.
DR   KEGG; hsa:10131; -.
DR   UCSC; uc002cvt.4; human. [Q12931-1]
DR   CTD; 10131; -.
DR   DisGeNET; 10131; -.
DR   GeneCards; TRAP1; -.
DR   HGNC; HGNC:16264; TRAP1.
DR   HPA; ENSG00000126602; Low tissue specificity.
DR   MIM; 606219; gene.
DR   neXtProt; NX_Q12931; -.
DR   OpenTargets; ENSG00000126602; -.
DR   PharmGKB; PA36781; -.
DR   VEuPathDB; HostDB:ENSG00000126602.10; -.
DR   eggNOG; KOG0019; Eukaryota.
DR   GeneTree; ENSGT01020000230401; -.
DR   HOGENOM; CLU_006684_3_1_1; -.
DR   InParanoid; Q12931; -.
DR   OMA; VITVEEM; -.
DR   PhylomeDB; Q12931; -.
DR   TreeFam; TF315234; -.
DR   PathwayCommons; Q12931; -.
DR   Reactome; R-HSA-611105; Respiratory electron transport.
DR   BioGRID-ORCS; 10131; 3 hits in 994 CRISPR screens.
DR   ChiTaRS; TRAP1; human.
DR   GeneWiki; TRAP1; -.
DR   GenomeRNAi; 10131; -.
DR   Pharos; Q12931; Tchem.
DR   PRO; PR:Q12931; -.
DR   Proteomes; UP000005640; Chromosome 16.
DR   RNAct; Q12931; protein.
DR   Bgee; ENSG00000126602; Expressed in heart left ventricle and 243 other tissues.
DR   ExpressionAtlas; Q12931; baseline and differential.
DR   Genevisible; Q12931; HS.
DR   GO; GO:0016020; C:membrane; HDA:UniProtKB.
DR   GO; GO:0005743; C:mitochondrial inner membrane; IDA:UniProtKB.
DR   GO; GO:0005758; C:mitochondrial intermembrane space; ISS:ParkinsonsUK-UCL.
DR   GO; GO:0005759; C:mitochondrial matrix; IDA:UniProtKB.
DR   GO; GO:0005739; C:mitochondrion; IDA:ParkinsonsUK-UCL.
DR   GO; GO:0005654; C:nucleoplasm; IDA:HPA.
DR   GO; GO:0005524; F:ATP binding; IEA:UniProtKB-KW.
DR   GO; GO:0016887; F:ATPase activity; IEA:InterPro.
DR   GO; GO:0019901; F:protein kinase binding; IPI:ParkinsonsUK-UCL.
DR   GO; GO:0003723; F:RNA binding; HDA:UniProtKB.
DR   GO; GO:0005164; F:tumor necrosis factor receptor binding; NAS:UniProtKB.
DR   GO; GO:0051082; F:unfolded protein binding; IBA:GO_Central.
DR   GO; GO:0061077; P:chaperone-mediated protein folding; TAS:ParkinsonsUK-UCL.
DR   GO; GO:1901856; P:negative regulation of cellular respiration; IMP:UniProtKB.
DR   GO; GO:1903751; P:negative regulation of intrinsic apoptotic signaling pathway in response to hydrogen peroxide; ISS:ParkinsonsUK-UCL.
DR   GO; GO:1903427; P:negative regulation of reactive oxygen species biosynthetic process; TAS:ParkinsonsUK-UCL.
DR   GO; GO:0006457; P:protein folding; IBA:GO_Central.
DR   GO; GO:0009386; P:translational attenuation; IMP:CACAO.
DR   Gene3D; 1.20.120.790; -; 1.
DR   Gene3D; 3.30.565.10; -; 1.
DR   HAMAP; MF_00505; HSP90; 1.
DR   InterPro; IPR003594; HATPase_C.
DR   InterPro; IPR036890; HATPase_C_sf.
DR   InterPro; IPR037196; HSP90_C.
DR   InterPro; IPR001404; Hsp90_fam.
DR   InterPro; IPR020575; Hsp90_N.
DR   InterPro; IPR020568; Ribosomal_S5_D2-typ_fold.
DR   PANTHER; PTHR11528; PTHR11528; 1.
DR   Pfam; PF00183; HSP90; 1.
DR   PIRSF; PIRSF002583; Hsp90; 1.
DR   PRINTS; PR00775; HEATSHOCK90.
DR   SMART; SM00387; HATPase_c; 1.
DR   SUPFAM; SSF110942; SSF110942; 1.
DR   SUPFAM; SSF54211; SSF54211; 1.
DR   SUPFAM; SSF55874; SSF55874; 1.
PE   1: Evidence at protein level;
KW   3D-structure; Acetylation; Alternative splicing; ATP-binding; Chaperone;
KW   Membrane; Mitochondrion; Mitochondrion inner membrane; Nucleotide-binding;
KW   Phosphoprotein; Reference proteome; Transit peptide.
FT   TRANSIT         1..59
FT                   /note="Mitochondrion"
FT                   /evidence="ECO:0000250"
FT   CHAIN           60..704
FT                   /note="Heat shock protein 75 kDa, mitochondrial"
FT                   /id="PRO_0000013604"
FT   BINDING         119
FT                   /note="ATP"
FT                   /evidence="ECO:0000250"
FT   BINDING         158
FT                   /note="ATP"
FT                   /evidence="ECO:0000250"
FT   BINDING         171
FT                   /note="ATP"
FT                   /evidence="ECO:0000250"
FT   BINDING         205
FT                   /note="ATP; via amide nitrogen"
FT                   /evidence="ECO:0000250"
FT   BINDING         402
FT                   /note="ATP"
FT                   /evidence="ECO:0000250"
FT   MOD_RES         170
FT                   /note="Phosphoserine"
FT                   /evidence="ECO:0000250|UniProtKB:Q5XHZ0"
FT   MOD_RES         174
FT                   /note="Phosphothreonine"
FT                   /evidence="ECO:0000250|UniProtKB:Q5XHZ0"
FT   MOD_RES         194
FT                   /note="Phosphoserine"
FT                   /evidence="ECO:0007744|PubMed:23186163"
FT   MOD_RES         262
FT                   /note="N6-acetyllysine"
FT                   /evidence="ECO:0000250|UniProtKB:Q9CQN1"
FT   MOD_RES         324
FT                   /note="N6-acetyllysine"
FT                   /evidence="ECO:0000250|UniProtKB:Q9CQN1"
FT   MOD_RES         332
FT                   /note="N6-acetyllysine"
FT                   /evidence="ECO:0007744|PubMed:19608861"
FT   MOD_RES         424
FT                   /note="N6-acetyllysine"
FT                   /evidence="ECO:0007744|PubMed:19608861"
FT   MOD_RES         431
FT                   /note="N6-acetyllysine"
FT                   /evidence="ECO:0000250|UniProtKB:Q9CQN1"
FT   MOD_RES         466
FT                   /note="N6-acetyllysine"
FT                   /evidence="ECO:0007744|PubMed:19608861"
FT   MOD_RES         494
FT                   /note="Phosphothreonine"
FT                   /evidence="ECO:0007744|PubMed:18669648"
FT   MOD_RES         568
FT                   /note="Phosphoserine"
FT                   /evidence="ECO:0007744|PubMed:24275569"
FT   VAR_SEQ         30..82
FT                   /note="Missing (in isoform 2)"
FT                   /evidence="ECO:0000303|PubMed:14702039"
FT                   /id="VSP_055061"
FT   VARIANT         307
FT                   /note="R -> G (in dbSNP:rs13926)"
FT                   /evidence="ECO:0000269|PubMed:10545594,
FT                   ECO:0000269|PubMed:7876093"
FT                   /id="VAR_016108"
FT   VARIANT         395
FT                   /note="D -> E (in dbSNP:rs1136948)"
FT                   /evidence="ECO:0000269|PubMed:8756626"
FT                   /id="VAR_049625"
FT   VARIANT         572
FT                   /note="E -> K (in dbSNP:rs55766649)"
FT                   /id="VAR_061272"
FT   VARIANT         692
FT                   /note="R -> H (in dbSNP:rs2791)"
FT                   /id="VAR_049626"
FT   CONFLICT        17..19
FT                   /note="PLL -> ALR (in Ref. 6; AAA87704)"
FT                   /evidence="ECO:0000305"
FT   CONFLICT        53
FT                   /note="L -> M (in Ref. 7; AAC02679)"
FT                   /evidence="ECO:0000305"
FT   CONFLICT        360
FT                   /note="G -> D (in Ref. 2; BAG61180)"
FT                   /evidence="ECO:0000305"
FT   CONFLICT        475..476
FT                   /note="Missing (in Ref. 7; AAC02679)"
FT                   /evidence="ECO:0000305"
FT   CONFLICT        488..491
FT                   /note="SRMR -> AHW (in Ref. 7; AAC02679)"
FT                   /evidence="ECO:0000305"
FT   STRAND          86..89
FT                   /evidence="ECO:0007829|PDB:7C04"
FT   HELIX           94..105
FT                   /evidence="ECO:0007829|PDB:7C04"
FT   HELIX           106..110
FT                   /evidence="ECO:0007829|PDB:7C04"
FT   HELIX           111..134
FT                   /evidence="ECO:0007829|PDB:7C04"
FT   STRAND          143..148
FT                   /evidence="ECO:0007829|PDB:7C04"
FT   TURN            149..152
FT                   /evidence="ECO:0007829|PDB:7C04"
FT   STRAND          153..158
FT                   /evidence="ECO:0007829|PDB:7C04"
FT   HELIX           165..170
FT                   /evidence="ECO:0007829|PDB:7C04"
FT   HELIX           171..173
FT                   /evidence="ECO:0007829|PDB:7C04"
FT   HELIX           174..178
FT                   /evidence="ECO:0007829|PDB:7C04"
FT   HELIX           181..189
FT                   /evidence="ECO:0007829|PDB:7C04"
FT   HELIX           194..200
FT                   /evidence="ECO:0007829|PDB:7C04"
FT   HELIX           203..205
FT                   /evidence="ECO:0007829|PDB:5F3K"
FT   HELIX           206..210
FT                   /evidence="ECO:0007829|PDB:7C04"
FT   STRAND          212..220
FT                   /evidence="ECO:0007829|PDB:7C04"
FT   STRAND          222..225
FT                   /evidence="ECO:0007829|PDB:5HPH"
FT   STRAND          229..233
FT                   /evidence="ECO:0007829|PDB:7C04"
FT   STRAND          235..246
FT                   /evidence="ECO:0007829|PDB:7C04"
FT   STRAND          249..257
FT                   /evidence="ECO:0007829|PDB:7C04"
FT   HELIX           262..265
FT                   /evidence="ECO:0007829|PDB:7C04"
FT   HELIX           267..274
FT                   /evidence="ECO:0007829|PDB:7C04"
FT   TURN            275..277
FT                   /evidence="ECO:0007829|PDB:7C04"
FT   HELIX           278..280
FT                   /evidence="ECO:0007829|PDB:7C04"
FT   STRAND          285..287
FT                   /evidence="ECO:0007829|PDB:7C04"
FT   TURN            298..300
FT                   /evidence="ECO:0007829|PDB:5HPH"
FT   TURN            303..305
FT                   /evidence="ECO:0007829|PDB:5Y3N"
FT   HELIX           308..319
FT                   /evidence="ECO:0007829|PDB:5Y3N"
FT   STRAND          325..337
FT                   /evidence="ECO:0007829|PDB:5Y3N"
FT   STRAND          339..348
FT                   /evidence="ECO:0007829|PDB:5Y3N"
FT   HELIX           352..356
FT                   /evidence="ECO:0007829|PDB:6XG6"
FT   STRAND          364..367
FT                   /evidence="ECO:0007829|PDB:5Y3N"
FT   STRAND          370..374
FT                   /evidence="ECO:0007829|PDB:5Y3N"
FT   HELIX           377..379
FT                   /evidence="ECO:0007829|PDB:7C05"
FT   HELIX           382..384
FT                   /evidence="ECO:0007829|PDB:5Y3N"
FT   STRAND          388..392
FT                   /evidence="ECO:0007829|PDB:5Y3N"
FT   STRAND          402..405
FT                   /evidence="ECO:0007829|PDB:5HPH"
FT   HELIX           411..432
FT                   /evidence="ECO:0007829|PDB:5Y3N"
FT   HELIX           435..454
FT                   /evidence="ECO:0007829|PDB:5Y3N"
FT   HELIX           458..465
FT                   /evidence="ECO:0007829|PDB:5Y3N"
FT   STRAND          470..472
FT                   /evidence="ECO:0007829|PDB:5Y3N"
FT   HELIX           483..489
FT                   /evidence="ECO:0007829|PDB:5Y3N"
FT   STRAND          497..501
FT                   /evidence="ECO:0007829|PDB:5Y3N"
FT   HELIX           505..509
FT                   /evidence="ECO:0007829|PDB:5Y3N"
FT   HELIX           512..515
FT                   /evidence="ECO:0007829|PDB:5Y3N"
FT   TURN            516..518
FT                   /evidence="ECO:0007829|PDB:5Y3N"
FT   STRAND          519..521
FT                   /evidence="ECO:0007829|PDB:5Y3N"
FT   STRAND          525..527
FT                   /evidence="ECO:0007829|PDB:5Y3N"
FT   HELIX           530..539
FT                   /evidence="ECO:0007829|PDB:5Y3N"
FT   STRAND          541..544
FT                   /evidence="ECO:0007829|PDB:4Z1H"
FT   STRAND          547..549
FT                   /evidence="ECO:0007829|PDB:5Y3N"
FT   HELIX           550..552
FT                   /evidence="ECO:0007829|PDB:6XG6"
FT   HELIX           576..589
FT                   /evidence="ECO:0007829|PDB:6XG6"
FT   TURN            590..593
FT                   /evidence="ECO:0007829|PDB:6XG6"
FT   STRAND          597..599
FT                   /evidence="ECO:0007829|PDB:6XG6"
FT   STRAND          607..611
FT                   /evidence="ECO:0007829|PDB:6XG6"
FT   HELIX           615..623
FT                   /evidence="ECO:0007829|PDB:6XG6"
FT   HELIX           631..638
FT                   /evidence="ECO:0007829|PDB:6XG6"
FT   STRAND          641..644
FT                   /evidence="ECO:0007829|PDB:6XG6"
FT   HELIX           649..660
FT                   /evidence="ECO:0007829|PDB:6XG6"
FT   HELIX           662..680
FT                   /evidence="ECO:0007829|PDB:6XG6"
FT   HELIX           689..700
FT                   /evidence="ECO:0007829|PDB:6XG6"
SQ   SEQUENCE   704 AA;  80110 MW;  4B16DE3D2B9E0285 CRC64;
     MARELRALLL WGRRLRPLLR APALAAVPGG KPILCPRRTT AQLGPRRNPA WSLQAGRLFS
     TQTAEDKEEP LHSIISSTES VQGSTSKHEF QAETKKLLDI VARSLYSEKE VFIRELISNA
     SDALEKLRHK LVSDGQALPE MEIHLQTNAE KGTITIQDTG IGMTQEELVS NLGTIARSGS
     KAFLDALQNQ AEASSKIIGQ FGVGFYSAFM VADRVEVYSR SAAPGSLGYQ WLSDGSGVFE
     IAEASGVRTG TKIIIHLKSD CKEFSSEARV RDVVTKYSNF VSFPLYLNGR RMNTLQAIWM
     MDPKDVREWQ HEEFYRYVAQ AHDKPRYTLH YKTDAPLNIR SIFYVPDMKP SMFDVSRELG
     SSVALYSRKV LIQTKATDIL PKWLRFIRGV VDSEDIPLNL SRELLQESAL IRKLRDVLQQ
     RLIKFFIDQS KKDAEKYAKF FEDYGLFMRE GIVTATEQEV KEDIAKLLRY ESSALPSGQL
     TSLSEYASRM RAGTRNIYYL CAPNRHLAEH SPYYEAMKKK DTEVLFCFEQ FDELTLLHLR
     EFDKKKLISV ETDIVVDHYK EEKFEDRSPA AECLSEKETE ELMAWMRNVL GSRVTNVKVT
     LRLDTHPAMV TVLEMGAARH FLRMQQLAKT QEERAQLLQP TLEINPRHAL IKKLNQLRAS
     EPGLAQLLVD QIYENAMIAA GLVDDPRAMV GRLNELLVKA LERH
```

|  |
| --- |
| **Mascot:** http://www.matrixscience.com/ |
