# Supplementary figures and images for: Chlorpromazine induces cytotoxic autophagy in glioblastoma cells via endoplasmic reticulum stress and unfolded protein response
Source: J Exp Clin Cancer Res. 2021 Nov 5;40:347. doi: 10.1186/s13046-021-02144-w (PMC8569984; doi:10.1186/s13046-021-02144-w)

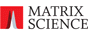

Supplement: Supplementary file 1 — Additional file 1. [file 13046_2021_2144_MOESM1_ESM.zip › MASCOT IDs/Mascot PMF_ ENPL_HUMAN_files/88x31_logo_white.gif]

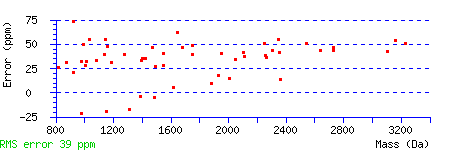

Supplement: Supplementary file 1 — Additional file 1. [file 13046_2021_2144_MOESM1_ESM.zip › MASCOT IDs/Mascot PMF_ ENPL_HUMAN_files/mass_error.pl.download]

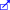

Supplement: Supplementary file 1 — Additional file 1. [file 13046_2021_2144_MOESM1_ESM.zip › MASCOT IDs/Mascot Search Results_ BIP_HUMAN_files/external_arrow.png]

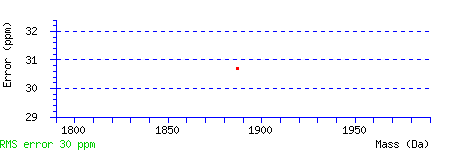

Supplement: Supplementary file 1 — Additional file 1. [file 13046_2021_2144_MOESM1_ESM.zip › MASCOT IDs/Mascot Search Results_ BIP_HUMAN_files/mass_error.pl.download]

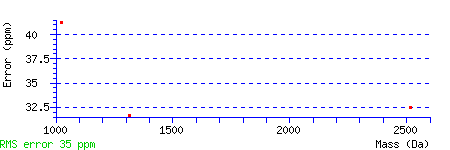

Supplement: Supplementary file 1 — Additional file 1. [file 13046_2021_2144_MOESM1_ESM.zip › MASCOT IDs/Mascot Search Results_ EF1A1_HUMAN_files/mass_error.pl.download]

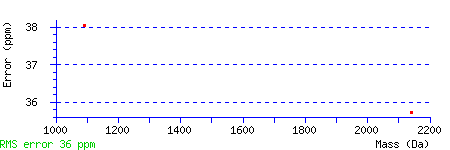

Supplement: Supplementary file 1 — Additional file 1. [file 13046_2021_2144_MOESM1_ESM.zip › MASCOT IDs/Mascot Search Results_ EF2_2HUMAN_files/mass_error.pl.download]

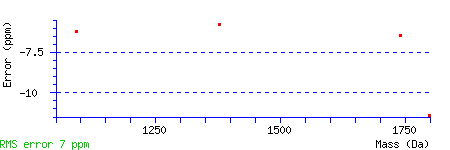

Supplement: Supplementary file 1 — Additional file 1. [file 13046_2021_2144_MOESM1_ESM.zip › MASCOT IDs/Mascot Search Results_ EF2_3HUMAN_files/mass_error.pl.download]

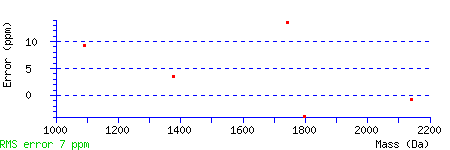

Supplement: Supplementary file 1 — Additional file 1. [file 13046_2021_2144_MOESM1_ESM.zip › MASCOT IDs/Mascot Search Results_ EF2_HUMAN_files/mass_error.pl.download]

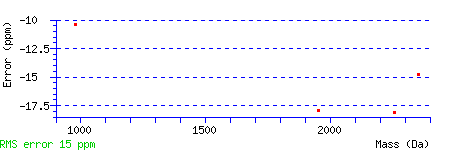

Supplement: Supplementary file 1 — Additional file 1. [file 13046_2021_2144_MOESM1_ESM.zip › MASCOT IDs/Mascot Search Results_ ENPL_HUMAN2_files/mass_error.pl.download]

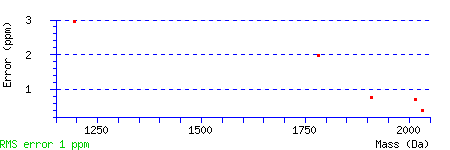

Supplement: Supplementary file 1 — Additional file 1. [file 13046_2021_2144_MOESM1_ESM.zip › MASCOT IDs/Mascot Search Results_ HS90B_2HUMAN_files/mass_error.pl.download]

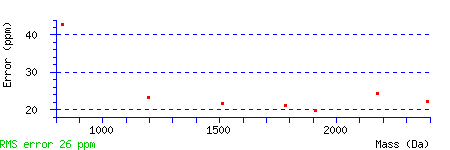

Supplement: Supplementary file 1 — Additional file 1. [file 13046_2021_2144_MOESM1_ESM.zip › MASCOT IDs/Mascot Search Results_ HS90B_HUMAN3_files/mass_error.pl.download]

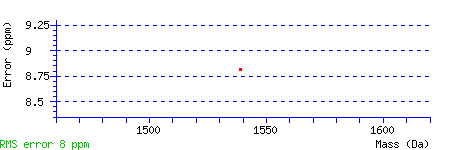

Supplement: Supplementary file 1 — Additional file 1. [file 13046_2021_2144_MOESM1_ESM.zip › MASCOT IDs/Mascot Search Results_ TCPA_HUMAN_files/mass_error.pl.download]

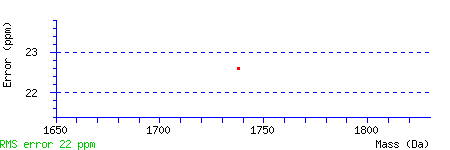

Supplement: Supplementary file 1 — Additional file 1. [file 13046_2021_2144_MOESM1_ESM.zip › MASCOT IDs/Mascot Search Results_ TCPE_HUMAN_files/mass_error.pl.download]

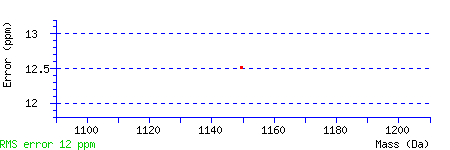

Supplement: Supplementary file 1 — Additional file 1. [file 13046_2021_2144_MOESM1_ESM.zip › MASCOT IDs/Mascot Search Results_ TCPQ_HUMAN_files/mass_error.pl.download]

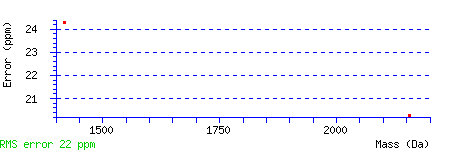

Supplement: Supplementary file 1 — Additional file 1. [file 13046_2021_2144_MOESM1_ESM.zip › MASCOT IDs/Mascot Search Results_ TCPZ_HUMAN_files/mass_error.pl.download]

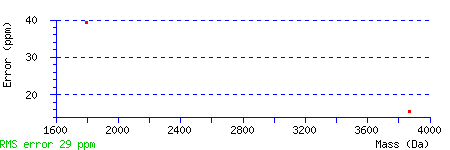

Supplement: Supplementary file 1 — Additional file 1. [file 13046_2021_2144_MOESM1_ESM.zip › MASCOT IDs/Mascot Search Results_ TERA_HUMAN_files/mass_error.pl.download]

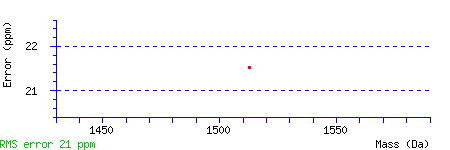

Supplement: Supplementary file 1 — Additional file 1. [file 13046_2021_2144_MOESM1_ESM.zip › MASCOT IDs/Mascot Search Results_ TRAP1_HUMAN2_files/mass_error.pl.download]

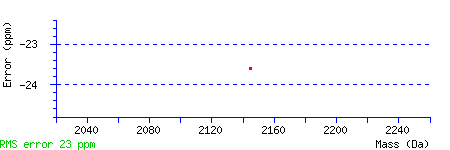

Supplement: Supplementary file 1 — Additional file 1. [file 13046_2021_2144_MOESM1_ESM.zip › MASCOT IDs/Mascot Search Results_ TRAP1_HUMAN3_files/mass_error.pl.download]

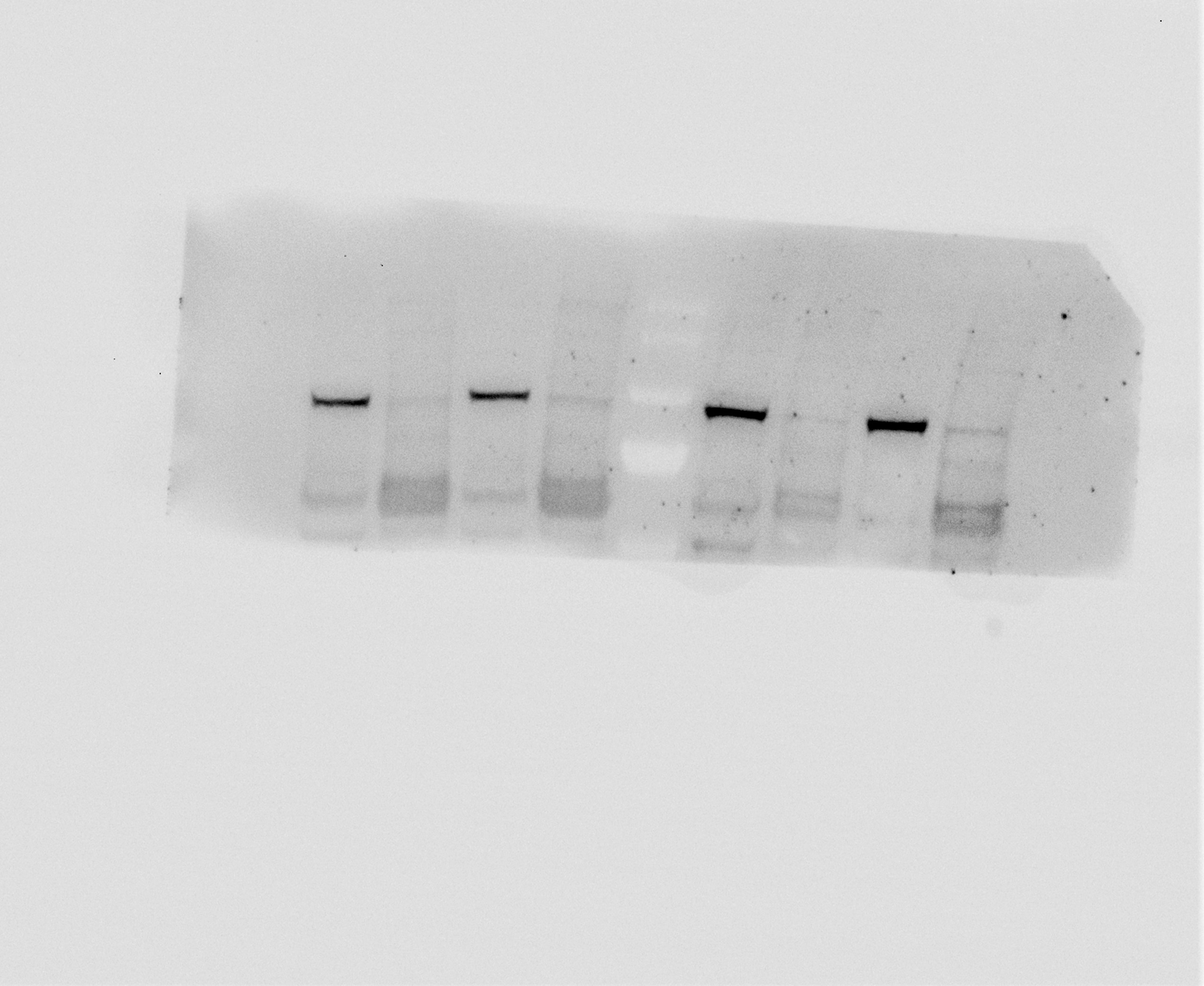

Supplement: Supplementary file 3 — Additional file 3. [file 13046_2021_2144_MOESM3_ESM.zip › ATF6 C-N/RPE-1/ATF6 RPE-1.jpg]

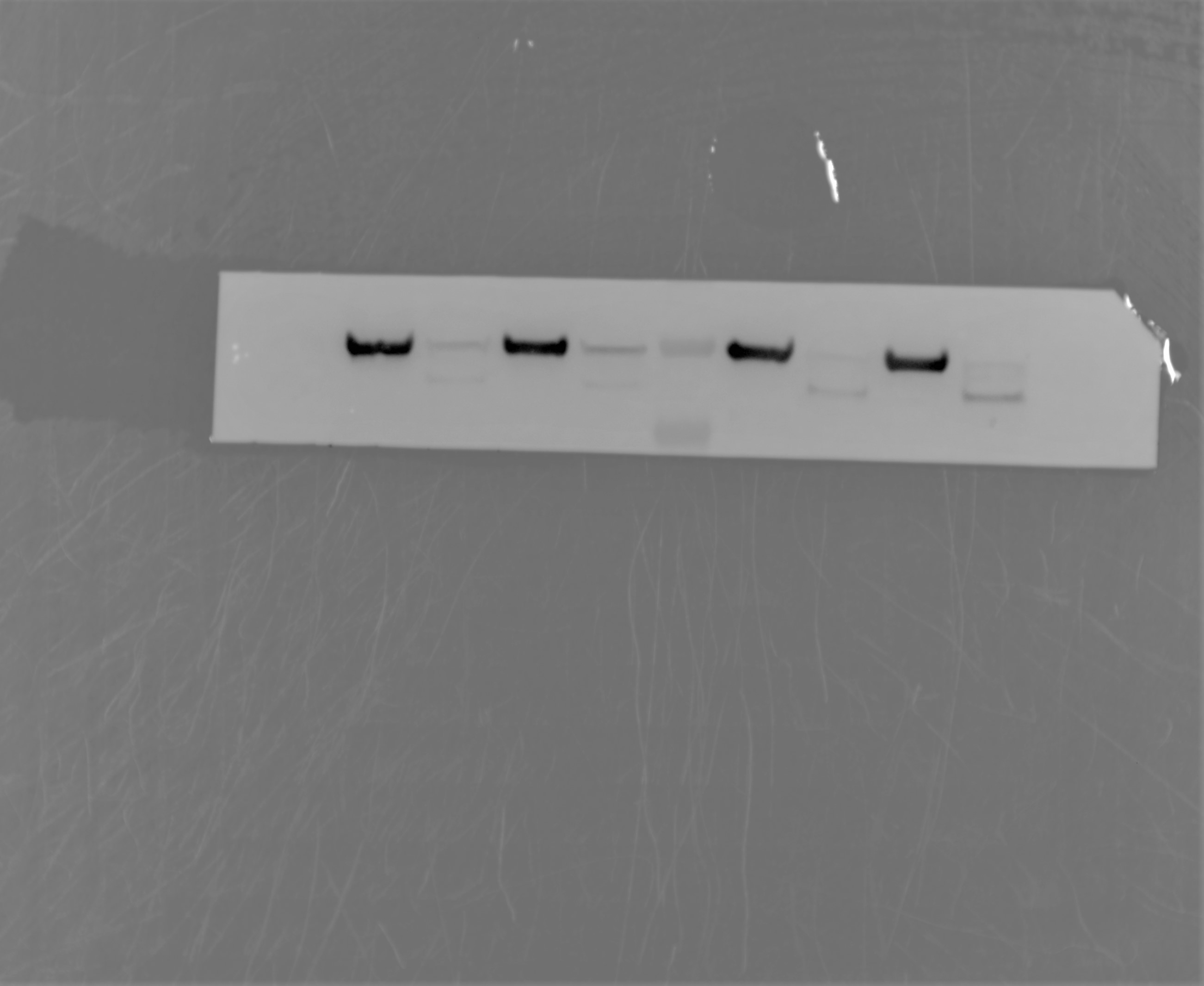

Supplement: Supplementary file 3 — Additional file 3. [file 13046_2021_2144_MOESM3_ESM.zip › ATF6 C-N/RPE-1/GAPDH RPE-1.jpg]

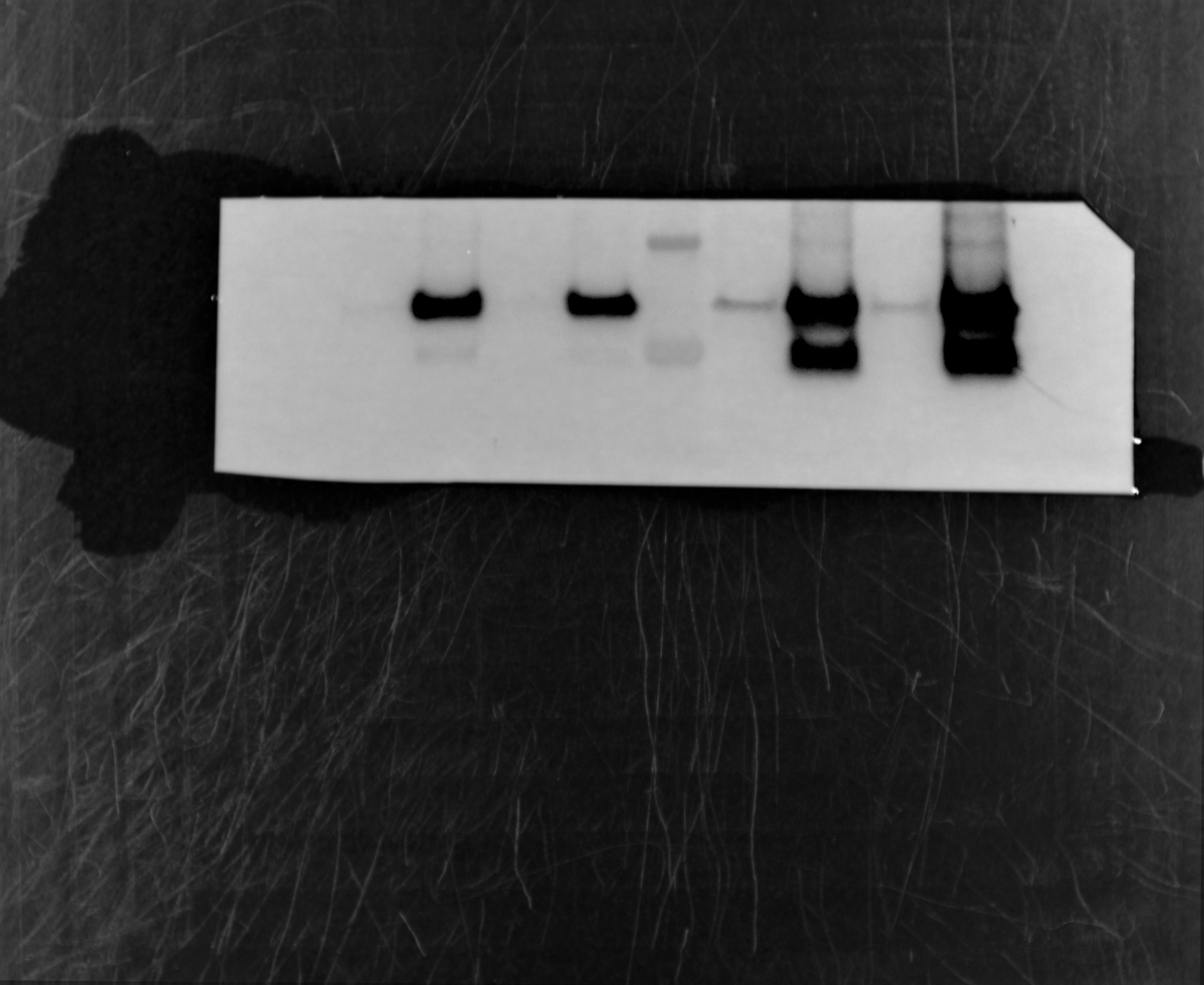

Supplement: Supplementary file 3 — Additional file 3. [file 13046_2021_2144_MOESM3_ESM.zip › ATF6 C-N/RPE-1/H3 RPE-1.jpg]

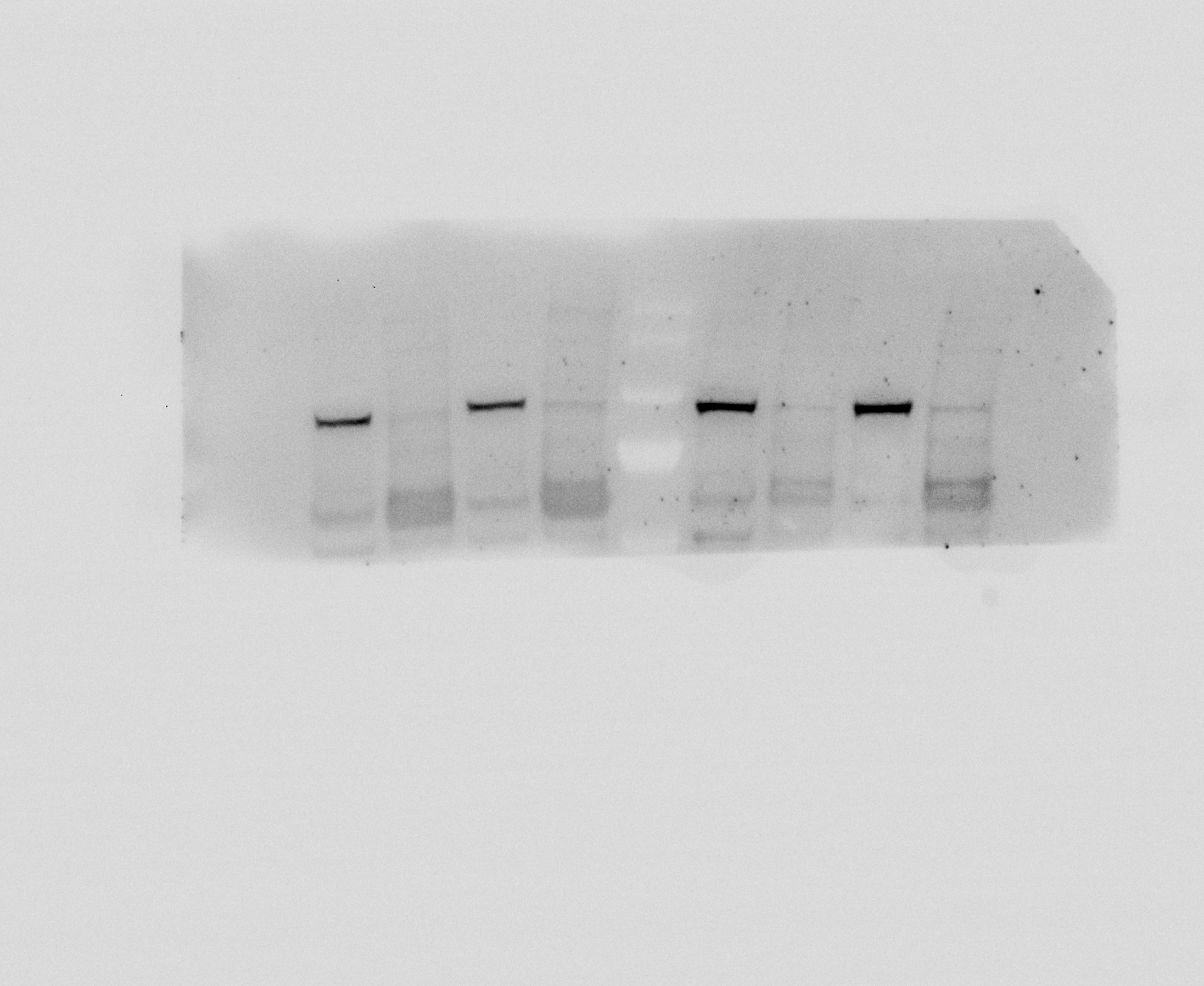

Supplement: Supplementary file 3 — Additional file 3. [file 13046_2021_2144_MOESM3_ESM.zip › ATF6 C-N/T98G/ATF6 T98G.jpg]

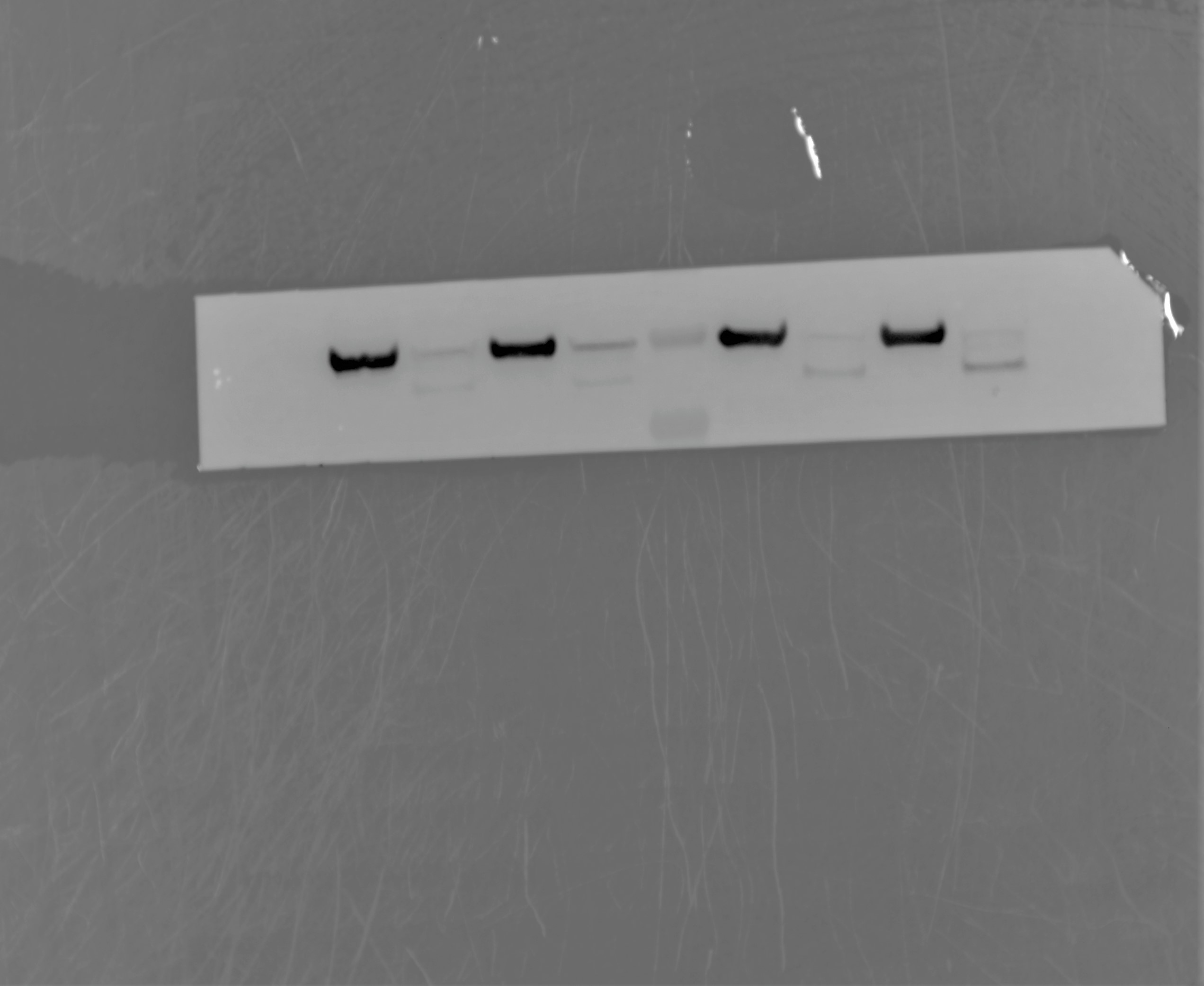

Supplement: Supplementary file 3 — Additional file 3. [file 13046_2021_2144_MOESM3_ESM.zip › ATF6 C-N/T98G/GAPDH T98G.jpg]

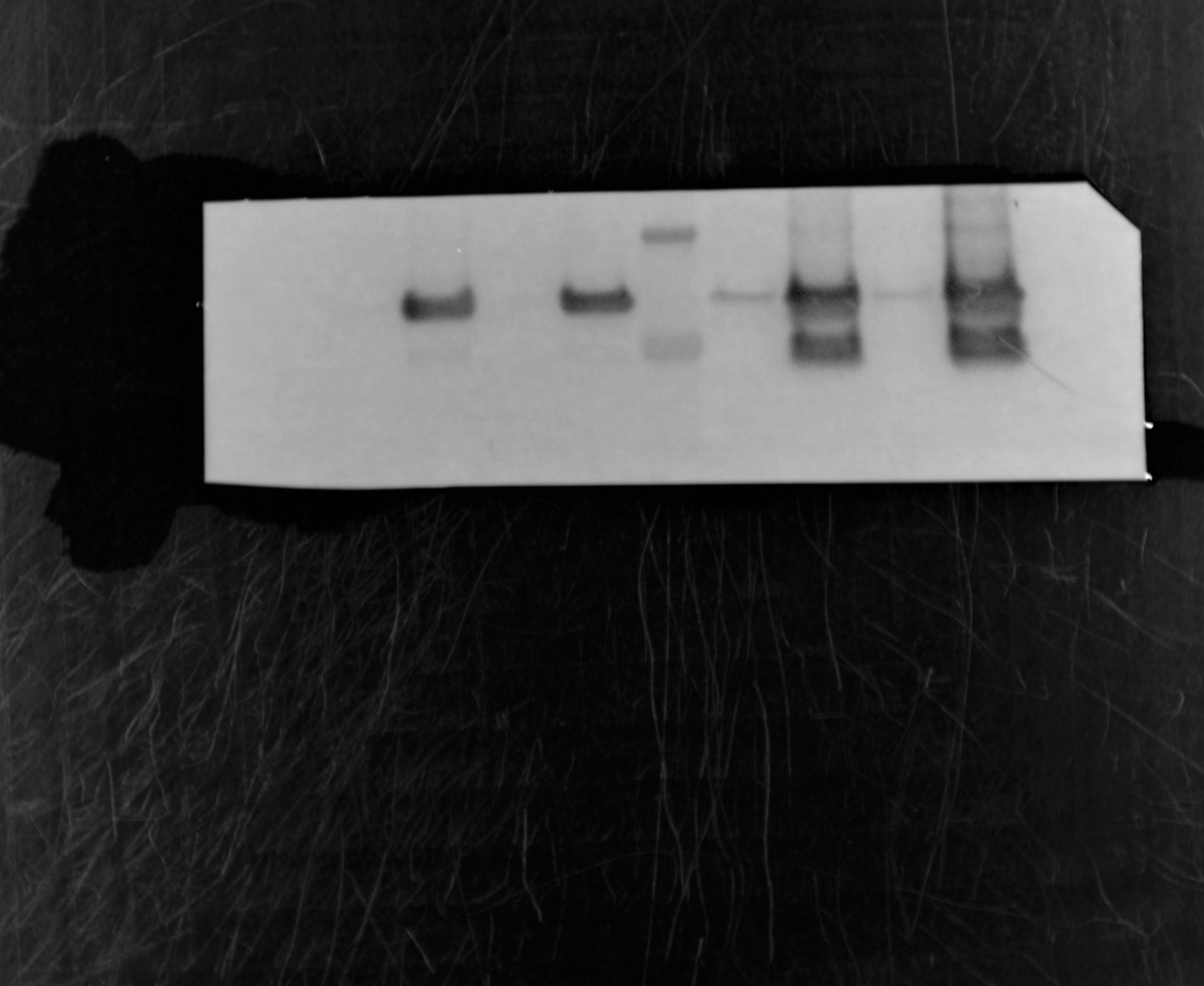

Supplement: Supplementary file 3 — Additional file 3. [file 13046_2021_2144_MOESM3_ESM.zip › ATF6 C-N/T98G/H3 T98G.jpg]

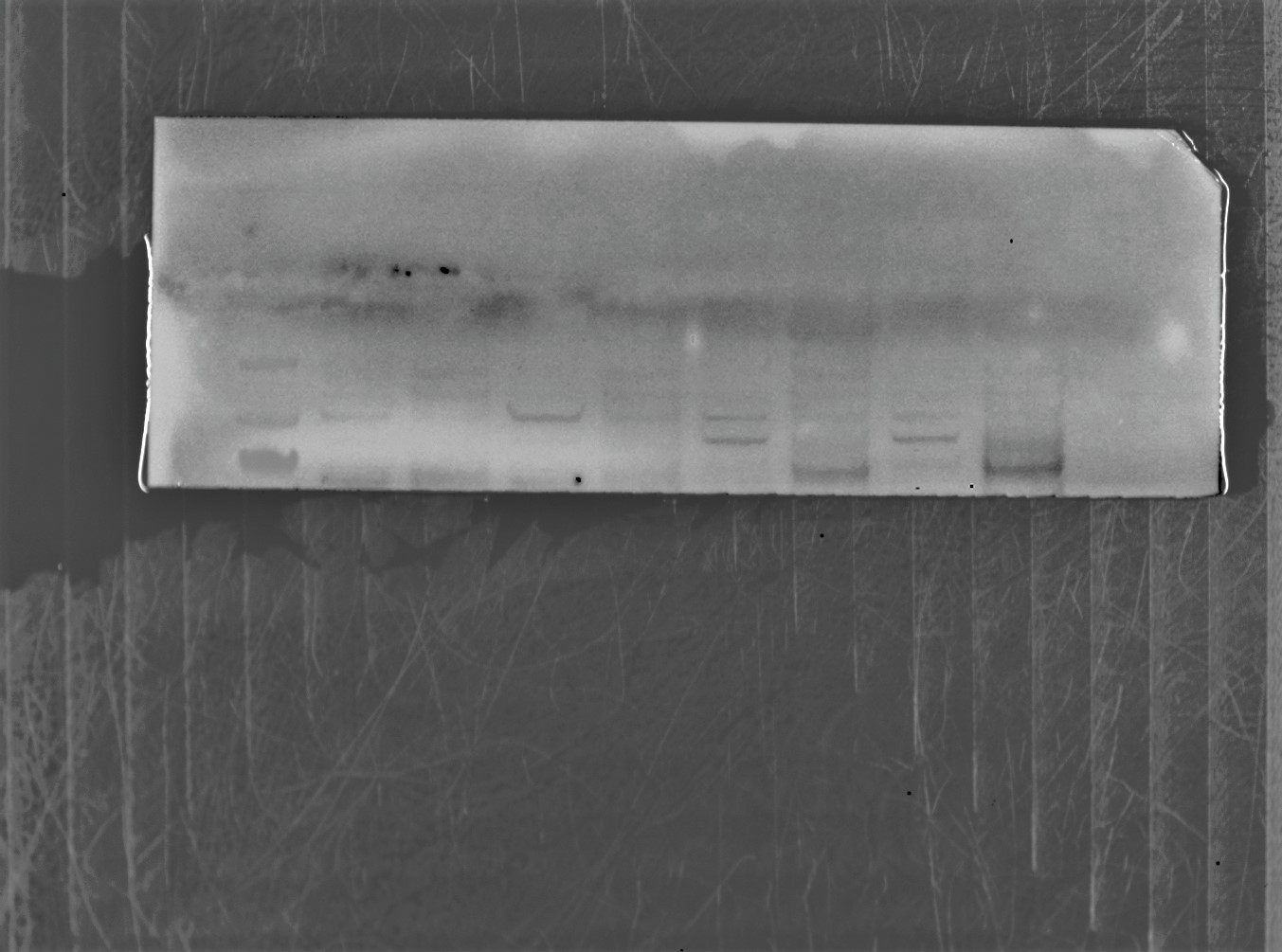

Supplement: Supplementary file 3 — Additional file 3. [file 13046_2021_2144_MOESM3_ESM.zip › ATF6 C-N/TS #163/ATF6 TS#163.jpg]

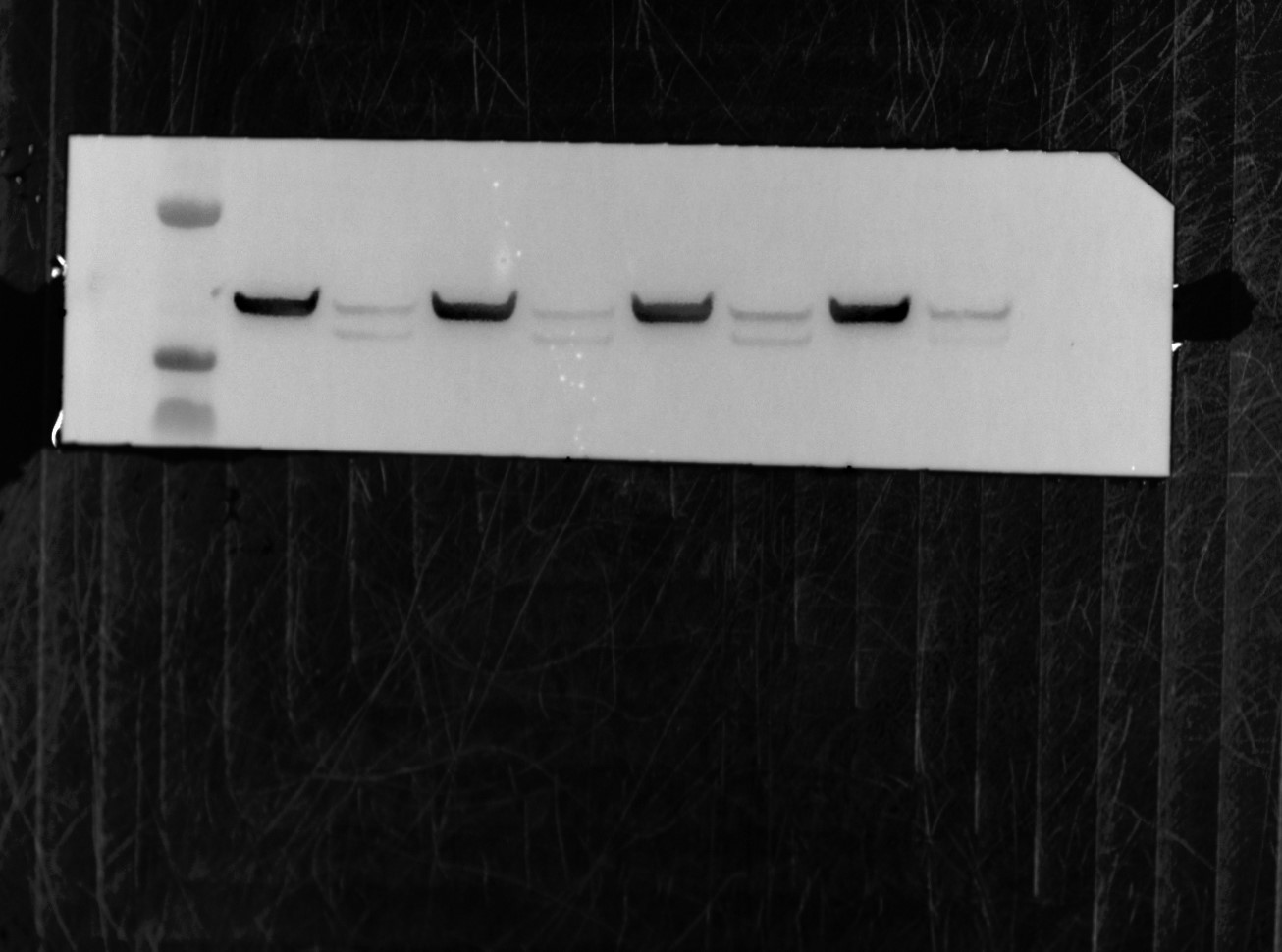

Supplement: Supplementary file 3 — Additional file 3. [file 13046_2021_2144_MOESM3_ESM.zip › ATF6 C-N/TS #163/GAPDH TS#163.jpg]

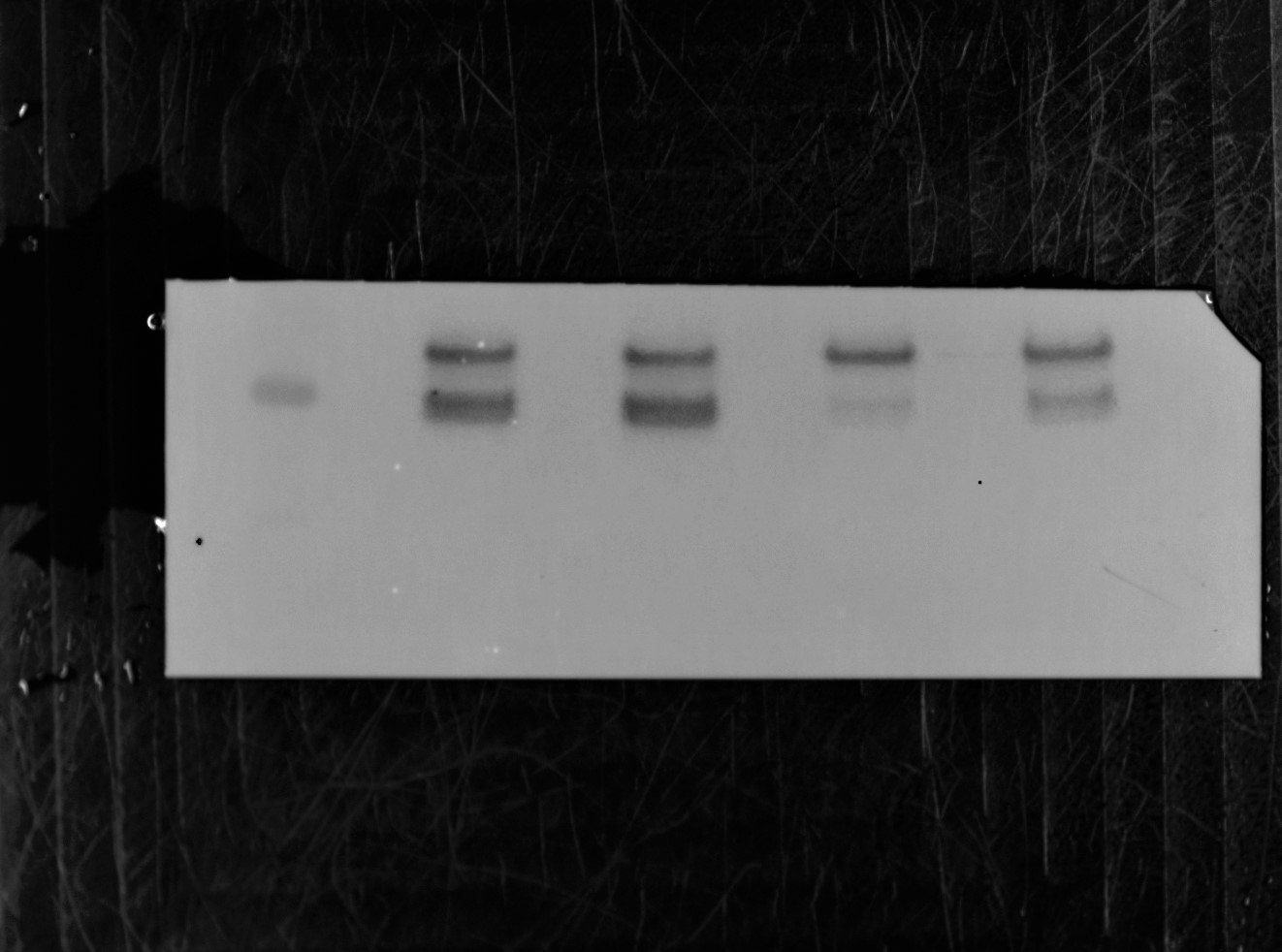

Supplement: Supplementary file 3 — Additional file 3. [file 13046_2021_2144_MOESM3_ESM.zip › ATF6 C-N/TS #163/H3 TS#163.jpg]

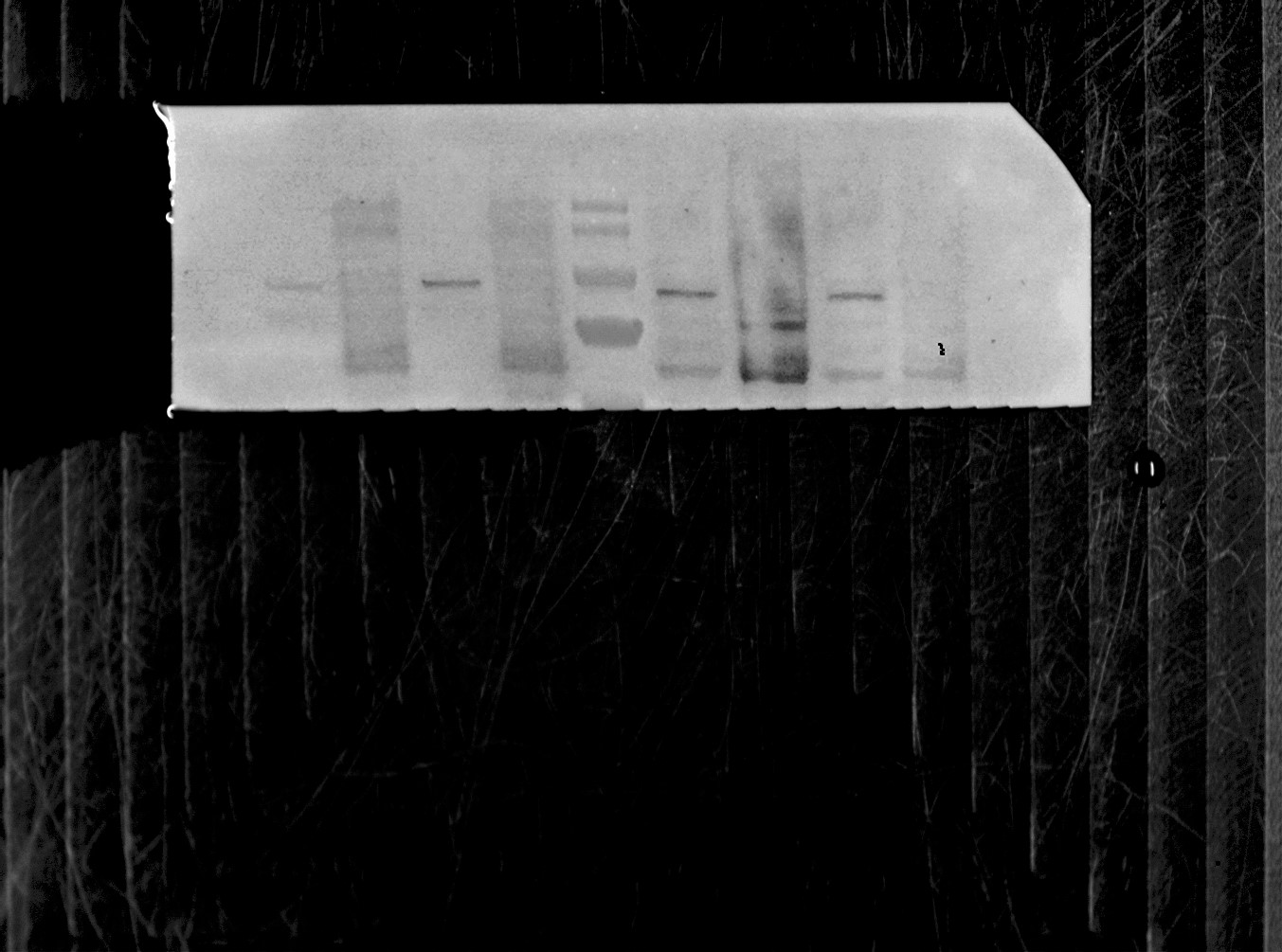

Supplement: Supplementary file 3 — Additional file 3. [file 13046_2021_2144_MOESM3_ESM.zip › ATF6 C-N/TS #1/ATF6 TS#1.jpg]

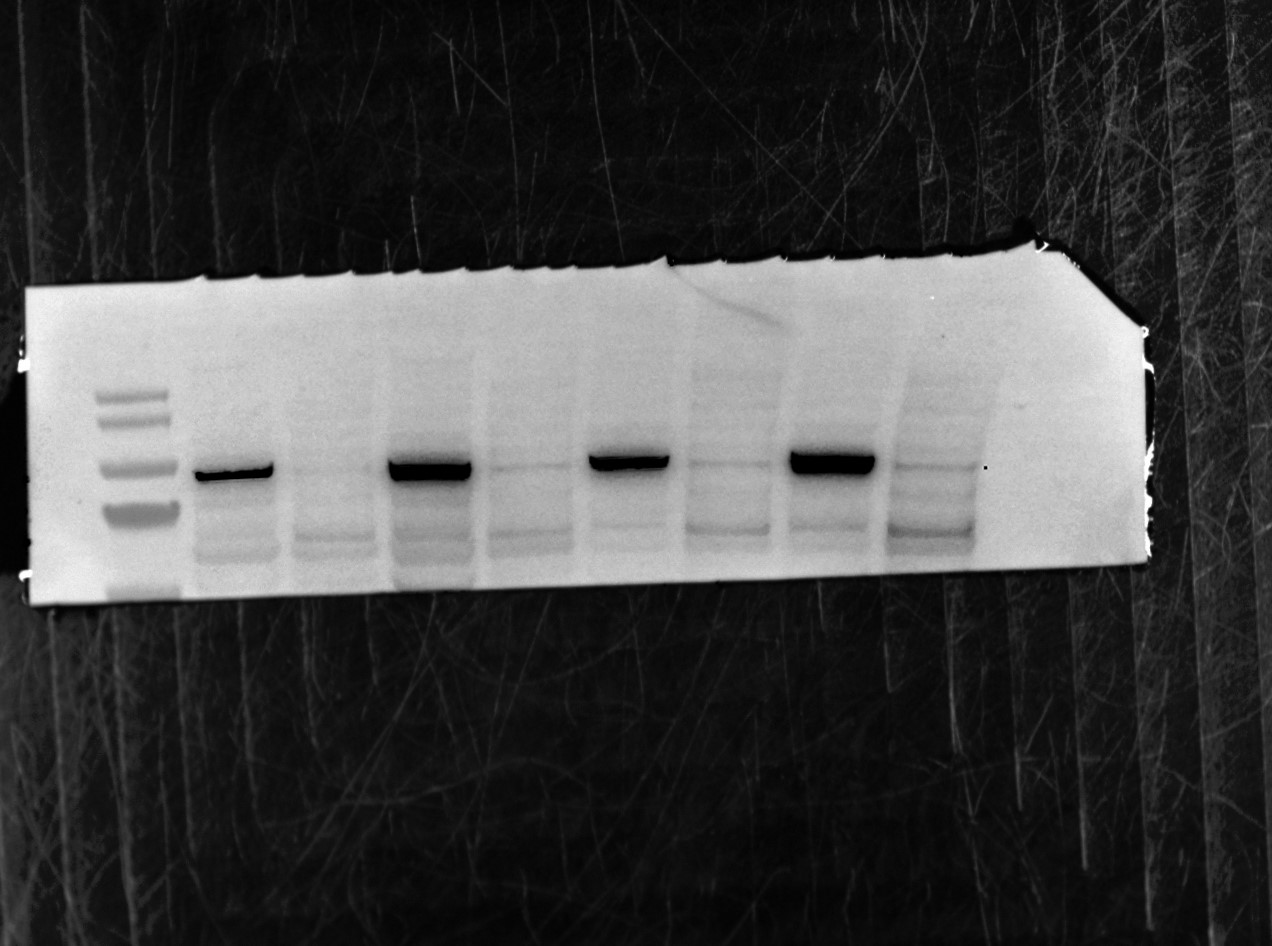

Supplement: Supplementary file 3 — Additional file 3. [file 13046_2021_2144_MOESM3_ESM.zip › ATF6 C-N/TS #83/ATF6 TS#83.jpg]

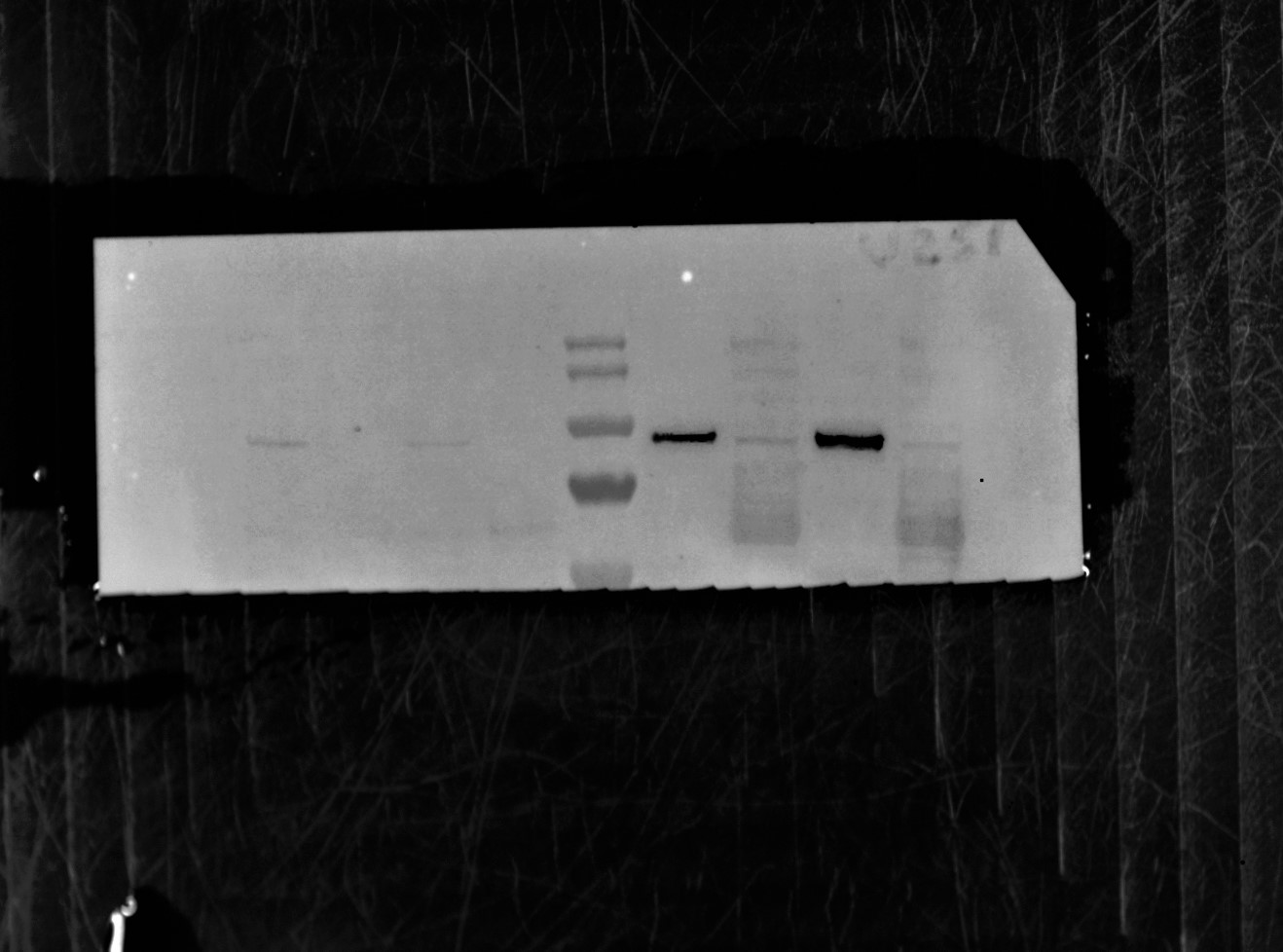

Supplement: Supplementary file 3 — Additional file 3. [file 13046_2021_2144_MOESM3_ESM.zip › ATF6 C-N/U-251 MG/ATF6 U251MG.jpg]

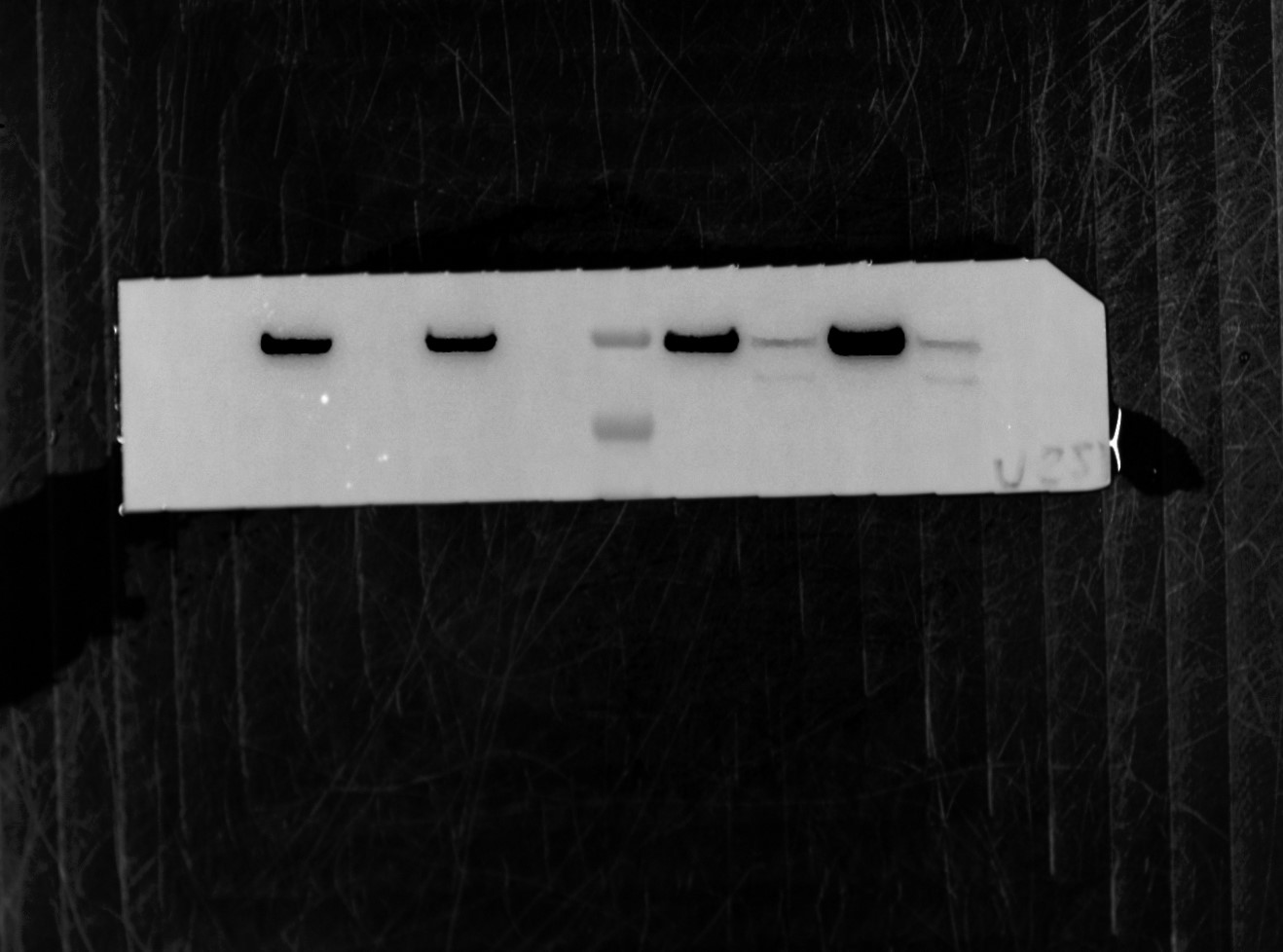

Supplement: Supplementary file 3 — Additional file 3. [file 13046_2021_2144_MOESM3_ESM.zip › ATF6 C-N/U-251 MG/GAPDH U251MG.jpg]

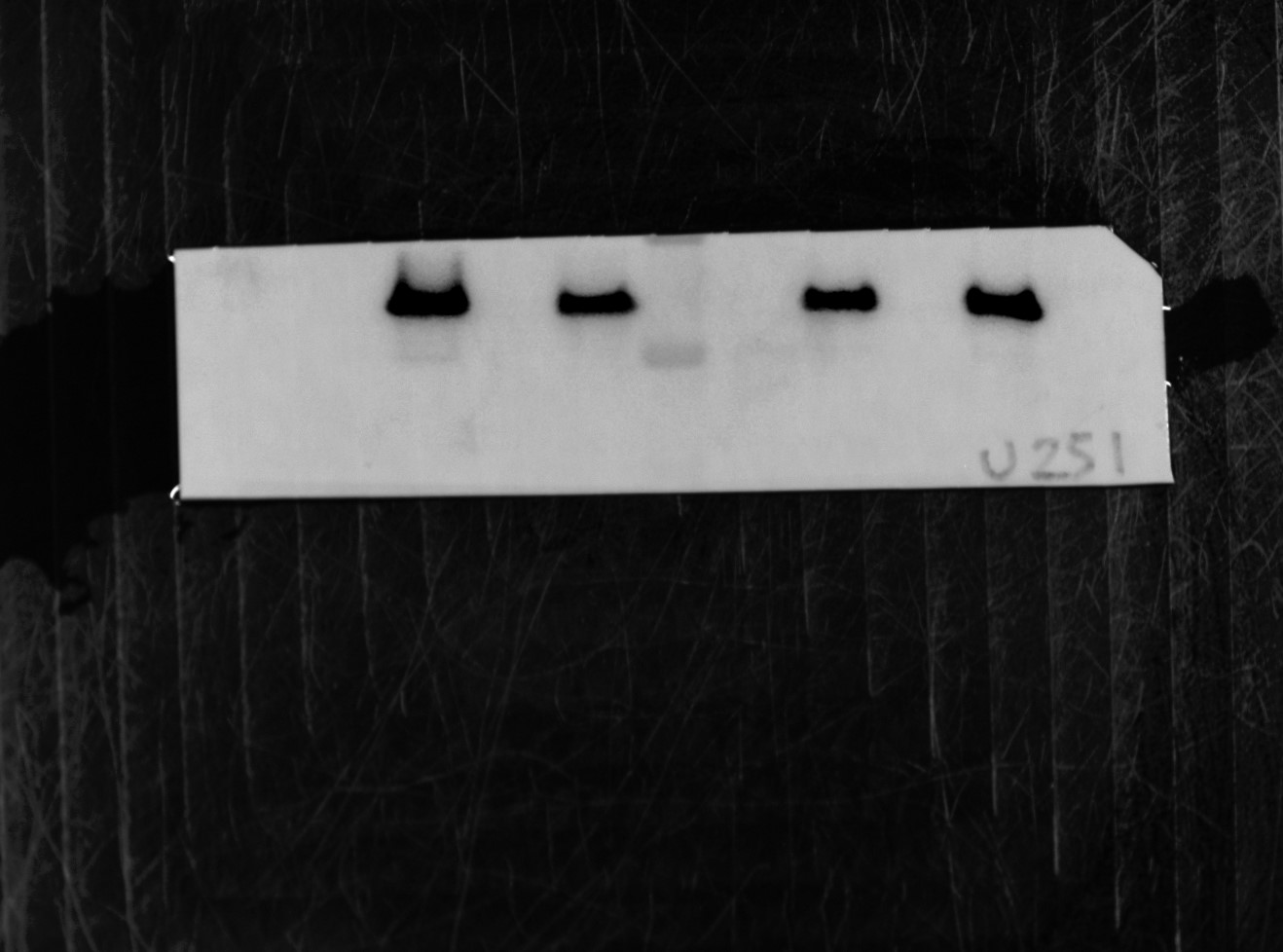

Supplement: Supplementary file 3 — Additional file 3. [file 13046_2021_2144_MOESM3_ESM.zip › ATF6 C-N/U-251 MG/H3 U251MG.jpg]

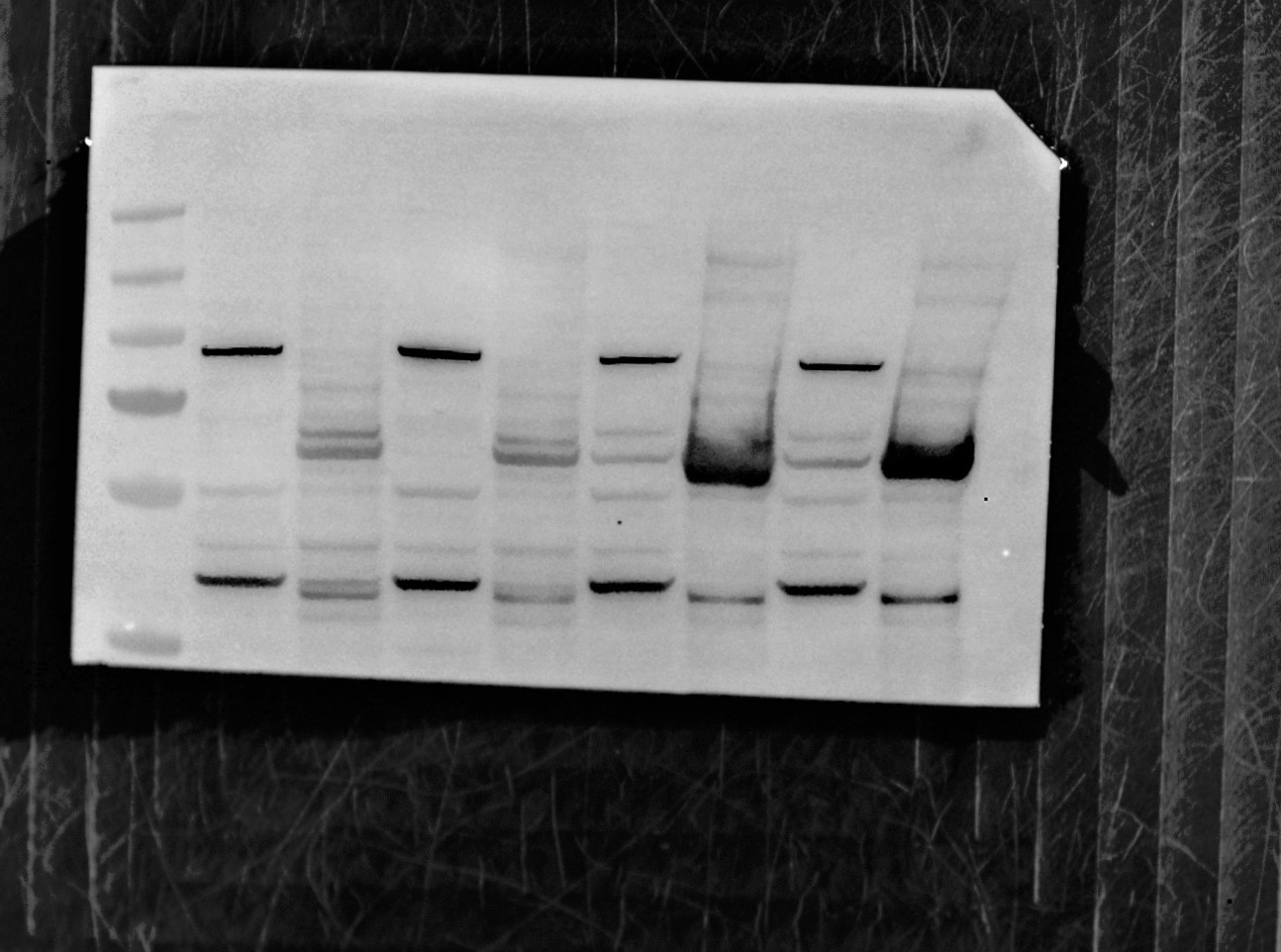

Supplement: Supplementary file 3 — Additional file 3. [file 13046_2021_2144_MOESM3_ESM.zip › ATF6 C-N/U-87 MG/ATF6 U87MG.jpg]

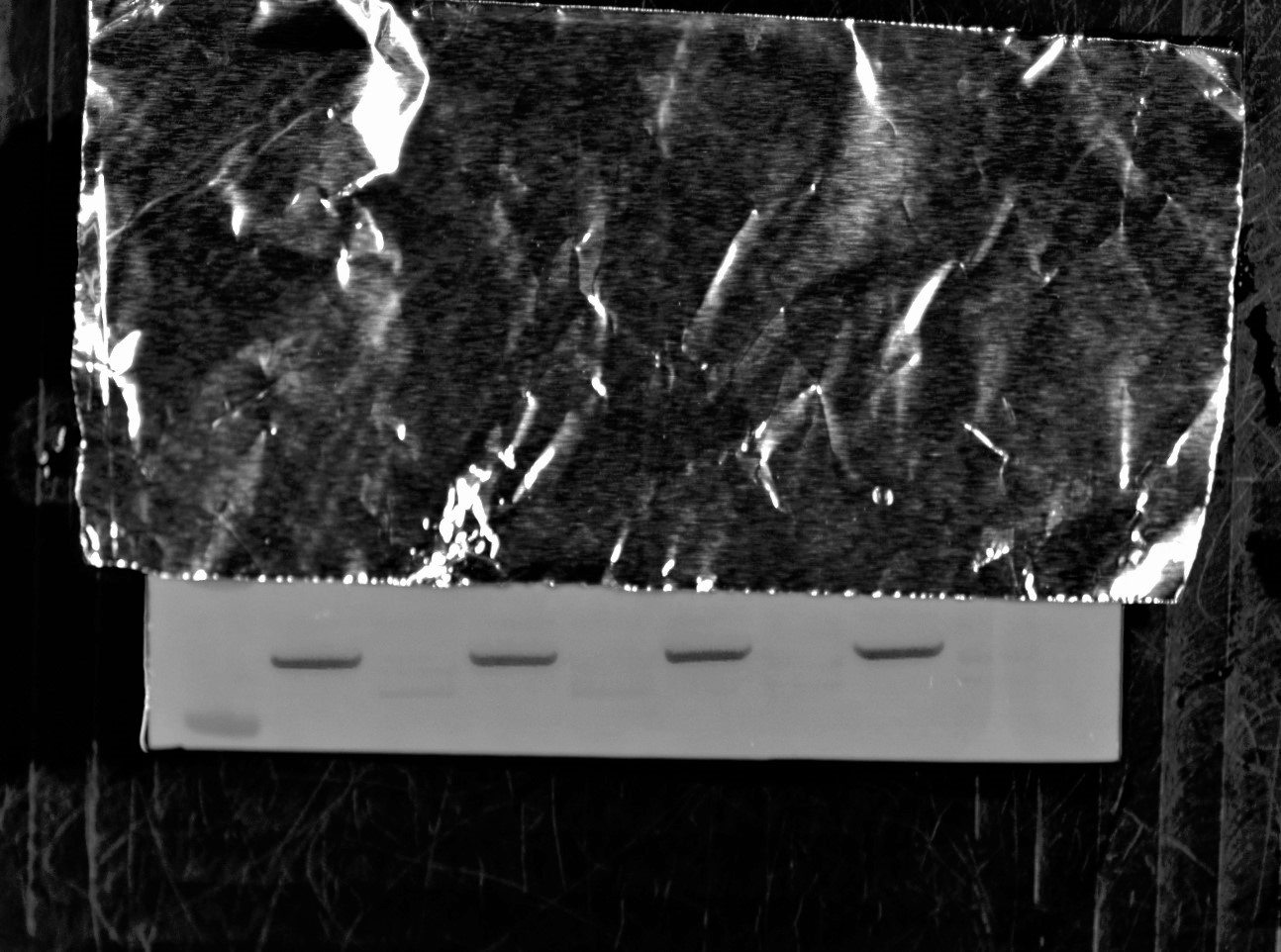

Supplement: Supplementary file 3 — Additional file 3. [file 13046_2021_2144_MOESM3_ESM.zip › ATF6 C-N/U-87 MG/GAPDH U87MG.jpg]

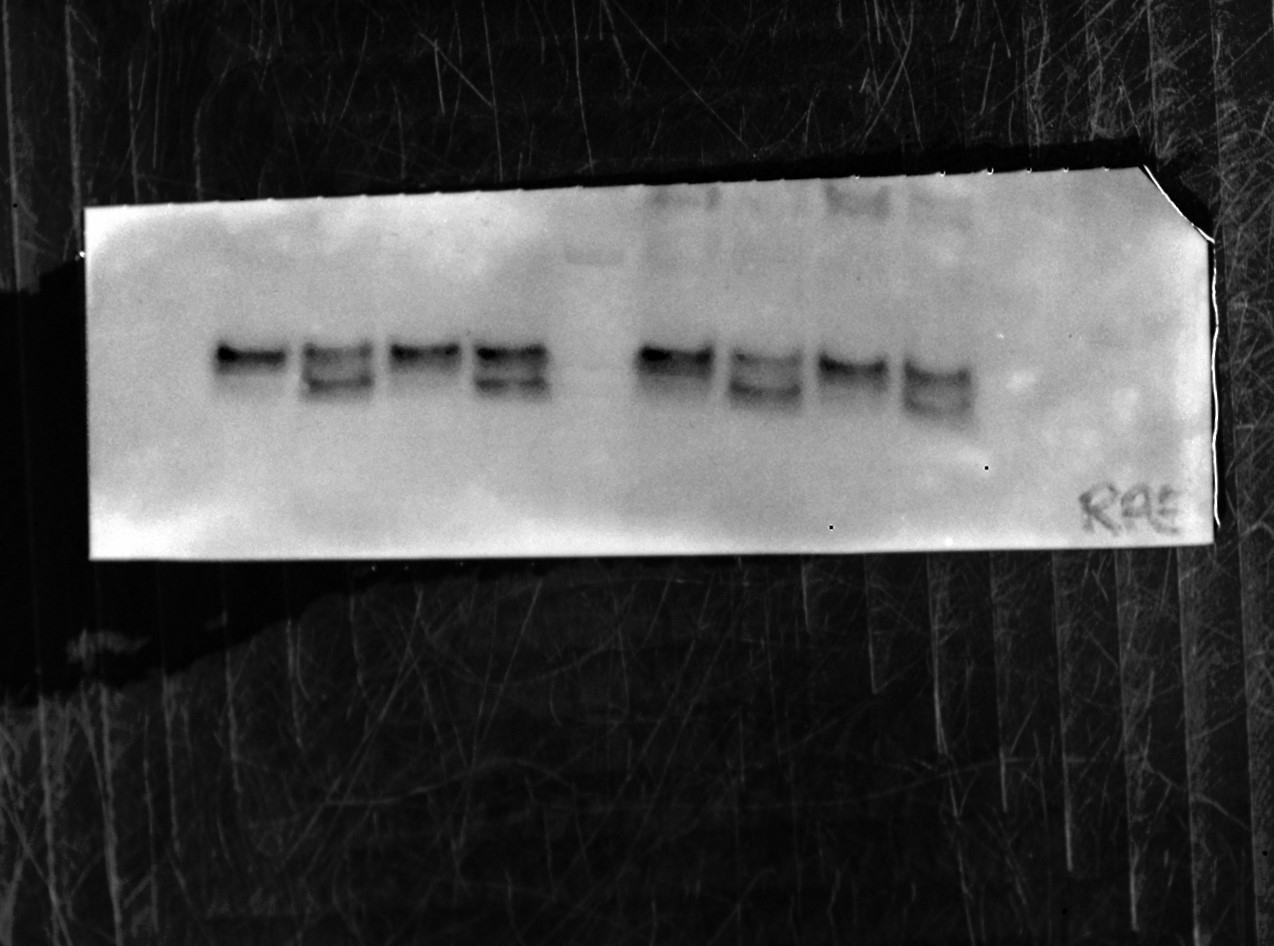

Supplement: Supplementary file 4 — Additional file 4. [file 13046_2021_2144_MOESM4_ESM.zip › WB Autophagy CPZ+PBA/RPE-1/LC3 RPE.jpg]

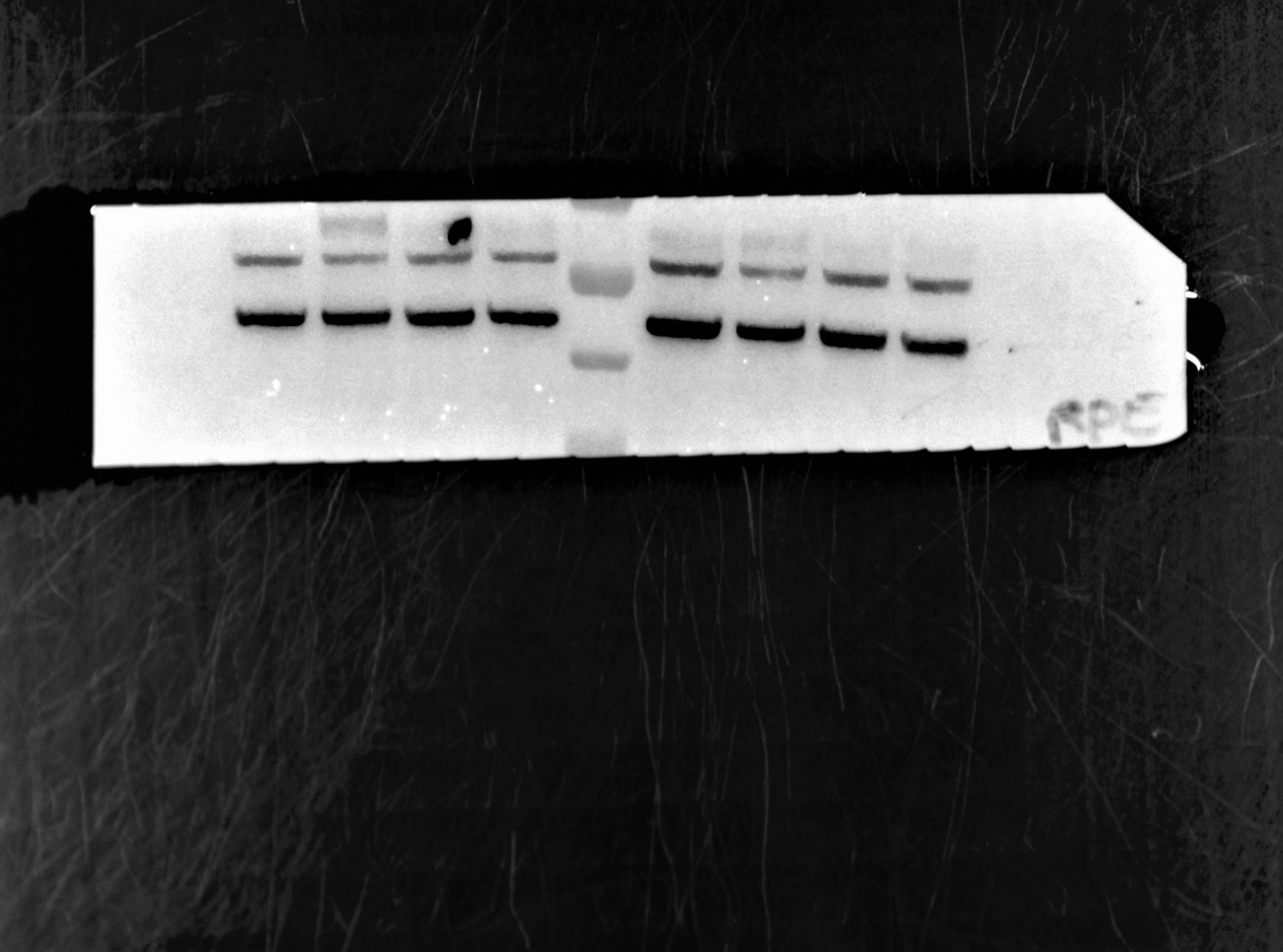

Supplement: Supplementary file 4 — Additional file 4. [file 13046_2021_2144_MOESM4_ESM.zip › WB Autophagy CPZ+PBA/RPE-1/p62 RPE.jpg]

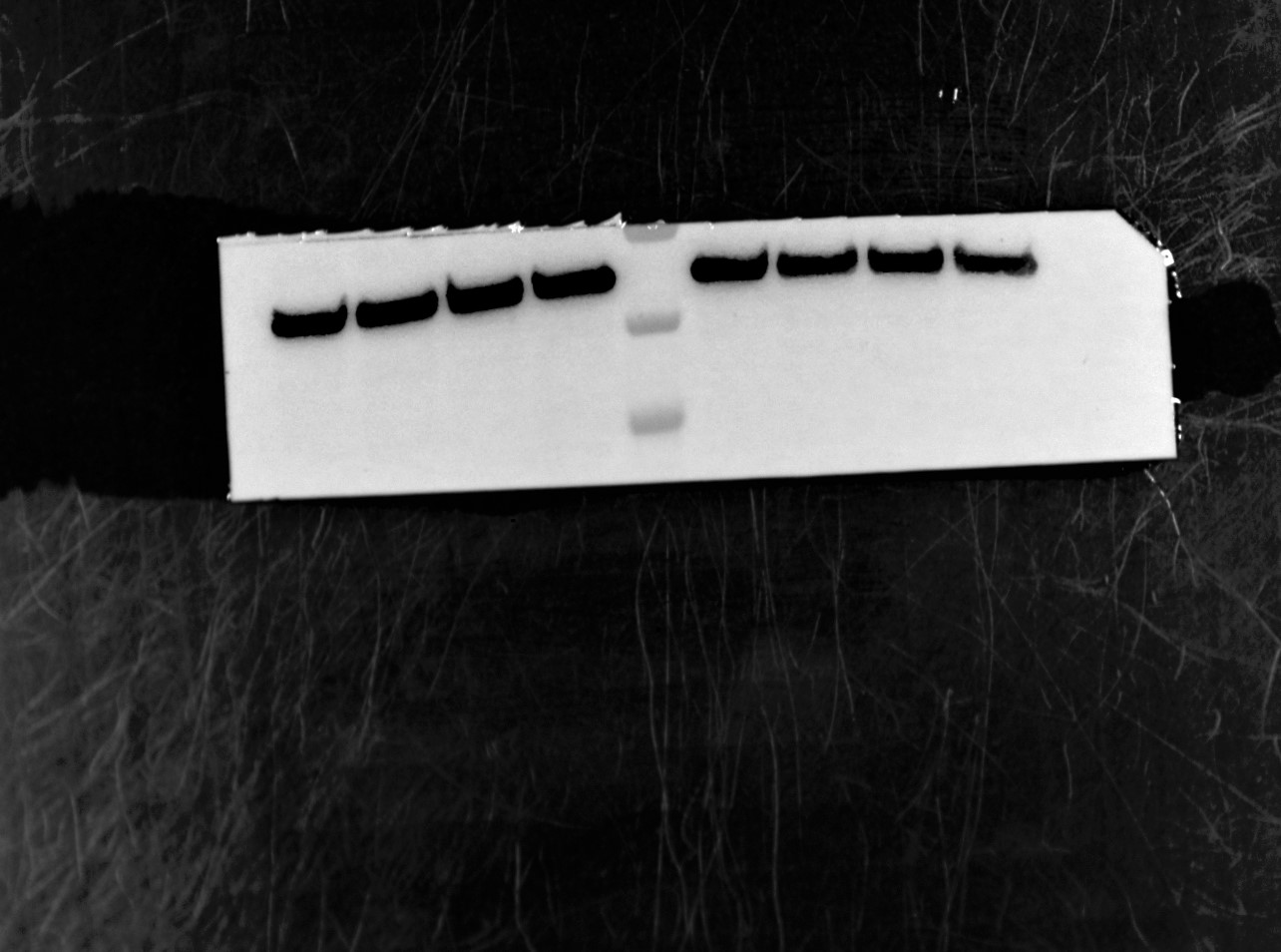

Supplement: Supplementary file 4 — Additional file 4. [file 13046_2021_2144_MOESM4_ESM.zip › WB Autophagy CPZ+PBA/T98G/actina T98G.jpg]

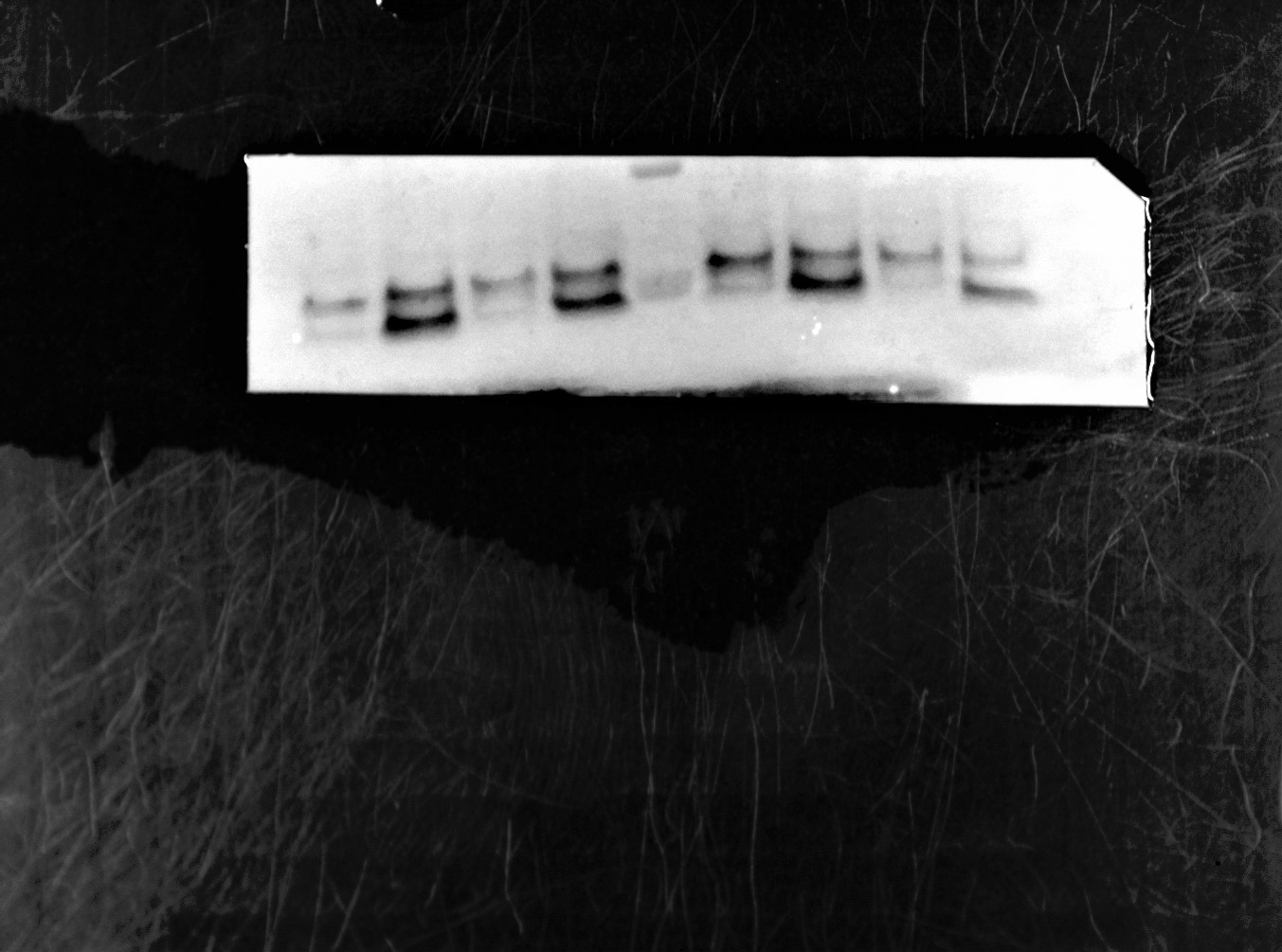

Supplement: Supplementary file 4 — Additional file 4. [file 13046_2021_2144_MOESM4_ESM.zip › WB Autophagy CPZ+PBA/T98G/LC3 T98G.jpg]

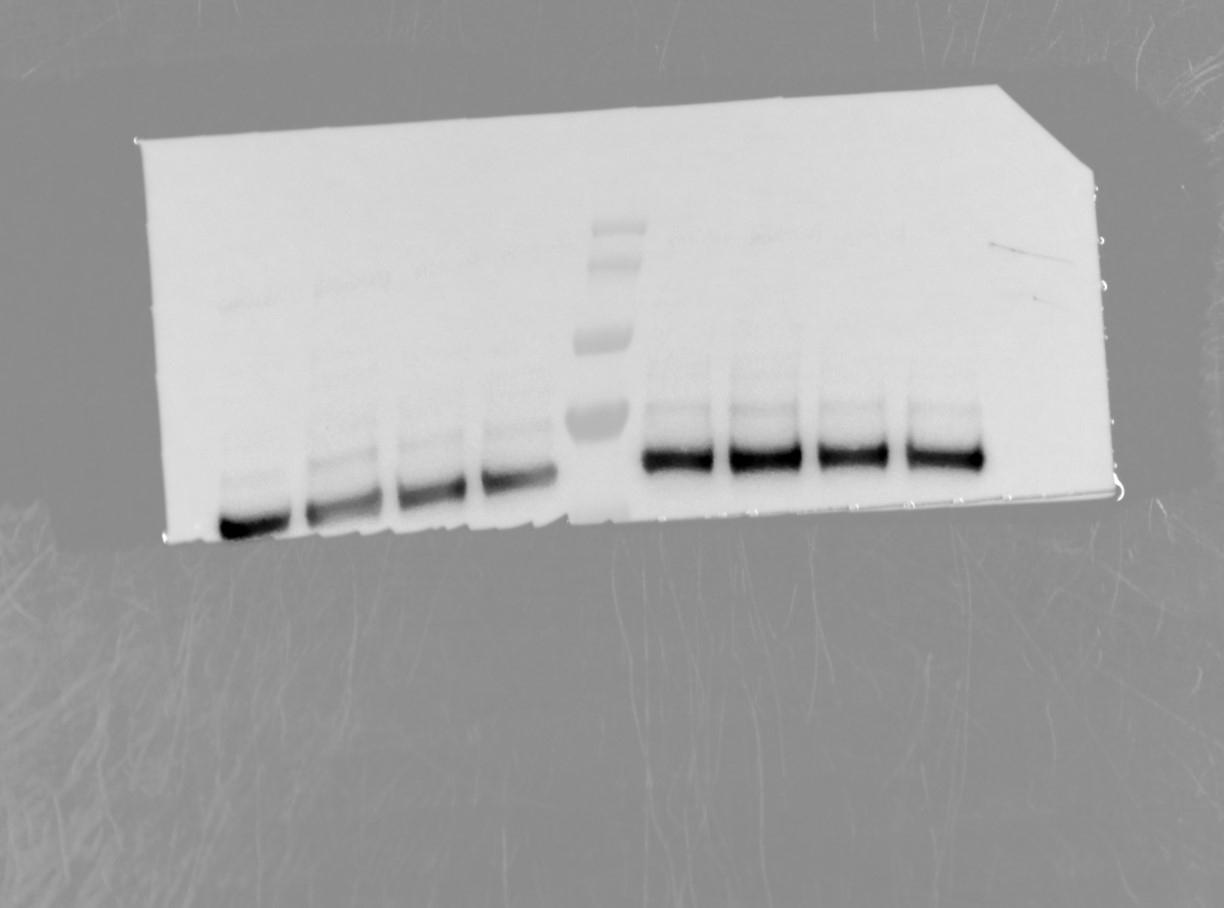

Supplement: Supplementary file 4 — Additional file 4. [file 13046_2021_2144_MOESM4_ESM.zip › WB Autophagy CPZ+PBA/T98G/p62 T98G.jpg]

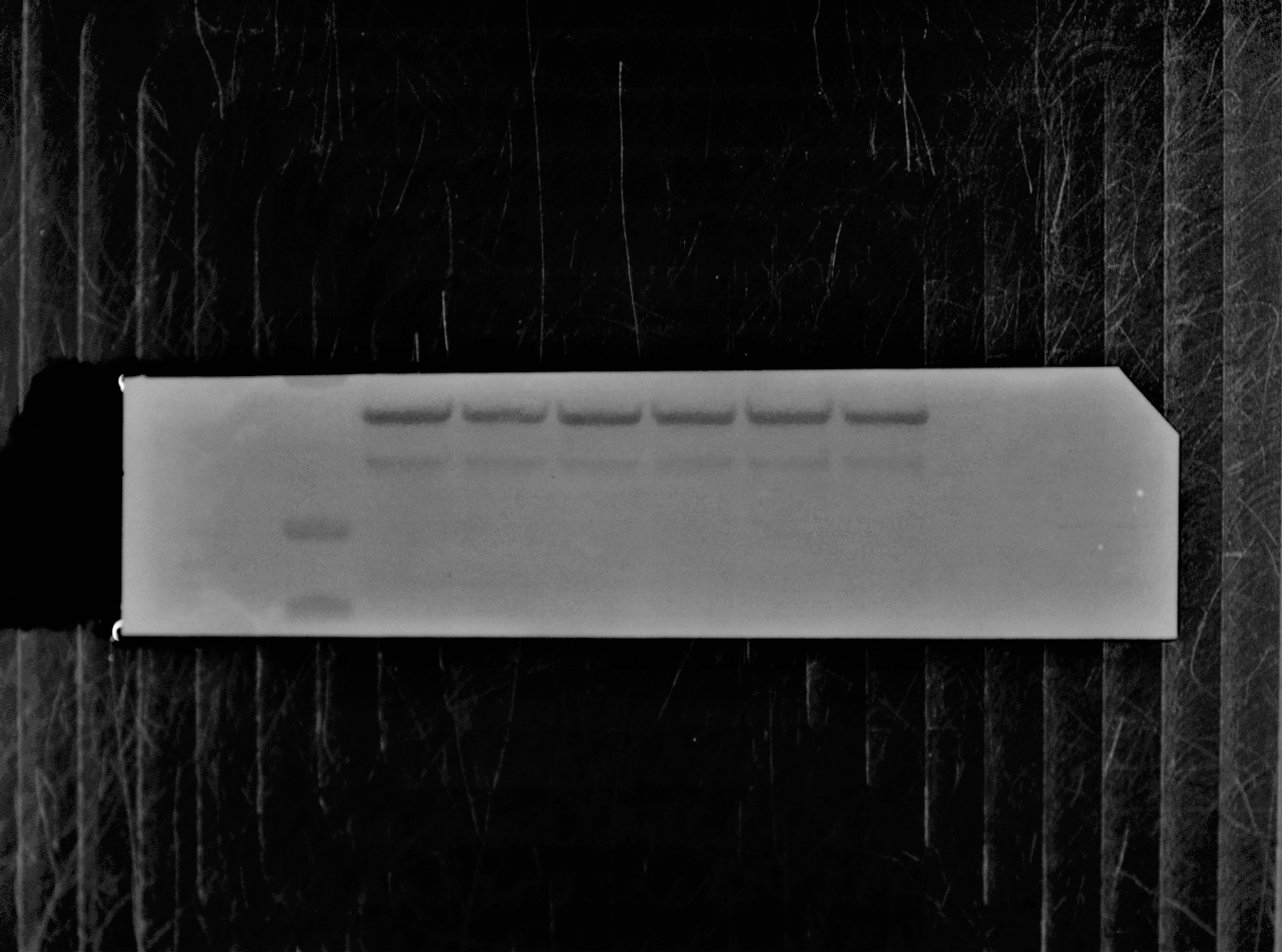

Supplement: Supplementary file 4 — Additional file 4. [file 13046_2021_2144_MOESM4_ESM.zip › WB Autophagy CPZ+PBA/TS #163/#163 actina (p62).Tif]

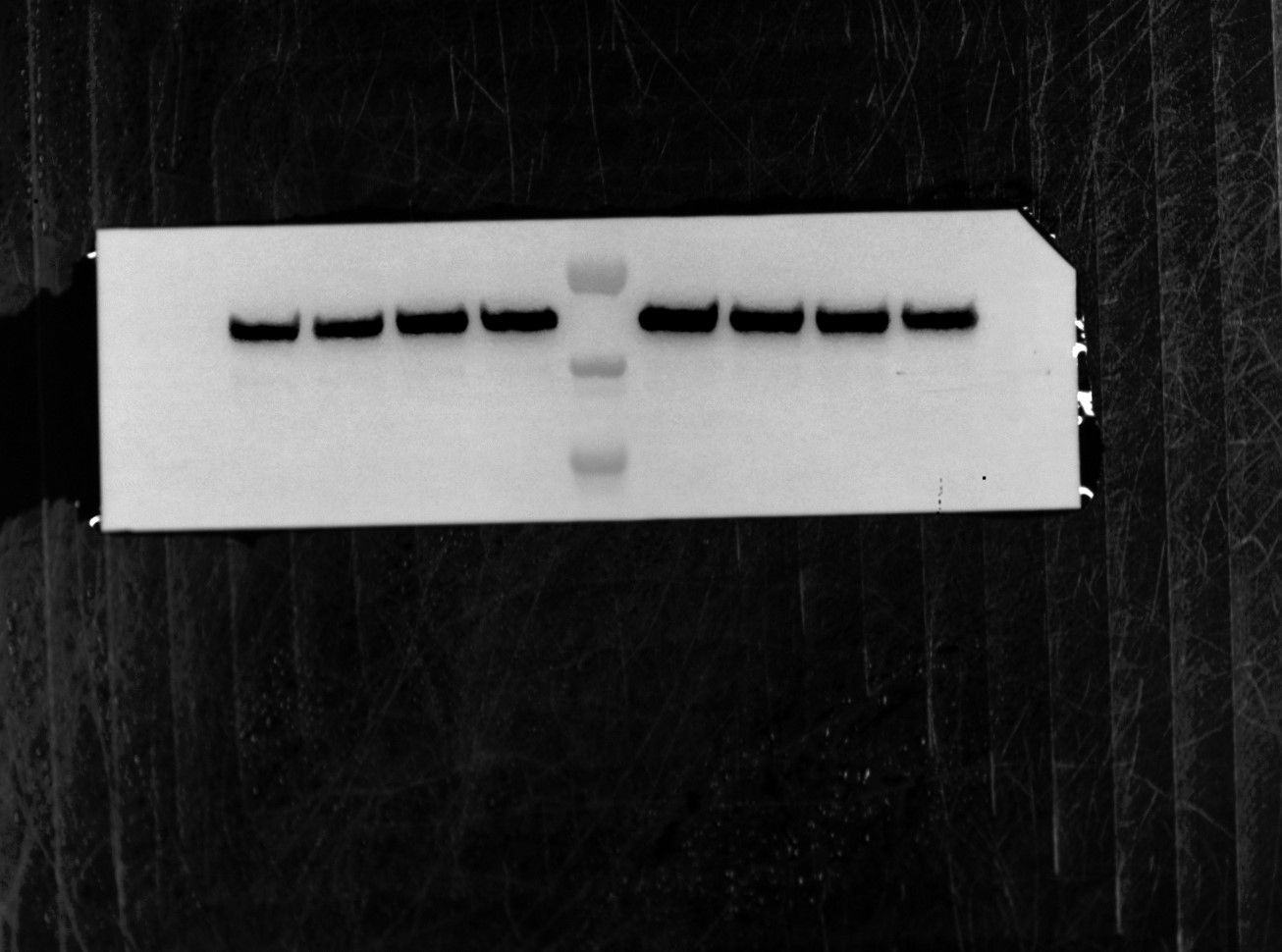

Supplement: Supplementary file 4 — Additional file 4. [file 13046_2021_2144_MOESM4_ESM.zip › WB Autophagy CPZ+PBA/TS #163/actina TS#163.jpg]

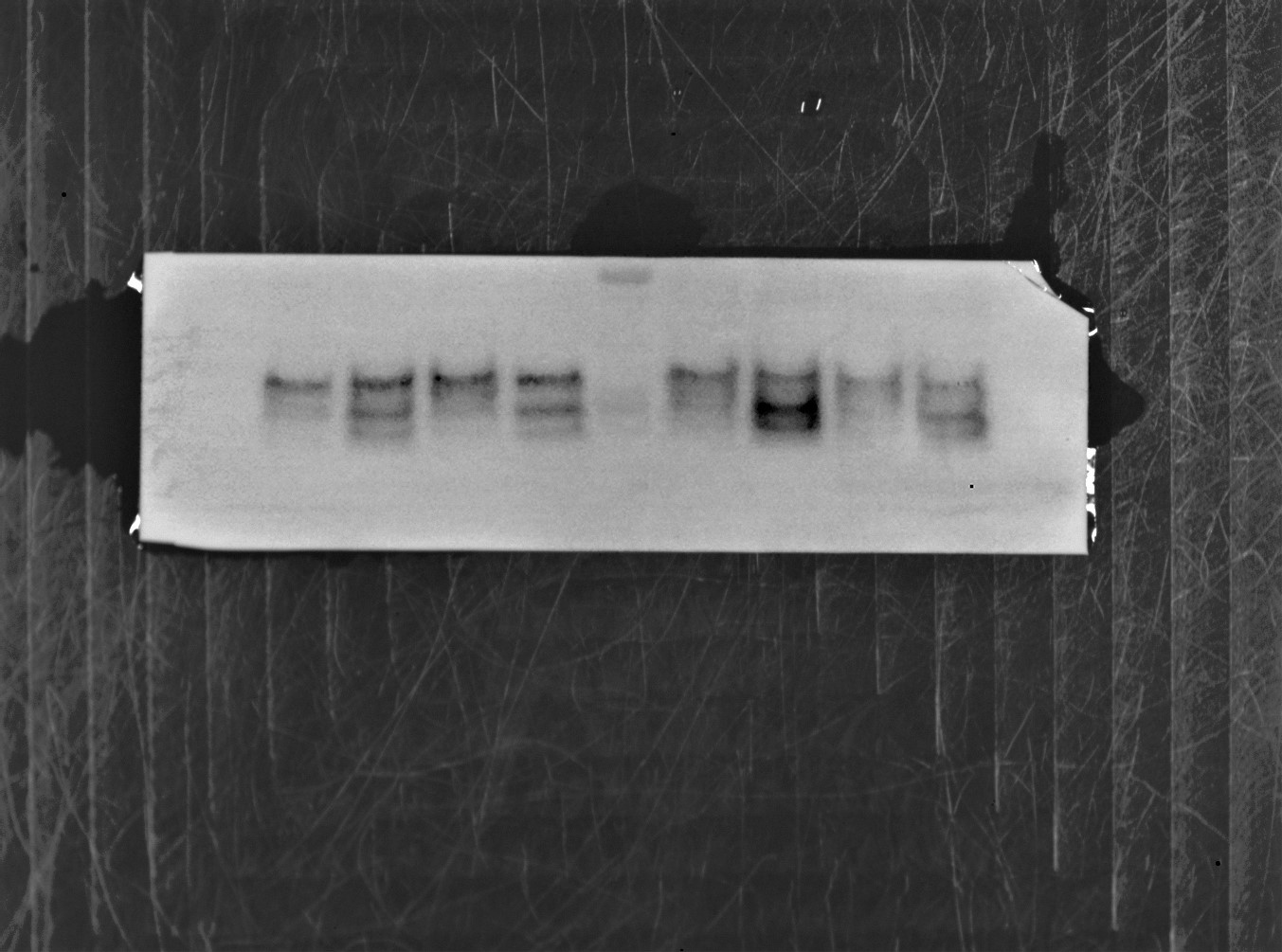

Supplement: Supplementary file 4 — Additional file 4. [file 13046_2021_2144_MOESM4_ESM.zip › WB Autophagy CPZ+PBA/TS #1/LC3 TS#1.jpg]

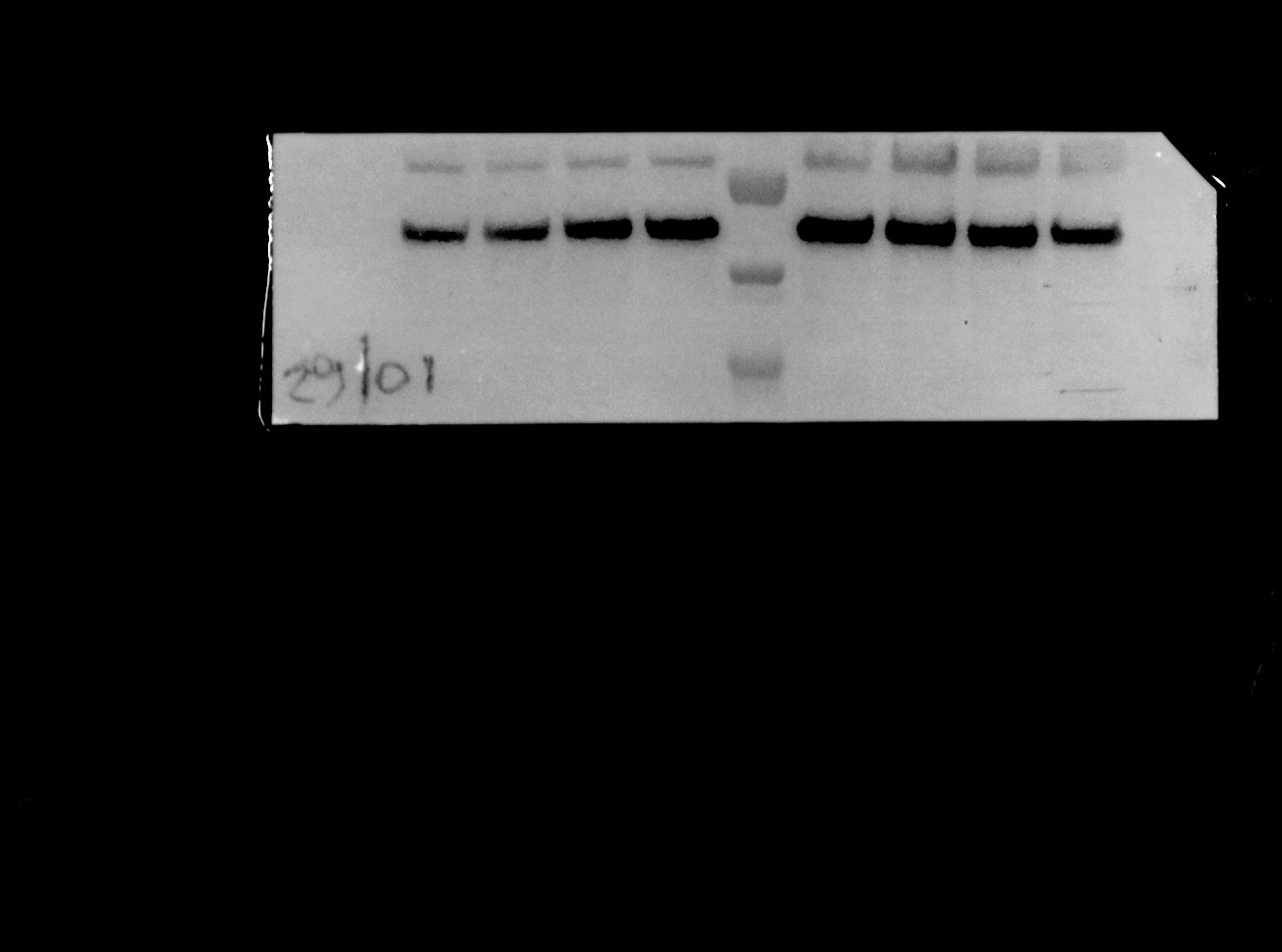

Supplement: Supplementary file 4 — Additional file 4. [file 13046_2021_2144_MOESM4_ESM.zip › WB Autophagy CPZ+PBA/TS #1/p62 TS#1.jpg]

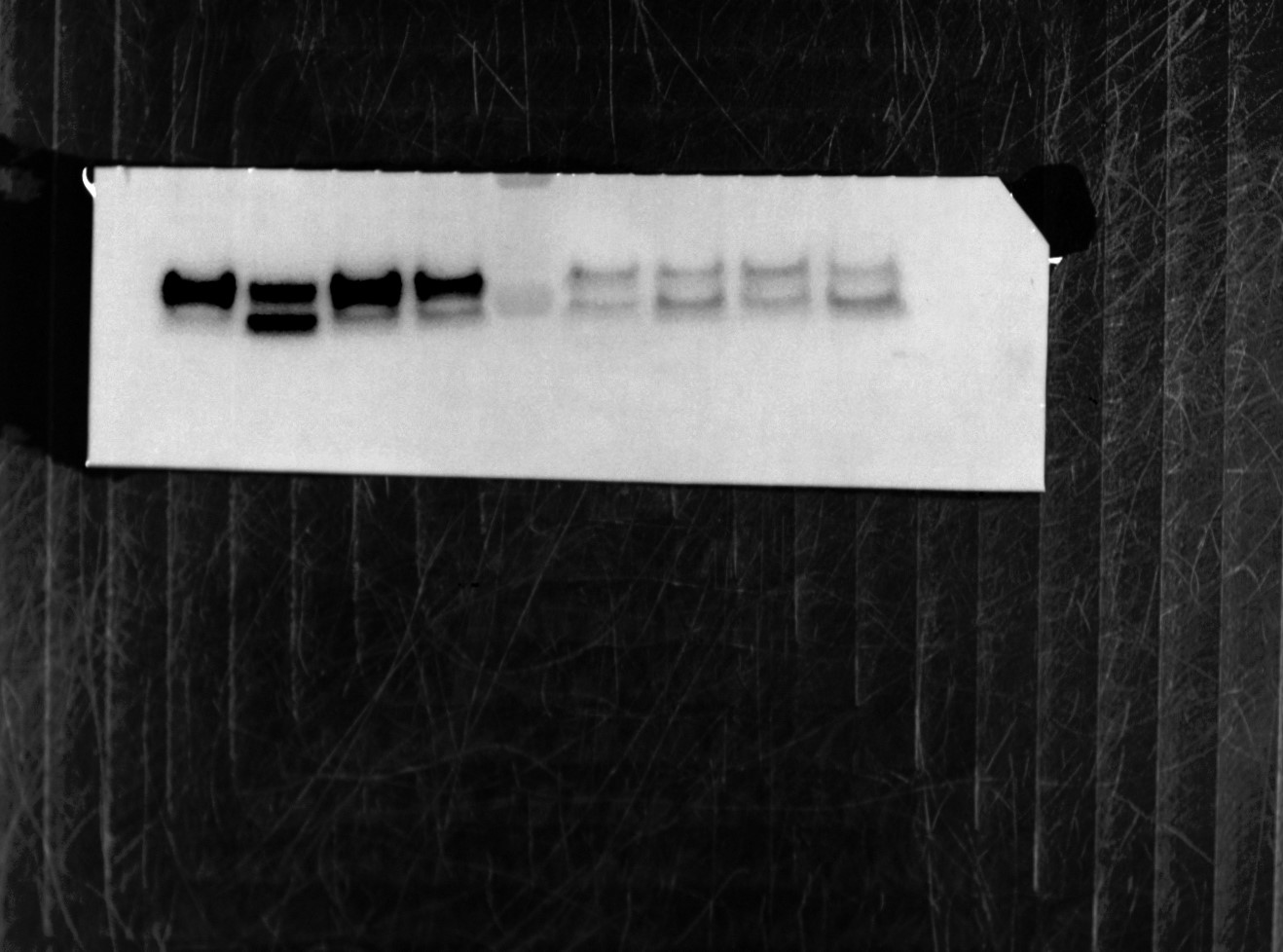

Supplement: Supplementary file 4 — Additional file 4. [file 13046_2021_2144_MOESM4_ESM.zip › WB Autophagy CPZ+PBA/TS #83/LC3 TS#83.jpg]

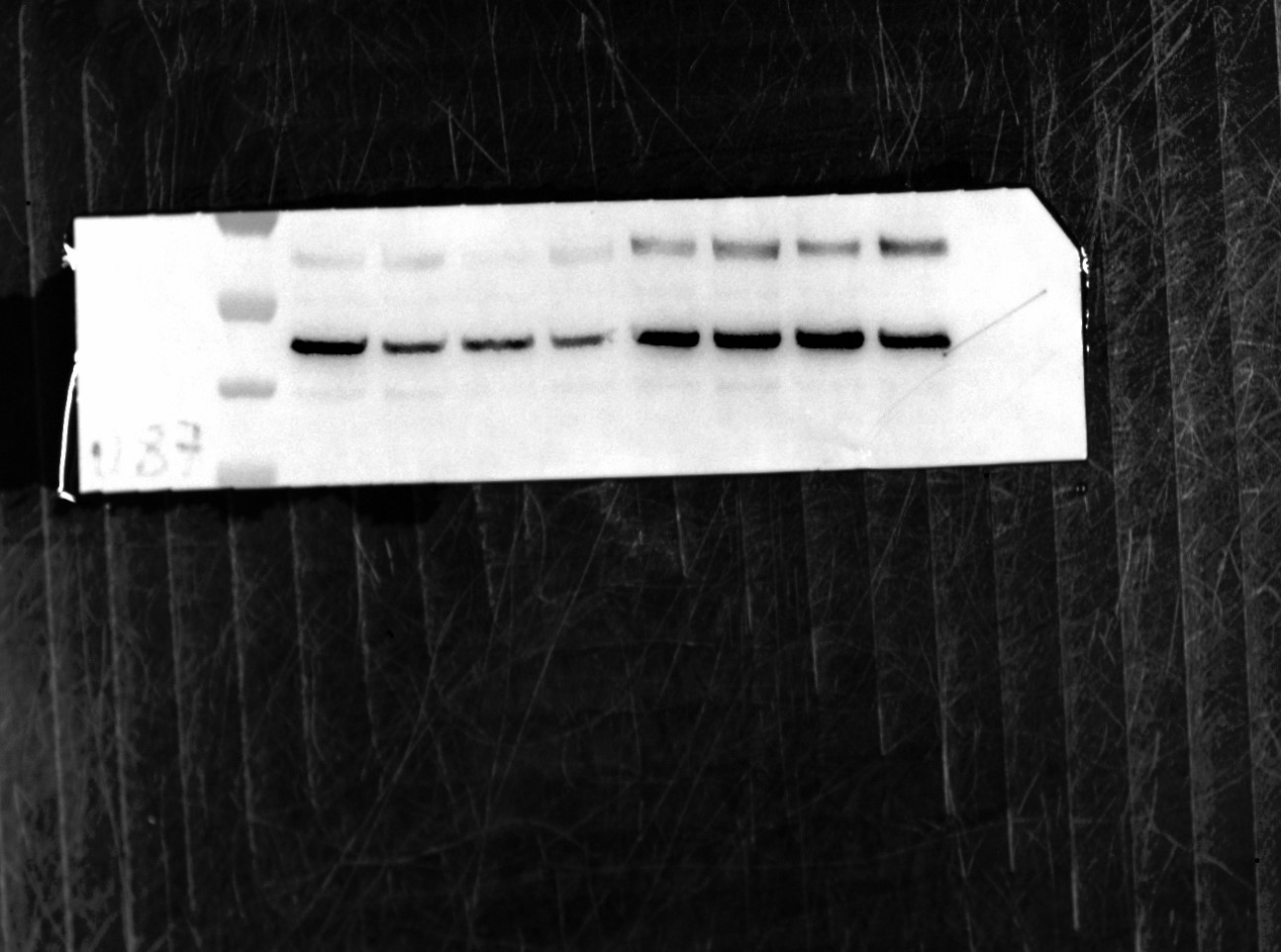

Supplement: Supplementary file 4 — Additional file 4. [file 13046_2021_2144_MOESM4_ESM.zip › WB Autophagy CPZ+PBA/U-251 MG/actina (p62) U251MG.jpg]

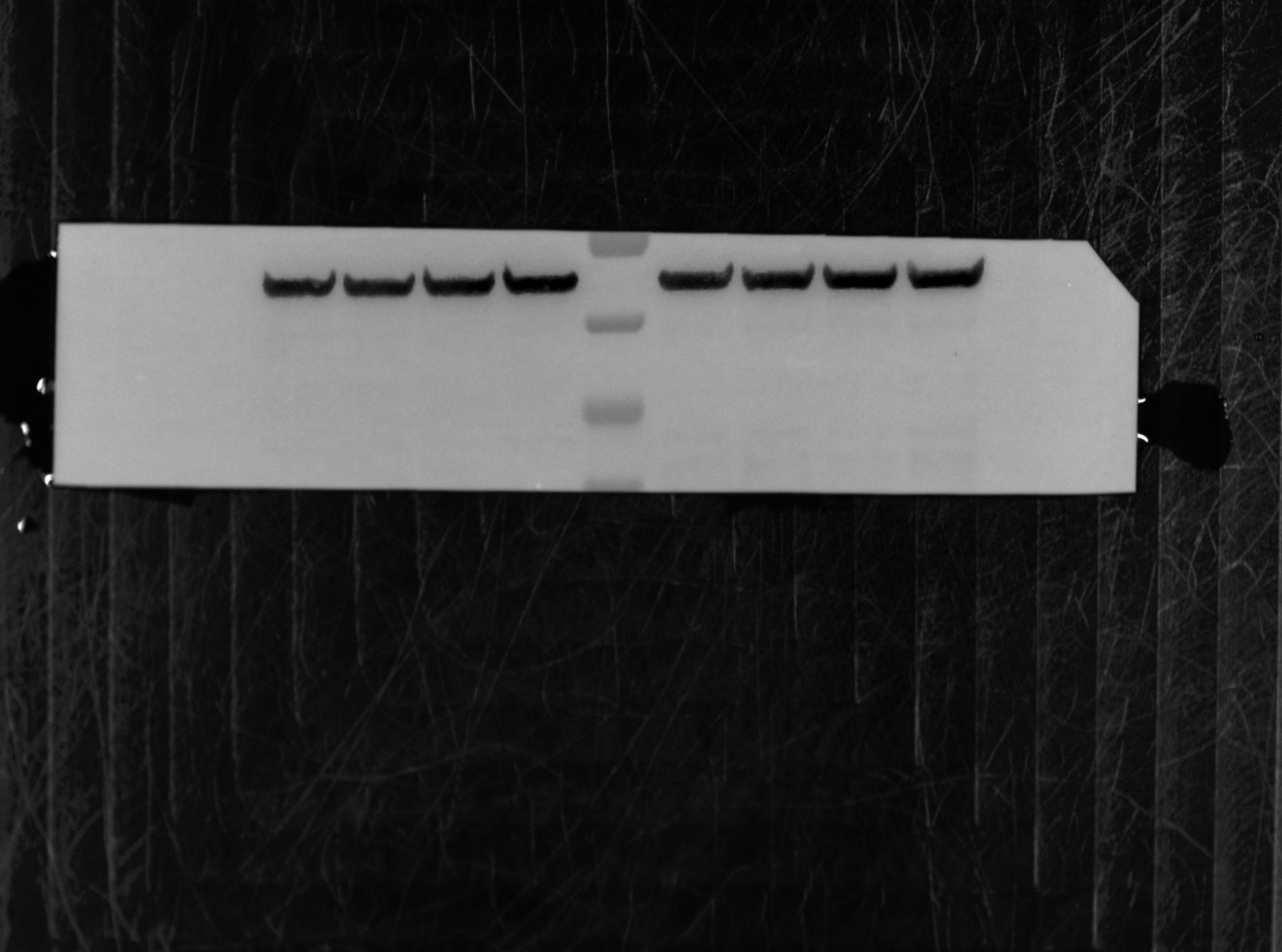

Supplement: Supplementary file 4 — Additional file 4. [file 13046_2021_2144_MOESM4_ESM.zip › WB Autophagy CPZ+PBA/U-251 MG/actina U251MG.jpg]

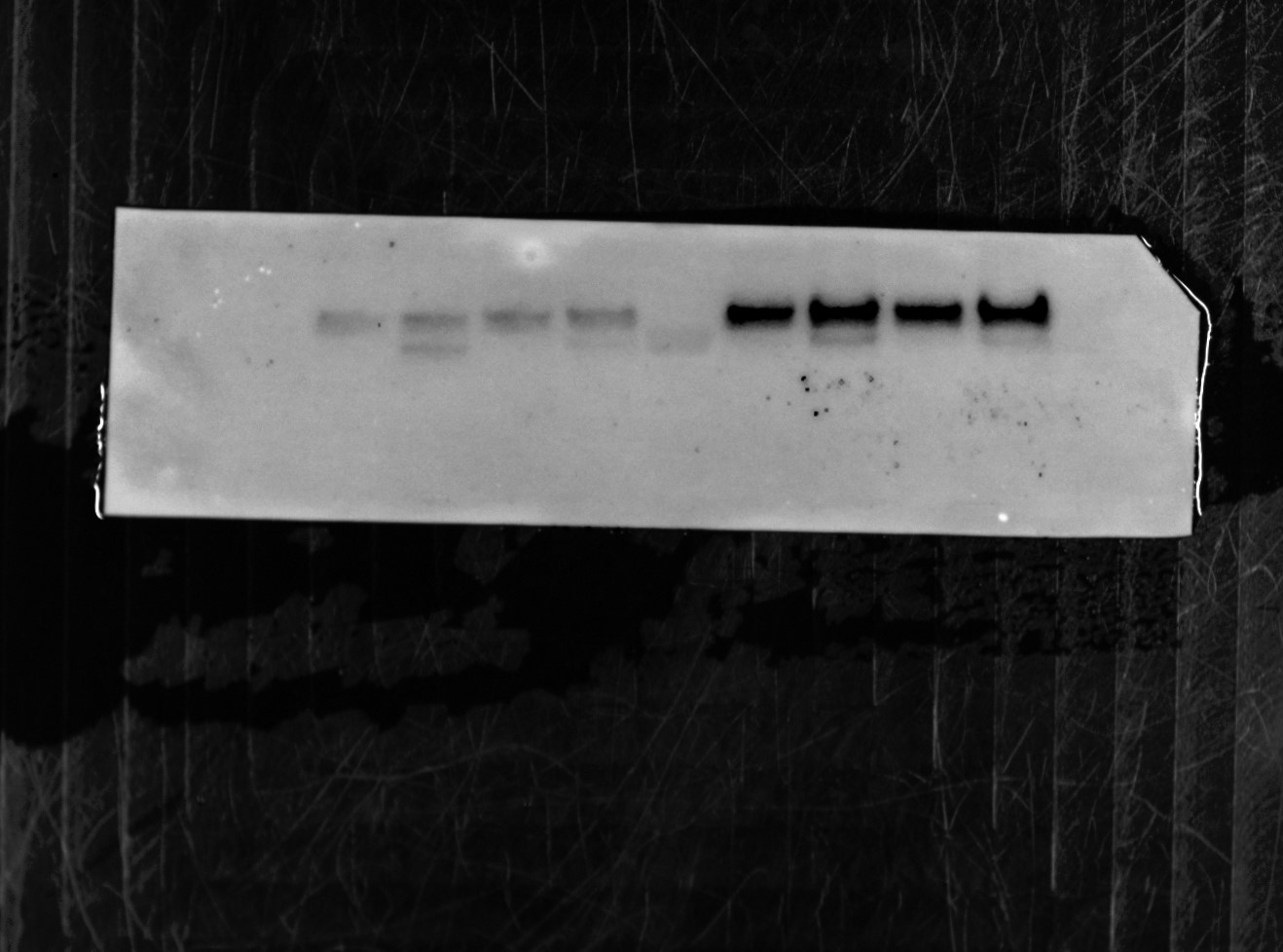

Supplement: Supplementary file 4 — Additional file 4. [file 13046_2021_2144_MOESM4_ESM.zip › WB Autophagy CPZ+PBA/U-251 MG/LC3 U251MG.jpg]

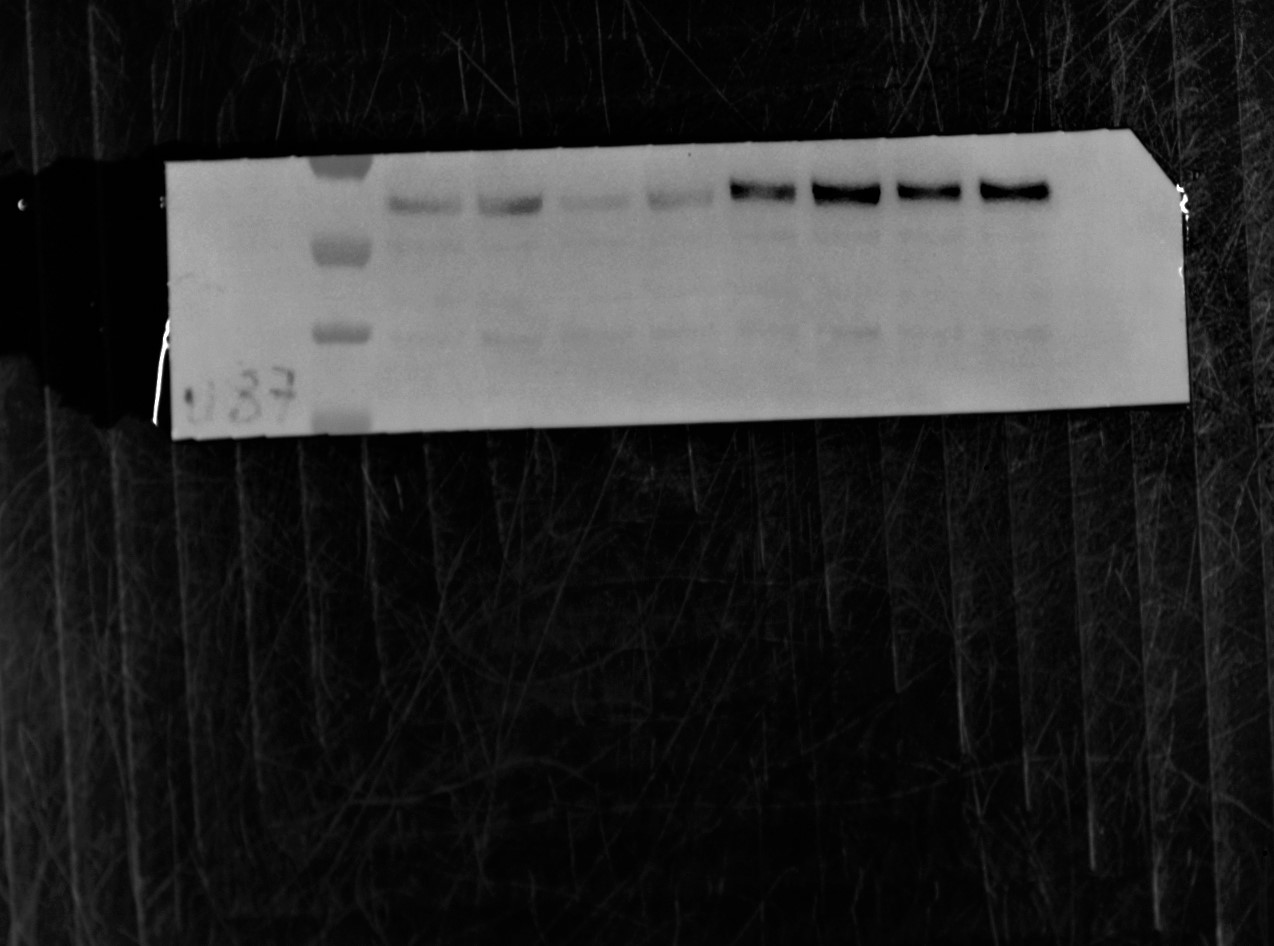

Supplement: Supplementary file 4 — Additional file 4. [file 13046_2021_2144_MOESM4_ESM.zip › WB Autophagy CPZ+PBA/U-251 MG/p62 U251MG.jpg]

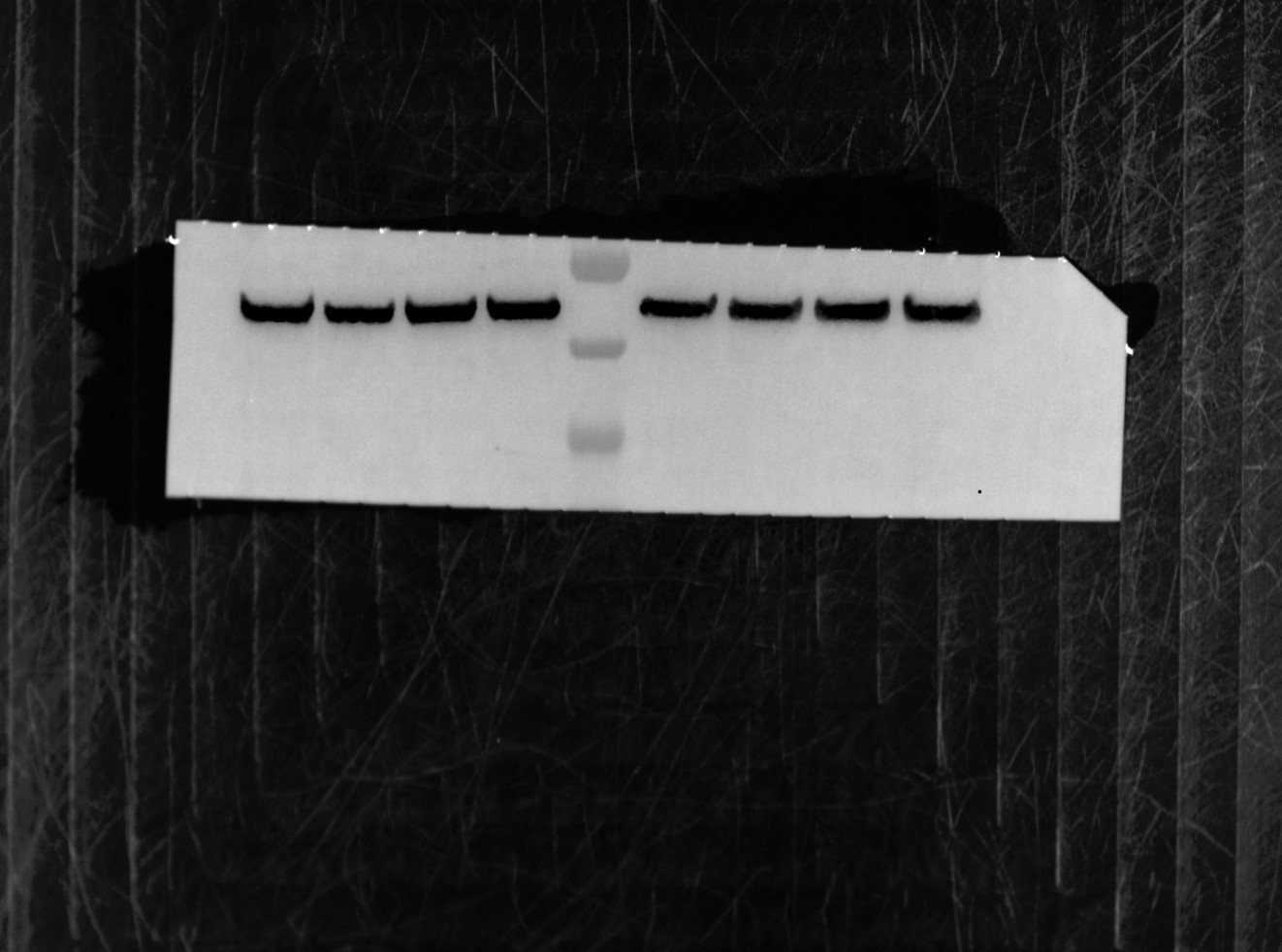

Supplement: Supplementary file 4 — Additional file 4. [file 13046_2021_2144_MOESM4_ESM.zip › WB Autophagy CPZ+PBA/U-87 MG/actina U87MG.jpg]

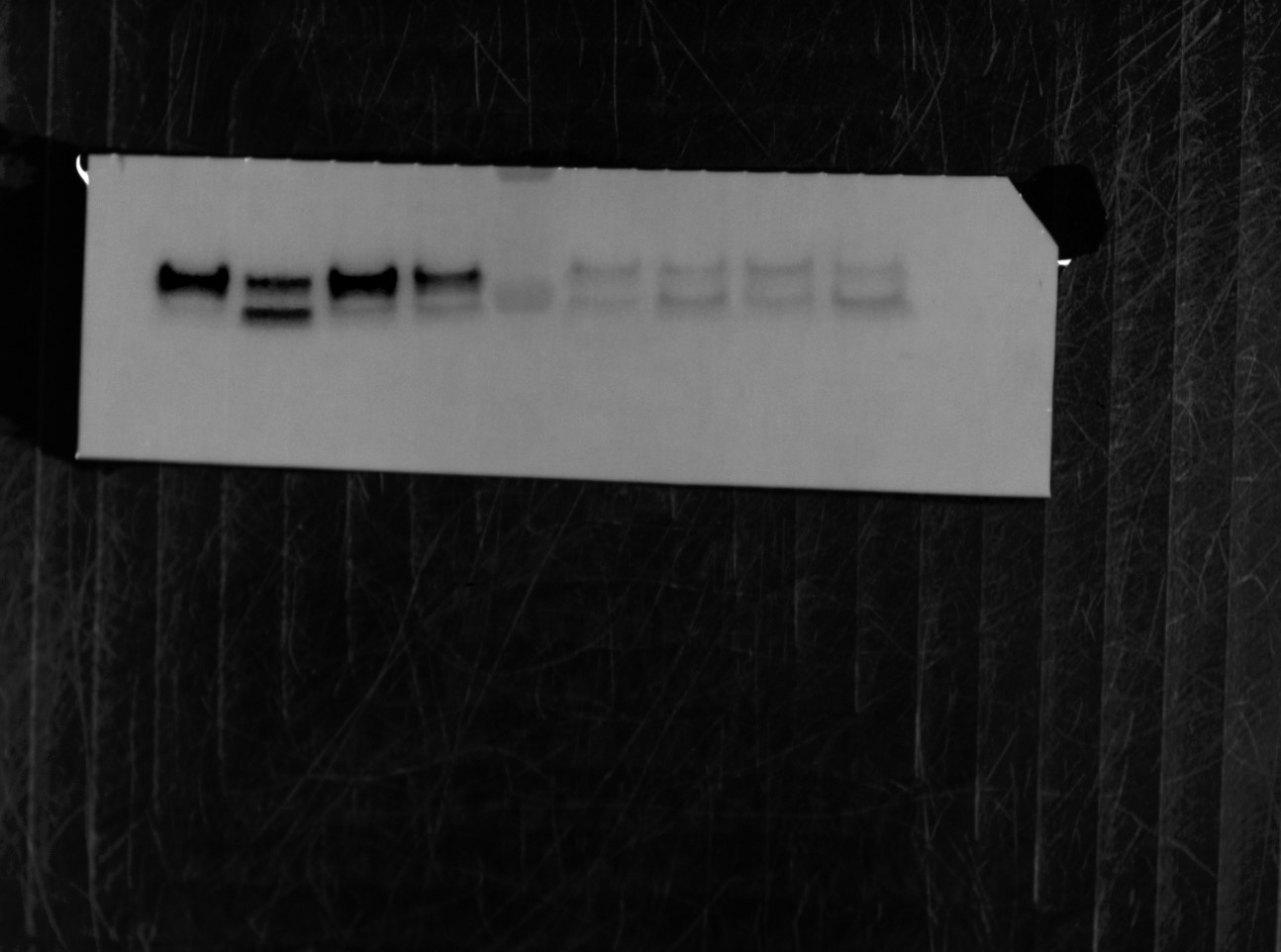

Supplement: Supplementary file 4 — Additional file 4. [file 13046_2021_2144_MOESM4_ESM.zip › WB Autophagy CPZ+PBA/U-87 MG/LC3 U87MG.jpg]

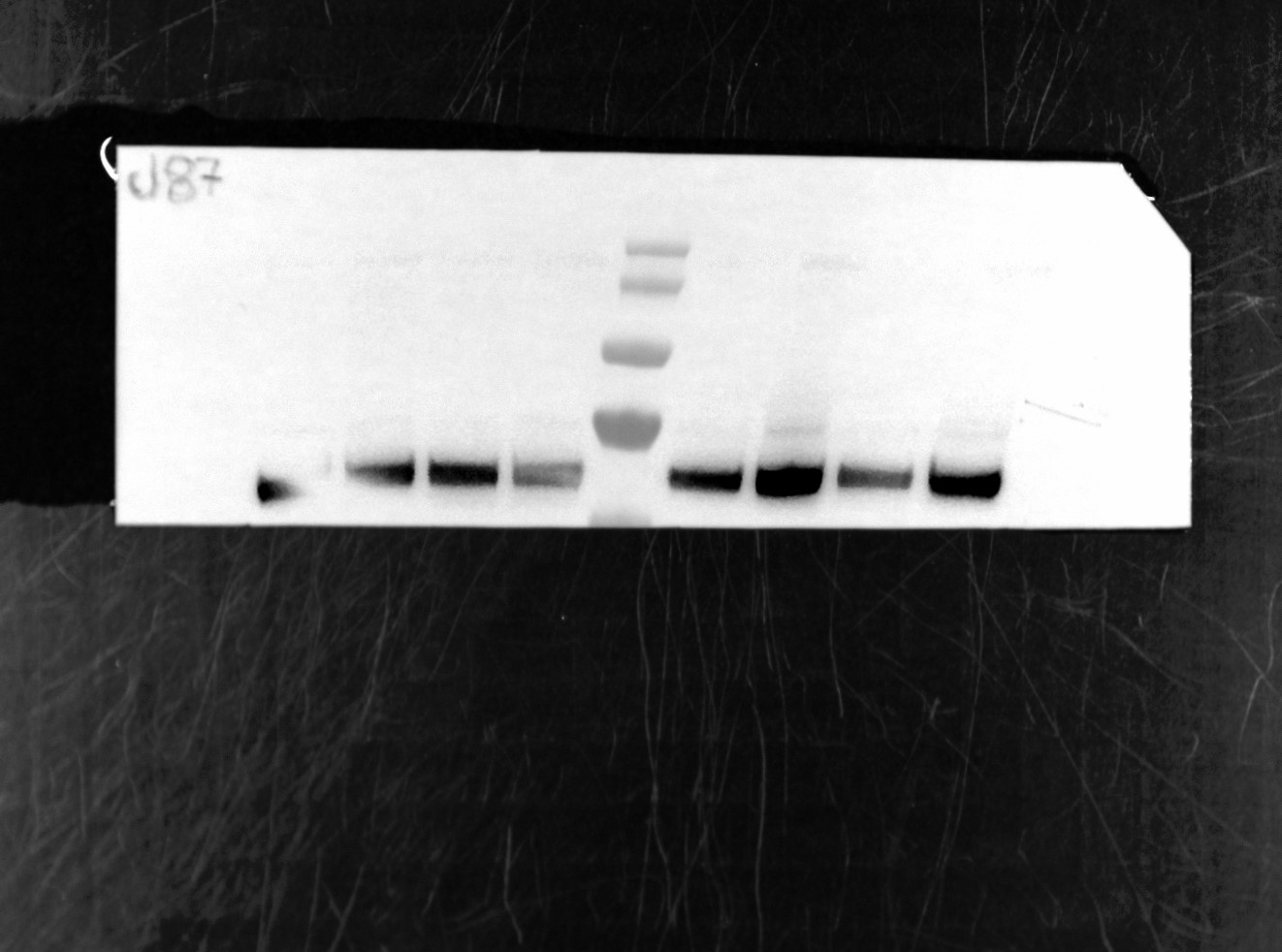

Supplement: Supplementary file 4 — Additional file 4. [file 13046_2021_2144_MOESM4_ESM.zip › WB Autophagy CPZ+PBA/U-87 MG/p62 U87MG.jpg]
