# Supplementary material for: Chlorpromazine induces cytotoxic autophagy in glioblastoma cells via endoplasmic reticulum stress and unfolded protein response
Source: J Exp Clin Cancer Res. 2021 Nov 5;40:347. doi: 10.1186/s13046-021-02144-w (PMC8569984; doi:10.1186/s13046-021-02144-w)
Supplement: Supplementary file 7 — Additional file 7: Table S1. CPZ IC30 values for each of the cell lines utilized. They represent the drug concentrations able to inhibit the cell growth by 30% after 48 hours of exposure. [file 13046_2021_2144_MOESM7_ESM.docx]

**Supplementary Information**

**Table S1**

CPZ IC30 values for each of the cell lines utilized. They represent the drug concentrations able to inhibit the cell growth by 30% after 48 hours of exposure.

| **Cell Line** | **CPZ IC30 (μM)** |  |
| --- | --- | --- |
| T98G | 7.6 ± 0.3 | Anchorage-dependent cells |
| U-87 MG | 7.4 ± 1.2 |  |
| U-251 MG | 7.0 ± 1.1 |  |
| RPE-1 | 10.4 ± 1.1 |  |
| TS#1 | 14.8 ± 1.0 | Neurospheres |
| TS#83 | 13.4 ± 1.4 |  |
| TS#163 | 10.9 ± 1.2 |  |

**Additional files**

Additional files and raw data are available at the following link:
<https://gbox.garr.it/garrbox/index.php/s/3oWbwZFWAdyvSxO>
